# Potent β-lactam-based tyrosyl-DNA phosphodiesterase 1 inhibitors identified by a virtual screen

**DOI:** 10.1038/s41598-025-12503-8

**Published:** 2025-07-21

**Authors:** Xue Zhi Zhao, Wenjie Wang, Kiall F. Suazo, Md Rasel Al Mahmud, Keli Agama, George T. Lountos, Thorkell Andresson, Yves Pommier, Terrence R. Burke

**Affiliations:** 1 Chemical Biology Laboratory, Center for Cancer Research, National Cancer Institute, National Institutes of Health, Frederick, MD USA; 2 Developmental Therapeutics Branch & Laboratory of Molecular Pharmacology, Center for Cancer Research, National Cancer Institute, National Institutes of Health, Bethesda, MD USA; 3 Protein Characterization Laboratory, Cancer Research Technology Program, Frederick National Laboratory for Cancer Research, Frederick, MD USA; 4 Basic Science Program, Frederick National Laboratory for Cancer Research, Frederick, MD USA

**Keywords:** Tyrosyl-DNA phosphodiesterase 1 (TDP1) inhibitors, β-Lactam, Virtual screen, DrugBank, Fluorescence assay, Surface plasmon resonance, Chemical biology, Drug discovery

## Abstract

Tyrosyl-DNA phosphodiesterase 1 (TDP1) is an important therapeutic target. We recently reported several crystal structures of TDP1 with small molecules bound within the catalytic pocket. These molecules bind by forming hydrogen bonds with residues of the catalytic HKN motifs. Guided by these interactions, in our current work, we used the MolSoft ICM Pro suite of software to perform a virtual screen of the publicly available DrugBank 5.0 (3449 structures) for the ability to bind to the TDP1 catalytic pocket. Among compounds identified as giving good binding scores were several β-lactams. The β-lactam pharmacophore serves as a key component in a range of antibiotics. We subjected a subset of the β-lactam hits to gel-based TDP1 fluorescence catalytic assays and established that certain members showed micromolar TDP1 inhibition. In follow-up, we evaluated a commercially available library of 90 β-lactam antibiotics. This led to our identification of additional β-lactams having micromolar TDP1 inhibitory potencies. In particular, cephalosporin C showed single-digit micromolar TDP1 IC_50_ values. Since β-lactams can form covalent bonds with serine residues in target penicillin-binding proteins (PBPs), we performed docking studies with cephalosporin C, which showed that it bound within the catalytic pocket and extended into the DNA substrate binding channel. Importantly, the modeling indicated that both noncovalent and covalent binding modes were theoretically possible. Surface plasmon resonance analysis demonstrated its non-covalent binding mode. Thus, β-lactams may serve as a new and potentially useful platform to design TDP1-binding ligands that interact with the catalytic pocket and extend into the DNA substrate binding channel.

**Supplementary Information:**

The online version contains supplementary material available at 10.1038/s41598-025-12503-8.

## Introduction

Tyrosyl-DNA phosphodiesterase 1 (TDP1) is a key enzyme involved in repairing a broad spectrum of DNA lesions, including those resulting from the actions of anticancer drugs^1,2^. Human TDP1 is comprised of a 68-kDa polypeptide of 608 amino acid residues. Its C-terminal domain shows that TDP1 belongs to the phospholipase D (PLD) family, whose catalytic machinery is characterized by two conserved HKN motifs (H263/K265/N283 and H493/K495/N516) in close proximity within a substrate-binding channel^3,4^. TDP1 catalyzes the hydrolysis of substrate DNA 3’- phosphodiesters without involvement of cofactors or metal ions^5–11^. TDP1 substrates embrace a growing list of small DNA adducts, including oxidized nucleotides and non-canonical nucleoside analogs as well as failed Schiff base reactions such as it can occur between PARP1 and DNA^12^. TDP1 hydrolyzes phosphodiester substrates by a two-step acid/base nucleophilic mechanism^3,7,13^. The first nucleophilic attack on the phosphate moiety is carried out by H263 to form a covalent enzyme–DNA complex. The second nucleophilic attack employs a water molecule activated by H493 to hydrolyze the intermediate phosphonamide adduct and regenerate the active site^4^. An important substrate of TDP1 arises from stalled topoisomerase type I (TOP1)-DNA covalent complexes (TOP1ccs) produced when TOP1 cleaves DNA^14,15^. By hydrolyzing the resulting single-strand 3’-adducts, TDP1 reverses TOP1-DNA lesions and in so doing, reduces the effectiveness of TOP1 inhibitors. Antagonizing this repair mechanism by TDP1 inhibitors could increase the anticancer efficacy of TOP1 inhibitors. As such, TDP1 inhibitors potentially represent a new therapeutic class of sensitizing anticancer agents^11,15–19^.

**Fig. 1:**
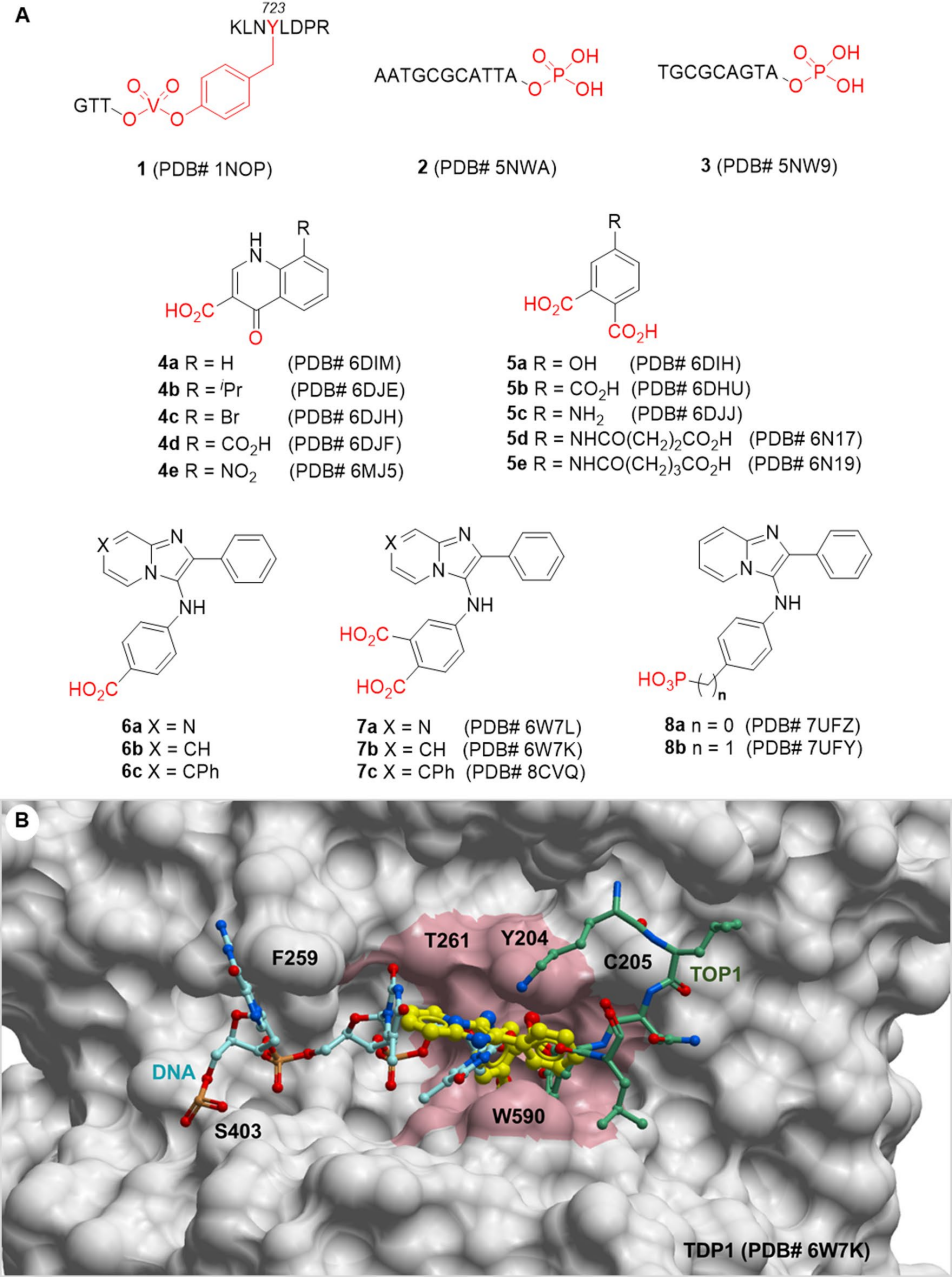
Structures of TDP1-bound small molecules and substrate mimetics and their binding surface. (**A**) Structures of TDP1 binding DNA substrate mimetics and small molecules in TDP1 cocrystals with Protein Data Bank accession numbers indicated. Phosphate and its mimetics are highlighted in red; (**B**) Binding area proximal to TDP1-bound **7b** (carbon bonds in yellow) (TDP1-XZ634p complex, PDB code: 6W7K) overlaid with the DNA-vanadate-TOP1 complex (**1**) (PDB code: 1NOP). The binding pocket of **7b** is shown in pink. The DNA-vanadate-TOP1 substrate complex (**1**) is overlaid for comparison. Carbon bonds of DNA in cyan and carbon bonds of TOP1 peptide are shown in green.

X-ray crystal structures reveal that DNA substrate mimetics (**1–3**) bind within the catalytic site of TDP1 by forming hydrogen bonds with the catalytic HKN motifs (Fig. 1A)^6,20^. The relatively shallow catalytic pocket and the open substrate binding channels have hampered the rapid development of efficient small-molecule inhibitors (Fig. 1B)^6,20^. Recently, we employed an X-ray crystallography screen to identify several TDP1-bound small-molecule quinolone and phthalic acid scaffolds (**4a-e** and **5a-e**, respectively, Fig. 1A)^21^. To follow-up, we applied small molecule microarray and oxime-based diversification strategies to develop imidazopyridine-based derivatives (**6a-c** and **7a-c**)^22,23^ and phosphates (**8a**,** b**)^24^. Structures of these TDP1-bound inhibitors inform interactions of small molecules within the TDP1 catalytic site and suggest possible ways in which these molecules could potentially compete with the DNA-TOP1 peptide substrate (Fig. 1B)^21–24^. However, the extended nature of the protein-DNA substrate and the way in which the substrate lays along an open binding groove have made it challenging to achieve effective inhibition using small molecules.

Our current work used molecular recognition features of our TDP-bound inhibitors identified by the MolSoft suite of ICM modeling software to perform a virtual screen of the publicly available DrugBank 5.0 (3449 structures)^25–27^. This database has proven to be a useful resource for in silico drug discovery^28^. The search led to our finding that a series of β-lactam hits, including cephalosporin C, show single-digit micromolar inhibitory potency against TDP1 in *in vitro* catalytic TDP1 inhibition assays. This prompted us to screen a commercially available library of approximately 90 β-lactams that resulted in the identification of additional β-lactams showing TDP1 inhibition. Molecular docking indicates that the β-lactams preferentially bond within the narrow and positively charged DNA substrate-binding pocket, such that the reactive lactam carbonyl groups are positioned in orientations that could potentially make them susceptible to nucleophilic ring opening by the S400 residue. However, surface plasmon resonance (SPR) analysis of cephalosporin C binding to TDP1 protein was also consistent with non-covalent interactions.

## Results and discussion

### DrugBank virtual screen

**Fig. 2:**
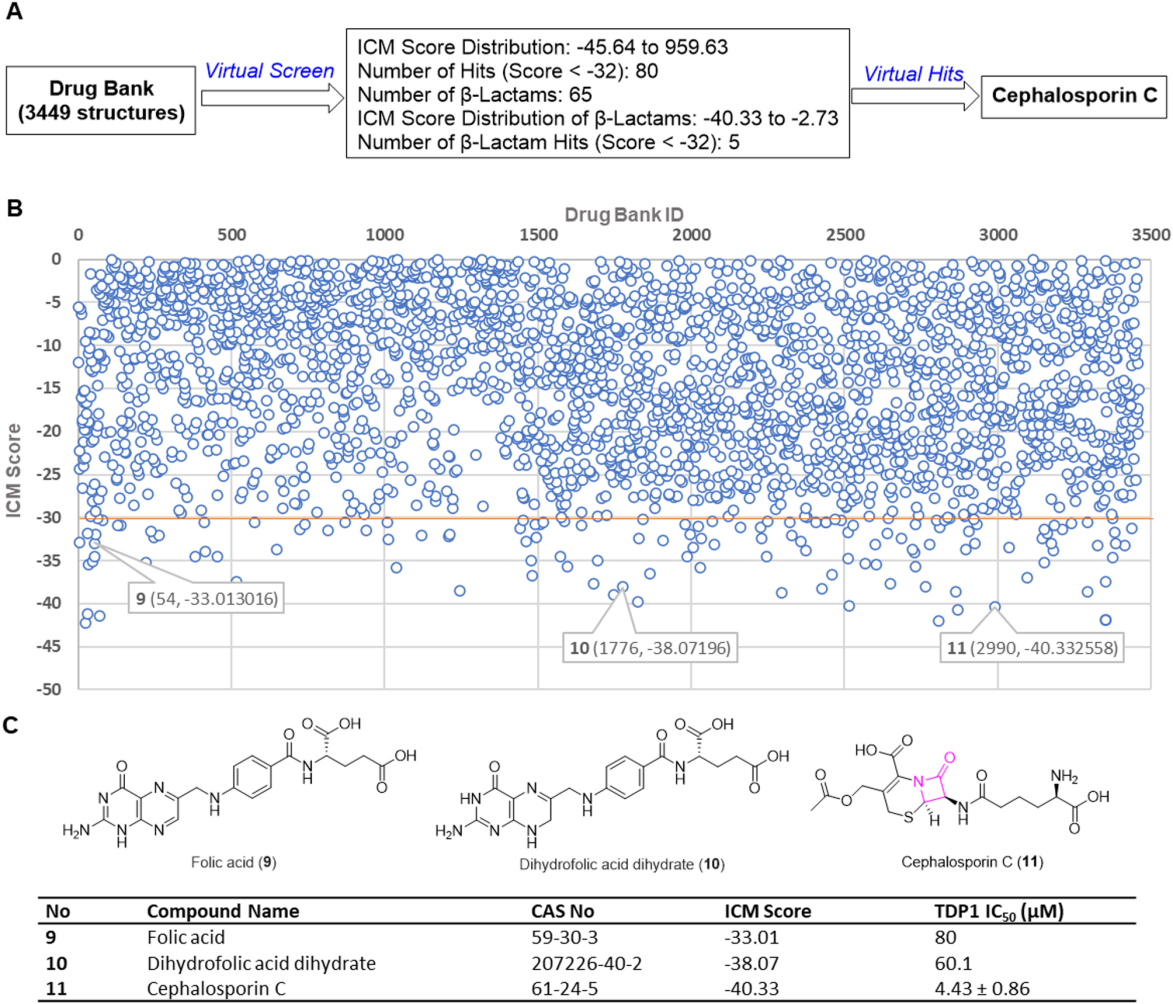
Results of MolSoft ICM virtual DrugBank screen. (**A**) Schematic overview of the DrugBank virtual screen (Table S1); (**B**) Map of DrugBank IDs with related ICM scores and structures of three select hits (labeled in the map). The distribution of ICM scores ranged from − 45.64 to 959.63. The y-axis is truncated at 0 to clarify presentation; (C) Structures of select hits and their TDP1 inhibitory potencies based on gel-based TDP1 fluorescence catalytic assays.

We performed a virtual screen of the DrugBank 5.0 library of 3449 structures (Table S1). We employed the MolSoft ICM Pro docking software suite and utilized our previously reported X-ray crystal small molecule **7b** (XZ634p) bound within the catalytic pocket of 1-148 amino terminal truncated TDP1 (149–608) (PDB code: 6W7K) (Fig. 2A)^22,23^. The screen employed ICM scoring weighted according to: (i) internal force-field energy of the ligand, (ii) entropy loss of the ligand between bound and unbound states, (iii) ligand-receptor hydrogen bond interactions, (iv) polar and non-polar solvation energy differences between bound and unbound states, (v) electrostatic energy, (vi) hydrophobic energy, and (vii) hydrogen bond donor or acceptor desolvation. A lower ICM score predicts better binding^29,30^. Structures with ICM scores of −32 and lower are generally considered to be potentially promising leads^30^. We found that the ICM binding scores ranged from − 45.64 to 959.63 with eighty compounds scoring lower than − 32 (Fig. 2B). Among representative commercially available molecules with better binding scores were folic acid (**9**, DrugBank ID: 1776), dihydrofolic acid dihydrate (**10**, DrugBank ID: 54) and the β-lactam cephalosporin C (**11**, DrugBank ID: 2990) (labeled in the Score map in Fig. 2B).

### Biological evaluation of β-lactams

**Fig. 3:**
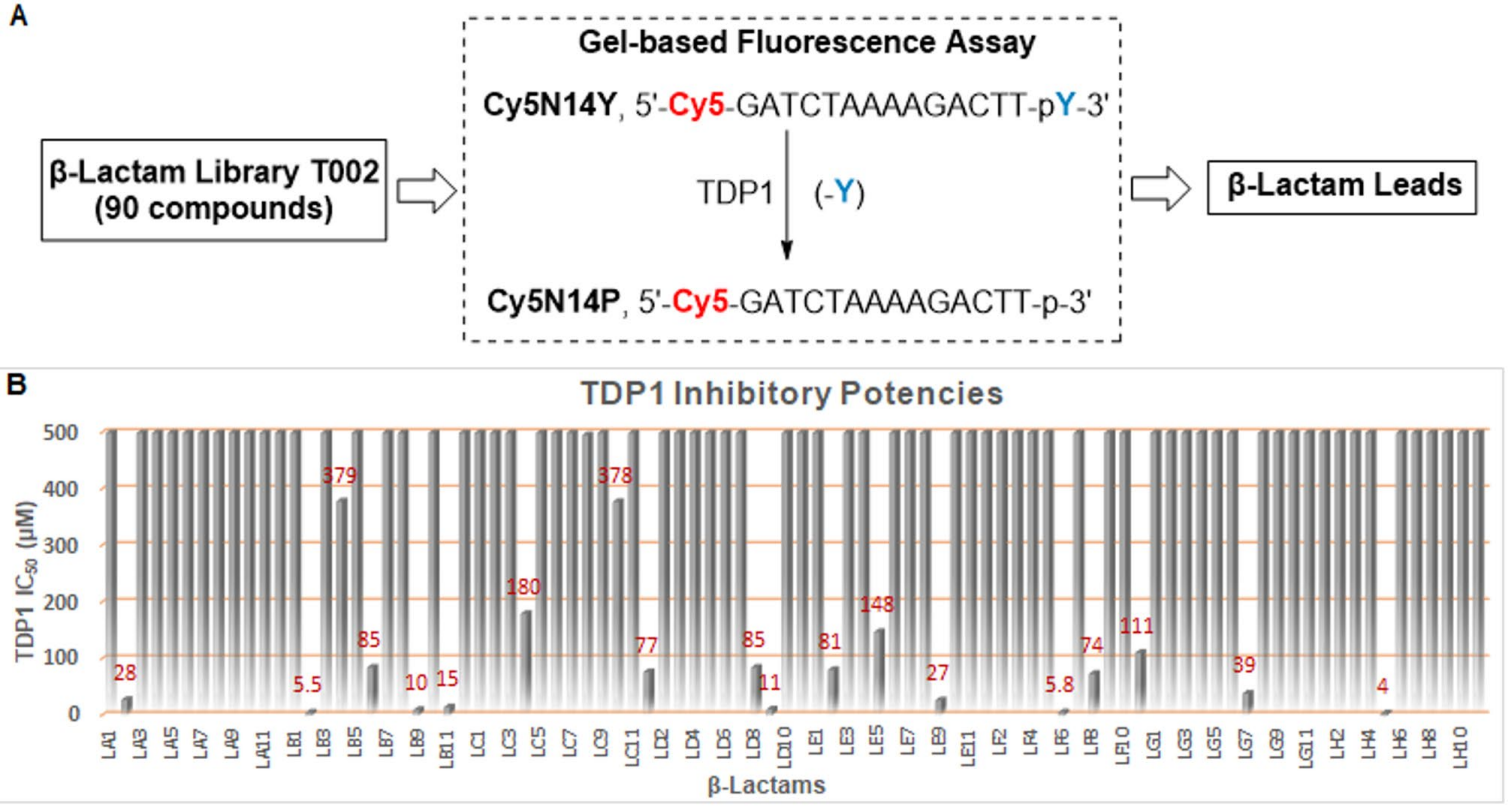
Evaluation of TDP1 inhibitory potencies of a library of commercial β-lactams. (**A**) Schematic overview of the biologic assay used to screen the β-lactam library; (**B**) Bar graph plots showing TDP1 inhibitory potencies of the β-lactams (maximum concentrations tested were 1000 µM as shown in Table S2). For clarity y-axis values were truncated at 500 µM. Among the 19 leads that exhibited micromolar TDP1 inhibition (highlighted in red), 14 β-lactams demonstrated IC₅₀ values below 100 µM.

We subjected select virtual screen hits to evaluation of TDP1 inhibitory potencies using *in vitro* gel-based fluorescence TDP1 catalytic assays (Fig. 2). Three compounds showed micromolar TDP1 inhibitory potencies (**9**, IC_50_ = 80 µM; **10**, IC_50_ = 60 µM and **11**, IC_50_ = 4.4 µM) (Fig. 2C). Among β-lactam members of the DrugBank, a total of 65 structures showed ICM scores from − 40.33 to −2.73, with five β-lactams having scores lower than − 32. To further explore this class of compounds, we purchased from TargetMol a library of approximately 90 β-lactams as 10 mM solutions in DMSO. The library included a selection of penicillin derivatives (penams), cephalosporins (cephems), monobactams, carbapenems and carbacephems (numbered as **LA1-LA12**,** LB1-LB12**,** LC1-LC11**,** LD1-LD11**,** LE1-LE11**,** LF1-LF11**,** LG1-LG11**, and **LH1-LH11** according to their location in a 96-well plate; see Table S2). We examined TDP1 inhibitory potencies of the compounds using the *in vitro* gel-based fluorescence assays as previously described^21–24^. Briefly, a 5’-Cy5-labeled DNA substrate (N14Y, 5’-Cy5-GATCTAAAAGACTT-pY-3’, 1 nM) was incubated in TDP1 reaction buffer with recombinant TDP1 or truncated TDP1(148–608) (40 pM) in the absence or presence of inhibitors for 15 min at room temperature. Inhibitors were evaluated at concentrations with 3-fold serial dilution of drugs ranging from 0.457 µM to 1 mM. The TDP1 inhibition IC_50_ values were calculated based on gel images of the cleavage product (N14P, 5’-Cy5-GATCTAAAAGACTT-p-3’) (Fig. 3A and Table S2). Although the majority of compounds showed poor TDP1 inhibitory potencies (TDP1 IC_50_ > 100 µM, see Fig. 3B), fourteen compounds showed low micromolar potencies (TDP1 IC_50_ < 100 µM). These compounds included compounds **LA2**,** LB2**,** LB6**,** LB9**,** LB11**,** LD1**,** LD8**,** LD9**,** LE2**,** LE9**,** LF6**,** LF8**,** LG7** and **LH5** (labeled in red in Fig. 3B). The most potent analogues (**LH5**, **LB2**, and **LF6**) showed single-digit micromolar TDP1 inhibitory potencies (IC_50_ = 4.0 µM, 5.5 µM, and 5.8 µM, respectively) (Fig. 3B).

### Relative inhibitory selectivity for TDP1 relative to TDP2

**Fig. 4:**
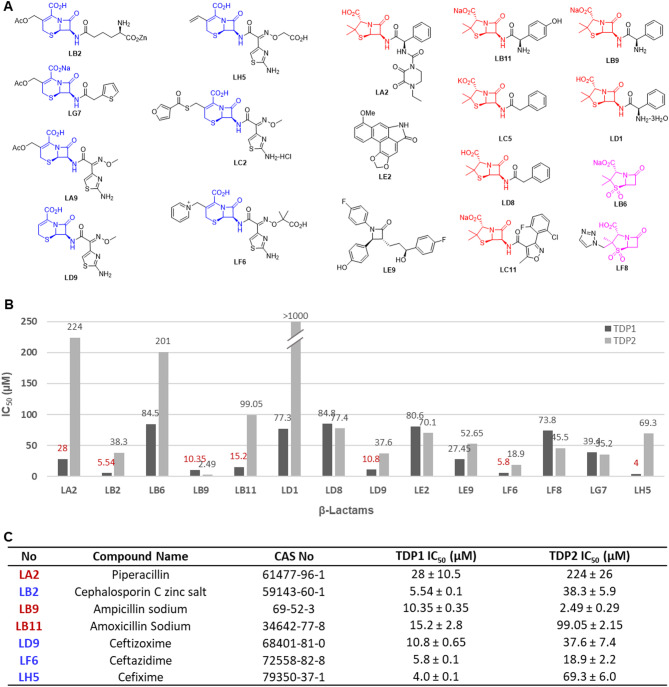
Inhibitory selectivity of β-lactams for TDP1 relative to TDP2. (**A**) Structures of lead compounds, including cephams (highlighted in blue) and penams (highlighted in red); (**B**) Comparison of inhibitory potencies against TDP1 and TDP2 using *in vitro* gel-based fluorescence assays; (**C**) Tabulation of inhibitory potencies of lead β-lactams against TDP1 and TDP2.

Both TDP1 and TDP2 are DNA repair enzymes that resolve covalent DNA-protein crosslinks, particularly those involving topoisomerases^31,32^. TDP2 repairs double-strand DNA breaks (DSBs) by cleaving 5’-phosphotyrosyl linkages between the DNA 5’-end and a tyrosine residue in TOP2ccs, and it requires divalent metal ions (e.g., Mg²⁺ or Mn²⁺) for its activity. In contrast, TDP1 repairs single-strand DNA breaks (SSBs) by cleaving 3’-phosphotyrosyl linkages between the DNA 3’-end and a tyrosine residue in TOP1ccs. TDP1 utilizes histidine-based catalysis and does not require metal ions for its enzymatic function. Selective inhibition of TDP1 or TDP2 is important and depends on the therapeutic strategy. Inhibiting TDP1 can enhance the efficacy of TOP1 poisons, while targeting TDP2 may help overcome resistance to TOP2 inhibitors. Additionally, dual inhibition of both TDP1 and TDP2 could also be advantageous due to their complementary roles in repairing topoisomerase-induced DNA damage. Two primary classes of β-lactams showing potent TDP1 inhibition (TDP1 IC_50_ < 100 µM) were cephams and penams (shown in blue and red, respectively in Fig. 4A). As counterscreen, we employed gel-based fluorescence *in vitro* assays to examine the inhibitory potencies against TDP2 (these were modified relative to the TDP1 assays described above, wherein recombinant TDP1 and DNA substrate were replaced with recombinant TDP2 at 40 pM) (Fig. 4B and Table S3)^33^. With the exception of **LB9**, which showed 4-fold greater potency against TDP2 than TDP1, the β-lactams displayed from 3-fold to 17-fold greater potency against TDP1 than against TDP2 (Fig. 4C). The zinc salt of cephalosporin C (**LB2)** retained single-digit micromolar TDP1 inhibitory potency (TDP1 IC_50_ = 5.5 µM). This was approximately 7-fold more potent than TDP2. The TDP1 inhibitory potencies are consistent with cephalosporin C (**11**).

### Noncovalent and covalent docking modes of cephalosporin C

**Fig. 5:**
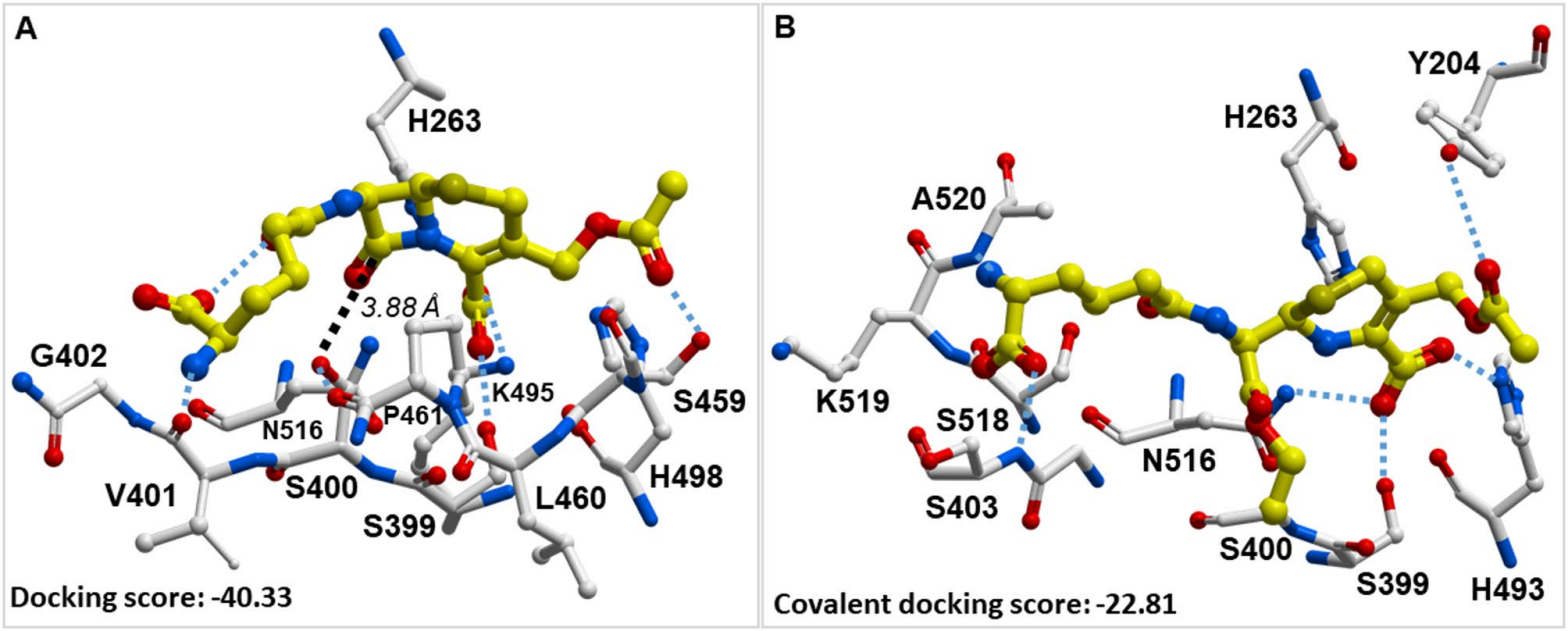
Docking of cephalosporin C in TDP1 based on the structure of the TDP1-XZ634p complex (PDB code: 6W7K). (**A**) Structure of TDP1 noncovalently bound to cephalosporin C (carbon bonds in yellow) within the DNA substrate binding pocket (carbon bonds in grey). Hydrogen bonds are shown as cyan dashed lines; (**B**) Structure of TDP1 covalently bound cephalosporin C (carbon bonds in yellow) within the DNA substrate binding pocket (carbon bonds in grey). Hydrogen bonds are shown as cyan dashed lines.

**Fig. 6:**
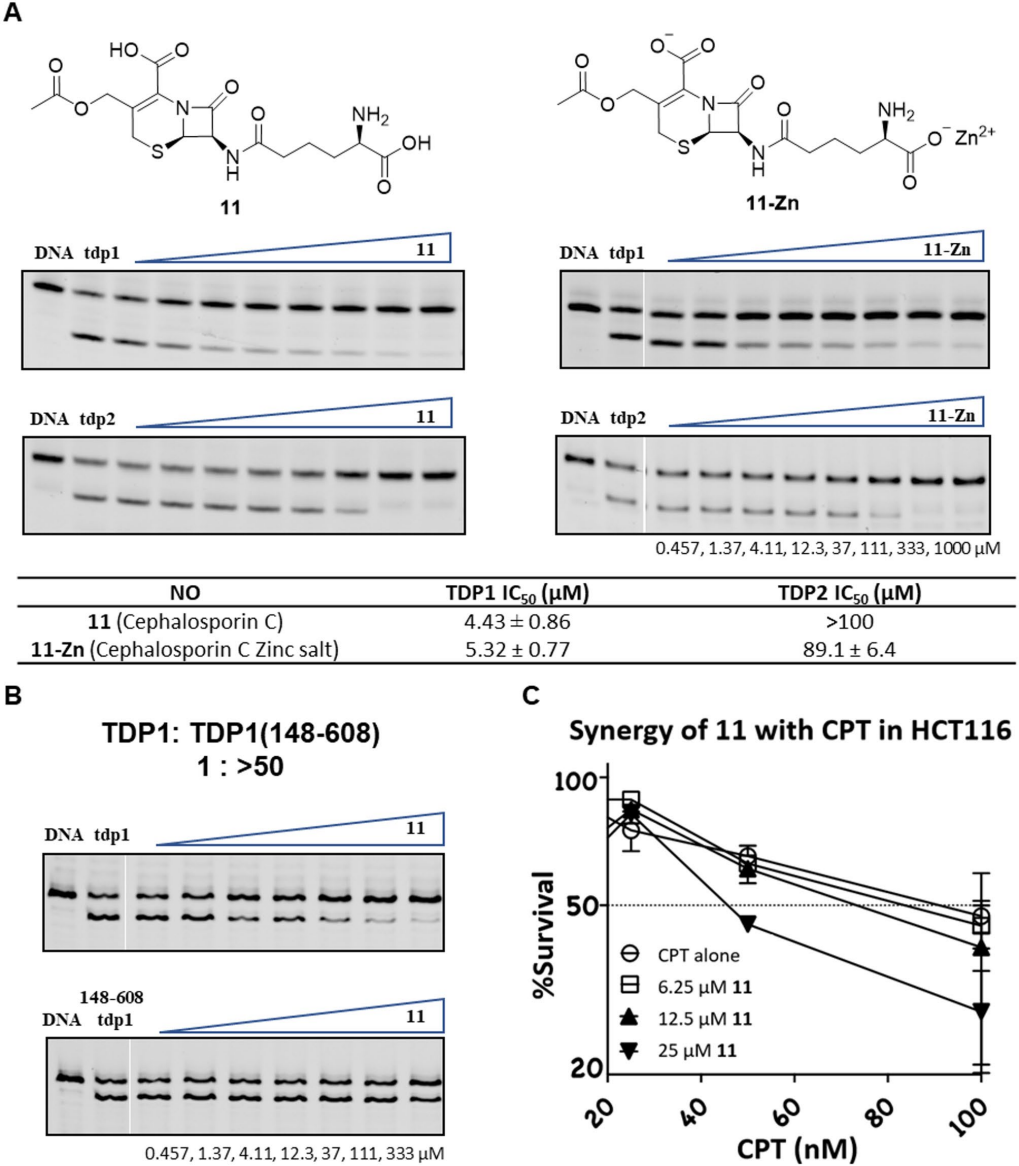
Biological activity of cephalosporin C (**11**). (**A**) Structures and gels from *in vitro* catalytic assays comparing cephalosporin C (**11**) and the zinc salt (**11-Zn**) against TDP1 and TDP2. In each gel: lane 1, N14Y only; lane 2, N14Y and TDP1 or TDP2 (DMSO control); lanes 3–10, 3-fold serial dilution of compounds from 0.457 µM to 1000 µM; Original gels are presented in Supplementary Figure S1. (**B**) Inhibition of cephalosporin C against full-length TDP1 and C-terminal TDP1(148–608). In each gel: lane 1, N14Y only; lane 2, N14Y and TDP1 or TDP1(148–608); lanes 3–9, 3-fold serial dilution of compounds from 0.457 µM to 333 µM; Original gels are presented in Supplementary Figure S1. (**C**) Synergistic effect of cephalosporin C with camptothecin (CPT) in human colon cancer cell line HCT116 as shown by cell viability.

Previously, we reported the X-ray crystal structure of **7b** (XZ634p) bound within the catalytic pocket TDP1 (149–608) (PDB code: 6W7K), shows that **7b** represents an ideal anchor that sits within the catalytic pocket, while projecting into both the DNA and TOP1 peptide substrate-binding regions^22^. More recently, we used this structure as a key design component in our oxime-based post-modification strategy and proteolysis-targeting chimeras (PROTACs)^23,34^. In the current study, we have applied this structure to guide our virtual screening effforts. To examine the potential binding modes of cephalosporin C with the TDP1 catalytic site, we conducted molecular docking studies using ICM Pro modeling software in conjunction with our previously reported high-resolution crystal structures of the TDP1 catalytic domain in complex with compound **7b** (PDB code: 6W7K)^22,23^. Crystal structures of imidazopyridine-based inhibitors containing dicarboxylate functionalities (**5a-e** and **7a-c**) bound to the TDP1 catalytic pocket show that the dicarboxylate groups engage in direct hydrogen bonds with the catalytic residues H263, K265, N283, H493, K495, and N516^21–24^. In our current work, we observed that cephalosporin C could potentially bind within the catalytic site of TDP1 by either noncovalent or covalent docking modes. When docked noncovalently, three hydrogen bonds are formed between two oxygens on the carboxylic acid of cephalosporin C and the N^π^ in H263 (2.74 Å), S399 (2.92 Å), and the side chain amine in K495 (3.15 Å) (Fig. 5A). The S459 residue forms a strong hydrogen bond with acetate carbonyl of cephalosporin C, characterized by a 1.54 Å hydrogen-to-oxygen distance. In this interaction, the side-chain hydroxyl of S459 acts as the hydrogen donor, while the acetate carbonyl oxygen of cephalosporin C serves as the acceptor, with a donor–hydrogen–acceptor angle of 149°. The terminal amino group of cephalosporin C forms hydrogen bond with the carbonyl oxygen in V401 (2.98 Å). The chain-terminal carboxylic oxygen of cephalosporin C forms an intramolecular hydrogen bond with the amide carbonyl oxygen (2.87 Å), which induces a bend in the methylene chain. The sidechain hydroxyl of S400 forms a strong hydrogen bond with the amide carbonyl of P461, characterized by a 1.82 Å hydrogen-to-oxygen distance and a donor–hydrogen–acceptor angle of 148°. Importantly, the sidechain hydroxyl of S400 is also in proximity to the lactam carbonyl carbon (3.88 Å) (Fig. 6A). An important property of β-lactam antibiotics is their ability to form covalent bonds with serine residues in penicillin-binding proteins (PBPs)^35^. This suggests that the hydroxyl group in S400 of TDP1 could potentially attack and open the lactam ring in cephalosporin C.

We repeated our simulations using the covalent docking protocols of our ICM software to examine whether the S400 hydroxyl could reasonably attack the cephalosporin C lactam ring^30^. In the covalent docking mode, the hydroxyl in S399 (2.9 Å), the N^π^ in H493 (2.72 Å) and the sidechain amide nitrogen in N516 (2.88 Å) interact with the inhibitor via hydrogen bonds with the two carboxylic acid oxygens of cephalosporin C (Fig. 5B). The Y204 residue interacts with the inhibitor via a hydrogen bond (3.54 Å) between the phenolic hydroxyl group and the acetate carbonyl oxygen of cephalosporin C. A terminal carboxylic acid oxygen of cephalosporin C forms a hydrogen bond with amide nitrogen in S403 (2.54 Å). The terminal amino group of cephalosporin C forms a hydrogen bond with amide nitrogen of A520 (2.8 Å). Both noncovalent and covalent docking modes of cephalosporin C suggest that there are possible multiple binding interactions between the molecule within the catalytic pocket of TDP1 and residues in the DNA substrate binding groove^31,36–38^. The more negative the binding score suggests a more favorable binding mode^29^. Noncovalent docking of cephalosporin C within the TDP1 DNA substrate binding groove resulted in an ICM docking score of −40.33, while covalent docking yielded a score of −22.81. This suggests that the noncovalent binding mode of cephalosporin C is more favorable.

### Confirmation of TDP1 inhibitory potencies

Cephalosporin C was originally isolated from a fungus of the genus *Acremonium* and first characterized in 1961^39^. It is produced from *Acremonium chrysogenum* through fermentation^40^. Cephalosporin C (**11**) obtained from commercial sources (Sigma-Aldrich) showed single-digit micromolar TDP1 inhibitory potency (TDP1 IC_50_ = 4.4 µM) but extremely poor TDP2 inhibitory potency (TDP2 IC_50_ > 100 µM) (Fig. 6A). Similarly, the zinc salt of cephalosporin C obtained as part of the TargetMol library showed similar TDP1 inhibitory potency (**LB2** IC_50_ = 5.5 µM). To independently confirm this latter data, we evaluated another commercially available zinc salt sample of cephalosporin C (**11-Zn)** (from A2B Chem LLC). We found that it also displayed single-digit micromolar TDP1 inhibitory potency (TDP1 IC_50_ = 5.3 µM) with poor TDP2 inhibition (TDP2 IC_50_ = 89.1 µM) (Fig. 6A).

Full-length TDP1 includes a catalytically competent C-terminal domain (residues 148–608) and an N-terminal domain (residues 1- 147) that is not necessary for catalysis. The N-terminal domain regulates and stabilizes the DNA-adducted end within the catalytic pocket, facilitating access of the phosphodiester linkage to hydrolysis^37,38^. To examine whether the binding of cephalosporin C occurs within the catalytic domain or relies on the presence of the N-terminal domain, we compared gel-based fluorescence *in vitro* assays using truncated TDP1 (148–608) and full-length TDP1 having the allosteric N-domain (residues 1–147). We observed that cephalosporin C (**11**) shows more than 50-fold loss of inhibitory potencies in the assay using only the C-terminal residues as compared with the full-length TDP1 (Fig. 6B). We undertook further assays to determine whether the β-lactams could act in cells as TDP1 inhibitors. Cephalosporin C showed no cytotoxicity on its own. We performed experiments combining cephalosporin C with the classical TOP1 inhibitor camptothecin (CPT, Fig. 6C)^41^. We found in the human colon cancer HCT116 cell line that cephalosporin C acted synergistically with the TOP1 inhibitor CPT at micromolar concentrations (25 µM) with higher concentrations of cephalosporin C leading to lower cell survival at the same concentration of TOP1 inhibitor (Fig. 6C).

### Mass spectral analysis of sites of protein covalent modification and biophysical characterization of binding

**Fig. 7:**
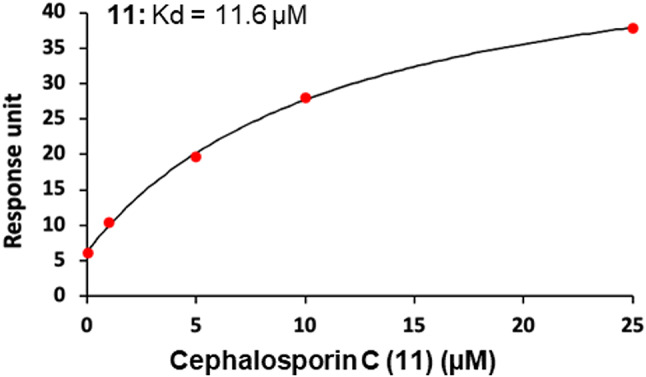
Surface Plasmon Resonance (SPR) binding curve of β-lactams cephalosporin C (**11**) using TDP1(148–608) on chips.

A central mechanism of action of β-lactam antibiotics involves covalent modification of serine residues in PBPs. However, β-lactams are also known to covalently modify proteins more broadly^35,42^. Mass spectroscopy has proven to be invaluable in characterizing protein modification by β-lactams^43,44^. We have recently reported the use of mass spectroscopy to identify sites of covalent modification by fluorosulfate-containing TDP1 inhibitors^45^. Since docking studies suggested that both covalent and non-covalent binding between the β-lactams and TDP1 active site could be possible, we employed Surface Plasmon Resonance (SPR) and mass spectrometry to investigate whether covalent modification was occurring. We evaluated cephalosporin C (**11**) in SPR studies. For SPR, we immobilized the TDP1(148–608) on an SPR chip and measured the binding of compounds as a function of concentration. The binding curves confirm binding of the compound to TDP1(148–608) (Fig. 7) with calculated Kd values of 11.6 µM for cephalosporin C (**11**). The fact that the compound could be eluted off the chip without impacting its binding capacity indicated that the compound was more likely bound non-covalently.

To further examine whether the compounds were capable of covalently binding to TDP1, we performed intact mass and post-translational modifications (PTM)-mapping analyses using mass spectrometry. Upon incubation of cephalosporin C (**11**) with TDP1(148–608) at different concentrations with enzyme-to-compound ratios ranging from 1:125 to 1:1000, we were not able to detect covalent modification of TDP1. We also digested the TDP1 following incubation with the compounds. We searched for possible covalent modifications with a peptide using a label-free quantitative proteomics approach, which we had used previously with our fluorosulfate-containing compounds^45^. Consistent with our intact mass analysis, we could not detect peptides from TDP1 that bore covalent modifications, even at high saturation levels of the compound to the enzyme. In conclusion, these biophysical approaches established that the β-lactams do not inhibit TDP1 by covalent modification but rather through non-covalent binding. This observation is consistent with the ICM docking scores (Fig. 5).

## Conclusions

After substantial effort, a series of structurally diverse TDP1 inhibitors has been discovered^46–54^ including natural product noncompetitive inhibitors of TDP1^55–57^ and peptide-based allosteric inhibitor^36^. Using previously reported crystal structures of our TDP1-bound small molecules to define ligand interactions, we employed the ICM Pro modeling software suite to perform a virtual screen of the publicly available DrugBank 5.0 library of 3449 structures for potential inhibitors. In this way we identified several hits with micromolar TDP1 inhibitory potencies in *in vitro* TDP1 catalytic assays. The β-lactam cephalosporin C was one of the most potent compounds, showing single-digit micromolar inhibitory potency. In follow-up, we performed a gel-based high-throughput TDP1 fluorescence assay to screen a commercially available library of 90 β-lactams. We found that several of the β-lactams showed good TDP1 inhibitory potencies. Taking cephalosporin C as a model compound, we performed docking studies, which showed that both noncovalent and covalent binding modes of β-lactam were possible, with the bound ligands engaging the catalytic pocket while binding to the DNA substrate-binding channel. Mass spectrometric and SPR analyses suggest that the TDP1 binding mode of β-lactams is non-covalent binding. Nonetheless, β-lactams may serve as a new and potentially useful platform to design TDP1-binding ligands that interact with the catalytic pocket and extend into the DNA substrate binding channel.

## Materials and methods

Folic acid (**9**, cat# F8758), dihydrofolic acid (**10**, cat# D7006) and cephalosporin C (**11**, cat# PH000602) were purchased from Millipore Sigma Inc. Cephalosporin C Zinc salt (**11-Zn**, cat# AI53703) was purchased from A2B Chem LLC. The β-lactam compound library (T002-00000006-UB100) was purchased from Target Molecule as DMSO solution (100 µL × 10 mM in DMSO). Molsoft ICM-Pro 3.9-3a was purchased from Molsoft LLC.

### Modeling protocol

Standard Molsoft parameters and procedures were used as defined in the unmodified ICM Chemist Pro version 3.9-3a using the DrugBank structures and a receptor from the crystal structure of TDP1-XZ634p complex (PDB code: 6W7K)^22,23^. The scoring function give an approximation of the binding free energy between a ligand and a receptor and is usually a function of different energy terms based on a force-field. Based on ICM manual, the ICM scoring function is weighted according to the parameters (i) internal force-field energy of the ligand, (ii) entropy loss of the ligand between bound and unbound states, (iii) ligand-receptor hydrogen bond interactions, (iv) polar and non-polar solvation energy differences between bound and unbound states, (v) electrostatic energy, (vi) hydrophobic energy, and (vii) hydrogen bond donor or acceptor desolvation. The lower the ICM score, the higher the chance the ligand is a binder^29^.

### TDP1 and TDP2 gel-based assay *in vitro*

The inhibition of TDP1 and TDP2 were conducted according to gel-based methods as previously described^21–24,45^. Briefly, 1 nM of the DNA substrate (N14Y, 5´Cy5-GATCTAAAAGACTT-pY-3´) was incubated with 40 pM full-length recombinant TDP1 or TDP1 (148–608) in the absence or presence of inhibitors for 15 min at room temperature in TDP1 reaction buffer (50 mM Tris-HCl, pH 7.5, 80 mM KCl, 2 mM EDTA, 1 mM DTT, 40 µg/mL BSA and 0.01% Tween 20). The inhibition of TDP2 was also conducted by using similar conditions. Briefly, 1 nM of DNA substrate (YN18, 5’-pY-TCCGTTGAAGCCTGCTTT-Cy5-3’) was incubated with 40 pM recombinant TDP2 in the absence or presence of inhibitors for 15 min at RT in TDP2 reaction buffer (50 mM Tris-HCl, pH 7.5, 80 mM KCl, 5 mM MgCl_2_, 0.1 mM EDTA, 1 mM DTT, 40 µg/mL BSA, and 0.01% Tween 20). The reactions of both TDP1 and TDP2 were stopped by adding an equal volume of gel loading buffer (99.5% (v/v) formamide, 5 mM EDTA). The samples were then subjected to a 20% denaturing PAGE gel followed by gel scanning using a Typhoon FLA 9500 scanner (GE Healthcare). The IC_50_ values of the TDP1 inhibitors were calculated by comparing the percentage of the cleavage product (N14P, 5´Cy5-GATCTAAAAGACTT-p-3´) produced to that in the DMSO control. The IC_50_ values of the TDP2 inhibitors were calculated by comparing the percentage of the cleavage product (PN18, 5’-p-TCCGTTGAAGCCTGCTTT-Cy5-3’) produced to that in the DMSO control.

### Synergistic effect of TDP1 inhibitors with camptothecin (CPT) in human colon cancer cell line HCT116

The synergistic effects of the TDP1 inhibitors with CPT were tested in human colon cancer cell line HCT116 based on cell viability^22,23^. Cells were first seeded in a 384-well black-clear plate until 30% confluency and then incubated with a serial dilution of CPT at the range of 0-100 nM (0, 12.5, 25, 50, 100 nM) in the present or the absence of desired concentrations of TDP1 inhibitors for 72 h at 37 ^o^C. DMSO was used as control. Viable cell numbers were counted from the brightfield images taken by Biotek Cytation 5.

### Surface plasmon resonance (SPR) analysis

SPR experiments were recorded for the truncated TDP1(148–608) and the β-lactam compound with Biacore T200 system (GE Healthcare). Using the amine coupling kit (Cytiva), 11,000 RU of TDP1 was covalently immobilized on a CM5 chip (Cytiva) via amine coupling. The β-lactam compounds were prepared in HBS-P buffer (Cytiva). Experiments in high-performance mode was carried out using five injections (30 µL/min) of increasing concentrations of the β-lactam (50 nM – 25 µM) passed over the sensor chip for 180 s, followed by a 1200 s dissociation. Buffer and reference signals were subtracted and binding affinities (K_d_) were determined using the Biacore T200 evaluation software (GE Healthcare).

## Electronic supplementary material

Below is the link to the electronic supplementary material.

Supplementary Material 1

Supplementary Material 2

Supplementary Material 3

Supplementary Material 4

Supplementary Material 5

Supplementary Material 6

## Data Availability

The authors declare that the data supporting the study are available within the article and Supplementary Information.
