# Supplementary material for: Potent β-lactam-based tyrosyl-DNA phosphodiesterase 1 inhibitors identified by a virtual screen
Source: Sci Rep. 2025 Jul 21;15:26510. doi: 10.1038/s41598-025-12503-8 (PMC12280126; doi:10.1038/s41598-025-12503-8)
Supplement: Supplementary file 1 — Supplementary Material 1 [file 41598_2025_12503_MOESM1_ESM.pdf]

**Electronic Supplementary Information (ESI)**

# Potent β-Lactam-based Tyrosyl-DNA Phosphodiesterase 1 Inhibitors Identified by a Virtual Screen

Xue Zhi Zhao[1,*], Wenjie Wang[2], Kiall F. Suazo[1,3], Md Rasel Al Mahmud[2], Keli Agama,[2] George T. Lountos[4], Thorkell Andresson[3], Yves Pommier[2], and Terrence R. Burke, Jr.[1]

[1]Chemical Biology Laboratory, Center for Cancer Research, National Cancer Institute, National Institutes of Health, Frederick, MD, USA.

[2]Developmental Therapeutics Branch & Laboratory of Molecular Pharmacology, Center for Cancer Research, National Cancer Institute, National Institutes of Health, Bethesda, MD, USA.

[3]Protein Characterization Laboratory, Cancer Research Technology Program, Frederick National Laboratory for Cancer Research, Frederick, MD, USA.

[4]Basic Science Program, Frederick National Laboratory for Cancer Research, Frederick, MD, USA.

Corresponding author[*]: xuezhi.zhao@nih.gov

**Table of Content**

| **Contents** | **Page** |
|---|---|

TABLES

Table S1. DrugBank answers.......................................................................................... S13

Table S2. Inhibitory potencies of 90 β-lactams against TDP1 in gel-based fluorescence assay in vitro.........................................................................................................................S3

Table S3. TDP1 selectivity of β-lactam leads compared to TDP2 in gel-based fluorescence assay in vitro............................................................................................................................ S6

Figure S1. Original images of gels and blots................................................................... S7

**Table S2.** Inhibitory potencies of 90 β-lactams against TDP1 in gel-based fluorescence assay *in vitro*.

| **No** | **Name** | **CAS** | **TDP1 $IC_{50}$ (μM)** |
|---|---|---|---|
| **LA1** | Cefotaxime sodium | 64485-93-4 | >1000 |
| **LA2** | piperacillin | 61477-96-1 | 28 ± 10.5 |
| **LA3** | Cefazolin | 25953-19-9 | >1000 |
| **LA4** | Cefathiamidine | 33075-00-2 | >1000 |
| **LA5** | Cefodizime Sodium | 86329-79-5 | >1000 |
| **LA6** | Sulbenicillin Sodium | 28002-18-8 | >1000 |
| **LA7** | Cephapirin Benzathine | 97468-37-6 | >1000 |
| **LA8** | Cefazedone | 56187-47-4 | >1000 |
| **LA9** | Cefotaxime | 63527-52-6 | >1000 |
| **LA10** | Pivmecillinam hydrochloride | 32887-03-9 | >1000 |
| **LA11** | Ceftaroline fosamil | 400827-46-5 | >1000 |
| **LA12** | Biapenem | 120410-24-4 | >1000 |
| **LB1** | Cefepime Dihydrochloride Monohydrate | 123171-59-5 | >1000 |
| **LB2** | Cephalosporin C zinc salt | 59143-60-1 | 5.54 ± 0.1 |
| **LB3** | Cefetamet pivoxil hydrochloride | 111696-23-2 | >1000 |
| **LB4** | Cefotetan | 69712-56-7 | 379 |
| **LB5** | Ticarcillin sodium | 29457-07-6 | >1000 |
| **LB6** | Sulbactam sodium | 69388-84-7 | 84.5 |
| **LB7** | Cefoselis Sulfate | 122841-12-7 | >1000 |
| **LB8** | Cefoperazone | 62893-19-0 | >1000 |
| **LB9** | Ampicillin sodium | 69-52-3 | 10.35 ± 0.35 |
| **LB10** | Cefdinir | 91832-40-5 | >1000 |
| **LB11** | Amoxicillin Sodium | 34642-77-8 | 15.2 ± 2.8 |
| **LB12** | Cefalexine EP IMpurity B | 22252-43-3 | >1000 |
| **LC1** | Cefsulodine sodium | 52152-93-9 | >1000 |
| **LC2** | Ceftiofur hydrochloride | 103980-44-5 | >1000 |
| **LC3** | Tebipenem Pivoxil | 161715-24-8 | >1000 |
| **LC4** | Latamoxef sodium | 64953-12-4 | 179.5 |
| **LC5** | Benzylpenicillin potassium | 113-98-4 | >1000 |
| **LC6** | Cephalotin acid | 153-61-7 | >1000 |
| **LC7** | Faropenem sodium | 122547-49-3 | >1000 |
| **LC8** | Cephapirin Sodium | 24356-60-3 | 495.8 |
| **LC9** | Cefotiam Hexetil Hydrochloride | 95789-30-3 | >1000 |
| **LC10** | Ceforanide | 60925-61-3 | 378 |

| | | | |
|---|---|---|---|
| **LC11** | Flucloxacillin sodium | 1847-24-1 | >1000 |
| **LD1** | Ampicillin Trihydrate | 7177-48-2 | 77.3 |
| **LD2** | Cefpodoxime proxetil | 87239-81-4 | >1000 |
| **LD3** | Cefpirome sulfate | 98753-19-6 | >1000 |
| **LD4** | Cefamandole nafate | 42540-40-9 | >1000 |
| **LD5** | Ertapenem sodium | 153773-82-1 | >1000 |
| **LD6** | Cefcapene Pivoxil Hydrochloride | 147816-24-8 | >1000 |
| **LD7** | 3-hydroxycephem | 54639-48-4 | >1000 |
| **LD8** | Procaine penicillin G | 54-35-3 | 84.8 |
| **LD9** | Ceftizoxime | 68401-81-0 | 10.8 ± 0.65 |
| **LD10** | Ceftibuten dihydrate | 118081-34-8 | >1000 |
| **LD11** | Cefquinome sulfate | 118443-89-3 | >1000 |
| **LE1** | Cefadroxil | 50370-12-2 | >1000 |
| **LE2** | Aristololactam I | 13395-02-3 | 80.6 |
| **LE3** | Cefminox Sodium | 92636-39-0 | >1000 |
| **LE4** | Sultamicillin Tosylate | 83105-70-8 | >1000 |
| **LE5** | Doripenem | 148016-81-3 | 148 |
| **LE6** | Mecillinam | 32887-01-7 | >1000 |
| **LE7** | Sulbactam | 68373-14-8 | 595 |
| **LE8** | Cefotiam hydrochloride | 66309-69-1 | >1000 |
| **LE9** | Ezetimibe | 163222-33-1 | 27.45 |
| **LE10** | Doripenem Hydrate | 364622-82-2 | >1000 |
| **LE11** | Cefaclor monohydrate | 70356-03-5 | >1000 |
| **LF1** | Meropenem | 96036-03-2 | >1000 |
| **LF2** | AAI101 | 1001404-83-6 | >1000 |
| **LF3** | Cefditoren pivoxil | 117467-28-4 | >1000 |
| **LF4** | Azlocillin sodium | 37091-65-9 | >1000 |
| **LF5** | nafcillin sodium | 7177-50-6 | >1000 |
| **LF6** | Ceftazidime | 72558-82-8 | 5.8 ± 0.1 |
| **LF7** | Sultamicillin | 76497-13-7 | >1000 |
| **LF8** | Tazobactam acid | 89786-04-9 | 73.8 |
| **LF9** | Cefonicid sodium | 61270-78-8 | >1000 |
| **LF10** | Cepazine | 64544-07-6 | >1000 |
| **LF11** | Cefuroxime sodium | 56238-63-2 | 110.8 |
| **LG1** | Sodium ceftiofur | 104010-37-9 | >1000 |
| **LG2** | Ceftriaxone sodium | 104376-79-6 | >1000 |
| **LG3** | Piperacillin sodium salt | 59703-84-3 | >1000 |
| **LG4** | Cefmenoxime hydrochloride | 75738-58-8 | >1000 |
| **LG5** | Carbenicillin disodium | 4800-94-6 | >1000 |

| | | | |
|---|---|---|---|
| **LG6** | Penicillin V potassium salt | 132-98-9 | >1000 |
| **LG7** | Cephalothin sodium | 58-71-9 | 39.4 |
| **LG8** | Cefprozil hydrate | 121123-17-9 | >1000 |
| **LG9** | Cefmetazole sodium | 56796-39-5 | >1000 |
| **LG10** | Ticarcillin disodium | 4697-14-7 | >1000 |
| **LG11** | Imipenem monohydrate | 74431-23-5 | >1000 |
| **LH1** | Cefpiramide acid | 70797-11-4 | >1000 |
| **LH2** | Cloxacillin sodium monohydrate | 7081-44-9 | >1000 |
| **LH3** | Aztreonam | 78110-38-0 | 620 ± 61 |
| **LH4** | Cefoxitin sodium | 33564-30-6 | >1000 |
| **LH5** | Cefixime | 79350-37-1 | 4 ± 0.1 |
| **LH6** | Amoxicillin | 26787-78-0 | >1000 |
| **LH7** | Dicloxacillin sodium monohydrate | 13412-64-1 | >1000 |
| **LH8** | Oxacillin sodium | 1173-88-2 | >1000 |
| **LH9** | Penicillin G sodium salt | 69-57-8 | >1000 |
| **LH10** | Cefazolin sodium salt | 27164-46-1 | 530 |
| **LH11** | Cephalexin | 15686-71-2 | >1000 |

**Table S3.** TDP1 selectivity of β-lactam leads compared to TDP2 in gel-based fluorescence assay *in vitro*.

| No | Name | CAS | TDP1 $IC_{50}$ (μM) | TDP2 $IC_{50}$ (μM) |
|---|---|---|---|---|
| **LA2** | piperacillin | 61477-96-1 | 28 ± 10.5 | 224 ± 26 |
| **LA9** | Cefotaxime | 63527-52-6 | > 1000 | 81.7 ± 5.2 |
| **LB2** | Cephalosporin C zinc salt | 59143-60-1 | 5.54 ± 0.1 | 38.3 ± 5.9 |
| **LB6** | Sulbactam sodium | 69388-84-7 | 84.5 | 201 |
| **LB9** | Ampicillin sodium | 69-52-3 | 10.35 ± 0.35 | 2.49 ± 0.29 |
| **LB11** | Amoxicillin Sodium | 34642-77-8 | 15.2 ± 2.8 | 99.05 ± 2.15 |
| **LC2** | Ceftiofur hydrochloride | 103980-44-5 | > 1000 | 80.9 ± 13.2 |
| **LC5** | Benzylpenicillin potassium | 113-98-4 | > 1000 | 26.15 ± 3.35 |
| **LC11** | Flucloxacillin sodium | 1847-24-1 | > 1000 | 36.05 ± 1.35 |
| **LD1** | Ampicillin Trihydrate | 7177-48-2 | 77.3 | > 1000 |
| **LD8** | Procaine penicillin G | 54-35-3 | 84.8 | 77.4 |
| **LD9** | Ceftizoxime | 68401-81-0 | 10.8 ± 0.65 | 37.6 ± 7.4 |
| **LE2** | Aristololactam I | 13395-02-3 | 80.6 | 70.1 |
| **LE9** | Ezetimibe | 163222-33-1 | 27.45 | 52.65 |
| **LF6** | Ceftazidime | 72558-82-8 | 5.8 ± 0.1 | 18.9 ± 2.2 |
| **LF8** | Tazobactam acid | 89786-04-9 | 73.8 | 45.5 |
| **LG7** | Cephalothin sodium | 58-71-9 | 39.4 | 35.2 |
| **LH5** | Cefixime | 79350-37-1 | 4 ± 0.1 | 69.3 ± 6 |

**Figure S1. Original images of gels and blots**

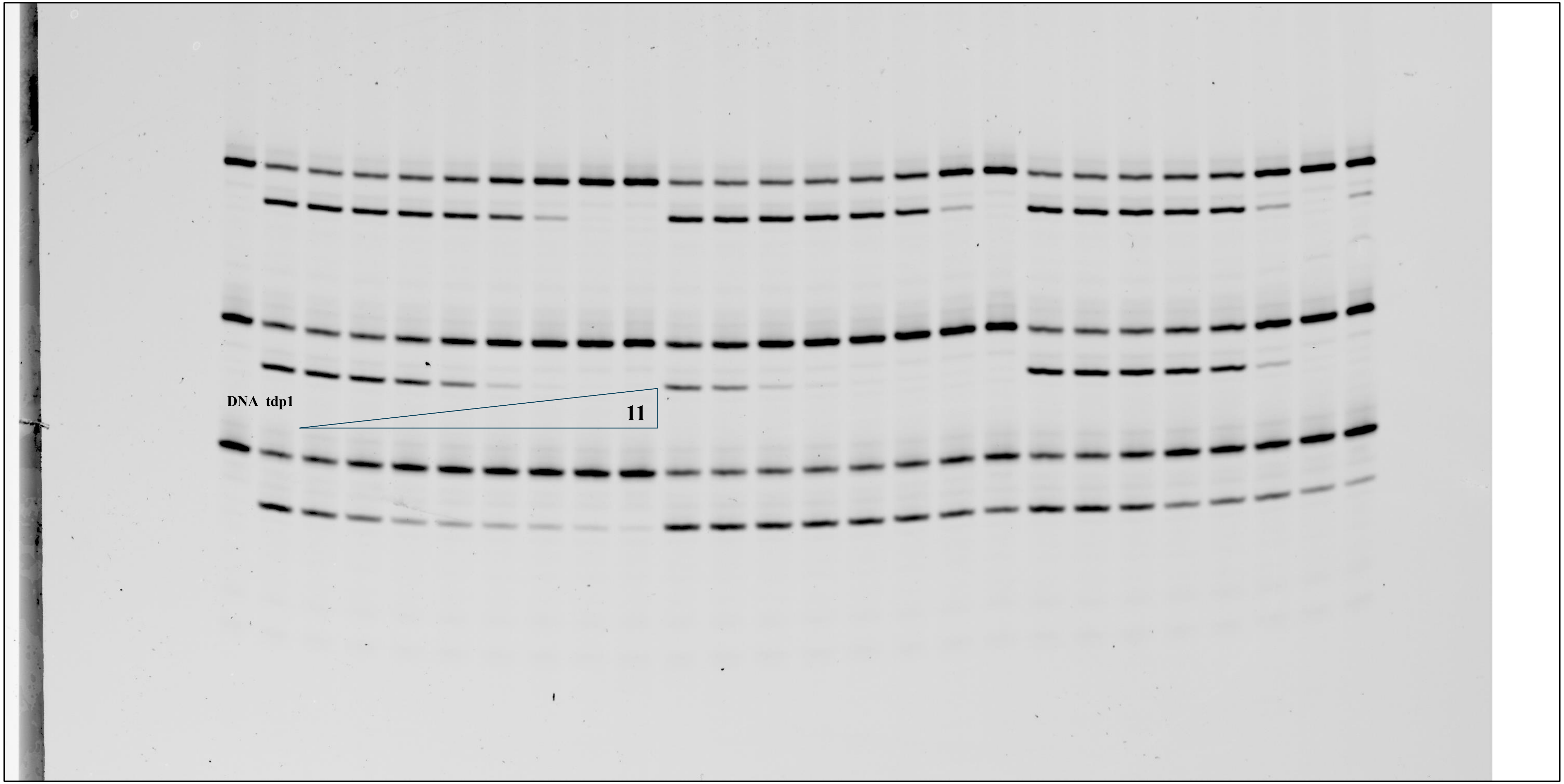


Original gel image of Figure 6A (left up)

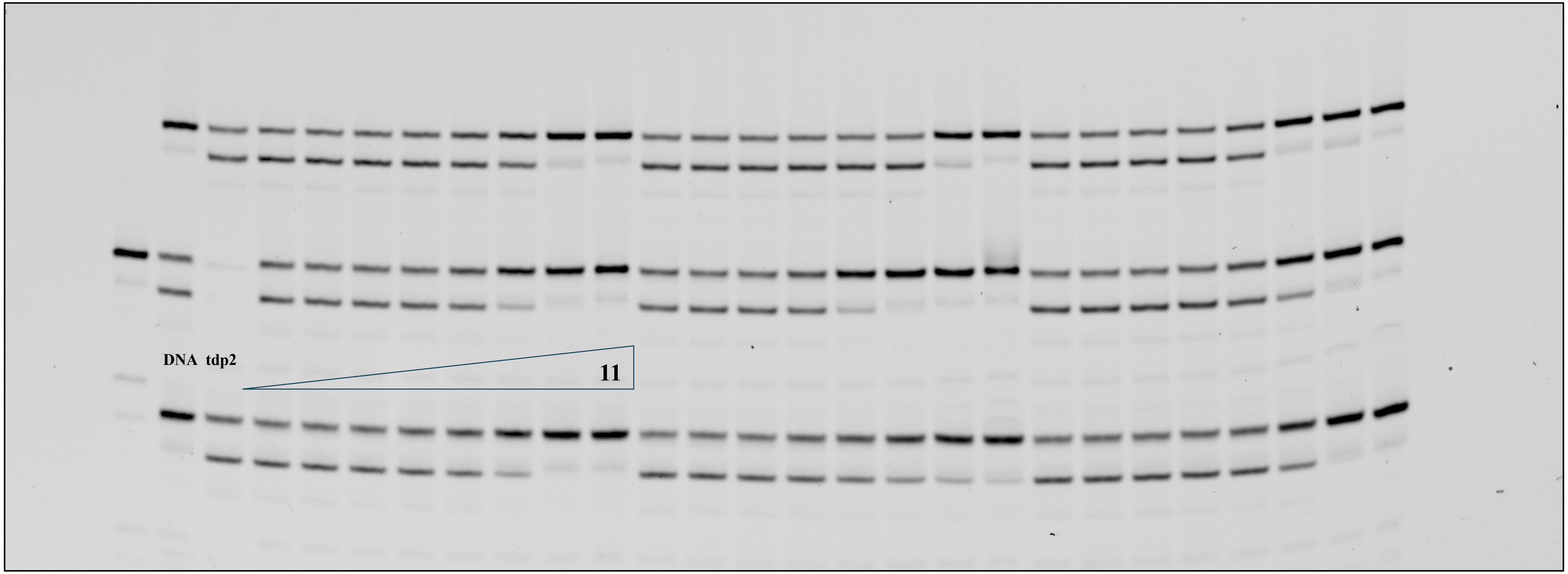


Original gel image of Figure 6A (left bottom)

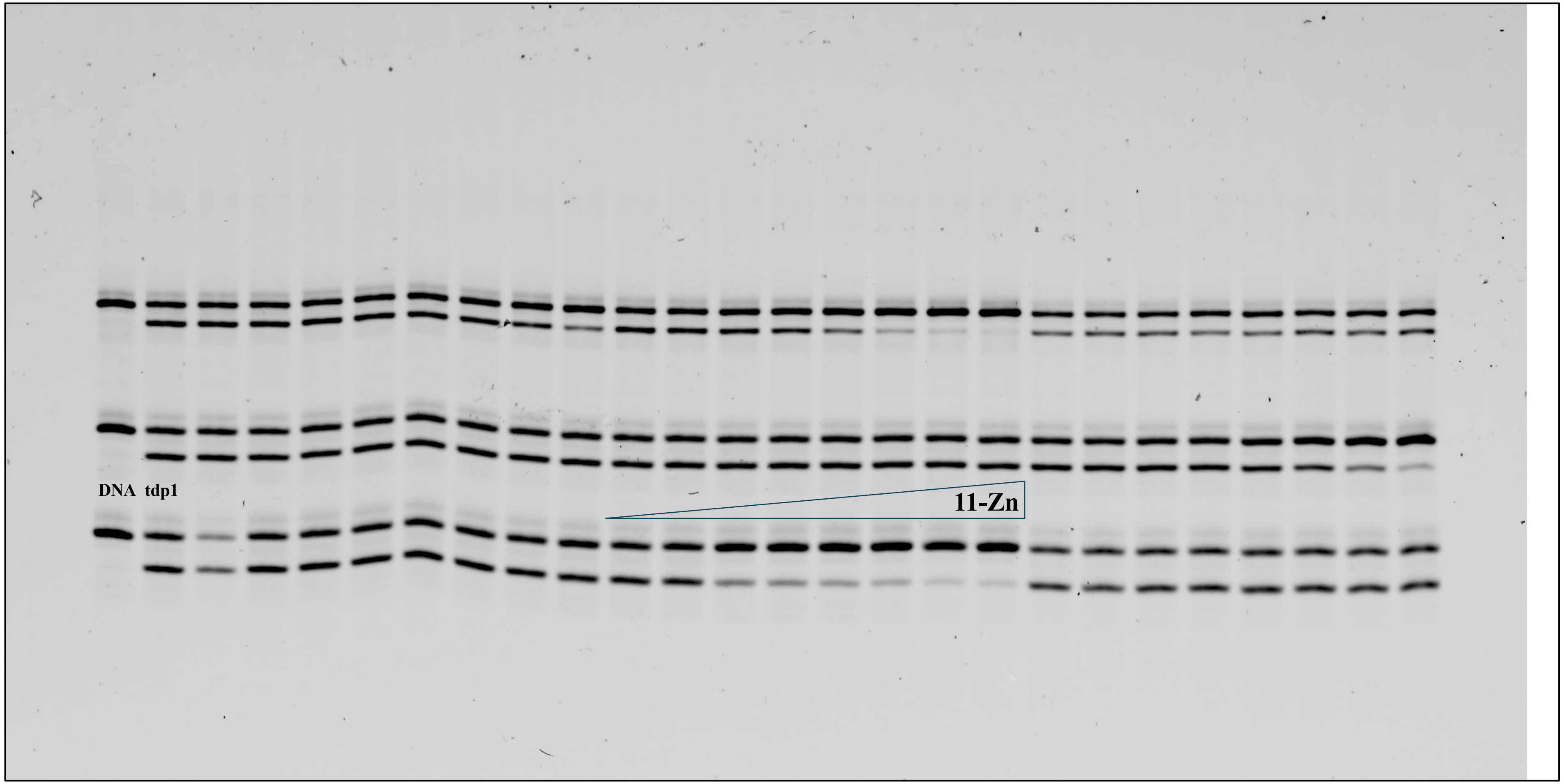


Original gel image of Figure 6A (right up)

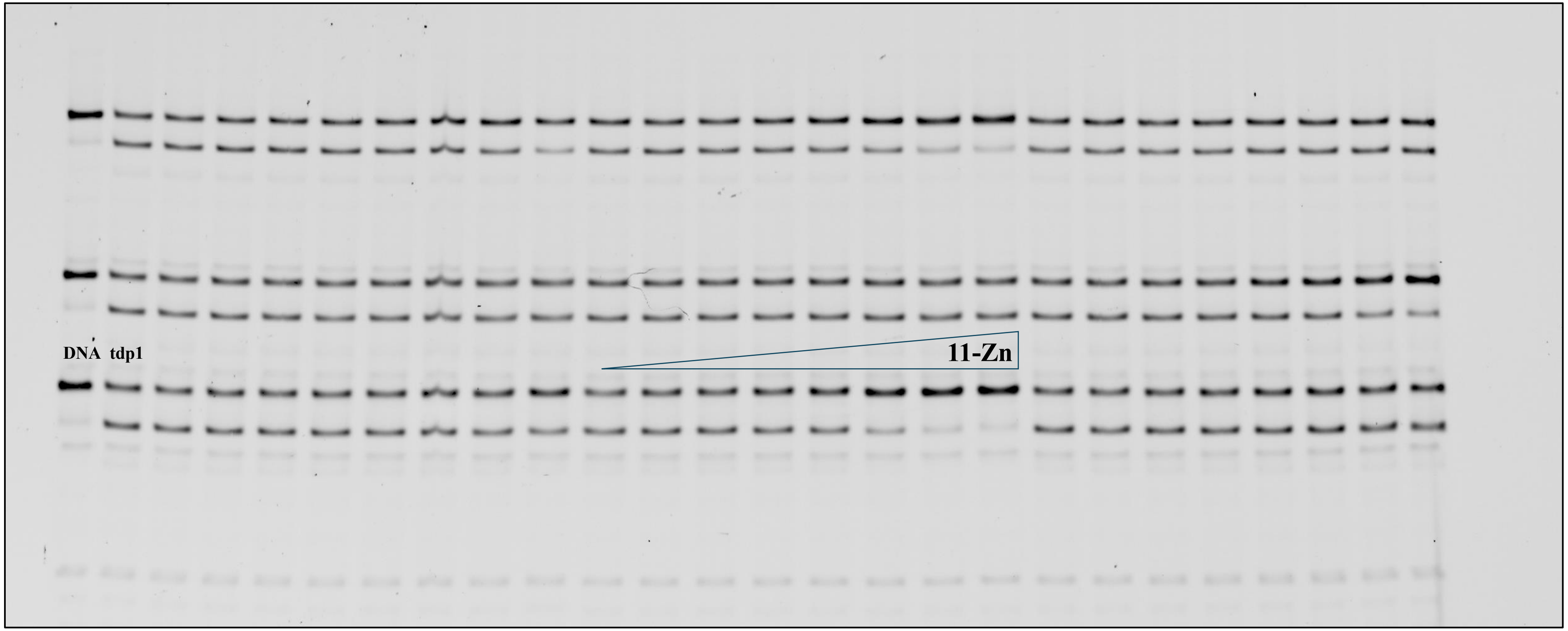


Original gel image of Figure 6A (right bottom)

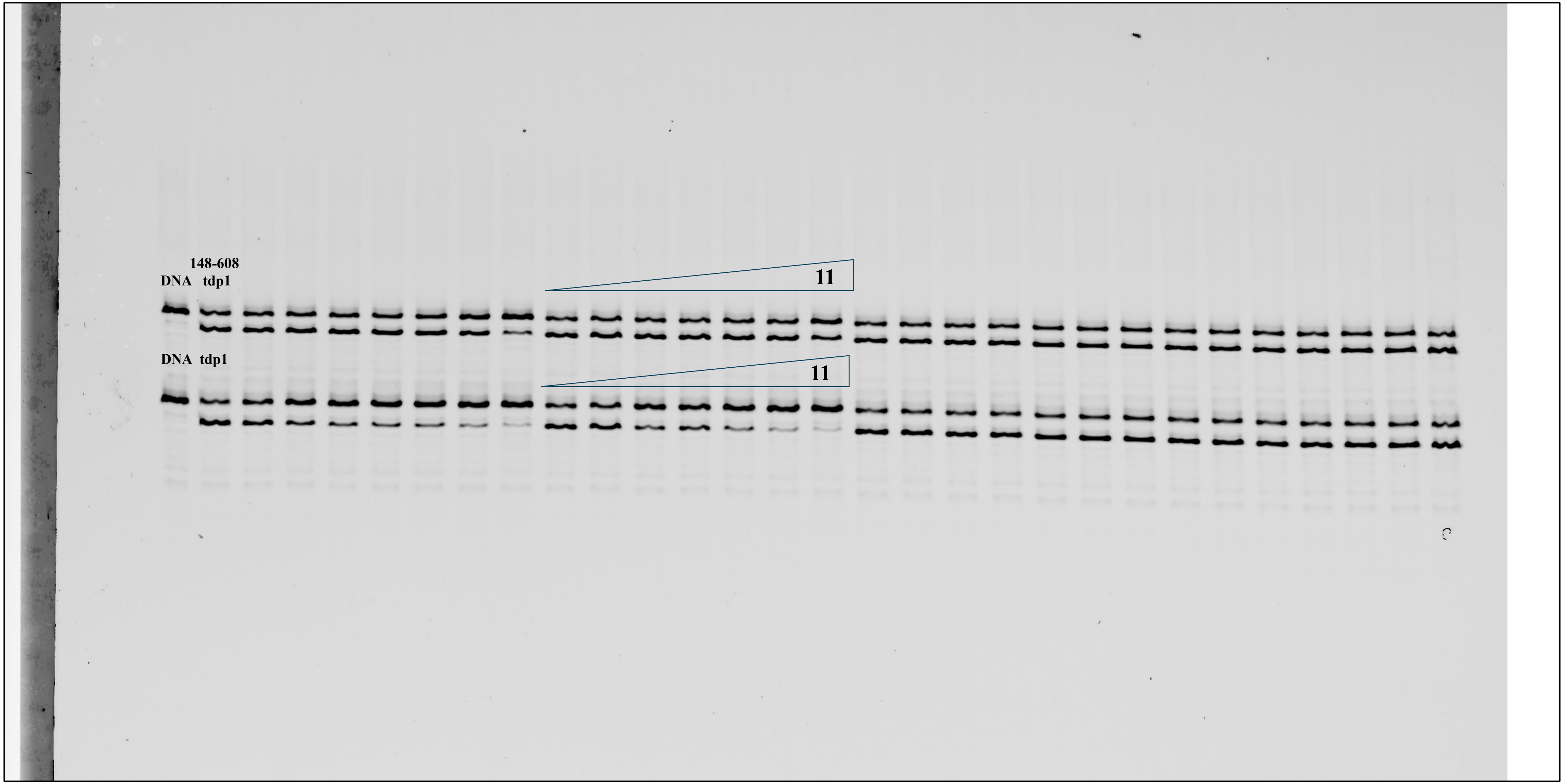


Original gel image of Figure 6B

# Table S1. DrugBank answers.

| mol | L | IX | NAME | Score | Natom | Nflex | Hbond | Hphob | VwInt | Eintl | Dsolv | SolEl | mfScore | dTSsc | RecConf |
|---|---|---|---|---|---|---|---|---|---|---|---|---|---|---|---|
| 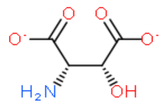 | 0 | 3289 | C4H7NO5 | -45.64 | 15 | 5 | -15.894 | -1.0143 | -10.85 | 0.6285 | 10.68 | -8.014 | -68.86688 | 0.41237 | 1 |
| 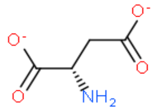 | 0 | 24 | 128 | -42.27 | 14 | 4 | -15.562 | -0.9705 | -10.02 | 1.3949 | 9.6572 | -4.107 | -61.19777 | 0.36276 | 1 |
| 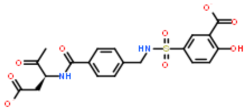 | 0 | 2808 | 161 | -42 | 50 | 9 | -15.003 | -4.27 | -23.51 | 0 | 19.88 | 3.0475 | -129.8508 | 1.52103 | 1 |
| 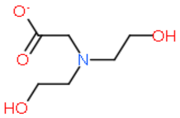 | 0 | 3352 | C6H13NO4 | -41.97 | 23 | 8 | -17.993 | -2.3301 | -10.74 | 0.4179 | 13.097 | 1.4803 | -68.40368 | 0.6514 | 1 |
| 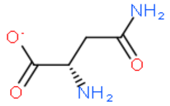 | 0 | 69 | 174 | -41.37 | 16 | 4 | -16.874 | -1.0091 | -9.885 | 2.4883 | 14.965 | -4.906 | -53.77205 | 0.41716 | 1 |
| 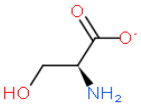 | 0 | 29 | C3H7NO3 | -41.18 | 13 | 4 | -15.248 | -1.0658 | -11.82 | 0 | 12.849 | -3.182 | -46.25409 | 0.35464 | 1 |
| 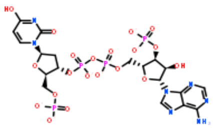 | 0 | 2868 | C19H27N7O20P4 | -40.71 | 71 | 16 | -19.301 | -4.9262 | -34.42 | 6.9505 | 37.093 | 6.4524 | -163.6429 | 2.32864 | 1 |
| 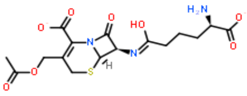 | # | 2990 | C16H21N3O8S | -40.33 | 47 | 11 | -15.278 | -4.1999 | -29.26 | 0.1544 | 23.973 | 8.6759 | -118.2111 | 1.66801 | 1 |
| 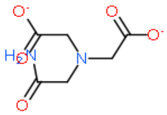 | 0 | 2515 | C6H10N2O5 | -40.23 | 21 | 6 | -18.922 | -1.4216 | -14.85 | 1.1843 | 17.622 | 8.961 | -64.66124 | 0.64355 | 1 |
| 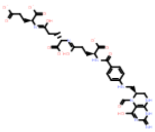 | 0 | 1825 | C30H37N9O13 | -39.83 | 85 | 21 | -19.052 | -4.8218 | -35.15 | 13.931 | 32.831 | 1.7728 | -148.4061 | 2.50139 | 1 |
| 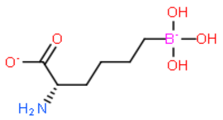 | 0 | 1747 | C6H15BNO5 | -38.97 | 27 | 10 | -12.264 | -2.2835 | -11.76 | 0 | 15.117 | -18.16 | -65.70261 | 0.79834 | 1 |

| | | | | | | | | | | | | | | | |
|---|---|---|---|---|---|---|---|---|---|---|---|---|---|---|---|
| 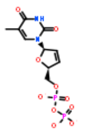 | 0 | 2296 | C10H14N2O10P2 | -38.79 | 35 | 6 | -17.566 | -2.9024 | -26.49 | 1.7717 | 24.596 | 16.631 | -102.1559 | 1.28046 | 1 |
| 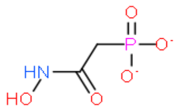 | 0 | 3294 | C2H6NO5P | -38.61 | 13 | 4 | -12.85 | -1.006 | -12.96 | 0 | 10.121 | -3.723 | -63.30363 | 0.36861 | 1 |
| 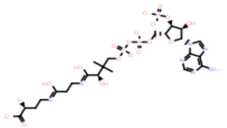 | 0 | 2864 | C23H37FN7O18P3 | -38.58 | 84 | 24 | -17.955 | -6.1446 | -36.09 | 2.9654 | 31.316 | 11.206 | -197.5432 | 2.65917 | 1 |
| 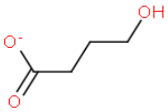 | 0 | 1246 | C4H8O3 | -38.45 | 14 | 4 | -13.753 | -1.6248 | -10.32 | 0 | 8.8285 | -1.924 | -52.44703 | 0.33854 | 1 |
| 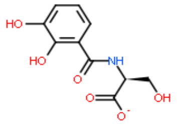 | 0 | 2425 | DBS | -38.24 | 27 | 4 | -13.991 | -2.4311 | -18.29 | 1.3951 | 13.782 | 5.2994 | -80.21549 | 0.97421 | 1 |
| 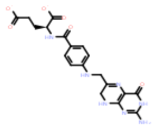 | 0 | 1776 | C19H21N7O6 | -38.07 | 51 | 7 | -14.6 | -4.901 | -29.57 | 4.1311 | 29.952 | 3.9101 | -143.3665 | 1.95317 | 1 |
| 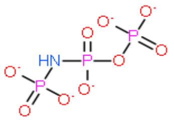 | 0 | 2764 | H6NO9P3 | -38 | 14 | 4 | -11.817 | -0.7126 | -10.25 | 0 | 16.384 | -18.27 | -39.48725 | 0.74592 | 1 |
| 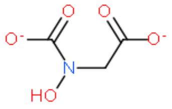 | 0 | 2650 | C3H5NO5 | -37.73 | 12 | 4 | -14.145 | -0.8605 | -10.39 | 0 | 11.324 | -2.846 | -66.84328 | 0.35321 | 1 |
| 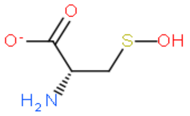 | 0 | 1682 | C3H7NO3S | -37.63 | 14 | 5 | -15.055 | -0.9673 | -10.86 | 0 | 14.514 | -2.908 | -41.4448 | 0.40317 | 1 |
| 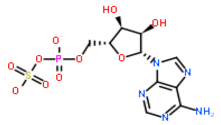 | 0 | 3351 | C10H14N5O10PS | -37.43 | 39 | 8 | -18.403 | -3.3475 | -27.62 | 0 | 26.826 | 21.317 | -97.2177 | 1.3236 | 1 |
| 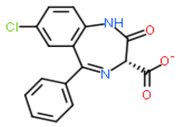 | 0 | 515 | 628 | -37.41 | 32 | 2 | -13.403 | -3.8204 | -19.5 | 3.3185 | 16.446 | 4.608 | -116.7291 | 1.13464 | 1 |

| | | | | | | | | | | | | | | | |
|---|---|---|---|---|---|---|---|---|---|---|---|---|---|---|---|
| 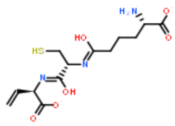 | 0 | 3095 | C13H21N3O6S | -36.98 | 42 | 15 | -18.07 | -3.2597 | -22.37 | 3.6588 | 24.958 | 5.1199 | -57.4296 | 1.24776 | 1 |
| 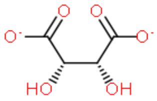 | 0 | 1481 | C4H6O6 | -36.76 | 14 | 5 | -14.817 | -0.9425 | -9.302 | 0.1748 | 12.754 | -3.05 | -69.7085 | 0.39934 | 1 |
| 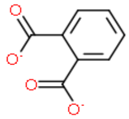 | 0 | 2458 | C8H6O4 | -36.68 | 16 | 2 | -13.275 | -1.8996 | -11.77 | 0.8342 | 12.539 | -1.749 | -81.88333 | 0.63229 | 1 |
| 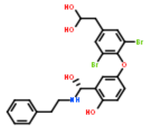 | 0 | 1864 | C23H23Br2NO5 | -36.51 | 54 | 12 | -18.073 | -5.5502 | -30.46 | 8.5213 | 26.292 | 17.218 | -117.9742 | 2.01286 | 1 |
| 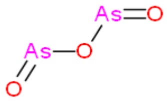 | 0 | 1039 | As2O3 | -35.85 | 5 | 2 | -13.398 | -0.4246 | -9.423 | 0 | 9.2888 | -0.621 | -29.0712 | 0.29846 | 1 |
| 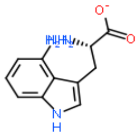 | 0 | 2029 | C11H13N3O2 | -35.78 | 28 | 4 | -12.41 | -2.8538 | -18.04 | 0 | 15.703 | 2.1034 | -65.86128 | 0.77456 | 1 |
| 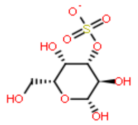 | 0 | 1595 | C6H12O9S | -35.74 | 27 | 7 | -15.911 | -2.1831 | -14.98 | 2.2611 | 18.866 | 1.8838 | -96.49989 | 0.92563 | 1 |
| 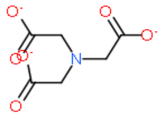 | 0 | 2731 | C6H9NO6 | -35.69 | 19 | 6 | -15.273 | -1.54 | -14.21 | 0 | 15.282 | 4.75 | -74.87811 | 0.70952 | 1 |
| 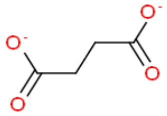 | 0 | 35 | 139 | -35.51 | 12 | 3 | -13.656 | -1.1906 | -7.613 | 0.9265 | 11.649 | -5.516 | -64.4677 | 0.35778 | 1 |
| 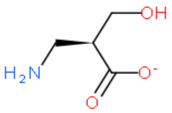 | 0 | 2604 | C4H9NO3 | -35.49 | 16 | 5 | -13.657 | -1.3722 | -10.63 | 0 | 10.242 | -0.117 | -46.85567 | 0.4118 | 1 |
| 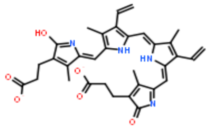 | 0 | 2566 | C33H34N4O6 | -35.34 | 75 | 7 | -13.812 | -6.8741 | -33.12 | 6.0897 | 23.144 | 18.352 | -103.1876 | 2.40588 | 1 |

| | | | | | | | | | | | | | | | |
|---|---|---|---|---|---|---|---|---|---|---|---|---|---|---|---|
| 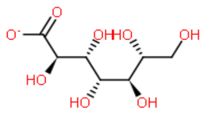 | 0 | 220 | C14H26CaO16 | -35.27 | 28 | 12 | -14.799 | -1.9242 | -14.66 | 1.3586 | 11.951 | 1.7281 | -76.0786 | 0.91001 | 1 |
| 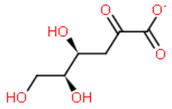 | 0 | 3153 | C6H10O6 | -35.19 | 21 | 8 | -15.444 | -1.6858 | -13.68 | 1.0217 | 14.931 | 3.1502 | -70.90764 | 0.70796 | 1 |
| 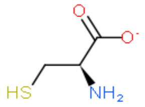 | 0 | 47 | C3H7NO2S | -35.13 | 13 | 4 | -11.931 | -0.9795 | -12.16 | 0 | 7.9038 | -0.689 | -38.82056 | 0.36593 | 1 |
| 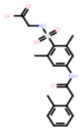 | 0 | 1477 | I84 | -35.03 | 48 | 6 | -10.54 | -5.3244 | -25.42 | 2.2086 | 14.305 | 7.5252 | -94.94736 | 1.15905 | 1 |
| 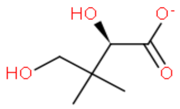 | 0 | 1695 | C6H11O4 | -34.96 | 21 | 5 | -13.257 | -2.1997 | -12.05 | 0 | 11.056 | 1.234 | -76.92692 | 0.58611 | 1 |
| 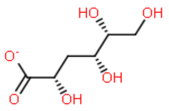 | 0 | 2512 | C6H12O6 | -34.71 | 23 | 9 | -16.343 | -1.4817 | -10.36 | 0 | 13.875 | 3.3023 | -70.12104 | 0.69811 | 1 |
| 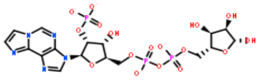 | 0 | 3373 | C17H24N5O17P3 | -34.6 | 62 | 15 | -13.977 | -4.2105 | -28.11 | 0 | 22.904 | 8.3546 | -130.8868 | 2.28167 | 1 |
| 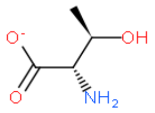 | 0 | 52 | C4H9NO3 | -34.53 | 16 | 4 | -14.228 | -1.4306 | -10.46 | 0 | 13.418 | 0.6463 | -55.74365 | 0.41784 | 1 |
| 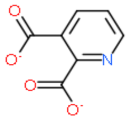 | 0 | 1574 | C7H5NO4 | -34.52 | 15 | 2 | -13.276 | -1.8385 | -11.61 | 0.7645 | 13.79 | -0.139 | -78.07652 | 0.62018 | 1 |
| 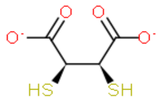 | 0 | 453 | C4H6O4S2 | -34.5 | 14 | 5 | -15.16 | -0.9752 | -10.78 | 4.7848 | 11.601 | 1.0906 | -65.23414 | 0.49239 | 1 |
| 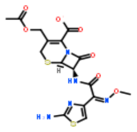 | 0 | 383 | m | -34.49 | 46 | 5 | -13.589 | -5.8738 | -27.6 | 4.576 | 24.23 | 11.382 | -144.3966 | 1.65274 | 1 |

| | | | | | | | | | | | | | | | |
|---|---|---|---|---|---|---|---|---|---|---|---|---|---|---|---|
| 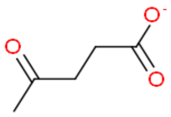 | 0 | 1986 | C5H8O3 | -34.49 | 15 | 3 | -12.789 | -2.1075 | -10.85 | 0.9672 | 10.466 | -0.146 | -60.70596 | 0.40105 | 1 |
| 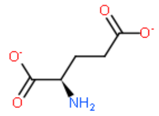 | 0 | 2247 | C5H9NO4 | -34.47 | 17 | 5 | -14.477 | -1.2926 | -11.67 | 4.9014 | 12.71 | -1.186 | -63.03674 | 0.39214 | 1 |
| 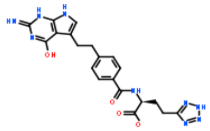 | 0 | 2729 | C20H21N9O4 | -34.04 | 53 | 9 | -20.252 | -4.2551 | -30.48 | 13.449 | 28.442 | 23.83 | -126.9526 | 1.66326 | 1 |
| 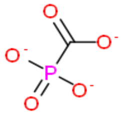 | 0 | 416 | CH3O5P | -33.91 | 7 | 1 | -13.997 | -0.4426 | -6.817 | 0 | 12.407 | -1.959 | -49.29499 | 0.31341 | 1 |
| 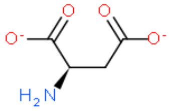 | 0 | 2375 | C4H7NO4 | -33.82 | 14 | 4 | -13.727 | -1.1444 | -8.487 | 0.0114 | 14.364 | -4.57 | -66.09765 | 0.39739 | 1 |
| 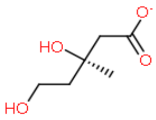 | 0 | 3181 | C6H12O4 | -33.82 | 21 | 6 | -14.734 | -2.1052 | -11.28 | 1.9935 | 13.548 | 1.8029 | -69.44064 | 0.64774 | 1 |
| 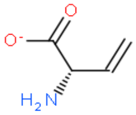 | 0 | 2895 | C4H7NO2 | -33.75 | 13 | 3 | -11.012 | -1.6781 | -12.72 | 0 | 9.2799 | -1.213 | -39.06551 | 0.36484 | 1 |
| 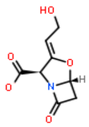 | 0 | 649 | 766 | -33.74 | 22 | 3 | -13.329 | -2.5872 | -15.25 | 1.7795 | 15.843 | 3.3678 | -67.86893 | 0.82731 | 1 |
| 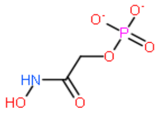 | 0 | 2720 | C2H6NO6P | -33.71 | 14 | 5 | -13.541 | -0.9344 | -13.89 | 0 | 14.405 | 1.9071 | -41.2484 | 0.43063 | 1 |
| 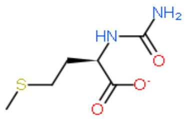 | 0 | 3038 | C6H12N2O3S | -33.5 | 23 | 6 | -13.124 | -2.4417 | -19.35 | 6.0161 | 16.371 | 1.8191 | -66.99065 | 0.67211 | 1 |
| 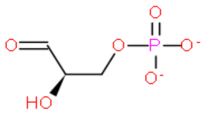 | 0 | 2007 | C3H7O6P | -33.42 | 15 | 5 | -14.93 | -0.9835 | -13.04 | 2.6028 | 12.437 | 6.3091 | -70.40249 | 0.43048 | 1 |

| | | | | | | | | | | | | | | |
|---|---|---|---|---|---|---|---|---|---|---|---|---|---|---|
| 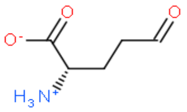 0 | 3413 | C5H10NO3 | -33.27 | 18 | 4 | -14.667 | -1.2197 | -12.55 | 2.5107 | 14.294 | 3.9079 | -46.64435 | 0.40228 | 1 |
| 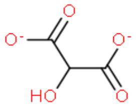 0 | 3326 | C3H2O5 | -33.27 | 10 | 3 | -12.461 | -0.6444 | -8.705 | 0 | 10.919 | -3.933 | -55.93174 | 0.31499 | 1 |
| 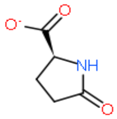 0 | 2776 | m | -33.16 | 15 | 1 | -12.352 | -1.5678 | -11.67 | 2.0494 | 11.979 | 0.0044 | -75.14117 | 0.38985 | 1 |
| 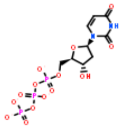 0 | 2074 | C9H15N2O14P3 | -33.11 | 39 | 9 | -12.564 | -2.7294 | -23.74 | 1.8432 | 27.413 | -4.274 | -77.05029 | 1.58283 | 1 |
| 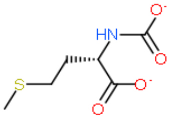 0 | 2599 | CXM | -33.08 | 21 | 6 | -13.727 | -2.3822 | -11.25 | 0.0837 | 13.837 | 0.7058 | -81.71757 | 0.62378 | 1 |
| 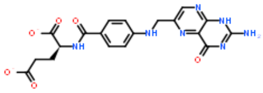 0 | 54 | 158 | -33.01 | 49 | 7 | -13.132 | -4.8127 | -23.41 | 2.9391 | 22.849 | 5.6746 | -126.59 | 1.60882 | 1 |
| 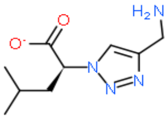 0 | 2925 | C9H16N4O2 | -32.97 | 30 | 6 | -16.04 | -3.3914 | -15.14 | 1.4845 | 19.485 | 8.9625 | -80.7114 | 1.03418 | 1 |
| 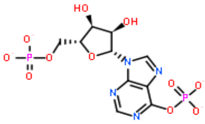 0 | 3173 | C10H14N4O11P2 | -32.96 | 37 | 7 | -18.635 | -3.3226 | -20.37 | 0.792 | 31.841 | 12.471 | -119.5245 | 1.31615 | 1 |
| 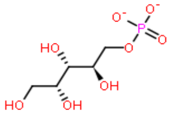 0 | 3385 | C5H13O8P | -32.95 | 25 | 10 | -15.476 | -1.8966 | -15.06 | 1.1987 | 15.736 | 6.0817 | -85.46236 | 0.90276 | 1 |
| 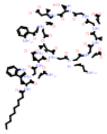 0 | 6 | C72H101N17O26 | -32.93 | 212 | 36 | -19.968 | -10.086 | -66.23 | 16.924 | 52.413 | 29.558 | -265.7175 | 4.82192 | 1 |
| 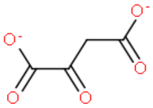 0 | 2358 | C4H3O5 | -32.81 | 11 | 3 | -13.467 | -0.8137 | -10.09 | 2.8984 | 12.966 | -2.067 | -64.82623 | 0.35896 | 1 |

| | | | | | | | | | | | | | | | |
|---|---|---|---|---|---|---|---|---|---|---|---|---|---|---|---|
| 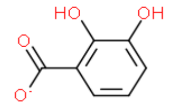 | 0 | 1461 | 2,3-Dihydroxybenzoic | -32.76 | 16 | 1 | -9.7405 | -1.992 | -12.84 | 0 | 8.315 | -1.183 | -52.70049 | 0.59911 | 1 |
| 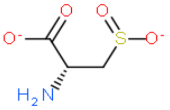 | 0 | 1908 | C3H7NO4S | -32.6 | 14 | 4 | -13.788 | -1.1589 | -12.13 | 1.2057 | 14.603 | 1.6852 | -60.19213 | 0.3976 | 1 |
| 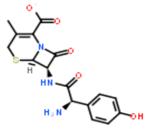 | 0 | 1011 | C16H17N3O5S | -32.54 | 41 | 5 | -13.757 | -3.8618 | -25.31 | 4.525 | 23.572 | 10.246 | -121.9049 | 1.45773 | 1 |
| 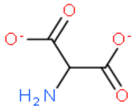 | 0 | 2032 | C3H5NO4 | -32.46 | 11 | 3 | -12.513 | -0.6338 | -9.43 | 1.2845 | 9.9862 | -1.519 | -52.43298 | 0.31922 | 1 |
| 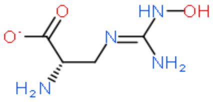 | 0 | 2229 | C4H10N4O3 | -32.41 | 20 | 6 | -11.823 | -1.3454 | -15.73 | 0 | 13.867 | 0.2026 | -51.37287 | 0.62686 | 1 |
| 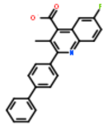 | 0 | 3144 | C23H16FNO2 | -32.4 | 42 | 2 | -9.0725 | -5.6508 | -23.79 | 2.8804 | 15.036 | 5.9361 | -146.4847 | 1.72411 | 1 |
| 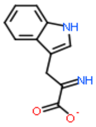 | 0 | 2684 | C11H10N2O2 | -32.38 | 24 | 3 | -11.685 | -2.8521 | -17.87 | 0 | 12.224 | 9.0808 | -69.91901 | 0.87437 | 1 |
| 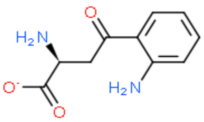 | 0 | 1828 | C10H12N2O3 | -32.37 | 26 | 4 | -17.414 | -2.6481 | -12.08 | 4.0159 | 19.504 | 9.2537 | -87.15441 | 0.90109 | 1 |
| 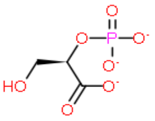 | 0 | 1495 | C3H7O7P | -32.35 | 15 | 5 | -11.061 | -0.9528 | -9.724 | 0 | 12.313 | -9.453 | -34.28228 | 0.58562 | 1 |
| 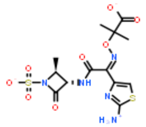 | 0 | 248 | C13H17N5O8S2 | -32.28 | 44 | 5 | -11.456 | -3.1114 | -22.24 | 0 | 20.805 | 3.6728 | -123.2235 | 1.35561 | 1 |
| 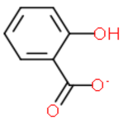 | 0 | 1208 | C14H10MgO6 | -32.2 | 15 | 1 | -9.5965 | -2.0995 | -12.38 | 0 | 9.3662 | -2.654 | -47.29767 | 0.52629 | 1 |

| | | | | | | | | | | | | | | | |
|---|---|---|---|---|---|---|---|---|---|---|---|---|---|---|---|
| 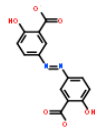 | 0 | 1119 | m | -32.11 | 30 | 2 | -10.465 | -3.0194 | -18.72 | 0 | 16.579 | 2.2474 | -93.69615 | 1.15516 | 1 |
| 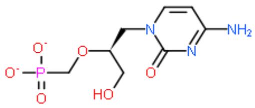 | 0 | 262 | 369 | -32.06 | 30 | 7 | -14.182 | -2.9421 | -15.84 | 0 | 15.847 | 8.399 | -90.36785 | 0.94329 | 1 |
| 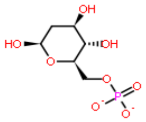 | 0 | 2329 | C6H13O8P | -32.03 | 26 | 6 | -15.499 | -2.0839 | -11.6 | 0 | 20.456 | 1.8714 | -81.11015 | 0.87293 | 1 |
| 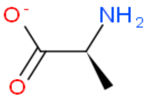 | 0 | 56 | C3H7NO2 | -31.96 | 12 | 2 | -11.156 | -1.4484 | -10.05 | 0 | 9.4997 | -1.58 | -35.51609 | 0.34612 | 1 |
| 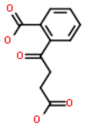 | 0 | 1995 | OSB | -31.96 | 24 | 4 | -13.322 | -2.9518 | -17.64 | 0 | 15.869 | 10.696 | -115.3455 | 1.08035 | 1 |
| 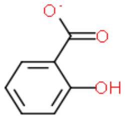 | 0 | 1211 | [NO | -31.95 | 15 | 1 | -9.5782 | -2.1576 | -12.39 | 0 | 9.3626 | -2.278 | -47.35593 | 0.52629 | 1 |
| 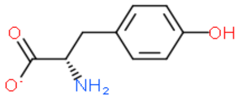 | 0 | 31 | 135 | -31.89 | 23 | 4 | -12.577 | -2.5459 | -15.54 | 0.7297 | 16.806 | 2.6253 | -79.24696 | 0.95724 | 1 |
| 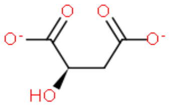 | 0 | 3162 | C4H6O5 | -31.81 | 13 | 4 | -14.143 | -1.0431 | -8.018 | 4.6405 | 12.878 | -3.266 | -64.43348 | 0.43827 | 1 |
| 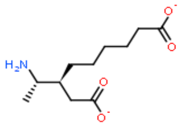 | 0 | 2638 | C11H21NO4 | -31.81 | 35 | 10 | -14.395 | -3.0072 | -12.35 | 3.6752 | 15.483 | -1.264 | -88.8108 | 1.15979 | 1 |
| 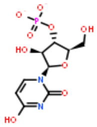 | 0 | 2510 | C9H13N2O9P | -31.77 | 32 | 7 | -18.369 | -2.4566 | -16.69 | 4.2834 | 21.82 | 15.002 | -98.39745 | 1.18834 | 1 |
| 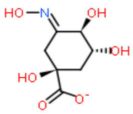 | 0 | 3380 | C7H11NO6 | -31.69 | 24 | 5 | -13.893 | -1.7739 | -9.894 | 0 | 14.382 | 1.1433 | -64.96088 | 0.66937 | 1 |

| | | | | | | | | | | | | | | | |
|---|---|---|---|---|---|---|---|---|---|---|---|---|---|---|---|
| 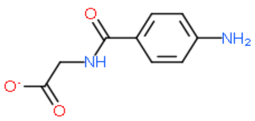 | 0 | 238 | 2148 | -31.6 | 23 | 2 | -11.468 | -2.8339 | -15.69 | 6.6958 | 13.141 | -0.113 | -60.3061 | 0.65605 | 1 |
| 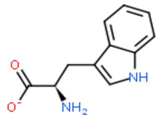 | 0 | 2906 | C11H12N2O2 | -31.57 | 26 | 4 | -13.127 | -2.8366 | -18.22 | 2.8866 | 14.035 | 10.756 | -67.95979 | 0.88775 | 1 |
| 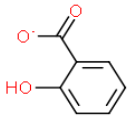 | 0 | 814 | Salicylic | -31.52 | 15 | 1 | -9.538 | -2.111 | -12.26 | 0 | 9.4819 | -2.165 | -46.90672 | 0.5327 | 1 |
| 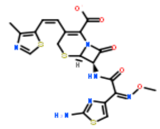 | 0 | 938 | C19H18N6O5S3 | -31.49 | 50 | 3 | -12.82 | -5.8024 | -28.9 | 7.1479 | 26.51 | 11.753 | -135.6152 | 2.0065 | 1 |
| 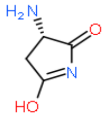 | 0 | 3151 | C4H6N2O2 | -31.41 | 14 | 2 | -14.88 | -1.0883 | -13.14 | 5.0559 | 16.353 | 5.4694 | -39.99859 | 0.3731 | 1 |
| 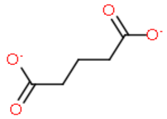 | 0 | 3209 | C5H8O4 | -31.36 | 15 | 4 | -12.795 | -1.5535 | -9.625 | 4.4682 | 11.739 | -3.341 | -68.11737 | 0.39014 | 1 |
| 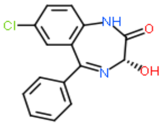 | 0 | 722 | 842 | -31.35 | 31 | 2 | -11.929 | -4.2258 | -19.22 | 0.9538 | 17.084 | 9.5323 | -115.4272 | 1.26431 | 1 |
| 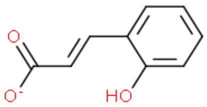 | 0 | 1442 | 2HC | -31.25 | 19 | 1 | -10.294 | -2.5977 | -15.82 | 0 | 14.269 | 1.5878 | -61.04762 | 0.81103 | 1 |
| 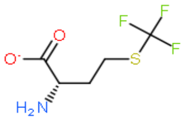 | 0 | 3436 | C5H8F3NO2S | -31.24 | 19 | 5 | -11.287 | -2.759 | -13.52 | 0.766 | 10.921 | 1.5782 | -76.33459 | 0.74707 | 1 |
| 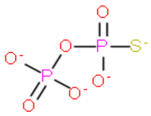 | 0 | 2159 | H3O6P2S | -31.17 | 9 | 2 | -14.33 | -0.6229 | -11.14 | 0 | 14.835 | 6.5428 | -44.2564 | 0.50663 | 1 |
| 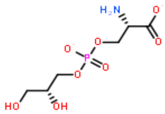 | 0 | 2227 | C6H14NO8P | -31.14 | 28 | 11 | -15.658 | -2.205 | -20.11 | 3.4676 | 21.279 | 8.2066 | -86.48567 | 1.2023 | 1 |

| | | | | | | | | | | | | | | | |
|---|---|---|---|---|---|---|---|---|---|---|---|---|---|---|---|
| 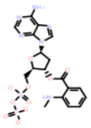 | 0 | 2810 | C18H22N6O10P2 | -31.1 | 55 | 7 | -13.518 | -5.4361 | -25.24 | 12.412 | 28.654 | -1.746 | -136.7774 | 2.00321 | 1 |
| 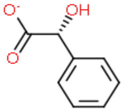 | 0 | 2024 | m | -31.07 | 18 | 3 | -9.7389 | -2.5152 | -12.18 | 0 | 8.8175 | -1.486 | -56.15239 | 0.55847 | 1 |
| 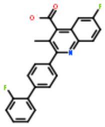 | 0 | 3184 | C23H15F2NO2 | -30.96 | 42 | 1 | -8.8346 | -5.8132 | -23.41 | 3.1857 | 15.26 | 7.1916 | -147.2195 | 1.75089 | 1 |
| 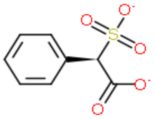 | 0 | 1641 | 256 | -30.95 | 20 | 3 | -12.979 | -2.3581 | -16.97 | 0 | 15.555 | 10.561 | -71.14018 | 0.68892 | 1 |
| 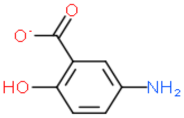 | 0 | 139 | 244 | -30.95 | 17 | 1 | -9.7766 | -2.1523 | -13.73 | 0 | 11.076 | 0.0805 | -50.18031 | 0.52866 | 1 |
| 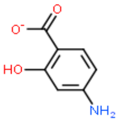 | 0 | 128 | 233 | -30.91 | 17 | 1 | -9.6054 | -2.1303 | -13.05 | 0 | 10.541 | -0.971 | -46.861 | 0.51866 | 1 |
| 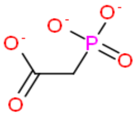 | 0 | 2527 | C2H5O5P | -30.85 | 10 | 2 | -12.814 | -0.9849 | -10.76 | 1.8439 | 12.427 | 2.058 | -53.43217 | 0.37138 | 1 |
| 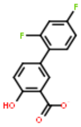 | 0 | 741 | 861 | -30.84 | 25 | 1 | -7.8999 | -3.8084 | -17.41 | 0 | 11.02 | 0.371 | -93.891 | 1.16013 | 1 |
| 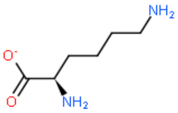 | 0 | 2933 | C6H14N2O2 | -30.8 | 23 | 7 | -15.69 | -2.0193 | -15.34 | 4.5731 | 20.043 | 5.462 | -53.49264 | 0.71032 | 1 |
| 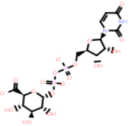 | 0 | 2732 | C15H22N2O18P2 | -30.79 | 56 | 14 | -19.113 | -3.0569 | -29.91 | 0.6454 | 34.58 | 22.437 | -153.7937 | 2.13922 | 1 |
| 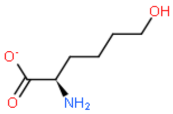 | 0 | 1980 | C6H13NO3 | -30.75 | 22 | 7 | -15.379 | -2.0455 | -15.32 | 3.72 | 15.417 | 10.381 | -56.24981 | 0.70977 | 1 |

| | | | | | | | | | | | | | | | |
|---|---|---|---|---|---|---|---|---|---|---|---|---|---|---|---|
| 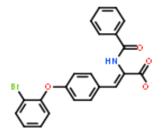 | 0 | 1506 | C22H16BrNO4 | -30.74 | 43 | 4 | -10.725 | -5.6626 | -28.12 | 5.0189 | 18.7 | 13.442 | -137.7198 | 1.79303 | 1 |
| 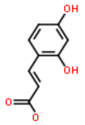 | 0 | 1490 | C9H8O4 | -30.73 | 20 | 1 | -10.375 | -2.4068 | -16.4 | 0 | 15.232 | 2.279 | -65.0305 | 0.88 | 1 |
| 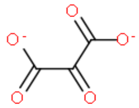 | 0 | 3241 | C3H2O5 | -30.68 | 8 | 2 | -12.356 | -0.4266 | -8.538 | 0 | 12.779 | -2.082 | -53.48209 | 0.27831 | 1 |
| 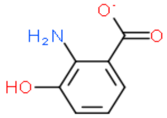 | 0 | 3293 | C7H7NO3 | -30.6 | 17 | 1 | -10.129 | -2.1658 | -13.25 | 0 | 7.9825 | 4.7445 | -50.9012 | 0.62528 | 1 |
| 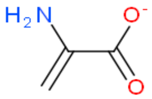 | 0 | 2405 | C3H5NO2 | -30.6 | 10 | 1 | -9.9938 | -1.488 | -11.03 | 0 | 7.6163 | 0.6076 | -32.22699 | 0.2989 | 1 |
| 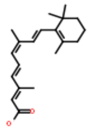 | 0 | 410 | 523 | -30.6 | 49 | 1 | -8.8815 | -5.2199 | -23.42 | 1.9692 | 14.8 | 8.9479 | -100.6146 | 1.52895 | 1 |
| 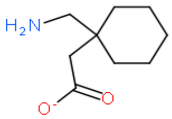 | 0 | 872 | 996 | -30.59 | 28 | 4 | -12.722 | -2.8837 | -11.38 | 1.4134 | 11.555 | 4.5407 | -72.03551 | 0.72499 | 1 |
| 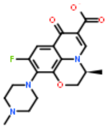 | 0 | 1008 | 1137 | -30.54 | 45 | 1 | -9.1323 | -4.0586 | -19.5 | 1.3879 | 15.685 | 2.3936 | -102.7616 | 1.34118 | 1 |
| 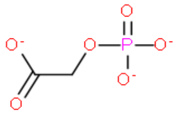 | 0 | 2440 | C2H5O6P | -30.53 | 11 | 3 | -12.653 | -0.8433 | -10.99 | 1.4763 | 13.982 | -0.196 | -53.56651 | 0.35463 | 1 |
| 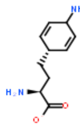 | 0 | 2439 | C10H16N2O2 | -30.53 | 29 | 6 | -13.716 | -2.6831 | -16.58 | 2.6957 | 17.131 | 6.7936 | -62.47058 | 0.83895 | 1 |
| 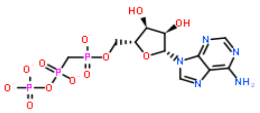 | 0 | 2321 | C11H18N5O12P3 | -30.51 | 45 | 10 | -13.7 | -4.0491 | -21.6 | 0 | 31.743 | -2.026 | -116.7005 | 1.8096 | 1 |

| | | | | | | | | | | | | | | | |
|---|---|---|---|---|---|---|---|---|---|---|---|---|---|---|---|
| 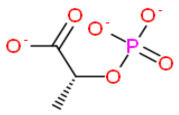 | 0 | 1517 | C3H7O6P | -30.5 | 14 | 3 | -12.31 | -1.3774 | -12.44 | 0 | 14.423 | 2.1005 | -58.19397 | 0.41696 | 1 |
| 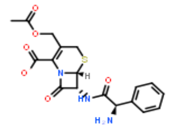 | 0 | 575 | 689 | -30.5 | 46 | 7 | -12.337 | -5.079 | -28.38 | 0.5087 | 24.811 | 14.212 | -130.0082 | 1.68633 | 1 |
| 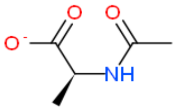 | 0 | 2248 | N-Acetyl-L-alanine.mol | -30.48 | 17 | 2 | -11.471 | -2.2206 | -12.28 | 1.2078 | 11.868 | 2.2455 | -53.53759 | 0.49193 | 1 |
| 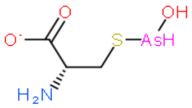 | 0 | 1585 | C3H8AsNO3S | -30.46 | 16 | 6 | -13.644 | -1.3574 | -12.76 | 0.4468 | 17.138 | 1.5211 | -40.1527 | 0.6452 | 1 |
| 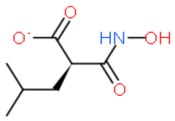 | 0 | 2068 | C7H13NO4 | -30.45 | 24 | 6 | -13.538 | -2.4355 | -15.08 | 0 | 17.276 | 6.0055 | -81.44569 | 0.92128 | 1 |
| 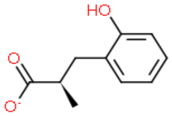 | 0 | 1451 | OAC | -30.36 | 24 | 3 | -12.397 | -3.1615 | -16.73 | 3.0472 | 13.676 | 9.0394 | -75.88277 | 0.80091 | 1 |
| 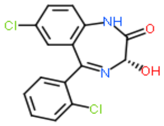 | 0 | 81 | 186 | -30.31 | 31 | 1 | -11.801 | -4.4286 | -17.6 | 1.6514 | 16.944 | 8.802 | -112.6676 | 1.25402 | 1 |
| 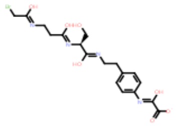 | 0 | 2490 | C18H23BrN4O7 | -30.29 | 52 | 16 | -12.502 | -3.8697 | -29.04 | 6.8122 | 20.081 | 6.3875 | -103.5978 | 1.90173 | 1 |
| 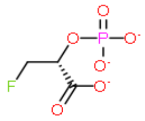 | 0 | 2800 | C3H6FO6P | -30.29 | 14 | 4 | -14.593 | -1.3237 | -9.354 | 0.4101 | 14.541 | 5.0286 | -74.78617 | 0.50564 | 1 |
| 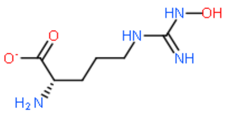 | 0 | 2827 | C6H14N4O3 | -30.28 | 26 | 9 | -15.386 | -1.8468 | -15.46 | 0 | 17.889 | 9.8881 | -67.8534 | 0.99774 | 1 |
| 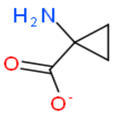 | 0 | 1843 | C4H7NO2 | -30.19 | 13 | 2 | -11.247 | -1.637 | -10.58 | 1.5212 | 9.9725 | 0.5362 | -45.74892 | 0.37908 | 1 |

| | | | | | | | | | | | | | | | |
|---|---|---|---|---|---|---|---|---|---|---|---|---|---|---|---|
| 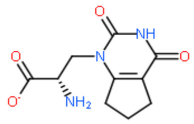 | 0 | 2921 | C10H13N3O4 | -30.16 | 29 | 4 | -12.533 | -2.5545 | -16.75 | 0.0671 | 17.551 | 6.6767 | -81.59696 | 0.97263 | 1 |
| 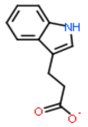 | 0 | 2469 | C11H11NO2 | -30.15 | 24 | 3 | -10.808 | -3.1337 | -16.88 | 0.9956 | 12.351 | 6.8411 | -64.30096 | 0.8663 | 1 |
| 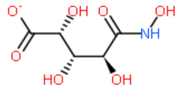 | 0 | 3375 | 5289593 | -30.12 | 21 | 9 | -14.911 | -1.3296 | -12.88 | 0 | 15.894 | 6.1416 | -79.32113 | 0.77419 | 1 |
| 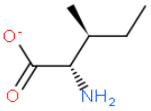 | 0 | 62 | 167 | -30.11 | 21 | 4 | -10.997 | -2.4873 | -12.61 | 0 | 13.325 | -0.613 | -62.52589 | 0.62082 | 1 |
| 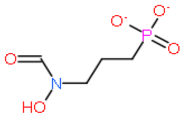 | 0 | 2644 | C4H10NO5P | -30.08 | 19 | 6 | -13.085 | -1.5995 | -15.37 | 0 | 12.264 | 10.101 | -59.88595 | 0.56064 | 1 |
| 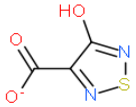 | 0 | 2844 | C3H2N2O3S | -30.05 | 10 | 1 | -9.9331 | -1.3809 | -11.12 | 0 | 10.552 | -2.112 | -34.66422 | 0.44068 | 1 |
| 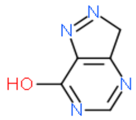 | 0 | 2835 | C5H4N4O | -30 | 14 | 0 | -13.645 | -2.6151 | -11.1 | 0.2172 | 16.109 | 7.8144 | -38.63958 | 0.43084 | 1 |
| 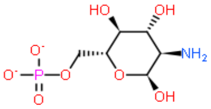 | 0 | 2377 | C6H14NO8P | -29.98 | 28 | 7 | -14.457 | -1.9826 | -11.87 | 0 | 17.507 | 3.7315 | -85.65424 | 0.97273 | 1 |
| 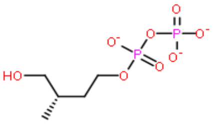 | 0 | 1577 | C5H14O8P2 | -29.92 | 26 | 8 | -18.226 | -2.3844 | -17.35 | 0.8516 | 20.852 | 21.509 | -73.10461 | 0.94073 | 1 |
| 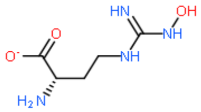 | 0 | 2120 | C5H12N4O3 | -29.88 | 23 | 8 | -15.021 | -1.8217 | -12.24 | 0 | 18.278 | 4.671 | -51.46402 | 0.81657 | 1 |
| 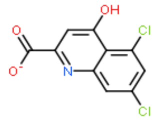 | 0 | 1696 | DK1 | -29.87 | 20 | 1 | -9.8805 | -3.1476 | -15.31 | 2.3401 | 14.005 | 0.0513 | -69.22984 | 0.95058 | 1 |

| | | | | | | | | | | | | | | | |
|---|---|---|---|---|---|---|---|---|---|---|---|---|---|---|---|
| 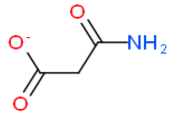 | 0 | 2370 | C3H5NO3 | -29.86 | 11 | 2 | -10.583 | -0.9366 | -11.16 | 0.1804 | 12.237 | -2.708 | -52.40779 | 0.34577 | 1 |
| 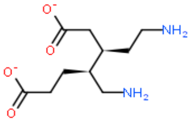 | 0 | 2579 | C10H20N2O4 | -29.82 | 34 | 11 | -16.632 | -2.9124 | -14.59 | 9.148 | 19.061 | 3.6611 | -83.39315 | 0.9898 | 1 |
| 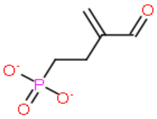 | 0 | 2892 | C5H9O4P | -29.81 | 17 | 3 | -13.14 | -1.7627 | -13.92 | 3.1452 | 12.379 | 8.4248 | -67.99323 | 0.49478 | 1 |
| 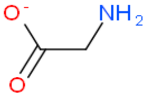 | 0 | 41 | C2H5NO2 | -29.79 | 9 | 2 | -11.066 | -0.9192 | -8.18 | 0 | 9.5021 | -2.012 | -26.47 | 0.29296 | 1 |
| 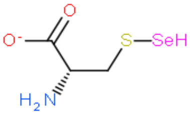 | 0 | 2740 | C3H7NO2SSe | -29.76 | 14 | 5 | -11.536 | -1.3515 | -11.86 | 0 | 12.094 | 0.1814 | -35.30131 | 0.53755 | 1 |
| 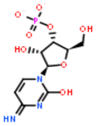 | 0 | 1725 | C9H14N3O8P | -29.73 | 33 | 7 | -16.58 | -2.2253 | -17.38 | 0 | 22.688 | 14.946 | -92.0282 | 1.11124 | 1 |
| 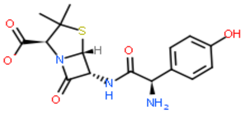 | 0 | 932 | 1060 | -29.62 | 43 | 5 | -12.931 | -3.9348 | -24.89 | 7.9489 | 21.071 | 10.681 | -116.2362 | 1.33601 | 1 |
| 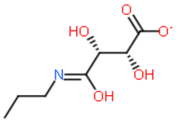 | 0 | 3061 | C7H13NO5 | -29.62 | 25 | 8 | -11.74 | -2.6216 | -15.08 | 0 | 10.897 | 5.9898 | -94.88487 | 0.87629 | 1 |
| 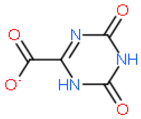 | 0 | 2890 | C4H3N3O4 | -29.59 | 13 | 1 | -11.172 | -0.5595 | -13.89 | 0 | 13.095 | 3.8128 | -68.81377 | 0.5125 | 1 |
| 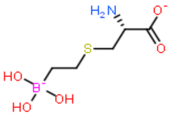 | 0 | 3372 | C5H13BNO5S | -29.56 | 25 | 10 | -12.994 | -2.0706 | -12.98 | 0 | 22.698 | -8.1 | -54.41414 | 0.91893 | 1 |
| 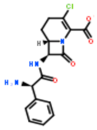 | 0 | 337 | C16H16ClN3O4 | -29.52 | 39 | 5 | -11.098 | -4.6059 | -25.67 | 2.4928 | 18.813 | 13.199 | -114.6391 | 1.3535 | 1 |

| | | | | | | | | | | | | | | | |
|---|---|---|---|---|---|---|---|---|---|---|---|---|---|---|---|
| 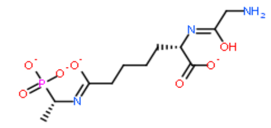 | 0 | 1642 | C11H21N3O7P | -29.38 | 40 | 12 | -14.953 | -3.3719 | -19.13 | 0 | 21.471 | 9.9047 | -124.1584 | 1.39762 | 1 |
| 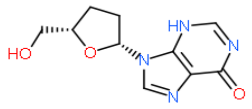 | 0 | 779 | 900 | -29.37 | 29 | 3 | -11.672 | -3.0803 | -19.03 | 1.7199 | 16.796 | 8.8303 | -64.70206 | 1.09239 | 1 |
| 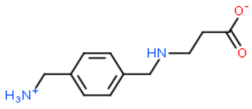 | 0 | 2656 | C11H17N2O2 | -29.3 | 31 | 6 | -12.896 | -3.232 | -16.08 | 1.9021 | 18.814 | 4.1184 | -75.26597 | 1.11046 | 1 |
| 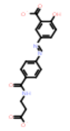 | 0 | 890 | C17H15N3O6 | -29.25 | 39 | 4 | -11.601 | -4.7202 | -21.8 | 0 | 22.267 | 9.0386 | -117.0649 | 1.65803 | 1 |
| 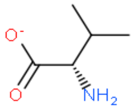 | 0 | 57 | C5H11NO2 | -29.24 | 18 | 3 | -11.407 | -2.1738 | -10.8 | 0 | 10.472 | 3.3673 | -56.24204 | 0.50153 | 1 |
| 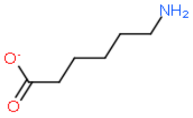 | 0 | 401 | 513 | -29.15 | 21 | 6 | -12.938 | -2.4402 | -12.68 | 2.9229 | 14.403 | 2.6437 | -58.92596 | 0.74903 | 1 |
| 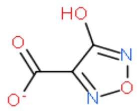 | 0 | 2137 | C3H2N2O4 | -29.12 | 10 | 1 | -9.9886 | -0.7161 | -11.12 | 0 | 10.779 | -1.412 | -39.64722 | 0.42798 | 1 |
| 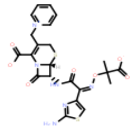 | 0 | 328 | 438 | -29.11 | 58 | 7 | -11.534 | -6.8806 | -30.16 | 5.6426 | 26.498 | 11.186 | -135.1379 | 2.01774 | 1 |
| 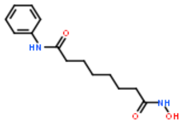 | 0 | 2274 | C14H20N2O3 | -29.08 | 39 | 10 | -11.09 | -4.5962 | -24.21 | 3.3574 | 17.229 | 8.0726 | -100.4089 | 1.24123 | 1 |
| 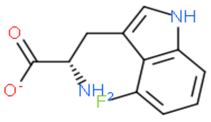 | 0 | 3057 | C11H11FN2O2 | -29.07 | 26 | 4 | -11.655 | -2.8373 | -14.84 | 0 | 15.543 | 4.7067 | -86.23952 | 0.95565 | 1 |
| 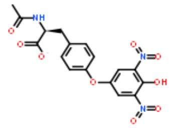 | 0 | 2920 | P28 | -29.06 | 43 | 8 | -10.918 | -4.5728 | -26.94 | 3.9885 | 20.888 | 8.6505 | -128.746 | 1.59314 | 1 |

| | | | | | | | | | | | | | | | |
|---|---|---|---|---|---|---|---|---|---|---|---|---|---|---|---|
| 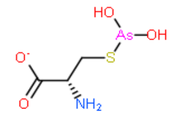 | 0 | 2966 | C3H8AsNO4S | -28.98 | 17 | 7 | -13.68 | -1.4066 | -13.11 | 0.4221 | 18.641 | 1.8996 | -48.06886 | 0.7248 | 1 |
| 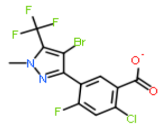 | 0 | 2950 | C12H6BrClF4N2O2 | -28.95 | 27 | 1 | -9.5187 | -4.7139 | -17.98 | 1.3534 | 14.386 | 6.1683 | -103.1666 | 1.43983 | 1 |
| 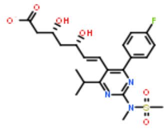 | 0 | 970 | C22H28FN3O6S | -28.93 | 60 | 11 | -13.364 | -5.8757 | -25.6 | 8.1358 | 17.417 | 14.959 | -111.958 | 1.53556 | 1 |
| 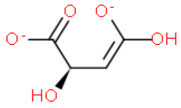 | 0 | 3018 | C4H4O5 | -28.91 | 13 | 3 | -12.173 | -1.0807 | -10.97 | 0 | 12.552 | 3.5886 | -59.10428 | 0.35789 | 1 |
| 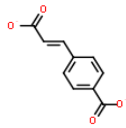 | 0 | 1883 | CIN | -28.9 | 20 | 3 | -9.8352 | -2.7153 | -11.8 | 1.7791 | 11.316 | -2.657 | -64.67856 | 0.73456 | 1 |
| 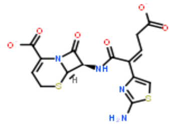 | 0 | 1223 | m | -28.9 | 39 | 4 | -9.9039 | -4.0348 | -25.25 | 0.5929 | 24.318 | 4.7252 | -122.7613 | 1.53778 | 1 |
| 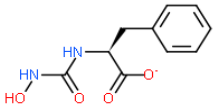 | 0 | 2373 | C10H12N2O4 | -28.88 | 27 | 7 | -11.725 | -2.8858 | -21.46 | 0.9901 | 15.068 | 12.246 | -72.60806 | 0.89751 | 1 |
| 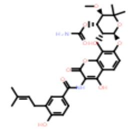 | 0 | 924 | m | -28.88 | 80 | 6 | -13.995 | -7.4557 | -34.46 | 8.4912 | 34.241 | 17.591 | -178.4767 | 2.77433 | 1 |
| 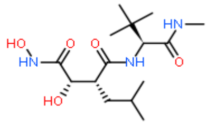 | 0 | 667 | 786 | -28.87 | 52 | 11 | -13.612 | -4.4628 | -24.38 | 0.7207 | 21.174 | 15.187 | -130.2943 | 1.78905 | 1 |
| 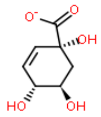 | 0 | 2506 | C7H10O5 | -28.8 | 21 | 4 | -9.1448 | -2.2779 | -11.55 | 0 | 9.0281 | -2.658 | -87.11324 | 0.65355 | 1 |
| 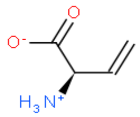 | 0 | 1581 | C4H7NO2 | -28.79 | 14 | 2 | -10.293 | -1.7205 | -11.52 | 0.3224 | 9.1493 | 2.5281 | -37.16056 | 0.42137 | 1 |

| | | | | | | | | | | | | | | | |
|---|---|---|---|---|---|---|---|---|---|---|---|---|---|---|---|
| 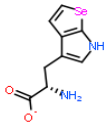 | 0 | 2734 | C9H10N2O2Se | -28.76 | 23 | 4 | -11.012 | -2.4424 | -17.76 | 0 | 16.599 | 5.5231 | -55.04586 | 0.88882 | 1 |
| 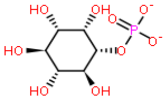 | 0 | 3200 | C6H11O9P | -28.76 | 27 | 7 | -12.883 | -1.9425 | -11.96 | 0 | 14.794 | 2.7289 | -82.06668 | 0.94631 | 1 |
| 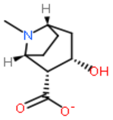 | 0 | 1321 | 1525 | -28.68 | 27 | 2 | -11.229 | -2.9883 | -8.898 | 0 | 10.299 | 2.5799 | -86.50349 | 0.76654 | 1 |
| 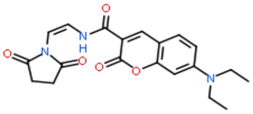 | 0 | 2504 | MDC | -28.67 | 49 | 2 | -13.536 | -5.5352 | -24.95 | 9.7548 | 19.675 | 18.836 | -127.5489 | 2.08006 | 1 |
| 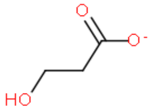 | 0 | 3332 | C3H6O3 | -28.65 | 11 | 3 | -11.132 | -1.2726 | -8.47 | 0 | 8.8734 | 0.5812 | -45.62653 | 0.33439 | 1 |
| 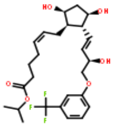 | 0 | 181 | 287 | -28.57 | 70 | 15 | -13.208 | -7.1167 | -32.84 | 6.346 | 24.143 | 17.484 | -189.1949 | 2.23014 | 1 |
| 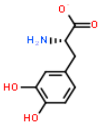 | 0 | 1104 | 1235 | -28.53 | 24 | 4 | -11.321 | -2.3627 | -16.53 | 0 | 15.526 | 6.3355 | -64.8416 | 0.89076 | 1 |
| 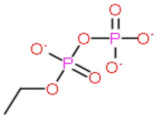 | 0 | 1576 | C2H8O7P2 | -28.5 | 16 | 4 | -14.203 | -1.7203 | -12.55 | 0 | 16.036 | 10.16 | -58.11137 | 0.5978 | 1 |
| 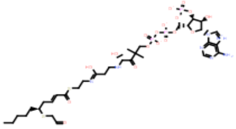 | 0 | 3341 | C33H55N7O18P3S2 | -28.5 | 114 | 32 | -14.037 | -7.8764 | -46.29 | 11.499 | 38.249 | 5.7411 | -182.5452 | 3.59493 | 1 |
| 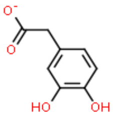 | 0 | 1488 | DHY | -28.48 | 19 | 2 | -10.871 | -2.1603 | -13.1 | 0.6016 | 13.693 | 2.6398 | -77.24678 | 0.98563 | 1 |
| 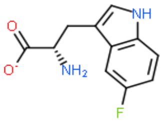 | 0 | 2991 | C11H11FN2O2 | -28.47 | 26 | 4 | -10.851 | -3.1141 | -18.1 | 0.72 | 15.876 | 6.7152 | -72.30623 | 0.96286 | 1 |

| | | | | | | | | | | | | | | | |
|---|---|---|---|---|---|---|---|---|---|---|---|---|---|---|---|
| 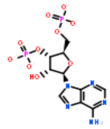 | 0 | 1589 | Adenosine | -28.44 | 38 | 7 | -12.991 | -3.4657 | -17.4 | 0 | 24.231 | 2.6953 | -108.1425 | 1.28864 | 1 |
| 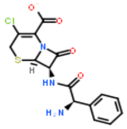 | 0 | 714 | C15H14ClN3O4S | -28.42 | 37 | 5 | -10.78 | -4.349 | -24.14 | 2.2285 | 19.504 | 10.523 | -102.3246 | 1.43628 | 1 |
| 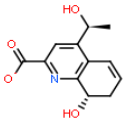 | 0 | 2293 | QUA | -28.4 | 29 | 4 | -10.135 | -3.2421 | -18.35 | 0.0388 | 15.421 | 5.71 | -80.50603 | 1.09611 | 1 |
| 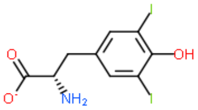 | 0 | 3048 | TYI | -28.35 | 23 | 4 | -12.191 | -3.5291 | -17 | 2.071 | 18.438 | 6.6499 | -75.17793 | 1.39042 | 1 |
| 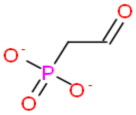 | 0 | 2856 | C2H5O4P | -28.33 | 10 | 2 | -11.299 | -0.9758 | -10.59 | 0 | 11.764 | 2.2425 | -52.14937 | 0.3585 | 1 |
| 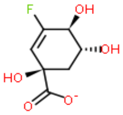 | 0 | 2492 | C7H9FO5 | -28.32 | 21 | 4 | -10.753 | -2.2609 | -13.61 | 0 | 15.763 | -0.32 | -65.18962 | 0.68386 | 1 |
| 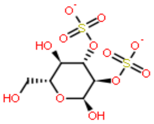 | 0 | 2580 | C6H12O12S2 | -28.32 | 30 | 8 | -13.37 | -2.2457 | -15.29 | 0 | 21.109 | 2.5208 | -99.9548 | 1.22406 | 1 |
| 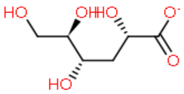 | 0 | 2980 | C6H12O6 | -28.31 | 23 | 9 | -15.363 | -1.566 | -13.13 | 2.4876 | 15.769 | 9.1253 | -70.19099 | 0.72585 | 1 |
| 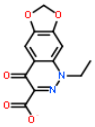 | 0 | 708 | 827 | -28.29 | 28 | 2 | -9.4631 | -3.0271 | -18.25 | 0 | 15.267 | 4.9599 | -94.58163 | 1.01925 | 1 |
| 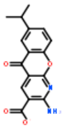 | 0 | 901 | 1025 | -28.29 | 35 | 2 | -9.1073 | -4.0867 | -17.78 | 0 | 14.373 | 4.9876 | -93.60377 | 1.24383 | 1 |
| 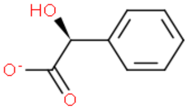 | 0 | 3031 | C8H8O3 | -28.14 | 18 | 3 | -10.265 | -2.5088 | -12.54 | 0 | 9.6064 | 4.6216 | -64.37365 | 0.54955 | 1 |

| | | | | | | | | | | | | | | | |
|---|---|---|---|---|---|---|---|---|---|---|---|---|---|---|---|
| 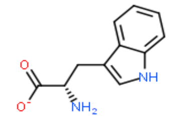 | 0 | 46 | 150 | -28.11 | 26 | 4 | -10.821 | -3.0144 | -18.02 | 0.4055 | 15.819 | 7.2652 | -69.13103 | 0.93624 | 1 |
| 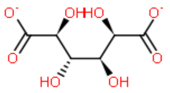 | 0 | 3254 | C6H10O8 | -28.1 | 22 | 9 | -12.942 | -1.2605 | -13.11 | 0 | 13.181 | 5.0306 | -70.8178 | 0.81025 | 1 |
| 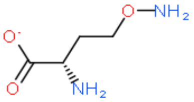 | 0 | 2525 | C4H10N2O3 | -28.08 | 18 | 6 | -14.038 | -1.476 | -12.74 | 1.1147 | 19.212 | 3.7683 | -44.21073 | 0.6046 | 1 |
| 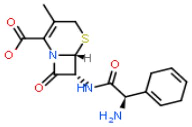 | 0 | 1167 | 1333 | -28.05 | 42 | 5 | -10.853 | -4.6802 | -21.89 | 0.1054 | 18.744 | 11.082 | -112.1455 | 1.39747 | 1 |
| 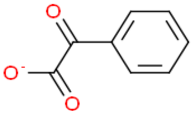 | 0 | 2023 | C8H6O3 | -28 | 16 | 2 | -10.135 | -2.3591 | -12.68 | 0.5579 | 11.776 | 2.3274 | -49.7379 | 0.6167 | 1 |
| 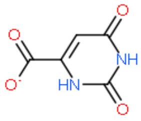 | 0 | 2006 | C5H4N2O4 | -27.98 | 14 | 1 | -10.587 | -1.0526 | -13.31 | 1.1846 | 14.122 | 1.3898 | -64.2122 | 0.62021 | 1 |
| 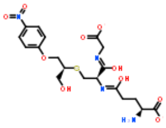 | 0 | 3349 | C19H26N4O10S | -27.93 | 58 | 20 | -14.492 | -4.498 | -27.79 | 0.4496 | 27.032 | 10.589 | -154.9419 | 2.19034 | 1 |
| 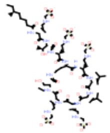 | 0 | 982 | 1111 | -27.87 | 212 | 46 | -23.362 | -12.14 | -57.26 | 12.884 | 53.088 | 32.556 | -262.3175 | 4.76368 | 1 |
| 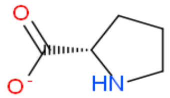 | 0 | 67 | 172 | -27.84 | 16 | 1 | -10.112 | -2.0829 | -12 | 0 | 9.2772 | 5.426 | -49.44741 | 0.49907 | 1 |
| 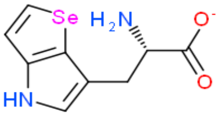 | 0 | 3176 | C9H10N2O2Se | -27.83 | 23 | 4 | -11.084 | -2.619 | -17.8 | 1.4402 | 16.391 | 6.3811 | -56.80362 | 0.88526 | 1 |
| 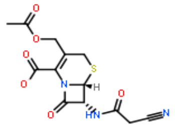 | 0 | 1222 | 1414 | -27.82 | 35 | 5 | -12.433 | -3.6913 | -22.07 | 1.1933 | 22.991 | 10.945 | -112.7909 | 1.56041 | 1 |

| | | | | | | | | | | | | | | | |
|---|---|---|---|---|---|---|---|---|---|---|---|---|---|---|---|
| 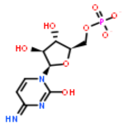 | 0 | 2189 | C9H14N3O8P | -27.79 | 33 | 7 | -14.85 | -2.1495 | -10.47 | 0 | 21.227 | 2.3766 | -76.97506 | 1.06456 | 1 |
| 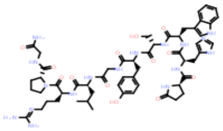 | 0 | 531 | 644 | -27.77 | 160 | 33 | -18.946 | -9.7556 | -64.38 | 16.07 | 45.307 | 41.907 | -285.0475 | 4.00003 | 1 |
| 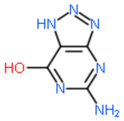 | 0 | 1456 | C4H4N6O | -27.76 | 15 | 0 | -13.837 | -1.8 | -12.65 | 0.8162 | 18.907 | 9.7619 | -33.06302 | 0.47047 | 1 |
| 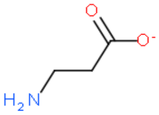 | 0 | 2794 | C3H7NO2 | -27.75 | 12 | 3 | -11.824 | -1.2835 | -7.911 | 1.0038 | 12.228 | -0.965 | -31.63078 | 0.30418 | 1 |
| 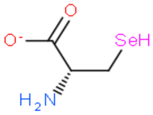 | 0 | 2085 | C3H7NO2Se | -27.74 | 13 | 4 | -11.15 | -1.2922 | -11.54 | 0 | 13.201 | 0.8797 | -35.04359 | 0.47297 | 1 |
| 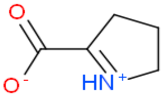 | 0 | 2541 | C5H7NO2 | -27.72 | 15 | 1 | -9.7673 | -2.0009 | -13.37 | 0.15 | 8.1251 | 7.4422 | -47.33859 | 0.52835 | 1 |
| 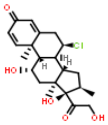 | 0 | 135 | C22H29ClO5 | -27.7 | 57 | 5 | -14.043 | -4.8274 | -15.71 | 4.3461 | 17.473 | 12.219 | -122.3495 | 1.58488 | 1 |
| 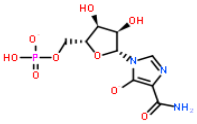 | 0 | 1709 | C9H12N3O9P | -27.7 | 34 | 7 | -12.689 | -2.3343 | -21.89 | 0 | 23.977 | 8.6064 | -104.1764 | 1.22949 | 1 |
| 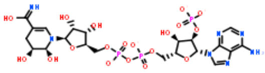 | 0 | 2061 | C21H32N7O19P3 | -27.68 | 78 | 18 | -19.682 | -4.0641 | -35.81 | 8.12 | 42.247 | 20.141 | -198.1358 | 2.57639 | 1 |
| 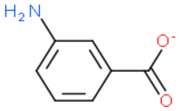 | 0 | 1813 | C7H7NO2 | -27.65 | 16 | 1 | -9.7779 | -2.3729 | -12.14 | 0.6525 | 11.208 | 2.155 | -42.7917 | 0.45966 | 1 |
| 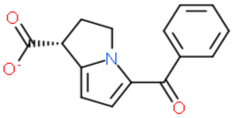 | 0 | 355 | 465 | -27.65 | 31 | 2 | -8.6009 | -3.7827 | -18.67 | 2.2567 | 11.472 | 6.2916 | -99.60377 | 0.94048 | 1 |

| | | | | | | | | | | | | | | | |
|---|---|---|---|---|---|---|---|---|---|---|---|---|---|---|---|
| 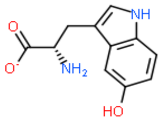 | 0 | 2655 | C11H12N2O3 | -27.65 | 27 | 4 | -10.826 | -2.8708 | -18.53 | 0.0107 | 17.165 | 7.4417 | -72.10713 | 0.98239 | 1 |
| 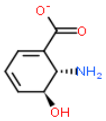 | 0 | 1881 | C7H9NO3 | -27.63 | 19 | 3 | -11.474 | -2.0077 | -15.36 | 0 | 12.799 | 10.154 | -50.2099 | 0.56382 | 1 |
| 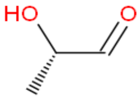 | 0 | 3415 | C3H6O2 | -27.63 | 11 | 2 | -11.558 | -1.422 | -7.394 | 0 | 6.8688 | 5.4716 | -41.46944 | 0.35972 | 1 |
| 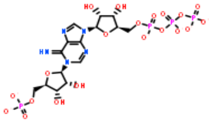 | 0 | 1450 | C15H25N5O20P4 | -27.6 | 63 | 16 | -18.335 | -3.5056 | -22.76 | 0 | 38.634 | 8.0566 | -101.0633 | 2.44434 | 1 |
| 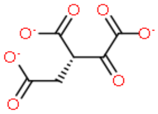 | 0 | 1943 | C6H6O7 | -27.58 | 16 | 5 | -13.731 | -1.1549 | -12.04 | 3.6963 | 16.832 | 3.2313 | -81.21442 | 0.6345 | 1 |
| 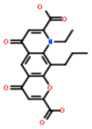 | 0 | 600 | 716 | -27.53 | 42 | 5 | -9.3182 | -3.8562 | -22.98 | 3.7256 | 15.638 | 7.0251 | -115.6957 | 1.29544 | 1 |
| 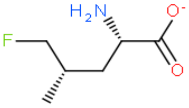 | 0 | 2270 | C6H12FNO2 | -27.53 | 21 | 5 | -11.39 | -2.5472 | -11.51 | 0.314 | 14.333 | 0.8537 | -61.98471 | 0.75882 | 1 |
| 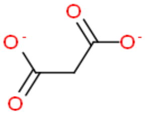 | 0 | 1953 | C3H2O4 | -27.51 | 9 | 2 | -11.857 | -0.7991 | -7.444 | 2.2821 | 11.578 | -1.194 | -52.2772 | 0.31102 | 1 |
| 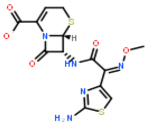 | 0 | 1166 | 1332 | -27.49 | 37 | 3 | -14.203 | -5.0518 | -17.21 | 5.7692 | 21.034 | 12.117 | -101.1509 | 1.42193 | 1 |
| 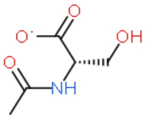 | 0 | 2081 | C5H9NO4 | -27.49 | 18 | 4 | -12.082 | -1.9893 | -13.58 | 5.042 | 14.158 | 2.9565 | -58.63723 | 0.57892 | 1 |
| 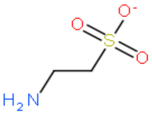 | 0 | 1720 | C2H7NO3S | -27.48 | 13 | 3 | -10.849 | -1.2689 | -12.77 | 0.8106 | 11.456 | 4.1049 | -38.44535 | 0.37475 | 1 |

| | | | | | | | | | | | | | | | |
|---|---|---|---|---|---|---|---|---|---|---|---|---|---|---|---|
| 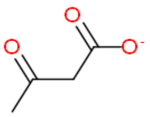 | 0 | 1544 | C4H6O3 | -27.47 | 12 | 2 | -11.013 | -1.5955 | -9.679 | 0.3497 | 11.519 | 1.6517 | -45.50407 | 0.4388 | 1 |
| 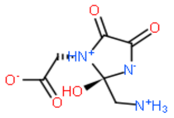 | 0 | 1575 | C6H10N3O5 | -27.46 | 23 | 4 | -14.019 | -1.4885 | -13.37 | 0 | 17.921 | 9.8963 | -56.64885 | 0.63967 | 1 |
| 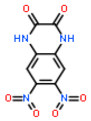 | 0 | 3399 | C8H4N4O6 | -27.45 | 22 | 2 | -9.0784 | -2.1309 | -18.27 | 0.007 | 13.197 | 6.2353 | -66.66212 | 1.08103 | 1 |
| 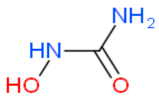 | 0 | 881 | CH4N2O2 | -27.45 | 9 | 2 | -11.225 | -0.0774 | -10.39 | 0 | 12.264 | 1.5067 | -6.299998 | 0.23042 | 1 |
| 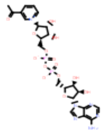 | 0 | 3037 | C22H28N6O14P2 | -27.44 | 71 | 14 | -18.597 | -4.936 | -37.85 | 11.143 | 32.863 | 31.864 | -168.6235 | 2.29996 | 1 |
| 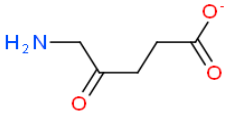 | 0 | 735 | 855 | -27.42 | 17 | 5 | -14.88 | -1.7622 | -10.68 | 4.7287 | 16.209 | 6.1962 | -57.39304 | 0.57736 | 1 |
| 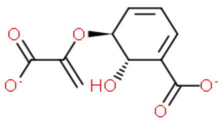 | 0 | 2498 | m | -27.41 | 24 | 4 | -10.626 | -2.4927 | -15.26 | 0 | 14.614 | 4.6064 | -79.76115 | 0.88717 | 1 |
| 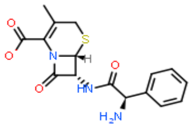 | 0 | 454 | 567 | -27.4 | 40 | 5 | -10.875 | -4.3729 | -23.44 | 2.085 | 19.886 | 11.086 | -108.6698 | 1.44507 | 1 |
| 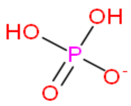 | 0 | 2534 | H2O4P | -27.4 | 7 | 2 | -10.938 | -0.3382 | -8.606 | 0 | 13.342 | -3.19 | -20.57854 | 0.21376 | 1 |
| 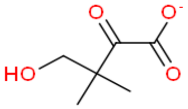 | 0 | 3432 | C6H10O4 | -27.4 | 19 | 4 | -11.433 | -2.0756 | -11.34 | 0 | 13.826 | 2.2621 | -71.24982 | 0.5534 | 1 |
| 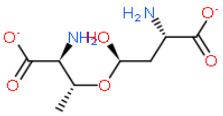 | 0 | 1594 | C8H16N2O6 | -27.4 | 30 | 10 | -13.329 | -2.4714 | -15.47 | 0 | 20.965 | 2.6203 | -94.49754 | 1.21582 | 1 |

| | | | | | | | | | | | | | | | |
|---|---|---|---|---|---|---|---|---|---|---|---|---|---|---|---|
| 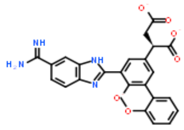 | 0 | 1842 | C25H20N4O6 | -27.39 | 54 | 4 | -14.52 | -5.0472 | -27.15 | 5.1496 | 26.345 | 22.163 | -136.7065 | 1.55247 | 1 |
| 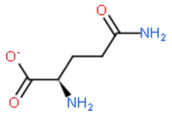 | 0 | 1927 | C5H10N2O3 | -27.37 | 19 | 5 | -13.102 | -1.4321 | -13.26 | 0.2018 | 17.173 | 6.0538 | -55.98634 | 0.63798 | 1 |
| 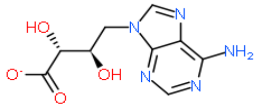 | 0 | 3408 | C9H11N5O4 | -27.33 | 28 | 6 | -12.514 | -2.8647 | -17.51 | 1.2182 | 20.982 | 5.5979 | -74.33099 | 0.88634 | 1 |
| 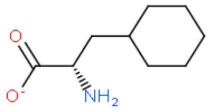 | 0 | 2585 | C9H17NO2 | -27.29 | 28 | 4 | -11.249 | -2.8521 | -14.1 | 1.4404 | 16.132 | 2.7592 | -81.47701 | 1.00193 | 1 |
| 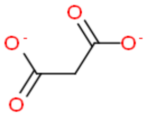 | 0 | 1928 | C3H4O4 | -27.24 | 9 | 2 | -11.795 | -0.7874 | -7.392 | 2.2662 | 11.547 | -1.061 | -52.40287 | 0.31102 | 1 |
| 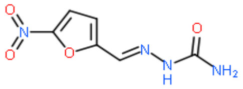 | 0 | 229 | 336 | -27.24 | 20 | 2 | -9.1095 | -1.6827 | -16.66 | 0 | 13.07 | 3.9364 | -50.94732 | 0.95803 | 1 |
| 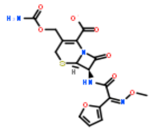 | 0 | 983 | C16H16N4O8S | -27.23 | 44 | 5 | -16.072 | -4.7218 | -25.65 | 13.488 | 27.012 | 16.513 | -119.3621 | 1.83553 | 1 |
| 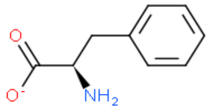 | 0 | 2284 | C9H11NO2 | -27.23 | 22 | 4 | -10.845 | -2.7849 | -16.91 | 1.9997 | 13.459 | 8.0272 | -59.3247 | 0.74054 | 1 |
| 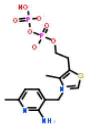 | 0 | 1448 | C13H19N3O7P2S | -27.21 | 44 | 9 | -12.979 | -4.2229 | -21.51 | 0 | 21.939 | 12.294 | -118.181 | 1.57287 | 1 |
| 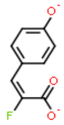 | 0 | 2123 | C9H6FO3 | -27.21 | 18 | 1 | -9.9385 | -2.8891 | -13.75 | 0.3544 | 12.922 | 4.7171 | -57.2142 | 0.83549 | 1 |
| 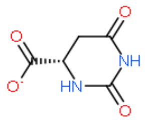 | 0 | 1886 | C5H6N2O4 | -27.2 | 16 | 1 | -12.83 | -1.1883 | -10.37 | 0.4615 | 15.765 | 5.6417 | -79.37022 | 0.55089 | 1 |

| | | | | | | | | | | | | | | | |
|---|---|---|---|---|---|---|---|---|---|---|---|---|---|---|---|
| 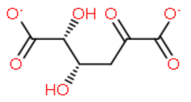 | 0 | 2918 | C6H6O7 | -27.19 | 19 | 7 | -12.11 | -1.4423 | -11.5 | 1.957 | 13.923 | 0.1519 | -61.75931 | 0.76145 | 1 |
| 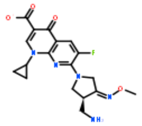 | 0 | 1025 | C18H20FN5O4 | -27.1 | 47 | 4 | -11.136 | -4.4478 | -22.51 | 5.3741 | 21.602 | 7.1746 | -118.7291 | 1.44003 | 1 |
| 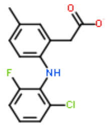 | 0 | 1141 | C15H13ClFNO2 | -27.08 | 32 | 2 | -9.5556 | -4.7472 | -18.21 | 3.0744 | 11.53 | 10.295 | -114.1252 | 1.49446 | 1 |
| 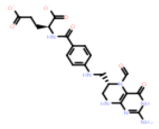 | 0 | 537 | C20H23N7O7 | -27.08 | 55 | 8 | -16.92 | -3.4556 | -19.33 | 4.7425 | 21.898 | 20.154 | -116.7951 | 1.60217 | 1 |
| 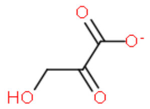 | 0 | 2647 | C3H4O4 | -27.05 | 10 | 3 | -10.351 | -0.8962 | -9.681 | 0 | 10.324 | -0.188 | -34.36427 | 0.33501 | 1 |
| 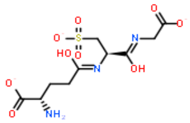 | 0 | 2698 | C10H17N3O9S | -27.03 | 37 | 13 | -14.95 | -2.6909 | -20.06 | 0 | 21.094 | 13.687 | -118.5875 | 1.48861 | 1 |
| 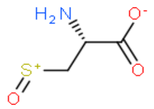 | 0 | 3053 | C3H7NO3S | -27.03 | 14 | 4 | -12.333 | -1.1658 | -11.76 | 2.3596 | 14.573 | 2.983 | -59.97129 | 0.42177 | 1 |
| 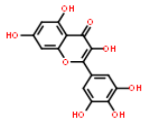 | 0 | 2114 | C15H10O8 | -27.02 | 33 | 0 | -9.9602 | -2.9617 | -21.25 | 0 | 18.566 | 11.336 | -103.4845 | 1.5083 | 1 |
| 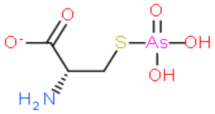 | 0 | 3027 | C3H8AsNO5S | -27.02 | 18 | 7 | -12.974 | -1.2188 | -15.77 | 0 | 16.579 | 8.7247 | -41.8671 | 0.69355 | 1 |
| 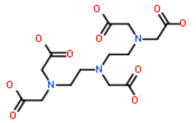 | 0 | 670 | C14H20GdN3O10 | -26.98 | 45 | 16 | -17.774 | -3.3245 | -22.39 | 6.3861 | 25.425 | 15.325 | -120.6866 | 1.57894 | 1 |
| 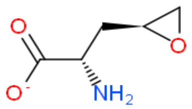 | 0 | 2872 | C5H9NO3 | -26.96 | 17 | 4 | -11.723 | -2.0062 | -11.76 | 1.6813 | 14.228 | 2.6123 | -45.76444 | 0.55281 | 1 |

| | | | | | | | | | | | | | | | |
|---|---|---|---|---|---|---|---|---|---|---|---|---|---|---|---|
| 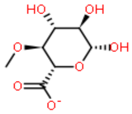 | 0 | 2436 | C7H12O7 | -26.96 | 25 | 5 | -13.085 | -2.2054 | -13.71 | 4.2288 | 15.454 | 6.3728 | -97.86655 | 0.78675 | 1 |
| 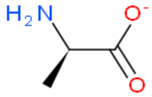 | 0 | 1565 | C3H7NO2 | -26.95 | 12 | 2 | -10.752 | -1.3081 | -9.18 | 0 | 11.786 | 0.4157 | -33.70616 | 0.33021 | 1 |
| 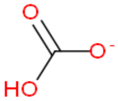 | 0 | 1202 | 1390 | -26.94 | 5 | 1 | -9.7797 | -0.2205 | -6.047 | 0 | 9.9741 | -6.192 | -13.99516 | 0.14296 | 1 |
| 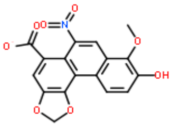 | 0 | 2357 | 9AR | -26.93 | 36 | 2 | -9.3566 | -3.8615 | -20.92 | 2.1512 | 17.25 | 7.2952 | -110.8842 | 1.54842 | 1 |
| 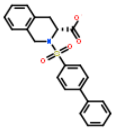 | 0 | 2888 | BSI | -26.88 | 46 | 4 | -9.1138 | -5.7237 | -26.07 | 3.048 | 15.576 | 15.418 | -130.4325 | 1.61202 | 1 |
| 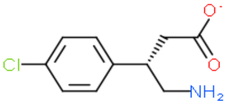 | 0 | 76 | 181 | -26.88 | 25 | 5 | -10.536 | -3.0096 | -13.47 | 0.9205 | 12.278 | 3.7895 | -79.28893 | 0.698 | 1 |
| 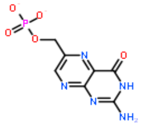 | 0 | 3244 | C7H8N5O5P | -26.88 | 24 | 3 | -17.139 | -2.0998 | -16.55 | 0.2418 | 24.733 | 21.128 | -83.41714 | 1.0875 | 1 |
| 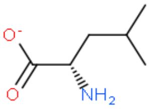 | 0 | 45 | C6H13NO2 | -26.87 | 21 | 4 | -11.331 | -2.5203 | -11.25 | 0.5518 | 14.227 | 2.034 | -55.27113 | 0.712 | 1 |
| 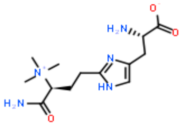 | 0 | 2904 | C13H24N5O3 | -26.86 | 44 | 9 | -16.812 | -4.0429 | -24.22 | 9.1193 | 23.927 | 20.891 | -105.0684 | 1.40298 | 1 |
| 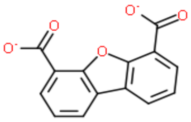 | 0 | 3327 | DBF | -26.73 | 25 | 2 | -9.685 | -3.101 | -17.1 | 0.063 | 15.458 | 6.2431 | -100.7292 | 1.20926 | 1 |
| 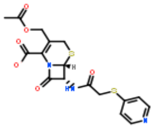 | 0 | 1010 | 1139 | -26.69 | 44 | 6 | -14.605 | -4.6115 | -28.07 | 6.8192 | 24.235 | 23.57 | -155.4732 | 1.792 | 1 |

| | | | | | | | | | | | | | | | |
|---|---|---|---|---|---|---|---|---|---|---|---|---|---|---|---|
| 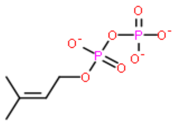 | 0 | 1564 | DMA | -26.69 | 23 | 5 | -13.783 | -2.5529 | -17.27 | 0 | 18.284 | 15.836 | -90.24934 | 0.98736 | 1 |
| 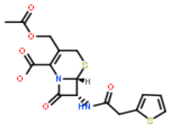 | 0 | 346 | 456 | -26.67 | 41 | 6 | -12.33 | -4.872 | -26.25 | 4.0498 | 23.218 | 16.055 | -115.8844 | 1.72589 | 1 |
| 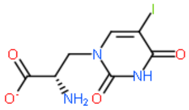 | 0 | 2522 | C7H8IN3O4 | -26.65 | 22 | 4 | -12.592 | -2.6126 | -18.29 | 2.7014 | 19.141 | 10.409 | -68.33269 | 1.22806 | 1 |
| 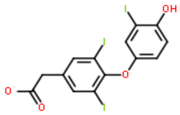 | 0 | 3255 | C14H9I3O4 | -26.64 | 29 | 4 | -11.088 | -4.9935 | -21.45 | 7.2167 | 18.761 | 8.195 | -85.30229 | 1.62787 | 1 |
| 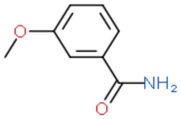 | 0 | 2762 | C8H9NO2 | -26.59 | 20 | 0 | -9.7641 | -2.8041 | -12.74 | 0 | 11.79 | 5.8932 | -39.66248 | 0.71941 | 1 |
| 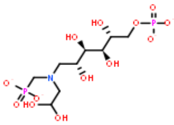 | 0 | 2169 | C9H23NO13P2 | -26.57 | 44 | 18 | -14.501 | -2.5402 | -13.3 | 0 | 16.568 | 2.8717 | -117.5219 | 1.1637 | 1 |
| 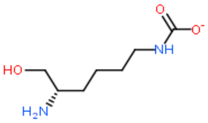 | 0 | 2173 | C7H16N2O3 | -26.56 | 27 | 9 | -14.17 | -2.5396 | -13.58 | 4.382 | 18.365 | 4.1864 | -75.56511 | 0.9761 | 1 |
| 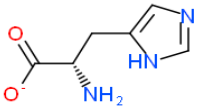 | 0 | 14 | 117 | -26.56 | 19 | 4 | -10.776 | -2.1551 | -13.01 | 0 | 13.732 | 3.7323 | -66.4136 | 0.75464 | 1 |
| 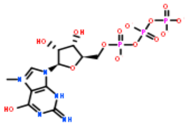 | 0 | 2431 | C11H19N5O14P3 | -26.54 | 48 | 11 | -17.19 | -2.7935 | -24.71 | 0 | 27.264 | 24.975 | -120.2686 | 1.4896 | 1 |
| 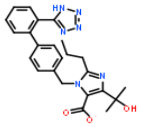 | 0 | 170 | C24H26N6O3 | -26.54 | 58 | 7 | -9.162 | -7.0422 | -33.76 | 1.7141 | 20.992 | 21.432 | -152.2007 | 2.0829 | 1 |
| 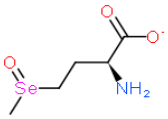 | 0 | 2654 | C5H11NO3Se | -26.52 | 20 | 5 | -11.359 | -2.0771 | -12.15 | 0 | 15.421 | 1.803 | -47.63789 | 0.7929 | 1 |

| | | | | | | | | | | | | | | | |
|---|---|---|---|---|---|---|---|---|---|---|---|---|---|---|---|
| 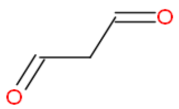 | 0 | 2746 | C3H4O2 | -26.52 | 9 | 2 | -10.446 | -0.8181 | -9.968 | 0 | 8.6281 | 4.1733 | -43.76586 | 0.2989 | 1 |
| 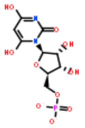 | 0 | 2590 | C9H13N2O10P | -26.5 | 33 | 7 | -17.876 | -2.4181 | -20.37 | 8.5214 | 26.508 | 17.455 | -123.6709 | 1.31414 | 1 |
| 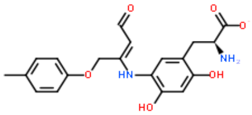 | 0 | 2266 | C20H22N2O6 | -26.47 | 49 | 6 | -13.055 | -5.1186 | -29.19 | 8.9349 | 25.835 | 16.426 | -147.1153 | 1.47666 | 1 |
| 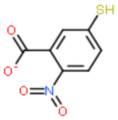 | 0 | 2474 | C7H5NO4S | -26.47 | 17 | 3 | -8.5658 | -1.8152 | -15.22 | 0.1363 | 12.198 | 0.9304 | -74.83105 | 0.83822 | 1 |
| 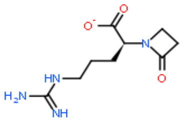 | 0 | 2208 | C9H16N4O3 | -26.44 | 31 | 7 | -13.586 | -2.7306 | -9.825 | 2.4101 | 17.747 | 0.955 | -73.29951 | 0.90966 | 1 |
| 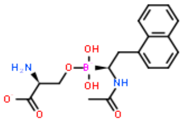 | 0 | 2396 | C17H22BN2O6 | -26.43 | 47 | 11 | -12.522 | -4.9858 | -29.3 | 3.2754 | 25.895 | 15.018 | -119.7119 | 1.49911 | 1 |
| 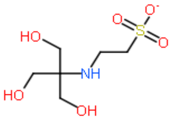 | 0 | 2110 | C6H15NO6S | -26.42 | 28 | 10 | -13.147 | -2.5167 | -12.18 | 0.6437 | 14.747 | 4.9413 | -55.16593 | 0.9601 | 1 |
| 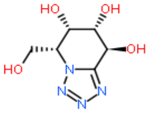 | 0 | 2036 | C6H10N4O4 | -26.41 | 24 | 5 | -17.115 | -2.0144 | -4.08 | 0 | 17.733 | 9.1257 | -54.42616 | 0.59124 | 1 |
| 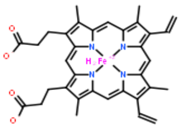 | 0 | 2303 | C34H34FeN4O4 | -26.39 | 73 | 6 | -10.39 | -7.8591 | -30.24 | 0.3201 | 23.364 | 21.336 | -160.4243 | 2.14561 | 1 |
| 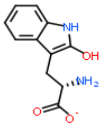 | 0 | 3325 | C11H12N2O3 | -26.39 | 27 | 4 | -10.757 | -2.5171 | -14.28 | 0 | 14.962 | 4.8094 | -87.27416 | 0.93074 | 1 |
| 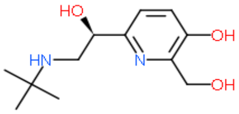 | 0 | 1145 | 1291 | -26.37 | 37 | 7 | -13.282 | -4.0814 | -15.2 | 1.276 | 19.987 | 7.9006 | -72.10606 | 1.00689 | 1 |

| | | | | | | | | | | | | | | |
|---|---|---|---|---|---|---|---|---|---|---|---|---|---|---|
| 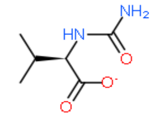 | 0 | 1623 | C6H12N2O3 | -26.36 | 22 | 4 | -13.599 | -2.0164 | -10.82 | 4.6961 | 15.113 | 5.5589 | -51.09389 | 0.54858 | 1 |
| 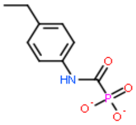 | 0 | 3277 | IS2 | -26.34 | 25 | 3 | -9.7951 | -3.187 | -14.92 | 0 | 15.381 | 3.2707 | -76.23614 | 1.08425 | 1 |
| 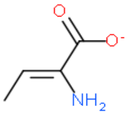 | 0 | 3361 | C4H7NO2 | -26.32 | 13 | 1 | -10.008 | -1.9442 | -11.23 | 0 | 11.368 | 3.6497 | -38.03035 | 0.38263 | 1 |
| 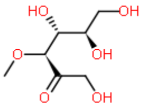 | 0 | 2174 | C7H14O6 | -26.29 | 27 | 10 | -14.921 | -2.8576 | -10.99 | 3.4803 | 16.093 | 6.3785 | -62.61613 | 0.8098 | 1 |
| 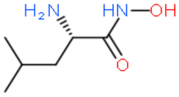 | 0 | 2985 | C6H14N2O2 | -26.28 | 24 | 6 | -13.029 | -2.4569 | -14.36 | 2.1421 | 16.678 | 8.0331 | -62.58296 | 0.65688 | 1 |
| 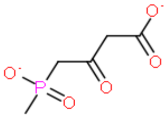 | 0 | 1609 | C5H9O5P | -26.27 | 18 | 4 | -12.108 | -2.1411 | -13.24 | 0.8251 | 15.43 | 6.926 | -61.11938 | 0.7174 | 1 |
| 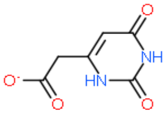 | 0 | 2739 | C6H6N2O4 | -26.27 | 17 | 2 | -11.271 | -1.5628 | -14.71 | 2.4685 | 15.201 | 5.9459 | -59.33777 | 0.67413 | 1 |
| 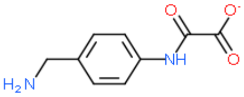 | 0 | 2156 | C9H10N2O3 | -26.26 | 23 | 3 | -9.3445 | -2.5744 | -16.48 | 0.5954 | 12.175 | 6.4997 | -72.02345 | 0.95912 | 1 |
| 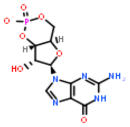 | 0 | 2057 | C10H12N5O7P | -26.2 | 34 | 2 | -11.389 | -2.7768 | -20.09 | 0 | 22.075 | 10.321 | -94.60291 | 1.14225 | 1 |
| 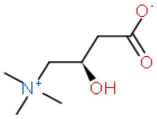 | 0 | 2369 | C7H16NO3 | -26.18 | 26 | 5 | -11.16 | -2.9439 | -13.48 | 1.8711 | 10.764 | 8.059 | -65.44894 | 0.77511 | 1 |
| 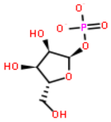 | 0 | 2788 | 439236 | -26.18 | 23 | 6 | -13.753 | -1.9427 | -11.88 | 0 | 17.332 | 7.8776 | -83.19175 | 0.85812 | 1 |

| | | | | | | | | | | | | | | | |
|---|---|---|---|---|---|---|---|---|---|---|---|---|---|---|---|
| 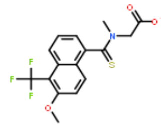 | 0 | 2122 | TOL | -26.13 | 37 | 4 | -8.7537 | -4.9655 | -21.3 | 3.8713 | 12.702 | 9.6879 | -109.3336 | 1.26165 | 1 |
| 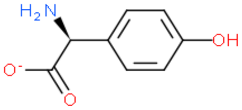 | 0 | 2326 | C8H9NO3 | -26.12 | 20 | 3 | -10.658 | -2.2747 | -13.66 | 0 | 13.781 | 5.8863 | -65.17857 | 0.61473 | 1 |
| 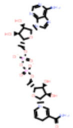 | 0 | 53 | 157 | -26.12 | 71 | 14 | -19.468 | -3.8737 | -37.28 | 7.8559 | 37.302 | 33.21 | -152.1474 | 2.26834 | 1 |
| 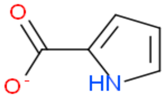 | 0 | 2271 | C5H5NO2 | -26.12 | 12 | 1 | -9.5665 | -1.7316 | -12.8 | 0.1644 | 11.251 | 4.4124 | -41.27562 | 0.46843 | 1 |
| 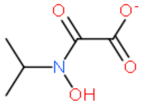 | 0 | 3058 | C5H9NO4 | -26.1 | 18 | 4 | -9.5585 | -2.1819 | -14.21 | 0 | 11.792 | 3.7997 | -57.97708 | 0.6565 | 1 |
| 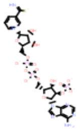 | 0 | 1545 | C21H28N7O16P3S | -26.1 | 72 | 15 | -13.251 | -4.8142 | -29.39 | 4.9545 | 32.941 | 5.0837 | -152.5344 | 2.33875 | 1 |
| 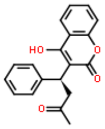 | 0 | 568 | C19H16O4 | -26.09 | 39 | 4 | -10.624 | -4.8637 | -21.83 | 5.8746 | 17.501 | 10.342 | -137.2396 | 1.47508 | 1 |
| 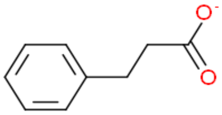 | 0 | 1785 | C9H10O2 | -26.07 | 20 | 3 | -8.5124 | -3.1017 | -15.62 | 0.4319 | 10.452 | 4.9511 | -54.79526 | 0.72038 | 1 |
| 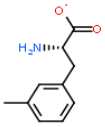 | 0 | 2403 | C10H13NO2 | -26.05 | 25 | 4 | -10.664 | -2.9337 | -13.83 | 0 | 14.894 | 4.7879 | -81.00131 | 0.92384 | 1 |
| 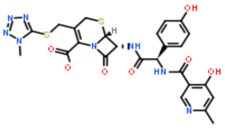 | 0 | 320 | C25H24N8O7S2 | -26 | 65 | 7 | -13.607 | -6.376 | -35.63 | 3.6915 | 33.868 | 25.019 | -169.1765 | 2.26351 | 1 |
| 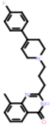 | 0 | 2761 | FRM | -25.93 | 52 | 5 | -9.8115 | -5.6645 | -25.74 | 5.96 | 15.52 | 15.462 | -116.6676 | 1.69011 | 1 |

| | | | | | | | | | | | | | | | |
|---|---|---|---|---|---|---|---|---|---|---|---|---|---|---|---|
| 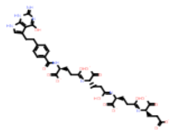 | 0 | 1971 | C35H42N8O15 | -25.91 | 95 | 27 | -22.422 | -5.1003 | -44.82 | 7.051 | 45.322 | 37.322 | -225.0981 | 3.22255 | 1 |
| 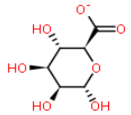 | 0 | 1746 | C6H10O7 | -25.91 | 22 | 5 | -11.787 | -1.6555 | -11.86 | 0 | 12.482 | 6.8572 | -67.62582 | 0.60937 | 1 |
| 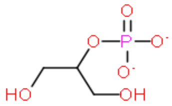 | 0 | 1559 | C3H9O6P | -25.88 | 17 | 6 | -13.092 | -1.7727 | -11.51 | 0 | 13.45 | 9.485 | -71.46438 | 0.51154 | 1 |
| 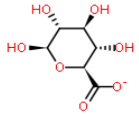 | 0 | 2838 | C6H10O7 | -25.83 | 22 | 5 | -11.117 | -1.6755 | -12.11 | 0 | 13.331 | 3.8399 | -57.7568 | 0.69839 | 1 |
| 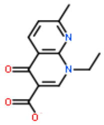 | 0 | 662 | 779 | -25.82 | 28 | 2 | -8.8626 | -3.3676 | -18.18 | 0.3314 | 14.028 | 7.7648 | -89.64646 | 1.05544 | 1 |
| 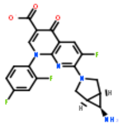 | 0 | 571 | C20H15F3N4O3 | -25.8 | 44 | 2 | -10.742 | -4.6179 | -23.61 | 6.3285 | 20.642 | 11.521 | -138.294 | 1.52017 | 1 |
| 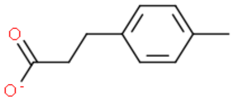 | 0 | 3069 | PPT | -25.78 | 23 | 3 | -8.5017 | -3.3736 | -14.82 | 0.218 | 10.651 | 4.3836 | -58.64853 | 0.86458 | 1 |
| 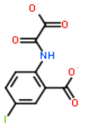 | 0 | 2382 | 878 | -25.74 | 20 | 2 | -10.266 | -2.4395 | -15.13 | 0.4287 | 14.024 | 7.5988 | -76.82797 | 1.00999 | 1 |
| 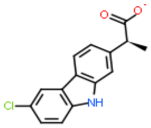 | 0 | 702 | 821 | -25.73 | 30 | 2 | -10.02 | -3.9342 | -15.5 | 4.3981 | 13.043 | 6.2561 | -92.50665 | 0.99598 | 1 |
| 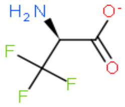 | 0 | 3431 | C3H4F3NO2 | -25.73 | 12 | 3 | -9.4494 | -1.7791 | -10.03 | 0 | 9.1346 | 1.1364 | -62.32594 | 0.37657 | 1 |
| 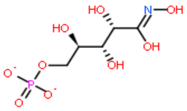 | 0 | 1851 | C5H12NO9P | -25.69 | 26 | 11 | -16.079 | -1.5338 | -14.78 | 0 | 21.771 | 11.927 | -77.47212 | 1.1004 | 1 |

| | | | | | | | | | | | | | | | |
|---|---|---|---|---|---|---|---|---|---|---|---|---|---|---|---|
| 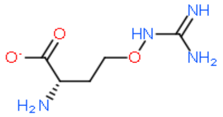 | 0 | 1610 | C5H12N4O3 | -25.69 | 23 | 6 | -14.125 | -1.5114 | -15.16 | 0 | 19.062 | 12.616 | -54.2697 | 0.72398 | 1 |
| 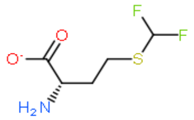 | 0 | 3451 | C5H9F2NO2S | -25.67 | 19 | 6 | -10.378 | -2.7491 | -11.92 | 0.0943 | 14.201 | 0.0631 | -61.1027 | 0.76416 | 1 |
| 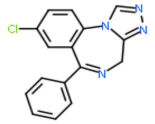 | 0 | 1085 | 3261 | -25.66 | 32 | 1 | -9.6865 | -5.4857 | -19.27 | 1.6594 | 15.775 | 12.67 | -105.5962 | 1.28657 | 1 |
| 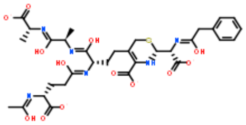 | 0 | 1893 | C32H42N6O13S | -25.66 | 90 | 25 | -15.786 | -6.2404 | -34.68 | 8.282 | 34.711 | 10.812 | -157.4913 | 2.6575 | 1 |
| 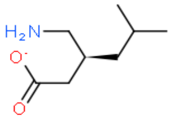 | 0 | 125 | C8H17NO2 | -25.63 | 27 | 6 | -10.769 | -2.9264 | -12.99 | 1.3492 | 11.26 | 5.6184 | -74.31747 | 0.76856 | 1 |
| 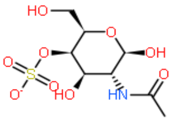 | 0 | 1645 | C8H15NO9S | -25.61 | 33 | 7 | -13.533 | -3.1158 | -14.34 | 0 | 19.078 | 9.9116 | -122.5004 | 1.2093 | 1 |
| 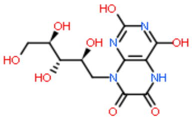 | 0 | 1962 | C11H14N4O8 | -25.59 | 37 | 9 | -14.666 | -2.8888 | -20.83 | 7.8498 | 25.305 | 7.8526 | -94.59047 | 1.47932 | 1 |
| 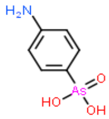 | 0 | 2701 | C6H8AsNO3 | -25.58 | 19 | 3 | -10.28 | -2.3942 | -15.25 | 0 | 14.948 | 6.4776 | -44.85106 | 0.59925 | 1 |
| 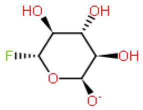 | 0 | 3238 | C5H8FO5 | -25.56 | 19 | 3 | -10.306 | -2.1474 | -8.17 | 0 | 9.7898 | 1.4814 | -71.25748 | 0.52256 | 1 |
| 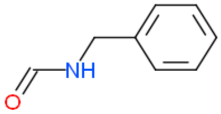 | 0 | 2212 | C8H9NO | -25.54 | 19 | 3 | -8.7325 | -2.8168 | -16.24 | 2.9538 | 9.6508 | 5.8685 | -53.26201 | 0.67238 | 1 |
| 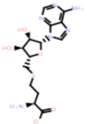 | 0 | 1535 | C14H20N6O5S | -25.5 | 45 | 10 | -14.393 | -4.5421 | -25.58 | 0 | 31.499 | 15.019 | -115.6509 | 1.28949 | 1 |

| | | | | | | | | | | | | | | | |
|---|---|---|---|---|---|---|---|---|---|---|---|---|---|---|---|
| 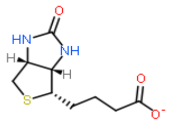 | 0 | 2393 | SNR | -25.49 | 28 | 4 | -11.424 | -3.1892 | -15.27 | 3.257 | 16.613 | 6.0618 | -84.39111 | 1.15054 | 1 |
| 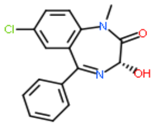 | 0 | 126 | 231 | -25.46 | 34 | 2 | -10.103 | -4.8252 | -19.46 | 3.9496 | 16.603 | 10.262 | -117.6517 | 1.39466 | 1 |
| 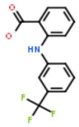 | 0 | 2010 | FLF | -25.45 | 29 | 1 | -8.4635 | -4.4823 | -20.38 | 0 | 11.756 | 15.046 | -114.5109 | 1.26189 | 1 |
| 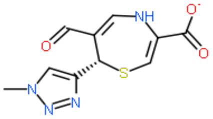 | 0 | 2520 | C10H10N4O3S | -25.44 | 27 | 2 | -9.1653 | -2.3819 | -16.67 | 1.7481 | 17.106 | 1.4229 | -76.15051 | 1.05376 | 1 |
| 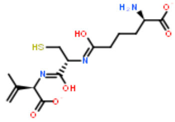 | 0 | 2308 | C14H23N3O6S | -25.41 | 45 | 15 | -15.02 | -4.0267 | -20.43 | 0 | 24.679 | 12.427 | -99.33503 | 1.4137 | 1 |
| 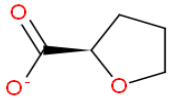 | 0 | 2741 | C5H8O3 | -25.4 | 15 | 1 | -9.0256 | -2.2557 | -9.388 | 0.7481 | 9.6337 | 0.0949 | -57.94007 | 0.41802 | 1 |
| 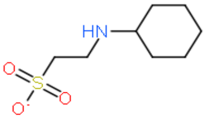 | 0 | 2986 | C8H17NO3S | -25.37 | 29 | 4 | -10.043 | -3.3651 | -16 | 4.2609 | 14.384 | 3.8618 | -57.41175 | 0.94641 | 1 |
| 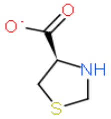 | 0 | 2549 | C4H7NO2S | -25.35 | 14 | 1 | -8.4838 | -2.2972 | -11.42 | 0 | 11.139 | 0.1722 | -46.40346 | 0.51041 | 1 |
| 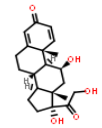 | 0 | 740 | C21H28O5 | -25.31 | 54 | 5 | -13.755 | -4.3749 | -14.75 | 5.6349 | 16.878 | 12.323 | -108.7141 | 1.41198 | 1 |
| 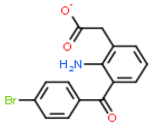 | 0 | 841 | 963 | -25.31 | 31 | 3 | -9.1979 | -4.5538 | -20.94 | 2.6561 | 14.59 | 11.548 | -102.6402 | 1.28148 | 1 |
| 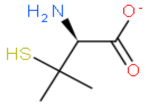 | 0 | 739 | C5H11NO2S | -25.27 | 19 | 4 | -9.2993 | -1.7611 | -12 | 0 | 8.9006 | 3.5802 | -55.19028 | 0.52429 | 1 |

| | | | | | | | | | | | | | | | |
|---|---|---|---|---|---|---|---|---|---|---|---|---|---|---|---|
| 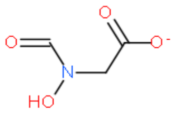 | 0 | 1867 | C3H5NO4 | -25.27 | 12 | 4 | -10.167 | -0.8936 | -10.6 | 0 | 10.05 | 2.5988 | -50.91417 | 0.38986 | 1 |
| 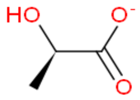 | 0 | 2755 | C3H6O3 | -25.25 | 11 | 2 | -10.823 | -1.288 | -8.526 | 1.8351 | 12.056 | 0.2671 | -41.04602 | 0.32289 | 1 |
| 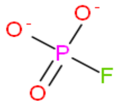 | 0 | 2088 | FO3P | -25.24 | 5 | 0 | -9.0535 | -0.6153 | -6.614 | 0.0787 | 9.3181 | -3.548 | -23.1325 | 0.20905 | 1 |
| 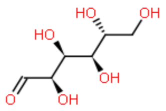 | 0 | 1681 | C6H12O6 | -25.2 | 24 | 10 | -13.342 | -1.7585 | -15.18 | 4.6731 | 12.295 | 10.429 | -70.00839 | 0.79191 | 1 |
| 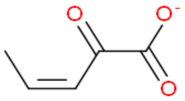 | 0 | 1767 | C5H6O3 | -25.2 | 13 | 1 | -9.1826 | -1.9389 | -12.89 | 0 | 11.428 | 4.6304 | -49.14428 | 0.52437 | 1 |
| 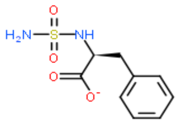 | 0 | 2707 | CXA | -25.19 | 27 | 6 | -8.7803 | -2.8641 | -18.16 | 1.4747 | 12.832 | 4.5395 | -54.80134 | 0.86359 | 1 |
| 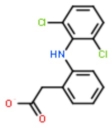 | 0 | 473 | 586 | -25.19 | 29 | 2 | -8.5011 | -4.6943 | -17.08 | 2.8694 | 10.294 | 8.9487 | -87.44639 | 1.16857 | 1 |
| 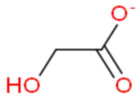 | 0 | 2773 | C2H4O3 | -25.16 | 8 | 2 | -10.651 | -0.8801 | -7.166 | 0 | 11.463 | -0.379 | -37.10253 | 0.26675 | 1 |
| 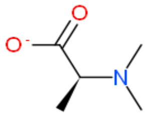 | 0 | 1921 | C5H11NO2 | -25.13 | 18 | 2 | -8.9958 | -2.7275 | -10.72 | 0.75 | 9.9562 | 1.6146 | -44.64582 | 0.49711 | 1 |
| 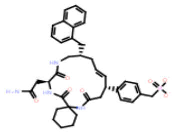 | 0 | 2954 | C37H45N4O7P | -25.09 | 92 | 7 | -6.786 | -7.6505 | -33.08 | 5.7464 | 27.672 | 2.8453 | -152.4948 | 2.69655 | 1 |
| 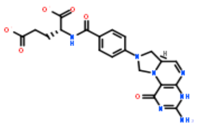 | 0 | 2043 | C20H21N7O6 | -25.08 | 52 | 5 | -10.645 | -4.2319 | -22.21 | 1.2961 | 27.21 | 4.117 | -116.7386 | 1.76256 | 1 |

| | | | | | | | | | | | | | | | |
|---|---|---|---|---|---|---|---|---|---|---|---|---|---|---|---|
| 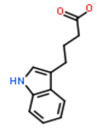 | 0 | 2452 | 3IB | -25.05 | 27 | 4 | -9.2721 | -3.7391 | -16.54 | 2.283 | 13.23 | 5.7087 | -91.16441 | 0.97952 | 1 |
| 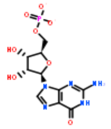 | 0 | 1736 | C10H14N5O8P | -25.04 | 36 | 6 | -13.001 | -2.6873 | -20.44 | 1.952 | 25.012 | 9.9411 | -85.78504 | 1.24402 | 1 |
| 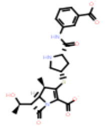 | 0 | 197 | C22H25N3O7S | -25.03 | 56 | 6 | -17.495 | -5.2649 | -24.14 | 8.2352 | 32.327 | 21.38 | -159.6733 | 1.80878 | 1 |
| 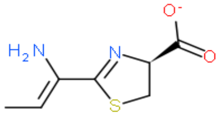 | 0 | 2637 | TZB | -25.02 | 21 | 1 | -8.4938 | -2.6853 | -13.45 | 0.1763 | 14.191 | 0.5621 | -58.45703 | 0.59333 | 1 |
| 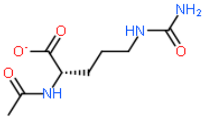 | 0 | 2107 | C8H15N3O4 | -25.01 | 29 | 7 | -12.028 | -2.8167 | -16.32 | 4.595 | 18.182 | 4.598 | -88.61235 | 1.18562 | 1 |
| 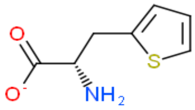 | 0 | 3320 | C7H9NO2S | -25 | 19 | 4 | -9.6947 | -2.6645 | -13.15 | 0 | 14.092 | 2.2718 | -55.62628 | 0.69683 | 1 |
| 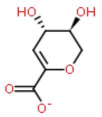 | 0 | 2961 | C6H8O5 | -24.99 | 18 | 3 | -11.626 | -1.8587 | -12.11 | 0 | 16.38 | 5.6003 | -63.56576 | 0.58219 | 1 |
| 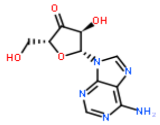 | 0 | 2951 | C10H11N5O4 | -24.98 | 30 | 4 | -14.477 | -3.7546 | -17.22 | 4.3993 | 24.71 | 11.834 | -83.63154 | 1.15498 | 1 |
| 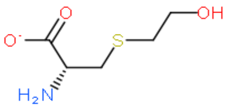 | 0 | 2149 | C5H11NO3S | -24.97 | 20 | 7 | -13.397 | -2.2193 | -10.78 | 3.8416 | 17.963 | 1.8517 | -58.21028 | 0.66462 | 1 |
| 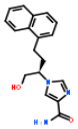 | 0 | 3226 | FR3 | -24.97 | 42 | 6 | -14.176 | -4.0771 | -20.29 | 9.7972 | 20.134 | 14.15 | -78.97423 | 1.25465 | 1 |
| 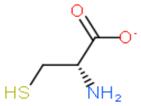 | 0 | 2882 | C3H7NO2S | -24.96 | 13 | 4 | -12.534 | -1.0099 | -8.721 | 0 | 12.512 | 6.619 | -29.95774 | 0.47303 | 1 |

| | | | | | | | | | | | | | | | |
|---|---|---|---|---|---|---|---|---|---|---|---|---|---|---|---|
| 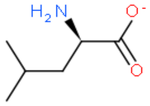 | 0 | 1529 | C6H13NO2 | -24.95 | 21 | 4 | -11.09 | -2.411 | -11.72 | 0 | 15.104 | 4.115 | -49.82432 | 0.66234 | 1 |
| 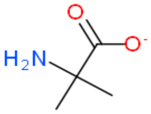 | 0 | 2648 | C4H9NO2 | -24.91 | 15 | 2 | -10.665 | -1.8167 | -9.761 | 0 | 13.342 | 2.7963 | -45.407 | 0.42773 | 1 |
| 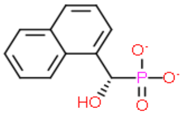 | 0 | 2898 | I59 | -24.89 | 25 | 3 | -10.097 | -3.0035 | -15.96 | 0.2646 | 12.712 | 10.809 | -79.57917 | 0.84105 | 1 |
| 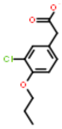 | 0 | 2481 | 34C | -24.84 | 27 | 4 | -9.1395 | -4.141 | -14.55 | 3.0969 | 12.865 | 2.5733 | -72.94099 | 1.20131 | 1 |
| 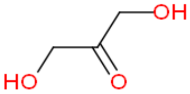 | 0 | 1555 | C3H6O3 | -24.84 | 12 | 4 | -10.854 | -1.0302 | -9.107 | 2.1934 | 9.4199 | 2.4817 | -5.062957 | 0.31835 | 1 |
| 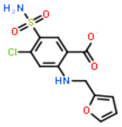 | 0 | 581 | 695 | -24.78 | 31 | 5 | -9.4012 | -3.9968 | -24.6 | 0 | 14.324 | 19.004 | -115.9323 | 1.57863 | 1 |
| 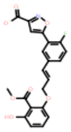 | 0 | 1775 | 234 | -24.78 | 45 | 3 | -10.641 | -4.7335 | -26.44 | 3.1374 | 22.779 | 16.652 | -132.5013 | 1.8277 | 1 |
| 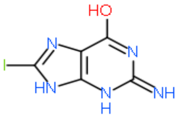 | 0 | 2681 | C5H4IN5O | -24.77 | 16 | 1 | -11.681 | -2.0048 | -18.99 | 1.2922 | 19.321 | 14.006 | -48.69702 | 0.78778 | 1 |
| 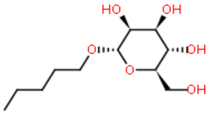 | 0 | 2411 | C11H22O6 | -24.77 | 39 | 10 | -17.557 | -3.2475 | -9.026 | 7.022 | 20.874 | 7.5415 | -76.73463 | 1.23409 | 1 |
| 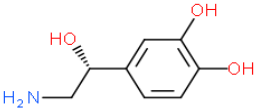 | 0 | 261 | 368 | -24.75 | 23 | 4 | -12.689 | -2.3071 | -16.98 | 4.0824 | 16.629 | 12.962 | -43.92217 | 0.73079 | 1 |
| 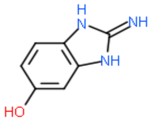 | 0 | 3370 | C7H7N3O | -24.73 | 18 | 0 | -8.7832 | -1.2795 | -16.19 | 0.4581 | 13.063 | 6.7622 | -18.76523 | 0.57073 | 1 |

| | | | | | | | | | | | | | | | |
|---|---|---|---|---|---|---|---|---|---|---|---|---|---|---|---|
| 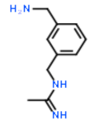 | 0 | 1804 | 14W | -24.72 | 28 | 4 | -10.888 | -4.062 | -15.02 | 2.6089 | 15.503 | 7.5435 | -48.62602 | 0.88367 | 1 |
| 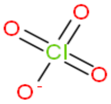 | 0 | 2821 | ClO4 | -24.71 | 5 | 0 | -8.8801 | -0.316 | -10.23 | 0.0381 | 10.16 | 0.8368 | -18.9737 | 0.2136 | 1 |
| 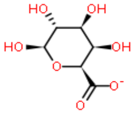 | 0 | 3301 | C6H10O7 | -24.69 | 22 | 5 | -10.853 | -1.8467 | -11.76 | 0 | 14.493 | 2.8651 | -66.81895 | 0.78533 | 1 |
| 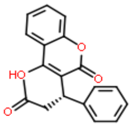 | 0 | 2237 | C19H16O4 | -24.69 | 39 | 4 | -10.304 | -4.821 | -21.17 | 5.6075 | 17.447 | 10.495 | -135.7898 | 1.47321 | 1 |
| 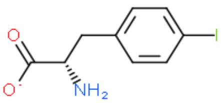 | 0 | 3308 | PHI | -24.69 | 22 | 4 | -8.3806 | -3.3865 | -15.77 | 0 | 14.152 | 2.3284 | -55.14016 | 0.99859 | 1 |
| 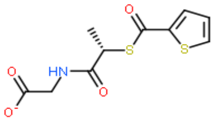 | 0 | 1230 | 1423 | -24.69 | 27 | 4 | -8.539 | -3.8404 | -17.54 | 0 | 13.578 | 6.7351 | -93.72022 | 1.17649 | 1 |
| 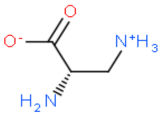 | 0 | 2997 | C3H9N2O2 | -24.69 | 15 | 3 | -10.184 | -1.2672 | -11.18 | 0 | 9.9153 | 5.8721 | -32.49344 | 0.42739 | 1 |
| 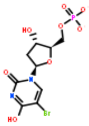 | 0 | 1673 | C9H12BrN2O8P | -24.68 | 31 | 6 | -13.683 | -2.7352 | -24.82 | 3.0455 | 25.967 | 17.711 | -77.82717 | 1.24426 | 1 |
| 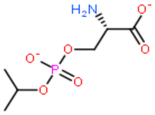 | 0 | 1582 | C6H14NO6P | -24.68 | 26 | 7 | -9.5419 | -2.0764 | -12.01 | 4.006 | 14.543 | -7.04 | -39.41575 | 0.96565 | 1 |
| 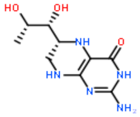 | 0 | 253 | m | -24.68 | 32 | 4 | -11.523 | -2.8089 | -20.05 | 0.188 | 18.078 | 15.668 | -90.04771 | 1.23789 | 1 |
| 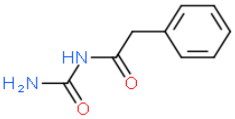 | 0 | 992 | 1121 | -24.67 | 23 | 3 | -9.6403 | -2.9845 | -15.95 | 0 | 15.214 | 6.7541 | -56.80708 | 0.83591 | 1 |

| | | | | | | | | | | | | | | | |
|---|---|---|---|---|---|---|---|---|---|---|---|---|---|---|---|
| 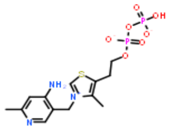 | 0 | 2519 | C13H19N3O7P2S | -24.66 | 44 | 9 | -15.051 | -5.0728 | -22.28 | 3.8528 | 22.952 | 21.474 | -118.73 | 1.36446 | 1 |
| 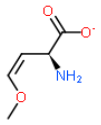 | 0 | 3262 | C5H9NO3 | -24.65 | 17 | 3 | -13.086 | -2.1199 | -10.33 | 3.595 | 15.73 | 6.742 | -50.78635 | 0.59031 | 1 |
| 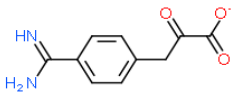 | 0 | 1779 | C10H10N2O3 | -24.62 | 24 | 3 | -9.2794 | -2.5065 | -17.86 | 0 | 14.934 | 8.1796 | -70.53074 | 0.88739 | 1 |
| 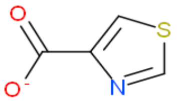 | 0 | 3090 | C4H3NO2S | -24.61 | 10 | 1 | -8.5834 | -2.1091 | -10.9 | 0 | 10.538 | 1.4006 | -41.20578 | 0.47002 | 1 |
| 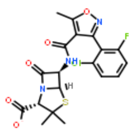 | 0 | 195 | 301 | -24.58 | 46 | 2 | -9.1766 | -4.85 | -24.64 | 8.572 | 18.333 | 9.7486 | -147.7619 | 1.46893 | 1 |
| 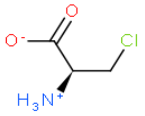 | 0 | 1519 | C3H6ClNO2 | -24.58 | 13 | 2 | -9.9273 | -1.7615 | -10.35 | 0 | 9.5751 | 5.5746 | -38.83546 | 0.41406 | 1 |
| 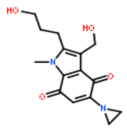 | 0 | 2131 | C15H18N2O4 | -24.57 | 39 | 6 | -11.136 | -4.8185 | -23.08 | 3.0789 | 19.72 | 14.667 | -115.7593 | 1.61371 | 1 |
| 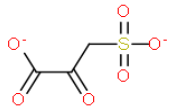 | 0 | 1911 | C3H4O6S | -24.55 | 12 | 3 | -10.674 | -1.1151 | -11.72 | 0.4032 | 15.004 | 2.4545 | -45.96428 | 0.58853 | 1 |
| 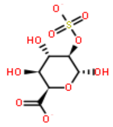 | 0 | 2008 | C6H10O10S | -24.55 | 25 | 6 | -12.515 | -1.5707 | -14.39 | 0 | 19.78 | 6.2468 | -71.28324 | 0.88852 | 1 |
| 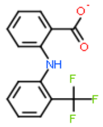 | 0 | 1932 | OFL | -24.55 | 29 | 1 | -7.6916 | -4.1551 | -17.72 | 3.2943 | 12.222 | 5.5708 | -93.91958 | 1.38471 | 1 |
| 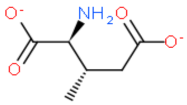 | 0 | 1669 | C6H11NO4 | -24.54 | 20 | 5 | -13.187 | -1.7292 | -9.887 | 2.7745 | 16.467 | 4.3253 | -56.16486 | 0.65036 | 1 |

| | | | | | | | | | | | | | | | |
|---|---|---|---|---|---|---|---|---|---|---|---|---|---|---|---|
| 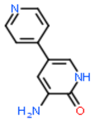 | 0 | 1234 | 1427 | -24.52 | 23 | 0 | -7.4904 | -3.4238 | -19.47 | 0.5785 | 13.108 | 9.1185 | -64.14204 | 0.8671 | 1 |
| 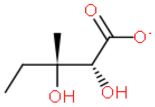 | 0 | 3321 | C6H12O4 | -24.51 | 21 | 5 | -11.327 | -2.3555 | -10.33 | 2.0362 | 11.919 | 4.3908 | -71.40373 | 0.58364 | 1 |
| 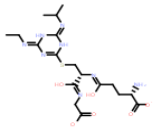 | 0 | 1903 | C18H30N8O6S | -24.49 | 61 | 15 | -15.445 | -5.111 | -26.09 | 4.7212 | 22.387 | 23.446 | -139.1007 | 2.07333 | 1 |
| 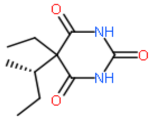 | 0 | 132 | 237 | -24.46 | 31 | 3 | -11.395 | -3.2249 | -17.49 | 2.2178 | 15.586 | 13.94 | -92.29673 | 0.92237 | 1 |
| 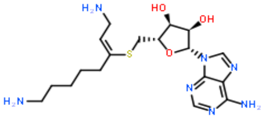 | 0 | 2547 | C18H29N7O3S | -24.42 | 58 | 13 | -18.396 | -5.4125 | -32.36 | 12.478 | 35.961 | 24.017 | -131.5594 | 1.95223 | 1 |
| 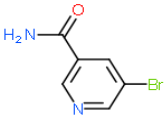 | 0 | 1629 | C6H5BrN2O | -24.41 | 15 | 0 | -9.833 | -2.7973 | -12.2 | 0.4691 | 12.592 | 7.296 | -32.99927 | 0.76227 | 1 |
| 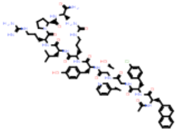 | 0 | 5 | [NO | -24.39 | 194 | 42 | -17.06 | -11.871 | -75.58 | 30.301 | 46.427 | 36.513 | -306.123 | 5.05128 | 1 |
| 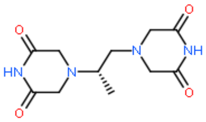 | 0 | 272 | 380 | -24.39 | 35 | 3 | -12.177 | -2.9278 | -19.36 | 3.0106 | 19.732 | 14.149 | -108.4008 | 1.27914 | 1 |
| 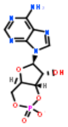 | 0 | 2257 | C10H12N5O6P | -24.38 | 33 | 2 | -11.099 | -3.4818 | -18.5 | 0.453 | 21.665 | 10.332 | -90.92592 | 1.13913 | 1 |
| 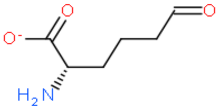 | 0 | 2298 | C6H11NO3 | -24.37 | 20 | 6 | -10.627 | -2.0681 | -11.49 | 0 | 15.643 | 0.088 | -68.72284 | 0.81986 | 1 |
| 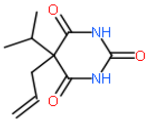 | 0 | 1180 | C10H14N2O3 | -24.36 | 29 | 3 | -11.506 | -3.1973 | -19.69 | 3.8475 | 15.505 | 16.432 | -90.51614 | 0.91086 | 1 |

| | | | | | | | | | | | | | | | |
|---|---|---|---|---|---|---|---|---|---|---|---|---|---|---|---|
| 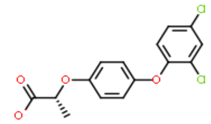 | 0 | 3419 | D1L | -24.36 | 32 | 4 | -8.8363 | -5.7346 | -21.49 | 1.4328 | 17.635 | 10.373 | -125.3101 | 1.38942 | 1 |
| 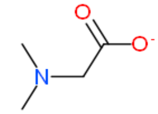 | 0 | 1841 | C4H9NO2 | -24.23 | 15 | 2 | -9.2544 | -2.3134 | -10.11 | 0.9932 | 9.8338 | 2.5258 | -39.68833 | 0.49854 | 1 |
| 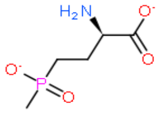 | 0 | 2383 | C5H12NO4P | -24.23 | 21 | 5 | -12.257 | -2.1587 | -15.06 | 0 | 17.853 | 10.447 | -53.44928 | 0.75954 | 1 |
| 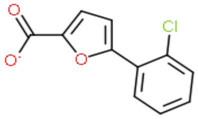 | 0 | 2609 | FCD | -24.22 | 21 | 1 | -8.4535 | -3.559 | -15.43 | 1.0829 | 12.117 | 7.0319 | -72.79428 | 0.93106 | 1 |
| 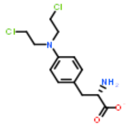 | 0 | 916 | C13H18Cl2N2O2 | -24.22 | 36 | 8 | -10.365 | -4.7913 | -17.51 | 0 | 17.246 | 7.568 | -79.2291 | 1.10519 | 1 |
| 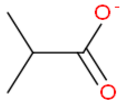 | 0 | 2261 | C4H8O2 | -24.19 | 13 | 1 | -8.7086 | -2.0854 | -9.153 | 0.1415 | 8.953 | 1.507 | -41.91448 | 0.35637 | 1 |
| 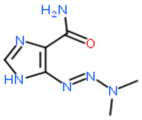 | 0 | 731 | C6H10N6O | -24.16 | 23 | 0 | -10.568 | -3.1995 | -15.65 | 3.8695 | 17.237 | 7.7591 | -38.73374 | 0.85038 | 1 |
| 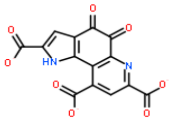 | 0 | 2886 | C14H6N2O8 | -24.15 | 27 | 3 | -10.972 | -2.3153 | -21.43 | 0 | 19.227 | 15.711 | -128.8018 | 1.5439 | 1 |
| 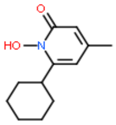 | 0 | 1058 | 1188 | -24.14 | 32 | 2 | -7.5552 | -4.1363 | -19.24 | 0.2257 | 13.463 | 8.4079 | -80.78357 | 1.22396 | 1 |
| 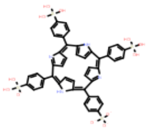 | 0 | 1763 | C44H34N4O12S4 | -24.13 | 98 | 12 | -16.841 | -6.7688 | -44.28 | 6.9206 | 49.788 | 28.175 | -177.4724 | 3.53939 | 1 |
| 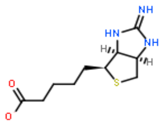 | 0 | 3028 | C10H17N3O2S | -24.09 | 32 | 5 | -12.718 | -3.2958 | -16.57 | 1.3935 | 19.218 | 13.05 | -84.48431 | 1.18531 | 1 |

| | | | | | | | | | | | | | | | |
|---|---|---|---|---|---|---|---|---|---|---|---|---|---|---|---|
| 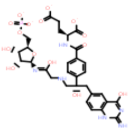 | 0 | 2499 | C30H37N6O15P | -24.09 | 85 | 21 | -19.616 | -5.4549 | -29.83 | 0 | 36.893 | 28.417 | -171.5067 | 2.66031 | 1 |
| 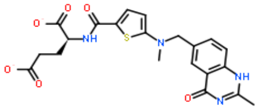 | 0 | 187 | 293 | -24.05 | 52 | 7 | -14.678 | -5.7764 | -29.86 | 9.1812 | 23.118 | 29.954 | -155.5874 | 1.58309 | 1 |
| 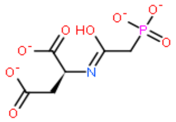 | 0 | 3125 | C6H10NO8P | -24 | 22 | 7 | -13.092 | -1.8406 | -14.1 | 0 | 18.376 | 9.8004 | -79.4211 | 0.91754 | 1 |
| 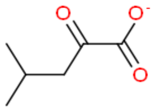 | 0 | 2910 | C6H10O3 | -23.99 | 18 | 3 | -9.3801 | -2.4185 | -12.59 | 0 | 12.315 | 4.4162 | -56.98485 | 0.66161 | 1 |
| 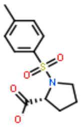 | 0 | 2463 | TPR | -23.99 | 32 | 3 | -9.0718 | -4.0544 | -19.11 | 3.0343 | 12.02 | 12.346 | -96.05077 | 1.14027 | 1 |
| 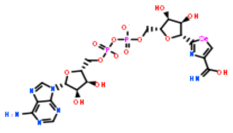 | 0 | 2759 | C19H25N7O14P2Se | -23.98 | 66 | 15 | -19.609 | -4.2712 | -34.51 | 9.6144 | 36.157 | 31.992 | -185.0186 | 2.24507 | 1 |
| 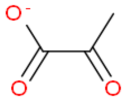 | 0 | 16 | C3H4O3 | -23.97 | 9 | 1 | -9.1837 | -1.2026 | -8.92 | 0 | 10.422 | 0.8733 | -37.01013 | 0.33491 | 1 |
| 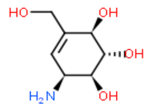 | 0 | 1877 | C7H13NO4 | -23.97 | 25 | 6 | -12.337 | -2.0574 | -14.91 | 1.6338 | 16.698 | 9.7425 | -60.31506 | 0.79052 | 1 |
| 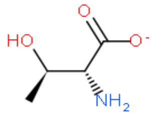 | 0 | 3343 | C4H9NO3 | -23.97 | 16 | 4 | -11.105 | -1.653 | -10.72 | 1.1507 | 12.014 | 5.8408 | -58.31144 | 0.53278 | 1 |
| 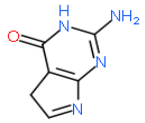 | 0 | 1991 | C6H6N4O | -23.96 | 17 | 0 | -9.4579 | -1.6676 | -14.09 | 0 | 13.924 | 7.1718 | -60.6938 | 0.75155 | 1 |
| 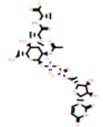 | 0 | 1462 | m | -23.95 | 82 | 19 | -18.341 | -4.5259 | -34.57 | 6.9751 | 38.298 | 23.856 | -123.9397 | 2.5794 | 1 |

| | | | | | | | | | | | | | | | |
|---|---|---|---|---|---|---|---|---|---|---|---|---|---|---|---|
| 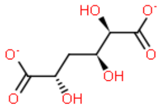 | 0 | 2893 | C6H8O7 | -23.94 | 21 | 8 | -10.888 | -1.536 | -11.64 | 0 | 12.398 | 3.3068 | -70.04833 | 0.7927 | 1 |
| 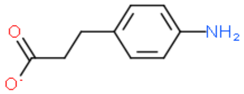 | 0 | 2891 | AHC | -23.93 | 22 | 3 | -8.5971 | -3.1199 | -16.02 | 0.1707 | 12.757 | 6.7633 | -57.88824 | 0.74006 | 1 |
| 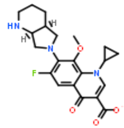 | 0 | 113 | 218 | -23.92 | 52 | 2 | -7.6659 | -5.3484 | -21.9 | 1.7059 | 16.502 | 9.6244 | -128.4572 | 1.63208 | 1 |
| 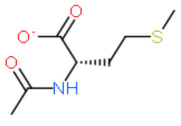 | 0 | 1438 | C7H13NO3S | -23.92 | 24 | 5 | -9.6366 | -3.3796 | -17.02 | 2.3343 | 13.411 | 8.0079 | -70.49933 | 0.88818 | 1 |
| 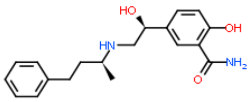 | 0 | 485 | 598 | -23.91 | 48 | 8 | -12.964 | -4.3464 | -21.37 | 9.3132 | 21.007 | 10.649 | -92.02509 | 1.36348 | 1 |
| 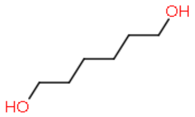 | 0 | 1959 | C6H14O2 | -23.91 | 22 | 7 | -11.59 | -2.9643 | -12.74 | 4.0021 | 13.85 | 4.7687 | -51.46894 | 0.71623 | 1 |
| 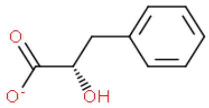 | 0 | 2224 | HFA | -23.91 | 21 | 4 | -8.3347 | -2.968 | -16.1 | 0 | 12.35 | 5.4659 | -64.78383 | 0.75794 | 1 |
| 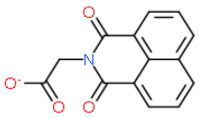 | 0 | 1781 | ALR | -23.9 | 27 | 2 | -8.5816 | -2.976 | -17.84 | 3.9244 | 14.338 | 5.0674 | -99.76279 | 0.94488 | 1 |
| 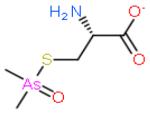 | 0 | 3322 | C5H12AsNO3S | -23.89 | 22 | 5 | -10.221 | -2.5403 | -15.65 | 0 | 17.131 | 5.2588 | -47.63272 | 0.87273 | 1 |
| 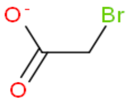 | 0 | 1950 | C2H3BrO2 | -23.89 | 7 | 1 | -8.5939 | -1.8216 | -8.035 | 0.3886 | 8.6094 | -0.278 | -29.08055 | 0.35829 | 1 |
| 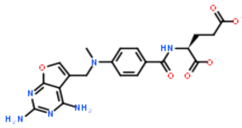 | 0 | 1787 | C20H22N6O6 | -23.88 | 52 | 7 | -13.563 | -5.4915 | -25.08 | 8.8899 | 31.482 | 9.1673 | -126.9359 | 2.06812 | 1 |

| | | | | | | | | | | | | | | | |
|---|---|---|---|---|---|---|---|---|---|---|---|---|---|---|---|
| 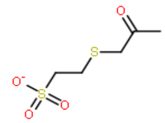 | 0 | 2845 | C5H10O4S2 | -23.86 | 20 | 5 | -9.3593 | -2.8039 | -13.89 | 0 | 13.565 | 3.6475 | -52.41246 | 0.94649 | 1 |
| 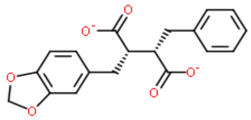 | 0 | 2183 | BYS | -23.86 | 41 | 7 | -13.058 | -4.0281 | -23.26 | 1.6104 | 17.304 | 25.741 | -157.996 | 1.40759 | 1 |
| 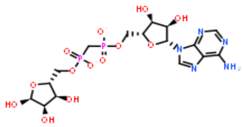 | 0 | 1739 | C16H25N5O13P2 | -23.84 | 59 | 14 | -16.67 | -4.4858 | -32.76 | 4.8915 | 36.596 | 23.207 | -160.648 | 2.02922 | 1 |
| 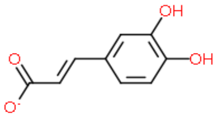 | 0 | 1653 | DHC | -23.83 | 20 | 1 | -8.805 | -2.5209 | -14.39 | 1.4363 | 13.155 | 4.8527 | -57.42283 | 0.90198 | 1 |
| 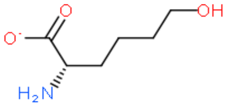 | 0 | 3080 | C6H13NO3 | -23.81 | 22 | 7 | -12.992 | -2.1757 | -9.631 | 1.6003 | 14.943 | 5.7321 | -54.71513 | 0.81606 | 1 |
| 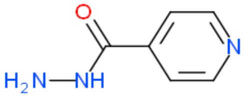 | 0 | 829 | 951 | -23.8 | 17 | 1 | -8.881 | -2.3094 | -14.77 | 0.3855 | 14.343 | 5.1512 | -57.29471 | 0.74262 | 1 |
| 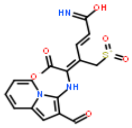 | 0 | 2577 | C16H14N3O6S | -23.8 | 39 | 4 | -14.946 | -3.6406 | -24.35 | 10.841 | 21.028 | 24.443 | -85.50879 | 1.6726 | 1 |
| 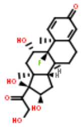 | 0 | 507 | 620 | -23.79 | 55 | 6 | -10.367 | -3.654 | -20.68 | 1.5885 | 19.354 | 9.8524 | -89.67769 | 1.29585 | 1 |
| 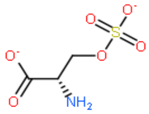 | 0 | 3160 | C3H7NO6S | -23.78 | 16 | 5 | -11.089 | -1.4132 | -13.4 | 0 | 18.299 | 2.8805 | -52.46943 | 0.71014 | 1 |
| 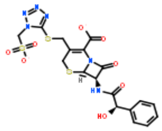 | 0 | 1162 | C18H18N6O8S3 | -23.77 | 51 | 9 | -14.681 | -3.5412 | -29.14 | 9.5864 | 29.469 | 17.614 | -110.9446 | 2.00784 | 1 |
| 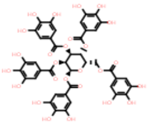 | 0 | 2889 | C41H32O26 | -23.71 | 99 | 6 | -11.765 | -4.9684 | -46.67 | 12.602 | 35.975 | 27.132 | -149.3931 | 2.99251 | 1 |

| | | | | | | | | | | | | | | | |
|---|---|---|---|---|---|---|---|---|---|---|---|---|---|---|---|
| 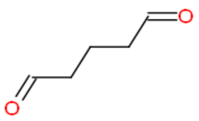 | 0 | 2945 | C5H8O2 | -23.68 | 15 | 4 | -9.9845 | -1.5018 | -12.12 | 3.1541 | 8.8353 | 5.7488 | -41.3747 | 0.39086 | 1 |
| 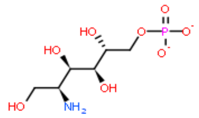 | 0 | 2178 | C6H16NO8P | -23.68 | 30 | 12 | -14.854 | -2.1093 | -14.34 | 0 | 18.209 | 13.338 | -94.95658 | 1.01258 | 1 |
| 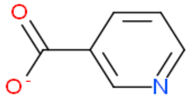 | 0 | 514 | 627 | -23.66 | 13 | 1 | -9.9684 | -2.2647 | -12.33 | 0.7582 | 11.493 | 8.6828 | -41.04776 | 0.47056 | 1 |
| 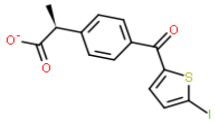 | 0 | 3392 | ISF | -23.65 | 29 | 2 | -9.5658 | -5.0743 | -20.7 | 3.665 | 18.066 | 11.674 | -120.3378 | 1.29177 | 1 |
| 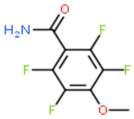 | 0 | 1604 | BEK | -23.64 | 20 | 0 | -9.0026 | -3.0604 | -13.18 | 0 | 12.208 | 7.8933 | -68.39256 | 0.79013 | 1 |
| 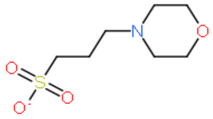 | 0 | 3101 | C7H15NO4S | -23.62 | 27 | 4 | -8.9612 | -3.3092 | -14.12 | 1.3032 | 12.386 | 4.4141 | -72.23865 | 0.72594 | 1 |
| 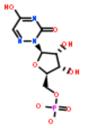 | 0 | 3359 | C8H12N3O9P | -23.61 | 31 | 7 | -15.884 | -2.1398 | -16.9 | 0 | 22.572 | 20.873 | -105.1081 | 1.27257 | 1 |
| 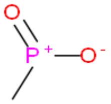 | 0 | 2529 | CH3O2P | -23.57 | 7 | 0 | -9.1146 | -1.1866 | -4.963 | 0.0839 | 8.5276 | -1.821 | -16.66473 | 0.25945 | 1 |
| 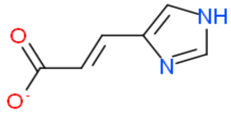 | 0 | 1735 | C6H6N2O2 | -23.55 | 15 | 1 | -9.643 | -2.4546 | -14.26 | 0.7118 | 12.135 | 10.04 | -49.22999 | 0.65497 | 1 |
| 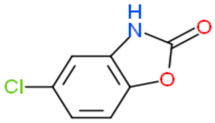 | 0 | 249 | 2733 | -23.55 | 15 | 0 | -9.5571 | -2.7562 | -14.75 | 0.5476 | 11.716 | 12.289 | -49.1696 | 0.72709 | 1 |
| 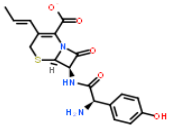 | 0 | 1021 | C18H19N3O5S | -23.55 | 45 | 5 | -11.276 | -4.9474 | -27.51 | 1.1861 | 24.115 | 21.23 | -131.6079 | 1.73329 | 1 |

| | | | | | | | | | | | | | | | |
|---|---|---|---|---|---|---|---|---|---|---|---|---|---|---|---|
| 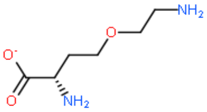 | 0 | 2667 | C6H14N2O3 | -23.53 | 24 | 8 | -13.003 | -2.284 | -14.78 | 3.9073 | 17.717 | 7.9597 | -71.77908 | 0.86616 | 1 |
| 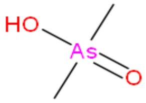 | 0 | 2690 | C2H7AsO2 | -23.51 | 12 | 1 | -9.6849 | -1.9918 | -9.404 | 0 | 10.587 | 4.8361 | -21.44208 | 0.41414 | 1 |
| 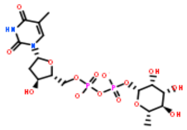 | 0 | 3364 | m | -23.5 | 59 | 12 | -18.329 | -3.547 | -31.62 | 8.7171 | 30.738 | 32.412 | -154.3183 | 1.84395 | 1 |
| 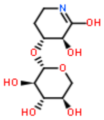 | 0 | 3358 | C10H17NO7 | -23.43 | 35 | 7 | -11.745 | -3.0517 | -17.22 | 2.2386 | 19.846 | 7.7705 | -107.6634 | 1.16109 | 1 |
| 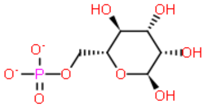 | 0 | 2600 | C6H13O9P | -23.39 | 27 | 7 | -15.13 | -1.807 | -10.39 | 0 | 20.211 | 10.796 | -80.89259 | 0.86302 | 1 |
| 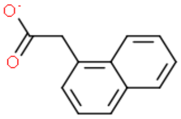 | 0 | 1533 | NLA | -23.39 | 23 | 2 | -7.3753 | -3.4198 | -15.17 | 1.0756 | 10.626 | 4.4191 | -78.06341 | 0.77862 | 1 |
| 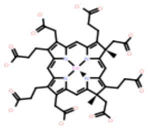 | 0 | 2535 | C42H42FeN4O16 | -23.39 | 97 | 20 | -18.229 | -6.809 | -26.89 | 0 | 36.251 | 22.716 | -133.2399 | 2.25902 | 1 |
| 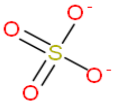 | 0 | 540 | MgO4S | -23.38 | 5 | 0 | -8.7451 | -0.3241 | -7.115 | 0.0405 | 10.372 | -2.616 | -20.97643 | 0.22269 | 1 |
| 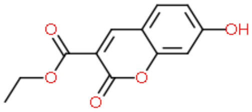 | 0 | 2442 | YZ9 | -23.38 | 27 | 1 | -8.9503 | -3.124 | -18.1 | 1.2178 | 17.523 | 7.5596 | -75.46439 | 1.36171 | 1 |
| 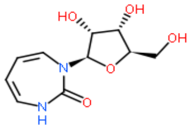 | 0 | 2867 | C10H14N2O5 | -23.37 | 31 | 5 | -10.493 | -2.7137 | -15.82 | 2.5359 | 16.841 | 5.5516 | -92.48728 | 1.11111 | 1 |
| 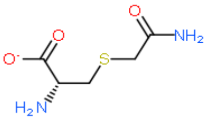 | 0 | 2683 | C5H10N2O3S | -23.37 | 20 | 6 | -11.891 | -1.8209 | -16.34 | 3.5846 | 18.294 | 7.2739 | -50.0367 | 0.76859 | 1 |

| | | | | | | | | | | | | | | | |
|---|---|---|---|---|---|---|---|---|---|---|---|---|---|---|---|
| 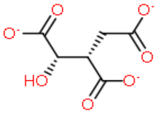 | 0 | 1512 | C6H8O7 | -23.37 | 18 | 6 | -14.457 | -1.1776 | -14.91 | 1.5639 | 15.9 | 18.846 | -89.87734 | 0.68878 | 1 |
| 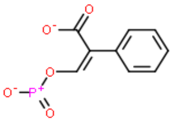 | 0 | 2244 | C9H6O5P | -23.37 | 21 | 3 | -10.134 | -2.6821 | -18.43 | 0.9739 | 15.977 | 12.322 | -93.31986 | 1.12218 | 1 |
| 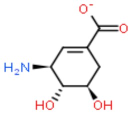 | 0 | 3386 | C7H10NO4 | -23.36 | 22 | 4 | -10.695 | -1.985 | -13.57 | 0 | 15.682 | 6.7322 | -57.70565 | 0.63437 | 1 |
| 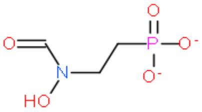 | 0 | 2818 | C3H8NO5P | -23.36 | 16 | 5 | -14.13 | -1.3661 | -10.51 | 3.0474 | 14.649 | 12.119 | -53.94172 | 0.53082 | 1 |
| 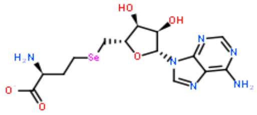 | 0 | 3091 | C14H20N6O5Se | -23.34 | 45 | 10 | -16.413 | -4.4159 | -24.9 | 2.4912 | 29.103 | 25.33 | -130.7009 | 1.73941 | 1 |
| 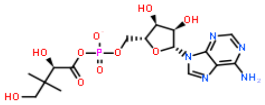 | 0 | 2410 | C16H24N5O10P | -23.34 | 55 | 12 | -14.316 | -5.0125 | -26.84 | 5.4944 | 30.704 | 14.473 | -146.4609 | 1.87369 | 1 |
| 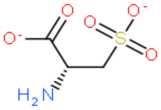 | 0 | 3309 | C3H7NO5S | -23.33 | 15 | 4 | -10.615 | -1.3206 | -11.23 | 0 | 15.054 | 2.9595 | -46.32957 | 0.60863 | 1 |
| 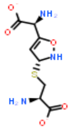 | 0 | 1738 | C8H13N3O5S | -23.31 | 28 | 8 | -15.323 | -2.2011 | -16.64 | 1.9705 | 24.299 | 14.194 | -67.56754 | 1.19077 | 1 |
| 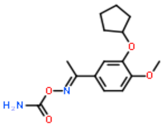 | 0 | 2380 | FIL | -23.27 | 41 | 1 | -8.1721 | -5.1453 | -24.54 | 5.0587 | 16.733 | 13.955 | -110.5909 | 1.34794 | 1 |
| 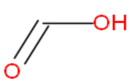 | 0 | 1707 | CH2O2 | -23.24 | 5 | 1 | -9.8258 | -0.1582 | -6.398 | 0.0349 | 9.0378 | 1.0996 | -10.06594 | 0.1241 | 1 |
| 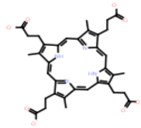 | 0 | 3368 | Coproporphyrin | -23.23 | 82 | 12 | -13.47 | -6.8738 | -30.01 | 3.0804 | 28.193 | 23.126 | -181.2764 | 2.33061 | 1 |

| | | | | | | | | | | | | | | | |
|---|---|---|---|---|---|---|---|---|---|---|---|---|---|---|---|
| 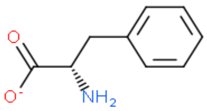 | 0 | 17 | 120 | -23.22 | 22 | 4 | -8.5349 | -2.9494 | -16.21 | 0.1755 | 13.872 | 5.5253 | -58.22055 | 0.7892 | 1 |
| 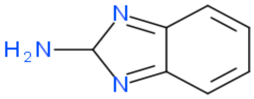 | 0 | 1454 | C7H7N3 | -23.22 | 17 | 1 | -9.097 | -2.5199 | -11.8 | 0 | 13.886 | 3.4507 | -47.76376 | 0.59117 | 1 |
| 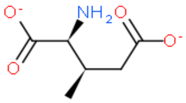 | 0 | 3445 | C6H11NO4 | -23.21 | 20 | 5 | -13.175 | -1.7008 | -9.02 | 3.1827 | 16.054 | 5.0484 | -67.37636 | 0.49718 | 1 |
| 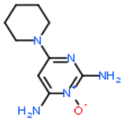 | 0 | 243 | m | -23.19 | 30 | 0 | -9.0474 | -3.9951 | -16.38 | 2.8413 | 16.084 | 7.5524 | -60.4021 | 0.88351 | 1 |
| 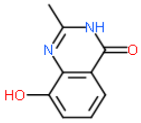 | 0 | 2407 | NU1 | -23.17 | 21 | 0 | -8.7891 | -3.0259 | -15.06 | 0.0075 | 12.46 | 10.305 | -86.10068 | 1.05289 | 1 |
| 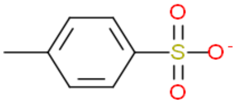 | 0 | 2806 | C7H8O3S | -23.16 | 18 | 1 | -9.3954 | -2.8134 | -14.39 | 0.5039 | 11.825 | 10.772 | -46.62114 | 0.74164 | 1 |
| 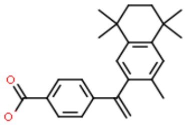 | 0 | 201 | 307 | -23.16 | 53 | 2 | -7.2397 | -6.0799 | -21.37 | 2.9952 | 15.223 | 9.3798 | -129.5669 | 1.53388 | 1 |
| 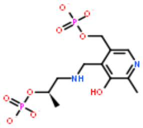 | 0 | 3100 | C11H20N2O9P2 | -23.13 | 40 | 9 | -10.67 | -3.9618 | -18.85 | 0 | 21.213 | 6.1483 | -106.4425 | 1.51419 | 1 |
| 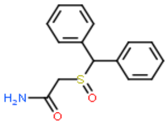 | 0 | 628 | 745 | -23.13 | 34 | 5 | -12.093 | -4.1139 | -14.92 | 4.5917 | 15.181 | 12.117 | -85.1051 | 1.19402 | 1 |
| 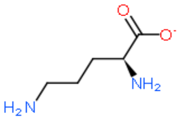 | 0 | 25 | C5H12N2O2 | -23.11 | 20 | 6 | -10.76 | -1.776 | -12.52 | 0.5668 | 16.704 | 2.0618 | -56.60269 | 0.61924 | 1 |
| 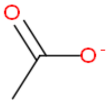 | 0 | 2848 | Acetic | -23.1 | 7 | 0 | -8.5651 | -1.2008 | -6.404 | 0.0446 | 8.4669 | -0.906 | -26.05805 | 0.24289 | 1 |

| | | | | | | | | | | | | | | | |
|---|---|---|---|---|---|---|---|---|---|---|---|---|---|---|---|
| 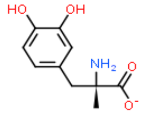 | 0 | 845 | C10H13NO4 | -23.09 | 27 | 4 | -10.987 | -2.7015 | -14.36 | 0 | 16.628 | 9.1015 | -81.41924 | 0.9855 | 1 |
| 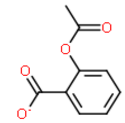 | 0 | 823 | 945 | -23.09 | 20 | 1 | -8.5167 | -3.067 | -15.8 | 0.8661 | 13.349 | 7.8539 | -74.41309 | 0.88954 | 1 |
| 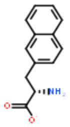 | 0 | 1548 | NAL | -23.05 | 28 | 4 | -8.7023 | -3.4653 | -17.7 | 0.6614 | 14.355 | 8.2229 | -76.05402 | 1.03654 | 1 |
| 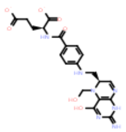 | 0 | 2505 | C20H23N7O7 | -23.02 | 55 | 10 | -13.838 | -3.9731 | -28.88 | 6.0846 | 29.732 | 17.541 | -132.1365 | 1.84235 | 1 |
| 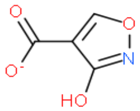 | 0 | 1869 | C4H3NO4 | -23 | 11 | 1 | -8.8762 | -1.155 | -12.33 | 0 | 13.55 | 2.7371 | -50.08942 | 0.45039 | 1 |
| 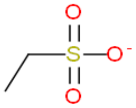 | 0 | 3284 | C2H6O3S | -22.98 | 11 | 1 | -8.6361 | -1.7867 | -9.632 | 0.0729 | 10.212 | 2.1082 | -35.20326 | 0.38268 | 1 |
| 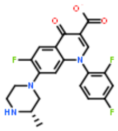 | 0 | 1213 | 1405 | -22.98 | 47 | 1 | -6.8283 | -5.018 | -22.93 | 2.4723 | 17.406 | 8.2578 | -134.5999 | 1.4768 | 1 |
| 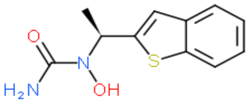 | 0 | 627 | 744 | -22.98 | 28 | 4 | -7.3415 | -3.367 | -17.08 | 1.1049 | 12.648 | 3.6095 | -48.31292 | 0.58085 | 1 |
| 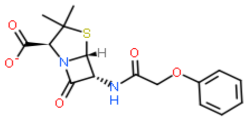 | 0 | 307 | 417 | -22.98 | 41 | 5 | -9.9712 | -4.6445 | -20.06 | 4.9487 | 19.19 | 7.6707 | -116.4599 | 1.39766 | 1 |
| 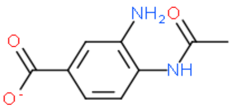 | 0 | 2012 | ST3 | -22.97 | 23 | 1 | -12.42 | -2.8145 | -10.15 | 2.7391 | 15.323 | 10.221 | -53.59747 | 0.83199 | 1 |
| 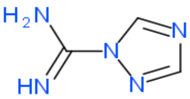 | 0 | 1808 | C3H6N5 | -22.96 | 14 | 0 | -7.312 | -1.4852 | -10.52 | 0 | 7.0188 | 2.715 | 15.055496 | 0.40169 | 1 |

| | | | | | | | | | | | | | | | |
|---|---|---|---|---|---|---|---|---|---|---|---|---|---|---|---|
| 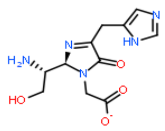 | 0 | 2095 | C11H15N5O4 | -22.96 | 34 | 8 | -12.432 | -3.4597 | -18.72 | 4.7271 | 18.813 | 11.801 | -96.29919 | 1.31406 | 1 |
| 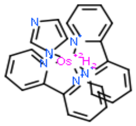 | 0 | 3156 | C23H21N6Os | -22.95 | 49 | 1 | -0.104 | -4.0057 | -11.01 | 3.0895 | 3.0006 | -20.75 | -34.01605 | 0.93532 | 1 |
| 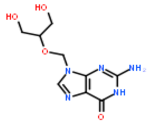 | 0 | 880 | C9H13N5O4 | -22.95 | 31 | 7 | -14.327 | -2.9821 | -21.96 | 4.2394 | 21.076 | 22.276 | -95.523 | 1.16743 | 1 |
| 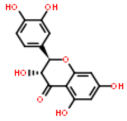 | 0 | 1972 | C15H12O7 | -22.94 | 34 | 2 | -8.3866 | -3.5242 | -23.6 | 0 | 18.995 | 13.318 | -105.1315 | 1.31406 | 1 |
| 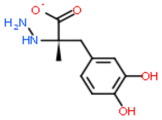 | 0 | 85 | C10H14N2O4 | -22.93 | 29 | 5 | -13.95 | -2.512 | -17.66 | 6.1878 | 22.059 | 12.802 | -93.68417 | 1.17754 | 1 |
| 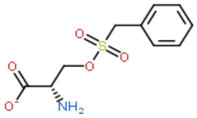 | 0 | 1737 | C10H13NO5S | -22.93 | 29 | 7 | -10.793 | -3.7747 | -19.82 | 0 | 16.82 | 15.014 | -106.3016 | 1.20174 | 1 |
| 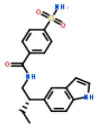 | 0 | 3248 | SBB | -22.92 | 47 | 6 | -9.9947 | -5.5611 | -30.85 | 9.8938 | 18.13 | 20.925 | -130.0492 | 1.81845 | 1 |
| 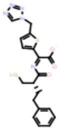 | 0 | 2422 | C19H19N5O3S2 | -22.92 | 47 | 9 | -10.769 | -5.225 | -26.07 | 9.5114 | 21.254 | 10.375 | -122.846 | 1.71434 | 1 |
| 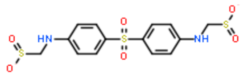 | 0 | 1016 | 1145 | -22.91 | 39 | 6 | -12.791 | -3.8845 | -23.11 | 8.0176 | 21.908 | 15.652 | -87.74868 | 1.7013 | 1 |
| 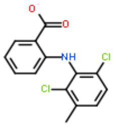 | 0 | 817 | 939 | -22.89 | 29 | 1 | -7.5533 | -4.8616 | -16.91 | 3.0897 | 10.716 | 8.9237 | -87.89622 | 1.28149 | 1 |
| 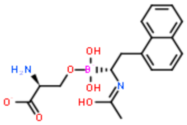 | 0 | 2967 | C17H22BN2O6 | -22.89 | 47 | 12 | -7.6355 | -4.5217 | -22.66 | 0 | 22.118 | -2.527 | -83.68323 | 1.4721 | 1 |

| | | | | | | | | | | | | | | | |
|---|---|---|---|---|---|---|---|---|---|---|---|---|---|---|---|
| 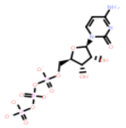 | 0 | 2167 | C9H16N3O14P3 | -22.89 | 41 | 10 | -18.583 | -2.4834 | -25.3 | 1.1114 | 31.438 | 31.586 | -100.1108 | 1.35864 | 1 |
| 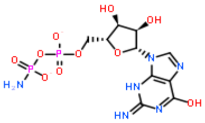 | 0 | 2346 | C10H16N6O10P2 | -22.87 | 42 | 10 | -14.225 | -3.1398 | -22.94 | 4.2187 | 30.124 | 10.676 | -123.9248 | 1.78893 | 1 |
| 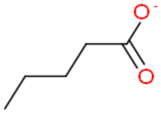 | 0 | 2142 | C5H10O2 | -22.86 | 16 | 3 | -8.5269 | -2.4673 | -9.918 | 0.1023 | 9.2723 | 2.2176 | -54.30912 | 0.52871 | 1 |
| 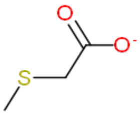 | 0 | 3180 | C3H5O2S | -22.85 | 11 | 2 | -8.2811 | -2.1775 | -9.219 | 1.9782 | 8.5957 | -0.075 | -33.7288 | 0.34788 | 1 |
| 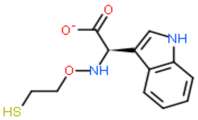 | 0 | 3122 | MPE | -22.84 | 31 | 7 | -10.542 | -3.2123 | -18.9 | 1.2887 | 16.391 | 11.537 | -94.94778 | 1.46557 | 1 |
| 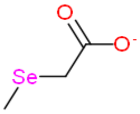 | 0 | 2785 | C3H5O2Se | -22.84 | 11 | 2 | -8.276 | -2.1783 | -9.218 | 1.972 | 8.6311 | -0.123 | -33.74164 | 0.34788 | 1 |
| 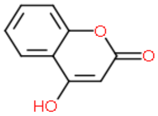 | 0 | 3078 | C9H6O3 | -22.81 | 18 | 0 | -7.7652 | -2.6555 | -15.29 | 1.248 | 11.826 | 6.4993 | -65.06949 | 0.75814 | 1 |
| 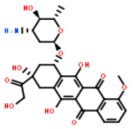 | 0 | 335 | 445 | -22.81 | 68 | 8 | -8.1834 | -5.944 | -32.62 | 0.3966 | 23.398 | 18.04 | -123.277 | 1.85255 | 1 |
| 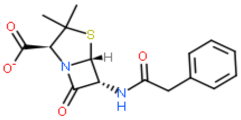 | 0 | 925 | 1053 | -22.81 | 40 | 4 | -10.047 | -4.2147 | -18.83 | 4.818 | 17.994 | 8.2991 | -113.3022 | 1.36611 | 1 |
| 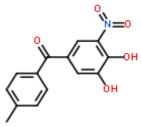 | 0 | 217 | 323 | -22.81 | 31 | 1 | -8.0693 | -2.9505 | -20.21 | 1.7734 | 12.915 | 12.797 | -80.92152 | 1.20916 | 1 |
| 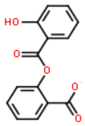 | 0 | 1209 | 1399 | -22.8 | 28 | 1 | -10.007 | -3.8953 | -20.92 | 3.5119 | 16.357 | 16.77 | -92.8527 | 1.19199 | 1 |

| | | | | | | | | | | | | | | | |
|---|---|---|---|---|---|---|---|---|---|---|---|---|---|---|---|
| 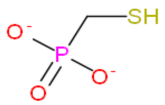 | 0 | 3161 | CH3O3PS | -22.78 | 9 | 2 | -8.9674 | -1.019 | -10.51 | 0 | 11.594 | 1.8374 | -28.97611 | 0.37983 | 1 |
| 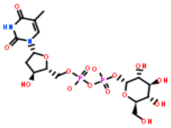 | 0 | 3391 | m | -22.77 | 60 | 14 | -22.266 | -3.5713 | -38.02 | 28.909 | 38.301 | 29.561 | -159.4653 | 2.05363 | 1 |
| 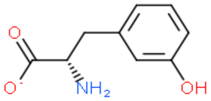 | 0 | 3208 | C9H11NO3 | -22.76 | 23 | 4 | -8.527 | -2.7767 | -16.88 | 0 | 14.871 | 6.057 | -61.26326 | 0.8417 | 1 |
| 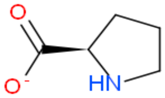 | 0 | 2556 | C5H9NO2 | -22.76 | 16 | 1 | -10.235 | -2.102 | -10.52 | 1.7727 | 12.747 | 5.8232 | -50.59149 | 0.4943 | 1 |
| 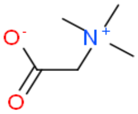 | 0 | 1296 | 1494 | -22.71 | 19 | 2 | -8.9548 | -2.7387 | -9.579 | 0.0784 | 9.2811 | 4.7776 | -44.62191 | 0.50752 | 1 |
| 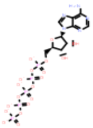 | 0 | 2451 | C10H18N5O19P5 | -22.67 | 51 | 14 | -12.828 | -3.5562 | -29.8 | 4.9108 | 34.766 | 6.8811 | -151.56 | 2.18017 | 1 |
| 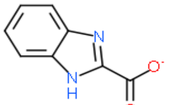 | 0 | 2721 | C8H6N2O2 | -22.66 | 17 | 1 | -9.8712 | -2.485 | -11.12 | 0.3569 | 13.019 | 6.8464 | -51.17206 | 0.68694 | 1 |
| 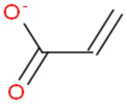 | 0 | 2305 | C3H4O2 | -22.66 | 8 | 1 | -8.0507 | -1.4971 | -8.199 | 0 | 8.4357 | -0.026 | -29.66091 | 0.30534 | 1 |
| 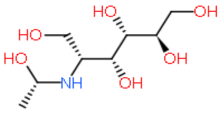 | 0 | 2493 | C8H19NO6 | -22.66 | 34 | 13 | -12.731 | -3.0122 | -12.84 | 0 | 15.551 | 7.5049 | -60.43383 | 0.95368 | 1 |
| 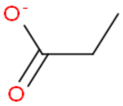 | 0 | 3405 | C3H6O2 | -22.64 | 10 | 1 | -8.698 | -1.609 | -7.791 | 0.1095 | 8.9923 | 1.2497 | -30.63184 | 0.31641 | 1 |
| 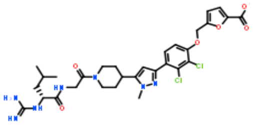 | 0 | 2307 | C30H37Cl2N7O6 | -22.64 | 81 | 12 | -14.926 | -7.5687 | -31.4 | 12.043 | 30.342 | 21.843 | -198.0607 | 3.19772 | 1 |

| | | | | | | | | | | | | | | | |
|---|---|---|---|---|---|---|---|---|---|---|---|---|---|---|---|
| 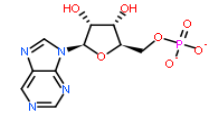 | 0 | 3367 | C10H13N4O7P | -22.63 | 33 | 6 | -13.511 | -3.415 | -21.78 | 3.8193 | 24.985 | 16.654 | -92.70321 | 1.28714 | 1 |
| 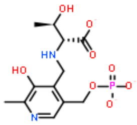 | 0 | 3230 | C12H19N2O8P | -22.63 | 39 | 9 | -10.831 | -3.8652 | -17.83 | 0 | 19.223 | 8.148 | -94.79747 | 1.40245 | 1 |
| 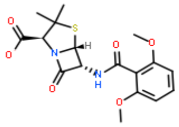 | 0 | 1396 | 1603 | -22.61 | 45 | 2 | -9.3092 | -5.0986 | -19.93 | 4.7974 | 18.472 | 9.6201 | -120.0208 | 1.44878 | 1 |
| 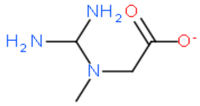 | 0 | 2220 | C4H11N3O2 | -22.6 | 19 | 5 | -9.9594 | -1.8123 | -11.21 | 0 | 15.856 | 0.1983 | -38.64139 | 0.68188 | 1 |
| 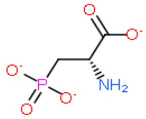 | 0 | 2969 | C3H8NO5P | -22.59 | 15 | 4 | -11.314 | -1.1342 | -11.9 | 0 | 15.77 | 6.7621 | -55.54069 | 0.57954 | 1 |
| 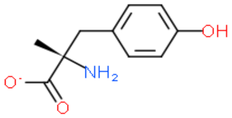 | 0 | 648 | 765 | -22.55 | 26 | 4 | -10.279 | -2.9158 | -14.75 | 0 | 15.5 | 9.2845 | -83.52178 | 0.86389 | 1 |
| 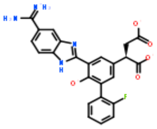 | 0 | 1525 | 693 | -22.55 | 51 | 4 | -14.093 | -4.0076 | -22.16 | 6.5567 | 24.734 | 19.8 | -142.2296 | 1.76204 | 1 |
| 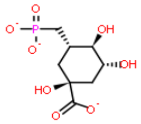 | 0 | 2317 | C8H15O8P | -22.54 | 29 | 6 | -12.314 | -2.1817 | -13.88 | 0 | 19.284 | 8.9163 | -79.63305 | 1.06489 | 1 |
| 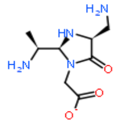 | 0 | 2781 | C8H16N4O3 | -22.54 | 30 | 6 | -11.701 | -2.796 | -13.48 | 2.5142 | 17.1 | 6.7966 | -80.5853 | 1.0484 | 1 |
| 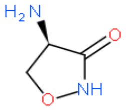 | 0 | 155 | C3H6N2O2 | -22.53 | 13 | 1 | -9.814 | -0.8868 | -8.793 | 0.2693 | 12.227 | 2.6534 | -5.076929 | 0.38689 | 1 |
| 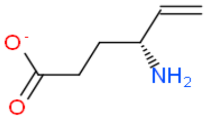 | 0 | 952 | 1080 | -22.52 | 19 | 5 | -11.159 | -2.2951 | -10.56 | 0.9795 | 11.634 | 8.3174 | -47.40371 | 0.63053 | 1 |

| | | | | | | | | | | | | | | | |
|---|---|---|---|---|---|---|---|---|---|---|---|---|---|---|---|
| 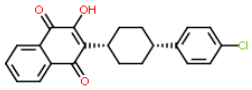 | 0 | 988 | m | -22.52 | 45 | 2 | -5.5166 | -5.1891 | -25.97 | 1.7606 | 13.458 | 12.929 | -129.0282 | 1.67562 | 1 |
| 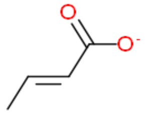 | 0 | 1832 | C4H6O2 | -22.48 | 11 | 1 | -8.411 | -1.8126 | -10.56 | 0.0841 | 10.097 | 3.5699 | -29.27105 | 0.46069 | 1 |
| 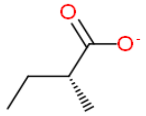 | 0 | 3382 | C5H10O2 | -22.41 | 16 | 2 | -8.641 | -2.568 | -9.835 | 1.8657 | 9.0785 | 2.8397 | -47.58089 | 0.45642 | 1 |
| 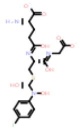 | 0 | 3006 | C17H23IN4O8S | -22.4 | 52 | 18 | -11.719 | -4.637 | -26.05 | 2.2307 | 20.787 | 13.149 | -108.5239 | 1.89631 | 1 |
| 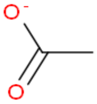 | 0 | 153 | 6116 | -22.4 | 7 | 0 | -8.5157 | -1.1745 | -6.372 | 0 | 8.4729 | -0.076 | -26.22062 | 0.24289 | 1 |
| 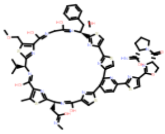 | 0 | 2671 | C56H55N15O10S6 | -22.4 | 142 | 17 | -14.199 | -9.6425 | -38.79 | 8.5228 | 39.142 | 21.373 | -211.2964 | 4.02736 | 1 |
| 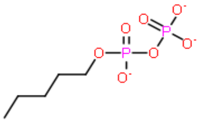 | 0 | 2423 | C5H14O7P2 | -22.39 | 25 | 7 | -14.384 | -2.6949 | -15.42 | 0.2625 | 17.661 | 20.655 | -86.51594 | 1.04181 | 1 |
| 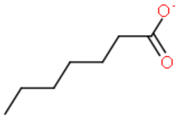 | 0 | 2636 | C7H14O2 | -22.38 | 22 | 5 | -8.8196 | -3.12 | -13.06 | 1.4724 | 10.829 | 4.6637 | -57.47213 | 0.73958 | 1 |
| 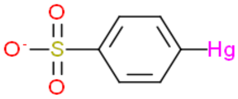 | 0 | 2679 | C6H5HgO3S | -22.38 | 15 | 1 | -9.1693 | -2.1984 | -15.34 | 0.5197 | 11.731 | 12 | -40.05001 | 0.77676 | 1 |
| 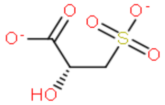 | 0 | 2075 | C3H6O6S | -22.38 | 14 | 4 | -10.488 | -1.3092 | -11.23 | 0.4742 | 15.548 | 2.9064 | -53.25687 | 0.59504 | 1 |
| 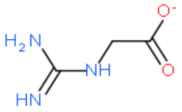 | 0 | 2462 | C3H7N3O2 | -22.37 | 14 | 3 | -10.068 | -0.9216 | -10.16 | 0 | 15.994 | 0.1321 | -32.75169 | 0.4893 | 1 |

| | | | | | | | | | | | | | | | |
|---|---|---|---|---|---|---|---|---|---|---|---|---|---|---|---|
| 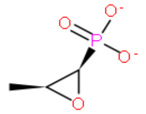 | 0 | 709 | C3H7O4P | -22.36 | 13 | 1 | -10.39 | -1.8353 | -9.88 | 0.476 | 11.924 | 7.8876 | -60.70136 | 0.45316 | 1 |
| 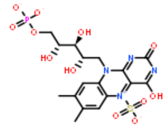 | 0 | 1918 | C17H21N4O12PS | -22.35 | 54 | 12 | -13.28 | -3.8803 | -21.94 | 1.55 | 22.559 | 16.271 | -110.6087 | 1.54038 | 1 |
| 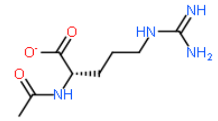 | 0 | 1749 | C8H16N4O3 | -22.34 | 30 | 7 | -10.861 | -2.973 | -18.57 | 4.056 | 17.656 | 8.8342 | -76.13799 | 0.95534 | 1 |
| 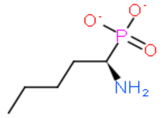 | 0 | 1846 | C5H14NO3P | -22.34 | 22 | 5 | -11.15 | -2.3884 | -11.75 | 0 | 15.625 | 6.8289 | -49.70116 | 0.72779 | 1 |
| 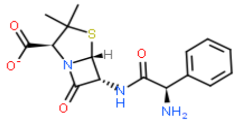 | 0 | 305 | 415 | -22.33 | 42 | 5 | -9.332 | -4.3669 | -23.71 | 7.497 | 18.026 | 10.456 | -114.1239 | 1.35437 | 1 |
| 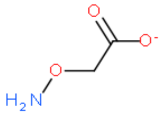 | 0 | 1837 | C2H5NO3 | -22.32 | 10 | 3 | -10.429 | -0.7789 | -9.02 | 2.68 | 14.24 | -0.727 | -35.6741 | 0.28265 | 1 |
| 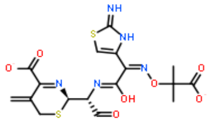 | 0 | 3189 | C17H19N5O7S2 | -22.32 | 48 | 8 | -11.445 | -4.5313 | -28.28 | 1.9356 | 26.011 | 18.911 | -120.0521 | 1.78767 | 1 |
| 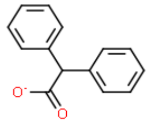 | 0 | 3240 | DFA | -22.3 | 27 | 3 | -8.7861 | -4.0261 | -17.59 | 1.8949 | 13.471 | 10.866 | -90.23071 | 1.16504 | 1 |
| 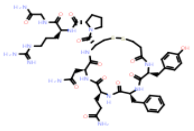 | 0 | 4 | [NO | -22.29 | 138 | 21 | -13.612 | -8.3651 | -58.95 | 18.424 | 45.893 | 28.234 | -241.6575 | 3.83164 | 1 |
| 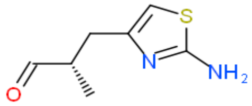 | 0 | 2718 | HII | -22.27 | 21 | 3 | -10.376 | -3.4558 | -13.92 | 0.8037 | 13.64 | 11.674 | -61.11819 | 0.80691 | 1 |
| 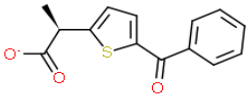 | 0 | 1393 | 1600 | -22.25 | 29 | 3 | -9.815 | -4.344 | -21.17 | 4.6787 | 15.071 | 16.301 | -112.4359 | 1.27519 | 1 |

| | | | | | | | | | | | | | | | |
|---|---|---|---|---|---|---|---|---|---|---|---|---|---|---|---|
| 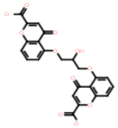 | 0 | 879 | C23H16O11 | -22.24 | 48 | 7 | -12.971 | -3.3432 | -22.88 | 2.1803 | 25.418 | 16.865 | -133.2613 | 1.86166 | 1 |
| 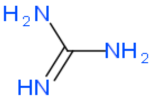 | 0 | 423 | CH5N3 | -22.21 | 9 | 0 | -7.7259 | -0.1148 | -9.388 | 0 | 8.3851 | 0.7811 | -2.362806 | 0.01421 | 1 |
| 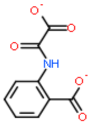 | 0 | 2345 | OBA | -22.19 | 20 | 2 | -8.4132 | -2.4141 | -16.3 | 0 | 12.728 | 9.5007 | -81.63066 | 0.99191 | 1 |
| 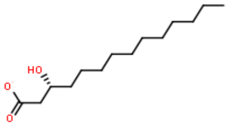 | 0 | 2477 | FTT | -22.16 | 44 | 13 | -11.53 | -5.0443 | -19.92 | 5.7514 | 14.192 | 12.755 | -95.58865 | 1.26027 | 1 |
| 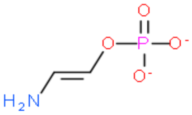 | 0 | 2607 | C2H6NO4P | -22.16 | 12 | 1 | -10.003 | -1.5063 | -11.2 | 0.1987 | 14.202 | 6.0465 | -35.2538 | 0.47942 | 1 |
| 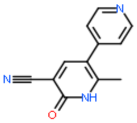 | 0 | 130 | 235 | -22.14 | 25 | 1 | -8.4705 | -2.4621 | -13.01 | 1.4352 | 9.894 | 7.6776 | -36.80618 | 0.52573 | 1 |
| 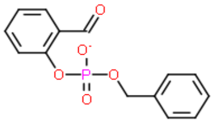 | 0 | 2767 | PSN | -22.14 | 32 | 4 | -8.9432 | -4.3414 | -21.08 | 5.3804 | 15.833 | 10.571 | -99.15884 | 1.44471 | 1 |
| 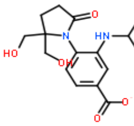 | 0 | 3139 | RA2 | -22.12 | 50 | 8 | -8.3282 | -4.7247 | -23 | 10.583 | 16.199 | 2.7543 | -116.1525 | 1.74927 | 1 |
| 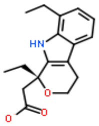 | 0 | 632 | 749 | -22.12 | 41 | 4 | -9.2401 | -4.9193 | -13.99 | 3.6529 | 13.221 | 6.1185 | -121.7915 | 1.27498 | 1 |
| 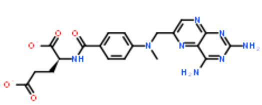 | 0 | 450 | 563 | -22.12 | 53 | 7 | -13.699 | -5.9848 | -33.56 | 9.094 | 28.36 | 29.063 | -171.7085 | 1.55013 | 1 |
| 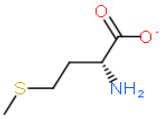 | 0 | 2593 | C5H11NO2S | -22.12 | 19 | 5 | -10.795 | -2.5336 | -13.06 | 1.3271 | 13.525 | 9.1096 | -50.56846 | 0.73409 | 1 |

| | | | | | | | | | | | | | | | |
|---|---|---|---|---|---|---|---|---|---|---|---|---|---|---|---|
| 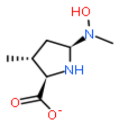 | 0 | 1630 | C7H14N2O3 | -22.08 | 25 | 3 | -9.329 | -2.6931 | -14.01 | 1.2212 | 16.194 | 4.025 | -69.46026 | 0.66909 | 1 |
| 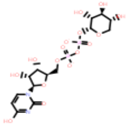 | 0 | 1499 | C14H22N2O16P2 | -22.07 | 54 | 14 | -17.252 | -3.5343 | -25.87 | 5.6801 | 30.489 | 22.94 | -156.462 | 1.99679 | 1 |
| 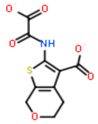 | 0 | 2789 | OPA | -22.06 | 25 | 2 | -9.8555 | -2.5972 | -15.12 | 2.0659 | 14.589 | 9.6322 | -80.11003 | 1.11903 | 1 |
| 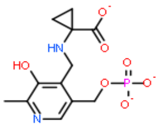 | 0 | 2552 | C12H17N2O7P | -22.06 | 36 | 7 | -10.872 | -3.7308 | -16.52 | 0 | 20.781 | 7.0956 | -91.30761 | 1.3666 | 1 |
| 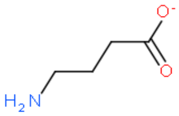 | 0 | 2260 | C4H9NO2 | -22.05 | 15 | 4 | -9.6121 | -1.6291 | -10.2 | 2.2198 | 10.765 | 2.6616 | -45.09626 | 0.42583 | 1 |
| 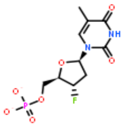 | 0 | 2876 | FDM | -22.05 | 33 | 4 | -12.655 | -2.9121 | -23.24 | 8.2971 | 22.145 | 16.938 | -84.00879 | 1.20152 | 1 |
| 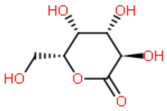 | 0 | 1658 | C6H10O6 | -22.04 | 22 | 5 | -14.348 | -1.6626 | -6.012 | 4.0538 | 15.303 | 6.784 | -58.39099 | 0.53003 | 1 |
| 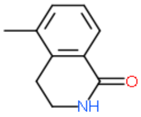 | 0 | 3363 | C10H11NO | -22.04 | 23 | 0 | -8.5511 | -3.1821 | -12.76 | 0.6662 | 11.518 | 8.259 | -82.69239 | 0.96854 | 1 |
| 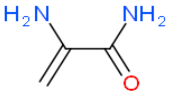 | 0 | 1765 | C3H6N2O | -22.02 | 12 | 0 | -9.2469 | -1.3789 | -9.387 | 0 | 11.572 | 4.5501 | -25.50561 | 0.31588 | 1 |
| 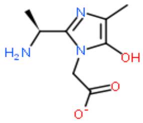 | 0 | 2760 | C8H13N3O3 | -22.02 | 26 | 4 | -9.9964 | -2.9241 | -12.07 | 0 | 14.992 | 5.5534 | -72.61813 | 1.0529 | 1 |
| 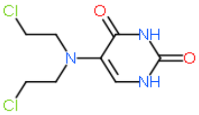 | 0 | 672 | 791 | -22.01 | 26 | 4 | -8.7108 | -3.6215 | -17.38 | 0.7436 | 13.508 | 10.396 | -70.03296 | 1.04152 | 1 |

| | | | | | | | | | | | | | | | |
|---|---|---|---|---|---|---|---|---|---|---|---|---|---|---|---|
| 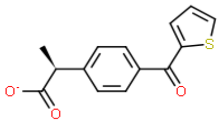 | 0 | 750 | 870 | -22.01 | 29 | 2 | -9.5179 | -4.4824 | -19.59 | 4.6068 | 14.694 | 14.664 | -130.2652 | 1.27154 | 1 |
| 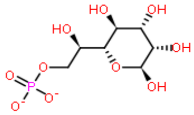 | 0 | 2203 | C7H15O10P | -22 | 31 | 9 | -14.6 | -2.2213 | -15.14 | 8.7162 | 21.027 | 7.8247 | -89.64694 | 1.05385 | 1 |
| 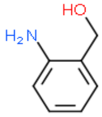 | 0 | 2747 | C7H9NO | -21.99 | 18 | 2 | -8.6381 | -3.0005 | -13.16 | 2.0649 | 11.813 | 5.6652 | -48.29093 | 0.56108 | 1 |
| 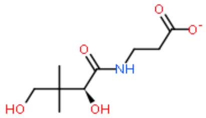 | 0 | 1562 | C9H17NO5 | -21.98 | 31 | 8 | -13.662 | -3.0866 | -16.9 | 8.3938 | 20.053 | 10.108 | -85.88841 | 1.19337 | 1 |
| 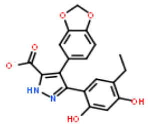 | 0 | 2543 | 4BC | -21.97 | 42 | 2 | -8.0331 | -3.3972 | -24.12 | 0 | 19.693 | 13.305 | -109.0669 | 1.56143 | 1 |
| 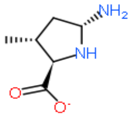 | 0 | 2866 | C6H12N2O2 | -21.94 | 21 | 2 | -8.8874 | -2.3483 | -12.28 | 0.2392 | 14.544 | 3.2589 | -60.05953 | 0.65416 | 1 |
| 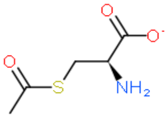 | 0 | 1800 | C5H9NO3S | -21.91 | 18 | 4 | -10.23 | -2.1336 | -14.66 | 1.6976 | 16.205 | 6.7967 | -47.95402 | 0.76978 | 1 |
| 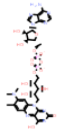 | 0 | 3146 | C28H36N10O15P2 | -21.88 | 89 | 19 | -12.877 | -5.5989 | -42.52 | 11.171 | 34.649 | 19.514 | -186.9893 | 2.69216 | 1 |
| 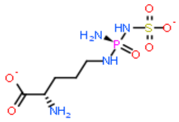 | 0 | 2661 | C5H15N4O6PS | -21.85 | 30 | 10 | -14.633 | -2.0595 | -19.46 | 0 | 22.345 | 20.071 | -81.05943 | 1.25142 | 1 |
| 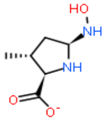 | 0 | 2040 | C6H12N2O3 | -21.84 | 22 | 3 | -9.3109 | -2.2586 | -13.25 | 0.9697 | 16.344 | 2.7882 | -60.82795 | 0.57662 | 1 |
| 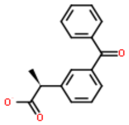 | 0 | 885 | 1009 | -21.83 | 32 | 3 | -9.2751 | -4.4742 | -19.57 | 2.4669 | 14.805 | 14.882 | -124.7484 | 1.35546 | 1 |

| | | | | | | | | | | | | | | | |
|---|---|---|---|---|---|---|---|---|---|---|---|---|---|---|---|
| 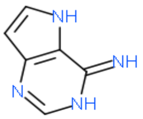 | 0 | 3169 | C6H6N4 | -21.79 | 16 | 0 | -6.8765 | -1.0217 | -13.75 | 0.2627 | 10.26 | 3.3176 | -11.85847 | 0.62177 | 1 |
| 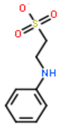 | 0 | 2027 | 171 | -21.76 | 23 | 4 | -9.6848 | -3.2409 | -13.01 | 1.3756 | 12.77 | 7.764 | -66.62038 | 0.92728 | 1 |
| 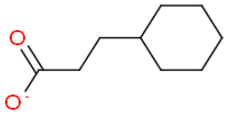 | 0 | 1989 | C9H16O2 | -21.75 | 26 | 3 | -8.4669 | -3.4115 | -12.48 | 1.317 | 10.988 | 5.4521 | -57.77161 | 0.81634 | 1 |
| 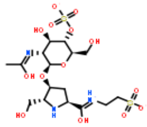 | 0 | 2320 | C16H30N3O14S2 | -21.73 | 63 | 16 | -14.853 | -4.5154 | -22.98 | 0 | 34.261 | 9.945 | -128.2223 | 2.1559 | 1 |
| 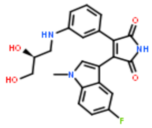 | 0 | 1552 | DFN | -21.68 | 50 | 5 | -9.7152 | -4.2824 | -30.29 | 7.6085 | 21.849 | 18.345 | -142.4843 | 1.93615 | 1 |
| 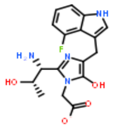 | 0 | 1463 | C17H19FN4O4 | -21.67 | 44 | 8 | -9.9283 | -4.0889 | -22.54 | 0 | 22.787 | 10.362 | -126.616 | 1.37942 | 1 |
| 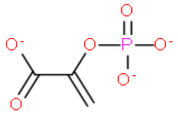 | 0 | 1596 | C3H5O6P | -21.67 | 12 | 2 | -13.711 | -1.0913 | -13.88 | 7.427 | 15.237 | 15.969 | -66.33882 | 0.52392 | 1 |
| 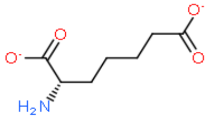 | 0 | 2817 | C7H13NO4 | -21.66 | 23 | 7 | -9.9167 | -2.3771 | -13.47 | 0 | 15.846 | 3.6453 | -59.97044 | 0.89846 | 1 |
| 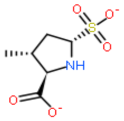 | 0 | 1993 | C6H11NO5S | -21.65 | 22 | 2 | -11.311 | -2.2523 | -14.46 | 0 | 17.644 | 12.881 | -80.81945 | 0.82224 | 1 |
| 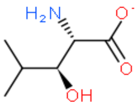 | 0 | 2674 | C6H13NO3 | -21.63 | 22 | 5 | -9.8481 | -2.4381 | -12.64 | 0 | 10.492 | 10.148 | -52.15453 | 0.68718 | 1 |
| 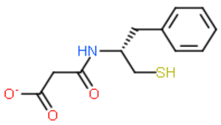 | 0 | 1700 | RRT | -21.63 | 31 | 7 | -9.5601 | -3.5446 | -14.47 | 0.7099 | 13.598 | 6.9357 | -88.89524 | 1.23637 | 1 |

| | | | | | | | | | | | | | | | |
|---|---|---|---|---|---|---|---|---|---|---|---|---|---|---|---|
| 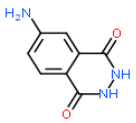 | 0 | 1801 | C8H7N3O2 | -21.62 | 20 | 0 | -7.5945 | -2.2561 | -18.1 | 0.5117 | 14.167 | 9.5054 | -67.11983 | 0.74168 | 1 |
| 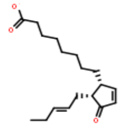 | 0 | 2702 | C18H28O3 | -21.61 | 48 | 11 | -10.174 | -6.0949 | -25.63 | 4.8259 | 17.928 | 16.522 | -127.1817 | 1.34328 | 1 |
| 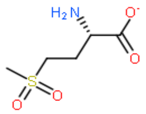 | 0 | 3427 | C5H11NO4S | -21.6 | 21 | 5 | -8.5873 | -2.4127 | -15.99 | 0 | 13.472 | 7.0058 | -60.92986 | 0.81087 | 1 |
| 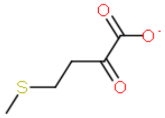 | 0 | 1985 | C5H8O3S | -21.6 | 16 | 4 | -8.536 | -2.5617 | -12.76 | 0 | 11.823 | 4.8608 | -53.71761 | 0.71896 | 1 |
| 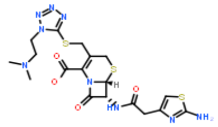 | 0 | 124 | 229 | -21.59 | 56 | 9 | -11.273 | -4.7764 | -33.95 | 4.5568 | 28.323 | 22.278 | -110.4846 | 2.07362 | 1 |
| 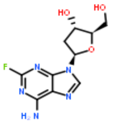 | 0 | 2643 | C10H12FN5O3 | -21.56 | 31 | 4 | -11.455 | -4.1832 | -13.26 | 0 | 19.175 | 10.157 | -104.5411 | 1.23265 | 1 |
| 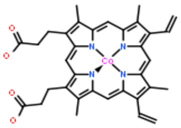 | 0 | 1868 | C34H32CoN4O4 | -21.53 | 73 | 6 | -8.3301 | -7.794 | -29.63 | 4.4454 | 23.193 | 16.258 | -163.5097 | 2.17146 | 1 |
| 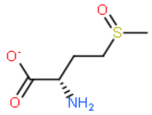 | 0 | 1982 | C5H11NO3S | -21.53 | 20 | 5 | -9.8918 | -2.2628 | -13.59 | 0 | 15.268 | 6.3007 | -49.78599 | 0.73517 | 1 |
| 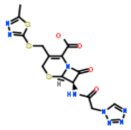 | 0 | 1161 | C14H14N8O4S3 | -21.5 | 42 | 6 | -12.064 | -4.9877 | -26.13 | 5.3514 | 30.696 | 13.113 | -119.2164 | 1.96313 | 1 |
| 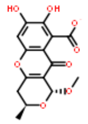 | 0 | 2318 | 113 | -21.48 | 36 | 2 | -10.187 | -3.8766 | -20.51 | 0 | 20.883 | 15.966 | -121.3245 | 1.52139 | 1 |
| 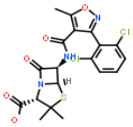 | 0 | 375 | 485 | -21.48 | 46 | 2 | -9.5107 | -5.1136 | -20.17 | 10.487 | 19.557 | 6.0829 | -120.3052 | 1.61458 | 1 |

| | | | | | | | | | | | | | | | |
|---|---|---|---|---|---|---|---|---|---|---|---|---|---|---|---|
| 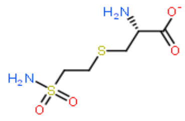 | 0 | 2406 | C5H12N2O4S2 | -21.47 | 24 | 8 | -12.119 | -2.1638 | -14.93 | 0 | 19.524 | 9.285 | -83.44823 | 1.13879 | 1 |
| 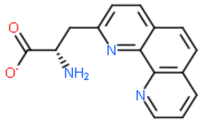 | 0 | 1449 | C15H13N3O2 | -21.47 | 32 | 4 | -8.4925 | -3.9571 | -18.77 | 2.5524 | 16.771 | 7.467 | -86.76008 | 1.17072 | 1 |
| 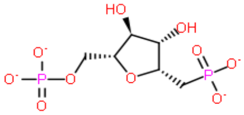 | 0 | 2129 | C6H14O10P2 | -21.46 | 28 | 7 | -12.149 | -2.1192 | -12.22 | 0 | 17.512 | 8.4632 | -92.2765 | 0.98125 | 1 |
| 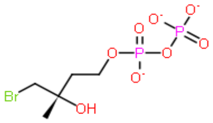 | 0 | 2211 | C5H13BrO8P2 | -21.44 | 26 | 8 | -15.572 | -2.951 | -18.37 | 3.1884 | 18.673 | 26.656 | -101.0036 | 1.17103 | 1 |
| 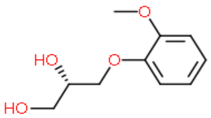 | 0 | 754 | 874 | -21.43 | 28 | 5 | -9.5912 | -4.2014 | -17.37 | 4.6854 | 15.445 | 8.5176 | -82.86591 | 0.92009 | 1 |
| 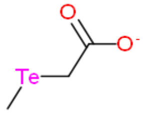 | 0 | 1684 | C3H5O2Te | -21.43 | 11 | 2 | -8.1668 | -2.1907 | -9.158 | 1.9731 | 8.6291 | 1.5197 | -33.01395 | 0.34746 | 1 |
| 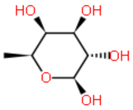 | 0 | 2960 | C6H12O5 | -21.43 | 23 | 4 | -12.901 | -2.0958 | -10.9 | 4.2428 | 16.888 | 8.9403 | -65.66116 | 0.72056 | 1 |
| 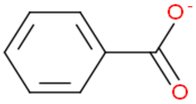 | 0 | 3430 | C7H6O2 | -21.42 | 14 | 1 | -8.2667 | -2.4004 | -11.23 | 0.4658 | 9.846 | 6.1629 | -43.54774 | 0.52691 | 1 |
| 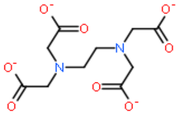 | 0 | 851 | C10H16N2O8 | -21.42 | 32 | 11 | -16.97 | -2.5122 | -15.14 | 4.9921 | 20.724 | 19.997 | -94.71121 | 1.2361 | 1 |
| 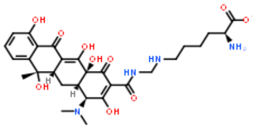 | 0 | 151 | m | -21.42 | 80 | 12 | -11.475 | -6.2305 | -34.62 | 11.988 | 25.947 | 19.018 | -169.9727 | 2.31983 | 1 |
| 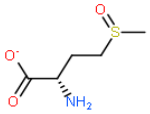 | 0 | 2200 | C5H11NO3S | -21.41 | 20 | 5 | -9.8979 | -2.3041 | -14.87 | 0 | 15.263 | 8.4743 | -58.23201 | 0.79947 | 1 |

| | | | | | | | | | | | | | | | |
|---|---|---|---|---|---|---|---|---|---|---|---|---|---|---|---|
| 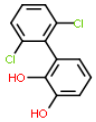 | 0 | 2940 | BP6 | -21.39 | 24 | 0 | -4.8749 | -4.2216 | -18.58 | 1.6729 | 9.6381 | 6.2425 | -72.58667 | 1.01752 | 1 |
| 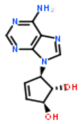 | 0 | 2897 | C10H11N5O2 | -21.39 | 28 | 3 | -10.149 | -3.728 | -16.19 | 0 | 21.907 | 7.1854 | -59.69867 | 0.9074 | 1 |
| 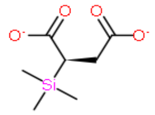 | 0 | 3119 | C7H14O4Si | -21.37 | 24 | 4 | -10.141 | -3.0299 | -9.817 | 0.7143 | 12.768 | 5.6449 | -70.27564 | 0.85385 | 1 |
| 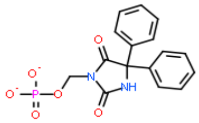 | 0 | 1155 | 1320 | -21.36 | 38 | 5 | -9.5412 | -4.1975 | -23.8 | 0.4539 | 21.794 | 14.808 | -98.2621 | 1.39589 | 1 |
| 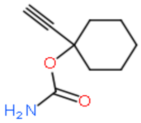 | 0 | 906 | 1031 | -21.36 | 25 | 1 | -10.09 | -3.2604 | -8.38 | 0.2432 | 12.207 | 7.3288 | -84.05475 | 0.75676 | 1 |
| 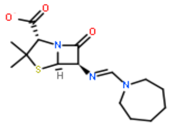 | 0 | 1033 | C15H23N3O3S | -21.32 | 44 | 3 | -8.8395 | -4.7832 | -20.17 | 4.0088 | 16.617 | 11.736 | -110.5061 | 1.39559 | 1 |
| 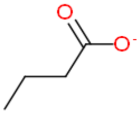 | 0 | 3222 | C4H8O2 | -21.28 | 13 | 2 | -8.6535 | -2.0303 | -9.244 | 0.1443 | 9.7764 | 3.9438 | -35.90319 | 0.48323 | 1 |
| 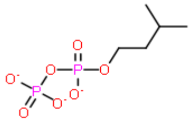 | 0 | 2238 | IPR | -21.25 | 25 | 6 | -14.332 | -2.8033 | -16.45 | 1.4475 | 17.992 | 23.299 | -94.90257 | 1.12569 | 1 |
| 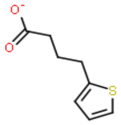 | 0 | 2170 | 4TB | -21.24 | 20 | 4 | -8.1248 | -3.3457 | -11.77 | 0.0717 | 10.365 | 4.7213 | -62.60078 | 0.84232 | 1 |
| 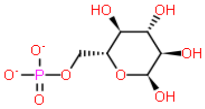 | 0 | 1769 | m | -21.23 | 27 | 7 | -13.06 | -1.9415 | -14.42 | 0 | 19.582 | 13.095 | -101.7675 | 1.00231 | 1 |
| 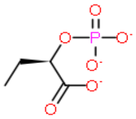 | 0 | 2929 | C4H9O6P | -21.21 | 17 | 4 | -12.4 | -1.681 | -12.18 | 2.491 | 15.885 | 11.599 | -60.06585 | 0.5521 | 1 |

| | | | | | | | | | | | | | | | |
|---|---|---|---|---|---|---|---|---|---|---|---|---|---|---|---|
| 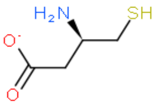 | 0 | 2487 | C4H9NO2S | -21.19 | 16 | 5 | -8.3405 | -1.5187 | -11.84 | 2.1855 | 9.1909 | 2.3665 | -46.12163 | 0.4148 | 1 |
| 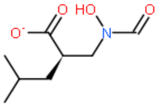 | 0 | 3328 | STN | -21.19 | 27 | 7 | -10.787 | -2.7864 | -13.04 | 0 | 12.892 | 10.815 | -93.61121 | 1.06991 | 1 |
| 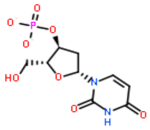 | 0 | 3115 | C9H13N2O8P | -21.18 | 31 | 5 | -11.603 | -2.6097 | -15.31 | 0 | 17.254 | 14.085 | -94.06352 | 1.13642 | 1 |
| 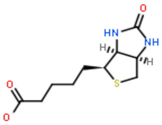 | 0 | 18 | 121 | -21.18 | 31 | 5 | -10.803 | -3.522 | -14.82 | 0.1054 | 16.845 | 11.572 | -98.63858 | 1.22795 | 1 |
| 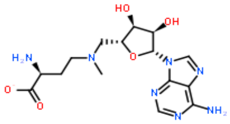 | 0 | 3124 | C15H23N7O5 | -21.17 | 49 | 10 | -16.652 | -4.7256 | -23.42 | 8.7004 | 28.553 | 22.595 | -108.312 | 1.70841 | 1 |
| 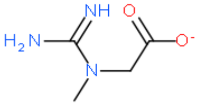 | 0 | 44 | C4H9N3O2 | -21.15 | 17 | 3 | -10.607 | -1.5907 | -8.17 | 0 | 12.593 | 5.5668 | -43.85708 | 0.47093 | 1 |
| 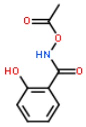 | 0 | 3314 | SCL | -21.15 | 23 | 1 | -10.378 | -3.4323 | -18.47 | 0.9404 | 16.234 | 18.994 | -59.88992 | 1.13827 | 1 |
| 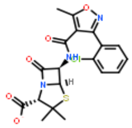 | 0 | 1018 | 1147 | -21.15 | 46 | 2 | -9.47 | -4.3598 | -21.22 | 8.7662 | 18.859 | 9.6137 | -132.1933 | 1.43699 | 1 |
| 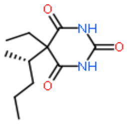 | 0 | 206 | 312 | -21.15 | 34 | 4 | -7.0906 | -2.9385 | -15.13 | 1.0542 | 9.2298 | 5.9586 | -38.64787 | 0.994 | 1 |
| 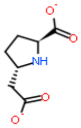 | 0 | 2896 | C7H11NO4 | -21.15 | 21 | 3 | -11.127 | -2.1626 | -12.16 | 1.1884 | 16.261 | 8.9466 | -78.19735 | 0.75404 | 1 |
| 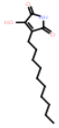 | 0 | 2753 | DHP | -21.13 | 41 | 9 | -8.5262 | -4.3525 | -22.71 | 1.5004 | 16.199 | 11.544 | -95.23012 | 1.17899 | 1 |

| | | | | | | | | | | | | | | | |
|---|---|---|---|---|---|---|---|---|---|---|---|---|---|---|---|
| 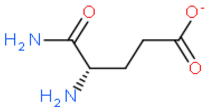 | 0 | 2778 | C5H10N2O3 | -21.13 | 19 | 5 | -12.434 | -1.3808 | -10.9 | 1.7408 | 18.454 | 6.4638 | -50.45326 | 0.63515 | 1 |
| 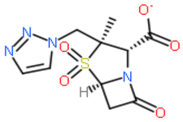 | 0 | 1399 | C10H12N4O5S | -21.09 | 31 | 3 | -10.094 | -3.1729 | -15.48 | 3.5573 | 17.523 | 7.5304 | -92.94733 | 1.21939 | 1 |
| 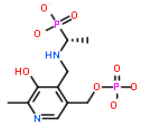 | 0 | 3003 | C10H18N2O8P2 | -21.09 | 36 | 7 | -10.248 | -3.6412 | -16.52 | 0 | 20.681 | 6.2055 | -93.63799 | 1.32812 | 1 |
| 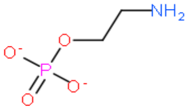 | 0 | 1522 | C2H8NO4P | -21.07 | 14 | 4 | -11.121 | -1.3053 | -10.89 | 0 | 13.035 | 10.091 | -46.16232 | 0.47079 | 1 |
| 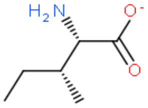 | 0 | 1523 | C6H13NO2 | -21.05 | 21 | 4 | -10.124 | -2.4432 | -12.33 | 3.6466 | 11.076 | 8.5291 | -65.62891 | 0.60202 | 1 |
| 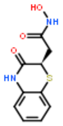 | 0 | 1797 | GNR | -21.04 | 26 | 4 | -9.6254 | -2.7488 | -18.97 | 2.1102 | 19.824 | 8.4696 | -82.93111 | 1.22992 | 1 |
| 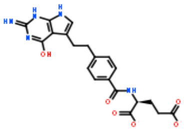 | 0 | 529 | C20H21N5O6 | -21.03 | 50 | 9 | -13.141 | -4.4596 | -28.3 | 2.4521 | 26.795 | 25.049 | -153.2647 | 1.66024 | 1 |
| 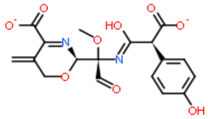 | 0 | 2313 | C18H18N2O9 | -21.03 | 45 | 9 | -10.861 | -3.8003 | -22.47 | 0 | 21.924 | 14.579 | -127.4043 | 1.52802 | 1 |
| 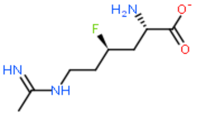 | 0 | 1612 | C8H16FN3O2 | -21.02 | 29 | 7 | -10.622 | -2.5185 | -15.52 | 1.4616 | 19.077 | 5.5782 | -9.860905 | 0.89642 | 1 |
| 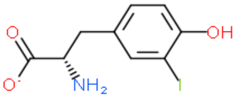 | 0 | 1540 | C9H10INO3 | -20.99 | 23 | 4 | -8.6594 | -3.1417 | -17.64 | 1.3083 | 16.479 | 7.76 | -59.5028 | 0.98327 | 1 |
| 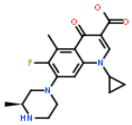 | 0 | 258 | 365 | -20.97 | 47 | 2 | -7.4334 | -5.2481 | -19.88 | 4.5308 | 16.317 | 7.6924 | -127.1145 | 1.46772 | 1 |

| | | | | | | | | | | | | | | | |
|---|---|---|---|---|---|---|---|---|---|---|---|---|---|---|---|
| 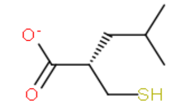 | 0 | 2692 | SLE | -20.97 | 23 | 5 | -9.2733 | -2.5711 | -12.38 | 0.6107 | 12.692 | 5.6198 | -68.60783 | 0.85311 | 1 |
| 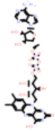 | 0 | 2073 | C27H34N9O15P2 | -20.96 | 85 | 19 | -16.299 | -5.7351 | -42.16 | 23.406 | 40.193 | 16.204 | -203.3366 | 2.43941 | 1 |
| 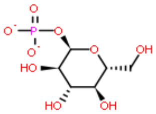 | 0 | 2546 | C6H13O9P | -20.94 | 27 | 7 | -12.641 | -1.9821 | -15.65 | 0 | 16.467 | 17.378 | -85.77815 | 0.87702 | 1 |
| 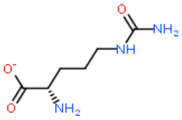 | 0 | 51 | C6H13N3O3 | -20.93 | 24 | 7 | -12.321 | -1.7477 | -14.98 | 0 | 19.399 | 11.573 | -80.34586 | 1.00035 | 1 |
| 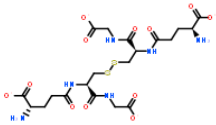 | 0 | 2987 | C20H32N6O12S2 | -20.93 | 68 | 23 | -13.669 | -4.1976 | -34.62 | 10.281 | 38.845 | 3.0156 | -87.40278 | 2.26204 | 1 |
| 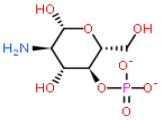 | 0 | 2567 | C6H14NO8P | -20.9 | 28 | 7 | -12.255 | -2.2146 | -12.79 | 0 | 19.644 | 8.2578 | -87.25436 | 0.88958 | 1 |
| 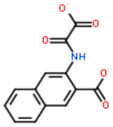 | 0 | 1518 | 761 | -20.89 | 26 | 2 | -8.3544 | -3.0958 | -18.35 | 0 | 14.933 | 12.525 | -96.92753 | 1.37723 | 1 |
| 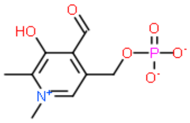 | 0 | 1431 | C9H13NO6P | -20.88 | 28 | 3 | -8.7039 | -3.2425 | -13.64 | 1.0523 | 13.865 | 6.2488 | -74.60687 | 1.02109 | 1 |
| 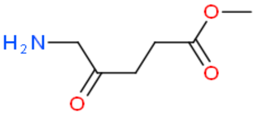 | 0 | 869 | 992 | -20.87 | 21 | 5 | -12.124 | -2.8017 | -11.42 | 1.41 | 16.841 | 10.029 | -59.02001 | 0.82224 | 1 |
| 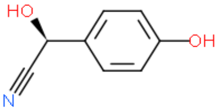 | 0 | 3098 | C8H7NO2 | -20.87 | 18 | 2 | -9.4741 | -2.6499 | -11.38 | 0.6526 | 11.96 | 8.6298 | -58.79726 | 0.60074 | 1 |
| 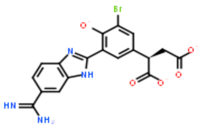 | 0 | 1822 | C18H13BrN4O5 | -20.84 | 40 | 4 | -15.775 | -4.0829 | -24.99 | 15.554 | 23.917 | 25.947 | -133.1499 | 1.46658 | 1 |

| | | | | | | | | | | | | | | | |
|---|---|---|---|---|---|---|---|---|---|---|---|---|---|---|---|
| 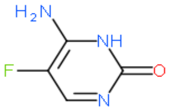 | 0 | 971 | 1099 | -20.83 | 13 | 0 | -8.8907 | -1.6125 | -13.66 | 0.0373 | 12.349 | 10.78 | -42.1699 | 0.43271 | 1 |
| 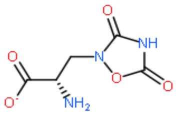 | 0 | 2694 | C5H7N3O5 | -20.82 | 19 | 4 | -9.996 | -1.4526 | -16.3 | 2.2099 | 15.587 | 9.58 | -64.35275 | 0.78842 | 1 |
| 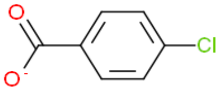 | 0 | 3369 | C7H5ClO2 | -20.81 | 14 | 1 | -8.2444 | -2.7425 | -12.87 | 0.5053 | 10.058 | 9.5624 | -46.73835 | 0.66566 | 1 |
| 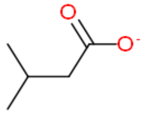 | 0 | 3390 | C5H10O2 | -20.8 | 16 | 2 | -9.2038 | -2.3925 | -8.195 | 0.399 | 9.8808 | 5.2025 | -39.08558 | 0.50658 | 1 |
| 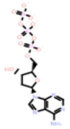 | 0 | 2903 | C10H16N5O12P3 | -20.79 | 42 | 9 | -15.487 | -3.1861 | -24.43 | 0 | 22.727 | 34.108 | -108.6646 | 1.35918 | 1 |
| 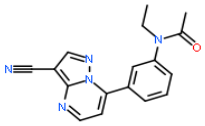 | 0 | 840 | 962 | -20.77 | 38 | 3 | -6.599 | -4.8476 | -28 | 3.937 | 15.899 | 16.797 | -99.18113 | 1.40057 | 1 |
| 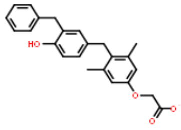 | 0 | 3425 | C24H24O4 | -20.74 | 51 | 6 | -9.7049 | -5.965 | -25.1 | 4.3786 | 21.225 | 16.301 | -140.424 | 1.74715 | 1 |
| 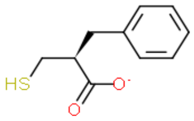 | 0 | 2649 | BTP | -20.73 | 24 | 5 | -8.6749 | -3.2011 | -17.08 | 3.3209 | 11.896 | 9.8769 | -66.13258 | 0.85169 | 1 |
| 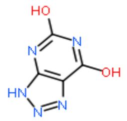 | 0 | 1648 | C4H3N5O2 | -20.73 | 14 | 0 | -10.831 | -1.3726 | -15.18 | 0 | 18.197 | 13.823 | -38.08892 | 0.53493 | 1 |
| 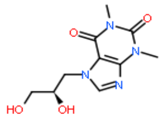 | 0 | 538 | 651 | -20.7 | 32 | 5 | -12.895 | -3.5789 | -16.58 | 5.6745 | 19.913 | 14.481 | -126.8544 | 1.31258 | 1 |
| 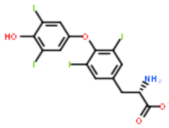 | 0 | 1379 | 1583 | -20.69 | 34 | 6 | -11.183 | -6.0246 | -21.26 | 3.2537 | 23.021 | 15.263 | -101.6848 | 1.678 | 1 |

| | | | | | | | | | | | | | | | |
|---|---|---|---|---|---|---|---|---|---|---|---|---|---|---|---|
| 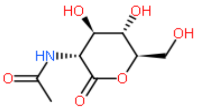 | 0 | 2518 | C8H13NO6 | -20.69 | 28 | 5 | -10.788 | -2.8211 | -16.91 | 1.4366 | 17.569 | 12.664 | -93.00407 | 0.92553 | 1 |
| 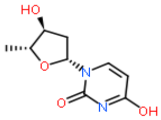 | 0 | 2952 | C9H12N2O4 | -20.68 | 27 | 3 | -8.9629 | -2.8653 | -16.87 | 0.8263 | 18.286 | 7.2427 | -70.61507 | 1.07995 | 1 |
| 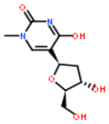 | 0 | 3402 | C10H14N2O5 | -20.67 | 31 | 5 | -10.166 | -2.7082 | -15.06 | 2.239 | 16.875 | 7.4585 | -96.90333 | 1.1281 | 1 |
| 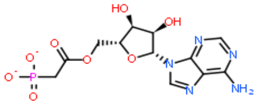 | 0 | 2331 | C12H16N5O8P | -20.67 | 40 | 7 | -14.673 | -4.0556 | -24.59 | 10.596 | 27.108 | 19.512 | -95.19901 | 1.31318 | 1 |
| 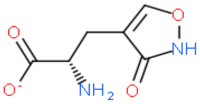 | 0 | 1453 | C6H8N2O4 | -20.66 | 19 | 4 | -10.79 | -1.3781 | -15.58 | 1.3892 | 17.407 | 10.377 | -65.90138 | 0.64442 | 1 |
| 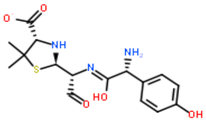 | 0 | 3306 | C16H21N3O5S | -20.59 | 45 | 8 | -13.5 | -4.3127 | -24.96 | 4.7118 | 25.707 | 21.987 | -151.9103 | 1.62471 | 1 |
| 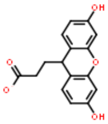 | 0 | 1436 | HXP | -20.57 | 34 | 3 | -10.491 | -3.3036 | -16.94 | 1.8448 | 18.605 | 12.508 | -66.42407 | 0.8778 | 1 |
| 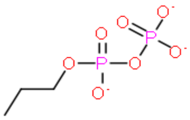 | 0 | 1468 | C3H10O7P2 | -20.57 | 19 | 5 | -13.32 | -2.3159 | -16.35 | 0.0663 | 16.79 | 23.325 | -80.98817 | 0.86615 | 1 |
| 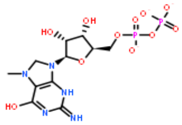 | 0 | 1724 | C11H19N5O11P2 | -20.57 | 45 | 9 | -16.272 | -3.3 | -26.43 | 0.4964 | 29.72 | 32.197 | -112.9994 | 1.49998 | 1 |
| 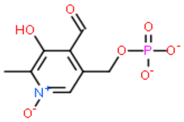 | 0 | 3278 | C8H10NO7P | -20.55 | 25 | 3 | -6.7334 | -1.9858 | -17.21 | 2.3295 | 14.195 | 1.7893 | -35.60396 | 0.90375 | 1 |
| 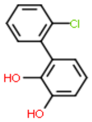 | 0 | 1691 | BP3 | -20.55 | 24 | 0 | -4.9197 | -3.9943 | -17.18 | 1.3391 | 9.4874 | 5.8072 | -67.50934 | 1.00337 | 1 |

| | | | | | | | | | | | | | | | |
|---|---|---|---|---|---|---|---|---|---|---|---|---|---|---|---|
| 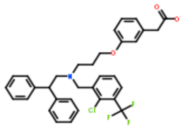 | 0 | 3428 | C33H31ClF3NO3 | -20.53 | 71 | 12 | -9.2969 | -8.3317 | -38.85 | 20.074 | 20.377 | 19.548 | -157.8141 | 1.88825 | 1 |
| 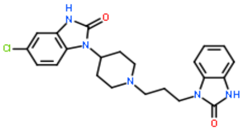 | 0 | 1054 | 1184 | -20.5 | 54 | 5 | -9.765 | -5.782 | -26.88 | 2.7968 | 21.331 | 21.639 | -122.6877 | 1.72901 | 1 |
| 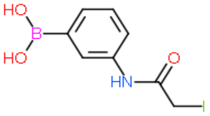 | 0 | 1879 | IAP | -20.49 | 23 | 3 | -10.519 | -3.3193 | -14.25 | 1.7246 | 17.154 | 10.45 | -51.1891 | 1.06826 | 1 |
| 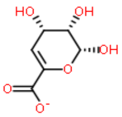 | 0 | 2448 | C6H8O6 | -20.49 | 19 | 4 | -10.486 | -1.6961 | -14.06 | 1.0067 | 16.268 | 9.1335 | -82.46667 | 0.71117 | 1 |
| 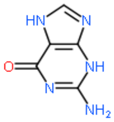 | 0 | 2116 | C5H5N5O | -20.49 | 16 | 0 | -8.1026 | -1.5725 | -15.38 | 0 | 14.935 | 7.9498 | -49.0722 | 0.6061 | 1 |
| 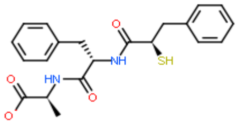 | 0 | 2286 | OIR | -20.49 | 51 | 10 | -11.674 | -4.9935 | -29.57 | 8.5535 | 22.614 | 21.018 | -140.3936 | 1.65357 | 1 |
| 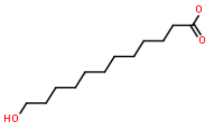 | 0 | 3347 | 12H | -20.48 | 38 | 12 | -12.574 | -4.5203 | -19.7 | 9.2722 | 16.27 | 13.778 | -106.1711 | 1.2444 | 1 |
| 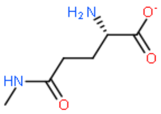 | 0 | 3137 | C6H12N2O3 | -20.48 | 22 | 5 | -10.144 | -2.5513 | -13.47 | 1.0365 | 15.129 | 8.1823 | -47.95802 | 0.77769 | 1 |
| 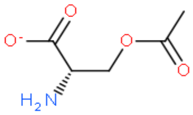 | 0 | 1614 | Untitled | -20.47 | 18 | 4 | -10.627 | -2.1414 | -13.1 | 2.9664 | 15.719 | 7.56 | -55.70586 | 0.70105 | 1 |
| 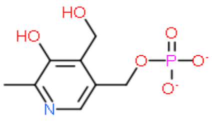 | 0 | 1958 | C8H12NO6P | -20.46 | 26 | 5 | -9.9912 | -3.2139 | -17.1 | 0 | 17.169 | 12.422 | -72.5455 | 1.07587 | 1 |
| 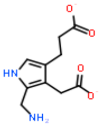 | 0 | 2016 | Pregnenolone.mol | -20.45 | 28 | 7 | -11.988 | -2.5624 | -14.17 | 3.8244 | 19.625 | 6.9021 | -80.31921 | 1.13654 | 1 |

| | | | | | | | | | | | | | | | |
|---|---|---|---|---|---|---|---|---|---|---|---|---|---|---|---|
| 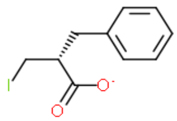 | 0 | 3108 | BIP | -20.45 | 23 | 4 | -8.8519 | -4.1072 | -15.07 | 0 | 12.145 | 12.519 | -77.15028 | 1.10344 | 1 |
| 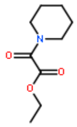 | 0 | 2155 | E1P | -20.44 | 28 | 3 | -9.8791 | -3.8843 | -15.15 | 1.7051 | 13.699 | 13.956 | -95.22625 | 1.11639 | 1 |
| 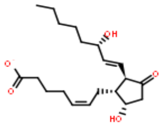 | 0 | 1815 | PG2 | -20.44 | 56 | 14 | -14.646 | -5.1653 | -21.71 | 13.491 | 23.151 | 12.014 | -102.9774 | 1.45436 | 1 |
| 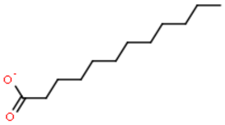 | 0 | 2711 | C12H24O2 | -20.44 | 37 | 10 | -8.4344 | -4.5276 | -16.12 | 3.0393 | 13.395 | 3.3069 | -89.37132 | 1.18456 | 1 |
| 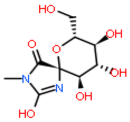 | 0 | 3143 | C9H14N2O7 | -20.44 | 32 | 6 | -11.275 | -2.8713 | -16.16 | 4.4693 | 17.998 | 9.6892 | -82.00172 | 1.09566 | 1 |
| 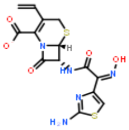 | 0 | 422 | 535 | -20.44 | 38 | 4 | -10.491 | -4.7111 | -26.99 | 3.2622 | 24.726 | 20.224 | -119.5825 | 1.52441 | 1 |
| 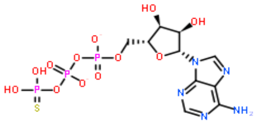 | 0 | 2628 | C10H16N5O12P3S | -20.43 | 45 | 12 | -15.484 | -3.4869 | -28.74 | 0 | 34.098 | 25.819 | -154.9859 | 1.77688 | 1 |
| 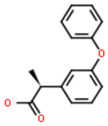 | 0 | 460 | 573 | -20.43 | 31 | 4 | -8.7569 | -5.0507 | -18.26 | 2.1803 | 12.859 | 15.175 | -118.1018 | 1.25114 | 1 |
| 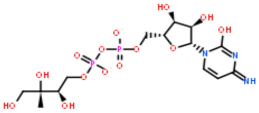 | 0 | 3331 | C14H25N3O14P2 | -20.42 | 56 | 17 | -17.023 | -3.0488 | -26.62 | 0 | 33.802 | 23.874 | -149.3222 | 2.10443 | 1 |
| 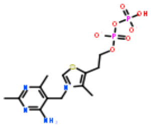 | 0 | 1470 | C13H20N4O7P2S | -20.42 | 46 | 9 | -11.808 | -4.8157 | -23.47 | 5.4847 | 22.768 | 15.746 | -84.12302 | 1.3419 | 1 |
| 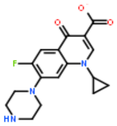 | 0 | 424 | 537 | -20.41 | 41 | 2 | -7.6265 | -4.456 | -19.56 | 3.8885 | 16.306 | 8.5859 | -108.8763 | 1.32585 | 1 |

| | | | | | | | | | | | | | | | |
|---|---|---|---|---|---|---|---|---|---|---|---|---|---|---|---|
| 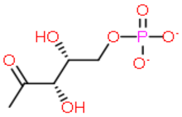 | 0 | 2226 | C5H11O7P | -20.41 | 22 | 7 | -10.569 | -2.3782 | -13.85 | 0 | 13.111 | 11.739 | -72.10709 | 0.70879 | 1 |
| 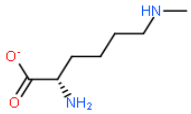 | 0 | 1500 | C7H16N2O2 | -20.4 | 26 | 7 | -11.366 | -2.9673 | -14.6 | 0.0999 | 17.495 | 11.455 | -66.22168 | 0.99883 | 1 |
| 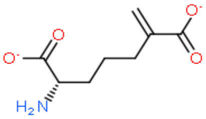 | 0 | 2592 | C8H13NO4 | -20.39 | 24 | 7 | -12.194 | -2.4224 | -15.66 | 2.6404 | 16.793 | 14.187 | -74.01804 | 1.08415 | 1 |
| 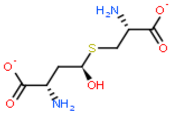 | 0 | 3165 | C7H14N2O5S | -20.38 | 27 | 10 | -15.142 | -1.8471 | -14.65 | 0 | 17.819 | 21.899 | -73.45573 | 1.17472 | 1 |
| 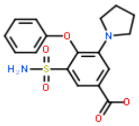 | 0 | 2624 | C17H18N2O5S | -20.38 | 42 | 5 | -7.8015 | -2.8128 | -20.88 | 0.7004 | 12.806 | 13.779 | -65.97842 | 0.86565 | 1 |
| 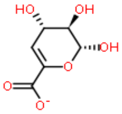 | 0 | 2047 | C6H8O6 | -20.37 | 19 | 4 | -13.298 | -1.5204 | -12.47 | 4.3199 | 19.546 | 10.78 | -61.52571 | 0.64503 | 1 |
| 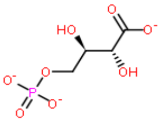 | 0 | 2795 | C4H9O8P | -20.32 | 19 | 7 | -13.066 | -1.4188 | -11.34 | 0 | 15.889 | 13.499 | -72.42327 | 1.03823 | 1 |
| 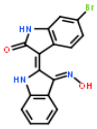 | 0 | 3111 | BRW | -20.31 | 32 | 1 | -7.3092 | -4.7791 | -24.6 | 0.7134 | 19.48 | 15.669 | -117.1442 | 1.77395 | 1 |
| 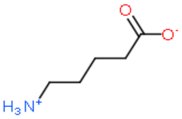 | 0 | 1826 | C5H12NO2 | -20.3 | 19 | 4 | -8.4725 | -2.2472 | -11.16 | 0.1601 | 11.296 | 4.2933 | -49.47385 | 0.59541 | 1 |
| 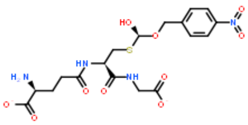 | 0 | 2814 | C18H24N4O10S | -20.29 | 55 | 17 | -15.778 | -4.9253 | -28.79 | 10.701 | 29.07 | 20.138 | -108.7878 | 1.73505 | 1 |
| 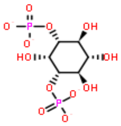 | 0 | 2639 | C6H14O12P2 | -20.29 | 30 | 8 | -8.3141 | -1.0753 | -14.17 | 0 | 19.271 | -5.489 | -27.55031 | 1.06704 | 1 |

| | | | | | | | | | | | | | | | |
|---|---|---|---|---|---|---|---|---|---|---|---|---|---|---|---|
| 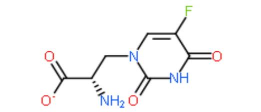 | 0 | 2662 | C7H8FN3O4 | -20.28 | 22 | 4 | -10.194 | -1.7662 | -15.57 | 1.0305 | 18.325 | 8.3358 | -70.87253 | 0.75314 | 1 |
| 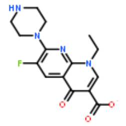 | 0 | 357 | 467 | -20.27 | 39 | 2 | -7.4187 | -4.3323 | -19.37 | 2.5549 | 16.001 | 9.1395 | -105.9087 | 1.27858 | 1 |
| 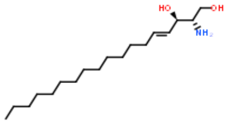 | 0 | 2884 | C18H37NO2 | -20.27 | 58 | 18 | -9.6862 | -6.3616 | -25.78 | 6.8743 | 14.142 | 13.255 | -113.937 | 1.25341 | 1 |
| 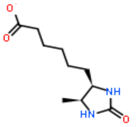 | 0 | 3414 | DTB | -20.26 | 32 | 6 | -12.792 | -3.3273 | -18.22 | 2.5669 | 18.02 | 21.056 | -87.8363 | 1.14799 | 1 |
| 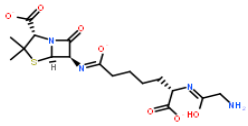 | 0 | 3455 | C17H25N4O7S | -20.24 | 52 | 12 | -12.906 | -4.6737 | -14.49 | 3.256 | 24.077 | 4.2731 | -107.4164 | 1.66219 | 1 |
| 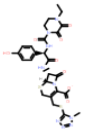 | 0 | 1163 | C25H27N9O8S2 | -20.24 | 70 | 10 | -14.008 | -5.5825 | -35.92 | 12.169 | 33.564 | 24.838 | -135.691 | 2.32207 | 1 |
| 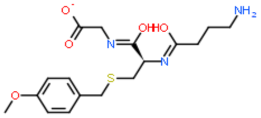 | 0 | 2640 | C17H25N3O5S | -20.22 | 50 | 14 | -14.565 | -4.7068 | -22.24 | 5.5219 | 26.495 | 15.808 | -120.2364 | 1.78254 | 1 |
| 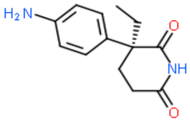 | 0 | 250 | 357 | -20.22 | 33 | 2 | -8.1667 | -3.7916 | -17.87 | 2.5008 | 13.627 | 12.028 | -94.37904 | 0.995 | 1 |
| 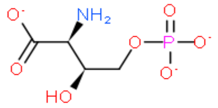 | 0 | 2333 | C4H10NO7P | -20.21 | 20 | 7 | -11.11 | -1.5138 | -12.37 | 0 | 13.347 | 10.724 | -61.59463 | 0.81748 | 1 |
| 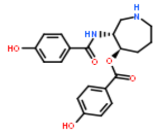 | 0 | 2335 | B1L | -20.19 | 49 | 2 | -7.962 | -5.918 | -28.98 | 4.8849 | 22.172 | 18.397 | -108.415 | 1.60379 | 1 |
| 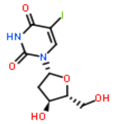 | 0 | 144 | C9H11IN2O5 | -20.17 | 28 | 4 | -10.575 | -3.0993 | -15.79 | 0 | 19.351 | 11.422 | -71.58276 | 1.38988 | 1 |

| | | | | | | | | | | | | | | | |
|---|---|---|---|---|---|---|---|---|---|---|---|---|---|---|---|
| 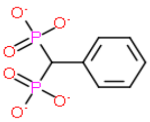 | 0 | 2608 | C7H10O6P2 | -20.15 | 21 | 3 | -14.734 | -2.3294 | -15.21 | 0 | 16.979 | 29.334 | -69.64883 | 0.68935 | 1 |
| 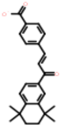 | 0 | 2199 | C24H26O3 | -20.13 | 52 | 2 | -7.3733 | -5.762 | -24.88 | 4.8898 | 18.478 | 14.059 | -113.4264 | 1.7839 | 1 |
| 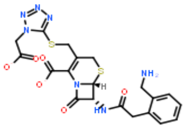 | 0 | 802 | 923 | -20.12 | 54 | 10 | -12.517 | -5.1566 | -29.41 | 3.6048 | 29.608 | 21.27 | -138.1256 | 2.16458 | 1 |
| 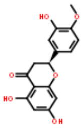 | 0 | 966 | 1094 | -20.11 | 36 | 1 | -8.9231 | -4.5836 | -22.8 | 0 | 20.259 | 18.996 | -132.0817 | 1.46937 | 1 |
| 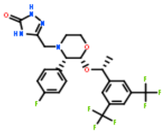 | 0 | 559 | C23H21F7N4O3 | -20.11 | 58 | 6 | -8.6297 | -6.6647 | -30.48 | 7.5971 | 19.105 | 21.49 | -167.4087 | 1.68729 | 1 |
| 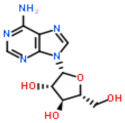 | 0 | 89 | 32326 | -20.11 | 32 | 5 | -11.1 | -3.7047 | -15.33 | 0.5336 | 21.7 | 9.3878 | -90.4594 | 1.09228 | 1 |
| 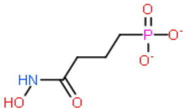 | 0 | 1482 | C4H8NO5P | -20.09 | 19 | 6 | -14.094 | -1.5719 | -10.02 | 0.5569 | 18.526 | 13.36 | -59.80427 | 0.9826 | 1 |
| 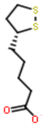 | 0 | 61 | C8H14O2S2 | -20.07 | 25 | 5 | -8.3894 | -3.2558 | -13.21 | 1.1775 | 12.33 | 5.4391 | -68.36469 | 1.0173 | 1 |
| 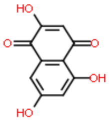 | 0 | 2251 | FLV | -20.07 | 21 | 0 | -6.1789 | -2.2662 | -17.12 | 0 | 11.742 | 8.1716 | -66.17874 | 0.91087 | 1 |
| 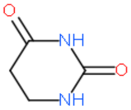 | 0 | 1624 | C4H6N2O2 | -20.05 | 14 | 0 | -7.8983 | -1.5098 | -12.13 | 0 | 11.504 | 6.7255 | -59.04921 | 0.45561 | 1 |
| 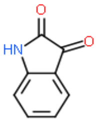 | 0 | 1853 | C8H5NO2 | -20.04 | 16 | 0 | -8.778 | -2.4093 | -12.73 | 0.3668 | 11.463 | 11.665 | -41.14141 | 0.6551 | 1 |

| | | | | | | | | | | | | | | | |
|---|---|---|---|---|---|---|---|---|---|---|---|---|---|---|---|
| 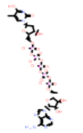 | 0 | 2958 | C20H30N7O23P5 | -20.02 | 80 | 20 | -18.136 | -4.2804 | -38.48 | 4.6717 | 39.531 | 34.492 | -160.3093 | 2.48045 | 1 |
| 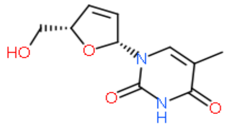 | 0 | 536 | 649 | -20.01 | 28 | 3 | -9.1427 | -2.9542 | -16.34 | 1.1664 | 16.781 | 9.6301 | -73.74787 | 1.25181 | 1 |
| 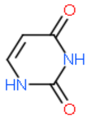 | 0 | 3087 | C4H4N2O2 | -20 | 12 | 0 | -6.9798 | -1.3236 | -14.3 | 0 | 11.668 | 6.1987 | -48.36962 | 0.43412 | 1 |
| 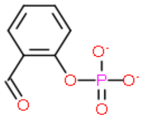 | 0 | 1711 | 262 | -19.98 | 18 | 1 | -9.3512 | -2.0034 | -13.44 | 1.7414 | 14.74 | 8.7243 | -67.20756 | 0.73192 | 1 |
| 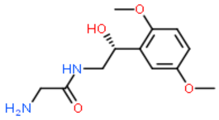 | 0 | 106 | 211 | -19.98 | 36 | 6 | -7.1141 | -3.1875 | -19.54 | 4.1603 | 12.72 | 6.1458 | -43.79525 | 1.14649 | 1 |
| 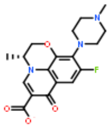 | 0 | 2726 | C18H20FN3O4 | -19.98 | 45 | 1 | -5.8442 | -4.3271 | -19.17 | 0.8937 | 16.533 | 5.057 | -130.0967 | 1.34962 | 1 |
| 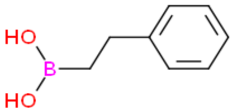 | 0 | 1727 | C8H11BO2 | -19.97 | 22 | 5 | -8.0527 | -3.1763 | -13.21 | 0.3078 | 12.168 | 5.2004 | -45.94817 | 0.65657 | 1 |
| 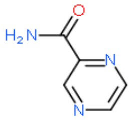 | 0 | 232 | 339 | -19.95 | 14 | 0 | -9.9124 | -2.1212 | -11.87 | 1.2467 | 14.115 | 10.727 | -32.92871 | 0.50301 | 1 |
| 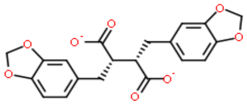 | 0 | 1909 | BDS | -19.94 | 44 | 7 | -13.256 | -4.2087 | -22.51 | 2.9763 | 18.628 | 28.749 | -176.5782 | 1.61534 | 1 |
| 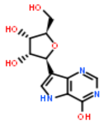 | 0 | 2501 | C11H13N3O5 | -19.93 | 32 | 5 | -10.324 | -3.2119 | -13.26 | 0.2198 | 16.284 | 9.4818 | -112.6085 | 1.03161 | 1 |
| 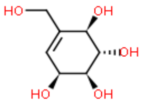 | 0 | 2779 | C7H12O5 | -19.93 | 24 | 6 | -10.73 | -2.3272 | -10.04 | 0.1144 | 12.998 | 8.1968 | -61.98346 | 0.69246 | 1 |

| | | | | | | | | | | | | | | | |
|---|---|---|---|---|---|---|---|---|---|---|---|---|---|---|---|
| 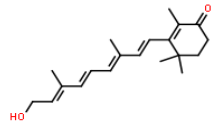 | 0 | 2415 | C20H28O2 | -19.91 | 50 | 2 | -7.0264 | -5.5725 | -23.82 | 0 | 19.395 | 14.645 | -64.03521 | 1.67429 | 1 |
| 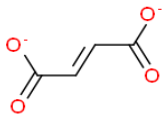 | 0 | 1466 | C4H4O4 | -19.91 | 10 | 2 | -9.3022 | -1.0498 | -10.13 | 0.1993 | 12.562 | 5.6343 | -44.7695 | 0.5188 | 1 |
| 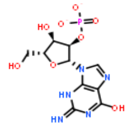 | 0 | 1702 | C10H14N5O8P | -19.91 | 36 | 7 | -14.225 | -2.5069 | -19.58 | 0 | 23.048 | 24.026 | -97.5137 | 1.45061 | 1 |
| 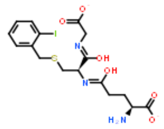 | 0 | 3249 | C17H22IN3O6S | -19.89 | 48 | 15 | -13.775 | -5.1621 | -26.52 | 3.8571 | 22.848 | 24.985 | -105.1809 | 1.45439 | 1 |
| 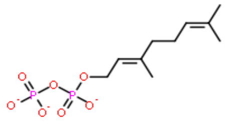 | 0 | 2280 | GPP | -19.86 | 36 | 8 | -13.568 | -3.7293 | -23.01 | 2.0125 | 20.483 | 28.183 | -86.20018 | 1.3532 | 1 |
| 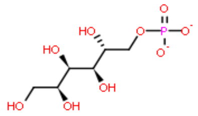 | 0 | 2276 | m | -19.86 | 29 | 12 | -12.147 | -1.8096 | -15.18 | 0 | 14.393 | 14.045 | -83.42932 | 1.06341 | 1 |
| 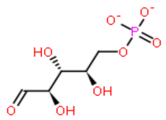 | 0 | 1812 | C5H11O8P | -19.84 | 23 | 9 | -14.268 | -1.2536 | -13.12 | 0.4851 | 19.186 | 15.356 | -75.79107 | 1.06596 | 1 |
| 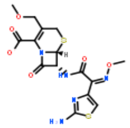 | 0 | 1224 | 1416 | -19.84 | 44 | 5 | -10.861 | -5.4265 | -23.76 | 2.8544 | 25.399 | 17.082 | -106.4373 | 1.69793 | 1 |
| 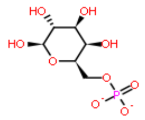 | 0 | 2054 | C6H13O9P | -19.83 | 27 | 7 | -14.591 | -1.7609 | -10.11 | 0 | 22.617 | 10.935 | -72.75678 | 0.96559 | 1 |
| 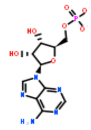 | 0 | 27 | 131 | -19.81 | 35 | 6 | -13.094 | -3.8266 | -16.33 | 0 | 21.772 | 18.607 | -124.6754 | 1.27008 | 1 |
| 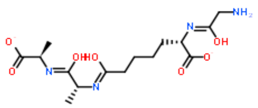 | 0 | 2304 | C15H26N4O7 | -19.81 | 50 | 16 | -16.253 | -4.7816 | -21.06 | 4.6269 | 27.912 | 18.583 | -91.91636 | 1.61779 | 1 |

| | | | | | | | | | | | | | | | |
|---|---|---|---|---|---|---|---|---|---|---|---|---|---|---|---|
| 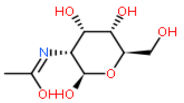 | 0 | 2796 | C8H15NO6 | -19.81 | 30 | 7 | -12.392 | -2.8849 | -14.7 | 4.2508 | 18.985 | 10.899 | -88.55423 | 1.0434 | 1 |
| 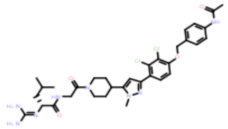 | 0 | 2283 | C33H42Cl2N8O4 | -19.81 | 89 | 10 | -5.0182 | -10.431 | -44.69 | 10.043 | 23.374 | 22.746 | -168.8156 | 2.27182 | 1 |
| 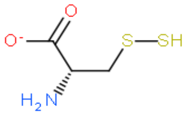 | 0 | 2472 | C3H7NO2S2 | -19.8 | 14 | 5 | -9.5501 | -1.4052 | -11.86 | 0.8543 | 16.874 | 1.5166 | -31.78821 | 0.62595 | 1 |
| 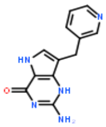 | 0 | 2295 | C12H11N5O | -19.79 | 29 | 2 | -9.8041 | -3.5271 | -14.95 | 2.101 | 16.497 | 11.342 | -68.94802 | 1.04632 | 1 |
| 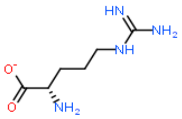 | 0 | 21 | C6H14N4O2 | -19.79 | 25 | 7 | -12.317 | -1.8893 | -12.26 | 4.2947 | 20.049 | 4.6709 | -59.77001 | 0.89562 | 1 |
| 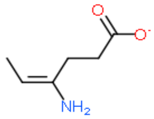 | 0 | 1485 | C6H11NO2 | -19.79 | 19 | 3 | -8.4493 | -2.6847 | -12.07 | 1.2822 | 13.007 | 4.7398 | -60.58243 | 0.62708 | 1 |
| 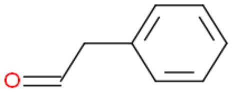 | 0 | 1931 | C8H8O | -19.78 | 17 | 2 | -6.7895 | -2.7155 | -12.5 | 0.8528 | 8.8605 | 5.0897 | -69.72022 | 0.72627 | 1 |
| 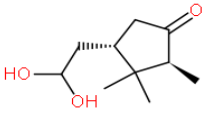 | 0 | 2606 | CAX | -19.78 | 31 | 4 | -10.781 | -3.5224 | -11.47 | 2.8902 | 14.894 | 9.1219 | -61.00501 | 0.88035 | 1 |
| 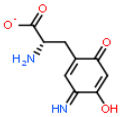 | 0 | 3280 | C9H10N2O4 | -19.77 | 24 | 4 | -10.113 | -1.9458 | -15.99 | 4.9285 | 17.706 | 6.789 | -80.16902 | 0.93592 | 1 |
| 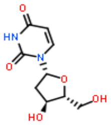 | 0 | 2000 | C9H12N2O5 | -19.76 | 28 | 4 | -10.479 | -2.4322 | -14.53 | 0 | 18.603 | 9.9414 | -78.93805 | 1.23824 | 1 |
| 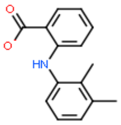 | 0 | 666 | 784 | -19.74 | 32 | 1 | -7.9615 | -4.7277 | -17.27 | 1.0591 | 11.864 | 16.229 | -102.3968 | 1.31573 | 1 |

| | | | | | | | | | | | | | | | |
|---|---|---|---|---|---|---|---|---|---|---|---|---|---|---|---|
| 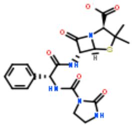 | 0 | 933 | C20H23N5O6S | -19.72 | 54 | 7 | -11.559 | -5.725 | -27.21 | 15.86 | 22.979 | 14.573 | -148.5633 | 1.52748 | 1 |
| 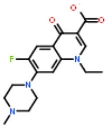 | 0 | 377 | 487 | -19.69 | 43 | 2 | -6.571 | -4.4164 | -17.86 | 0.3467 | 15.633 | 6.9903 | -115.5839 | 1.35165 | 1 |
| 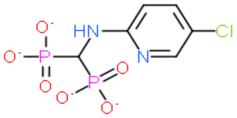 | 0 | 3298 | C6H9ClN2O6P2 | -19.69 | 22 | 3 | -14.162 | -2.6447 | -17.45 | 0 | 19.704 | 28.463 | -76.97197 | 0.97415 | 1 |
| 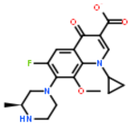 | 0 | 918 | 1044 | -19.67 | 48 | 2 | -6.2104 | -4.753 | -21.52 | 2.5352 | 17.999 | 6.8528 | -125.3542 | 1.50445 | 1 |
| 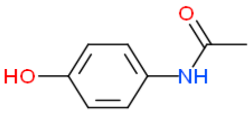 | 0 | 210 | 316 | -19.67 | 20 | 0 | -7.3349 | -3.0819 | -13.99 | 0.4953 | 11.642 | 8.9631 | -66.56789 | 0.89642 | 1 |
| 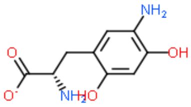 | 0 | 2626 | C9H12N2O4 | -19.65 | 26 | 4 | -10.206 | -2.3886 | -17.69 | 0.8242 | 19.943 | 11.533 | -75.01788 | 0.90729 | 1 |
| 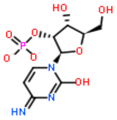 | 0 | 3404 | C9H14N3O8P | -19.65 | 33 | 7 | -11.945 | -2.4172 | -15.3 | 0 | 18.272 | 14.523 | -98.81905 | 1.15328 | 1 |
| 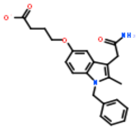 | 0 | 2634 | 6IN | -19.65 | 51 | 8 | -8.7999 | -5.3763 | -25.54 | 7.0145 | 18.563 | 13.37 | -74.73958 | 1.20431 | 1 |
| 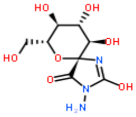 | 0 | 1620 | C8H13N3O7 | -19.61 | 31 | 7 | -13.169 | -2.2426 | -10.56 | 0.3291 | 21.854 | 7.5343 | -81.27948 | 1.09137 | 1 |
| 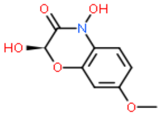 | 0 | 1938 | HBO | -19.61 | 24 | 2 | -9.1933 | -2.9921 | -15.68 | 0.3345 | 14.944 | 13.217 | -93.17179 | 1.07952 | 1 |
| 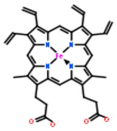 | 0 | 1951 | C36H32FeN4O4 | -19.57 | 75 | 6 | -6.81 | -7.7031 | -30.79 | 0.5619 | 24.338 | 17.296 | -158.4373 | 2.13753 | 1 |

| | | | | | | | | | | | | | | | |
|---|---|---|---|---|---|---|---|---|---|---|---|---|---|---|---|
| 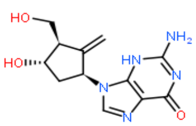 | 0 | 332 | 442 | -19.57 | 35 | 4 | -8.6705 | -2.8182 | -17.52 | 2.3876 | 19.927 | 4.537 | -28.63529 | 1.00432 | 1 |
| 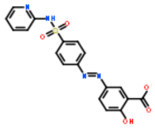 | 0 | 676 | m | -19.57 | 41 | 4 | -3.8935 | -4.9626 | -29.48 | 0 | 18.161 | 10.709 | -115.0982 | 1.61698 | 1 |
| 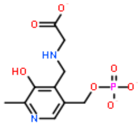 | 0 | 2528 | C10H15N2O7P | -19.54 | 32 | 7 | -10.015 | -3.5838 | -12.61 | 0 | 17.191 | 5.6248 | -108.4771 | 1.39229 | 1 |
| 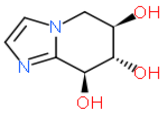 | 0 | 2113 | C7H10N2O3 | -19.53 | 22 | 3 | -8.917 | -2.7329 | -13.43 | 0.2592 | 13.852 | 9.0096 | -74.04508 | 0.68755 | 1 |
| 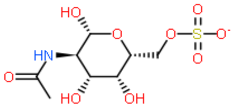 | 0 | 1939 | C8H15NO9S | -19.51 | 33 | 7 | -13.645 | -2.6246 | -15.37 | 0 | 23.886 | 15.187 | -84.55792 | 1.24841 | 1 |
| 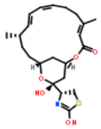 | 0 | 2344 | C22H31NO5S | -19.5 | 60 | 3 | -9.0742 | -3.5074 | -18.77 | 0 | 17.274 | 14.873 | -46.2158 | 1.14994 | 1 |
| 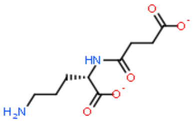 | 0 | 3234 | C9H16N2O5 | -19.5 | 30 | 9 | -10.727 | -2.8604 | -18 | 6.7269 | 19.714 | 5.048 | -100.5445 | 1.22813 | 1 |
| 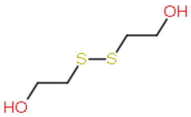 | 0 | 2217 | C4H10O2S2 | -19.49 | 18 | 7 | -11.485 | -2.4803 | -12.96 | 4.7766 | 16.285 | 7.4383 | -34.07313 | 0.72876 | 1 |
| 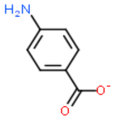 | 0 | 2101 | C7H7NO2 | -19.46 | 16 | 1 | -8.3606 | -2.3708 | -12.7 | 0.4541 | 11.804 | 9.4893 | -44.49191 | 0.52505 | 1 |
| 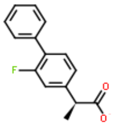 | 0 | 596 | 712 | -19.45 | 30 | 3 | -8.7757 | -4.426 | -18.69 | 4.068 | 14.668 | 13.881 | -132.6719 | 1.22246 | 1 |
| 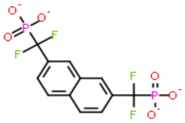 | 0 | 2836 | FNP | -19.44 | 30 | 4 | -9.3992 | -3.0791 | -15.19 | 0 | 16.564 | 10.26 | -111.5314 | 1.34399 | 1 |

| | | | | | | | | | | | | | | | | |
|---|---|---|---|---|---|---|---|---|---|---|---|---|---|---|---|---|
| 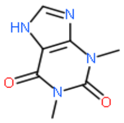 | 0 | 1093 | 1223 | -19.43 | 21 | 0 | -7.1884 | -3.1372 | -13.76 | 0.3108 | 12.065 | 8.1561 | -54.90244 | 0.95919 | 1 |
| 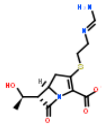 | 0 | 1391 | 1598 | -19.42 | 36 | 6 | -12.76 | -3.5466 | -16.7 | 3.8774 | 22.674 | 13.641 | -95.07436 | 1.68373 | 1 |
| 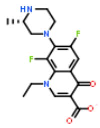 | 0 | 855 | 978 | -19.42 | 43 | 2 | -6.927 | -4.6408 | -19.61 | 2.5923 | 16.258 | 8.894 | -124.0061 | 1.37588 | 1 |
| 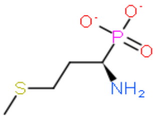 | 0 | 1906 | C4H12NO3PS | -19.41 | 20 | 5 | -11.173 | -2.5492 | -10.72 | 0 | 15.646 | 9.9163 | -58.3806 | 0.64153 | 1 |
| 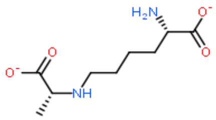 | 0 | 2109 | C9H18N2O4 | -19.39 | 31 | 9 | -15.183 | -3.0068 | -17.24 | 6.7252 | 21.027 | 19.812 | -79.44712 | 1.18109 | 1 |
| 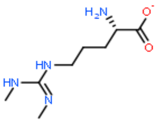 | 0 | 2044 | C8H18N4O2 | -19.39 | 31 | 8 | -13.021 | -3.89 | -18.55 | 4.8154 | 18.562 | 19.851 | -58.97775 | 1.13837 | 1 |
| 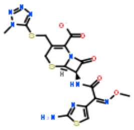 | 0 | 162 | C16H17N9O5S3 | -19.38 | 49 | 5 | -12.547 | -6.339 | -27.32 | 4.8723 | 30.463 | 22.957 | -130.3297 | 1.90568 | 1 |
| 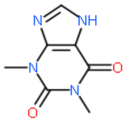 | 0 | 172 | C7H8N4O2 | -19.37 | 21 | 0 | -7.2857 | -3.1292 | -13.83 | 1.0412 | 12.15 | 7.9679 | -58.25063 | 0.96833 | 1 |
| 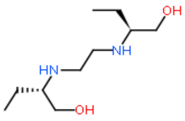 | 0 | 223 | C10H24N2O2 | -19.36 | 38 | 11 | -11.956 | -4.5732 | -14.82 | 4.9418 | 17.903 | 8.7516 | -71.4055 | 1.215 | 1 |
| 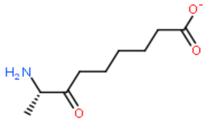 | 0 | 2018 | C9H17NO3 | -19.35 | 29 | 8 | -10.622 | -3.0124 | -12.57 | 1.9228 | 17.469 | 4.6323 | -65.95433 | 1.13198 | 1 |
| 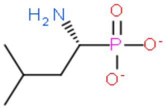 | 0 | 2125 | C5H14NO3P | -19.34 | 22 | 4 | -10.66 | -2.4508 | -12.39 | 0 | 15.54 | 11.528 | -56.92918 | 0.63476 | 1 |

| | | | | | | | | | | | | | | | |
|---|---|---|---|---|---|---|---|---|---|---|---|---|---|---|---|
| 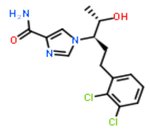 | 0 | 3044 | FR7 | -19.34 | 39 | 6 | -7.0761 | -3.5224 | -24.96 | 4.5633 | 17.697 | 9.5067 | -32.12902 | 0.98552 | 1 |
| 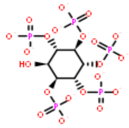 | 0 | 3019 | C6H17O21P5 | -19.32 | 39 | 11 | -10.071 | -1.9445 | -12.1 | 0 | 24.866 | -8.619 | -57.66587 | 1.87035 | 1 |
| 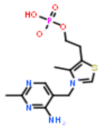 | 0 | 3084 | C12H17N4O4PS | -19.3 | 39 | 7 | -11.679 | -4.8951 | -16.14 | 0 | 19.317 | 16.628 | -91.89632 | 1.49113 | 1 |
| 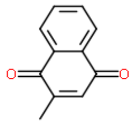 | 0 | 65 | 170 | -19.28 | 21 | 0 | -5.4874 | -3.4049 | -18.1 | 0.6427 | 10.95 | 9.6798 | -80.97279 | 0.99331 | 1 |
| 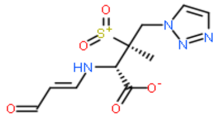 | 0 | 3112 | C10H13N4O5S | -19.28 | 32 | 6 | -10.149 | -2.9754 | -14.61 | 0 | 15.743 | 11.51 | -103.2105 | 1.25095 | 1 |
| 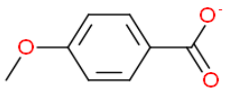 | 0 | 2500 | C8H8O3 | -19.27 | 18 | 2 | -8.0291 | -2.8397 | -14.46 | 0.3952 | 11.86 | 10.726 | -54.69255 | 0.73919 | 1 |
| 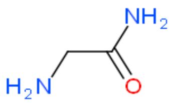 | 0 | 3285 | C2H6N2O | -19.26 | 11 | 2 | -7.243 | -0.5906 | -8.454 | 0 | 8.6652 | 0.3309 | -10.33664 | 0.15512 | 1 |
| 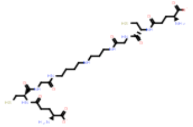 | 0 | 3134 | m | -19.23 | 95 | 31 | -14.657 | -4.99 | -37.84 | 6.9028 | 36.655 | 13.342 | -153.3847 | 3.20013 | 1 |
| 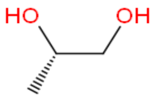 | 0 | 1616 | C3H8O2 | -19.22 | 13 | 3 | -10.978 | -1.7607 | -5.073 | 2.5827 | 11.976 | 3.7422 | -15.25301 | 0.34329 | 1 |
| 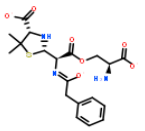 | 0 | 2664 | C19H23N3O7S | -19.2 | 52 | 10 | -14.91 | -5.1444 | -25.06 | 7.8367 | 28.464 | 22.688 | -128.6563 | 1.64774 | 1 |
| 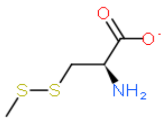 | 0 | 2100 | C4H9NO2S2 | -19.18 | 17 | 5 | -10.066 | -2.1391 | -12.19 | 0.1184 | 16.782 | 6.4352 | -43.14349 | 0.75147 | 1 |

| | | | | | | | | | | | | | | | |
|---|---|---|---|---|---|---|---|---|---|---|---|---|---|---|---|
| 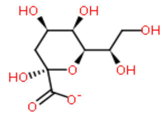 | 0 | 3204 | C8H14O8 | -19.18 | 29 | 8 | -12.071 | -2.306 | -14.98 | 5.1074 | 17.636 | 10.29 | -81.36153 | 0.98106 | 1 |
| 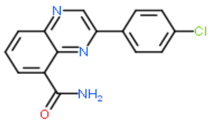 | 0 | 3172 | CNQ | -19.17 | 30 | 1 | -9.6595 | -4.7425 | -19.49 | 3.654 | 17.556 | 18.15 | -101.6613 | 1.4138 | 1 |
| 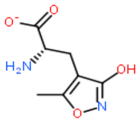 | 0 | 1816 | C7H10N2O4 | -19.17 | 22 | 4 | -10.376 | -2.1443 | -15.64 | 0.1157 | 16.777 | 13.801 | -49.49743 | 0.79971 | 1 |
| 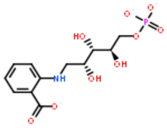 | 0 | 3201 | C12H18NO9P | -19.16 | 38 | 11 | -14.611 | -3.4721 | -17.67 | 0 | 20.151 | 24.369 | -103.1001 | 1.4205 | 1 |
| 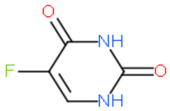 | 0 | 431 | 544 | -19.16 | 12 | 0 | -6.8866 | -1.4861 | -14.69 | 0 | 11.731 | 7.7991 | -53.03699 | 0.43655 | 1 |
| 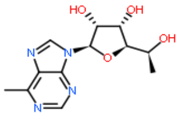 | 0 | 2632 | C12H16N4O4 | -19.15 | 36 | 5 | -10.332 | -4.2943 | -17.02 | 2.0063 | 19.943 | 11.708 | -85.4484 | 1.38453 | 1 |
| 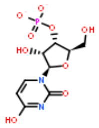 | 0 | 2429 | C9H13N2O9P | -19.14 | 32 | 7 | -10.499 | -2.5122 | -18.26 | 0 | 20.585 | 11.705 | -103.8052 | 1.37535 | 1 |
| 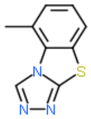 | 0 | 2591 | C9H7N3S | -19.13 | 20 | 0 | -7.1959 | -3.6832 | -14.09 | 0.722 | 13.401 | 7.8007 | -52.62288 | 0.88902 | 1 |
| 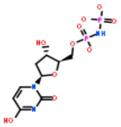 | 0 | 3290 | C9H15N3O10P2 | -19.12 | 36 | 8 | -14.19 | -2.5714 | -20.73 | 0 | 26.706 | 21.816 | -121.5605 | 1.30958 | 1 |
| 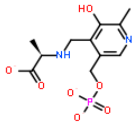 | 0 | 1757 | C11H17N2O7P | -19.12 | 35 | 7 | -9.075 | -2.653 | -17.9 | 0 | 20.588 | 5.8764 | -30.04418 | 1.18052 | 1 |
| 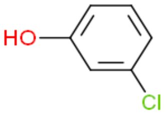 | 0 | 1721 | C6H5ClO | -19.1 | 13 | 0 | -6.0816 | -2.3033 | -12.04 | 0.6805 | 7.397 | 5.9238 | -19.97146 | 0.40566 | 1 |

| | | | | | | | | | | | | | | |
|---|---|---|---|---|---|---|---|---|---|---|---|---|---|---|
| 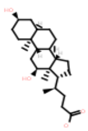 | 0 | 3270 | (3a,5b,12a)-3,12-dihydroxy-Cholan-24-oi | -19.08 | 67 | 6 | -6.7841 | -4.9102 | -18.97 | 2.6024 | 18.73 | 1.7138 | -97.70532 | 1.61355 | 1 |
| 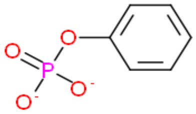 | 0 | 2975 | C6H7O4P | -19.08 | 16 | 2 | -8.5875 | -2.2908 | -13.63 | 0.3139 | 13.437 | 9.6074 | -65.52226 | 0.77288 | 1 |
| 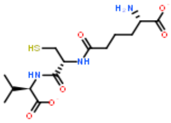 | 0 | 1786 | C14H25N3O6S | -19.06 | 47 | 13 | -10.068 | -2.9556 | -17.83 | 6.7894 | 20.93 | -2.05 | -65.459 | 1.53156 | 1 |
| 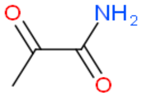 | 0 | 3232 | C3H5NO2 | -19.04 | 11 | 1 | -8.9445 | -1.1953 | -9.818 | 0.5075 | 11.853 | 6.6957 | -33.80448 | 0.36179 | 1 |
| 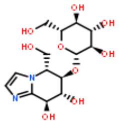 | 0 | 1778 | C14H22N2O9 | -19.03 | 47 | 11 | -12.413 | -3.771 | -12.86 | 0 | 19.893 | 9.4503 | -97.44384 | 1.57527 | 1 |
| 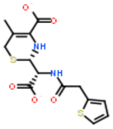 | 0 | 1992 | KCP | -19.02 | 37 | 6 | -10.394 | -4.1596 | -27.77 | 3.4975 | 17.102 | 29.257 | -107.5607 | 1.51904 | 1 |
| 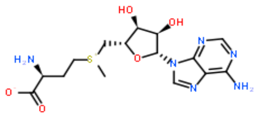 | 0 | 15 | C15H22N6O5S | -19.02 | 49 | 10 | -14.828 | -4.8281 | -17.75 | 0 | 25.96 | 21.233 | -91.02058 | 1.41596 | 1 |
| 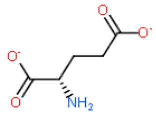 | 0 | 38 | 142 | -19 | 17 | 5 | -12.878 | -1.3914 | -8.223 | 0.5749 | 14.397 | 13.02 | -56.63902 | 0.5961 | 1 |
| 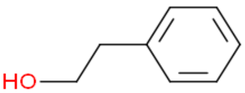 | 0 | 1945 | C8H10O | -19 | 19 | 3 | -6.3007 | -2.7533 | -12.79 | 2.2261 | 6.4744 | 5.4289 | -15.3553 | 0.43004 | 1 |
| 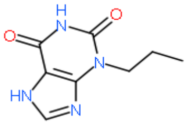 | 0 | 705 | 824 | -18.99 | 24 | 2 | -7.9734 | -2.9391 | -18.58 | 0.4821 | 15.439 | 13.122 | -58.34025 | 0.90361 | 1 |
| 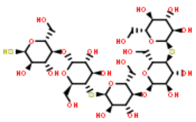 | 0 | 1505 | C30H52O23S3 | -18.97 | 108 | 30 | -24.462 | -5.6487 | -30.62 | 18.614 | 36.873 | 31.056 | -206.1691 | 3.04776 | 1 |

| | | | | | | | | | | | | | | | |
|---|---|---|---|---|---|---|---|---|---|---|---|---|---|---|---|
| 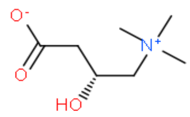 | 0 | 470 | 583 | -18.97 | 26 | 5 | -8.2583 | -3.1468 | -10.94 | 0 | 10.504 | 6.2062 | -54.35584 | 0.84243 | 1 |
| 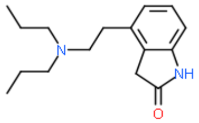 | 0 | 163 | 268 | -18.95 | 43 | 7 | -8.3953 | -5.3233 | -18.81 | 3.436 | 13.185 | 12.898 | -88.49807 | 1.33421 | 1 |
| 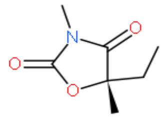 | 0 | 504 | 617 | -18.95 | 22 | 1 | -7.4944 | -3.3535 | -11.59 | 0.4399 | 9.7418 | 8.6016 | -66.66126 | 0.63656 | 1 |
| 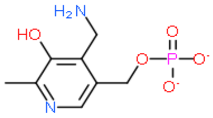 | 0 | 1899 | C8H13N2O5P | -18.94 | 27 | 5 | -10.721 | -3.1516 | -16.12 | 2.7517 | 19.424 | 10.909 | -62.5948 | 1.06113 | 1 |
| 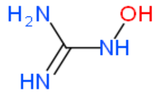 | 0 | 3409 | CH5N3O | -18.94 | 10 | 2 | -7.7336 | -0.1955 | -9.538 | 0.2288 | 8.5677 | 3.8408 | -3.031871 | 0.12622 | 1 |
| 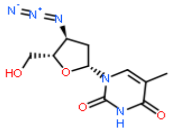 | 0 | 385 | 495 | -18.93 | 32 | 4 | -10.12 | -3.0448 | -17.32 | 0 | 18.847 | 14.394 | -88.03075 | 1.44791 | 1 |
| 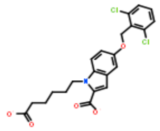 | 0 | 2058 | 669 | -18.92 | 49 | 9 | -10.611 | -6.3323 | -24.61 | 5.1598 | 20.388 | 19.674 | -139.9323 | 2.07377 | 1 |
| 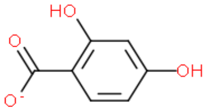 | 0 | 2542 | 2,4-Dihydroxybenzoic | -18.91 | 16 | 1 | -8.48 | -2.0512 | -12.5 | 0.8542 | 13.612 | 7.7147 | -50.48576 | 0.64993 | 1 |
| 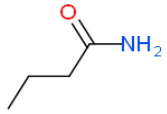 | 0 | 1878 | C4H9NO | -18.91 | 15 | 2 | -8.0002 | -2.0376 | -10.81 | 0.7465 | 9.5421 | 7.1263 | -31.09319 | 0.33546 | 1 |
| 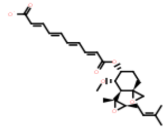 | 0 | 2361 | C26H34O7 | -18.9 | 66 | 6 | -10.045 | -6.0868 | -25.55 | 11.234 | 25.657 | 9.9345 | -67.11275 | 2.02935 | 1 |
| 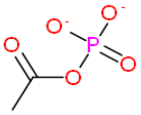 | 0 | 2597 | C2H5O5P | -18.9 | 11 | 1 | -10.477 | -1.3605 | -9.869 | 0.2913 | 14.017 | 10.758 | -48.02717 | 0.51799 | 1 |

| | | | | | | | | | | | | | | | |
|---|---|---|---|---|---|---|---|---|---|---|---|---|---|---|---|
| 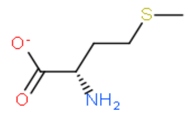 | 0 | 30 | 134 | -18.88 | 19 | 5 | -8.4551 | -2.7563 | -12.97 | 1.2566 | 13.407 | 5.3328 | -49.1008 | 0.76903 | 1 |
| 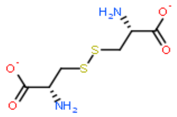 | 0 | 34 | C6H12N2O4S2 | -18.88 | 24 | 9 | -16.626 | -1.7565 | -15.06 | 7.4482 | 23.63 | 17.925 | -58.55512 | 1.08928 | 1 |
| 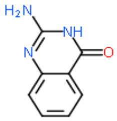 | 0 | 3418 | C8H7N3O | -18.88 | 19 | 0 | -7.3927 | -2.2659 | -15.93 | 0.2343 | 14.512 | 9.4616 | -76.90449 | 0.81109 | 1 |
| 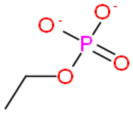 | 0 | 3457 | C2H7O4P | -18.87 | 12 | 2 | -9.2987 | -1.7213 | -10.46 | 0 | 11.914 | 9.2853 | -38.26992 | 0.50772 | 1 |
| 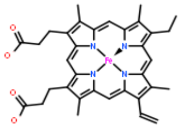 | 0 | 1935 | C34H34FeN4O4 | -18.86 | 75 | 7 | -8.1684 | -7.8028 | -29.66 | 0 | 23.97 | 21.994 | -172.4081 | 2.15896 | 1 |
| 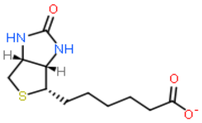 | 0 | 2799 | SHM | -18.86 | 34 | 6 | -9.9478 | -4.0536 | -20.79 | 4.9321 | 17.162 | 15.741 | -98.3152 | 1.2164 | 1 |
| 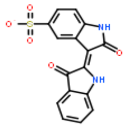 | 0 | 2249 | C16H10N2O5S | -18.84 | 33 | 1 | -6.8754 | -3.2453 | -18.41 | 1.1842 | 16.745 | 7.9961 | -78.56418 | 1.25854 | 1 |
| 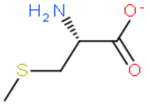 | 0 | 1964 | C4H9NO2S | -18.78 | 16 | 4 | -8.89 | -2.3671 | -11.87 | 2.4653 | 13.483 | 4.8036 | -40.12065 | 0.62127 | 1 |
| 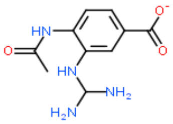 | 0 | 3017 | C10H14N4O3 | -18.77 | 30 | 4 | -7.5649 | -2.8886 | -17.35 | 0 | 20.135 | 3.2637 | -73.6133 | 1.15612 | 1 |
| 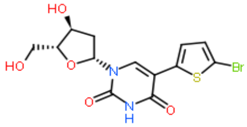 | 0 | 3441 | BTD | -18.77 | 35 | 4 | -8.4888 | -4.4718 | -23.28 | 0 | 18.01 | 19.809 | -121.4249 | 1.69739 | 1 |
| 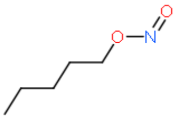 | 0 | 1405 | C5H11NO2 | -18.76 | 19 | 4 | -8.6902 | -2.8713 | -12.12 | 1.0942 | 11.432 | 8.5378 | -40.21679 | 0.70146 | 1 |

| | | | | | | | | | | | | | | | |
|---|---|---|---|---|---|---|---|---|---|---|---|---|---|---|---|
| 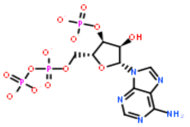 | 0 | 1619 | C10H16N5O13P3 | -18.75 | 42 | 9 | -14.885 | -3.271 | -21.61 | 0 | 25.78 | 27.231 | -120.9981 | 1.43353 | 1 |
| 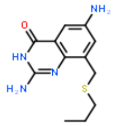 | 0 | 2324 | APQ | -18.73 | 34 | 4 | -10.115 | -3.6468 | -17.97 | 3.564 | 18.847 | 13.032 | -87.65807 | 1.27967 | 1 |
| 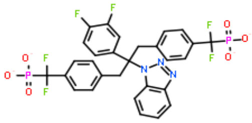 | 0 | 2372 | C29H23F6N3O6P2 | -18.73 | 65 | 10 | -12.907 | -6.8416 | -27.89 | 2.2901 | 29.889 | 25.154 | -192.1712 | 2.14576 | 1 |
| 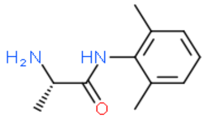 | 0 | 928 | 1056 | -18.73 | 30 | 2 | -4.9253 | -3.7568 | -16.77 | 0.2987 | 10.359 | 5.8628 | -36.74666 | 0.61495 | 1 |
| 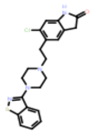 | 0 | 141 | 246 | -18.71 | 49 | 3 | -7.1161 | -6.6657 | -26.98 | 6.7436 | 20.91 | 13.961 | -118.4659 | 1.8203 | 1 |
| 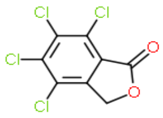 | 0 | 2979 | C8H2Cl4O2 | -18.69 | 16 | 0 | -6.0912 | -3.7235 | -14.76 | 0.6842 | 11.438 | 7.5492 | -57.80158 | 1.06721 | 1 |
| 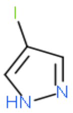 | 0 | 2435 | C3H3IN2 | -18.68 | 9 | 0 | -6.2101 | -2.4843 | -12.51 | 0.0881 | 8.9082 | 6.7699 | -20.2673 | 0.43303 | 1 |
| 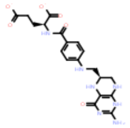 | 0 | 13 | 116 | -18.67 | 53 | 7 | -11.422 | -4.732 | -29.3 | 1.8108 | 26.328 | 26.757 | -144.3587 | 1.80767 | 1 |
| 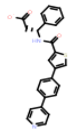 | 0 | 3041 | PF3 | -18.65 | 50 | 4 | -8.0019 | -7.3037 | -27.29 | 6.4809 | 20.903 | 17.9 | -178.1498 | 1.87202 | 1 |
| 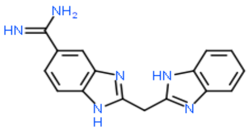 | 0 | 1549 | BAI | -18.63 | 36 | 2 | -8.8307 | -4.6818 | -24.12 | 0 | 20.918 | 21.328 | -106.531 | 1.07586 | 1 |
| 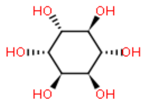 | 0 | 2793 | C6H12O6 | -18.62 | 24 | 6 | -9.9744 | -0.9501 | -10.23 | 4.3813 | 12.96 | 2.3264 | -25.50654 | 0.76565 | 1 |

| | | | | | | | | | | | | | | | |
|---|---|---|---|---|---|---|---|---|---|---|---|---|---|---|---|
| 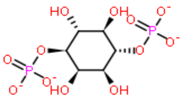 | 0 | 2840 | C6H14O12P2 | -18.62 | 30 | 8 | -12.228 | -1.9412 | -10.81 | 2.5034 | 18.412 | 6.5416 | -101.9144 | 1.17057 | 1 |
| 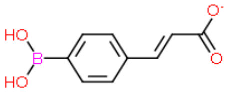 | 0 | 2233 | CVB | -18.61 | 22 | 3 | -7.6002 | -2.7862 | -13.54 | 0.954 | 14.659 | 4.0254 | -49.82019 | 0.91181 | 1 |
| 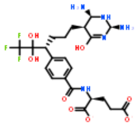 | 0 | 3203 | C22H30F3N5O8 | -18.6 | 66 | 16 | -15.81 | -4.9947 | -32.52 | 7.7438 | 35.811 | 24.477 | -167.2445 | 1.90851 | 1 |
| 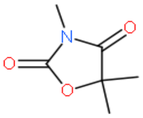 | 0 | 240 | 347 | -18.59 | 19 | 0 | -7.5001 | -2.9842 | -10.7 | 0.5814 | 9.0177 | 9.0407 | -55.98548 | 0.51075 | 1 |
| 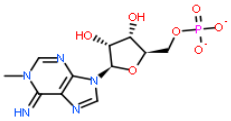 | 0 | 2995 | C11H16N5O7P | -18.58 | 38 | 6 | -14.129 | -3.3877 | -23.8 | 6.777 | 24.244 | 26.353 | -80.29157 | 1.36097 | 1 |
| 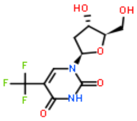 | 0 | 322 | 432 | -18.57 | 31 | 5 | -9.9538 | -3.1524 | -16.03 | 0.3902 | 18.377 | 11.716 | -90.01395 | 1.38462 | 1 |
| 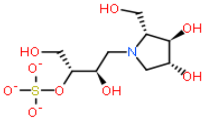 | 0 | 2222 | C9H18NO9S | -18.57 | 38 | 12 | -16.122 | -2.5507 | -17.77 | 13.218 | 24.744 | 12.132 | -100.5285 | 1.19479 | 1 |
| 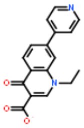 | 0 | 698 | 817 | -18.55 | 35 | 2 | -6.7832 | -4.1059 | -17.73 | 2.6951 | 15.34 | 7.2285 | -115.2778 | 1.20278 | 1 |
| 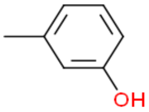 | 0 | 1556 | C7H8O | -18.52 | 16 | 0 | -6.2089 | -2.294 | -11.96 | 0.7684 | 7.3401 | 7.1494 | -18.41265 | 0.40808 | 1 |
| 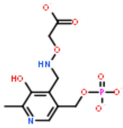 | 0 | 2489 | C10H15N2O8P | -18.51 | 33 | 8 | -9.1178 | -3.0417 | -20.16 | 0 | 22.583 | 7.6035 | -59.5183 | 1.04676 | 1 |
| 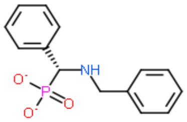 | 0 | 3231 | 2BF | -18.5 | 33 | 5 | -10.437 | -4.0478 | -18.97 | 3.2288 | 15.285 | 19.973 | -92.04534 | 1.13977 | 1 |

| | | | | | | | | | | | | | | | |
|---|---|---|---|---|---|---|---|---|---|---|---|---|---|---|---|
| 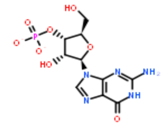 | 0 | 2992 | m | -18.47 | 36 | 6 | -7.9431 | -1.9929 | -17.98 | 0 | 22.702 | 0.4717 | -50.16225 | 1.24175 | 1 |
| 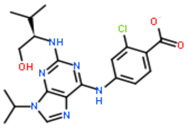 | 0 | 2447 | PVB | -18.46 | 54 | 6 | -7.6269 | -6.6853 | -27.07 | 6.9044 | 20.403 | 14.14 | -107.8351 | 1.5166 | 1 |
| 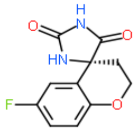 | 0 | 2427 | SBI | -18.46 | 26 | 0 | -5.9223 | -2.3442 | -17.4 | 0.7402 | 13.365 | 7.6032 | -34.63358 | 0.76094 | 1 |
| 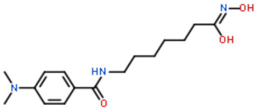 | 0 | 2292 | C16H25N3O3 | -18.42 | 47 | 9 | -9.5606 | -5.4893 | -20.14 | 0.7274 | 16.71 | 16.999 | -72.7318 | 1.1889 | 1 |
| 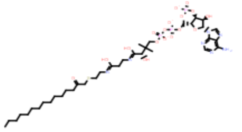 | 0 | 2015 | C36H64N7O17P3S | -18.4 | 124 | 37 | -15.495 | -8.1409 | -40.27 | 7.395 | 38.811 | 16.335 | -206.7685 | 3.33884 | 1 |
| 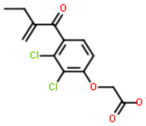 | 0 | 782 | 903 | -18.39 | 30 | 3 | -9.0776 | -4.668 | -20.58 | 4.7427 | 17.438 | 16.002 | -134.934 | 1.46053 | 1 |
| 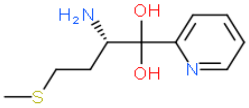 | 0 | 1655 | M2C | -18.37 | 31 | 8 | -10.161 | -4.058 | -18.03 | 2.8395 | 16.11 | 14.379 | -84.1554 | 1.2331 | 1 |
| 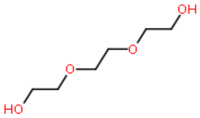 | 0 | 2069 | C6H14O4 | -18.37 | 24 | 9 | -11.745 | -3.3361 | -12.97 | 4.5682 | 15.959 | 9.7535 | -65.65995 | 0.91799 | 1 |
| 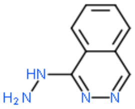 | 0 | 1137 | 1275 | -18.36 | 20 | 1 | -7.2087 | -3.2428 | -14.84 | 0.6779 | 14.109 | 8.0342 | -45.0265 | 0.79516 | 1 |
| 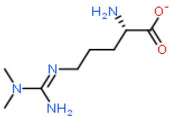 | 0 | 1474 | C8H18N4O2 | -18.36 | 31 | 7 | -11.412 | -3.527 | -17.49 | 0 | 19.671 | 17.288 | -83.21064 | 1.17172 | 1 |
| 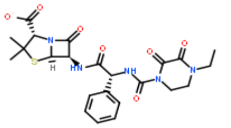 | 0 | 213 | C23H27N5O7S | -18.33 | 62 | 8 | -10.652 | -5.3404 | -25.88 | 6.1438 | 23.577 | 18.036 | -141.0089 | 2.12057 | 1 |

| | | | | | | | | | | | | | | |
|---|---|---|---|---|---|---|---|---|---|---|---|---|---|---|
| 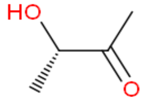 | 0 | 2494 | C4H8O2 | -18.33 | 14 | 2 | -9.0907 | -2.187 | -7.437 | 2.609 | 9.9286 | 5.0964 | -38.73429 | 0.35809 | 1 |
| 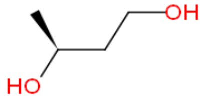 | 0 | 1954 | C4H10O2 | -18.31 | 16 | 4 | -8.4305 | -2.2675 | -9.84 | 0 | 9.0599 | 7.856 | -51.89635 | 0.46459 | 1 |
| 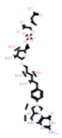 | 0 | 3145 | C31H45N6O16P | -18.31 | 96 | 21 | -17.204 | -5.8614 | -34.18 | 1.9677 | 37.236 | 32.752 | -174.2361 | 2.48132 | 1 |
| 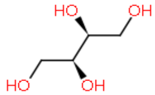 | 0 | 2956 | C4H10O4 | -18.3 | 18 | 7 | -10.92 | -1.5969 | -8.927 | 1.9173 | 12 | 7.5438 | -48.32786 | 0.44976 | 1 |
| 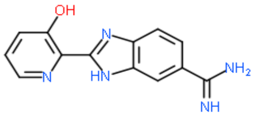 | 0 | 2710 | C13H11N5O | -18.28 | 30 | 0 | -8.0142 | -3.7999 | -18.74 | 0 | 19.767 | 12.82 | -106.8483 | 1.1918 | 1 |
| 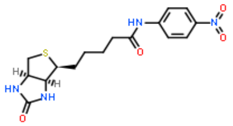 | 0 | 3205 | BNI | -18.28 | 45 | 7 | -6.9531 | -4.8047 | -24.78 | 3.2743 | 16.981 | 12.747 | -94.61752 | 1.49593 | 1 |
| 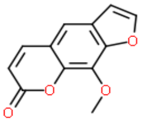 | 0 | 440 | Xanthotoxin | -18.25 | 24 | 0 | -5.244 | -3.6647 | -16.85 | 1.3602 | 11.312 | 7.6145 | -74.88487 | 0.85362 | 1 |
| 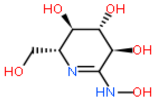 | 0 | 2115 | C6H12N2O5 | -18.24 | 25 | 7 | -12.307 | -2.0126 | -12.33 | 0 | 18.667 | 12.692 | -52.33595 | 0.69445 | 1 |
| 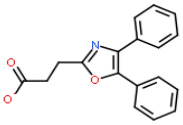 | 0 | 868 | 991 | -18.24 | 36 | 5 | -8.4169 | -5.1577 | -20.69 | 1.1899 | 16.689 | 16.63 | -141.4884 | 1.36947 | 1 |
| 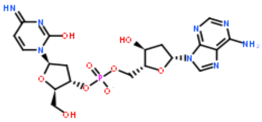 | 0 | 2300 | C19H25N8O9P | -18.23 | 61 | 11 | -15.442 | -4.5567 | -29.09 | 11.879 | 29.992 | 25.388 | -146.1995 | 2.01547 | 1 |
| 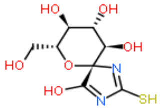 | 0 | 2660 | C8H12N2O6S | -18.23 | 29 | 7 | -12.058 | -1.9701 | -16.08 | 4.0534 | 20.289 | 11.856 | -78.87434 | 1.17655 | 1 |

| | | | | | | | | | | | | | | |
|---|---|---|---|---|---|---|---|---|---|---|---|---|---|---|
| 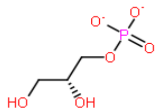 | 0 | 2245 | C3H9O6P | -18.22 | 17 | 6 | -12.246 | -1.6024 | -11.69 | 0 | 15.43 | 15.701 | -67.47324 | 0.64998 | 1 |
| 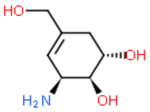 | 0 | 1567 | C7H13NO3 | -18.19 | 24 | 5 | -9.8539 | -2.3886 | -13.06 | 3.6716 | 13.97 | 8.6783 | -55.2032 | 0.72685 | 1 |
| 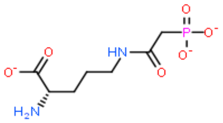 | 0 | 1773 | C7H15N2O6P | -18.19 | 28 | 8 | -12.957 | -2.3822 | -16.7 | 0 | 17.428 | 22.767 | -85.31904 | 1.17896 | 1 |
| 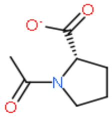 | 0 | 3034 | C7H11NO3 | -18.18 | 21 | 2 | -8.8459 | -2.7654 | -12.52 | 4.7682 | 11.773 | 8.6017 | -68.87727 | 0.74053 | 1 |
| 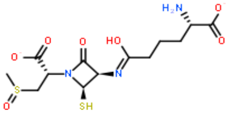 | 0 | 2699 | C13H21N3O7S2 | -18.16 | 44 | 13 | -12.96 | -3.5554 | -25.31 | 5.4759 | 25.044 | 18.873 | -115.5452 | 1.64923 | 1 |
| 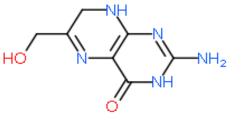 | 0 | 1876 | C7H9N5O2 | -18.16 | 23 | 2 | -10.624 | -1.909 | -17.73 | 3.2027 | 19.248 | 15.42 | -73.77535 | 1.04611 | 1 |
| 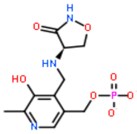 | 0 | 1799 | C11H16N3O7P | -18.15 | 36 | 6 | -12.059 | -3.5001 | -23.18 | 6.3023 | 20.682 | 22.717 | -100.994 | 1.3003 | 1 |
| 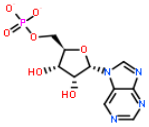 | 0 | 2881 | C10H13N4O7P | -18.14 | 33 | 6 | -10.987 | -2.8602 | -17.05 | 4.5108 | 17.321 | 14.137 | -96.00211 | 1.29326 | 1 |
| 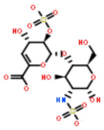 | 0 | 2092 | C12H16NO16S2 | -18.13 | 47 | 12 | -14.31 | -2.7015 | -20.68 | 0.931 | 29.971 | 15.654 | -93.69448 | 1.35655 | 1 |
| 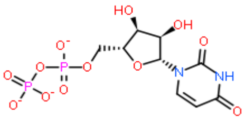 | 0 | 3102 | Uridine | -18.11 | 36 | 8 | -15.153 | -2.3695 | -18.45 | 0 | 25.679 | 24.553 | -118.2679 | 1.32158 | 1 |
| 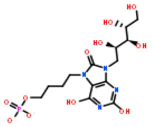 | 0 | 1892 | C14H23N4O11P | -18.1 | 51 | 15 | -12.825 | -4.1232 | -24.3 | 1.8961 | 30.424 | 12.795 | -113.8553 | 1.42446 | 1 |

| | | | | | | | | | | | | | | | | |
|---|---|---|---|---|---|---|---|---|---|---|---|---|---|---|---|---|
| 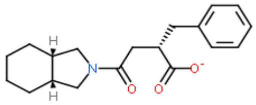 | 0 | 1121 | C19H25NO3 | -18.09 | 47 | 6 | -8.9102 | -5.3981 | -19.79 | 2.9073 | 15.648 | 16.321 | -124.0448 | 1.56314 | 1 |
| 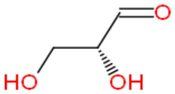 | 0 | 2265 | C3H6O3 | -18.08 | 12 | 4 | -10.358 | -1.0752 | -9.994 | 4.5448 | 10.614 | 8.7096 | -40.54583 | 0.40211 | 1 |
| 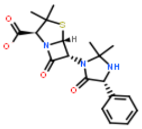 | 0 | 622 | 739 | -18.08 | 49 | 3 | -9.3335 | -4.0671 | -18.9 | 3.6353 | 18.54 | 14.043 | -121.0074 | 1.53145 | 1 |
| 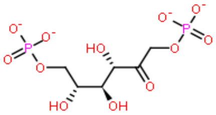 | 0 | 2242 | C6H14O12P2 | -18.08 | 30 | 12 | -12.647 | -1.8366 | -17.95 | 0 | 23.283 | 12.784 | -112.4675 | 1.16339 | 1 |
| 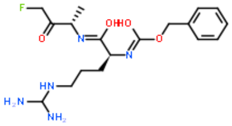 | 0 | 3195 | C18H28FN5O4 | -18.07 | 56 | 16 | -9.4029 | -5.6898 | -33.72 | 5.3846 | 27.424 | 14.927 | -77.3944 | 1.61243 | 1 |
| 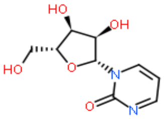 | 0 | 2757 | C9H12N2O5 | -18.06 | 28 | 5 | -9.1056 | -2.0762 | -14.72 | 3.8067 | 17.432 | 4.1373 | -31.64436 | 0.91028 | 1 |
| 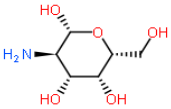 | 0 | 2397 | C6H13NO5 | -18.06 | 25 | 6 | -11.295 | -1.9456 | -13.2 | 2.5139 | 18.527 | 9.1158 | -64.44981 | 0.86077 | 1 |
| 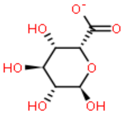 | 0 | 2641 | C6H10O7 | -18.06 | 22 | 5 | -8.6657 | -1.1693 | -8.191 | 0 | 14.574 | -1.65 | -26.06768 | 0.99393 | 1 |
| 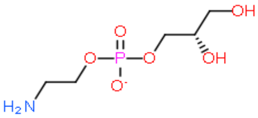 | 0 | 3148 | C5H14NO6P | -18.06 | 26 | 10 | -10.305 | -2.4041 | -15.16 | 0 | 16.684 | 9.5031 | -79.38762 | 0.97113 | 1 |
| 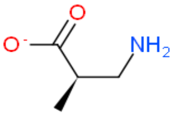 | 0 | 3099 | C4H8NO2 | -18.03 | 15 | 3 | -8.8211 | -1.7413 | -10.15 | 5.4955 | 12.976 | 1.1688 | -41.64593 | 0.4174 | 1 |
| 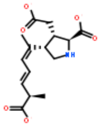 | 0 | 2555 | C15H21NO6 | -18.02 | 40 | 6 | -17.094 | -3.563 | -11.48 | 7.6647 | 23.249 | 20.399 | -104.2492 | 1.34586 | 1 |

| | | | | | | | | | | | | | | | |
|---|---|---|---|---|---|---|---|---|---|---|---|---|---|---|---|
| 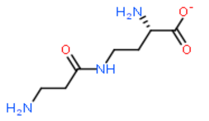 | 0 | 2917 | C7H15N3O3 | -18.02 | 27 | 8 | -10.86 | -2.2643 | -14.47 | 4.3369 | 17.922 | 6.9677 | -81.27322 | 0.96741 | 1 |
| 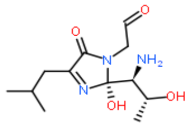 | 0 | 2218 | C12H21N3O4 | -17.99 | 40 | 9 | -10.04 | -3.6968 | -19.42 | 2.1778 | 20.088 | 11.412 | -94.86665 | 1.24986 | 1 |
| 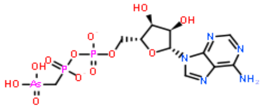 | 0 | 2635 | C11H18AsN5O12P2 | -17.99 | 47 | 12 | -16.764 | -3.3996 | -30.97 | 0 | 38.681 | 32.525 | -145.0654 | 1.58216 | 1 |
| 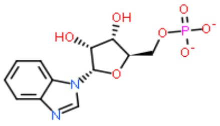 | 0 | 2768 | C12H15N2O7P | -17.98 | 35 | 6 | -4.404 | -3.768 | -18.92 | 0 | 15.96 | -1.462 | -75.37023 | 1.26931 | 1 |
| 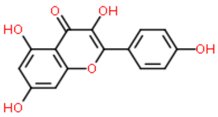 | 0 | 1627 | KMP | -17.97 | 31 | 0 | -7.6514 | -3.3789 | -19.66 | 0 | 18.96 | 13.776 | -88.90299 | 1.24529 | 1 |
| 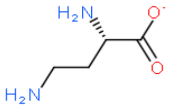 | 0 | 3452 | C4H10N2O2 | -17.96 | 17 | 5 | -10.061 | -1.6526 | -10.98 | 0 | 16.349 | 6.5387 | -45.18782 | 0.62602 | 1 |
| 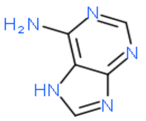 | 0 | 68 | 173 | -17.95 | 15 | 0 | -8.6464 | -2.2532 | -5.325 | 0.9762 | 14.092 | -0.604 | -2.466379 | 0.46381 | 1 |
| 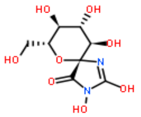 | 0 | 2180 | C8H12N2O8 | -17.95 | 30 | 7 | -10.896 | -1.9971 | -16.6 | 2.3849 | 19.402 | 11.17 | -86.97363 | 1.09318 | 1 |
| 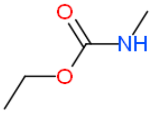 | 0 | 2559 | C4H9NO2 | -17.94 | 16 | 2 | -7.5262 | -2.7375 | -11.9 | 3.4682 | 10.685 | 5.3662 | -36.41633 | 0.66834 | 1 |
| 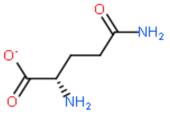 | 0 | 26 | C5H10N2O3 | -17.93 | 19 | 5 | -10.165 | -1.6922 | -11.82 | 1.6239 | 16.252 | 6.9263 | -50.7247 | 0.74818 | 1 |
| 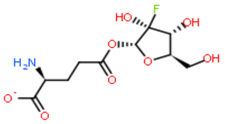 | 0 | 3045 | C10H16FNO8 | -17.92 | 35 | 10 | -16.764 | -2.6431 | -16.11 | 4.3104 | 29.145 | 18.072 | -110.4998 | 1.24605 | 1 |

| | | | | | | | | | | | | | | | |
|---|---|---|---|---|---|---|---|---|---|---|---|---|---|---|---|
| 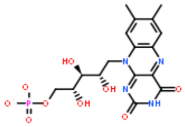 | 0 | 2928 | C17H21N4O9P | -17.9 | 50 | 10 | -13.724 | -3.9929 | -32.78 | 2.5814 | 25.521 | 38.767 | -140.6091 | 1.66619 | 1 |
| 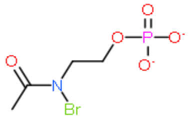 | 0 | 2001 | C4H9BrNO5P | -17.9 | 19 | 5 | -10.63 | -3.0357 | -10.34 | 0 | 14.162 | 11.736 | -55.15729 | 0.77995 | 1 |
| 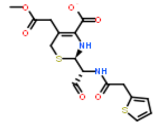 | 0 | 3117 | CEP | -17.89 | 43 | 8 | -12.408 | -4.4281 | -24.91 | 7.0045 | 21.448 | 24.684 | -105.3163 | 1.77332 | 1 |
| 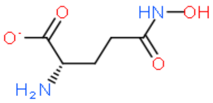 | 0 | 2179 | C5H10N2O4 | -17.89 | 20 | 7 | -9.7086 | -1.6631 | -15.09 | 0 | 18.353 | 7.4492 | -62.57579 | 0.75651 | 1 |
| 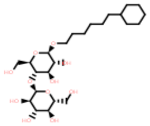 | 0 | 3136 | C24H44O11 | -17.88 | 79 | 19 | -11.5 | -6.0425 | -22.29 | 9.3559 | 18.389 | 10.226 | -152.7276 | 1.79411 | 1 |
| 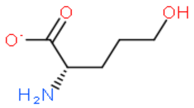 | 0 | 2792 | C5H11NO3 | -17.85 | 19 | 6 | -9.803 | -1.9268 | -11.73 | 0 | 16.756 | 5.7663 | -51.04336 | 0.68162 | 1 |
| 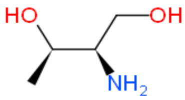 | 0 | 1509 | C4H11NO2 | -17.85 | 18 | 5 | -10.079 | -1.9068 | -11.31 | 2.7988 | 13.621 | 8.0938 | -42.04968 | 0.47396 | 1 |
| 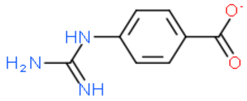 | 0 | 2192 | C8H9N3O2 | -17.85 | 21 | 3 | -9.203 | -2.1297 | -15.17 | 1.1367 | 16.115 | 11.318 | -54.30246 | 0.76662 | 1 |
| 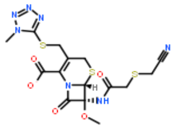 | 0 | 169 | 274 | -17.83 | 46 | 8 | -13.628 | -5.2381 | -27.76 | 5.6762 | 29.775 | 26.327 | -137.0719 | 2.10831 | 1 |
| 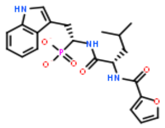 | 0 | 1806 | FLX | -17.82 | 55 | 8 | -5.818 | -3.7823 | -26.14 | 13.625 | 19.716 | -3.317 | -60.6322 | 1.57356 | 1 |
| 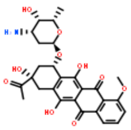 | 0 | 580 | 694 | -17.78 | 67 | 6 | -8.4008 | -6.0431 | -24.41 | 0 | 22 | 17.936 | -119.4286 | 1.65328 | 1 |

| | | | | | | | | | | | | | | | |
|---|---|---|---|---|---|---|---|---|---|---|---|---|---|---|---|
| 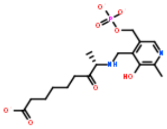 | 0 | 2842 | C17H27N2O8P | -17.77 | 52 | 13 | -9.2874 | -4.3032 | -25.24 | 0 | 19.927 | 16.835 | -44.80204 | 1.45374 | 1 |
| 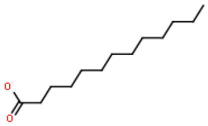 | 0 | 2181 | TDA | -17.76 | 40 | 11 | -8.9762 | -5.0008 | -18.27 | 3.2034 | 13.753 | 11.714 | -79.56374 | 1.20815 | 1 |
| 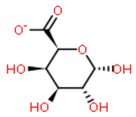 | 0 | 3174 | C6H10O7 | -17.75 | 22 | 5 | -10.017 | -1.7849 | -11.1 | 0 | 16.573 | 6.7454 | -71.28912 | 0.76262 | 1 |
| 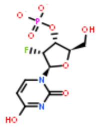 | 0 | 2837 | C9H12FN2O8P | -17.7 | 31 | 6 | -12.316 | -2.7244 | -14.77 | 9.7772 | 21.085 | 7.2317 | -99.25247 | 1.24664 | 1 |
| 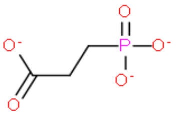 | 0 | 3050 | C3H7O5P | -17.68 | 13 | 3 | -10.511 | -1.3212 | -10.25 | 0.9811 | 14.463 | 10.333 | -49.63997 | 0.57617 | 1 |
| 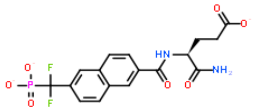 | 0 | 3356 | TPI | -17.66 | 43 | 7 | -9.8424 | -3.5484 | -17.07 | 1.2808 | 25.379 | 4.0781 | -115.7236 | 2.03766 | 1 |
| 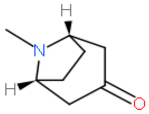 | 0 | 1647 | C8H13NO | -17.65 | 23 | 0 | -6.9451 | -3.4257 | -10.33 | 0 | 8.9439 | 8.8502 | -66.8586 | 0.65004 | 1 |
| 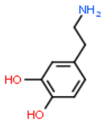 | 0 | 865 | 988 | -17.64 | 22 | 3 | -7.8955 | -2.7112 | -16.11 | 2.9914 | 15.131 | 8.0523 | -46.57449 | 0.78724 | 1 |
| 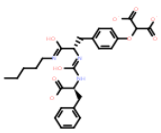 | 0 | 2172 | C27H33N3O9 | -17.64 | 69 | 18 | -13.303 | -5.962 | -34.32 | 3.4634 | 30.16 | 28.542 | -141.022 | 2.09518 | 1 |
| 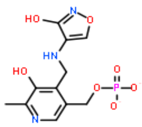 | 0 | 2784 | C11H14N3O7P | -17.62 | 34 | 5 | -9.6858 | -3.2122 | -19.58 | 0 | 18.775 | 17.483 | -103.0427 | 1.35028 | 1 |
| 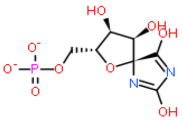 | 0 | 2223 | C7H11N2O9P | -17.61 | 28 | 7 | -11.872 | -1.6775 | -17.93 | 0 | 22.646 | 15.585 | -90.82493 | 1.06506 | 1 |

| | | | | | | | | | | | | | | | | |
|---|---|---|---|---|---|---|---|---|---|---|---|---|---|---|---|---|
| 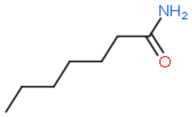 | 0 | 2362 | C7H15NO | -17.61 | 24 | 5 | -8.8454 | -3.0159 | -10.48 | 0.8848 | 12.092 | 7.0771 | -62.93454 | 0.8361 | 1 |
| 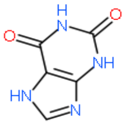 | 0 | 1891 | m | -17.6 | 15 | 0 | -6.8091 | -1.4078 | -15.62 | 0 | 14.12 | 8.4756 | -62.52643 | 0.56156 | 1 |
| 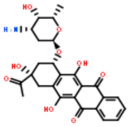 | 0 | 1047 | 1177 | -17.59 | 63 | 6 | -8.727 | -5.7221 | -34.06 | 6.5106 | 25.766 | 23.575 | -137.0732 | 1.96655 | 1 |
| 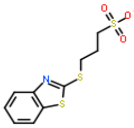 | 0 | 2815 | BTS | -17.57 | 27 | 4 | -9.9889 | -3.6894 | -16.7 | 4.0915 | 16.832 | 14.239 | -83.15843 | 1.2533 | 1 |
| 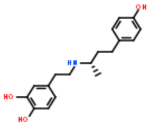 | 0 | 721 | 841 | -17.55 | 45 | 7 | -8.2924 | -4.7838 | -26.34 | 4.2869 | 17.71 | 19.562 | -89.77087 | 1.34925 | 1 |
| 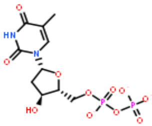 | 0 | 2790 | C10H16N2O11P2 | -17.55 | 38 | 7 | -15.145 | -2.8766 | -17.84 | 0 | 24.43 | 27.301 | -118.3462 | 1.29011 | 1 |
| 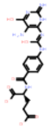 | 0 | 2097 | C17H19N7O7 | -17.51 | 48 | 8 | -11.401 | -4.2633 | -26.9 | 10.213 | 32.605 | 8.6525 | -133.5758 | 1.82398 | 1 |
| 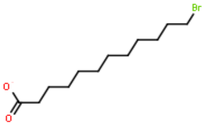 | 0 | 2141 | BRC | -17.49 | 37 | 11 | -9.1909 | -5.149 | -19.01 | 4.2718 | 14.042 | 12.92 | -75.53278 | 1.19659 | 1 |
| 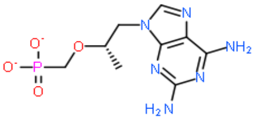 | 0 | 1970 | C9H13N6O4P | -17.49 | 33 | 5 | -11.462 | -4.0748 | -18.93 | 0 | 23.496 | 19.013 | -115.7713 | 1.14536 | 1 |
| 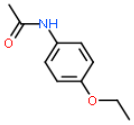 | 0 | 3421 | 3783 | -17.48 | 26 | 1 | -7.3049 | -4.2168 | -15.83 | 0.7937 | 12.638 | 13.793 | -81.36588 | 1.07167 | 1 |
| 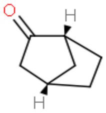 | 0 | 3198 | C7H10O | -17.47 | 18 | 0 | -7.6166 | -2.9145 | -7.63 | 0.1926 | 8.0525 | 7.9152 | -61.239 | 0.50009 | 1 |

| | | | | | | | | | | | | | | | |
|---|---|---|---|---|---|---|---|---|---|---|---|---|---|---|---|
| 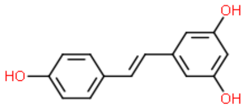 | 0 | 2424 | Resveratrol | -17.46 | 29 | 0 | -7.3477 | -3.8475 | -22.5 | 2.1046 | 18.559 | 16.705 | -82.59509 | 1.2587 | 1 |
| 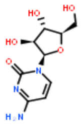 | 0 | 864 | 987 | -17.44 | 30 | 5 | -10.523 | -2.5532 | -16.81 | 2.1465 | 21.947 | 10.541 | -75.16554 | 1.04981 | 1 |
| 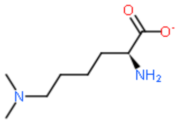 | 0 | 3036 | C8H18N2O2 | -17.44 | 29 | 7 | -11.074 | -3.5763 | -16.38 | 1.5172 | 17.004 | 17.41 | -66.79637 | 1.1378 | 1 |
| 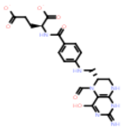 | 0 | 2937 | C20H23N7O7 | -17.38 | 55 | 9 | -13.775 | -4.2601 | -27.16 | 0.198 | 30.307 | 29.154 | -146.4884 | 1.58588 | 1 |
| 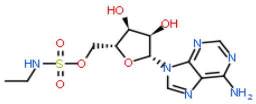 | 0 | 1916 | C12H18N6O6S | -17.37 | 43 | 8 | -12.517 | -4.4955 | -27.1 | 7.7424 | 26.016 | 23.407 | -129.0001 | 1.69539 | 1 |
| 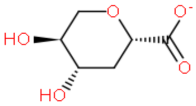 | 0 | 1975 | C6H10O5 | -17.35 | 20 | 3 | -8.5902 | -2.1112 | -10.21 | 0.325 | 14.07 | 5.2489 | -64.24256 | 0.66722 | 1 |
| 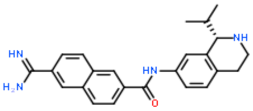 | 0 | 2421 | 303 | -17.34 | 55 | 1 | -5.8219 | -5.7096 | -27.45 | 6.4683 | 19.12 | 14.917 | -54.64777 | 1.08892 | 1 |
| 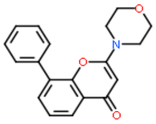 | 0 | 2376 | LY2 | -17.33 | 40 | 1 | -7.2087 | -5.219 | -17.88 | 3.2542 | 15.013 | 12.835 | -92.32898 | 1.65552 | 1 |
| 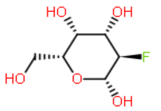 | 0 | 1976 | C6H11FO5 | -17.33 | 23 | 5 | -10.454 | -2.2961 | -11.46 | 3.5762 | 15.643 | 7.9524 | -70.15573 | 0.82154 | 1 |
| 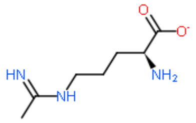 | 0 | 2982 | C7H15N3O2 | -17.31 | 26 | 6 | -11.175 | -2.7015 | -13.58 | 0 | 18.692 | 13.236 | -76.19679 | 1.01377 | 1 |
| 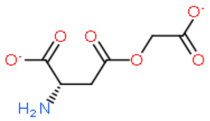 | 0 | 3183 | C6H9NO6 | -17.3 | 20 | 6 | -11.566 | -1.6285 | -12.03 | 0 | 16.047 | 14.324 | -65.96818 | 0.93681 | 1 |

| | | | | | | | | | | | | | | | |
|---|---|---|---|---|---|---|---|---|---|---|---|---|---|---|---|
| 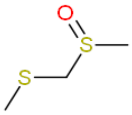 | 0 | 2053 | C3H8OS2 | -17.3 | 14 | 2 | -6.9674 | -2.8753 | -8.823 | 0.3405 | 8.3283 | 5.1978 | -19.40219 | 0.67431 | 1 |
| 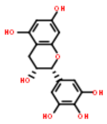 | 0 | 3458 | C15H14O7 | -17.29 | 36 | 2 | -8.4443 | -3.282 | -23.64 | 2.1163 | 21.545 | 17.099 | -110.8325 | 1.37024 | 1 |
| 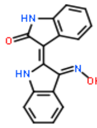 | 0 | 1811 | IXM | -17.28 | 32 | 1 | -7.3437 | -4.1745 | -22.7 | 1.5126 | 18.265 | 17.563 | -103.5964 | 1.55157 | 1 |
| 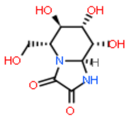 | 0 | 2454 | C8H12N2O6 | -17.28 | 28 | 5 | -11.521 | -1.9981 | -11.72 | 1.012 | 15.516 | 14.675 | -64.25534 | 0.75571 | 1 |
| 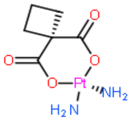 | 0 | 836 | C6H12N2O4Pt | -17.27 | 25 | 0 | -9.2313 | -2.1035 | -16.43 | 0 | 19.042 | 14.613 | -53.1367 | 0.90565 | 1 |
| 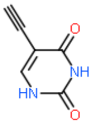 | 0 | 3179 | 3516 | -17.26 | 14 | 0 | -7.7411 | -1.9482 | -12.53 | 0.0738 | 13.274 | 9.3215 | -54.97952 | 0.77471 | 1 |
| 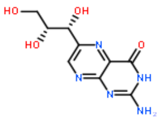 | 0 | 2124 | C9H11N5O4 | -17.24 | 29 | 6 | -12.273 | -2.2723 | -20.89 | 0 | 22.819 | 23.486 | -97.59572 | 1.23192 | 1 |
| 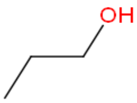 | 0 | 2857 | C3H8O | -17.24 | 12 | 2 | -7.7134 | -2.2915 | -8.468 | 0.7087 | 8.5861 | 6.3858 | -33.1211 | 0.33786 | 1 |
| 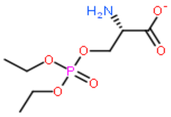 | 0 | 2103 | C7H16NO6P | -17.24 | 30 | 9 | -10.213 | -3.5163 | -16.48 | 0.4525 | 19.369 | 10.972 | -91.40221 | 1.31539 | 1 |
| 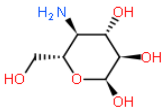 | 0 | 2968 | C6H13NO5 | -17.23 | 25 | 6 | -9.9227 | -2.0402 | -13.11 | 0 | 16.671 | 9.4659 | -79.64047 | 0.74632 | 1 |
| 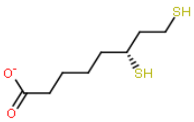 | 0 | 3400 | RED | -17.23 | 27 | 9 | -8.7428 | -2.9635 | -12.76 | 1.6291 | 13.344 | 4.9236 | -66.11104 | 1.16005 | 1 |

| | | | | | | | | | | | | | | | |
|---|---|---|---|---|---|---|---|---|---|---|---|---|---|---|---|
| 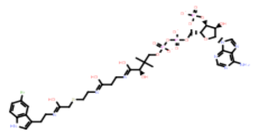 | 0 | 3016 | C33H47BrN9O17P3S | -17.22 | 107 | 29 | -7.9097 | -7.2994 | -48.42 | 7.6423 | 34.258 | 12.763 | -188.7745 | 2.9806 | 1 |
| 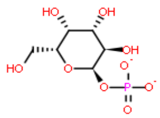 | 0 | 2059 | C6H13O9P | -17.22 | 27 | 7 | -10.57 | -2.2617 | -14.09 | 0 | 14.555 | 15.162 | -95.81008 | 0.91353 | 1 |
| 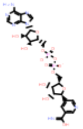 | 0 | 2714 | C21H27N7O14P2 | -17.18 | 69 | 15 | -16.331 | -4.1259 | -28.97 | 0 | 33.98 | 32.402 | -173.4819 | 2.24521 | 1 |
| 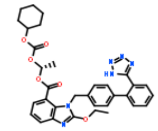 | 0 | 677 | C33H34N6O6 | -17.15 | 79 | 6 | -10.133 | -8.3212 | -37.11 | 15.755 | 26.171 | 28.016 | -163.9373 | 2.40009 | 1 |
| 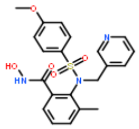 | 0 | 1829 | WAY | -17.15 | 51 | 7 | -6.8839 | -5.9828 | -28.96 | 4.9504 | 19.29 | 17.574 | -128.6402 | 1.50378 | 1 |
| 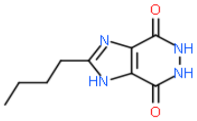 | 0 | 2175 | BDI | -17.12 | 27 | 3 | -7.5692 | -3.2241 | -21 | 0.7529 | 15.816 | 16.785 | -95.103 | 1.07516 | 1 |
| 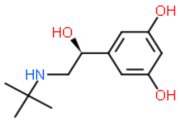 | 0 | 751 | 871 | -17.1 | 35 | 5 | -10.936 | -4.2016 | -13.08 | 2.8256 | 17.746 | 12.809 | -66.20866 | 1.25154 | 1 |
| 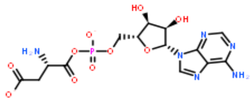 | 0 | 1666 | C14H19N6O10P | -17.1 | 48 | 11 | -14.113 | -4.2505 | -23.32 | 6.6961 | 31.886 | 15.514 | -104.3477 | 1.85858 | 1 |
| 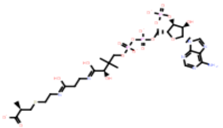 | 0 | 2804 | C25H42N7O18P3S | -17.09 | 91 | 26 | -18.691 | -6.982 | -42 | 5.5755 | 39.496 | 42.887 | -193.5392 | 2.78258 | 1 |
| 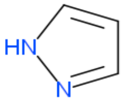 | 0 | 2468 | C3H4N2 | -17.06 | 9 | 0 | -5.9026 | -1.243 | -7.135 | 0.0864 | 5.0619 | 3.0129 | -1.253673 | 0.10107 | 1 |
| 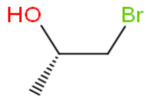 | 0 | 3010 | C3H7BrO | -17.05 | 12 | 2 | -7.3027 | -2.7979 | -9.701 | 1.784 | 8.3278 | 6.7911 | -26.68123 | 0.38497 | 1 |

| | | | | | | | | | | | | | | | |
|---|---|---|---|---|---|---|---|---|---|---|---|---|---|---|---|
| 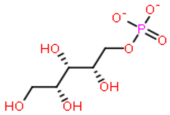 | 0 | 2353 | C5H13O8P | -17.05 | 25 | 10 | -13.92 | -1.9188 | -14.05 | 6.9586 | 20.689 | 11.836 | -81.5294 | 1.08144 | 1 |
| 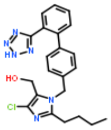 | 0 | 564 | 678 | -17.03 | 53 | 7 | -8.2342 | -6.4193 | -25.26 | 4.4847 | 19.527 | 17.91 | -107.8514 | 1.44421 | 1 |
| 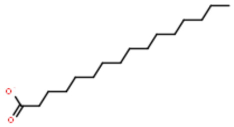 | 0 | 3433 | C16H32O2 | -17.03 | 49 | 14 | -9.1689 | -6.3946 | -19.74 | 2.6169 | 16.334 | 12.076 | -107.513 | 1.46581 | 1 |
| 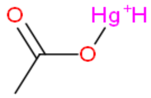 | 0 | 1929 | C2H4HgO2 | -16.98 | 8 | 0 | -5.1477 | -1.1957 | -10.26 | 0.1265 | 6.4127 | 3.3601 | -5.959504 | 0.32129 | 1 |
| 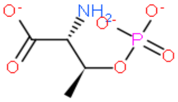 | 0 | 2213 | C4H10NO6P | -16.98 | 19 | 5 | -14.47 | -1.4872 | -12.31 | 9.5655 | 14.036 | 20.711 | -77.21557 | 0.5803 | 1 |
| 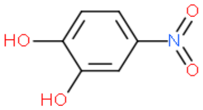 | 0 | 3075 | C6H5NO4 | -16.96 | 16 | 1 | -6.741 | -1.885 | -14.76 | 1.0287 | 9.5866 | 11.674 | -38.53303 | 0.6633 | 1 |
| 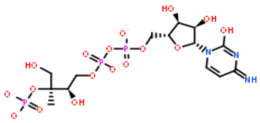 | 0 | 1634 | C14H26N3O17P3 | -16.96 | 59 | 18 | -14.913 | -3.766 | -29.27 | 4.156 | 30.191 | 25.186 | -153.9607 | 2.15992 | 1 |
| 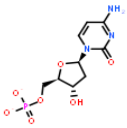 | 0 | 3435 | C9H14N3O7P | -16.96 | 32 | 5 | -12.534 | -2.8339 | -18.78 | 1.9077 | 21.192 | 23.327 | -111.0561 | 1.32622 | 1 |
| 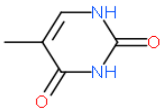 | 0 | 3128 | C5H6N2O2 | -16.96 | 15 | 0 | -6.4703 | -1.8369 | -13.58 | 0.0928 | 11.973 | 7.8631 | -51.6058 | 0.52493 | 1 |
| 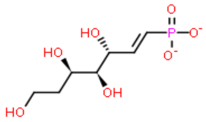 | 0 | 1617 | C7H15O7P | -16.95 | 28 | 10 | -14.137 | -2.5002 | -16.37 | 9.3735 | 19.749 | 15.788 | -78.59303 | 1.15453 | 1 |
| 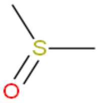 | 0 | 965 | C2H6OS | -16.95 | 10 | 0 | -6.6503 | -2.0626 | -7.254 | 0.0428 | 7.3412 | 4.5215 | -23.90575 | 0.40269 | 1 |

| | | | | | | | | | | | | | | | |
|---|---|---|---|---|---|---|---|---|---|---|---|---|---|---|---|
| 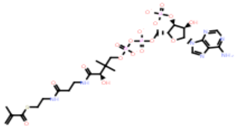 | 0 | 1464 | m | -16.94 | 89 | 21 | -17.9 | -6.3889 | -42.95 | 8.0475 | 35.878 | 47.337 | -195.5492 | 2.40104 | 1 |
| 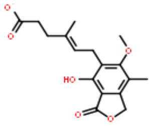 | 0 | 900 | 1024 | -16.94 | 42 | 5 | -9.4701 | -4.9856 | -22.17 | 3.6226 | 16.112 | 23.14 | -108.598 | 1.38265 | 1 |
| 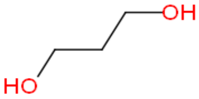 | 0 | 2482 | C3H8O2 | -16.92 | 13 | 4 | -8.0669 | -1.7983 | -8.737 | 0 | 8.8681 | 6.6357 | -41.10866 | 0.37725 | 1 |
| 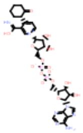 | 0 | 2850 | C27H35N7O15P2 | -16.87 | 85 | 16 | -17.614 | -4.8627 | -30.83 | 11.178 | 34.985 | 29.139 | -189.6164 | 2.63922 | 1 |
| 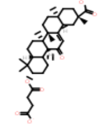 | 0 | 2071 | C34H50O7 | -16.86 | 89 | 5 | -9.4317 | -6.1317 | -19.37 | 3.794 | 26.019 | 8.6205 | -147.0719 | 2.1968 | 1 |
| 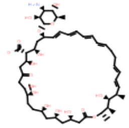 | 0 | 533 | 646 | -16.84 | 139 | 14 | -15.888 | -7.2817 | -36.68 | 21.662 | 43.677 | 16.309 | -194.7458 | 3.62008 | 1 |
| 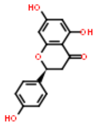 | 0 | 3131 | NAR | -16.83 | 32 | 1 | -6.9176 | -4.0693 | -20.14 | 0 | 17.476 | 14.888 | -122.6115 | 1.28985 | 1 |
| 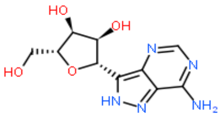 | 0 | 2025 | C10H13N5O4 | -16.76 | 32 | 5 | -11.518 | -3.2814 | -12.91 | 0 | 21.219 | 12.995 | -104.2419 | 1.2917 | 1 |
| 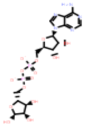 | 0 | 1818 | C15H23N5O14P2 | -16.74 | 57 | 14 | -15.873 | -4.0856 | -32.89 | 12.298 | 31.412 | 29.853 | -139.2398 | 1.94755 | 1 |
| 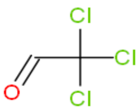 | 0 | 2371 | C2HCl3O | -16.73 | 7 | 1 | -6.2478 | -2.4517 | -11.57 | 0.2754 | 7.5362 | 8.8969 | -37.06432 | 0.45299 | 1 |
| 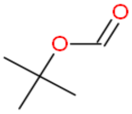 | 0 | 2478 | C5H10O2 | -16.71 | 17 | 1 | -7.4853 | -2.4944 | -10.22 | 3.8389 | 8.451 | 7.3953 | -54.41763 | 0.50727 | 1 |

| | | | | | | | | | | | | | | | |
|---|---|---|---|---|---|---|---|---|---|---|---|---|---|---|---|
| 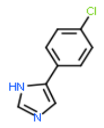 | 0 | 2670 | C9H7ClN2 | -16.68 | 19 | 1 | -3.1367 | -3.3712 | -16.75 | 0.6113 | 6.9095 | 6.2361 | -13.89604 | 0.51912 | 1 |
| 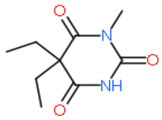 | 0 | 353 | 463 | -16.66 | 28 | 2 | -7.3645 | -3.4755 | -15.02 | 1.092 | 12.795 | 11.924 | -79.06365 | 0.81522 | 1 |
| 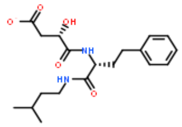 | 0 | 3227 | R99 | -16.65 | 53 | 12 | -10.993 | -6.0537 | -26.06 | 4.7118 | 18.543 | 26.28 | -149.8942 | 1.47706 | 1 |
| 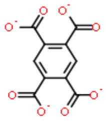 | 0 | 2460 | PMA | -16.64 | 20 | 4 | -10.183 | -1.7905 | -15.58 | 0 | 18.828 | 14.208 | -79.53661 | 1.08512 | 1 |
| 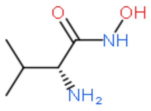 | 0 | 2413 | C5H12N2O2 | -16.64 | 21 | 5 | -9.7045 | -2.3273 | -13.29 | 0.3636 | 15.268 | 12.26 | -44.05579 | 0.68557 | 1 |
| 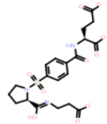 | 0 | 3214 | C20H25N3O10S | -16.64 | 56 | 12 | -13.049 | -5.6405 | -28.38 | 3.469 | 28.415 | 27.185 | -178.4755 | 2.02816 | 1 |
| 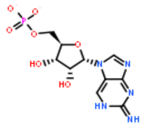 | 0 | 2479 | C10H14N5O7P | -16.62 | 35 | 6 | -14.19 | -2.2574 | -18.98 | 3.3477 | 26.281 | 21.902 | -88.97146 | 1.20655 | 1 |
| 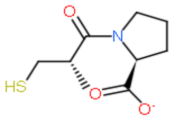 | 0 | 1067 | 1197 | -16.62 | 28 | 5 | -8.6202 | -3.3367 | -13.37 | 0.8382 | 13.909 | 10.399 | -84.1928 | 1.14434 | 1 |
| 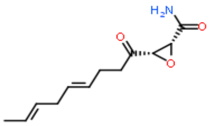 | 0 | 909 | 1034 | -16.61 | 33 | 7 | -5.1578 | -3.6635 | -21.09 | 0 | 11.568 | 10.766 | -44.93711 | 0.93984 | 1 |
| 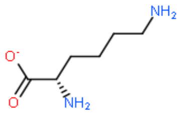 | 0 | 20 | C6H14N2O2 | -16.6 | 23 | 7 | -11.093 | -2.1036 | -12.63 | 0.8471 | 19.267 | 9.6076 | -62.03313 | 0.83767 | 1 |
| 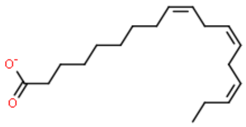 | 0 | 28 | 132 | -16.6 | 49 | 13 | -8.562 | -5.5778 | -23.2 | 4.5246 | 15.652 | 14.764 | -105.0201 | 1.75376 | 1 |

| | | | | | | | | | | | | | | | |
|---|---|---|---|---|---|---|---|---|---|---|---|---|---|---|---|
| 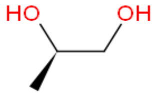 | 0 | 1913 | C3H8O2 | -16.59 | 13 | 3 | -8.1534 | -1.3319 | -8.464 | 2.0693 | 9.7859 | 4.5577 | -3.414861 | 0.51363 | 1 |
| 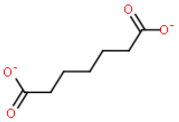 | 0 | 1631 | C7H12O4 | -16.59 | 21 | 6 | -11.597 | -2.2535 | -9.08 | 0.8571 | 14.165 | 13.035 | -64.9615 | 0.97789 | 1 |
| 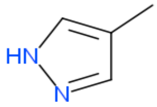 | 0 | 1083 | 1213 | -16.56 | 12 | 0 | -5.8707 | -1.6337 | -8.043 | 0.2358 | 5.7161 | 4.5488 | -6.788432 | 0.21892 | 1 |
| 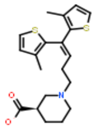 | 0 | 785 | 906 | -16.55 | 49 | 4 | -8.7626 | -5.743 | -21.96 | 5.9375 | 17.227 | 19.004 | -141.9101 | 1.72463 | 1 |
| 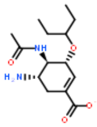 | 0 | 2325 | C14H24N2O4 | -16.55 | 43 | 7 | -9.1983 | -4.3127 | -15.53 | 2.8683 | 17.232 | 9.5308 | -90.87499 | 1.33081 | 1 |
| 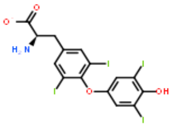 | 0 | 398 | C15H11I4NO4 | -16.52 | 34 | 6 | -8.2548 | -6.4598 | -24.49 | 1.8479 | 24.509 | 15.287 | -123.2017 | 1.76044 | 1 |
| 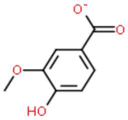 | 0 | 1887 | C8H7O4 | -16.52 | 19 | 1 | -9.1738 | -2.4802 | -13.19 | 4.1215 | 15.467 | 10.413 | -47.35703 | 0.73391 | 1 |
| 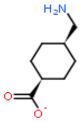 | 0 | 196 | C8H15NO2 | -16.52 | 25 | 3 | -8.8445 | -2.657 | -11.67 | 0.4357 | 14.561 | 9.5873 | -61.02988 | 0.71932 | 1 |
| 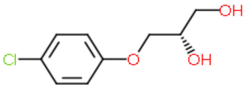 | 0 | 736 | 856 | -16.52 | 24 | 6 | -8.3486 | -3.6076 | -13.02 | 1.8653 | 14.735 | 6.4789 | -53.49895 | 0.97229 | 1 |
| 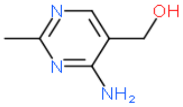 | 0 | 1783 | C6H9N3O | -16.51 | 19 | 2 | -8.8135 | -3.2278 | -15.8 | 2.2366 | 15.627 | 14.375 | -44.56484 | 0.66611 | 1 |
| 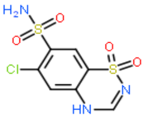 | 0 | 760 | 880 | -16.51 | 23 | 2 | -3.2343 | -2.2658 | -21.24 | 0 | 12.286 | 6.0878 | -62.06804 | 1.12615 | 1 |

| | | | | | | | | | | | | | | | |
|---|---|---|---|---|---|---|---|---|---|---|---|---|---|---|---|
| 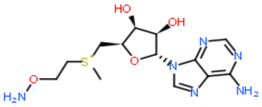 | 0 | 2253 | C13H21N6O4S | -16.5 | 45 | 9 | -10.839 | -4.7029 | -24.26 | 5.4887 | 27.821 | 13.301 | -102.6355 | 1.40522 | 1 |
| 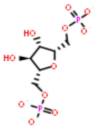 | 0 | 2485 | C6H14O11P2 | -16.49 | 29 | 8 | -11.729 | -1.9228 | -15.56 | 0.8803 | 26.027 | 7.8471 | -82.98218 | 1.24356 | 1 |
| 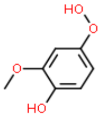 | 0 | 3403 | C7H8O4 | -16.47 | 19 | 1 | -7.3956 | -2.7346 | -13.45 | 0.6625 | 12.266 | 11.102 | -43.90031 | 0.70045 | 1 |
| 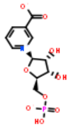 | 0 | 2121 | C11H14NO9P | -16.47 | 35 | 8 | -11.689 | -2.8442 | -21.42 | 0 | 24.294 | 20.32 | -89.80326 | 1.2869 | 1 |
| 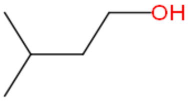 | 0 | 2038 | C5H12O | -16.47 | 18 | 3 | -7.7103 | -2.9664 | -9.998 | 0.9701 | 9.448 | 8.4163 | -51.5205 | 0.54158 | 1 |
| 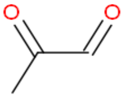 | 0 | 3239 | C3H4O2 | -16.47 | 9 | 1 | -6.9073 | -1.2742 | -9.545 | 0 | 8.8289 | 6.3381 | -23.22144 | 0.35918 | 1 |
| 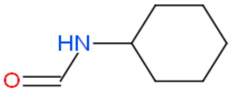 | 0 | 3215 | C7H13NO | -16.46 | 22 | 2 | -8.9415 | -2.8534 | -7.414 | 2.4107 | 9.7005 | 8.4307 | -45.11934 | 0.63819 | 1 |
| 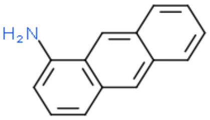 | 0 | 1740 | C14H11N | -16.44 | 26 | 0 | -4.3363 | -4.6136 | -16.2 | 1.4224 | 10.633 | 7.5736 | -83.03426 | 1.0729 | 1 |
| 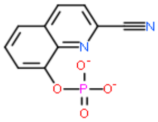 | 0 | 2473 | 772 | -16.42 | 22 | 1 | -12.492 | -2.9252 | -14.11 | 0.87 | 19.033 | 24.033 | -109.3554 | 1.17547 | 1 |
| 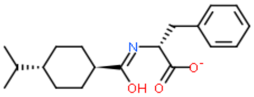 | 0 | 614 | C19H27NO3 | -16.41 | 49 | 7 | -10.331 | -5.2369 | -18.05 | 0 | 15.796 | 23.097 | -135.862 | 1.85192 | 1 |
| 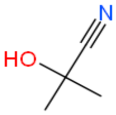 | 0 | 1955 | C4H7NO | -16.4 | 13 | 1 | -8.1563 | -2.1431 | -7.866 | 0.219 | 10.52 | 7.446 | -45.25974 | 0.45701 | 1 |

| | | | | | | | | | | | | | | | |
|---|---|---|---|---|---|---|---|---|---|---|---|---|---|---|---|
| 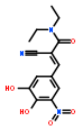 | 0 | 384 | 494 | -16.39 | 37 | 4 | -5.2641 | -4.5342 | -24.7 | 0 | 14.593 | 17.156 | -89.79851 | 1.49407 | 1 |
| 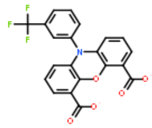 | 0 | 2414 | BPD | -16.38 | 40 | 3 | -9.7184 | -4.54 | -17.22 | 4.0132 | 16.923 | 17.531 | -129.0768 | 1.47419 | 1 |
| 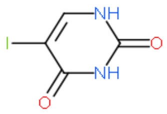 | 0 | 3210 | C4H3IN2O2 | -16.38 | 12 | 0 | -7.413 | -1.9293 | -13.05 | 0 | 13.195 | 10.343 | -52.64296 | 0.73802 | 1 |
| 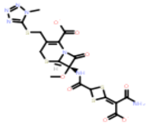 | 0 | 1164 | C17H17N7O8S4 | -16.38 | 51 | 7 | -15.05 | -4.7495 | -31.74 | 1.8992 | 33.895 | 39.19 | -140.9179 | 2.29417 | 1 |
| 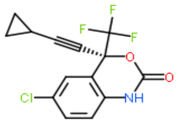 | 0 | 512 | 625 | -16.36 | 30 | 1 | -4.7651 | -4.7198 | -19.55 | 0.398 | 13.036 | 11.795 | -89.78996 | 1.4005 | 1 |
| 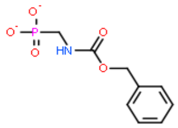 | 0 | 2363 | FOS | -16.35 | 26 | 5 | -10 | -3.4002 | -17.47 | 0 | 17.731 | 18.782 | -77.51055 | 1.07402 | 1 |
| 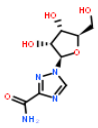 | 0 | 692 | 811 | -16.34 | 29 | 5 | -11.528 | -2.156 | -17.62 | 0 | 24.706 | 15.691 | -77.37184 | 1.15319 | 1 |
| 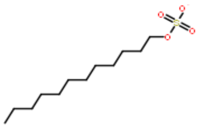 | 0 | 696 | C12H25NaO4S | -16.33 | 42 | 12 | -9.6219 | -5.2717 | -18.32 | 1.7419 | 19.61 | 10.47 | -93.24259 | 1.43134 | 1 |
| 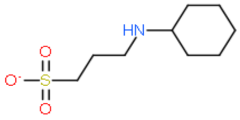 | 0 | 1967 | C9H19NO3S | -16.32 | 32 | 5 | -9.7512 | -3.9762 | -15.14 | 2.4346 | 15.422 | 15.345 | -82.1891 | 1.11526 | 1 |
| 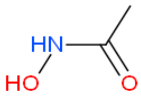 | 0 | 438 | C2H5NO2 | -16.32 | 10 | 2 | -6.3295 | -0.787 | -8.916 | 0 | 10.01 | 0.6521 | -8.574692 | 0.42833 | 1 |
| 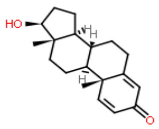 | 0 | 1337 | 1541 | -16.32 | 47 | 1 | -5.8896 | -4.3718 | -19.86 | 1.3288 | 13.695 | 14.699 | -93.50603 | 1.03872 | 1 |

| | | | | | | | | | | | | | | | |
|---|---|---|---|---|---|---|---|---|---|---|---|---|---|---|---|
| 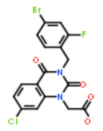 | 0 | 1889 | ZES | -16.31 | 36 | 4 | -8.2894 | -5.3286 | -21.81 | 1.775 | 18.594 | 19.135 | -144.4883 | 1.43036 | 1 |
| 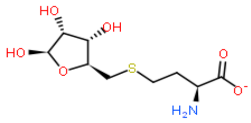 | 0 | 2063 | m | -16.31 | 33 | 10 | -13.324 | -2.8532 | -18.54 | 3.0065 | 27.403 | 14.387 | -84.85407 | 1.32523 | 1 |
| 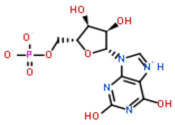 | 0 | 2051 | C10H14N4O9P | -16.3 | 36 | 6 | -14.638 | -2.8662 | -21.44 | 0 | 26.822 | 30.874 | -125.1987 | 1.35313 | 1 |
| 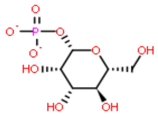 | 0 | 2569 | C6H13O9P | -16.29 | 27 | 7 | -11.55 | -2.1398 | -13.17 | 2.5748 | 21.073 | 9.0642 | -91.30503 | 0.94086 | 1 |
| 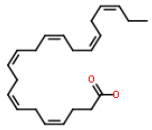 | 0 | 3396 | C22H32O2 | -16.29 | 55 | 14 | -8.028 | -6.0256 | -29.98 | 5.8744 | 16.277 | 21.068 | -126.5781 | 1.7065 | 1 |
| 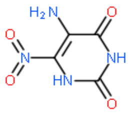 | 0 | 2786 | C4H4N4O4 | -16.27 | 16 | 1 | -6.491 | -1.1125 | -16.73 | 0 | 12.929 | 11.108 | -57.03143 | 0.7266 | 1 |
| 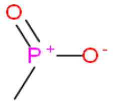 | 0 | 2548 | CH4O2P | -16.26 | 7 | 0 | -6.9046 | -1.2132 | -4.217 | 0.094 | 7.7299 | 0.5887 | -14.13525 | 0.23728 | 1 |
| 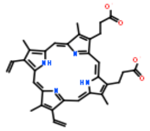 | 0 | 2028 | m | -16.25 | 74 | 6 | -11.791 | -6.8189 | -29.4 | 3.8929 | 24.18 | 35.565 | -145.8143 | 2.07346 | 1 |
| 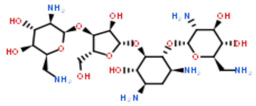 | 0 | 342 | 452 | -16.25 | 88 | 22 | -16.729 | -5.2113 | -28.89 | 9.7054 | 32.911 | 22.354 | -179.2901 | 2.25587 | 1 |
| 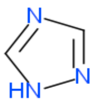 | 0 | 3246 | C2H3N3 | -16.25 | 8 | 0 | -5.9022 | -1.2204 | -8.394 | 0.1255 | 8.0838 | 2.6675 | -5.439551 | 0.32548 | 1 |
| 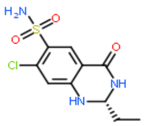 | 0 | 1159 | 1325 | -16.23 | 30 | 3 | -7.0445 | -3.5363 | -23.21 | 0.7375 | 17.646 | 17.715 | -92.20985 | 1.49854 | 1 |

| | | | | | | | | | | | | | | | |
|---|---|---|---|---|---|---|---|---|---|---|---|---|---|---|---|
| 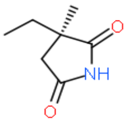 | 0 | 480 | 593 | -16.23 | 21 | 1 | -7.945 | -2.5847 | -11.12 | 1.1138 | 11.296 | 10.591 | -62.60101 | 0.57671 | 1 |
| 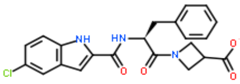 | 0 | 3384 | 700 | -16.21 | 49 | 6 | -6.2121 | -5.6779 | -17.89 | 7.1197 | 14.637 | 3.5649 | -62.14966 | 1.43759 | 1 |
| 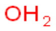 | 0 | 1370 | H41AlMgO15Si4 | -16.2 | 3 | 0 | -7.8358 | -0.0461 | -2.8 | 0.0074 | 7.0845 | 1.7208 | -4.966778 | 0.07329 | 1 |
| 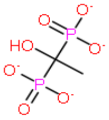 | 0 | 949 | C2H8O7P2 | -16.19 | 15 | 3 | -12.811 | -1.3454 | -11.93 | 0 | 14.632 | 24.663 | -69.49788 | 0.47284 | 1 |
| 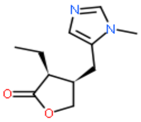 | 0 | 957 | 1085 | -16.17 | 31 | 3 | -7.4699 | -4.3616 | -15.42 | 1.7985 | 14.894 | 10.64 | -81.98206 | 1.14884 | 1 |
| 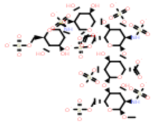 | 0 | 456 | C31H53N3O49S8 | -16.16 | 134 | 36 | -17.149 | -5.3873 | -48.18 | 0 | 62.079 | 16.443 | -168.3963 | 3.90224 | 1 |
| 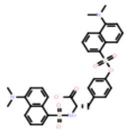 | 0 | 2839 | C33H33N3O7S2 | -16.15 | 77 | 8 | -8.7238 | -6.7962 | -39.54 | 14.051 | 21.984 | 30.758 | -201.0563 | 2.62439 | 1 |
| 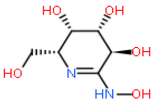 | 0 | 2255 | C6H12N2O5 | -16.14 | 25 | 7 | -9.6177 | -2.0123 | -15.03 | 0 | 18.086 | 10.305 | -44.70398 | 0.77063 | 1 |
| 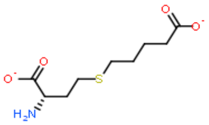 | 0 | 2078 | C9H17NO4S | -16.14 | 30 | 10 | -13.074 | -2.9461 | -16.43 | 2.8927 | 20.976 | 18.001 | -86.73821 | 1.24274 | 1 |
| 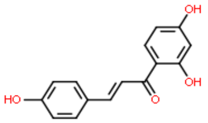 | 0 | 2962 | C15H12O4 | -16.12 | 31 | 0 | -8.6236 | -3.9498 | -21.32 | 1.1013 | 21.382 | 19.638 | -89.41995 | 1.46079 | 1 |
| 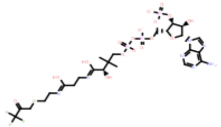 | 0 | 1733 | C24H37F3N7O17P3S | -16.1 | 88 | 25 | -9.869 | -5.4287 | -34.68 | 12.753 | 32.919 | -0.245 | -163.1915 | 3.10542 | 1 |

| | | | | | | | | | | | | | | | |
|---|---|---|---|---|---|---|---|---|---|---|---|---|---|---|---|
| 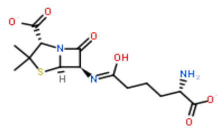 | 0 | 3206 | C14H21N3O6S | -16.09 | 43 | 9 | -7.6935 | -3.3627 | -18.63 | 0 | 19.477 | 6.4413 | -96.79979 | 1.49296 | 1 |
| 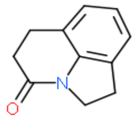 | 0 | 2467 | C11H11NO | -16.09 | 24 | 0 | -5.4204 | -3.8205 | -12.18 | 0.5822 | 9.1856 | 7.7695 | -73.54794 | 0.81736 | 1 |
| 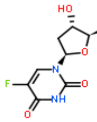 | 0 | 216 | 322 | -16.09 | 28 | 4 | -6.7973 | -2.6655 | -17.17 | 0.7784 | 17.958 | 5.6537 | -78.6339 | 1.04931 | 1 |
| 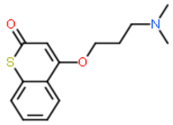 | 0 | 2195 | FC1 | -16.07 | 35 | 4 | -8.4602 | -4.8438 | -20.74 | 4.1146 | 16.813 | 17.937 | -95.12394 | 1.50894 | 1 |
| 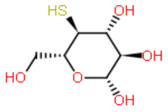 | 0 | 3236 | C6H12O5S | -16.07 | 24 | 6 | -10.459 | -2.1079 | -11.62 | 3.5336 | 14.766 | 10.054 | -63.85308 | 0.93066 | 1 |
| 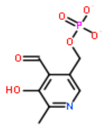 | 0 | 11 | 114 | -16.06 | 24 | 3 | -10.512 | -2.8233 | -16.47 | 8.0378 | 18.226 | 13.037 | -66.55671 | 1.03809 | 1 |
| 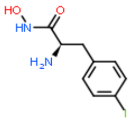 | 0 | 1744 | IPO | -16.06 | 25 | 6 | -7.9889 | -3.3457 | -18.75 | 1.9249 | 17.38 | 11.147 | -42.92811 | 1.16427 | 1 |
| 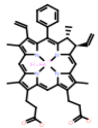 | 0 | 2201 | C40H40FeN4O4 | -16.06 | 85 | 8 | -8.8321 | -7.5482 | -31.08 | 4.6761 | 24.294 | 25.141 | -164.6976 | 2.23812 | 1 |
| 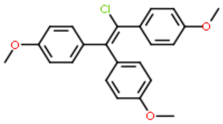 | 0 | 164 | 269 | -16.04 | 48 | 0 | -4.8581 | -7.0457 | -28.25 | 6.6536 | 16.163 | 19.856 | -105.8125 | 1.48062 | 1 |
| 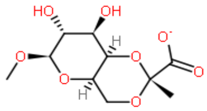 | 0 | 1443 | C10H16O8 | -16.04 | 33 | 4 | -9.4911 | -3.3536 | -10.44 | 2.9785 | 18.646 | 3.773 | -87.14909 | 1.05436 | 1 |
| 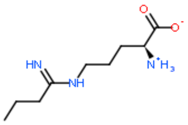 | 0 | 3353 | C9H20N3O2 | -16.03 | 33 | 7 | -10.257 | -3.5564 | -17.71 | 3.3938 | 16.551 | 17.205 | -83.85803 | 1.21337 | 1 |

| | | | | | | | | | | | | | | | |
|---|---|---|---|---|---|---|---|---|---|---|---|---|---|---|---|
| 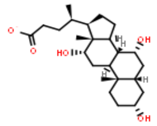 | 0 | 2379 | CHD | -16.03 | 68 | 7 | -6.7011 | -4.6286 | -19.2 | 1.1438 | 21.778 | 3.0276 | -101.2735 | 1.66283 | 1 |
| 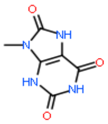 | 0 | 2970 | C6H6N4O3 | -16.01 | 19 | 0 | -6.5152 | -1.4561 | -18.41 | 0 | 16.317 | 11.542 | -81.07187 | 0.87409 | 1 |
| 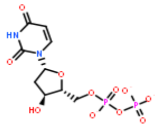 | 0 | 3081 | C9H14N2O11P2 | -16.01 | 35 | 7 | -17.4 | -2.2877 | -23.73 | 5.7317 | 27.878 | 37.477 | -123.6218 | 1.42299 | 1 |
| 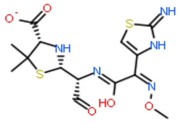 | 0 | 3104 | C14H19N5O5S2 | -16 | 44 | 6 | -6.7752 | -4.0738 | -23.12 | 0 | 23.631 | 8.589 | -72.38785 | 1.36454 | 1 |
| 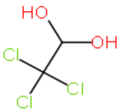 | 0 | 1359 | 1563 | -15.99 | 10 | 3 | -8.1387 | -2.6205 | -10.04 | 0 | 11.541 | 8.9947 | -48.55934 | 0.50176 | 1 |
| 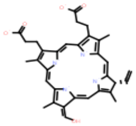 | 0 | 2905 | C33H32FeN4O5 | -15.99 | 72 | 7 | -10.456 | -5.643 | -21.96 | 0 | 23.411 | 21.893 | -135.7113 | 1.86837 | 1 |
| 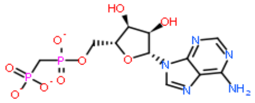 | 0 | 2831 | C11H17N5O9P2 | -15.97 | 41 | 8 | -12.106 | -3.6277 | -19.39 | 0 | 25.278 | 19.281 | -120.7814 | 1.49614 | 1 |
| 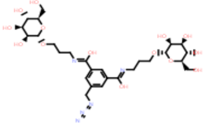 | 0 | 3283 | C27H41N5O14 | -15.96 | 87 | 24 | -22.875 | -5.9506 | -28.39 | 20.474 | 38.592 | 28.266 | -161.0793 | 2.29632 | 1 |
| 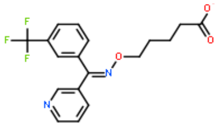 | 0 | 1077 | 1207 | -15.94 | 42 | 6 | -4.2977 | -6.0195 | -26.87 | 3.5906 | 15.023 | 13.357 | -89.67558 | 1.4473 | 1 |
| 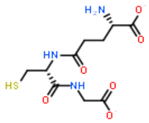 | 0 | 39 | 143 | -15.94 | 35 | 11 | -11.848 | -2.5764 | -21.65 | 4.9037 | 20.323 | 19.138 | -76.65736 | 1.23402 | 1 |
| 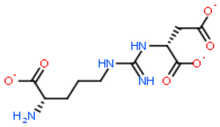 | 0 | 2011 | C10H18N4O6 | -15.94 | 35 | 12 | -14.344 | -2.8091 | -18.24 | 0 | 26.068 | 20.788 | -82.30534 | 1.21786 | 1 |

| | | | | | | | | | | | | | | | |
|---|---|---|---|---|---|---|---|---|---|---|---|---|---|---|---|
| 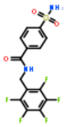 | 0 | 2334 | FFB | -15.92 | 34 | 4 | -4.4891 | -5.0093 | -28.46 | 2.3995 | 16.066 | 17.212 | -130.905 | 1.30258 | 1 |
| 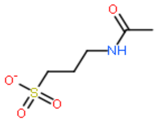 | 0 | 546 | 659 | -15.9 | 21 | 4 | -8.4472 | -2.6573 | -13.58 | 1.6297 | 14.843 | 9.5891 | -64.06223 | 0.76922 | 1 |
| 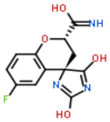 | 0 | 1859 | C12H10FN3O4 | -15.89 | 30 | 4 | -9.3643 | -2.6269 | -19.02 | 0 | 23.133 | 13.382 | -91.94256 | 1.00338 | 1 |
| 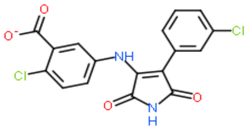 | 0 | 1571 | 679 | -15.87 | 34 | 1 | -7.405 | -3.6117 | -23.48 | 5.4642 | 19.08 | 16.027 | -76.92299 | 0.95744 | 1 |
| 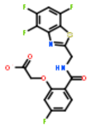 | 0 | 2537 | ID5 | -15.85 | 37 | 4 | -9.2841 | -5.934 | -25.07 | 5.1472 | 18.468 | 26.291 | -159.8236 | 1.79128 | 1 |
| 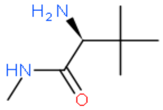 | 0 | 1713 | C7H16N2O | -15.84 | 26 | 3 | -9.8595 | -3.2783 | -11.11 | 3.2562 | 14.473 | 11.826 | -56.63139 | 0.88058 | 1 |
| 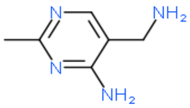 | 0 | 2885 | C6H10N4 | -15.82 | 20 | 2 | -6.4216 | -2.6 | -14.04 | 0.837 | 12.433 | 7.8467 | -15.95132 | 0.45628 | 1 |
| 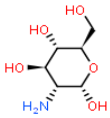 | 0 | 1148 | 1296 | -15.79 | 25 | 6 | -10.41 | -1.8593 | -12.82 | 0.8134 | 16.93 | 11.849 | -76.22932 | 0.81098 | 1 |
| 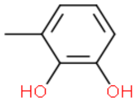 | 0 | 3121 | C7H8O2 | -15.79 | 17 | 0 | -6.6135 | -2.7192 | -12.77 | 0.9481 | 9.4776 | 11.894 | -36.12964 | 0.5599 | 1 |
| 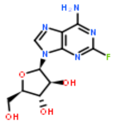 | 0 | 945 | C10H12FN5O4 | -15.78 | 32 | 5 | -11.389 | -3.9858 | -14.82 | 2.4344 | 25.004 | 11.084 | -95.43031 | 1.1761 | 1 |
| 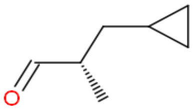 | 0 | 3377 | C7H12O | -15.77 | 20 | 3 | -7.1872 | -3.1071 | -12.64 | 0.7696 | 9.1405 | 12.147 | -59.25283 | 0.73211 | 1 |

| | | | | | | | | | | | | | | | |
|---|---|---|---|---|---|---|---|---|---|---|---|---|---|---|---|
| 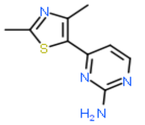 | 0 | 1849 | CK2 | -15.76 | 24 | 0 | -5.7033 | -4.755 | -16.37 | 0.8866 | 15.298 | 9.4184 | -58.30199 | 1.08587 | 1 |
| 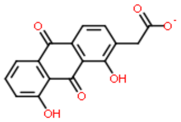 | 0 | 2728 | OAL | -15.75 | 31 | 2 | -7.8911 | -3.1849 | -17.72 | 0 | 18.607 | 13.504 | -141.799 | 1.30021 | 1 |
| 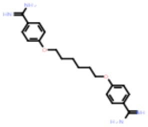 | 0 | 3443 | DID | -15.75 | 52 | 7 | -7.5324 | -5.732 | -33.03 | 6.5989 | 24.821 | 20.298 | -119.6237 | 1.86919 | 1 |
| 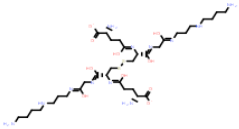 | 0 | 2281 | C34H66N12O10S2 | -15.75 | 122 | 47 | -18.613 | -8.2996 | -46.21 | 12.601 | 37.407 | 29.234 | -143.9808 | 3.15335 | 1 |
| 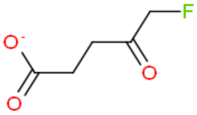 | 0 | 2531 | C5H7FO3 | -15.72 | 15 | 4 | -7.5978 | -2.2268 | -9.577 | 0.0555 | 12.054 | 4.692 | -54.95174 | 0.59719 | 1 |
| 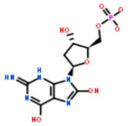 | 0 | 1784 | C10H14N5O8P | -15.71 | 36 | 6 | -12.19 | -2.7365 | -19.81 | 0 | 20.726 | 26.709 | -106.977 | 1.4003 | 1 |
| 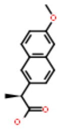 | 0 | 669 | C14H14O3 | -15.71 | 30 | 2 | -9.4124 | -4.2892 | -16.53 | 4.0974 | 15.471 | 18.558 | -115.7756 | 1.31507 | 1 |
| 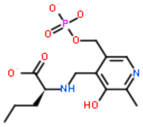 | 0 | 3310 | C13H21N2O7P | -15.69 | 41 | 9 | -8.8973 | -3.677 | -15.38 | 0 | 19.115 | 7.4679 | -95.6409 | 1.37618 | 1 |
| 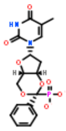 | 0 | 1965 | DPB | -15.68 | 45 | 3 | -5.999 | -2.5731 | -19.54 | 2.1929 | 20.723 | 3.1769 | -50.92584 | 1.34679 | 1 |
| 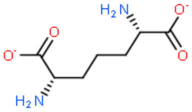 | 0 | 3242 | C7H14N2O4 | -15.67 | 25 | 8 | -10.37 | -2.0537 | -11.8 | 0.1551 | 19.206 | 6.73 | -50.863 | 0.92973 | 1 |
| 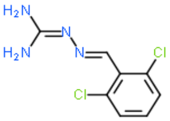 | 0 | 516 | 629 | -15.67 | 22 | 0 | -6.2937 | -3.4337 | -14.98 | 0.9029 | 14.695 | 9.0443 | -71.8467 | 1.10356 | 1 |

| | | | | | | | | | | | | | | | | |
|---|---|---|---|---|---|---|---|---|---|---|---|---|---|---|---|---|
| 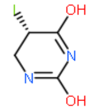 | 0 | 2045 | C4H5IN2O2 | -15.67 | 14 | 2 | -8.5615 | -2.2962 | -12.32 | 0 | 13.425 | 13.011 | -49.28334 | 0.6267 | 1 |
| 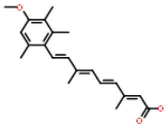 | 0 | 349 | C21H26O3 | -15.66 | 49 | 1 | -6.1918 | -6.7341 | -23.77 | 0.288 | 19.4 | 19.648 | -110.8007 | 1.53301 | 1 |
| 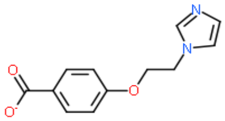 | 0 | 2742 | C12H12N2O3 | -15.66 | 28 | 5 | -5.4856 | -4.1413 | -17.56 | 1.5147 | 13.867 | 6.4373 | -76.66582 | 1.05797 | 1 |
| 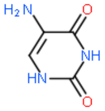 | 0 | 3429 | C4H5N3O2 | -15.65 | 14 | 0 | -6.5247 | -1.3161 | -14.83 | 0 | 14.42 | 8.7193 | -51.33281 | 0.44969 | 1 |
| 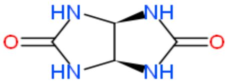 | 0 | 3192 | C4H6N4O2 | -15.62 | 16 | 0 | -7.0141 | -0.5467 | -12.29 | 0.0867 | 14.063 | 6.3351 | -30.91386 | 0.43521 | 1 |
| 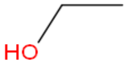 | 0 | 777 | C2H6O | -15.61 | 9 | 1 | -5.7532 | -1.389 | -5.906 | 0.0902 | 5.0464 | 2.0296 | 0.560089 | 0.07995 | 1 |
| 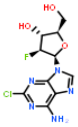 | 0 | 518 | 631 | -15.61 | 31 | 4 | -9.4198 | -4.5239 | -13.92 | 1.602 | 19.527 | 10.865 | -97.06859 | 1.22987 | 1 |
| 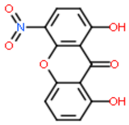 | 0 | 1924 | MNX | -15.6 | 27 | 1 | -4.2947 | -3.2696 | -19.1 | 0 | 12.394 | 10.081 | -95.78874 | 1.32875 | 1 |
| 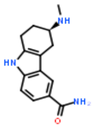 | 0 | 874 | 998 | -15.57 | 35 | 1 | -8.2674 | -3.1572 | -13.13 | 2.0415 | 17.14 | 8.948 | -23.85014 | 0.94903 | 1 |
| 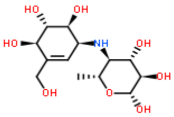 | 0 | 2948 | C13H23NO8 | -15.56 | 45 | 10 | -10.924 | -3.7237 | -21.47 | 1.942 | 23.552 | 16.988 | -108.2297 | 1.3344 | 1 |
| 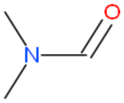 | 0 | 1621 | C3H7NO | -15.54 | 12 | 1 | -6.2205 | -2.0779 | -10.24 | 0.2289 | 7.5632 | 8.2045 | -38.12227 | 0.42973 | 1 |

| | | | | | | | | | | | | | | | |
|---|---|---|---|---|---|---|---|---|---|---|---|---|---|---|---|
| 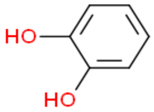 | 0 | 1979 | C6H6O2 | -15.52 | 14 | 0 | -6.5416 | -2.2083 | -11.87 | 0.7272 | 9.1085 | 10.779 | -27.86184 | 0.49337 | 1 |
| 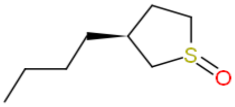 | 0 | 2573 | C8H16OS | -15.51 | 26 | 3 | -6.8743 | -3.8415 | -10.08 | 0.7975 | 9.1793 | 8.1454 | -51.4306 | 0.9124 | 1 |
| 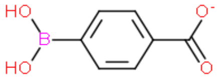 | 0 | 2823 | C7H7BO4 | -15.48 | 18 | 4 | -8.1151 | -2.0812 | -13.28 | 0 | 14.928 | 9.3002 | -46.48742 | 0.76566 | 1 |
| 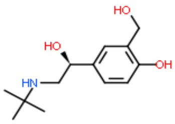 | 0 | 877 | 1001 | -15.47 | 38 | 7 | -9.354 | -4.8561 | -19.29 | 3.4564 | 17.465 | 17.212 | -111.2103 | 1.19419 | 1 |
| 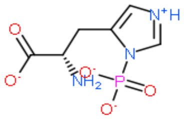 | 0 | 1670 | C6H11N3O5P | -15.47 | 23 | 5 | -5.7269 | -1.5666 | -16.16 | 4.4486 | 15.862 | -2.034 | -32.23955 | 0.76294 | 1 |
| 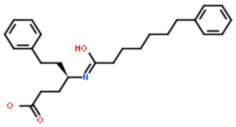 | 0 | 3135 | C25H33NO3 | -15.47 | 61 | 15 | -9.7088 | -6.2093 | -24.05 | 6.1017 | 19.854 | 14.775 | -127.1348 | 1.51679 | 1 |
| 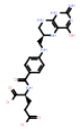 | 0 | 1792 | C19H23N7O6 | -15.42 | 53 | 8 | -15.255 | -4.4565 | -28.41 | 10.368 | 33.091 | 28.391 | -133.0299 | 1.84192 | 1 |
| 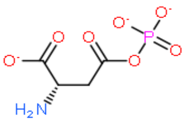 | 0 | 1632 | C4H8NO7P | -15.39 | 18 | 5 | -11.829 | -1.2954 | -13.14 | 0 | 20.563 | 15.343 | -56.31863 | 0.79921 | 1 |
| 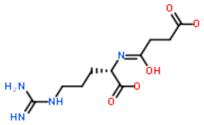 | 0 | 2231 | C10H18N4O5 | -15.35 | 35 | 11 | -12.46 | -2.7037 | -15.5 | 0 | 22.943 | 14.669 | -92.38695 | 1.50322 | 1 |
| 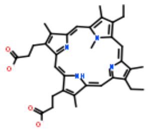 | 0 | 1679 | C35H40N4O4 | -15.34 | 81 | 8 | -8.0889 | -5.9411 | -25.31 | 2.981 | 21.071 | 18.21 | -148.4009 | 2.10581 | 1 |
| 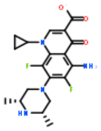 | 0 | 1078 | 1208 | -15.33 | 49 | 2 | -8.9086 | -5.5521 | -17.93 | 6.5553 | 20.132 | 13.065 | -145.7711 | 1.52094 | 1 |

| | | | | | | | | | | | | | | | | |
|---|---|---|---|---|---|---|---|---|---|---|---|---|---|---|---|---|
| 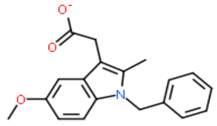 | 0 | 2807 | C19H19NO3 | -15.33 | 41 | 4 | -8.2359 | -5.1011 | -18.23 | 3.8597 | 16.737 | 14.981 | -135.4599 | 1.71307 | 1 |
| 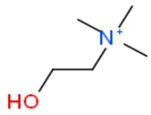 | 0 | 19 | 122 | -15.31 | 21 | 3 | -5.8142 | -2.4102 | -10.4 | 0.2435 | 7.883 | 5.392 | -4.520224 | 0.38844 | 1 |
| 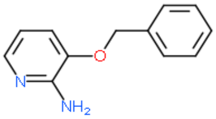 | 0 | 2091 | 3IP | -15.29 | 27 | 2 | -6.477 | -4.6631 | -17.84 | 4.0933 | 14.958 | 10.893 | -84.6823 | 1.11961 | 1 |
| 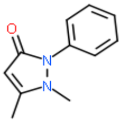 | 0 | 1297 | C15H18Cl6N2O5 | -15.28 | 26 | 1 | -5.2533 | -4.1009 | -18.55 | 1.5806 | 11.372 | 13.99 | -84.54738 | 1.13051 | 1 |
| 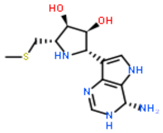 | 0 | 1912 | C12H19N5O2S | -15.27 | 39 | 6 | -7.4602 | -3.4311 | -23.72 | 0.1411 | 21.448 | 14.927 | -58.37715 | 1.15947 | 1 |
| 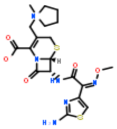 | 0 | 1221 | 1413 | -15.26 | 56 | 5 | -11.367 | -7.0858 | -25.41 | 8.4257 | 25.774 | 24.657 | -139.203 | 1.88533 | 1 |
| 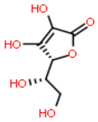 | 0 | 22 | C6H8O6 | -15.24 | 20 | 4 | -9.6748 | -1.8221 | -12.13 | 2.1914 | 19.122 | 6.8735 | -87.05962 | 0.86056 | 1 |
| 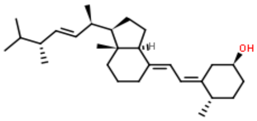 | 0 | 942 | C28H46O | -15.2 | 75 | 5 | -6.9789 | -8.728 | -29.61 | 9.5159 | 19.428 | 22.165 | -137.4041 | 1.93755 | 1 |
| 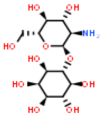 | 0 | 3417 | C12H23NO10 | -15.18 | 46 | 12 | -13.041 | -3.102 | -15.22 | 0.9421 | 20.001 | 18.699 | -83.24503 | 1.41779 | 1 |
| 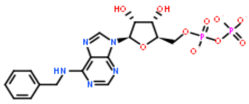 | 0 | 1665 | C17H21N5O10P2 | -15.17 | 52 | 10 | -13.234 | -5.3534 | -31.68 | 5.2168 | 30.302 | 32.907 | -134.7973 | 1.57063 | 1 |
| 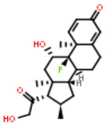 | 0 | 434 | 547 | -15.17 | 56 | 4 | -9.3704 | -4.9822 | -12.69 | 3.4549 | 16.84 | 11.31 | -118.331 | 1.53768 | 1 |

| | | | | | | | | | | | | | | | |
|---|---|---|---|---|---|---|---|---|---|---|---|---|---|---|---|
| 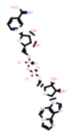 | 0 | 2214 | C23H27N7O14P2 | -15.14 | 72 | 15 | -18.186 | -4.0834 | -36.45 | 17.152 | 36.941 | 34.934 | -186.3897 | 2.48014 | 1 |
| 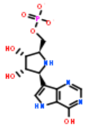 | 0 | 1833 | C11H15N4O7P | -15.14 | 36 | 6 | -7.353 | -3.2444 | -17.99 | 0.3718 | 24.095 | 2.6244 | -89.22801 | 1.48785 | 1 |
| 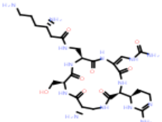 | 0 | 208 | 3000502 | -15.13 | 91 | 14 | -13.201 | -6.0678 | -34.88 | 13.9 | 34.249 | 22.408 | -154.2484 | 2.3641 | 1 |
| 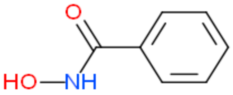 | 0 | 1690 | C7H7NO2 | -15.12 | 17 | 3 | -7.0841 | -2.5871 | -13.99 | 0 | 11.518 | 12.253 | -33.21368 | 0.73885 | 1 |
| 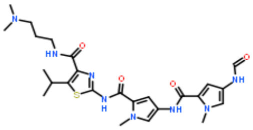 | 0 | 2037 | C25H34N8O4S | -15.11 | 72 | 6 | -10.083 | -8.3771 | -41.59 | 30.249 | 22.657 | 28.58 | -167.6892 | 2.25379 | 1 |
| 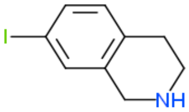 | 0 | 3459 | IDI | -15.08 | 21 | 0 | -4.2674 | -4.2658 | -12.37 | 0.5009 | 10.8 | 3.8541 | -51.10621 | 1.03834 | 1 |
| 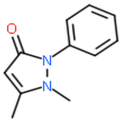 | 0 | 1241 | C11H12N2O | -15.06 | 26 | 1 | -5.245 | -4.1009 | -18.56 | 1.5734 | 11.403 | 14.275 | -84.50046 | 1.13051 | 1 |
| 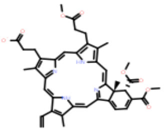 | 0 | 350 | C41H42N4O8 | -15.06 | 94 | 7 | -5.9095 | -8.1651 | -32.84 | 6.109 | 22.175 | 20.76 | -181.3036 | 2.81874 | 1 |
| 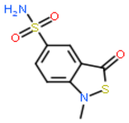 | 0 | 2971 | SG2 | -15.03 | 23 | 2 | -6.8041 | -2.5182 | -20.33 | 0 | 16.023 | 16.65 | -65.0313 | 1.25463 | 1 |
| 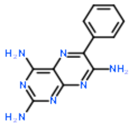 | 0 | 276 | 384 | -15.02 | 30 | 1 | -7.4097 | -4.9339 | -21.87 | 0 | 21.71 | 18.2 | -88.36476 | 1.18567 | 1 |
| 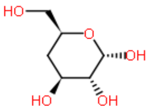 | 0 | 2390 | C6H12O5 | -15.02 | 23 | 5 | -9.6396 | -2.4006 | -12.1 | 0.3225 | 14.344 | 13.818 | -59.58194 | 0.73599 | 1 |

| | | | | | | | | | | | | | | | |
|---|---|---|---|---|---|---|---|---|---|---|---|---|---|---|---|
| 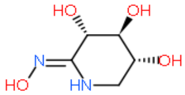 | 0 | 1687 | C5H10N2O4 | -15.02 | 21 | 4 | -8.1079 | -1.9321 | -12.47 | 0 | 14.775 | 8.756 | -55.7844 | 0.58811 | 1 |
| 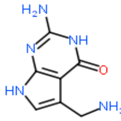 | 0 | 2981 | C7H9N5O | -15.01 | 22 | 2 | -6.473 | -2.0387 | -15.36 | 2.1479 | 16.605 | 4.7722 | -66.33559 | 0.78153 | 1 |
| 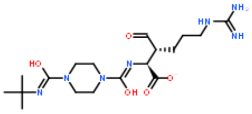 | 0 | 3085 | C18H33N7O5 | -15 | 62 | 14 | -14.459 | -4.9782 | -23.24 | 0 | 28.393 | 27.972 | -103.4149 | 1.71498 | 1 |
| 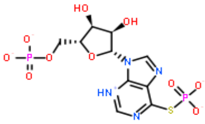 | 0 | 2829 | C10H15N4O10P2S | -15 | 38 | 7 | -9.9713 | -3.1457 | -21.26 | 0 | 22.902 | 18.507 | -104.2832 | 1.3447 | 1 |
| 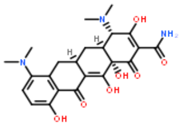 | 0 | 893 | C23H27N3O7 | -14.95 | 60 | 2 | -11.187 | -4.9286 | -21.77 | 4.7213 | 26.289 | 22.243 | -86.49194 | 1.83227 | 1 |
| 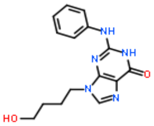 | 0 | 2225 | BPG | -14.94 | 39 | 7 | -7.4613 | -4.4381 | -25.39 | 2.0874 | 18.32 | 19.851 | -84.56257 | 1.11308 | 1 |
| 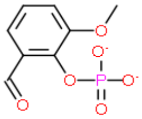 | 0 | 2983 | 300 | -14.93 | 22 | 1 | -10.333 | -2.6579 | -13.95 | 0.6916 | 16.769 | 20.217 | -86.40248 | 0.97623 | 1 |
| 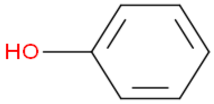 | 0 | 2936 | C6H6O | -14.89 | 13 | 0 | -4.6988 | -2.5891 | -11.69 | 0.4797 | 8.8189 | 5.3355 | -28.37964 | 0.45623 | 1 |
| 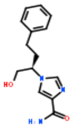 | 0 | 1854 | FR2 | -14.86 | 36 | 6 | -6.1497 | -3.534 | -21.55 | 0.8733 | 13.433 | 15.781 | -71.0317 | 1.00245 | 1 |
| 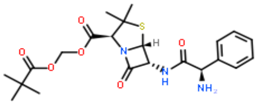 | 0 | 1397 | 1604 | -14.86 | 61 | 8 | -7.2551 | -5.7093 | -28.01 | 2.8732 | 21.255 | 19.504 | -108.1637 | 2.35612 | 1 |
| 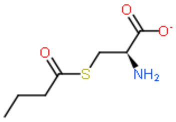 | 0 | 1914 | C7H13NO3S | -14.84 | 24 | 6 | -9.2502 | -2.4806 | -12.07 | 3.4039 | 17.895 | 4.8973 | -48.83308 | 0.92953 | 1 |

| | | | | | | | | | | | | | | | |
|---|---|---|---|---|---|---|---|---|---|---|---|---|---|---|---|
| 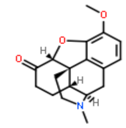 | 0 | 834 | 956 | -14.84 | 43 | 0 | -4.6011 | -3.5886 | -15.08 | 0.3885 | 10.475 | 9.3888 | -75.97409 | 1.10041 | 1 |
| 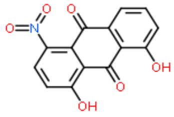 | 0 | 2727 | HNA | -14.83 | 28 | 1 | -2.2356 | -2.9199 | -21.73 | 0 | 12.68 | 6.6736 | -119.1568 | 1.29975 | 1 |
| 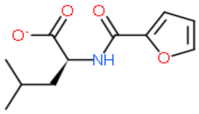 | 0 | 1963 | FLE | -14.82 | 30 | 4 | -7.4905 | -3.739 | -14.98 | 3.3137 | 13.021 | 11.305 | -106.1704 | 1.18271 | 1 |
| 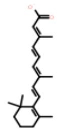 | 0 | 638 | C20H28O2 | -14.81 | 49 | 1 | -5.0317 | -6.3243 | -24.76 | 0 | 16.65 | 20.945 | -105.514 | 1.48583 | 1 |
| 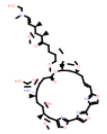 | 0 | 3267 | C48H75N5O14 | -14.81 | 142 | 21 | -11.565 | -10.872 | -39.15 | 11.05 | 37.064 | 20.99 | -142.6624 | 3.08433 | 1 |
| 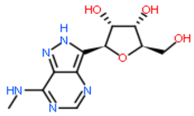 | 0 | 1824 | C11H15N5O4 | -14.79 | 35 | 5 | -8.8443 | -3.2222 | -16.81 | 0 | 19.838 | 13.144 | -80.01744 | 1.16726 | 1 |
| 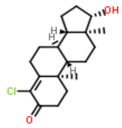 | 0 | 1317 | 1521 | -14.79 | 49 | 1 | -5.7378 | -4.826 | -18.19 | 1.5208 | 13.755 | 14.127 | -99.47215 | 1.08761 | 1 |
| 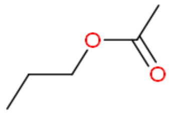 | 0 | 1459 | C5H10O2 | -14.79 | 17 | 2 | -4.9051 | -2.2815 | -9.527 | 0.2656 | 5.725 | 4.5794 | -11.42831 | 0.37322 | 1 |
| 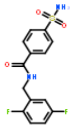 | 0 | 2730 | IOA | -14.79 | 34 | 4 | -2.8172 | -4.5375 | -23.93 | 1.6268 | 14.5 | 7.6624 | -73.39695 | 1.37614 | 1 |
| 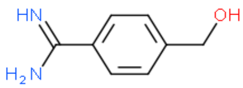 | 0 | 2310 | C8H10N2O | -14.79 | 21 | 2 | -8.0199 | -2.5544 | -12.59 | 2.6776 | 15.217 | 8.4572 | -42.26576 | 0.71649 | 1 |
| 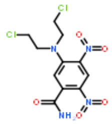 | 0 | 2909 | SN2 | -14.77 | 34 | 6 | -8.1952 | -3.5688 | -22.45 | 3.7297 | 15.229 | 20.523 | -84.76938 | 1.32862 | 1 |

| | | | | | | | | | | | | | | | | |
|---|---|---|---|---|---|---|---|---|---|---|---|---|---|---|---|---|
| 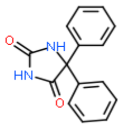 | 0 | 147 | 1775 | -14.73 | 31 | 2 | -4.8151 | -3.6984 | -20.86 | 1.6175 | 15.797 | 10.271 | -119.7456 | 1.31095 | 1 |
| 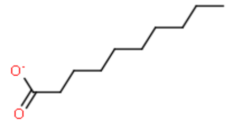 | 0 | 3251 | DKA | -14.73 | 31 | 8 | -8.8894 | -4.3355 | -16.97 | 3.3971 | 12.847 | 16.922 | -95.50925 | 1.16243 | 1 |
| 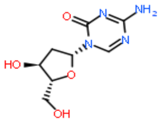 | 0 | 1129 | 1262 | -14.72 | 28 | 4 | -9.0467 | -2.2057 | -15.85 | 1.4435 | 18.501 | 12.646 | -96.99365 | 1.07034 | 1 |
| 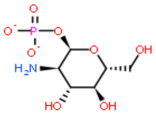 | 0 | 2798 | C6H14NO8P | -14.71 | 28 | 7 | -11.248 | -2.0483 | -15.75 | 0 | 17.363 | 20.678 | -80.18271 | 0.92011 | 1 |
| 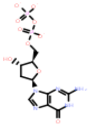 | 0 | 3155 | C10H15N5O10P2 | -14.71 | 39 | 7 | -13.347 | -3.1279 | -22.75 | 0 | 26.161 | 30.437 | -92.93916 | 1.31929 | 1 |
| 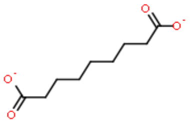 | 0 | 435 | 548 | -14.65 | 27 | 8 | -11.725 | -3.0306 | -14.12 | 4.6229 | 16.065 | 17.49 | -90.91495 | 1.14802 | 1 |
| 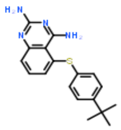 | 0 | 1722 | TQ5 | -14.65 | 43 | 1 | -6.2979 | -5.8191 | -19.74 | 2.8867 | 19.998 | 11.504 | -99.29034 | 1.36741 | 1 |
| 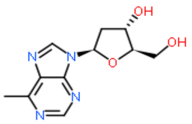 | 0 | 3376 | C11H14N4O3 | -14.64 | 32 | 4 | -6.6062 | -4.6225 | -17.53 | 1.4288 | 19.308 | 7.517 | -97.32131 | 1.2683 | 1 |
| 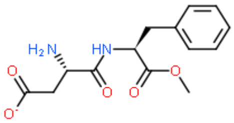 | 0 | 63 | C14H18N2O5 | -14.63 | 38 | 8 | -10.323 | -4.0771 | -17.17 | 3.1073 | 19.442 | 15.357 | -75.29862 | 1.22216 | 1 |
| 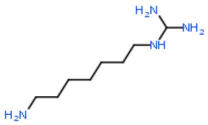 | 0 | 3288 | GC7 | -14.62 | 34 | 11 | -9.0593 | -2.8663 | -20.78 | 2.2021 | 21.022 | 11.073 | -32.84678 | 0.80237 | 1 |
| 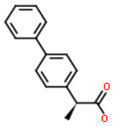 | 0 | 1807 | BFL | -14.62 | 30 | 3 | -2.8102 | -3.9402 | -15.07 | 0.8621 | 12.138 | -1.855 | -73.27143 | 1.31786 | 1 |

| | | | | | | | | | | | | | | | |
|---|---|---|---|---|---|---|---|---|---|---|---|---|---|---|---|
| 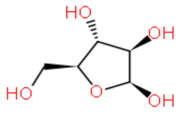 | 0 | 2927 | C5H10O5 | -14.6 | 20 | 5 | -13.22 | -1.9781 | -7.136 | 8.0732 | 16.366 | 10.889 | -63.97067 | 0.50828 | 1 |
| 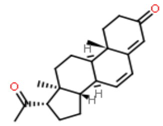 | 0 | 270 | 378 | -14.59 | 51 | 1 | -2.6702 | -4.7536 | -20.64 | 2.7957 | 10.807 | 8.4886 | -85.83367 | 1.04522 | 1 |
| 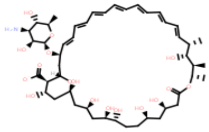 | 0 | 567 | m | -14.58 | 137 | 14 | -14.568 | -10.127 | -41.85 | 26.216 | 42.084 | 23.064 | -250.7991 | 4.05037 | 1 |
| 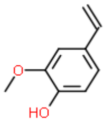 | 0 | 3177 | C9H10O2 | -14.57 | 21 | 0 | -4.0333 | -3.705 | -16.96 | 0.2339 | 8.8347 | 12.591 | -48.43895 | 0.83549 | 1 |
| 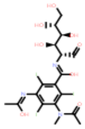 | 0 | 1374 | C18H22I3N3O8 | -14.57 | 54 | 13 | -10.58 | -5.0102 | -24.84 | 5.849 | 25.971 | 14.55 | -89.19159 | 1.96033 | 1 |
| 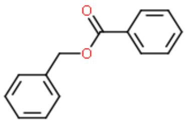 | 0 | 562 | 676 | -14.55 | 28 | 3 | -4.013 | -4.7511 | -19.22 | 1.8226 | 11.645 | 9.6603 | -80.25473 | 1.2495 | 1 |
| 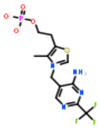 | 0 | 1998 | C12H13F3N4O4PS | -14.55 | 38 | 6 | -11.261 | -5.4146 | -20.28 | 0 | 19.051 | 30.245 | -119.6813 | 1.5495 | 1 |
| 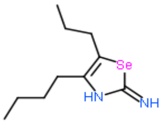 | 0 | 1524 | C10H18N2Se | -14.52 | 31 | 5 | -6.9491 | -3.0687 | -19.66 | 2.0645 | 14.755 | 14.342 | -21.26766 | 0.88142 | 1 |
| 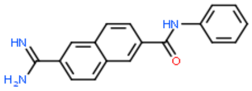 | 0 | 1741 | 675 | -14.51 | 37 | 1 | -6.5737 | -5.2159 | -22.6 | 4.3554 | 19.017 | 16.274 | -123.3289 | 1.57592 | 1 |
| 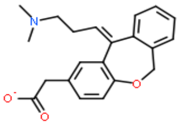 | 0 | 651 | 768 | -14.45 | 47 | 5 | -8.4745 | -5.5293 | -23.44 | 6.337 | 16.953 | 22.146 | -110.3642 | 1.678 | 1 |
| 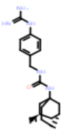 | 0 | 3420 | AGB | -14.45 | 52 | 6 | -5.688 | -4.95 | -26.22 | 4.5442 | 21.334 | 10.831 | -102.2063 | 1.53047 | 1 |

| | | | | | | | | | | | | | | | |
|---|---|---|---|---|---|---|---|---|---|---|---|---|---|---|---|
| 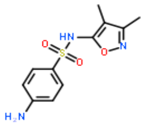 | 0 | 158 | 263 | -14.44 | 31 | 3 | -5.3033 | -3.548 | -20.57 | 1.6731 | 12.292 | 15 | -74.95256 | 1.20291 | 1 |
| 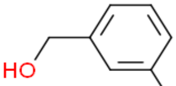 | 0 | 3014 | C7H7IO | -14.44 | 16 | 2 | -5.0074 | -3.6831 | -13.25 | 1.5172 | 10.322 | 6.2067 | -36.9363 | 0.74584 | 1 |
| 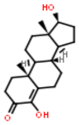 | 0 | 1288 | C19H28O3 | -14.44 | 50 | 1 | -5.623 | -4.1969 | -19.04 | 1.4689 | 13.375 | 15.348 | -99.1297 | 1.0727 | 1 |
| 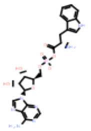 | 0 | 1608 | 446202 | -14.43 | 60 | 11 | -18.355 | -4.6218 | -29.49 | 17.516 | 37.328 | 29.531 | -164.6007 | 1.88005 | 1 |
| 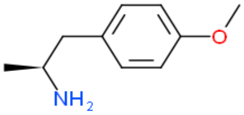 | 0 | 1275 | 1472 | -14.43 | 27 | 3 | -5.7272 | -4.0573 | -17.49 | 4.8907 | 14.695 | 6.8361 | -58.04884 | 1.04488 | 1 |
| 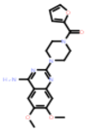 | 0 | 347 | 457 | -14.42 | 49 | 1 | -5.5062 | -7.0814 | -25.13 | 4.6855 | 20.371 | 16.199 | -131.1897 | 1.53819 | 1 |
| 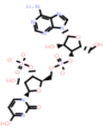 | 0 | 1570 | C19H25N7O15P2 | -14.42 | 65 | 14 | -16.558 | -4.5284 | -28.6 | 12.258 | 34.399 | 26.585 | -186.9505 | 2.26958 | 1 |
| 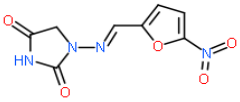 | 0 | 584 | C8H6N4O5 | -14.38 | 23 | 1 | -4.7196 | -2.5661 | -25.24 | 2.6388 | 14.26 | 17.639 | -69.47747 | 1.03153 | 1 |
| 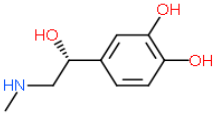 | 0 | 554 | 668 | -14.36 | 26 | 4 | -8.323 | -3.3447 | -15.32 | 2.7557 | 16.664 | 11.675 | -72.27214 | 1.24107 | 1 |
| 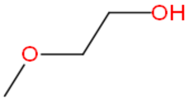 | 0 | 2511 | C3H8O2 | -14.34 | 13 | 3 | -7.6006 | -2.3734 | -7.352 | 0 | 9.5956 | 7.3238 | -27.80305 | 0.33827 | 1 |
| 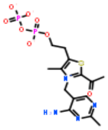 | 0 | 2146 | Structure | -14.34 | 47 | 8 | -10.096 | -5.5713 | -26.14 | 6.2686 | 23.288 | 22.807 | -99.99406 | 1.34144 | 1 |

| | | | | | | | | | | | | | | | |
|---|---|---|---|---|---|---|---|---|---|---|---|---|---|---|---|
| 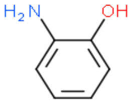 | 0 | 1511 | C6H7NO | -14.34 | 15 | 0 | -5.2679 | -2.4853 | -11.59 | 0.865 | 11.079 | 5.1821 | -29.75472 | 0.43444 | 1 |
| 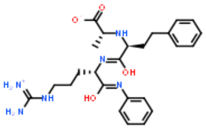 | 0 | 2459 | C25H35N6O4 | -14.33 | 69 | 16 | -7.3029 | -6.435 | -31.52 | 0 | 23.222 | 19.633 | -81.28877 | 1.71161 | 1 |
| 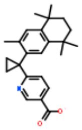 | 0 | 1706 | C24H29NO2 | -14.32 | 55 | 3 | -7.1323 | -5.8377 | -18.86 | 3.8362 | 14.702 | 17.229 | -90.8775 | 1.36416 | 1 |
| 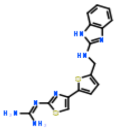 | 0 | 2339 | FR0 | -14.31 | 40 | 2 | -3.6134 | -6.3358 | -27.47 | 0 | 19.016 | 16.798 | -104.4701 | 1.46046 | 1 |
| 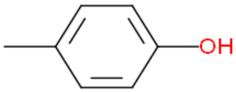 | 0 | 1476 | C7H8O | -14.31 | 16 | 0 | -4.6833 | -2.9385 | -11.49 | 0.5938 | 9.2552 | 5.602 | -31.77879 | 0.54959 | 1 |
| 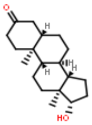 | 0 | 2601 | Untitled | -14.31 | 51 | 1 | -5.7479 | -4.5741 | -17.03 | 1.1978 | 13.439 | 13.524 | -103.2382 | 1.06557 | 1 |
| 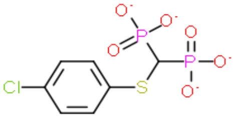 | 0 | 1004 | 1133 | -14.31 | 22 | 4 | -14.096 | -2.7742 | -16.77 | 1.1747 | 19.25 | 34.001 | -76.38801 | 1.04585 | 1 |
| 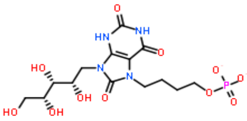 | 0 | 2426 | C14H23N4O11P | -14.3 | 51 | 15 | -15.88 | -3.8559 | -19.93 | 2.7932 | 30.336 | 22.674 | -97.25918 | 1.57959 | 1 |
| 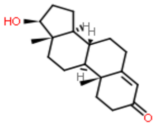 | 0 | 511 | C19H28O2 | -14.29 | 49 | 1 | -5.7102 | -4.3865 | -17.89 | 1.1897 | 13.621 | 14.317 | -97.76568 | 1.06219 | 1 |
| 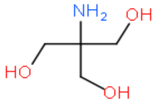 | 0 | 3394 | Structure | -14.29 | 19 | 7 | -8.3616 | -1.7576 | -10.54 | 0 | 12.025 | 8.1189 | -53.59016 | 0.48037 | 1 |
| 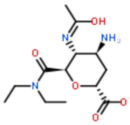 | 0 | 2259 | C13H23N3O5 | -14.29 | 43 | 8 | -7.4502 | -3.8968 | -18.42 | 0 | 18.682 | 10.261 | -120.7924 | 1.52176 | 1 |

| | | | | | | | | | | | | | | | |
|---|---|---|---|---|---|---|---|---|---|---|---|---|---|---|---|
| 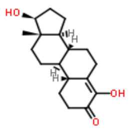 | 0 | 1302 | C18H26O3 | -14.28 | 47 | 1 | -5.5394 | -4.1483 | -18.87 | 1.1838 | 13.443 | 15.14 | -96.91626 | 1.08235 | 1 |
| 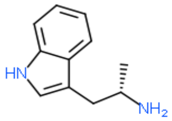 | 0 | 1252 | 1446 | -14.27 | 27 | 3 | -4.6962 | -3.8561 | -17.52 | 3.7058 | 12.844 | 6.15 | -52.41525 | 0.89929 | 1 |
| 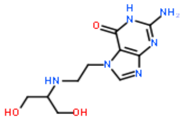 | 0 | 2128 | C10H16N6O3 | -14.22 | 35 | 8 | -11.286 | -3.4267 | -21.39 | 5.5217 | 22.381 | 19.884 | -105.2919 | 1.31131 | 1 |
| 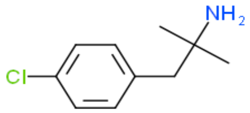 | 0 | 1352 | 1556 | -14.21 | 26 | 3 | -3.7793 | -4.0674 | -15.64 | 0.523 | 10.762 | 5.3443 | -57.26644 | 1.02241 | 1 |
| 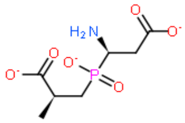 | 0 | 3015 | C7H14NO6P | -14.2 | 26 | 7 | -10.892 | -2.2296 | -13.11 | 0 | 17.849 | 15.715 | -75.86919 | 0.99362 | 1 |
| 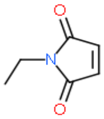 | 0 | 2663 | C6H7NO2 | -14.2 | 16 | 1 | -4.7382 | -2.7501 | -13.35 | 0.0252 | 8.0149 | 9.6295 | -52.19508 | 0.55185 | 1 |
| 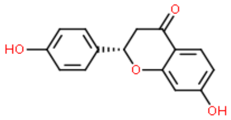 | 0 | 3252 | C15H12O4 | -14.17 | 31 | 1 | -5.7639 | -4.1165 | -21.8 | 1.7514 | 17.904 | 14.977 | -113.6472 | 1.36013 | 1 |
| 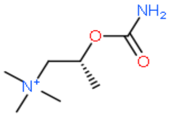 | 0 | 895 | C7H17N2O2 | -14.14 | 28 | 3 | -6.5993 | -3.2137 | -13.2 | 0.084 | 12.116 | 10.566 | -54.2995 | 0.88616 | 1 |
| 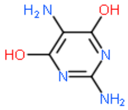 | 0 | 1798 | C4H6N4O2 | -14.12 | 16 | 0 | -10.972 | -2.0052 | -9.288 | 0.4706 | 19.437 | 14.26 | -31.3584 | 0.49747 | 1 |
| 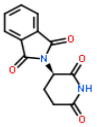 | 0 | 915 | 1041 | -14.12 | 29 | 1 | -6.9791 | -3.0708 | -17.92 | 2.0277 | 15.419 | 15.358 | -87.03075 | 1.01273 | 1 |
| 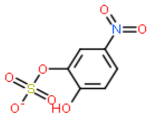 | 0 | 1578 | CSN | -14.12 | 19 | 2 | -6.8995 | -1.8981 | -15.32 | 0 | 14.606 | 11.786 | -84.52843 | 1.09544 | 1 |

| | | | | | | | | | | | | | | | |
|---|---|---|---|---|---|---|---|---|---|---|---|---|---|---|---|
| 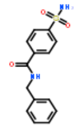 | 0 | 1531 | BSB | -14.1 | 34 | 4 | -4.4626 | -4.5019 | -26.51 | 2.8693 | 15.993 | 16.052 | -121.2389 | 1.25976 | 1 |
| 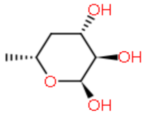 | 0 | 1618 | C6H12O4 | -14.1 | 22 | 3 | -9.2251 | -1.7228 | -10.61 | 4.8881 | 14.28 | 8.4234 | -20.20678 | 0.58666 | 1 |
| 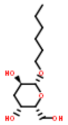 | 0 | 3411 | DLG | -14.09 | 41 | 10 | -8.1619 | -3.6184 | -14.4 | 7.366 | 11.775 | 6.2618 | -40.10984 | 0.90137 | 1 |
| 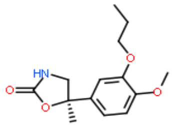 | 0 | 1439 | 5RM | -14.06 | 38 | 3 | -7.9931 | -4.8042 | -21.15 | 5.1771 | 17.551 | 18.885 | -129.7032 | 1.32726 | 1 |
| 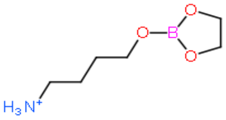 | 0 | 2050 | C6H15BNO3 | -14.06 | 26 | 4 | -5.7533 | -2.336 | -13.98 | 0.7885 | 11.295 | 7.1221 | -13.00955 | 1.03771 | 1 |
| 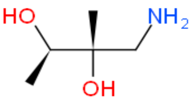 | 0 | 2550 | C5H13NO2 | -14.06 | 21 | 5 | -8.0108 | -2.3808 | -11.36 | 3.5183 | 10.518 | 9.3503 | -59.59065 | 0.58771 | 1 |
| 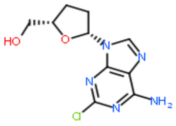 | 0 | 1861 | CDY | -14.05 | 30 | 3 | -8.6363 | -4.3431 | -13.51 | 0 | 18.066 | 13.445 | -96.43286 | 1.1962 | 1 |
| 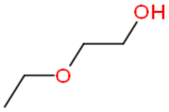 | 0 | 1994 | C4H10O2 | -14.05 | 16 | 4 | -7.0809 | -2.7897 | -7.989 | 0 | 9.4119 | 6.4666 | -41.39475 | 0.48507 | 1 |
| 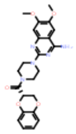 | 0 | 477 | 590 | -14.01 | 58 | 2 | -8.3827 | -7.9875 | -27.86 | 5.4467 | 26.482 | 24.355 | -153.6059 | 2.07403 | 1 |
| 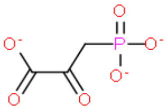 | 0 | 2252 | C3H5O6P | -14.01 | 12 | 3 | -11.325 | -1.0384 | -10.56 | 0.7151 | 15.543 | 18.195 | -58.25663 | 0.56706 | 1 |
| 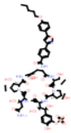 | 0 | 1012 | C56H71N9O23S | -13.97 | 159 | 21 | -16.217 | -10.849 | -50.7 | 37.621 | 39.58 | 30.568 | -234.1682 | 4.44085 | 1 |

| | | | | | | | | | | | | | | | |
|---|---|---|---|---|---|---|---|---|---|---|---|---|---|---|---|
| 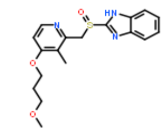 | 0 | 1000 | 1129 | -13.96 | 46 | 7 | -8.5353 | -6.7855 | -22.54 | 4.9621 | 20.89 | 17.994 | -124.1859 | 1.68917 | 1 |
| 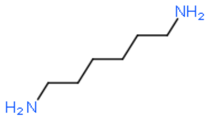 | 0 | 2941 | C6H16N2 | -13.91 | 24 | 7 | -8.6302 | -2.0783 | -12.99 | 1.8471 | 14.438 | 9.3552 | -19.24415 | 0.63442 | 1 |
| 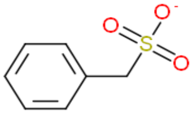 | 0 | 2974 | C7H8O3S | -13.86 | 18 | 2 | -6.328 | -2.8389 | -13.72 | 1.11 | 11.421 | 11.123 | -63.85255 | 0.78039 | 1 |
| 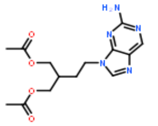 | 0 | 316 | 426 | -13.84 | 42 | 7 | -8.0444 | -5.549 | -25.77 | 4.3485 | 22.917 | 18.194 | -88.91924 | 1.41541 | 1 |
| 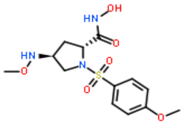 | 0 | 1650 | C13H19N3O6S | -13.83 | 42 | 7 | -9.3617 | -3.4215 | -23.15 | 5.2342 | 22.218 | 17.11 | -40.04294 | 1.39986 | 1 |
| 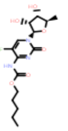 | 0 | 973 | 1101 | -13.83 | 47 | 8 | -10.796 | -4.9175 | -26.21 | 15.429 | 21.668 | 19.224 | -103.9559 | 1.72721 | 1 |
| 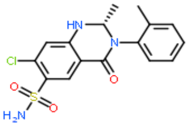 | 0 | 411 | 524 | -13.83 | 40 | 2 | -3.1352 | -4.7942 | -27.36 | 3.4088 | 17.507 | 12.585 | -110.2203 | 1.36674 | 1 |
| 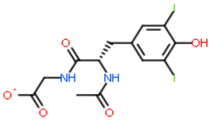 | 0 | 2323 | C13H14I2N2O5 | -13.82 | 35 | 6 | -10.804 | -4.9985 | -21.11 | 8.3279 | 22.127 | 19.365 | -91.55001 | 1.39293 | 1 |
| 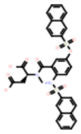 | 0 | 1652 | C32H26N2O10S2 | -13.82 | 70 | 10 | -8.4053 | -5.473 | -39.14 | 12.157 | 24.711 | 27.922 | -134.6828 | 1.94497 | 1 |
| 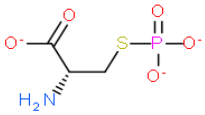 | 0 | 3202 | C3H8NO5PS | -13.79 | 16 | 5 | -12.105 | -1.2051 | -11.18 | 0 | 20.291 | 16.061 | -53.89376 | 0.7155 | 1 |
| 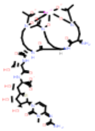 | 0 | 2438 | C37H57FeN12O18S | -13.78 | 125 | 14 | -8.5381 | -7.6102 | -36.93 | 8.918 | 32.217 | 18.079 | -139.908 | 2.75972 | 1 |

| | | | | | | | | | | | | | | | |
|---|---|---|---|---|---|---|---|---|---|---|---|---|---|---|---|
| 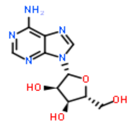 | 0 | 527 | 640 | -13.78 | 32 | 5 | -8.2728 | -3.9953 | -15.79 | 0.2191 | 23.281 | 7.6151 | -100.5952 | 1.32226 | 1 |
| 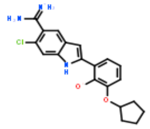 | 0 | 1551 | 991 | -13.77 | 46 | 1 | -4.3406 | -4.4049 | -24.58 | 2.9699 | 17.463 | 14.031 | -13.35932 | 1.12189 | 1 |
| 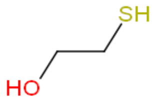 | 0 | 3020 | C2H6OS | -13.77 | 10 | 3 | -7.61 | -1.3881 | -8.299 | 2.5467 | 9.818 | 6.0984 | -16.63004 | 0.30031 | 1 |
| 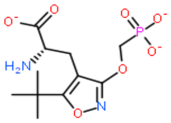 | 0 | 2087 | C11H19N2O7P | -13.76 | 37 | 7 | -7.3609 | -2.6848 | -16.12 | 0.5257 | 20.513 | 4.3743 | -39.50503 | 1.35301 | 1 |
| 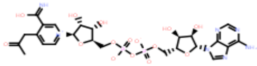 | 0 | 2446 | C24H31N7O15P2 | -13.71 | 78 | 17 | -8.3943 | -3.4952 | -27.36 | 4.4071 | 28.042 | 5.0717 | -54.43646 | 1.9329 | 1 |
| 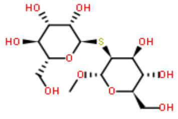 | 0 | 2158 | C13H24O10S | -13.71 | 48 | 12 | -14.179 | -3.3033 | -12.63 | 3.079 | 22.727 | 16.532 | -104.2126 | 1.59597 | 1 |
| 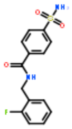 | 0 | 1827 | FSB | -13.71 | 34 | 4 | -2.7366 | -4.4704 | -23.81 | 1.6505 | 14.444 | 8.7759 | -70.34858 | 1.35012 | 1 |
| 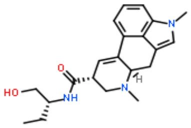 | 0 | 142 | C21H27N3O2 | -13.7 | 53 | 5 | -9.4614 | -6.8488 | -22.8 | 5.0726 | 19.6 | 25.579 | -129.1391 | 1.47931 | 1 |
| 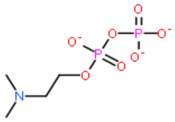 | 0 | 2847 | C4H13NO7P2 | -13.68 | 24 | 6 | -13.035 | -2.5844 | -14.38 | 0.0059 | 19.112 | 26.471 | -70.48315 | 0.86288 | 1 |
| 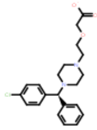 | 0 | 234 | 341 | -13.67 | 51 | 8 | -9.2412 | -6.6131 | -25.87 | 9.5706 | 20.81 | 21.006 | -124.0012 | 1.75189 | 1 |
| 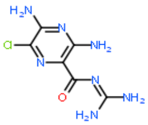 | 0 | 481 | 594 | -13.65 | 23 | 0 | -7.7327 | -2.9308 | -20.34 | 3.1193 | 22.13 | 14.771 | -58.45585 | 0.858 | 1 |

| | | | | | | | | | | | | | | | |
|---|---|---|---|---|---|---|---|---|---|---|---|---|---|---|---|
| 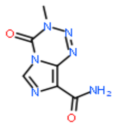 | 0 | 733 | 853 | -13.65 | 20 | 0 | -8.4193 | -2.7176 | -16.09 | 0.4865 | 19.114 | 16.568 | -76.84995 | 1.06594 | 1 |
| 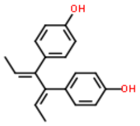 | 0 | 770 | C18H18O2 | -13.65 | 38 | 0 | -6.6663 | -5.0897 | -21.33 | 4.0329 | 17.309 | 19.03 | -108.2275 | 1.40602 | 1 |
| 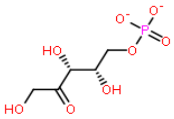 | 0 | 1689 | C5H11O8P | -13.61 | 23 | 9 | -10.938 | -1.8971 | -11.92 | 0 | 16.047 | 14.873 | -77.77634 | 0.90417 | 1 |
| 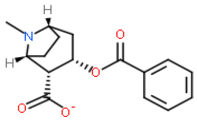 | 0 | 1313 | 1515 | -13.59 | 39 | 3 | -6.558 | -4.7359 | -18.54 | 1.6777 | 12.684 | 18.774 | -72.00105 | 1.20938 | 1 |
| 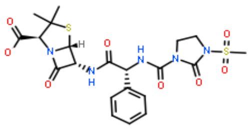 | 0 | 826 | 948 | -13.59 | 60 | 8 | -1.8812 | -6.448 | -35.29 | 4.5001 | 21.24 | 11.135 | -133.6229 | 1.72677 | 1 |
| 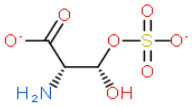 | 0 | 2449 | C3H7NO7S | -13.58 | 17 | 6 | -9.3066 | -1.2064 | -10.78 | 0 | 18.824 | 5.8124 | -75.57568 | 0.74239 | 1 |
| 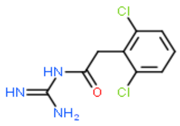 | 0 | 894 | m | -13.58 | 24 | 3 | -5.635 | -2.9446 | -17.83 | 0 | 13.091 | 13.421 | -48.03224 | 0.90477 | 1 |
| 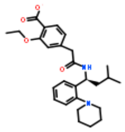 | 0 | 791 | 912 | -13.57 | 68 | 8 | -9.1913 | -7.2679 | -30.2 | 7.3726 | 23.509 | 27.12 | -150.7698 | 2.04431 | 1 |
| 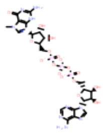 | 0 | 1441 | C21H30N10O17P3 | -13.57 | 78 | 16 | -20.119 | -4.2887 | -30.97 | 18.349 | 37.614 | 33.872 | -176.6621 | 2.5279 | 1 |
| 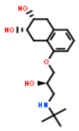 | 0 | 1073 | 1203 | -13.56 | 49 | 8 | -10.102 | -5.5469 | -15.52 | 4.726 | 15.987 | 17.586 | -102.6959 | 1.61886 | 1 |
| 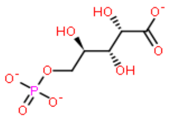 | 0 | 2733 | C5H11O9P | -13.55 | 23 | 9 | -11.932 | -1.4465 | -14.08 | 0 | 17.787 | 19.614 | -81.93851 | 1.08219 | 1 |

| | | | | | | | | | | | | | | | |
|---|---|---|---|---|---|---|---|---|---|---|---|---|---|---|---|
| 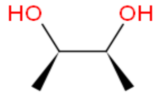 | 0 | 2154 | C4H10O2 | -13.53 | 16 | 3 | -6.8215 | -2.5055 | -9.774 | 2.05 | 10.255 | 6.7527 | -40.09742 | 0.41984 | 1 |
| 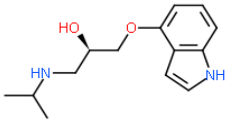 | 0 | 838 | 960 | -13.52 | 38 | 6 | -5.5795 | -4.9405 | -18.72 | 1.98 | 14.837 | 10.16 | -86.40112 | 1.21932 | 1 |
| 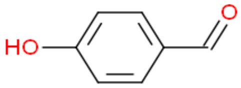 | 0 | 3216 | C7H6O2 | -13.52 | 15 | 0 | -3.4505 | -1.5863 | -13.23 | 0.3076 | 6.8439 | 6.3397 | -24.77472 | 0.4282 | 1 |
| 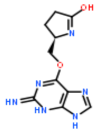 | 0 | 2598 | C10H12N6O2 | -13.51 | 30 | 3 | -9.4588 | -3.3477 | -16.91 | 0.9211 | 21.237 | 17.114 | -80.1619 | 1.23113 | 1 |
| 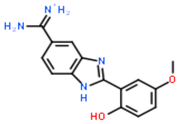 | 0 | 1674 | 123 | -13.49 | 36 | 0 | -6.6893 | -4.629 | -24.32 | 1.7523 | 18.53 | 24.089 | -104.7329 | 1.5771 | 1 |
| 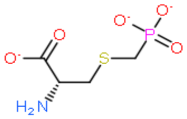 | 0 | 2194 | C4H10NO5PS | -13.49 | 19 | 6 | -10.614 | -2.0114 | -12.46 | 0 | 17.512 | 15.813 | -57.70954 | 0.78569 | 1 |
| 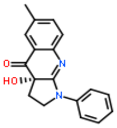 | 0 | 1708 | BIT | -13.49 | 38 | 2 | -6.6136 | -5.0655 | -22.35 | 4.4803 | 17.059 | 18.64 | -131.5414 | 1.7692 | 1 |
| 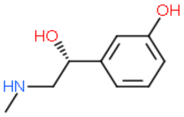 | 0 | 279 | 388 | -13.47 | 25 | 4 | -4.732 | -3.7269 | -15.59 | 0.1178 | 13.055 | 6.5541 | -40.06893 | 0.90941 | 1 |
| 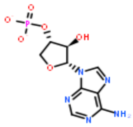 | 0 | 2187 | C9H12N5O6P | -13.47 | 31 | 4 | -12.628 | -3.444 | -16.86 | 3.5844 | 23.972 | 22.913 | -99.59731 | 1.20583 | 1 |
| 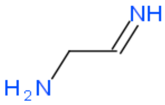 | 0 | 1866 | C2H6N2 | -13.42 | 10 | 2 | -7.1317 | -0.6658 | -11.1 | 2.182 | 9.3096 | 10.113 | -2.516649 | 0.00836 | 1 |
| 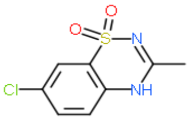 | 0 | 990 | 1119 | -13.41 | 21 | 0 | -2.1687 | -3.4908 | -19.31 | 0.3723 | 10.492 | 8.5275 | -66.63664 | 1.11097 | 1 |

| | | | | | | | | | | | | | | | |
|---|---|---|---|---|---|---|---|---|---|---|---|---|---|---|---|
| 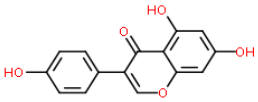 | 0 | 1437 | Genistein | -13.41 | 30 | 0 | -7.073 | -3.627 | -18.91 | 0 | 18.316 | 18.318 | -113.8161 | 1.25323 | 1 |
| 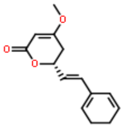 | 0 | 1157 | C14H16O3 | -13.4 | 33 | 1 | -5.6794 | -4.9088 | -19.19 | 1.0366 | 14.929 | 16.718 | -66.46903 | 1.19914 | 1 |
| 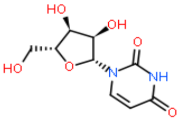 | 0 | 2457 | Tyramine.mol | -13.39 | 29 | 5 | -7.6759 | -2.445 | -15.37 | 0 | 16.484 | 11.632 | -116.6685 | 1.25237 | 1 |
| 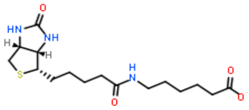 | 0 | 2822 | BH7 | -13.39 | 50 | 11 | -8.9354 | -5.1405 | -25.27 | 4.8417 | 20.257 | 20.057 | -138.915 | 1.55169 | 1 |
| 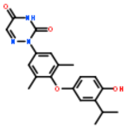 | 0 | 2863 | PFA | -13.38 | 48 | 3 | -6.8609 | -5.1395 | -26.27 | 10.021 | 21.686 | 14.566 | -138.2522 | 1.97942 | 1 |
| 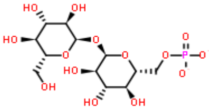 | 0 | 2166 | C12H23O14P | -13.38 | 48 | 13 | -13.126 | -2.6495 | -13.17 | 1.9756 | 23.016 | 12.922 | -106.9149 | 1.29429 | 1 |
| 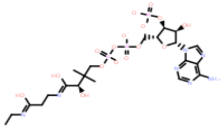 | 0 | 1606 | C21H36N7O16P3 | -13.38 | 79 | 21 | -16.4 | -6.0351 | -31.98 | 0 | 35.633 | 37.59 | -182.065 | 2.69764 | 1 |
| 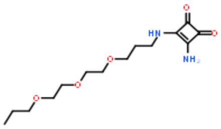 | 0 | 2766 | LNQ | -13.37 | 45 | 12 | -9.5715 | -4.8263 | -24.73 | 5.4797 | 17.846 | 22.629 | -94.49115 | 1.67789 | 1 |
| 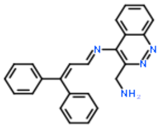 | 0 | 2583 | C24H20N4 | -13.31 | 48 | 4 | -5.1756 | -6.9437 | -30 | 2.9998 | 18.678 | 24.592 | -133.4317 | 1.50555 | 1 |
| 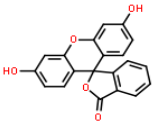 | 0 | 579 | C20H12O5 | -13.29 | 37 | 0 | -5.8655 | -4.6882 | -22.71 | 3.8468 | 18.257 | 17.276 | -127.7828 | 1.42588 | 1 |
| 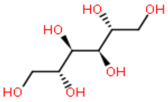 | 0 | 625 | C6H14O6 | -13.27 | 26 | 11 | -10.412 | -2.1649 | -11.64 | 0 | 18.476 | 8.6465 | -77.13928 | 0.88029 | 1 |

| | | | | | | | | | | | | | | | |
|---|---|---|---|---|---|---|---|---|---|---|---|---|---|---|---|
| 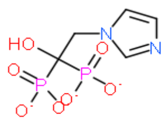 | 0 | 290 | 68740 | -13.24 | 22 | 5 | -13.78 | -2.2755 | -16.12 | 1.1275 | 19.988 | 31.213 | -83.4196 | 0.77579 | 1 |
| 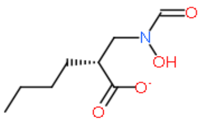 | 0 | 2348 | C8H15NO4 | -13.23 | 27 | 8 | -9.9752 | -2.9865 | -12.02 | 0 | 13.237 | 17.12 | -86.17839 | 1.14574 | 1 |
| 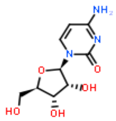 | 0 | 1855 | Cytidine.mol | -13.18 | 30 | 5 | -11.33 | -2.4745 | -18.07 | 2.4506 | 23.501 | 19.871 | -93.31426 | 1.07847 | 1 |
| 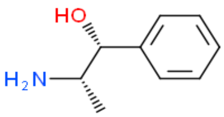 | 0 | 288 | C9H13NO | -13.12 | 24 | 4 | -5.9626 | -3.3259 | -13.85 | 3.1829 | 12.64 | 6.4458 | -56.20419 | 0.86368 | 1 |
| 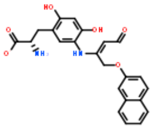 | 0 | 2241 | NBQ | -13.12 | 52 | 6 | -4.4825 | -5.3025 | -29.16 | 0.3445 | 22.986 | 14.984 | -115.8629 | 1.88333 | 1 |
| 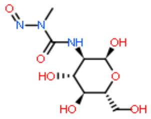 | 0 | 318 | 428 | -13.1 | 33 | 8 | -11.658 | -3.1056 | -18.91 | 3.6095 | 25.882 | 16.673 | -89.2145 | 1.25698 | 1 |
| 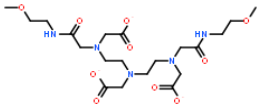 | 0 | 425 | 538 | -13.1 | 69 | 22 | -15.013 | -6.0636 | -30.97 | 10.9 | 25.643 | 31.727 | -130.875 | 2.05693 | 1 |
| 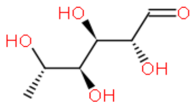 | 0 | 2657 | C6H12O5 | -13.09 | 23 | 8 | -10.018 | -1.9021 | -10.64 | 3.2126 | 15.892 | 8.3673 | -41.86173 | 0.67716 | 1 |
| 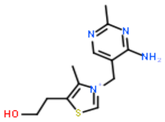 | 0 | 48 | 152 | -13.07 | 35 | 5 | -6.8694 | -4.7847 | -19 | 4.9008 | 15.3 | 13.786 | -58.36606 | 0.85493 | 1 |
| 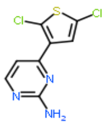 | 0 | 2713 | C8H5Cl2N3S | -13.06 | 19 | 0 | -5.2417 | -4.6716 | -16.5 | 1.7581 | 14.298 | 12.21 | -50.60485 | 1.00057 | 1 |
| 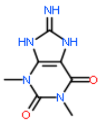 | 0 | 1558 | C7H9N5O2 | -13.06 | 23 | 0 | -4.0611 | -2.513 | -16.15 | 0 | 15.486 | 5.2079 | -61.74183 | 1.02031 | 1 |

| | | | | | | | | | | | | | | | |
|---|---|---|---|---|---|---|---|---|---|---|---|---|---|---|---|
| 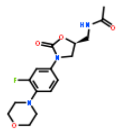 | 0 | 488 | 601 | -13.05 | 44 | 2 | -6.5874 | -5.3649 | -22.16 | 1.9863 | 19.772 | 18.4 | -123.3009 | 1.74308 | 1 |
| 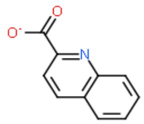 | 0 | 2164 | QND | -13.03 | 19 | 1 | -5.6467 | -2.9412 | -12.83 | 0.2984 | 12.433 | 9.0158 | -68.60728 | 0.82318 | 1 |
| 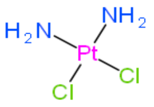 | 0 | 403 | H6Cl2N2Pt | -13.03 | 11 | 0 | -5.7565 | -0.9838 | -9.47 | 0.0399 | 11.328 | 4.7693 | -5.742145 | 0.43413 | 1 |
| 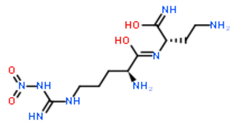 | 0 | 1598 | C10H22N8O4 | -13.02 | 44 | 16 | -13.242 | -2.792 | -22.84 | 3.6969 | 28.583 | 18.091 | -57.59754 | 1.48565 | 1 |
| 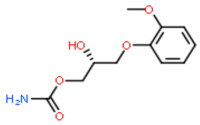 | 0 | 313 | C11H15NO5 | -13.01 | 32 | 5 | -9.106 | -3.8627 | -17.83 | 2.9107 | 20.845 | 15.25 | -108.0193 | 1.45635 | 1 |
| 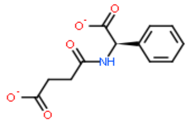 | 0 | 2976 | NPG | -13.01 | 29 | 6 | -11.427 | -3.3541 | -14.22 | 4.8012 | 18.397 | 18.324 | -79.48463 | 1.23726 | 1 |
| 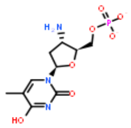 | 0 | 2914 | C10H16N3O7P | -13 | 35 | 6 | -13.739 | -2.2799 | -11.98 | 6.2036 | 20.563 | 18.966 | -79.2519 | 1.25146 | 1 |
| 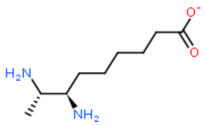 | 0 | 1501 | C9H20N2O2 | -12.98 | 32 | 9 | -13.432 | -3.0945 | -7.945 | 5.5741 | 18.107 | 13.179 | -64.86671 | 0.99966 | 1 |
| 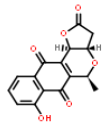 | 0 | 1457 | NOM | -12.96 | 34 | 0 | -5.2155 | -3.6937 | -21.25 | 0 | 17.111 | 16.862 | -110.8205 | 1.47413 | 1 |
| 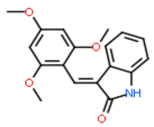 | 0 | 2771 | 5288600 | -12.96 | 40 | 0 | -3.8588 | -5.4134 | -23.43 | 3.3284 | 14.562 | 16.582 | -83.73215 | 0.96139 | 1 |
| 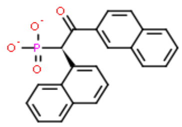 | 0 | 2099 | KTP | -12.96 | 42 | 3 | -10.338 | -4.9861 | -21 | 13.454 | 16.062 | 23.67 | -135.136 | 1.55098 | 1 |

| | | | | | | | | | | | | | | |
|---|---|---|---|---|---|---|---|---|---|---|---|---|---|---|
| 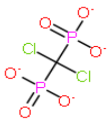 | 0 | 604 | CH4Cl2O6P2 | -12.94 | 11 | 2 | -12.776 | -1.6573 | -11.92 | 0 | 15.454 | 29.734 | -52.18788 | 0.49479 | 1 |
| 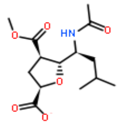 | 0 | 2938 | ABW | -12.92 | 43 | 6 | -9.0894 | -4.364 | -17.94 | 7.2311 | 18.768 | 13.523 | -115.7766 | 1.37935 | 1 |
| 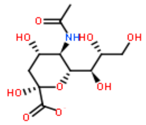 | 0 | 3362 | N-Acetyl-a-neuraminic | -12.91 | 39 | 10 | -12.49 | -2.8468 | -13.5 | 0.5236 | 23.217 | 15.712 | -116.3307 | 1.24135 | 1 |
| 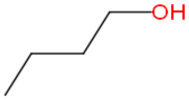 | 0 | 1902 | C4H10O | -12.91 | 15 | 3 | -6.7414 | -2.6155 | -9.331 | 1.428 | 10.184 | 7.4815 | -35.81138 | 0.53364 | 1 |
| 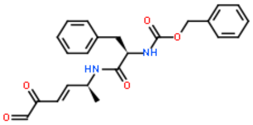 | 0 | 1587 | 186 | -12.87 | 54 | 10 | -5.3652 | -6.1229 | -34.95 | 4.4639 | 22.852 | 21.133 | -134.0927 | 1.91033 | 1 |
| 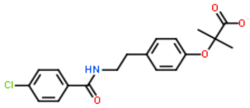 | 0 | 1204 | 1393 | -12.86 | 44 | 6 | -4.3777 | -6.4093 | -26.11 | 4.9191 | 16.76 | 14.403 | -127.9526 | 1.37624 | 1 |
| 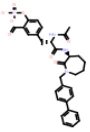 | 0 | 1467 | C31H34N3O8P | -12.85 | 75 | 9 | -14.055 | -6.5869 | -28.73 | 16.813 | 29.178 | 27.997 | -168.3543 | 2.18845 | 1 |
| 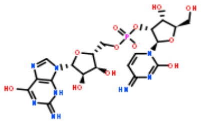 | 0 | 3287 | C19H25N8O12P | -12.84 | 64 | 14 | -12.025 | -3.9756 | -30.63 | 1.5695 | 35.33 | 22.782 | -147.1848 | 2.0891 | 1 |
| 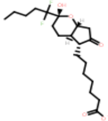 | 0 | 920 | C20H32F2O5 | -12.82 | 58 | 12 | -10.022 | -6.2797 | -24.77 | 6.5943 | 21.538 | 21.516 | -146.9405 | 1.58557 | 1 |
| 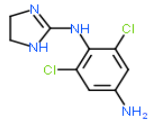 | 0 | 842 | 964 | -12.81 | 25 | 1 | -3.1265 | -3.7662 | -18.35 | 0.4217 | 13.018 | 8.1176 | -15.34956 | 0.74732 | 1 |
| 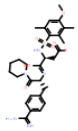 | 0 | 2769 | C29H39N5O7S | -12.8 | 80 | 12 | -6.5508 | -7.5859 | -33.33 | 9.1784 | 25.478 | 15.702 | -170.3175 | 2.39938 | 1 |

| | | | | | | | | | | | | | | | |
|---|---|---|---|---|---|---|---|---|---|---|---|---|---|---|---|
| 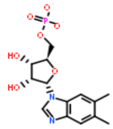 | 0 | 1791 | C14H19N2O7P | -12.8 | 41 | 6 | -4.4272 | -4.8179 | -18.69 | 0 | 17.158 | 5.8347 | -102.4968 | 1.61913 | 1 |
| 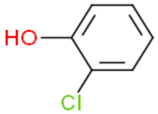 | 0 | 2797 | C6H5ClO | -12.8 | 13 | 0 | -4.4016 | -2.9709 | -12.31 | 0.3658 | 9.3645 | 8.1757 | -26.7471 | 0.57836 | 1 |
| 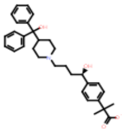 | 0 | 828 | C32H39NO4 | -12.8 | 75 | 12 | -9.3555 | -8.3341 | -30.23 | 6.6253 | 23.721 | 26.855 | -177.5462 | 2.15011 | 1 |
| 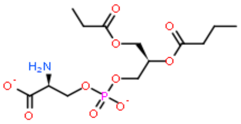 | 0 | 40 | 6323481 | -12.77 | 47 | 14 | -11.761 | -4.5943 | -28.64 | 7.7114 | 21.494 | 29.635 | -143.6609 | 1.75993 | 1 |
| 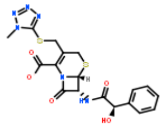 | 0 | 1160 | 1326 | -12.76 | 48 | 7 | -11.957 | -5.0936 | -25.27 | 8.949 | 26.833 | 24.973 | -127.6227 | 1.83076 | 1 |
| 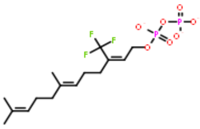 | 0 | 2513 | C15H25F3O7P2 | -12.75 | 49 | 11 | -14.981 | -5.1989 | -23.44 | 2.9358 | 19.521 | 44.051 | -105.4474 | 1.43186 | 1 |
| 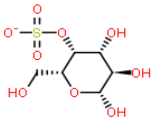 | 0 | 2540 | C6H12O9S | -12.74 | 27 | 7 | -10.484 | -2.1262 | -17.07 | 3.6855 | 22.228 | 13.922 | -79.17742 | 0.94583 | 1 |
| 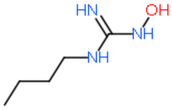 | 0 | 2441 | C5H13N3O | -12.73 | 22 | 6 | -6.5569 | -2.8042 | -11.48 | 0.5208 | 12.462 | 5.9954 | -38.38771 | 0.77664 | 1 |
| 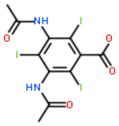 | 0 | 166 | 2140 | -12.72 | 28 | 1 | -4.8015 | -3.8276 | -16.12 | 9.7282 | 12.791 | 3.1438 | -40.57957 | 0.98447 | 1 |
| 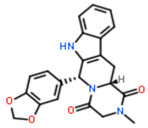 | 0 | 701 | 820 | -12.72 | 48 | 1 | -4.8784 | -6.153 | -23.57 | 1.962 | 17.828 | 18.439 | -149.7448 | 1.61619 | 1 |
| 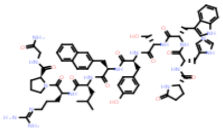 | 0 | 553 | C66H83N17O13 | -12.72 | 179 | 35 | -14.032 | -11.548 | -62.22 | 23.474 | 45.834 | 35.272 | -286.2004 | 4.5942 | 1 |

| | | | | | | | | | | | | | | | |
|---|---|---|---|---|---|---|---|---|---|---|---|---|---|---|---|
| 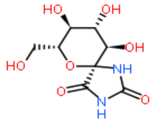 | 0 | 1600 | C8H12N2O7 | -12.68 | 29 | 5 | -9.1808 | -1.9075 | -18.23 | 0.261 | 21.385 | 16.446 | -86.65702 | 0.92245 | 1 |
| 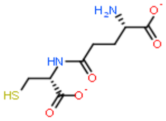 | 0 | 3076 | C8H14N2O5S | -12.67 | 28 | 9 | -12.682 | -2.194 | -15.62 | 5.5635 | 20.618 | 18.645 | -101.1445 | 1.22439 | 1 |
| 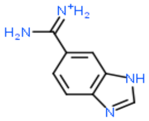 | 0 | 1704 | ABI | -12.65 | 21 | 0 | -5.1874 | -2.0166 | -18.06 | 0.2401 | 14.058 | 13.935 | -31.20076 | 0.68982 | 1 |
| 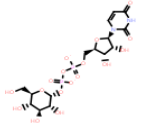 | 0 | 1636 | m | -12.65 | 58 | 15 | -7.8034 | -2.1044 | -24.03 | 8.9076 | 27.885 | -4.122 | -68.36242 | 1.50016 | 1 |
| 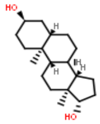 | 0 | 1326 | 5a-Androstane-3a,17b-diol.mol | -12.61 | 53 | 2 | -6.5884 | -4.6309 | -16.93 | 2.3271 | 14.839 | 15.702 | -98.86267 | 1.09808 | 1 |
| 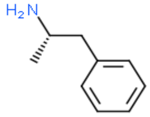 | 0 | 77 | C9H13N | -12.61 | 23 | 3 | -3.8842 | -3.6608 | -13.8 | 0.8508 | 9.7646 | 5.8136 | -47.00847 | 0.86971 | 1 |
| 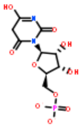 | 0 | 3315 | C9H13N2O10P | -12.6 | 33 | 7 | -12.239 | -2.4582 | -14.26 | 0 | 23.612 | 18.734 | -96.04933 | 1.35956 | 1 |
| 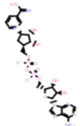 | 0 | 2228 | C22H30N7O13P2 | -12.6 | 72 | 15 | -15.945 | -4.7304 | -31.31 | 13.298 | 34.192 | 29.692 | -164.4436 | 2.34406 | 1 |
| 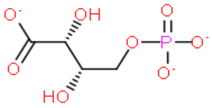 | 0 | 1539 | C4H9O8P | -12.6 | 19 | 7 | -11.095 | -1.4219 | -11.98 | 0 | 15.422 | 19.153 | -78.77724 | 0.77658 | 1 |
| 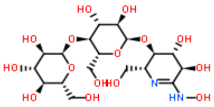 | 0 | 1688 | C18H32N2O15 | -12.59 | 67 | 19 | -12.127 | -3.8248 | -26.93 | 0 | 30.318 | 20.367 | -129.4907 | 1.80919 | 1 |
| 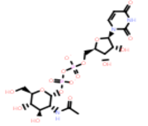 | 0 | 3067 | C17H27N3O17P2 | -12.59 | 64 | 15 | -14.725 | -4.2583 | -29.9 | 8.979 | 30.186 | 30.901 | -171.3456 | 2.26501 | 1 |

| | | | | | | | | | | | | | | | |
|---|---|---|---|---|---|---|---|---|---|---|---|---|---|---|---|
| 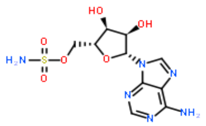 | 0 | 2615 | C10H14N6O6S | -12.58 | 37 | 7 | -12.73 | -3.8966 | -22.51 | 8.0505 | 29.766 | 20.287 | -113.1095 | 1.65174 | 1 |
| 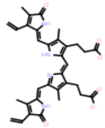 | 0 | 1831 | m | -12.58 | 75 | 6 | -11.281 | -6.2361 | -25.61 | 11.908 | 24.387 | 25.327 | -166.5682 | 2.04097 | 1 |
| 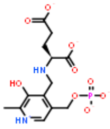 | 0 | 1590 | C13H20N2O9P | -12.58 | 41 | 10 | -13.179 | -3.786 | -19.83 | 0 | 26.878 | 25.721 | -131.2887 | 1.62556 | 1 |
| 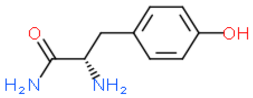 | 0 | 3051 | TYC | -12.57 | 25 | 4 | -7.8267 | -2.097 | -15.17 | 3.2319 | 15.673 | 11.678 | -51.08909 | 0.83953 | 1 |
| 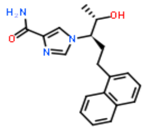 | 0 | 2901 | FR6 | -12.57 | 45 | 6 | -7.7528 | -4.8899 | -26.33 | 2.8974 | 22.283 | 22.12 | -123.238 | 1.44444 | 1 |
| 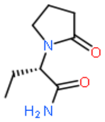 | 0 | 1072 | C8H14N2O2 | -12.53 | 26 | 3 | -7.5431 | -2.8844 | -13.06 | 0.5379 | 13.486 | 14.1 | -63.29669 | 0.81491 | 1 |
| 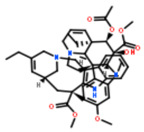 | 0 | 254 | C45H54N4O8 | -12.52 | 111 | 7 | -3.4786 | -8.5637 | -27.01 | 7.4768 | 18.009 | 10.366 | -160.384 | 2.53194 | 1 |
| 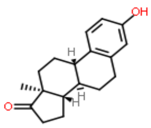 | 0 | 542 | 655 | -12.51 | 42 | 0 | -5.0809 | -4.0086 | -18.17 | 0.9556 | 14.155 | 15.162 | -96.92878 | 1.00786 | 1 |
| 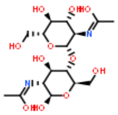 | 0 | 2708 | C16H28N2O11 | -12.51 | 57 | 14 | -13.101 | -4.5623 | -19.07 | 3.5456 | 27.927 | 17.11 | -153.4854 | 1.913 | 1 |
| 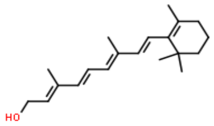 | 0 | 58 | Carotene-beta | -12.5 | 51 | 2 | -4.9482 | -6.8364 | -24.85 | 2.3163 | 17.4 | 20.919 | -120.6314 | 1.77302 | 1 |
| 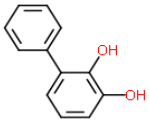 | 0 | 2622 | BPY | -12.49 | 24 | 1 | -5.4949 | -3.7588 | -16.77 | 5.3334 | 13.367 | 10.41 | -59.09816 | 1.00932 | 1 |

| | | | | | | | | | | | | | | | |
|---|---|---|---|---|---|---|---|---|---|---|---|---|---|---|---|
| 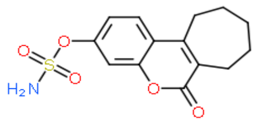 | 0 | 2034 | 667 | -12.47 | 36 | 2 | -7.3538 | -3.9735 | -20.03 | 3.8446 | 18.512 | 17.323 | -126.6524 | 1.61516 | 1 |
| 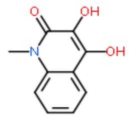 | 0 | 1537 | LI6 | -12.45 | 23 | 0 | -4.0466 | -3.0375 | -13.75 | 1.2465 | 7.9609 | 10.398 | -76.66818 | 0.94675 | 1 |
| 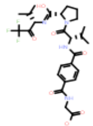 | 0 | 3345 | C26H33F3N4O7 | -12.44 | 72 | 11 | -7.7465 | -8.1515 | -37.61 | 15.034 | 26.379 | 22.464 | -174.9946 | 2.10377 | 1 |
| 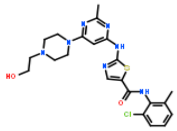 | 0 | 1123 | 1254 | -12.44 | 59 | 3 | -5.1989 | -8.4976 | -30.08 | 5.8196 | 25.764 | 17.989 | -141.031 | 2.3258 | 1 |
| 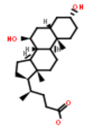 | 0 | 1380 | 1586 | -12.43 | 67 | 6 | -5.2568 | -5.1463 | -17 | 3.0415 | 17.804 | 3.8573 | -94.31152 | 1.60643 | 1 |
| 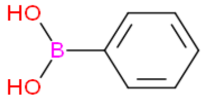 | 0 | 1573 | C6H7BO2 | -12.42 | 16 | 3 | -7.7201 | -2.4391 | -10.26 | 0 | 12.874 | 11.435 | -24.55927 | 0.57823 | 1 |
| 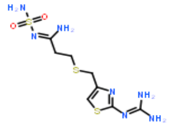 | 0 | 806 | C8H15N7O2S3 | -12.41 | 35 | 7 | -5.8509 | -3.5508 | -29.84 | 1.9293 | 22.793 | 18.423 | -86.33498 | 1.29178 | 1 |
| 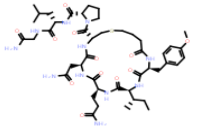 | 0 | 1140 | C45H69N11O12S | -12.4 | 138 | 18 | -9.6008 | -8.7318 | -44.15 | 6.9273 | 34.778 | 31.585 | -247.7379 | 3.51513 | 1 |
| 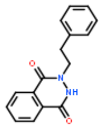 | 0 | 3089 | I06 | -12.4 | 34 | 3 | -5.1247 | -4.435 | -18.91 | 1.862 | 13.15 | 14.647 | -113.3635 | 1.25079 | 1 |
| 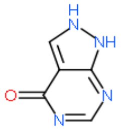 | 0 | 327 | 437 | -12.36 | 14 | 0 | -7.1547 | -1.1525 | -12.26 | 0.9062 | 13.407 | 12.383 | -40.88126 | 0.54638 | 1 |
| 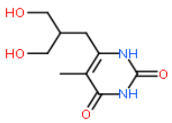 | 0 | 2230 | C9H14N2O4 | -12.35 | 29 | 6 | -6.7869 | -2.6763 | -15.33 | 1.0627 | 16.834 | 7.6939 | -102.626 | 1.22353 | 1 |

| | | | | | | | | | | | | | | | |
|---|---|---|---|---|---|---|---|---|---|---|---|---|---|---|---|
| 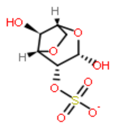 | 0 | 1745 | C6H10O8S | -12.35 | 24 | 4 | -7.7112 | -2.0813 | -11.61 | 0.1349 | 18.628 | 5.6282 | -76.28999 | 0.87102 | 1 |
| 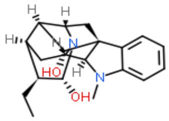 | 0 | 1233 | m | -12.3 | 50 | 3 | -4.0636 | -5.2061 | -20.46 | 0.6212 | 13.981 | 14.034 | -73.6351 | 1.13048 | 1 |
| 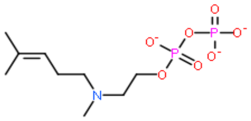 | 0 | 2108 | C9H18NO7P2 | -12.29 | 37 | 9 | -12.283 | -3.7689 | -19.75 | 2.4172 | 17.647 | 31.788 | -77.43578 | 1.23545 | 1 |
| 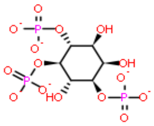 | 0 | 3070 | C6H15O15P3 | -12.29 | 33 | 9 | -11.663 | -1.9005 | -15.34 | 0 | 25.287 | 14.353 | -89.26679 | 1.31491 | 1 |
| 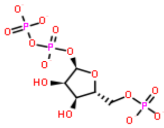 | 0 | 1424 | C5H13O14P3 | -12.28 | 30 | 9 | -16.446 | -2.0637 | -19.2 | 0 | 26.353 | 37.492 | -114.5741 | 1.27119 | 1 |
| 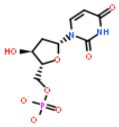 | 0 | 3437 | C9H13N2O8P | -12.28 | 31 | 5 | -11.351 | -2.2231 | -22.04 | 4.8887 | 24.625 | 23.575 | -88.99021 | 1.20727 | 1 |
| 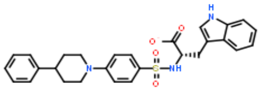 | 0 | 2182 | C28H29N3O4S | -12.27 | 64 | 7 | -9.0607 | -6.7939 | -32.12 | 15.543 | 21.269 | 27.045 | -166.0275 | 2.06712 | 1 |
| 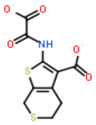 | 0 | 1830 | COL | -12.26 | 25 | 2 | -6.3197 | -2.9984 | -18.97 | 1.5949 | 15.173 | 16.918 | -95.24806 | 1.30291 | 1 |
| 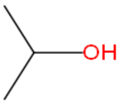 | 0 | 2067 | C3H8O | -12.26 | 12 | 1 | -3.4796 | -1.5857 | -7.853 | 0.0994 | 5.7283 | 0.7661 | -2.984992 | 0.208 | 1 |
| 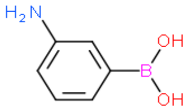 | 0 | 1667 | C6H8BNO2 | -12.26 | 18 | 2 | -5.8538 | -2.6094 | -14.93 | 0 | 15.088 | 10.179 | -35.57206 | 0.67504 | 1 |
| 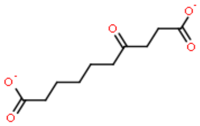 | 0 | 2004 | 4OX | -12.25 | 29 | 9 | -12.64 | -3.1271 | -13.48 | 4.0572 | 17.762 | 21.429 | -98.24157 | 1.18701 | 1 |

| | | | | | | | | | | | | | | | |
|---|---|---|---|---|---|---|---|---|---|---|---|---|---|---|---|
| 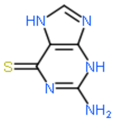 | 0 | 245 | 2723601 | -12.2 | 16 | 0 | -4.1782 | -1.3752 | -14.12 | 0.3413 | 12.624 | 5.6715 | -20.01295 | 0.44452 | 1 |
| 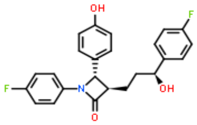 | 0 | 850 | 973 | -12.18 | 51 | 7 | -7.6606 | -5.6045 | -23.22 | 6.3632 | 19.717 | 17.231 | -161.19 | 1.54801 | 1 |
| 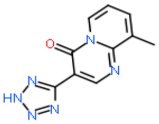 | 0 | 765 | 885 | -12.16 | 25 | 0 | -4.8239 | -3.3913 | -20.36 | 0.5218 | 16.53 | 15.099 | -70.89658 | 1.211 | 1 |
| 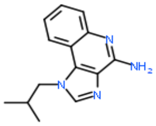 | 0 | 608 | 724 | -12.15 | 34 | 2 | -6.4156 | -4.9849 | -16.84 | 1.9469 | 17.484 | 13.301 | -93.27425 | 1.20192 | 1 |
| 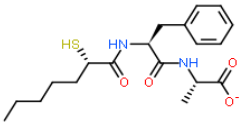 | 0 | 2322 | TI1 | -12.15 | 53 | 12 | -11.934 | -5.2878 | -26.05 | 16.339 | 23.189 | 20.068 | -143.4251 | 1.52042 | 1 |
| 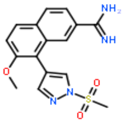 | 0 | 2737 | UI3 | -12.14 | 40 | 1 | -6.4652 | -4.6124 | -24.8 | 3.7351 | 19.907 | 21.716 | -137.6962 | 1.53199 | 1 |
| 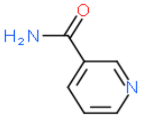 | 0 | 2417 | C6H6N2O | -12.13 | 15 | 0 | -4.2584 | -1.8324 | -13.63 | 0.2714 | 9.022 | 9.9569 | -45.67637 | 0.53631 | 1 |
| 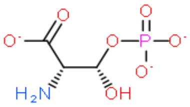 | 0 | 3456 | C3H8NO7P | -12.12 | 17 | 6 | -9.4314 | -1.0708 | -13.01 | 0 | 13.833 | 17.417 | -56.0388 | 0.70184 | 1 |
| 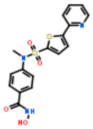 | 0 | 2616 | NHB | -12.1 | 41 | 5 | -3.6752 | -5.2143 | -26.95 | 0.3534 | 15.484 | 19.414 | -85.8032 | 1.25794 | 1 |
| 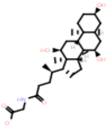 | 0 | 2408 | C26H42NO6 | -12.1 | 75 | 9 | -7.2431 | -5.0955 | -23.97 | 4.3042 | 24.588 | 10.884 | -91.73432 | 2.02464 | 1 |
| 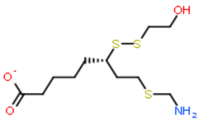 | 0 | 2869 | C11H23NO3S3 | -12.09 | 40 | 15 | -8.6904 | -3.2009 | -19.15 | 7.3692 | 20.858 | 3.2164 | -15.93658 | 1.31632 | 1 |

| | | | | | | | | | | | | | | | |
|---|---|---|---|---|---|---|---|---|---|---|---|---|---|---|---|
| 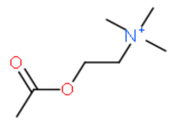 | 0 | 2812 | C7H16NO2 | -12.09 | 26 | 3 | -5.9789 | -3.892 | -13.19 | 1.6745 | 10.679 | 12.16 | -35.80612 | 0.89119 | 1 |
| 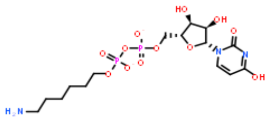 | 0 | 2412 | C15H27N3O12P2 | -12.07 | 57 | 17 | -16.636 | -3.9792 | -22.37 | 3.6726 | 29.303 | 31.325 | -138.5346 | 1.89382 | 1 |
| 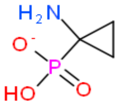 | 0 | 2743 | C3H7NO3P | -12.07 | 15 | 3 | -7.1077 | -1.7707 | -8.742 | 0.3897 | 15.49 | 3.3225 | -33.5832 | 0.52228 | 1 |
| 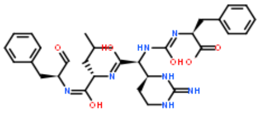 | 0 | 1471 | C31H41N7O6 | -12.03 | 84 | 19 | -13.275 | -6.0284 | -39.08 | 6.647 | 34.043 | 35.996 | -162.3612 | 2.39112 | 1 |
| 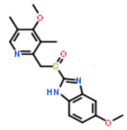 | 0 | 619 | C17H19N3O3S | -12.03 | 43 | 3 | -8.5743 | -5.9107 | -21.13 | 5.7356 | 21.342 | 20.47 | -122.8133 | 1.78523 | 1 |
| 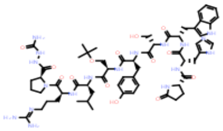 | 0 | 2 | [NO | -12.02 | 175 | 36 | -14.276 | -12.358 | -65.75 | 21.857 | 48.079 | 41.446 | -270.0884 | 4.39012 | 1 |
| 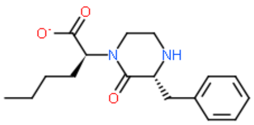 | 0 | 2777 | C17H24N2O3 | -12.01 | 45 | 7 | -9.3789 | -5.43 | -22.76 | 6.9538 | 19.013 | 23.462 | -143.4969 | 1.66071 | 1 |
| 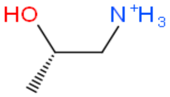 | 0 | 2302 | C3H10NO | -12.01 | 15 | 2 | -6.5375 | -1.4091 | -7.098 | 0 | 11.262 | 4.4368 | -17.63329 | 0.46714 | 1 |
| 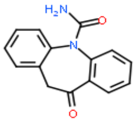 | 0 | 659 | C15H12N2O2 | -12 | 31 | 1 | -4.2444 | -4.0471 | -19.49 | 4.0119 | 14.424 | 10.791 | -89.66502 | 1.25697 | 1 |
| 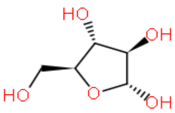 | 0 | 2825 | C5H10O5 | -11.99 | 20 | 5 | -8.3853 | -1.544 | -9.728 | 0.4109 | 14.255 | 9.1673 | -52.86006 | 0.79627 | 1 |
| 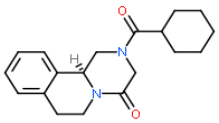 | 0 | 930 | 1058 | -11.92 | 47 | 2 | -5.2831 | -5.9368 | -20.96 | 2.8999 | 15.379 | 18.049 | -146.8168 | 1.60472 | 1 |

| | | | | | | | | | | | | | | | |
|---|---|---|---|---|---|---|---|---|---|---|---|---|---|---|---|
| 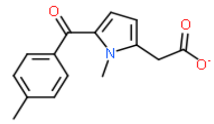 | 0 | 390 | 500 | -11.92 | 33 | 2 | -2.7262 | -4.3748 | -19.06 | 2.0554 | 13.786 | 6.3574 | -74.44578 | 1.31702 | 1 |
| 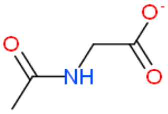 | 0 | 2428 | C4H7NO3 | -11.91 | 14 | 2 | -6.9447 | -1.8852 | -9.838 | 0 | 13.203 | 8.5784 | -52.22356 | 0.65243 | 1 |
| 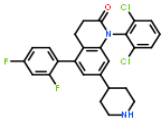 | 0 | 1712 | C26H22Cl2F2N2O | -11.9 | 55 | 1 | -1.9115 | -7.2576 | -29.78 | 2.5259 | 15.89 | 20.522 | -117.9715 | 1.7035 | 1 |
| 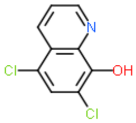 | 0 | 1112 | 1243 | -11.88 | 18 | 0 | -2.7512 | -3.8418 | -14.21 | 0 | 8.9938 | 7.7482 | -59.9025 | 0.90404 | 1 |
| 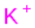 | 0 | 1174 | K | -11.85 | 1 | 0 | -4.7357 | 0.12086 | -1.382 | 0 | 1.9831 | -0.088 | 22.185972 | -0.1226 | 1 |
| 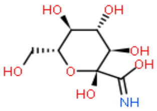 | 0 | 2433 | C7H13NO7 | -11.84 | 28 | 8 | -10.365 | -2.039 | -10.13 | 4.2493 | 19.514 | 5.8902 | -84.78429 | 0.82696 | 1 |
| 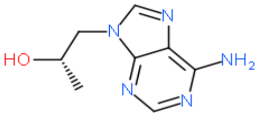 | 0 | 2695 | C8H11N5O | -11.84 | 25 | 3 | -8.5719 | -4.2269 | -12.83 | 4.0696 | 18.459 | 11.248 | -52.53167 | 0.84863 | 1 |
| 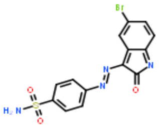 | 0 | 2669 | 106 | -11.84 | 32 | 2 | -6.8368 | -4.4947 | -23.45 | 3.2037 | 20.151 | 20.73 | -118.1779 | 1.66576 | 1 |
| 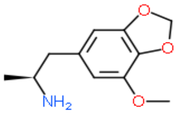 | 0 | 1248 | 1442 | -11.83 | 30 | 3 | -4.9839 | -4.3089 | -15.95 | 1.6758 | 16.379 | 6.8542 | -84.08044 | 1.2948 | 1 |
| 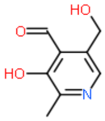 | 0 | 43 | 147 | -11.82 | 21 | 2 | -5.7563 | -2.7163 | -15.89 | 1.6864 | 12.946 | 12.929 | -46.67593 | 0.91115 | 1 |
| 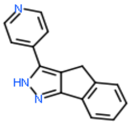 | 0 | 3154 | LIG | -11.82 | 29 | 0 | -4.0304 | -4.4678 | -18.74 | 2.7724 | 14.391 | 11.589 | -82.09375 | 1.24802 | 1 |

| | | | | | | | | | | | | | | | |
|---|---|---|---|---|---|---|---|---|---|---|---|---|---|---|---|
| 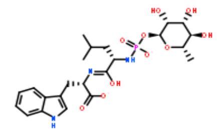 | 0 | 2285 | C23H34N3O10P | -11.79 | 69 | 15 | -12.132 | -5.769 | -19.26 | 6.4864 | 24.867 | 15.885 | -153.5662 | 2.25451 | 1 |
| 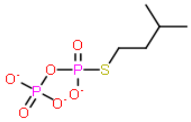 | 0 | 2014 | DST | -11.79 | 25 | 6 | -12.812 | -2.6164 | -16.67 | 0 | 18.064 | 33.178 | -70.39632 | 0.98294 | 1 |
| 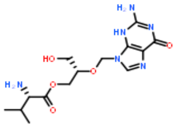 | 0 | 1403 | 1610 | -11.78 | 47 | 10 | -14.278 | -4.2546 | -25.54 | 9.447 | 32.391 | 25.411 | -127.4059 | 1.83239 | 1 |
| 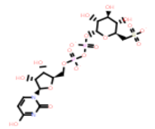 | 0 | 2282 | C15H24N2O19P2S | -11.78 | 60 | 16 | -17.602 | -3.7762 | -24.81 | 8.7038 | 35.262 | 28.517 | -150.2434 | 2.30581 | 1 |
| 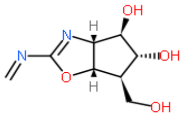 | 0 | 3197 | C8H12N2O4 | -11.78 | 26 | 4 | -5.8561 | -1.6121 | -15.81 | 2.8423 | 16.087 | 5.6887 | -16.66899 | 0.74634 | 1 |
| 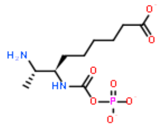 | 0 | 2625 | C10H21N2O7P | -11.76 | 38 | 11 | -12.29 | -3.3352 | -15.22 | 11.82 | 20.969 | 11.161 | -107.9905 | 1.18386 | 1 |
| 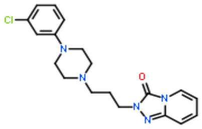 | 0 | 543 | 656 | -11.75 | 48 | 4 | -4.839 | -6.7882 | -27.61 | 6.7162 | 15.577 | 22.026 | -119.7426 | 1.58511 | 1 |
| 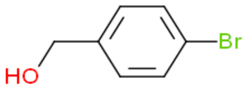 | 0 | 2526 | C7H7BrO | -11.75 | 16 | 2 | -4.8151 | -3.5357 | -13.03 | 2.0053 | 10.749 | 8.1347 | -39.447 | 0.81413 | 1 |
| 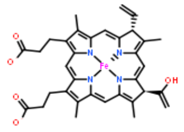 | 0 | 2645 | C34H34FeN4O5 | -11.75 | 76 | 8 | -8.0341 | -7.697 | -24.87 | 9.7982 | 21.84 | 17.498 | -130.6673 | 2.19435 | 1 |
| 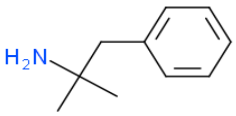 | 0 | 86 | 191 | -11.74 | 26 | 3 | -4.3209 | -3.8183 | -15.14 | 1.865 | 12.11 | 7.4209 | -48.70507 | 0.95907 | 1 |
| 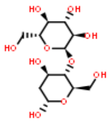 | 0 | 2959 | C12H22O10 | -11.73 | 44 | 11 | -11.661 | -3.4042 | -16.55 | 6.3305 | 25.069 | 11.22 | -130.7859 | 1.51442 | 1 |

| | | | | | | | | | | | | | | | |
|---|---|---|---|---|---|---|---|---|---|---|---|---|---|---|---|
| 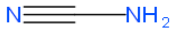 | 0 | 2398 | CH2N2 | -11.7 | 5 | 1 | -4.9086 | -0.7634 | -4.668 | 0.1029 | 5.3885 | 1.8122 | 3.880722 | 0.0261 | 1 |
| 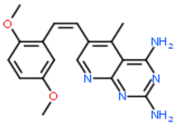 | 0 | 2749 | LII | -11.68 | 44 | 0 | -2.2244 | -4.7834 | -26.03 | 1.1844 | 18.172 | 13.397 | -15.42263 | 1.21404 | 1 |
| 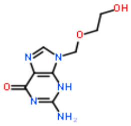 | 0 | 668 | 787 | -11.67 | 27 | 5 | -11.029 | -2.9631 | -16.7 | 4.0391 | 23.86 | 17.581 | -80.08379 | 1.16465 | 1 |
| 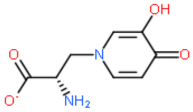 | 0 | 927 | 1055 | -11.66 | 23 | 4 | -7.4569 | -2.0095 | -16.2 | 0 | 14.566 | 17.315 | -76.23799 | 1.0975 | 1 |
| 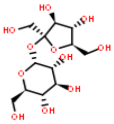 | 0 | 2480 | m | -11.66 | 45 | 13 | -12.789 | -3.1935 | -12.65 | 3.3348 | 20.215 | 15.941 | -125.6086 | 1.4877 | 1 |
| 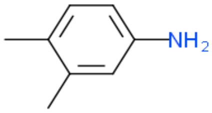 | 0 | 2712 | C8H11N | -11.63 | 20 | 0 | -2.3528 | -3.0163 | -14.87 | 0.6621 | 6.6346 | 8.8425 | -22.53653 | 0.5239 | 1 |
| 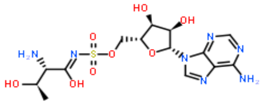 | 0 | 3030 | C14H21N7O8S | -11.63 | 51 | 12 | -14.889 | -4.6268 | -23.36 | 6.0153 | 34.736 | 23.692 | -135.3432 | 1.8691 | 1 |
| 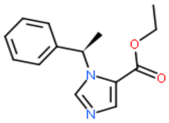 | 0 | 186 | C14H16N2O2 | -11.63 | 34 | 3 | -5.3496 | -5.4187 | -20.27 | 8.5654 | 13.66 | 13.063 | -100.0666 | 1.17077 | 1 |
| 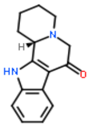 | 0 | 1944 | IQZ | -11.62 | 34 | 0 | -4.6391 | -4.605 | -14.76 | 1.0984 | 13.005 | 11.43 | -95.62471 | 1.34174 | 1 |
| 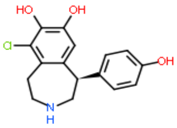 | 0 | 681 | 800 | -11.62 | 37 | 1 | -4.8872 | -4.0839 | -21.46 | 4.3093 | 16.186 | 14.575 | -75.79668 | 0.9652 | 1 |
| 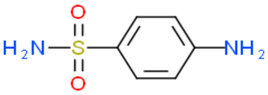 | 0 | 154 | 259 | -11.61 | 19 | 2 | -2.9002 | -2.2662 | -16.26 | 0.0817 | 11.551 | 5.4409 | -59.58364 | 0.68283 | 1 |

| | | | | | | | | | | | | | | | |
|---|---|---|---|---|---|---|---|---|---|---|---|---|---|---|---|
| 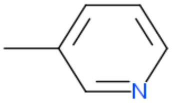 | 0 | 1760 | C6H7N | -11.59 | 14 | 0 | -3.6269 | -3.2551 | -11.5 | 0.3905 | 7.9716 | 7.6532 | -27.67479 | 0.56243 | 1 |
| 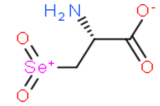 | 0 | 2176 | C3H6NO4Se | -11.58 | 14 | 4 | -4.7663 | -1.1561 | -13.56 | 1.4556 | 10.947 | 5.0546 | -59.0165 | 0.3976 | 1 |
| 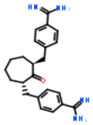 | 0 | 1839 | C23H28N4O | -11.57 | 56 | 4 | -8.5108 | -5.008 | -28.69 | 9.4594 | 23.457 | 24.757 | -153.9145 | 1.52376 | 1 |
| 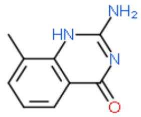 | 0 | 1602 | MAQ | -11.56 | 22 | 0 | -4.1137 | -2.2032 | -14.92 | 1.2081 | 12.799 | 7.4523 | -24.96325 | 0.71672 | 1 |
| 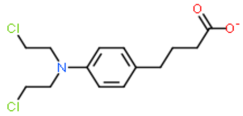 | 0 | 185 | 291 | -11.55 | 37 | 8 | -5.3825 | -4.8896 | -17.33 | 2.0688 | 14.393 | 8.8366 | -105.8071 | 1.27561 | 1 |
| 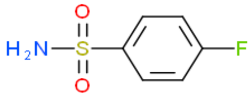 | 0 | 1563 | C6H6FNO2S | -11.55 | 17 | 2 | -2.598 | -2.3926 | -15.61 | 0.0308 | 9.8943 | 5.4584 | -67.14765 | 0.7578 | 1 |
| 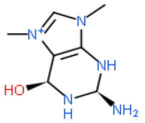 | 0 | 1742 | C7H14N5O | -11.54 | 27 | 2 | -7.8445 | -2.3513 | -12.67 | 0 | 17.554 | 12.401 | -28.21376 | 0.63832 | 1 |
| 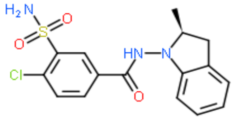 | 0 | 689 | 808 | -11.53 | 40 | 3 | -3.2556 | -5.2828 | -27.46 | 2.6963 | 16.354 | 18.219 | -101.3001 | 1.47116 | 1 |
| 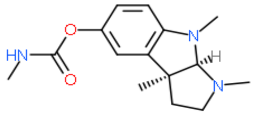 | 0 | 858 | 981 | -11.53 | 41 | 1 | -8.7953 | -5.1254 | -21.61 | 12.62 | 18.54 | 20.739 | -105.9075 | 1.60085 | 1 |
| 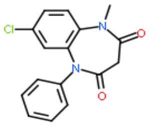 | 0 | 242 | 349 | -11.52 | 34 | 1 | -4.913 | -4.8192 | -17.88 | 3.5177 | 13.555 | 13.847 | -108.1158 | 1.41946 | 1 |
| 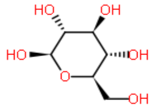 | 0 | 2118 | b-D-Glucose.mol | -11.52 | 24 | 6 | -11.118 | -2.0953 | -6.942 | 2.4692 | 15.629 | 12.349 | -40.73768 | 0.6719 | 1 |

| | | | | | | | | | | | | | | | |
|---|---|---|---|---|---|---|---|---|---|---|---|---|---|---|---|
| 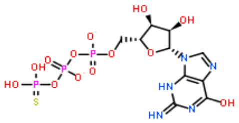 | 0 | 1639 | C10H16N5O13P3S | -11.51 | 46 | 13 | -13.972 | -3.0033 | -32.2 | 0 | 37.251 | 33.81 | -121.5698 | 1.85181 | 1 |
| 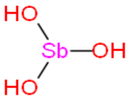 | 0 | 2186 | H3O3Sb | -11.51 | 7 | 3 | -7.2649 | -0.0122 | -9.367 | 0 | 11.061 | 9.3182 | -0.124527 | 0.00836 | 1 |
| 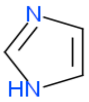 | 0 | 3040 | C3H4N2 | -11.5 | 9 | 0 | -3.5689 | -1.7321 | -7.815 | 0.1008 | 5.2171 | 3.83 | -5.135292 | 0.10999 | 1 |
| 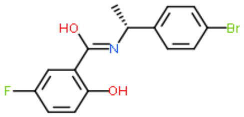 | 0 | 2581 | C15H13BrFNO2 | -11.5 | 33 | 3 | -3.2104 | -4.7235 | -19.74 | 6.9969 | 10.484 | 8.5839 | -55.96241 | 0.87051 | 1 |
| 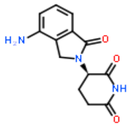 | 0 | 370 | 480 | -11.48 | 32 | 1 | -8.6895 | -3.7965 | -16.69 | 0.9635 | 17.756 | 23.098 | -120.9599 | 1.2174 | 1 |
| 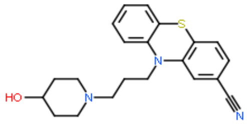 | 0 | 1401 | 1608 | -11.47 | 49 | 5 | -6.0648 | -5.0629 | -20.35 | 4.2405 | 14.776 | 16.653 | -59.28243 | 1.34243 | 1 |
| 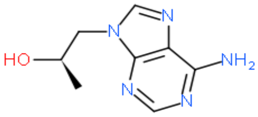 | 0 | 2475 | C8H11N5O | -11.47 | 25 | 3 | -5.9027 | -4.2252 | -16.19 | 1.4801 | 18.213 | 9.2961 | -68.79234 | 0.91858 | 1 |
| 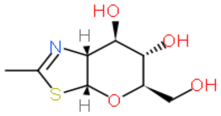 | 0 | 3387 | C8H13NO4S | -11.46 | 27 | 4 | -7.9723 | -3.0029 | -11.61 | 1.3351 | 16.775 | 9.9183 | -58.38502 | 1.00141 | 1 |
| 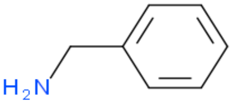 | 0 | 2197 | C7H9N | -11.46 | 17 | 2 | -5.7171 | -2.9811 | -12.62 | 1.8714 | 11.669 | 9.9233 | -30.03605 | 0.66235 | 1 |
| 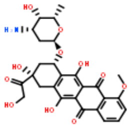 | 0 | 873 | 997 | -11.45 | 68 | 8 | -11.924 | -5.9572 | -16.79 | 3.9306 | 25.301 | 20.244 | -114.924 | 1.62636 | 1 |
| 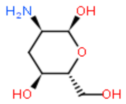 | 0 | 2416 | C6H13NO4 | -11.45 | 24 | 5 | -8.0587 | -2.1807 | -9.991 | 0 | 18.056 | 5.8339 | -65.16792 | 0.8266 | 1 |

| | | | | | | | | | | | | | | | |
|---|---|---|---|---|---|---|---|---|---|---|---|---|---|---|---|
| 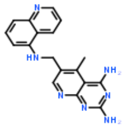 | 0 | 3026 | LIH | -11.42 | 42 | 2 | -5.6926 | -6.164 | -22.25 | 4.89 | 20.824 | 14.562 | -78.44766 | 1.41633 | 1 |
| 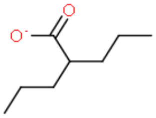 | 0 | 207 | C8H16O2 | -11.41 | 25 | 5 | -7.9284 | -3.6114 | -11.21 | 1.8173 | 12.289 | 13.601 | -64.53349 | 1.02373 | 1 |
| 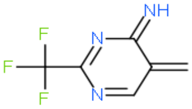 | 0 | 2586 | C6H4F3N3 | -11.4 | 16 | 1 | -4.9329 | -2.6503 | -16.28 | 0.4565 | 13.644 | 12.173 | -54.49394 | 0.74951 | 1 |
| 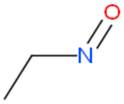 | 0 | 2367 | C2H5NO | -11.4 | 9 | 1 | -5.5702 | -1.7783 | -7.921 | 0.126 | 8.6663 | 7.0171 | -32.4237 | 0.28842 | 1 |
| 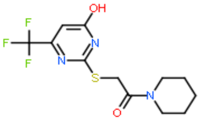 | 0 | 2704 | B1V | -11.38 | 35 | 4 | -8.9569 | -4.5871 | -19.41 | 2.8441 | 18.539 | 23.863 | -83.80077 | 1.40804 | 1 |
| 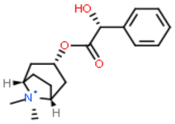 | 0 | 609 | C17H24BrNO3 | -11.38 | 45 | 4 | -5.2781 | -5.0781 | -18.41 | 1.7342 | 16.631 | 11.921 | -118.3458 | 1.6066 | 1 |
| 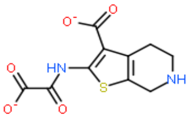 | 0 | 3317 | C10H10N2O5S | -11.38 | 26 | 2 | -6.3562 | -2.838 | -18.52 | 1.2374 | 15.679 | 17.304 | -92.02508 | 1.28169 | 1 |
| 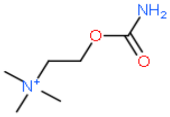 | 0 | 301 | 51-83-2 | -11.37 | 25 | 3 | -6.4921 | -2.5094 | -13.56 | 1.7174 | 11.726 | 13.127 | -11.09458 | 0.68534 | 1 |
| 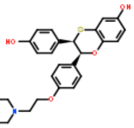 | 0 | 3383 | C27H29NO4S | -11.37 | 62 | 5 | -7.978 | -6.3508 | -29.31 | 6.6011 | 27.793 | 22.072 | -117.9376 | 2.22072 | 1 |
| 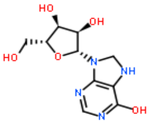 | 0 | 2205 | C10H14N4O5 | -11.37 | 33 | 5 | -11.458 | -3.6334 | -16.68 | 3.1336 | 25.7 | 19.065 | -94.50671 | 1.2847 | 1 |
| 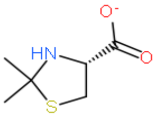 | 0 | 1427 | C6H11NO2S | -11.37 | 20 | 1 | -3.2506 | -2.6875 | -10.22 | 2.0061 | 10.376 | -1.074 | -42.3255 | 0.63297 | 1 |

| | | | | | | | | | | | | | | | | |
|---|---|---|---|---|---|---|---|---|---|---|---|---|---|---|---|---|
| 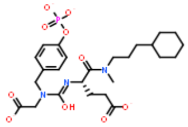 | 0 | 1553 | C25H38N3O10P | -11.36 | 73 | 17 | -10.474 | -6.7536 | -29.69 | 7.1043 | 27.127 | 22.049 | -138.908 | 2.05199 | | 1 |
| 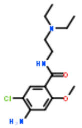 | 0 | 1102 | 1233 | -11.35 | 42 | 5 | -4.9695 | -6.2823 | -22.51 | 6.1976 | 14.258 | 15.948 | -66.45287 | 1.21234 | | 1 |
| 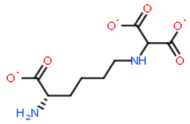 | 0 | 1592 | C9H16N2O6 | -11.35 | 30 | 10 | -16.216 | -2.307 | -16.63 | 0.6522 | 23.742 | 35.843 | -101.4027 | 1.31693 | | 1 |
| 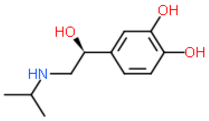 | 0 | 936 | 1064 | -11.34 | 32 | 5 | -6.8047 | -4.07 | -17.5 | 2.2847 | 17.083 | 13.518 | -98.72292 | 1.42286 | | 1 |
| 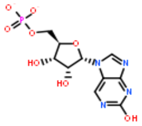 | 0 | 2605 | C10H13N4O8P | -11.31 | 34 | 6 | -13.856 | -3.216 | -20.25 | 8.5278 | 28.133 | 24.75 | -101.9905 | 1.29847 | | 1 |
| 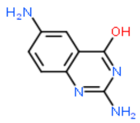 | 0 | 3168 | DQU | -11.3 | 21 | 0 | -5.237 | -3.1567 | -18.59 | 0.9693 | 17.977 | 13.006 | -53.46115 | 0.75096 | | 1 |
| 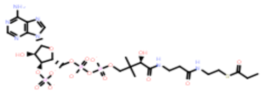 | 0 | 2612 | C24H40N7O17P3S | -11.3 | 88 | 22 | -20.234 | -5.6101 | -37.05 | 11.348 | 41.33 | 44.985 | -190.7185 | 3.14433 | | 1 |
| 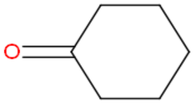 | 0 | 1819 | C6H10O | -11.3 | 17 | 0 | -4.7606 | -2.8588 | -8.696 | 0.0529 | 7.7432 | 8.3596 | -46.92083 | 0.53566 | | 1 |
| 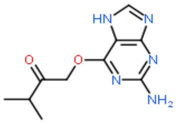 | 0 | 3311 | MBP | -11.3 | 30 | 3 | -7.2167 | -4.5357 | -20.1 | 3.4534 | 19.61 | 17.46 | -95.62151 | 1.06918 | | 1 |
| 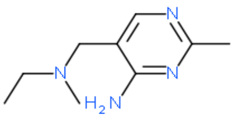 | 0 | 3302 | C9H16N4 | -11.3 | 29 | 3 | -3.8383 | -4.0795 | -15.48 | 2.2987 | 10.714 | 8.1996 | -13.99715 | 0.7595 | | 1 |
| 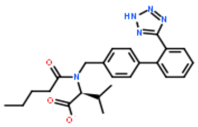 | 0 | 72 | 177 | -11.29 | 60 | 9 | -6.2865 | -6.9271 | -27.08 | 1.8684 | 21.959 | 20.229 | -144.1283 | 1.79097 | | 1 |

| | | | | | | | | | | | | | | | |
|---|---|---|---|---|---|---|---|---|---|---|---|---|---|---|---|
| 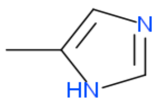 | 0 | 3056 | C4H6N2 | -11.28 | 12 | 0 | -3.3244 | -2.2457 | -9.058 | 0.1938 | 5.7537 | 4.936 | -7.15498 | 0.23402 | 1 |
| 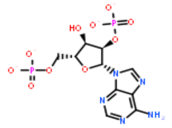 | 0 | 1856 | C10H15N5O10P2 | -11.26 | 38 | 7 | -14.168 | -3.3014 | -21.32 | 0 | 28.478 | 34.175 | -95.62348 | 1.39664 | 1 |
| 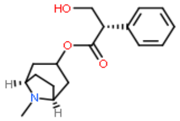 | 0 | 314 | C17H23NO3 | -11.23 | 44 | 5 | -5.4724 | -5.6471 | -21.66 | 0.6787 | 17.906 | 16.992 | -128.853 | 1.47153 | 1 |
| 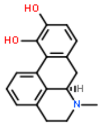 | 0 | 598 | 714 | -11.23 | 37 | 0 | -4.5029 | -4.0312 | -15.37 | 1.9984 | 11.1 | 13.187 | -85.51861 | 1.00552 | 1 |
| 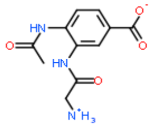 | 0 | 2532 | C11H14N3O4 | -11.23 | 31 | 2 | -6.5248 | -2.9259 | -20.12 | 3.3204 | 16.444 | 17.934 | -44.5888 | 0.83429 | 1 |
| 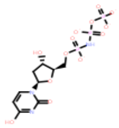 | 0 | 1729 | C9H16N3O13P3 | -11.22 | 40 | 10 | -6.8988 | -2.7109 | -9.411 | 0 | 22.252 | -7.917 | -66.20612 | 1.68564 | 1 |
| 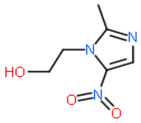 | 0 | 795 | 916 | -11.22 | 21 | 4 | -4.7415 | -2.8329 | -13.74 | 2.1312 | 9.6059 | 8.3866 | -48.00957 | 0.628 | 1 |
| 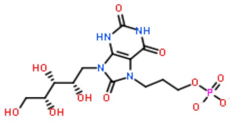 | 0 | 3447 | C13H21N4O11P | -11.21 | 48 | 14 | -10.865 | -3.2716 | -26.59 | 19.682 | 28.854 | 4.8749 | -92.24363 | 1.80639 | 1 |
| 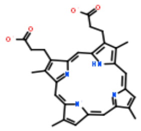 | 0 | 1926 | C30H28CoN4O4 | -11.2 | 65 | 6 | -7.352 | -4.8668 | -21.47 | 0 | 20.553 | 19.878 | -150.8021 | 1.86279 | 1 |
| 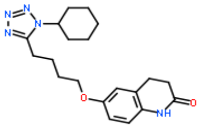 | 0 | 1036 | 1166 | -11.17 | 54 | 6 | -6.1704 | -5.7291 | -28.45 | 6.3839 | 22.369 | 18.971 | -135.9245 | 1.87866 | 1 |
| 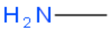 | 0 | 1605 | CH5N | -11.14 | 7 | 1 | -5.0239 | -1.167 | -7.51 | 0.0459 | 6.1154 | 7.0453 | -1.734927 | 0.00836 | 1 |

| | | | | | | | | | | | | | | | |
|---|---|---|---|---|---|---|---|---|---|---|---|---|---|---|---|
| 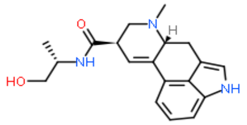 | 0 | 1122 | 1253 | -11.1 | 47 | 4 | -4.0621 | -5.6661 | -24.37 | 3.2254 | 15.867 | 16.823 | -95.54327 | 1.24495 | 1 |
| 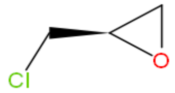 | 0 | 545 | 658 | -11.1 | 10 | 1 | -5.6254 | -2.6354 | -6.198 | 0.1354 | 7.6163 | 7.129 | -18.45441 | 0.41771 | 1 |
| 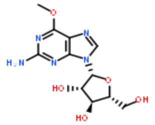 | 0 | 1139 | 1280 | -11.09 | 36 | 5 | -8.2072 | -3.4253 | -18.4 | 1.9624 | 19.796 | 17.184 | -50.79247 | 1.19879 | 1 |
| 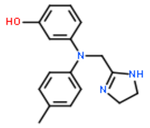 | 0 | 578 | 692 | -11.09 | 40 | 2 | -4.2753 | -5.271 | -24.61 | 1.2335 | 15.461 | 21.712 | -75.2213 | 1.06171 | 1 |
| 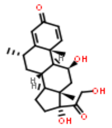 | 0 | 837 | C22H30O5 | -11.06 | 57 | 5 | -6.5968 | -4.2767 | -22 | 3.0754 | 20.262 | 15.833 | -99.17411 | 1.2397 | 1 |
| 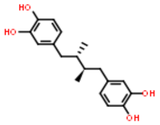 | 0 | 74 | 179 | -11.06 | 44 | 5 | -6.7452 | -4.5311 | -24.28 | 2.7915 | 18.076 | 22.873 | -103.1055 | 1.4186 | 1 |
| 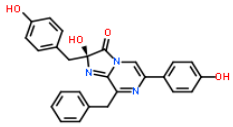 | 0 | 1946 | C26H21N3O4 | -11.04 | 54 | 5 | -9.1922 | -6.0953 | -21.65 | 8.0369 | 24.347 | 17.986 | -91.5557 | 1.78552 | 1 |
| 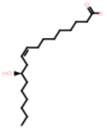 | 0 | 2651 | C18H34O3 | -11.03 | 54 | 16 | -10.523 | -5.7241 | -23.51 | 5.8506 | 19.036 | 23.568 | -112.7946 | 1.57396 | 1 |
| 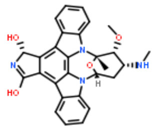 | 0 | 1698 | C28H26N4O4 | -11.03 | 62 | 4 | -2.6356 | -5.2783 | -25.8 | 2.9984 | 17.38 | 11.739 | -100.5936 | 1.51941 | 1 |
| 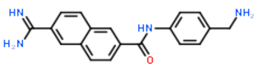 | 0 | 2134 | 239 | -11.02 | 42 | 2 | -6.2883 | -4.142 | -24.84 | 7.459 | 19.619 | 18.355 | -64.17905 | 1.33828 | 1 |
| 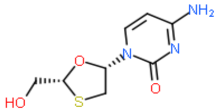 | 0 | 593 | 709 | -11.02 | 26 | 3 | -5.7049 | -3.2505 | -17.38 | 1.1732 | 16.468 | 12.305 | -64.70698 | 1.01865 | 1 |

| | | | | | | | | | | | | | | | |
|---|---|---|---|---|---|---|---|---|---|---|---|---|---|---|---|
| 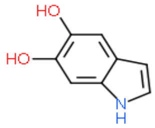 | 0 | 1588 | C8H7NO2 | -11.02 | 18 | 0 | -6.1881 | -2.4058 | -12.29 | 4.8578 | 13.731 | 8.0773 | -72.28975 | 0.77885 | 1 |
| 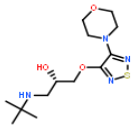 | 0 | 266 | C13H24N4O3S | -11.01 | 45 | 6 | -8.8668 | -5.3509 | -17.4 | 5.946 | 18.165 | 17.59 | -104.0605 | 1.62583 | 1 |
| 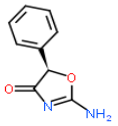 | 0 | 1099 | 1230 | -11.01 | 21 | 1 | -6.4743 | -2.4629 | -12.73 | 1.8674 | 14.231 | 11.141 | -65.62634 | 0.64871 | 1 |
| 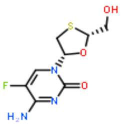 | 0 | 759 | 879 | -11.01 | 26 | 3 | -6.0605 | -3.1996 | -16.89 | 3.7551 | 16.876 | 10.066 | -77.51566 | 1.25428 | 1 |
| 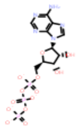 | 0 | 66 | C10H16N5O13P3 | -11.01 | 43 | 10 | -19.799 | -3.137 | -24.39 | 1.334 | 36.324 | 47.78 | -108.5662 | 1.39508 | 1 |
| 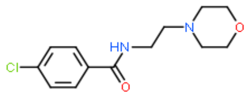 | 0 | 1041 | 1171 | -10.97 | 35 | 4 | -6.7382 | -5.3733 | -21.1 | 4.4535 | 15.822 | 20.968 | -81.53713 | 1.4576 | 1 |
| 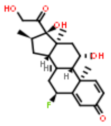 | 0 | 1200 | C22H29FO5 | -10.96 | 57 | 5 | -6.4714 | -4.767 | -20.99 | 3.0065 | 19.871 | 14.98 | -136.2854 | 1.56138 | 1 |
| 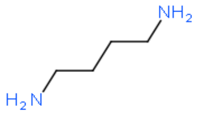 | 0 | 1683 | C4H12N2 | -10.96 | 18 | 5 | -7.6892 | -2.0955 | -9.692 | 2.4833 | 15.397 | 5.3853 | -22.63728 | 0.60126 | 1 |
| 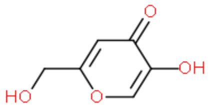 | 0 | 1541 | C6H6O4 | -10.95 | 16 | 2 | -6.7047 | -1.92 | -15.63 | 0.9673 | 16.461 | 13.346 | -65.29704 | 0.64241 | 1 |
| 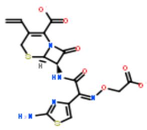 | 0 | 557 | C16H15N5O7S2 | -10.94 | 43 | 5 | -12.122 | -5.1709 | -29.02 | 4.9194 | 29.875 | 36.141 | -124.442 | 1.73984 | 1 |
| 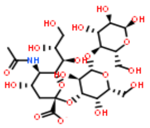 | 0 | 2973 | C23H39NO19 | -10.89 | 81 | 22 | -14.205 | -5.1435 | -25.02 | 0 | 34.478 | 21.897 | -172.5754 | 2.52272 | 1 |

| | | | | | | | | | | | | | | | |
|---|---|---|---|---|---|---|---|---|---|---|---|---|---|---|---|
| 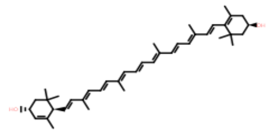 | 0 | 33 | Lutein | -10.89 | 98 | 3 | -2.7426 | -11.508 | -36.49 | 0 | 23.886 | 31.115 | -180.3372 | 3.13252 | 1 |
| 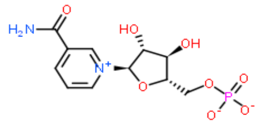 | 0 | 2908 | C11H16N2O8P | -10.87 | 36 | 6 | -10.294 | -3.1372 | -19.26 | 0.9003 | 24.514 | 21.23 | -121.9959 | 1.45292 | 1 |
| 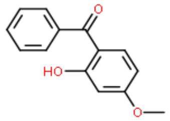 | 0 | 1235 | 1428 | -10.87 | 29 | 1 | -4.539 | -4.3417 | -20.21 | 0.3798 | 16.155 | 16.355 | -105.6253 | 1.41549 | 1 |
| 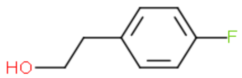 | 0 | 3316 | C8H9FO | -10.84 | 19 | 3 | -4.8963 | -3.4335 | -12.38 | 0.6411 | 10.501 | 9.3468 | -45.10518 | 0.71071 | 1 |
| 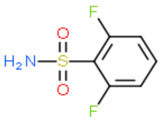 | 0 | 2949 | C6H5F2NO2S | -10.81 | 17 | 2 | -2.3594 | -2.5726 | -15.66 | 0 | 9.6411 | 6.2268 | -63.49522 | 0.68462 | 1 |
| 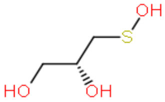 | 0 | 1472 | C3H8O3S | -10.81 | 15 | 6 | -8.0978 | -1.856 | -9.947 | 0 | 15.079 | 9.0318 | -36.90848 | 0.60286 | 1 |
| 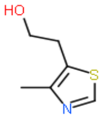 | 0 | 2665 | C6H9NOS | -10.8 | 18 | 3 | -5.9212 | -3.5547 | -8.007 | 0.7639 | 10.824 | 6.3549 | -44.65183 | 0.68065 | 1 |
| 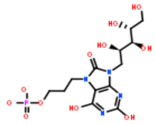 | 0 | 2716 | C13H21N4O11P | -10.77 | 48 | 14 | -15.241 | -4.2207 | -24.07 | 11.543 | 28.094 | 27.715 | -116.1477 | 1.33991 | 1 |
| 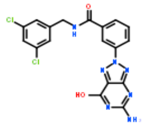 | 0 | 3225 | 45P | -10.77 | 42 | 2 | -10.351 | -6.3951 | -25.32 | 15.536 | 25.335 | 23.481 | -126.8088 | 1.85395 | 1 |
| 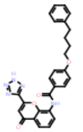 | 0 | 1219 | C27H23N5O4 | -10.75 | 59 | 5 | -4.2363 | -6.2942 | -36.19 | 0 | 26.043 | 27.036 | -121.7478 | 2.20874 | 1 |
| 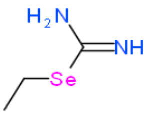 | 0 | 2314 | C3H8N2Se | -10.74 | 14 | 1 | -6.2524 | -1.7302 | -10.17 | 2.8163 | 12.554 | 7.1127 | -13.40323 | 0.40606 | 1 |

| | | | | | | | | | | | | | | | |
|---|---|---|---|---|---|---|---|---|---|---|---|---|---|---|---|
| 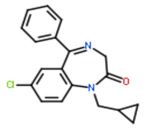 | 0 | 1382 | 1588 | -10.73 | 40 | 3 | -5.9773 | -5.8524 | -20.24 | 4.4648 | 14.904 | 19.551 | -118.6006 | 1.78732 | 1 |
| 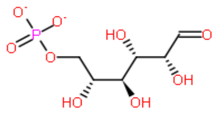 | 0 | 3233 | C6H13O9P | -10.72 | 27 | 11 | -10.637 | -1.6287 | -16.35 | 3.0393 | 15.819 | 20.214 | -88.03271 | 1.23422 | 1 |
| 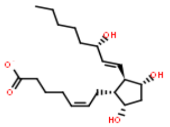 | 0 | 1030 | C24H45NO8 | -10.69 | 58 | 15 | -6.959 | -4.6165 | -24.68 | 6.6754 | 21.764 | 8.1214 | -59.45387 | 1.75819 | 1 |
| 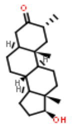 | 0 | 738 | C20H32O2 | -10.68 | 54 | 1 | -4.8886 | -4.9472 | -15.38 | 1.831 | 12.99 | 13.551 | -103.62 | 1.08976 | 1 |
| 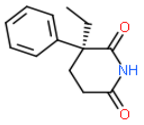 | 0 | 1243 | 1437 | -10.68 | 31 | 2 | -6.0185 | -3.8126 | -14.46 | 1.9454 | 12.549 | 14.823 | -69.93146 | 0.95274 | 1 |
| 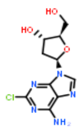 | 0 | 137 | 242 | -10.65 | 31 | 4 | -7.0515 | -3.7096 | -18.36 | 0.8811 | 19.641 | 15.715 | -53.27772 | 0.9747 | 1 |
| 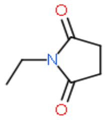 | 0 | 1672 | C6H9NO2 | -10.65 | 18 | 1 | -4.6701 | -2.9463 | -10.58 | 0.0777 | 8.1575 | 10.538 | -55.39904 | 0.56548 | 1 |
| 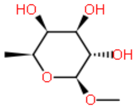 | 0 | 2875 | C7H14O5 | -10.65 | 26 | 4 | -7.2359 | -2.4249 | -11.02 | 1.4612 | 12.185 | 11.949 | -56.43464 | 0.87934 | 1 |
| 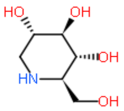 | 0 | 2887 | C6H13NO4 | -10.64 | 24 | 5 | -9.6425 | -2.1173 | -11.38 | 4.1204 | 17.85 | 11.661 | -67.31875 | 0.69533 | 1 |
| 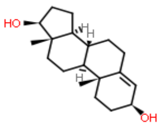 | 0 | 1322 | 1526 | -10.62 | 51 | 2 | -5.9897 | -4.5618 | -15.31 | 1.8121 | 14.486 | 14.774 | -88.47065 | 1.04218 | 1 |
| 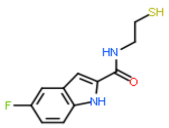 | 0 | 3055 | FXN | -10.59 | 27 | 3 | -4.5367 | -3.6956 | -18.35 | 3.4108 | 11.816 | 13.651 | -51.96758 | 0.89994 | 1 |

| | | | | | | | | | | | | | | | |
|---|---|---|---|---|---|---|---|---|---|---|---|---|---|---|---|
| 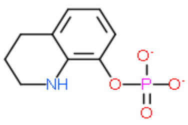 | 0 | 3186 | 790 | -10.58 | 25 | 1 | -8.1518 | -3.2846 | -14.48 | 0 | 17.137 | 20.116 | -99.44367 | 1.19899 | 1 |
| 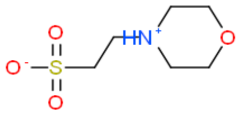 | 0 | 3449 | C6H13NO4S | -10.57 | 25 | 3 | -4.6435 | -3.2824 | -12.83 | 0 | 10.59 | 9.7837 | -62.34679 | 0.88358 | 1 |
| 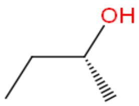 | 0 | 2330 | C4H10O | -10.56 | 15 | 2 | -6.0876 | -2.6271 | -7.623 | 1.0768 | 9.5032 | 7.9516 | -32.5757 | 0.52732 | 1 |
| 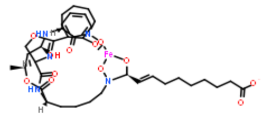 | 0 | 1692 | C36H51FeN5O12 | -10.56 | 104 | 9 | -5.4748 | -8.1224 | -28.99 | 7.03 | 29.142 | 9.4776 | -90.7868 | 2.48784 | 1 |
| 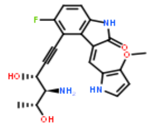 | 0 | 3378 | FMD | -10.54 | 48 | 6 | -5.488 | -5.3141 | -27.19 | 1.0137 | 21.68 | 20.656 | -96.08944 | 1.54474 | 1 |
| 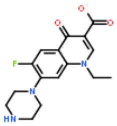 | 0 | 931 | 1059 | -10.51 | 40 | 2 | -3.7435 | -3.8138 | -16.97 | 2.5723 | 14.433 | 7.4451 | -56.3921 | 1.2551 | 1 |
| 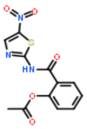 | 0 | 396 | 507 | -10.5 | 30 | 1 | -4.1086 | -4.1746 | -23.74 | 3.2651 | 15.567 | 18.482 | -88.06453 | 1.42319 | 1 |
| 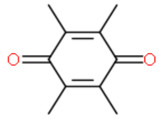 | 0 | 1693 | DQN | -10.5 | 24 | 0 | -2.5694 | -3.6014 | -14.7 | 0.4067 | 10.505 | 7.5562 | -67.75057 | 1.00844 | 1 |
| 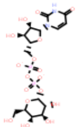 | 0 | 3164 | C15H24N2O17P2 | -10.48 | 58 | 15 | -11.199 | -3.8468 | -28.53 | 4.4781 | 28.252 | 24.367 | -156.0407 | 2.14315 | 1 |
| 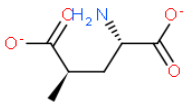 | 0 | 3093 | C6H10NO4 | -10.48 | 20 | 5 | -6.9789 | -2.0363 | -11.15 | 0.7457 | 12.292 | 10.635 | -76.11951 | 0.75977 | 1 |
| 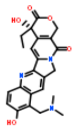 | 0 | 905 | 1030 | -10.45 | 54 | 4 | -5.4696 | -6.1471 | -26.95 | 1.9766 | 22.564 | 21.128 | -138.7192 | 2.04043 | 1 |

| | | | | | | | | | | | | | | | | |
|---|---|---|---|---|---|---|---|---|---|---|---|---|---|---|---|---|
| 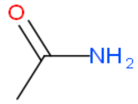 | 0 | 2450 | C2H5NO | -10.44 | 9 | 0 | -4.8565 | -1.0391 | -7.663 | 0.1825 | 6.9725 | 7.3706 | -14.77263 | 0.27561 | 1 |
| 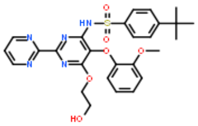 | 0 | 446 | 559 | -10.4 | 68 | 9 | -6.3043 | -8.3281 | -30.6 | 10.119 | 23.605 | 19.017 | -149.6258 | 2.36436 | 1 |
| 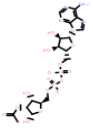 | 0 | 3142 | C17H25N5O15P2 | -10.4 | 62 | 14 | -11.083 | -3.3813 | -26.72 | 2.3934 | 31.47 | 19.994 | -168.7585 | 2.36104 | 1 |
| 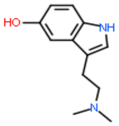 | 0 | 1251 | 1445 | -10.39 | 31 | 3 | -4.4321 | -4.3582 | -19.35 | 0.5996 | 13.157 | 16.763 | -71.56076 | 1.14523 | 1 |
| 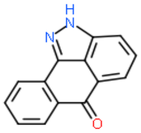 | 0 | 1561 | 537 | -10.38 | 25 | 0 | -2.6608 | -3.7017 | -17.02 | 0.9161 | 12.621 | 8.8407 | -89.82417 | 1.20128 | 1 |
| 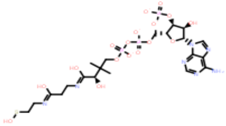 | 0 | 1622 | C21H36N7O17P3S | -10.37 | 81 | 24 | -14.791 | -4.5792 | -26.36 | 0 | 37.409 | 21.238 | -137.5765 | 3.01052 | 1 |
| 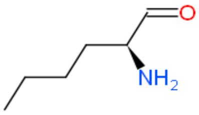 | 0 | 2922 | C6H13NO | -10.37 | 21 | 5 | -6.4282 | -2.6315 | -12.06 | 1.7994 | 12.74 | 9.2178 | -40.74559 | 0.80771 | 1 |
| 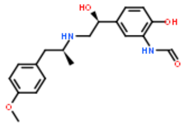 | 0 | 860 | 983 | -10.36 | 49 | 8 | -6.9853 | -4.7393 | -27.43 | 6.7961 | 20.475 | 20.72 | -105.0579 | 1.42968 | 1 |
| 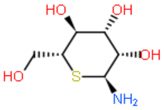 | 0 | 3082 | C6H13NO4S | -10.36 | 25 | 6 | -8.9926 | -1.6871 | -8.137 | 2.4223 | 13.468 | 9.8574 | -24.91248 | 0.75351 | 1 |
| 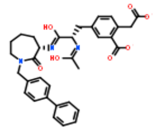 | 0 | 3355 | C33H35N3O7 | -10.36 | 76 | 13 | -12.779 | -6.7738 | -31.07 | 8.4902 | 26.717 | 37.344 | -159.7855 | 2.06688 | 1 |
| 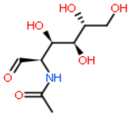 | 0 | 37 | C8H15NO6 | -10.35 | 30 | 10 | -8.3656 | -2.7639 | -17.46 | 5.471 | 15.12 | 14.403 | -86.36725 | 0.91796 | 1 |

| | | | | | | | | | | | | | | | |
|---|---|---|---|---|---|---|---|---|---|---|---|---|---|---|---|
| 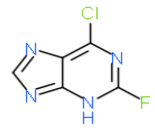 | 0 | 3182 | C5H2ClFN4 | -10.34 | 13 | 0 | -4.4823 | -2.1327 | -16.02 | 0.1602 | 12.683 | 13.381 | -41.05866 | 0.61064 | 1 |
| 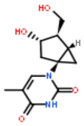 | 0 | 2620 | C12H16N2O4 | -10.33 | 34 | 4 | -6.3058 | -3.4841 | -17.07 | 5.6775 | 15.15 | 11.913 | -70.55401 | 1.29249 | 1 |
| 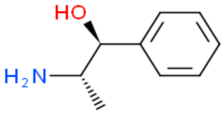 | 0 | 1289 | 1486 | -10.31 | 24 | 4 | -4.7674 | -3.2222 | -16 | 0.8453 | 15.474 | 8.1764 | -91.41731 | 0.99275 | 1 |
| 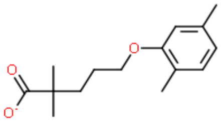 | 0 | 1110 | 1241 | -10.28 | 39 | 5 | -3.2268 | -4.7323 | -16.49 | 2.7352 | 13.906 | 3.7551 | -59.59034 | 1.43213 | 1 |
| 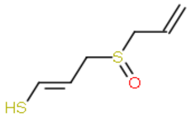 | 0 | 2595 | C6H10OS2 | -10.28 | 19 | 5 | -7.3673 | -3.0688 | -13.33 | 0.9816 | 13.175 | 15.567 | -36.85498 | 1.10217 | 1 |
| 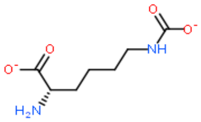 | 0 | 3438 | C7H14N2O4 | -10.28 | 25 | 8 | -11.234 | -2.2016 | -12.21 | 0 | 20.444 | 17.727 | -83.82864 | 1.2062 | 1 |
| 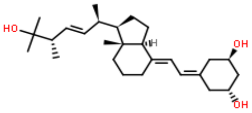 | 0 | 789 | C27H44O3 | -10.24 | 74 | 7 | -6.6717 | -6.2164 | -26.17 | 4.8433 | 21.438 | 20.903 | -112.1024 | 2.22278 | 1 |
| 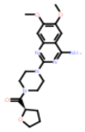 | 0 | 1032 | 1162 | -10.24 | 53 | 2 | -4.7129 | -7.3687 | -25.13 | 4.2616 | 21.471 | 18.025 | -141.4478 | 1.59145 | 1 |
| 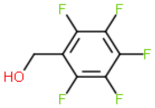 | 0 | 1497 | C7H3F5O | -10.22 | 16 | 2 | -2.3841 | -2.8449 | -12.78 | 0.7881 | 7.1963 | 5.1944 | -14.94556 | 0.52012 | 1 |
| 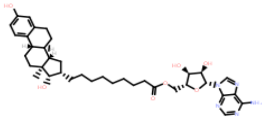 | 0 | 2065 | C37H51N5O7 | -10.22 | 100 | 15 | -8.6219 | -8.0143 | -36.47 | 18.099 | 26.277 | 21 | -206.6677 | 2.65798 | 1 |
| 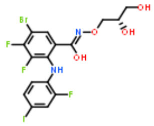 | 0 | 2802 | C16H13BrF3IN2O4 | -10.21 | 40 | 6 | -5.2662 | -6.5974 | -26.16 | 5.5447 | 20.228 | 17.576 | -117.6604 | 1.54198 | 1 |

| | | | | | | | | | | | | | | | |
|---|---|---|---|---|---|---|---|---|---|---|---|---|---|---|---|
| 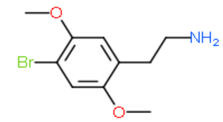 | 0 | 1333 | 1537 | -10.21 | 28 | 3 | -5.6312 | -4.5059 | -13.9 | 1.8626 | 13.599 | 11.883 | -70.4169 | 1.20351 | 1 |
| 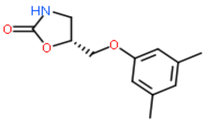 | 0 | 547 | 660 | -10.2 | 31 | 2 | -5.8222 | -4.3968 | -20.12 | 2.8879 | 15.586 | 19.654 | -107.9578 | 1.1688 | 1 |
| 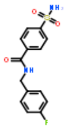 | 0 | 2165 | INV | -10.19 | 34 | 4 | -3.4109 | -4.4278 | -25.21 | 1.2778 | 14.569 | 18.98 | -79.44645 | 1.45191 | 1 |
| 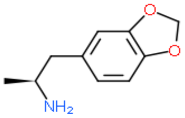 | 0 | 1308 | 1509 | -10.18 | 26 | 3 | -3.8605 | -3.7158 | -14.72 | 1.5242 | 12.448 | 7.1859 | -64.77868 | 1.05521 | 1 |
| 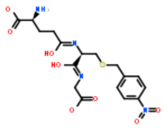 | 0 | 3330 | C17H22N4O8S | -10.18 | 50 | 16 | -5.5071 | -3.4155 | -28.83 | 2.3454 | 21.865 | 11.32 | -42.73222 | 1.73511 | 1 |
| 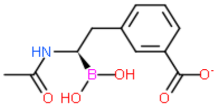 | 0 | 2337 | BJI | -10.16 | 31 | 7 | -9.5513 | -3.1697 | -14.26 | 0 | 19.054 | 18.03 | -71.61884 | 1.27861 | 1 |
| 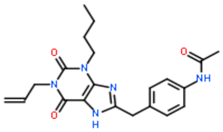 | 0 | 2946 | TSX | -10.15 | 54 | 7 | -4.8685 | -5.4844 | -27.01 | 6.5652 | 19.258 | 15.566 | -92.25111 | 1.56586 | 1 |
| 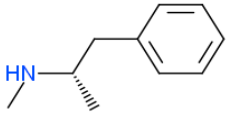 | 0 | 1373 | C10H15N | -10.14 | 26 | 3 | -3.5439 | -4.3275 | -13.27 | 0.1988 | 9.7808 | 8.6959 | -49.88126 | 1.03379 | 1 |
| 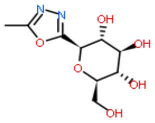 | 0 | 3029 | C9H14N2O6 | -10.13 | 31 | 6 | -8.3173 | -3.5966 | -14.76 | 2.0554 | 17.842 | 14.957 | -106.193 | 1.22939 | 1 |
| 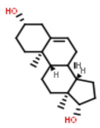 | 0 | 1320 | HMDB03818.mol | -10.11 | 51 | 2 | -6.6453 | -4.6008 | -15.18 | 3.2597 | 14.29 | 16.801 | -83.28162 | 1.12861 | 1 |
| 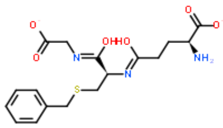 | 0 | 3253 | C17H23N3O6S | -10.08 | 48 | 15 | -11.559 | -4.7426 | -27.48 | 4.5096 | 23.997 | 30.479 | -103.8144 | 1.26848 | 1 |

| | | | | | | | | | | | | | | | |
|---|---|---|---|---|---|---|---|---|---|---|---|---|---|---|---|
| 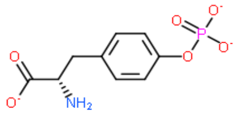 | 0 | 1726 | C9H12NO6P | -10.07 | 26 | 5 | -11.304 | -2.1664 | -16.47 | 5.9704 | 22.522 | 19.654 | -108.3228 | 1.23128 | 1 |
| 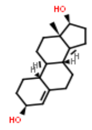 | 0 | 1350 | C18H28O2 | -10.06 | 48 | 2 | -5.9084 | -4.5243 | -14.91 | 1.9649 | 14.351 | 14.672 | -86.5832 | 1.04218 | 1 |
| 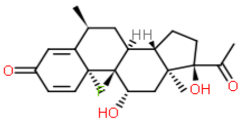 | 0 | 218 | 324 | -10.05 | 56 | 3 | -4.2474 | -4.6077 | -19.49 | 1.9598 | 16.126 | 12.431 | -90.58607 | 1.23898 | 1 |
| 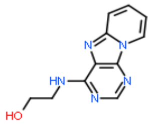 | 0 | 1758 | C11H11N5O | -10.05 | 28 | 3 | -5.1332 | -4.5601 | -20.68 | 2.2642 | 17.471 | 15.776 | -89.27292 | 1.15589 | 1 |
| 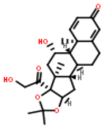 | 0 | 1127 | C24H32O6 | -10.05 | 62 | 4 | -8.7579 | -5.5029 | -18.58 | 6.5437 | 21.794 | 17.736 | -134.9303 | 1.64936 | 1 |
| 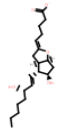 | 0 | 1109 | C20H32O5 | -10.05 | 56 | 12 | -6.611 | -6.6067 | -24.74 | 2.4211 | 20.466 | 17.94 | -121.4755 | 1.76139 | 1 |
| 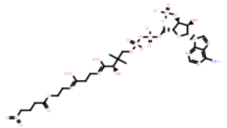 | 0 | 2926 | C25H41N8O19P3S | -10.04 | 93 | 27 | -20.383 | -6.9522 | -40.68 | 10.161 | 41.541 | 50.632 | -211.3203 | 2.52372 | 1 |
| 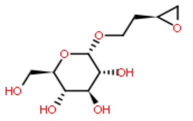 | 0 | 2366 | C10H18O7 | -10.02 | 35 | 9 | -10.265 | -2.7492 | -17.73 | 3.5282 | 19.469 | 20.302 | -29.15014 | 0.812 | 1 |
| 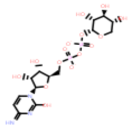 | 0 | 2758 | C14H23N3O15P2 | -10.02 | 55 | 14 | -14.538 | -3.8043 | -26.15 | 0 | 34.59 | 32.008 | -116.3519 | 1.77201 | 1 |
| 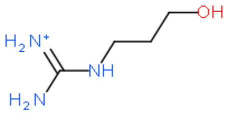 | 0 | 3286 | C4H12N3O | -9.977 | 20 | 4 | -7.572 | -1.6016 | -9.439 | 0 | 13.921 | 10.38 | -21.73421 | 0.68262 | 1 |
| 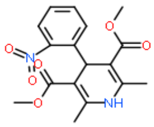 | 0 | 986 | 1115 | -9.972 | 43 | 2 | -4.643 | -5.4467 | -21.52 | 4.6207 | 15.949 | 16.675 | -45.96679 | 1.1637 | 1 |

| | | | | | | | | | | | | | | | |
|---|---|---|---|---|---|---|---|---|---|---|---|---|---|---|---|
| 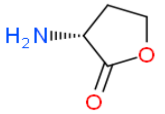 | 0 | 2347 | C4H7NO2 | -9.967 | 14 | 1 | -6.3889 | -1.7733 | -9.619 | 0.4605 | 12.888 | 9.7911 | -43.60282 | 0.46488 | 1 |
| 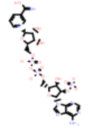 | 0 | 3127 | C21H28N7O17P3 | -9.957 | 73 | 16 | -11.264 | -5.5462 | -40.24 | 8.2027 | 39.933 | 27.307 | -178.7156 | 2.15722 | 1 |
| 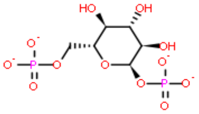 | 0 | 2538 | C6H14O12P2 | -9.932 | 30 | 8 | -14.534 | -1.6927 | -15.15 | 0 | 24.326 | 30.419 | -96.16255 | 1.31748 | 1 |
| 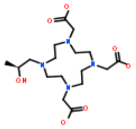 | 0 | 484 | 60714 | -9.914 | 57 | 9 | -11.36 | -4.3455 | -17.14 | 4.5214 | 23.28 | 20.164 | -125.1606 | 1.83434 | 1 |
| 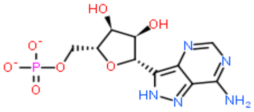 | 0 | 3129 | C10H14N5O7P | -9.912 | 35 | 6 | -13.385 | -2.9879 | -18.51 | 0 | 24.447 | 34.135 | -126.311 | 1.28753 | 1 |
| 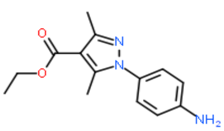 | 0 | 2865 | 5DE | -9.899 | 36 | 1 | -4.6141 | -5.4496 | -20.63 | 2.7621 | 16.549 | 17.26 | -111.8108 | 1.53888 | 1 |
| 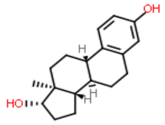 | 0 | 665 | 783 | -9.89 | 44 | 1 | -5.2012 | -4.0676 | -17.06 | 1.9295 | 15.252 | 14.941 | -84.02895 | 1.00086 | 1 |
| 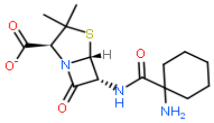 | 0 | 876 | 1000 | -9.877 | 45 | 4 | -9.443 | -3.9876 | -16.22 | 5.1649 | 22.834 | 15.584 | -122.4329 | 1.54804 | 1 |
| 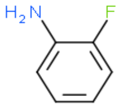 | 0 | 2139 | C6H6FN | -9.853 | 14 | 0 | -3.0261 | -2.9209 | -7.947 | 0.3314 | 8.6126 | 1.5883 | -28.53778 | 0.55982 | 1 |
| 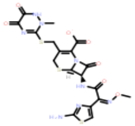 | 0 | 1082 | m | -9.843 | 53 | 5 | -14.662 | -5.1206 | -28.26 | 11.692 | 31.808 | 37.989 | -127.9786 | 2.48334 | 1 |
| 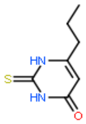 | 0 | 437 | 550 | -9.837 | 21 | 2 | -4.789 | -2.4374 | -15.91 | 0.3709 | 12.291 | 13.918 | -65.84516 | 0.90749 | 1 |

| | | | | | | | | | | | | | | | | |
|---|---|---|---|---|---|---|---|---|---|---|---|---|---|---|---|---|
| 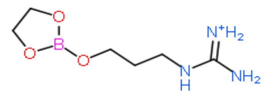 | 0 | 2813 | SBP | -9.827 | 28 | 4 | -7.6939 | -3.405 | -16.89 | 0.6757 | 18.823 | 18.013 | -55.01256 | 1.12169 | 1 |
| 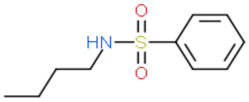 | 0 | 1814 | C10H15NO2S | -9.825 | 29 | 5 | -1.2428 | -4.2706 | -21.46 | 1.2174 | 10.119 | 9.5842 | -78.16779 | 1.1656 | 1 |
| 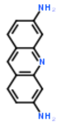 | 0 | 994 | 1123 | -9.823 | 27 | 0 | -4.8801 | -4.5207 | -15 | 2.3916 | 15.069 | 11.839 | -83.39152 | 1.14885 | 1 |
| 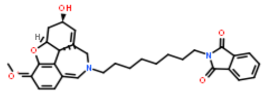 | 0 | 2392 | C32H37N2O5 | -9.822 | 76 | 10 | -2.8539 | -5.7427 | -35 | 12.937 | 18.141 | 13.454 | -95.16101 | 1.72441 | 1 |
| 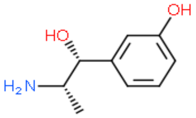 | 0 | 497 | 610 | -9.82 | 25 | 4 | -6.7714 | -3.1215 | -14.59 | 0.8165 | 17.061 | 12.585 | -63.20254 | 0.86132 | 1 |
| 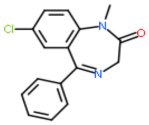 | 0 | 710 | 829 | -9.807 | 33 | 1 | -4.7953 | -5.2416 | -18.11 | 2.1171 | 12.592 | 19.138 | -128.6418 | 1.32333 | 1 |
| 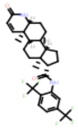 | 0 | 997 | C27H30F6N2O2 | -9.805 | 67 | 1 | -1.4002 | -7.895 | -26.21 | 1.9875 | 14.981 | 18.506 | -164.3033 | 1.92143 | 1 |
| 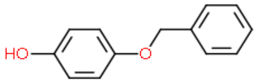 | 0 | 487 | 600 | -9.781 | 27 | 2 | -3.6684 | -3.8651 | -17.27 | 1.561 | 11.026 | 13.565 | -68.85239 | 0.77513 | 1 |
| 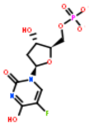 | 0 | 3401 | C9H12FN2O8P | -9.771 | 31 | 6 | -10.567 | -2.5775 | -18.79 | 3.6323 | 24.575 | 20.111 | -80.06754 | 1.28317 | 1 |
| 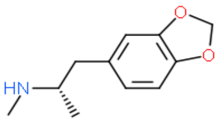 | 0 | 1258 | 1454 | -9.768 | 29 | 3 | -3.5614 | -4.5011 | -14.43 | 0.4739 | 12.113 | 8.3601 | -70.34589 | 1.20286 | 1 |
| 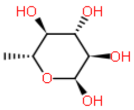 | 0 | 3412 | C6H12O5 | -9.763 | 23 | 4 | -6.8371 | -2.3653 | -13.01 | 0.5121 | 12.951 | 14.714 | -64.34917 | 0.71538 | 1 |

| | | | | | | | | | | | | | | | |
|---|---|---|---|---|---|---|---|---|---|---|---|---|---|---|---|
| 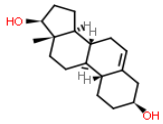 | 0 | 1259 | C18H28O2 | -9.744 | 48 | 2 | -4.3683 | -4.6893 | -16.71 | 1.5667 | 13.988 | 12.928 | -93.71651 | 1.0325 | 1 |
| 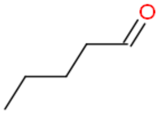 | 0 | 1685 | C5H10O | -9.735 | 16 | 3 | -4.3692 | -2.3987 | -10.23 | 0.3043 | 7.0951 | 8.8731 | -41.49674 | 0.38447 | 1 |
| 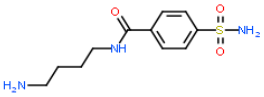 | 0 | 3340 | C11H17N3O3S | -9.728 | 35 | 7 | -5.1495 | -3.7125 | -23.92 | 1.7848 | 19.801 | 14.522 | -90.13821 | 1.38675 | 1 |
| 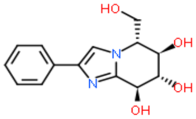 | 0 | 2565 | C14H16N2O4 | -9.722 | 36 | 6 | -7.4317 | -4.3672 | -18.22 | 4.0338 | 19.137 | 14.928 | -105.9459 | 1.30939 | 1 |
| 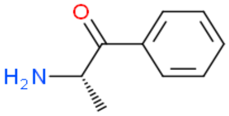 | 0 | 1356 | 1560 | -9.711 | 22 | 3 | -5.5786 | -2.7377 | -11.16 | 0.511 | 11.002 | 10.669 | -51.09771 | 0.44174 | 1 |
| 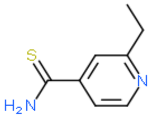 | 0 | 496 | 609 | -9.707 | 21 | 1 | -3.0875 | -3.0546 | -14.11 | 1.8594 | 10.611 | 6.9473 | -40.96804 | 0.77386 | 1 |
| 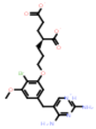 | 0 | 2514 | C20H24BrN4O6 | -9.706 | 55 | 10 | -10.206 | -6.9196 | -23.22 | 8.4145 | 25.694 | 20.689 | -140.4211 | 1.70367 | 1 |
| 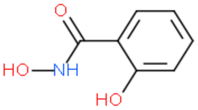 | 0 | 3454 | C7H7NO3 | -9.692 | 18 | 2 | -3.1189 | -1.8694 | -12.68 | 0 | 8.4634 | 6.9383 | -7.301088 | 0.59848 | 1 |
| 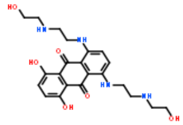 | 0 | 1074 | C22H28N4O6 | -9.681 | 60 | 12 | -8.2838 | -5.6543 | -32.25 | 6.9855 | 27.504 | 23.179 | -130.7205 | 1.96572 | 1 |
| 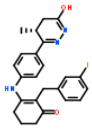 | 0 | 1432 | C24H24IN3O2 | -9.676 | 54 | 3 | -3.1932 | -7.6337 | -28.88 | 3.5602 | 20.719 | 19.58 | -121.967 | 1.92973 | 1 |
| 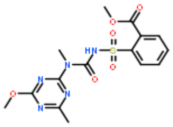 | 0 | 3304 | m | -9.671 | 44 | 4 | -4.5998 | -5.6556 | -20.2 | 0 | 19.82 | 13.172 | -83.30421 | 1.47998 | 1 |

| | | | | | | | | | | | | | | | | |
|---|---|---|---|---|---|---|---|---|---|---|---|---|---|---|---|---|
| 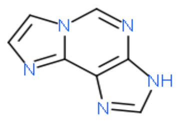 | 0 | 1716 | C7H5N5 | -9.648 | 17 | 0 | -5.3188 | -2.981 | -13.72 | 0.241 | 12.356 | 15.327 | -61.55768 | 0.90972 | 1 |
| 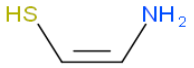 | 0 | 1732 | C2H5NS | -9.623 | 9 | 1 | -4.6933 | -1.3111 | -9.337 | 1.4952 | 7.7551 | 7.8077 | -2.713015 | 0.00836 | 1 |
| 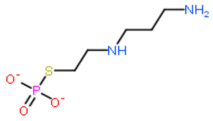 | 0 | 1014 | 1143 | -9.604 | 25 | 8 | -10.358 | -2.6114 | -14.47 | 1.506 | 20.243 | 18.083 | -78.99679 | 1.13107 | 1 |
| 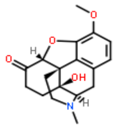 | 0 | 387 | 497 | -9.592 | 44 | 1 | -3.1307 | -4.1261 | -14.25 | 0 | 11.915 | 8.7974 | -50.69102 | 1.04593 | 1 |
| 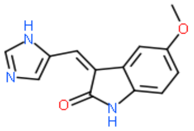 | 0 | 3096 | SU9 | -9.564 | 29 | 0 | -4.5019 | -4.1332 | -17.87 | 1.7034 | 15.617 | 14.749 | -54.52448 | 0.90807 | 1 |
| 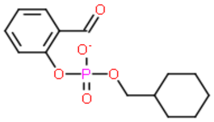 | 0 | 2801 | 219 | -9.54 | 38 | 4 | -9.0681 | -4.4475 | -20.14 | 7.3032 | 19.288 | 23.14 | -120.1654 | 1.61001 | 1 |
| 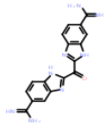 | 0 | 1649 | C17H14N8O | -9.519 | 40 | 0 | -9.7613 | -3.9625 | -20.96 | 1.8486 | 27.397 | 25.964 | -116.5024 | 1.76362 | 1 |
| 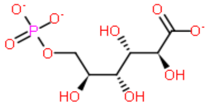 | 0 | 1445 | C6H13O10P | -9.512 | 27 | 11 | -13.814 | -1.4348 | -16.21 | 5.1365 | 20.349 | 26.761 | -68.86409 | 1.14817 | 1 |
| 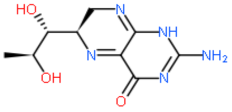 | 0 | 2290 | C9H13N5O3 | -9.506 | 30 | 4 | -7.4272 | -2.3481 | -17.76 | 4.2206 | 19.946 | 13.214 | -39.54102 | 0.85525 | 1 |
| 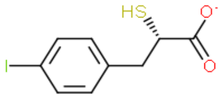 | 0 | 2297 | ISA | -9.504 | 21 | 4 | -3.2467 | -2.9855 | -13.33 | 0.0504 | 11.995 | 3.9623 | -38.58529 | 0.97289 | 1 |
| 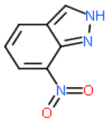 | 0 | 1957 | C7H5N3O2 | -9.498 | 17 | 1 | -0.8972 | -2.4543 | -16.05 | 0.4629 | 8.4195 | 4.9627 | -55.41277 | 0.66567 | 1 |

| | | | | | | | | | | | | | | | |
|---|---|---|---|---|---|---|---|---|---|---|---|---|---|---|---|
| 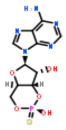 | 0 | 1568 | C10H12N5O5PS | -9.498 | 34 | 3 | -8.5597 | -4.0264 | -20.21 | 3.8944 | 27.794 | 15.283 | -101.5413 | 1.56461 | 1 |
| 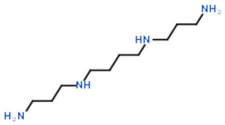 | 0 | 23 | 127 | -9.488 | 40 | 13 | -8.894 | -4.8533 | -21.38 | 4.2391 | 21.876 | 16.511 | -68.93148 | 1.46258 | 1 |
| 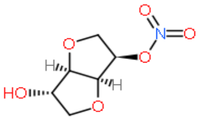 | 0 | 896 | 1020 | -9.466 | 22 | 3 | -4.5866 | -2.4651 | -13.07 | 2.2247 | 12.854 | 6.182 | -67.08056 | 0.80664 | 1 |
| 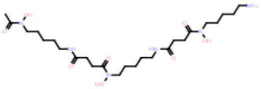 | 0 | 629 | C25H48N6O8 | -9.462 | 87 | 30 | -14.487 | -7.2284 | -36.1 | 16.269 | 24.169 | 33.705 | -176.8453 | 2.84685 | 1 |
| 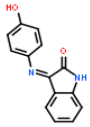 | 0 | 3299 | LI7 | -9.456 | 28 | 0 | -5.288 | -4.1552 | -18.06 | 1.7598 | 17.066 | 16.532 | -91.4154 | 1.44865 | 1 |
| 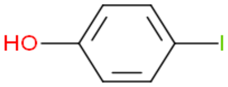 | 0 | 2697 | C6H5IO | -9.451 | 13 | 0 | -3.6384 | -2.5163 | -11.3 | 0.2542 | 8.2924 | 9.6512 | -39.0749 | 0.61599 | 1 |
| 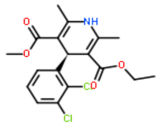 | 0 | 899 | C18H19Cl2NO4 | -9.449 | 44 | 2 | -4.6162 | -6.0765 | -23.2 | 6.6929 | 16.229 | 18.348 | -56.94176 | 1.36164 | 1 |
| 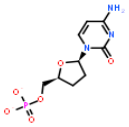 | 0 | 2584 | C9H14N3O6P | -9.432 | 31 | 4 | -10.275 | -2.8424 | -17.14 | 3.297 | 21.251 | 23.174 | -73.2375 | 1.10892 | 1 |
| 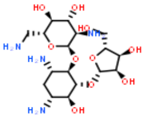 | 0 | 3266 | C17H34N4O10 | -9.416 | 65 | 16 | -16.165 | -4.0439 | -21.03 | 9.6783 | 31.009 | 25.091 | -128.2113 | 1.7594 | 1 |
| 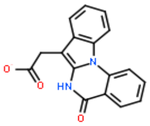 | 0 | 1547 | IQA | -9.415 | 33 | 2 | -5.2716 | -3.9309 | -17.17 | 2.3151 | 15.734 | 14.146 | -86.81403 | 1.00212 | 1 |
| 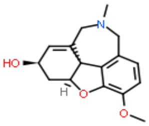 | 0 | 560 | 674 | -9.395 | 42 | 1 | -3.6909 | -4.5814 | -19.91 | 0 | 15.763 | 15.91 | -60.19209 | 1.02884 | 1 |

| | | | | | | | | | | | | | | | |
|---|---|---|---|---|---|---|---|---|---|---|---|---|---|---|---|
| 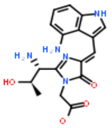 | 0 | 1433 | C17H19N5O4 | -9.379 | 44 | 6 | -10.181 | -4.0804 | -23.78 | 8.0184 | 27.502 | 20.99 | -130.7067 | 1.4564 | 1 |
| 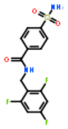 | 0 | 1969 | IOE | -9.37 | 34 | 4 | -1.6251 | -3.7637 | -25.42 | 3.3216 | 13.838 | 11.986 | -66.51469 | 1.23865 | 1 |
| 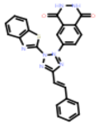 | 0 | 1668 | C24H16N7O2S | -9.353 | 50 | 1 | -5.4367 | -6.1848 | -31.55 | 6.2986 | 21.144 | 30.095 | -114.6576 | 1.8547 | 1 |
| 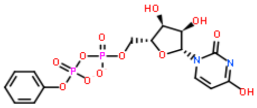 | 0 | 2496 | C15H18N2O12P2 | -9.353 | 47 | 11 | -12.004 | -3.5844 | -27.28 | 0.7939 | 27.819 | 34.479 | -150.0784 | 1.92476 | 1 |
| 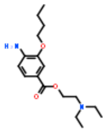 | 0 | 772 | C17H28N2O3 | -9.338 | 50 | 8 | -4.7424 | -4.9512 | -22.39 | 5.4044 | 17.471 | 10.946 | -17.29845 | 1.33244 | 1 |
| 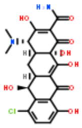 | 0 | 505 | C21H21ClN2O8 | -9.326 | 53 | 3 | -6.88 | -4.1051 | -27.41 | 0 | 26.887 | 24.652 | -119.3598 | 1.78452 | 1 |
| 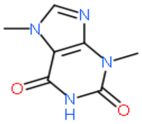 | 0 | 1220 | C7H8N4O2 | -9.314 | 21 | 0 | -3.671 | -2.891 | -15.05 | 0.0528 | 14.681 | 8.9713 | -78.58352 | 0.94656 | 1 |
| 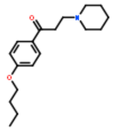 | 0 | 532 | 645 | -9.301 | 48 | 6 | -6.1179 | -6.5756 | -22.04 | 6.8772 | 16.432 | 19.049 | -116.6251 | 1.89474 | 1 |
| 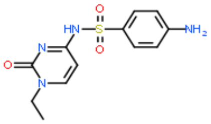 | 0 | 1150 | 1298 | -9.285 | 34 | 3 | -6.2163 | -4.1838 | -24.21 | 2.5688 | 18.393 | 24.723 | -89.49022 | 1.3465 | 1 |
| 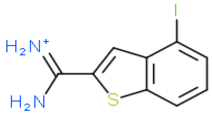 | 0 | 2819 | ESI | -9.275 | 21 | 0 | -3.8403 | -2.9936 | -17.36 | 0.4016 | 11.854 | 16.171 | -37.28486 | 0.7434 | 1 |
| 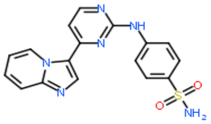 | 0 | 1949 | HDT | -9.273 | 40 | 2 | -3.9991 | -5.3518 | -26.24 | 4.0264 | 20.368 | 17.851 | -93.49477 | 1.74278 | 1 |

| | | | | | | | | | | | | | | | |
|---|---|---|---|---|---|---|---|---|---|---|---|---|---|---|---|
| 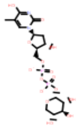 | 0 | 2843 | C15H24N2O15P2 | -9.269 | 56 | 13 | -15.385 | -4.1349 | -23.56 | 1.6621 | 33.33 | 33.628 | -127.6404 | 1.91048 | 1 |
| 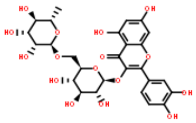 | 0 | 1484 | C27H30O16 | -9.244 | 73 | 10 | -14.285 | -4.49 | -27.34 | 14.565 | 36.421 | 23.019 | -102.9404 | 1.84621 | 1 |
| 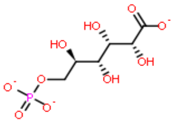 | 0 | 1834 | C6H13O10P | -9.235 | 27 | 11 | -11.324 | -1.5814 | -13.55 | 0 | 17.61 | 21.547 | -86.73055 | 1.1651 | 1 |
| 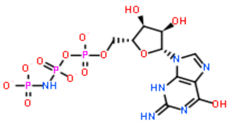 | 0 | 1840 | C10H17N6O13P3 | -9.222 | 45 | 11 | -13.017 | -1.9374 | -28.99 | 0 | 28.528 | 39.403 | -90.90895 | 1.84756 | 1 |
| 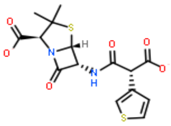 | 0 | 1400 | 1607 | -9.209 | 39 | 5 | -8.6834 | -4.6439 | -24.64 | 4.9732 | 23.275 | 25.871 | -120.4415 | 1.64429 | 1 |
| 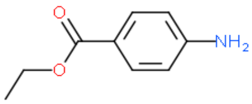 | 0 | 958 | 1086 | -9.195 | 23 | 1 | -4.3155 | -3.7008 | -15.64 | 0.9449 | 13.757 | 12.67 | -55.26016 | 1.04078 | 1 |
| 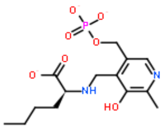 | 0 | 2677 | C14H23N2O7P | -9.19 | 44 | 10 | -9.9939 | -4.7411 | -18.38 | 0 | 21.774 | 23.216 | -112.3131 | 1.80076 | 1 |
| 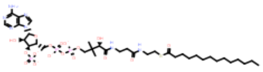 | 0 | 1933 | C35H62N7O17P3S | -9.176 | 121 | 33 | -16.13 | -8.2647 | -50.62 | 7.4797 | 35.435 | 55.954 | -241.0623 | 3.00621 | 1 |
| 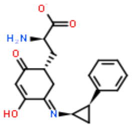 | 0 | 1447 | C18H20N2O4 | -9.149 | 43 | 6 | -10.002 | -4.4236 | -20.49 | 5.5175 | 23.003 | 23.407 | -127.3499 | 1.61329 | 1 |
| 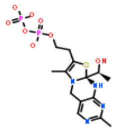 | 0 | 3035 | C14H22N4O8P2S | -9.145 | 48 | 8 | -11.775 | -5.0339 | -24.04 | 4.678 | 20.965 | 37.473 | -105.4232 | 1.48454 | 1 |
| 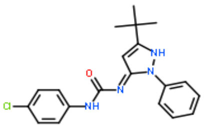 | 0 | 1584 | L10 | -9.119 | 47 | 4 | -1.9476 | -6.4847 | -26.75 | 2.9784 | 14.299 | 18.173 | -86.99067 | 1.53744 | 1 |

| | | | | | | | | | | | | | | | |
|---|---|---|---|---|---|---|---|---|---|---|---|---|---|---|---|
| 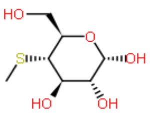 | 0 | 2444 | C7H14O5S | -9.109 | 27 | 6 | -9.7561 | -3.1098 | -10.24 | 2.465 | 18.69 | 13.328 | -64.38544 | 0.80882 | 1 |
| 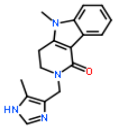 | 0 | 846 | 969 | -9.104 | 40 | 2 | -1.3463 | -5.9782 | -25.3 | 1.2065 | 13.375 | 17.654 | -109.5473 | 1.35211 | 1 |
| 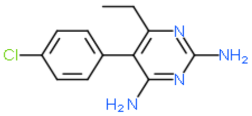 | 0 | 100 | 205 | -9.1 | 30 | 2 | -4.8438 | -5.2339 | -16.28 | 0.1281 | 16.056 | 14.585 | -79.16249 | 1.22447 | 1 |
| 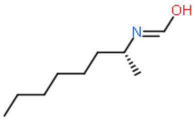 | 0 | 1888 | C9H19NO | -9.095 | 30 | 7 | -8.0711 | -4.0412 | -13.07 | 6.421 | 13.196 | 13.868 | -70.85252 | 1.00826 | 1 |
| 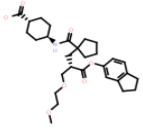 | 0 | 503 | C29H41NO7 | -9.086 | 77 | 11 | -11.762 | -7.6445 | -18.38 | 18.325 | 21.14 | 16.243 | -163.446 | 2.05024 | 1 |
| 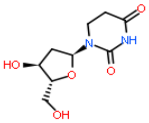 | 0 | 3217 | C9H14N2O5 | -9.081 | 30 | 4 | -8.3656 | -2.7305 | -15.86 | 0.8917 | 18.877 | 19.214 | -101.9389 | 1.24939 | 1 |
| 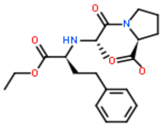 | 0 | 471 | C20H28N2O5 | -9.073 | 54 | 10 | -9.0548 | -6.4668 | -25.23 | 10.228 | 20.774 | 23.766 | -128.1048 | 1.70851 | 1 |
| 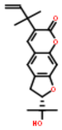 | 0 | 1956 | BRZ | -9.045 | 45 | 4 | -4.2493 | -5.7253 | -24.88 | 6.9227 | 17.094 | 16.734 | -126.5257 | 1.43243 | 1 |
| 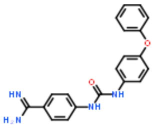 | 0 | 3012 | GP8 | -9.041 | 44 | 4 | -6.2926 | -6.401 | -27.57 | 6.5818 | 22.197 | 23.848 | -122.7895 | 1.67867 | 1 |
| 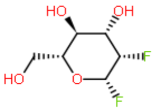 | 0 | 2060 | C6H10F2O4 | -9.032 | 22 | 4 | -6.2378 | -1.8713 | -9.779 | 2.9035 | 12.332 | 6.6542 | -37.79643 | 0.66069 | 1 |
| 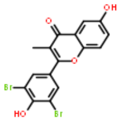 | 0 | 1615 | FL8 | -9.029 | 32 | 0 | -3.1552 | -5.1054 | -19.51 | 1.9319 | 16.735 | 12.379 | -104.5385 | 1.61421 | 1 |

| | | | | | | | | | | | | | | | |
|---|---|---|---|---|---|---|---|---|---|---|---|---|---|---|---|
| 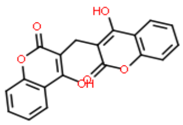 | 0 | 161 | C19H12O6 | -9.023 | 37 | 2 | -0.8412 | -4.9812 | -25.7 | 0 | 15.379 | 14.249 | -109.0534 | 1.53925 | 1 |
| 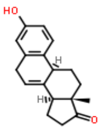 | 0 | 1940 | EQI | -9 | 40 | 0 | -4.01 | -4.2127 | -16.38 | 1.3511 | 13.216 | 14.584 | -96.64412 | 0.97162 | 1 |
| 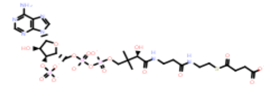 | 0 | 3342 | C25H40N7O19P3S | -8.999 | 90 | 24 | -15.834 | -6.1737 | -44.36 | 14.717 | 41.643 | 38.031 | -228.0107 | 2.83039 | 1 |
| 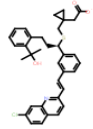 | 0 | 361 | 471 | -8.982 | 76 | 11 | -8.8864 | -8.6562 | -34 | 10.337 | 23.337 | 34.841 | -184.2393 | 2.55428 | 1 |
| 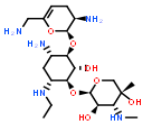 | 0 | 833 | C21H41N5O7 | -8.975 | 74 | 14 | -7.3217 | -6.7996 | -28.26 | 4.84 | 21.404 | 22.737 | -123.3909 | 1.78176 | 1 |
| 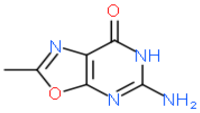 | 0 | 2689 | C6H6N4O2 | -8.971 | 18 | 0 | -5.3167 | -2.0187 | -16.29 | 0 | 16.481 | 14.798 | -69.02561 | 0.82262 | 1 |
| 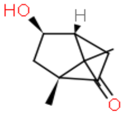 | 0 | 2521 | CAH | -8.967 | 28 | 1 | -3.39 | -3.099 | -9.71 | 1.3222 | 8.2692 | 5.7586 | -42.85447 | 0.53491 | 1 |
| 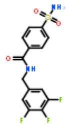 | 0 | 2564 | IOF | -8.962 | 34 | 4 | -2.7586 | -4.7335 | -25.53 | 3.2474 | 16.306 | 15.365 | -103.6747 | 1.45761 | 1 |
| 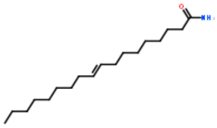 | 0 | 3422 | C18H35NO | -8.956 | 55 | 15 | -9.8094 | -6.0565 | -21.55 | 6.6717 | 18.399 | 22.219 | -115.0181 | 1.50771 | 1 |
| 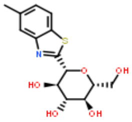 | 0 | 2931 | TH1 | -8.95 | 38 | 6 | -5.335 | -4.2607 | -22.7 | 3.9429 | 19.871 | 13.996 | -56.08219 | 1.16652 | 1 |
| 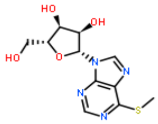 | 0 | 2596 | C11H14N4O4S | -8.936 | 34 | 5 | -3.776 | -3.2355 | -16.01 | 0.7454 | 15.777 | 5.3281 | -21.59991 | 0.99527 | 1 |

| | | | | | | | | | | | | | | | |
|---|---|---|---|---|---|---|---|---|---|---|---|---|---|---|---|
| 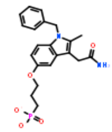 | 0 | 2234 | 8IN | -8.903 | 52 | 8 | -9.398 | -5.4397 | -26.7 | 6.4179 | 23.625 | 28.558 | -161.2444 | 1.68587 | 1 |
| 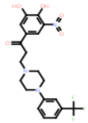 | 0 | 3011 | BIA | -8.891 | 51 | 4 | -4.6975 | -6.4178 | -26.54 | 2.7637 | 18.156 | 24.482 | -134.8235 | 1.85049 | 1 |
| 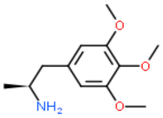 | 0 | 1314 | 1516 | -8.875 | 35 | 3 | -5.1494 | -5.0552 | -17.3 | 6.6045 | 16.727 | 9.894 | -93.72135 | 1.36158 | 1 |
| 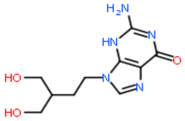 | 0 | 193 | 299 | -8.873 | 33 | 7 | -10.458 | -2.9087 | -19.49 | 1.8632 | 22.68 | 25.298 | -98.88043 | 1.27332 | 1 |
| 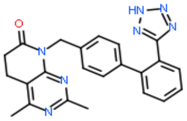 | 0 | 1178 | 1349 | -8.867 | 52 | 2 | -5.6551 | -6.0106 | -28.67 | 5.1088 | 23.325 | 24.818 | -182.8991 | 1.8146 | 1 |
| 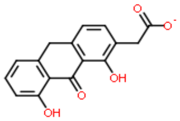 | 0 | 1780 | ADL | -8.851 | 32 | 2 | -8.2369 | -3.6352 | -20.57 | 3.3864 | 19.22 | 26.283 | -134.0469 | 1.38923 | 1 |
| 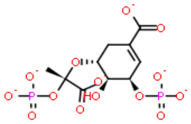 | 0 | 2803 | C10H16O14P2 | -8.851 | 36 | 9 | -15.519 | -2.4089 | -16.22 | 1.38 | 24.503 | 35.82 | -112.1767 | 1.4072 | 1 |
| 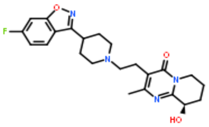 | 0 | 1133 | 1267 | -8.841 | 58 | 5 | -5.6514 | -6.9364 | -28.79 | 2.4021 | 22.797 | 26.301 | -149.3115 | 1.77156 | 1 |
| 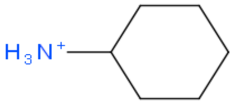 | 0 | 2691 | C6H14N | -8.831 | 21 | 0 | -3.6205 | -1.8764 | -8.593 | 0.3913 | 7.5368 | 6.5019 | -27.79896 | 0.42611 | 1 |
| 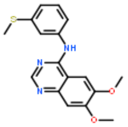 | 0 | 2680 | MSQ | -8.831 | 40 | 0 | -4.037 | -6.1947 | -23.14 | 1.1235 | 19.541 | 20.217 | -109.3898 | 1.84925 | 1 |
| 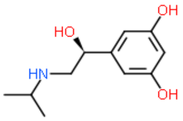 | 0 | 697 | 816 | -8.83 | 32 | 5 | -5.0281 | -3.8309 | -15.29 | 3.1631 | 13.222 | 10.511 | -62.83032 | 0.79796 | 1 |

| | | | | | | | | | | | | | | |
|---|---|---|---|---|---|---|---|---|---|---|---|---|---|---|
| 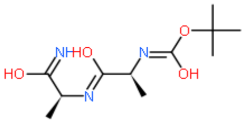 | 0 | 3397 | C11H21N3O4 | -8.825 | 39 | 8 | -8.7537 | -4.4095 | -18.6 | 7.4354 | 18.384 | 17.917 | -103.5894 | 1.50931 | 1 |
| 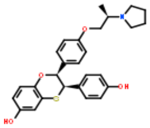 | 0 | 2338 | C27H29NO4S | -8.821 | 62 | 5 | -5.8759 | -7.2158 | -27.91 | 10.468 | 21.197 | 20.624 | -115.3425 | 1.90749 | 1 |
| 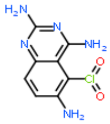 | 0 | 1694 | CLZ | -8.812 | 24 | 1 | -2.0495 | -3.2154 | -20.47 | 0 | 15.499 | 10.26 | -55.28167 | 0.83645 | 1 |
| 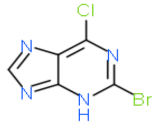 | 0 | 1492 | C5H2BrClN4 | -8.806 | 13 | 0 | -2.1766 | -1.8547 | -14.91 | 0.0265 | 10.726 | 7.266 | 12.282273 | 0.60115 | 1 |
| 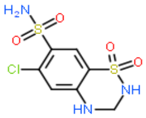 | 0 | 875 | 999 | -8.802 | 25 | 2 | -1.8035 | -2.6428 | -21.9 | 0.1766 | 13.121 | 12.56 | -91.23826 | 1.31508 | 1 |
| 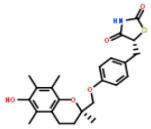 | 0 | 92 | 197 | -8.795 | 58 | 4 | -8.2528 | -6.8228 | -24.42 | 4.3619 | 22.239 | 29.361 | -157.2663 | 1.74101 | 1 |
| 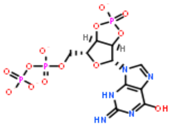 | 0 | 2539 | C10H14N5O13P3 | -8.791 | 41 | 7 | -17.211 | -2.6572 | -26.46 | 3.3422 | 30.745 | 51.108 | -124.7292 | 1.40637 | 1 |
| 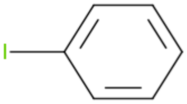 | 0 | 1996 | C6H5I | -8.791 | 12 | 0 | 0 | -3.0517 | -12.92 | 0.2212 | 3.0692 | 5.6634 | -13.8489 | 0.39272 | 1 |
| 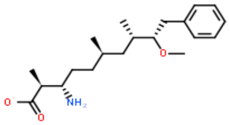 | 0 | 2236 | ADD | -8.783 | 56 | 12 | -7.3735 | -6.277 | -23.57 | 0.5537 | 17.469 | 25.776 | -112.9601 | 1.42772 | 1 |
| 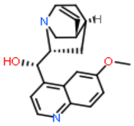 | 0 | 787 | 908 | -8.782 | 48 | 4 | -4.4537 | -5.9959 | -21.43 | 3.0697 | 15.257 | 18.557 | -84.31812 | 1.22946 | 1 |
| 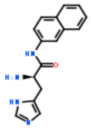 | 0 | 1703 | HBN | -8.763 | 37 | 4 | -4.6739 | -5.2474 | -26.71 | 1.4724 | 19.045 | 23.821 | -103.7248 | 1.36107 | 1 |

| | | | | | | | | | | | | | | | |
|---|---|---|---|---|---|---|---|---|---|---|---|---|---|---|---|
| 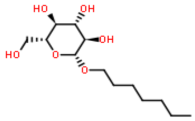 | 0 | 3013 | B7G | -8.754 | 45 | 12 | -7.2946 | -4.8495 | -18.87 | 5.8162 | 18.091 | 11.46 | -111.8225 | 1.47124 | 1 |
| 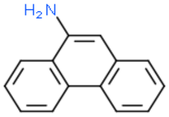 | 0 | 3043 | 9AP | -8.732 | 26 | 0 | -2.6015 | -4.5132 | -13.76 | 1.7418 | 11.158 | 7.9162 | -74.2343 | 1.02135 | 1 |
| 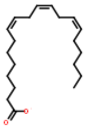 | 0 | 50 | 5280581 | -8.721 | 55 | 15 | -8.4175 | -6.3704 | -26.76 | 6.2765 | 16.288 | 28.208 | -118.919 | 1.60635 | 1 |
| 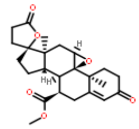 | 0 | 586 | C24H30O6 | -8.712 | 60 | 1 | -3.0667 | -5.096 | -20.77 | 2.9394 | 15.647 | 13.841 | -104.3316 | 1.40317 | 1 |
| 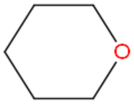 | 0 | 2148 | C5H10O | -8.707 | 16 | 0 | -3.7658 | -3.0046 | -8.458 | 0.0418 | 7.8169 | 8.1878 | -50.11339 | 0.55352 | 1 |
| 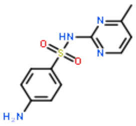 | 0 | 1377 | 1581 | -8.704 | 30 | 3 | -2.3125 | -4.2239 | -21.78 | 2.1721 | 14.177 | 12.152 | -74.34567 | 1.16701 | 1 |
| 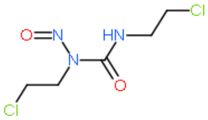 | 0 | 157 | 262 | -8.693 | 21 | 6 | -4.6156 | -3.5919 | -14.79 | 1.3896 | 13.261 | 8.7928 | -69.17165 | 0.90343 | 1 |
| 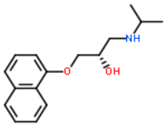 | 0 | 458 | C16H21NO2 | -8.667 | 40 | 6 | -6.5861 | -5.1173 | -20.34 | 7.0878 | 15.12 | 19.049 | -73.17939 | 1.08945 | 1 |
| 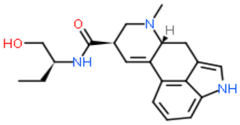 | 0 | 246 | 8226 | -8.667 | 50 | 5 | -4.7724 | -6.1295 | -23.67 | 2.5569 | 18.452 | 19.417 | -112.2373 | 1.72174 | 1 |
| 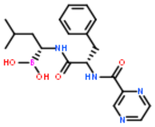 | 0 | 83 | C19H25BN4O4 | -8.662 | 53 | 10 | -6.4034 | -6.0661 | -24.62 | 2.8618 | 19.818 | 20.672 | -101.9645 | 1.48277 | 1 |
| 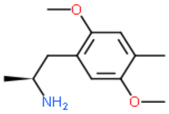 | 0 | 1324 | 1528 | -8.63 | 34 | 3 | -4.9353 | -4.9548 | -16.57 | 5.7456 | 14.997 | 10.992 | -83.40981 | 1.34128 | 1 |

| | | | | | | | | | | | | | | | |
|---|---|---|---|---|---|---|---|---|---|---|---|---|---|---|---|
| 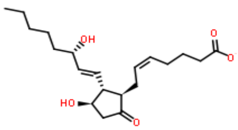 | 0 | 796 | C20H32O5 | -8.624 | 56 | 14 | -9.5635 | -4.8428 | -23.61 | 3.5526 | 22.927 | 22.268 | -123.6725 | 1.78205 | 1 |
| 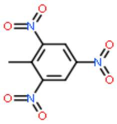 | 0 | 1465 | TNL | -8.605 | 21 | 3 | 0 | -2.373 | -19.25 | 0.5907 | 7.5502 | 6.6985 | -66.44646 | 1.02469 | 1 |
| 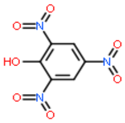 | 0 | 3300 | C6H3N3O7 | -8.605 | 19 | 3 | -0.4528 | -1.8591 | -18.42 | 0 | 8.8509 | 5.7132 | -62.79554 | 0.96731 | 1 |
| 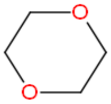 | 0 | 2993 | C4H8O2 | -8.598 | 14 | 0 | -3.864 | -2.7707 | -8.356 | 0 | 8.5317 | 7.5631 | -48.32834 | 0.5254 | 1 |
| 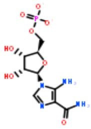 | 0 | 1486 | C9H15N4O8P | -8.578 | 35 | 6 | -10.559 | -2.345 | -17.87 | 0 | 21.966 | 26.525 | -78.72286 | 1.21656 | 1 |
| 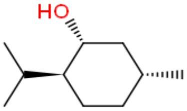 | 0 | 706 | menthol | -8.577 | 31 | 2 | -5.2129 | -4.0105 | -11.34 | 1.0525 | 11.911 | 11.937 | -84.25839 | 1.08698 | 1 |
| 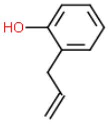 | 0 | 2263 | C9H10O | -8.553 | 20 | 2 | -2.542 | -3.5943 | -14.63 | 1.191 | 9.2147 | 9.2253 | -53.89661 | 0.67886 | 1 |
| 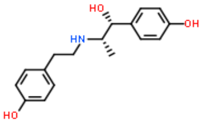 | 0 | 747 | 867 | -8.549 | 42 | 7 | -5.344 | -4.4474 | -22.67 | 4.9346 | 20.19 | 12.599 | -76.96178 | 1.52622 | 1 |
| 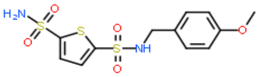 | 0 | 3187 | AL9 | -8.543 | 36 | 6 | -2.6448 | -4.1186 | -27.15 | 4.4813 | 15.856 | 14.96 | -118.8044 | 1.31667 | 1 |
| 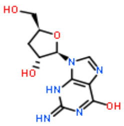 | 0 | 3260 | C10H13N5O4 | -8.535 | 32 | 5 | -7.4847 | -2.353 | -21.33 | 3.6437 | 22.413 | 17.141 | -60.72406 | 0.86164 | 1 |
| 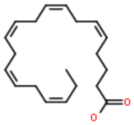 | 0 | 55 | C20H30O2 | -8.53 | 51 | 13 | -8.2357 | -6.3011 | -28.27 | 10.585 | 15.185 | 29.182 | -110.1929 | 1.46006 | 1 |

| | | | | | | | | | | | | | | | |
|---|---|---|---|---|---|---|---|---|---|---|---|---|---|---|---|
| 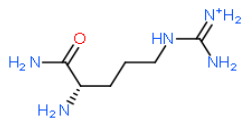 | 0 | 2041 | C6H16N5O | -8.518 | 28 | 6 | -8.7474 | -1.521 | -16.38 | 0.8977 | 22.105 | 15.604 | -50.388 | 0.91248 | 1 |
| 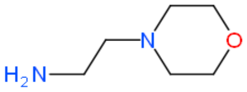 | 0 | 2783 | C6H14N2O | -8.511 | 23 | 3 | -5.1165 | -3.215 | -11.14 | 0.4163 | 12.918 | 9.0732 | -53.16834 | 0.77111 | 1 |
| 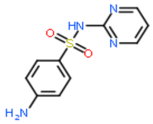 | 0 | 252 | 359 | -8.508 | 27 | 3 | -2.3105 | -3.915 | -19.06 | 1.9037 | 13.114 | 9.4612 | -72.3053 | 1.06384 | 1 |
| 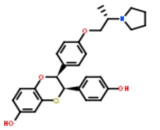 | 0 | 2430 | C27H29NO4S | -8.504 | 62 | 5 | -6.3492 | -6.0322 | -22.58 | 8.0563 | 21.966 | 14.955 | -82.77023 | 2.17107 | 1 |
| 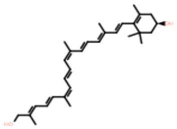 | 0 | 1997 | C30H42O2 | -8.5 | 74 | 3 | -3.9417 | -8.3209 | -25.01 | 8.6364 | 19.038 | 16.361 | -96.95946 | 2.25508 | 1 |
| 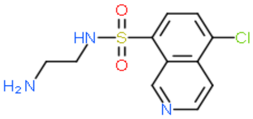 | 0 | 3336 | CKI | -8.497 | 30 | 5 | -3.443 | -3.3188 | -19.75 | 4.0329 | 14.765 | 8.6214 | -38.15096 | 0.92047 | 1 |
| 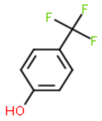 | 0 | 3261 | C7H5F3O | -8.497 | 16 | 0 | -3.5405 | -2.6144 | -11.47 | 0.5182 | 8.0922 | 11.066 | -38.65878 | 0.64036 | 1 |
| 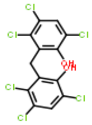 | 0 | 639 | 756 | -8.496 | 27 | 2 | -1.1412 | -4.7282 | -20.72 | 2.7325 | 10.273 | 11.724 | -36.56391 | 0.96355 | 1 |
| 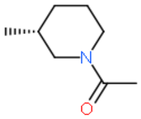 | 0 | 1526 | C8H15NO | -8.493 | 25 | 1 | -4.9249 | -3.7504 | -9.96 | 0.3729 | 9.7554 | 12.575 | -56.14724 | 0.87988 | 1 |
| 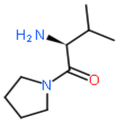 | 0 | 1657 | A3M | -8.473 | 30 | 4 | -3.4727 | -3.9605 | -15.49 | 0.0497 | 13.054 | 9.4423 | -80.11386 | 1.01874 | 1 |
| 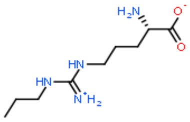 | 0 | 2365 | C9H21N4O2 | -8.471 | 35 | 8 | -9.7862 | -3.3191 | -17.34 | 2.0965 | 21.977 | 20.163 | -75.77708 | 1.19209 | 1 |

| | | | | | | | | | | | | | | | | |
|---|---|---|---|---|---|---|---|---|---|---|---|---|---|---|---|---|
| 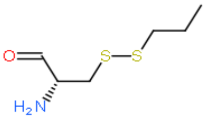 | 0 | 3303 | PR3 | -8.458 | 23 | 7 | -7.2458 | -2.7729 | -15.81 | 2.9805 | 16.424 | 13.998 | -46.14091 | 0.9019 | 1 |
| 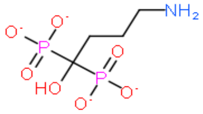 | 0 | 517 | 630 | -8.457 | 23 | 7 | -16.084 | -1.5426 | -13.8 | 3.6152 | 20.624 | 38.226 | -67.42313 | 0.70307 | 1 |
| 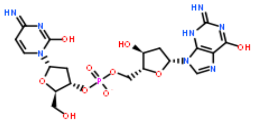 | 0 | 3002 | C19H25N8O10P | -8.457 | 62 | 12 | -14.111 | -4.6095 | -29.15 | 6.9913 | 34.756 | 33.366 | -133.021 | 1.94762 | 1 |
| 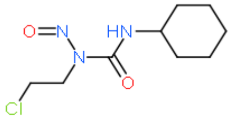 | 0 | 1076 | 1206 | -8.45 | 31 | 5 | -6.7114 | -4.6218 | -20.35 | 3.1594 | 14.82 | 24.188 | -87.27332 | 1.33854 | 1 |
| 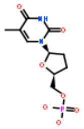 | 0 | 2832 | C10H15N2O7P | -8.438 | 33 | 4 | -10.73 | -3.2861 | -15.39 | 1.1299 | 21.905 | 25.452 | -91.09776 | 1.28681 | 1 |
| 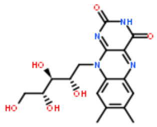 | 0 | 36 | C17H20N4O6 | -8.432 | 47 | 9 | -8.3025 | -3.5239 | -25.13 | 5.2455 | 23.347 | 21.224 | -77.809 | 1.49168 | 1 |
| 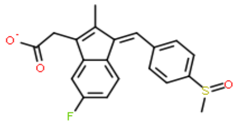 | 0 | 492 | C20H17FO3S | -8.432 | 41 | 3 | -4.4678 | -4.3507 | -21.38 | 1.9962 | 16.512 | 17.864 | -63.4879 | 1.50292 | 1 |
| 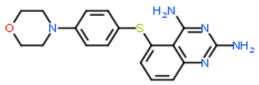 | 0 | 1764 | TQ6 | -8.414 | 44 | 0 | -6.0329 | -5.6141 | -16.35 | 3.3555 | 22.266 | 12.499 | -65.08901 | 1.46942 | 1 |
| 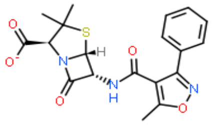 | 0 | 597 | 713 | -8.41 | 46 | 3 | -8.4853 | -5.1125 | -24.79 | 13.868 | 18.876 | 25.755 | -132.7692 | 1.58156 | 1 |
| 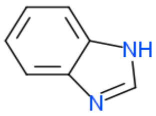 | 0 | 2658 | C7H6N2 | -8.389 | 15 | 0 | -3.2934 | -2.6085 | -11.16 | 0.3087 | 7.4045 | 10.783 | -57.74675 | 0.51788 | 1 |
| 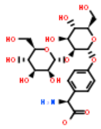 | 0 | 2394 | C20H29NO13 | -8.364 | 62 | 15 | -10.066 | -4.1537 | -19.93 | 3.0075 | 29.407 | 10.545 | -113.326 | 2.12389 | 1 |

| | | | | | | | | | | | | | | | |
|---|---|---|---|---|---|---|---|---|---|---|---|---|---|---|---|
| 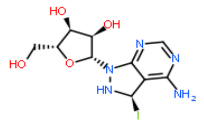 | 0 | 2152 | C10H14IN5O4 | -8.363 | 34 | 5 | -6.3301 | -3.9166 | -19.17 | 0 | 20.229 | 17.083 | -86.30513 | 1.11896 | 1 |
| 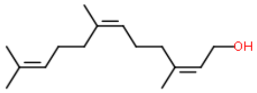 | 0 | 2239 | FOH | -8.354 | 42 | 8 | -4.376 | -5.5139 | -22.09 | 3.7286 | 14.01 | 16.6 | -89.7278 | 1.36049 | 1 |
| 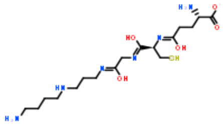 | 0 | 2972 | C17H34N6O5S | -8.347 | 62 | 23 | -14.49 | -5.3436 | -33.08 | 21.404 | 30.286 | 23.656 | -110.0571 | 2.05756 | 1 |
| 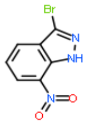 | 0 | 1761 | C7H4BrN3O2 | -8.302 | 17 | 1 | -3.1772 | -2.7834 | -13.71 | 0.8906 | 8.8591 | 11.419 | -44.60983 | 0.81093 | 1 |
| 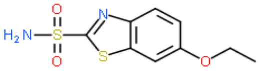 | 0 | 205 | 311 | -8.28 | 26 | 3 | -4.0883 | -4.0487 | -20.28 | 2.6463 | 15.08 | 15.693 | -94.7639 | 1.2573 | 1 |
| 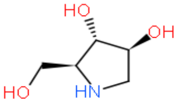 | 0 | 3079 | C5H11NO3 | -8.26 | 20 | 4 | -8.2461 | -2.2331 | -12.37 | 4.7979 | 16.864 | 12.937 | -62.84405 | 0.59868 | 1 |
| 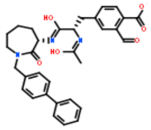 | 0 | 2947 | C32H33N3O6 | -8.257 | 73 | 11 | -4.5554 | -7.7733 | -36.73 | 4.9911 | 23.93 | 26.763 | -179.3257 | 2.27797 | 1 |
| 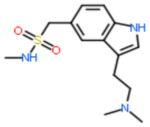 | 0 | 555 | 669 | -8.255 | 41 | 6 | -2.5264 | -3.5926 | -21.18 | 3.1894 | 15.586 | 6.8535 | -29.27943 | 1.28299 | 1 |
| 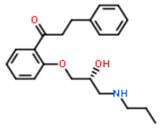 | 0 | 1052 | C21H27NO3 | -8.24 | 52 | 10 | -6.9885 | -6.9151 | -20.83 | 0.6383 | 17.192 | 23.679 | -120.1774 | 1.82405 | 1 |
| 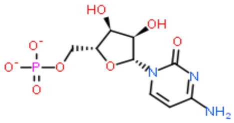 | 0 | 3072 | Cytidine | -8.231 | 33 | 6 | -11.614 | -2.2495 | -20.7 | 2.7798 | 25.491 | 28.748 | -84.91771 | 1.26197 | 1 |
| 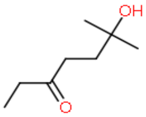 | 0 | 3354 | C8H16O2 | -8.227 | 26 | 5 | -4.4929 | -3.6618 | -13.91 | 0.946 | 11.772 | 10.774 | -69.51648 | 0.88599 | 1 |

| | | | | | | | | | | | | | | | |
|---|---|---|---|---|---|---|---|---|---|---|---|---|---|---|---|
| 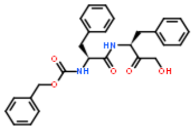 | 0 | 1644 | T10 | -8.221 | 62 | 13 | -7.1728 | -7.2077 | -36.86 | 17.63 | 21.07 | 26.419 | -180.3871 | 1.93695 | 1 |
| 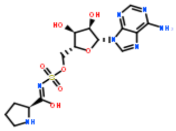 | 0 | 2240 | C15H21N7O7S | -8.192 | 51 | 9 | -10.779 | -4.6925 | -24.85 | 4.2419 | 30.445 | 24.694 | -128.1562 | 1.90122 | 1 |
| 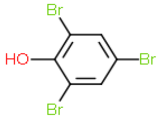 | 0 | 2153 | C6H3Br3O | -8.173 | 13 | 0 | -0.7441 | -3.4189 | -16.65 | 0.4743 | 8.6193 | 8.9463 | -22.13431 | 0.68336 | 1 |
| 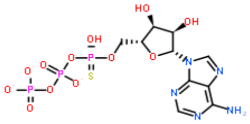 | 0 | 2094 | C10H16N5O12P3S | -8.172 | 44 | 11 | -14.433 | -3.5216 | -30.98 | 0 | 35.816 | 42.75 | -146.9992 | 1.69348 | 1 |
| 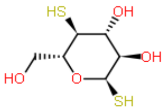 | 0 | 1643 | C6H12O4S2 | -8.164 | 24 | 6 | -6.5541 | -1.212 | -13.98 | 3.7474 | 14.088 | 10.296 | -45.19841 | 0.70876 | 1 |
| 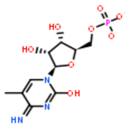 | 0 | 1759 | C10H16N3O8P | -8.15 | 36 | 7 | -10.407 | -2.9075 | -16.9 | 4.6701 | 26.492 | 15.391 | -86.06086 | 1.33272 | 1 |
| 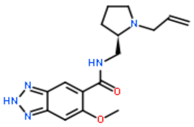 | 0 | 1232 | 1425 | -8.14 | 44 | 4 | -4.4772 | -4.7467 | -23.3 | 5.4722 | 15.201 | 19.063 | -93.29194 | 1.26973 | 1 |
| 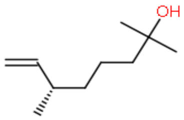 | 0 | 2017 | C10H20O | -8.138 | 31 | 6 | -4.1291 | -4.5909 | -14.23 | 1.0014 | 11.371 | 10.433 | -61.91482 | 1.036 | 1 |
| 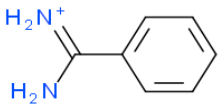 | 0 | 2811 | C7H9N2 | -8.136 | 18 | 1 | -3.4944 | -1.9146 | -13.29 | 0.2497 | 8.9431 | 11.851 | -16.95644 | 0.44734 | 1 |
| 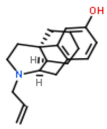 | 0 | 394 | m | -8.116 | 46 | 2 | -3.4221 | -5.0539 | -19.84 | 2.4321 | 12.789 | 17.452 | -92.88983 | 0.92041 | 1 |
| 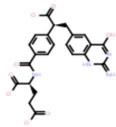 | 0 | 2268 | C23H22N4O8 | -8.113 | 54 | 10 | -11.161 | -4.554 | -31.02 | 17.266 | 30.357 | 22.835 | -145.3759 | 1.80206 | 1 |

| | | | | | | | | | | | | | | | | |
|---|---|---|---|---|---|---|---|---|---|---|---|---|---|---|---|---|
| 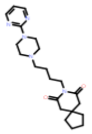 | 0 | 380 | 490 | -8.105 | 59 | 5 | -6.0255 | -7.8003 | -25 | 4.9881 | 19.633 | 25.221 | -121.9548 | 1.84051 | | 1 |
| 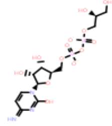 | 0 | 2215 | C12H21N3O13P2 | -8.102 | 49 | 15 | -16.98 | -2.5783 | -20.58 | 1.9101 | 32.205 | 34.642 | -122.5455 | 1.85402 | | 1 |
| 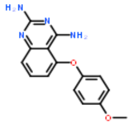 | 0 | 2138 | MQ1 | -8.073 | 35 | 2 | -5.5896 | -5.9139 | -19.7 | 1.7812 | 19.956 | 18.926 | -102.2993 | 1.5329 | | 1 |
| 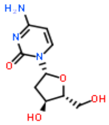 | 0 | 2319 | C9H13N3O4 | -8.071 | 29 | 4 | -5.0662 | -2.0068 | -16.13 | 2.6299 | 18.312 | 6.8274 | -18.89286 | 1.01159 | | 1 |
| 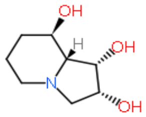 | 0 | 1795 | C8H15NO3 | -8.052 | 27 | 3 | -5.6671 | -2.6817 | -10.59 | 1.9308 | 13.088 | 8.9359 | -42.48705 | 0.73445 | | 1 |
| 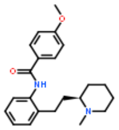 | 0 | 1097 | C22H28N2O2 | -8.046 | 54 | 3 | -3.2403 | -7.031 | -26.01 | 5.1545 | 18.488 | 18.361 | -131.9782 | 1.98911 | | 1 |
| 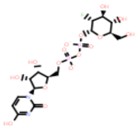 | 0 | 3152 | C15H23FN2O16P2 | -8.039 | 57 | 15 | -16.993 | -3.4917 | -30.02 | 6.0306 | 33.007 | 45.358 | -163.6793 | 2.22308 | | 1 |
| 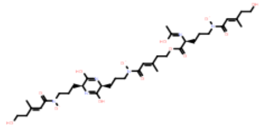 | 0 | 1530 | C35H53FeN6O13 | -8.032 | 107 | 29 | -12.751 | -6.7415 | -39.62 | 7.1803 | 35.05 | 30.856 | -134.357 | 3.32296 | | 1 |
| 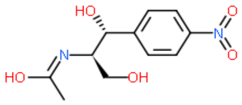 | 0 | 2332 | C11H14N2O5 | -8.025 | 32 | 8 | -7.9965 | -2.7667 | -17.6 | 7.3853 | 15.972 | 15.828 | -58.54892 | 1.00021 | | 1 |
| 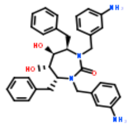 | 0 | 1860 | C33H36N4O3 | -8.021 | 76 | 10 | -6.6074 | -7.9528 | -32.61 | 8.4528 | 23.414 | 27.28 | -159.6749 | 1.73836 | | 1 |
| 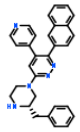 | 0 | 1752 | C30H27N5 | -8.018 | 62 | 2 | -1.8953 | -7.8837 | -27.72 | 3.6075 | 18.313 | 19.208 | -91.53625 | 2.07865 | | 1 |

| | | | | | | | | | | | | | | | |
|---|---|---|---|---|---|---|---|---|---|---|---|---|---|---|---|
| 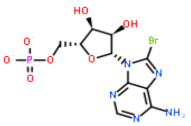 | 0 | 3024 | C10H13BrN5O7P | -8.014 | 35 | 6 | -10.278 | -3.9393 | -19.84 | 0 | 28.986 | 22.913 | -99.48419 | 1.34745 | 1 |
| 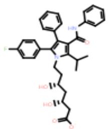 | 0 | 948 | C33H35FN2O5 | -7.997 | 75 | 13 | -6.0924 | -8.1381 | -29.95 | 1.6685 | 24.542 | 23.657 | -181.3356 | 2.5997 | 1 |
| 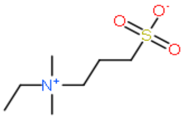 | 0 | 2341 | C7H17NO3S | -7.997 | 29 | 5 | -3.2826 | -3.0186 | -14.15 | 0.7393 | 11.735 | 6.4246 | -22.87403 | 0.81979 | 1 |
| 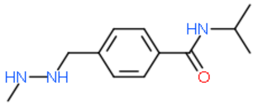 | 0 | 1038 | 1168 | -7.989 | 35 | 4 | -4.5236 | -5.2374 | -17.55 | 1.4229 | 13.888 | 16.424 | -50.21686 | 1.20556 | 1 |
| 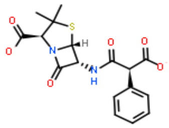 | 0 | 465 | 578 | -7.981 | 42 | 5 | -9.4438 | -4.6732 | -25.17 | 3.0065 | 22.198 | 34.478 | -136.9605 | 1.64096 | 1 |
| 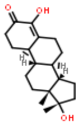 | 0 | 1336 | C19H28O3 | -7.978 | 50 | 1 | -4.0478 | -4.1112 | -17.44 | 0.7562 | 12.416 | 18.325 | -63.48873 | 1.11636 | 1 |
| 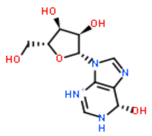 | 0 | 2709 | C10H15N4O5 | -7.954 | 34 | 6 | -9.1117 | -2.9625 | -12.25 | 0 | 21.026 | 15.089 | -89.47023 | 1.20368 | 1 |
| 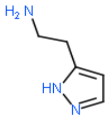 | 0 | 167 | 272 | -7.947 | 17 | 3 | -4.8549 | -1.8174 | -13.75 | 0.4948 | 10.917 | 13.664 | -29.58691 | 0.57656 | 1 |
| 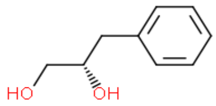 | 0 | 2508 | C9H12O2 | -7.942 | 23 | 5 | -4.1684 | -3.6058 | -15.51 | 2.113 | 11.61 | 11.441 | -66.6591 | 0.78733 | 1 |
| 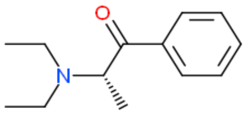 | 0 | 815 | 937 | -7.896 | 34 | 5 | -3.0454 | -5.0859 | -18.53 | 0.5056 | 11.563 | 14.802 | -95.85492 | 1.25618 | 1 |
| 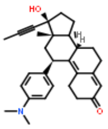 | 0 | 715 | C29H35NO2 | -7.886 | 67 | 2 | -2.6595 | -7.0976 | -25.91 | 3.0754 | 15.646 | 22.318 | -89.98434 | 1.72434 | 1 |

| | | | | | | | | | | | | | | | |
|---|---|---|---|---|---|---|---|---|---|---|---|---|---|---|---|
| 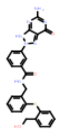 | 0 | 2912 | C25H21N7O3S | -7.841 | 57 | 4 | -7.4992 | -5.3679 | -32.29 | 6.1111 | 31.047 | 26.768 | -137.6719 | 1.94836 | 1 |
| 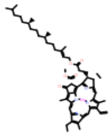 | 0 | 1890 | m | -7.825 | 137 | 19 | -3.8225 | -10.932 | -31.92 | 0 | 25.809 | 15.654 | -172.1072 | 3.1116 | 1 |
| 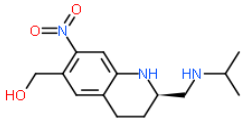 | 0 | 968 | 1096 | -7.812 | 41 | 6 | -2.612 | -4.4981 | -22.77 | 3.128 | 12.683 | 14.531 | -100.7064 | 1.23464 | 1 |
| 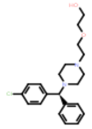 | 0 | 444 | 557 | -7.805 | 53 | 9 | -2.5415 | -6.5878 | -24.13 | 2.3962 | 15.036 | 13.716 | -117.081 | 1.65274 | 1 |
| 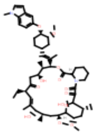 | 0 | 3271 | C52H76N2O13 | -7.796 | 143 | 10 | -4.3017 | -11.164 | -35.64 | 7.2814 | 28.539 | 21.875 | -220.9923 | 2.64532 | 1 |
| 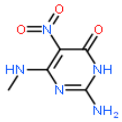 | 0 | 3348 | C5H7N5O3 | -7.794 | 20 | 1 | -4.4321 | -1.4826 | -15.25 | 0.888 | 10.805 | 15.796 | -26.60033 | 0.69954 | 1 |
| 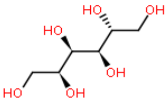 | 0 | 1430 | C6H14O6 | -7.79 | 26 | 11 | -10.075 | -2.1372 | -13.03 | 6.3266 | 21.054 | 9.0472 | -92.26641 | 0.97371 | 1 |
| 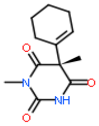 | 0 | 1183 | [NO | -7.789 | 33 | 1 | -4.1341 | -3.7066 | -13.91 | 0.1991 | 12.671 | 13.4 | -85.9606 | 1.14196 | 1 |
| 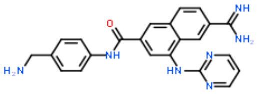 | 0 | 2770 | C23H21N7O | -7.763 | 52 | 2 | -2.8466 | -5.71 | -27.96 | 0 | 22.245 | 20.212 | -131.0283 | 1.9716 | 1 |
| 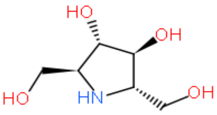 | 0 | 1925 | C6H13NO4 | -7.758 | 24 | 6 | -6.0364 | -2.5292 | -9.576 | 1.1621 | 13.317 | 6.8179 | -68.90038 | 0.8324 | 1 |
| 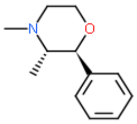 | 0 | 1375 | C12H17NO | -7.757 | 31 | 1 | -2.4385 | -4.037 | -12.58 | 0.8772 | 10.773 | 6.7993 | -23.52161 | 0.88026 | 1 |

| | | | | | | | | | | | | | | | |
|---|---|---|---|---|---|---|---|---|---|---|---|---|---|---|---|
| 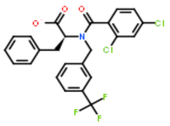 | 0 | 3256 | C24H18Cl2F3NO3 | -7.752 | 50 | 7 | -1.4533 | -5.7549 | -28.98 | 7.4172 | 14.394 | 14.112 | -98.76459 | 1.31387 | 1 |
| 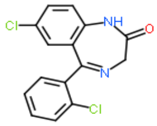 | 0 | 1309 | 1511 | -7.735 | 30 | 0 | -4.8524 | -4.8008 | -17.25 | 1.2573 | 14.842 | 19.886 | -99.008 | 1.22694 | 1 |
| 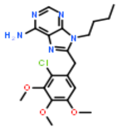 | 0 | 3167 | PU8 | -7.705 | 52 | 5 | -4.4636 | -7.1433 | -30.28 | 5.9874 | 24.44 | 20.812 | -130.176 | 1.53513 | 1 |
| 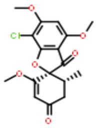 | 0 | 291 | 400 | -7.702 | 41 | 0 | -6.9541 | -5.8563 | -11.91 | 7.1992 | 17.075 | 13.028 | -98.02365 | 1.4418 | 1 |
| 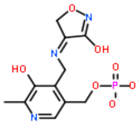 | 0 | 3424 | C11H14N3O7P | -7.702 | 34 | 6 | -9.9516 | -3.7201 | -16.23 | 1.4085 | 24.294 | 20.5 | -91.01505 | 1.4789 | 1 |
| 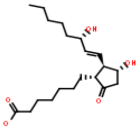 | 0 | 653 | C20H34O5 | -7.702 | 58 | 15 | -12.015 | -5.4743 | -23.6 | 10.238 | 25.097 | 24.244 | -130.8282 | 1.64232 | 1 |
| 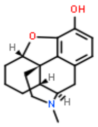 | 0 | 1327 | C17H21NO2 | -7.69 | 41 | 0 | -3.0952 | -4.3826 | -16.77 | 0.308 | 10.728 | 17.596 | -88.3738 | 1.0784 | 1 |
| 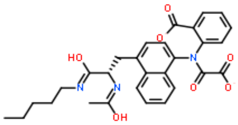 | 0 | 1597 | C29H31N3O7 | -7.685 | 68 | 13 | -14.381 | -5.1726 | -29.79 | 17.474 | 26.085 | 36.53 | -177.2653 | 1.94424 | 1 |
| 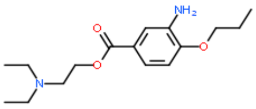 | 0 | 688 | 807 | -7.684 | 47 | 7 | -4.8207 | -6.5096 | -24.29 | 1.9387 | 17.953 | 21.716 | -95.28025 | 1.87561 | 1 |
| 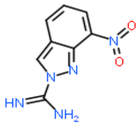 | 0 | 2754 | 7I2 | -7.68 | 22 | 2 | -2.793 | -2.3711 | -18.54 | 0.0652 | 14.54 | 11.17 | -81.29057 | 0.97035 | 1 |
| 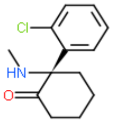 | 0 | 1091 | 1221 | -7.676 | 32 | 2 | -4.3554 | -4.6199 | -11.8 | 3.0165 | 11.81 | 9.6608 | -77.84809 | 1.02145 | 1 |

| | | | | | | | | | | | | | | | |
|---|---|---|---|---|---|---|---|---|---|---|---|---|---|---|---|
| 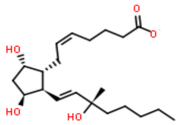 | 0 | 319 | C25H47NO8 | -7.671 | 61 | 15 | -12.336 | -5.6055 | -22.01 | 13.6 | 23.134 | 22.417 | -113.0621 | 1.54569 | 1 |
| 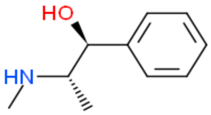 | 0 | 732 | 852 | -7.663 | 27 | 4 | -5.0705 | -4.1456 | -14.86 | 2.5359 | 13.879 | 12.833 | -76.06832 | 1.00905 | 1 |
| 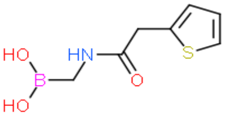 | 0 | 1852 | CTB | -7.646 | 23 | 6 | -3.2774 | -2.0261 | -14.93 | 0 | 13.289 | 5.1001 | -12.95472 | 0.73591 | 1 |
| 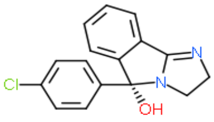 | 0 | 466 | 579 | -7.645 | 33 | 2 | -1.5449 | -5.0999 | -18.73 | 1.8352 | 13.097 | 9.5215 | -79.2544 | 1.00751 | 1 |
| 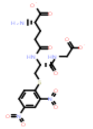 | 0 | 2191 | C16H19N5O10S | -7.642 | 49 | 13 | -7.9125 | -3.7385 | -25.99 | 10.623 | 23.831 | 13.377 | -111.3574 | 1.77362 | 1 |
| 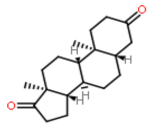 | 0 | 1357 | 1561 | -7.64 | 49 | 0 | -1.831 | -4.6368 | -19.85 | 1.3646 | 11.176 | 16.287 | -95.32983 | 1.16647 | 1 |
| 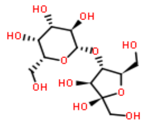 | 0 | 468 | 581 | -7.6 | 45 | 13 | -12.506 | -3.4467 | -12.13 | 3.8995 | 23.61 | 16.11 | -127.6855 | 1.55152 | 1 |
| 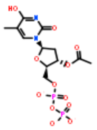 | 0 | 2277 | C12H18N2O12P2 | -7.588 | 43 | 8 | -16.042 | -3.6576 | -22.28 | 10.481 | 28.148 | 38.679 | -121.3567 | 1.54411 | 1 |
| 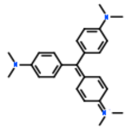 | 0 | 297 | 3468 | -7.575 | 58 | 0 | 0 | -7.8093 | -25.47 | 3.7413 | 16.072 | 13.38 | -78.14441 | 2.03108 | 1 |
| 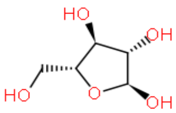 | 0 | 1701 | C5H10O5 | -7.561 | 20 | 5 | -6.5307 | -1.5688 | -11.71 | 0 | 16.8 | 9.2718 | -49.7782 | 0.8784 | 1 |
| 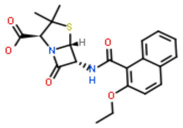 | 0 | 494 | 607 | -7.556 | 50 | 3 | -8.0216 | -6.0998 | -19.71 | 6.6363 | 19.128 | 24.86 | -156.3561 | 1.6626 | 1 |

| | | | | | | | | | | | | | | | |
|---|---|---|---|---|---|---|---|---|---|---|---|---|---|---|---|
| 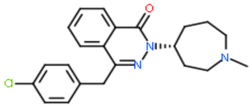 | 0 | 849 | 972 | -7.549 | 51 | 3 | -2.8165 | -5.8635 | -26.79 | 4.203 | 14.22 | 23.187 | -91.81674 | 1.34887 | 1 |
| 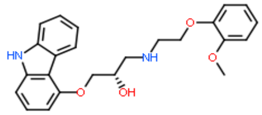 | 0 | 1007 | C24H26N2O4 | -7.536 | 56 | 8 | -4.7783 | -6.3722 | -27.51 | 11.546 | 17.073 | 17.874 | -114.3116 | 1.60004 | 1 |
| 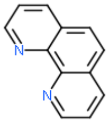 | 0 | 2104 | PHN | -7.532 | 22 | 0 | -4.1548 | -4.463 | -15.21 | 0.8068 | 12.162 | 17.538 | -91.44948 | 1.13021 | 1 |
| 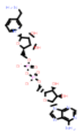 | 0 | 3434 | C21H29N7O13P2 | -7.531 | 71 | 16 | -12.228 | -5.0836 | -33.96 | 8.0074 | 39.698 | 25.14 | -133.3371 | 2.57389 | 1 |
| 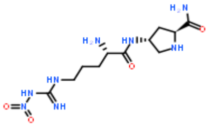 | 0 | 1835 | C11H22N8O4 | -7.526 | 45 | 11 | -10.628 | -3.4262 | -23.5 | 7.5892 | 26.478 | 21.439 | -78.56869 | 1.43301 | 1 |
| 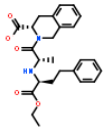 | 0 | 761 | 881 | -7.525 | 61 | 10 | -8.3643 | -6.9509 | -25.73 | 9.1853 | 21.231 | 25.141 | -150.473 | 1.98936 | 1 |
| 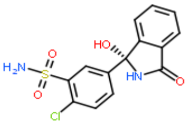 | 0 | 204 | C14H11ClN2O4S | -7.508 | 33 | 4 | -4.9107 | -3.5031 | -23.94 | 1.1732 | 20.291 | 19.519 | -117.5216 | 1.33856 | 1 |
| 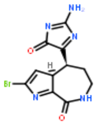 | 0 | 2646 | HMD | -7.506 | 29 | 1 | -3.2877 | -3.0231 | -20.51 | 0.5931 | 15.915 | 15.841 | -61.26037 | 0.93335 | 1 |
| 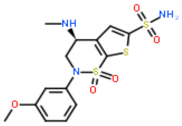 | 0 | 2902 | C14H17N3O5S3 | -7.502 | 42 | 4 | -3.2194 | -5.0647 | -26.83 | 4.3418 | 18.981 | 17.622 | -116.3087 | 1.8708 | 1 |
| 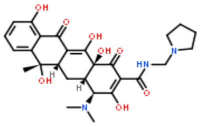 | 0 | 1152 | m | -7.49 | 71 | 5 | -3.0438 | -6.712 | -23.79 | 0 | 23.48 | 11.956 | -119.7346 | 1.75489 | 1 |
| 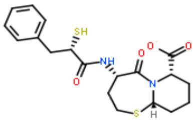 | 0 | 766 | C19H24N2O4S2 | -7.489 | 50 | 6 | -7.7786 | -5.3104 | -27.4 | 9.5296 | 20.701 | 27.739 | -116.3622 | 1.71959 | 1 |

| | | | | | | | | | | | | | | |
|---|---|---|---|---|---|---|---|---|---|---|---|---|---|---|
| 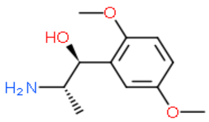 | 0 | 607 | 723 | -7.488 | 32 | 4 | -5.9826 | -4.2213 | -14.58 | 4.9789 | 15.746 | 11.91 | -80.33441 | 1.04624 | 1 |
| 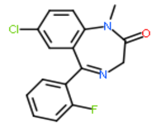 | 0 | 1363 | 1567 | -7.48 | 33 | 0 | -1.9636 | -5.1572 | -22.54 | 1.0893 | 13.27 | 19.518 | -70.95442 | 1.09225 | 1 |
| 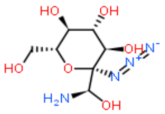 | 0 | 2963 | C7H14N4O6 | -7.471 | 31 | 9 | -8.531 | -1.4467 | -16.65 | 0.7157 | 23.455 | 12.595 | -37.65799 | 1.00397 | 1 |
| 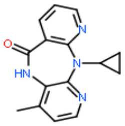 | 0 | 133 | 238 | -7.469 | 34 | 1 | -2.3278 | -4.396 | -19.43 | 1.1913 | 11.468 | 16.491 | -47.36518 | 0.82332 | 1 |
| 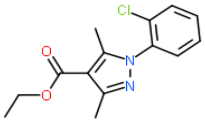 | 0 | 3442 | 6DE | -7.468 | 34 | 1 | -1.887 | -5.6865 | -21.37 | 1.2488 | 14.128 | 15.967 | -106.1811 | 1.64135 | 1 |
| 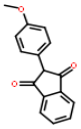 | 0 | 996 | 1125 | -7.452 | 31 | 1 | -2.5334 | -4.6044 | -23.31 | 2.1806 | 13.751 | 19.894 | -100.1521 | 1.25106 | 1 |
| 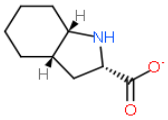 | 0 | 3077 | C9H15NO2 | -7.439 | 26 | 1 | -6.4322 | -3.1298 | -8.087 | 2.4578 | 12.849 | 11.07 | -69.02258 | 0.8447 | 1 |
| 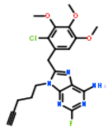 | 0 | 2278 | PU1 | -7.439 | 51 | 5 | -3.6976 | -7.4517 | -28.99 | 4.976 | 22.227 | 20.063 | -125.8459 | 1.58715 | 1 |
| 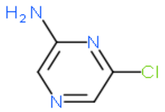 | 0 | 2039 | C4H4ClN3 | -7.428 | 12 | 0 | -3.5642 | -2.483 | -10.32 | 0.3674 | 9.2183 | 9.757 | -48.49601 | 0.39405 | 1 |
| 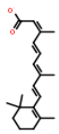 | 0 | 859 | C20H28O2 | -7.424 | 49 | 1 | -1.797 | -6.0607 | -23.36 | 0 | 16.106 | 17.977 | -70.75812 | 1.75524 | 1 |
| 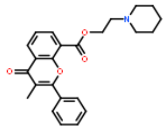 | 0 | 1019 | 1148 | -7.414 | 54 | 4 | -2.6399 | -6.0149 | -24.46 | 4.6465 | 16.387 | 15.565 | -133.0006 | 2.07921 | 1 |

| | | | | | | | | | | | | | | | |
|---|---|---|---|---|---|---|---|---|---|---|---|---|---|---|---|
| 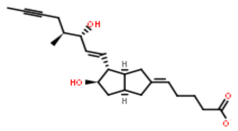 | 0 | 960 | C22H32O4 | -7.406 | 57 | 10 | -8.5886 | -6.7772 | -23.95 | 9.3826 | 20.41 | 24.052 | -113.3363 | 1.87799 | 1 |
| 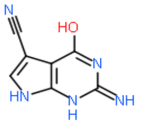 | 0 | 2763 | Structure | -7.402 | 18 | 1 | -6.1906 | -1.3215 | -15.54 | 3.9556 | 15.98 | 14.732 | -37.10443 | 0.64366 | 1 |
| 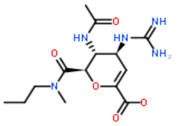 | 0 | 3166 | C14H23N5O5 | -7.393 | 46 | 8 | -7.4125 | -4.053 | -19.9 | 3.4117 | 21.68 | 16.474 | -89.65616 | 1.52751 | 1 |
| 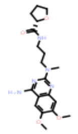 | 0 | 239 | C19H27N5O4 | -7.382 | 55 | 5 | -6.2149 | -7.2424 | -24.2 | 7.8901 | 20.066 | 22.14 | -124.601 | 1.66033 | 1 |
| 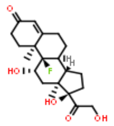 | 0 | 573 | C21H29FO5 | -7.38 | 56 | 5 | -5.1819 | -4.2832 | -18.89 | 3.5292 | 17.329 | 14.21 | -104.1346 | 1.21596 | 1 |
| 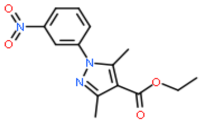 | 0 | 1723 | 7DE | -7.366 | 36 | 2 | -1.4842 | -5.1209 | -23.54 | 2.6383 | 13.1 | 16.291 | -103.8212 | 1.67855 | 1 |
| 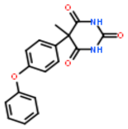 | 0 | 3042 | HQQ | -7.364 | 37 | 3 | -4.8717 | -4.8494 | -20.96 | 2.3895 | 19.309 | 17.35 | -125.8003 | 1.71689 | 1 |
| 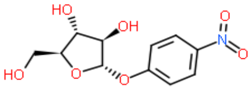 | 0 | 2877 | KHP | -7.359 | 32 | 7 | -7.0701 | -3.4386 | -16.59 | 2.8428 | 19.771 | 13.161 | -83.78372 | 1.21796 | 1 |
| 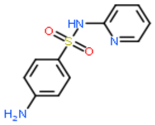 | 0 | 771 | 891 | -7.358 | 28 | 3 | -1.4057 | -3.985 | -20.81 | 1.7318 | 13.081 | 10.644 | -74.44111 | 1.11528 | 1 |
| 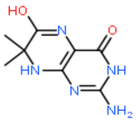 | 0 | 2022 | ROI | -7.352 | 26 | 1 | -8.6571 | -1.8782 | -13.89 | 0.0792 | 21.345 | 19.757 | -71.50089 | 1.20129 | 1 |
| 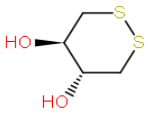 | 0 | 1599 | C4H8O2S2 | -7.333 | 16 | 2 | -4.9845 | -2.2027 | -10.31 | 0 | 12.724 | 9.5798 | -38.86559 | 0.57746 | 1 |

| | | | | | | | | | | | | | | | |
|---|---|---|---|---|---|---|---|---|---|---|---|---|---|---|---|
| 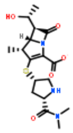 | 0 | 643 | C17H25N3O5S | -7.323 | 50 | 6 | -11.828 | -4.9892 | -20.97 | 3.7517 | 25.369 | 33.384 | -127.0964 | 2.13072 | 1 |
| 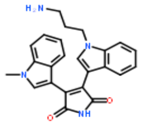 | 0 | 1710 | BI8 | -7.318 | 52 | 4 | -4.355 | -5.2041 | -26.77 | 6.0721 | 19.004 | 20.694 | -109.1386 | 1.44426 | 1 |
| 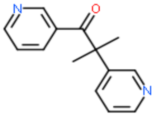 | 0 | 887 | C14H14N2O | -7.318 | 31 | 2 | -6.3561 | -4.7902 | -18.28 | 6.2177 | 15.632 | 20.602 | -110.7343 | 1.22478 | 1 |
| 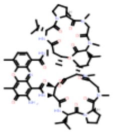 | 0 | 847 | C62H86N12O16 | -7.316 | 176 | 6 | -7.5082 | -12.604 | -49.95 | 26.895 | 43.201 | 27.173 | -280.7732 | 4.02915 | 1 |
| 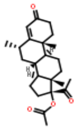 | 0 | 490 | C24H34O4 | -7.308 | 62 | 2 | -3.7974 | -5.2703 | -19.41 | 4.1228 | 16.561 | 13.858 | -90.51878 | 1.80477 | 1 |
| 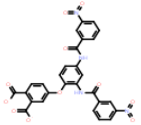 | 0 | 2756 | C28H18N4O11 | -7.305 | 59 | 6 | -6.2922 | -5.8677 | -32.66 | 8.465 | 28.877 | 22.534 | -150.7599 | 2.35055 | 1 |
| 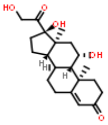 | 0 | 624 | C21H30O5 | -7.276 | 56 | 5 | -5.0746 | -4.3595 | -19.53 | 4.1437 | 17.562 | 14.177 | -101.0119 | 1.22815 | 1 |
| 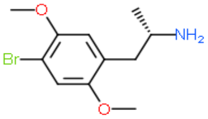 | 0 | 1287 | 1484 | -7.267 | 31 | 3 | -3.862 | -4.6705 | -17.01 | 0 | 13.917 | 15.767 | -97.9524 | 1.13799 | 1 |
| 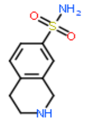 | 0 | 3132 | SKF | -7.26 | 26 | 2 | -2.6816 | -3.2855 | -15.05 | 0 | 12.957 | 8.8784 | -64.17279 | 1.07037 | 1 |
| 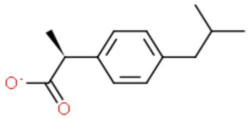 | 0 | 923 | 1050 | -7.255 | 32 | 4 | -4.9037 | -4.6025 | -17.02 | 1.3926 | 13.604 | 17.911 | -122.0043 | 1.21801 | 1 |
| 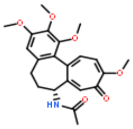 | 0 | 1205 | C22H25NO6 | -7.252 | 54 | 1 | -5.9291 | -6.0289 | -22.49 | 5.6407 | 20.26 | 22.883 | -75.35109 | 1.40687 | 1 |

| | | | | | | | | | | | | | | | |
|---|---|---|---|---|---|---|---|---|---|---|---|---|---|---|---|
| 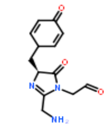 | 0 | 2019 | C13H15N3O3 | -7.245 | 34 | 6 | -6.3609 | -3.3548 | -23.58 | 3.2397 | 19.806 | 21.638 | -91.32706 | 1.22922 | 1 |
| 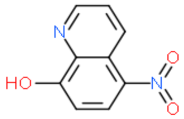 | 0 | 1229 | 1422 | -7.234 | 20 | 1 | 0 | -3.0463 | -17.91 | 0.8148 | 7.7523 | 8.802 | -67.91411 | 0.8666 | 1 |
| 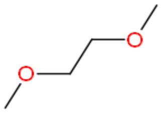 | 0 | 1532 | C4H10O2 | -7.224 | 16 | 3 | -2.5545 | -2.4379 | -8.042 | 0 | 8.9243 | 0.6374 | -2.607001 | 0.52998 | 1 |
| 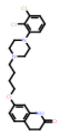 | 0 | 1107 | 1238 | -7.217 | 57 | 5 | -4.2844 | -6.7582 | -29.75 | 8.7745 | 19.804 | 22.403 | -133.7761 | 1.9664 | 1 |
| 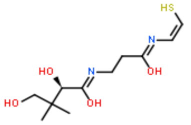 | 0 | 3379 | C11H20N2O4S | -7.202 | 38 | 11 | -8.9785 | -4.3127 | -19.27 | 1.4301 | 18.344 | 24.94 | -98.53204 | 1.53024 | 1 |
| 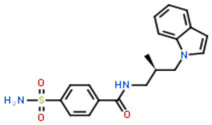 | 0 | 2210 | C19H21N3O3S | -7.201 | 47 | 6 | -2.484 | -5.3239 | -29.67 | 4.7511 | 16.01 | 21 | -123.8762 | 1.5243 | 1 |
| 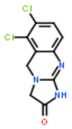 | 0 | 156 | C10H7Cl2N3O | -7.198 | 23 | 0 | -2.7837 | -3.6603 | -16.75 | 0.7972 | 14.573 | 11.581 | -89.80601 | 1.23168 | 1 |
| 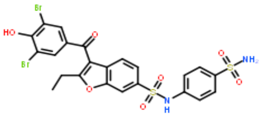 | 0 | 2003 | C23H18Br2N2O7S2 | -7.187 | 54 | 6 | -4.1177 | -5.2885 | -30.83 | 9.5948 | 23.171 | 16.473 | -98.60558 | 2.13773 | 1 |
| 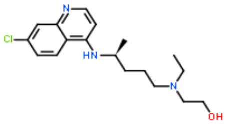 | 0 | 1404 | 1611 | -7.17 | 49 | 9 | -5.6866 | -6.919 | -23.71 | 6.6831 | 16.942 | 20.376 | -119.9281 | 1.39036 | 1 |
| 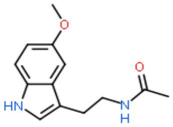 | 0 | 937 | C13H16N2O2 | -7.161 | 33 | 3 | -3.47 | -4.636 | -17.02 | 2.0588 | 12.397 | 14.267 | -91.23522 | 1.16541 | 1 |
| 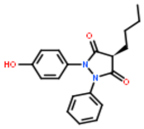 | 0 | 3237 | 3585 | -7.148 | 44 | 4 | -4.7713 | -5.4973 | -22.6 | 3.4515 | 17.528 | 20.591 | -109.0223 | 1.68954 | 1 |

| | | | | | | | | | | | | | | |
|---|---|---|---|---|---|---|---|---|---|---|---|---|---|---|
| 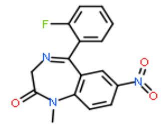 | 0 | 1340 | 1544 | -7.131 | 35 | 1 | -0.6049 | -4.8784 | -22.46 | 1.7406 | 10.666 | 15.885 | -110.8874 | 1.47081 | 1 |
| 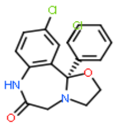 | 0 | 1349 | 1553 | -7.116 | 37 | 1 | -2.7409 | -4.9705 | -20.27 | 2.0424 | 13.426 | 17.453 | -76.47147 | 1.04185 | 1 |
| 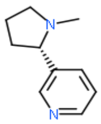 | 0 | 79 | C10H14N2 | -7.112 | 26 | 1 | -1.2093 | -3.7795 | -14.72 | 0.4168 | 8.0884 | 9.5286 | -33.17678 | 0.69705 | 1 |
| 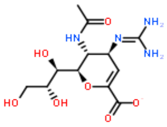 | 0 | 445 | 558 | -7.111 | 42 | 9 | -11.848 | -2.8172 | -18.73 | 1.32 | 27.719 | 24.997 | -36.03628 | 1.04233 | 1 |
| 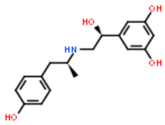 | 0 | 1143 | 1288 | -7.092 | 43 | 7 | -6.558 | -4.982 | -23.68 | 5.4516 | 20.812 | 20.36 | -90.054 | 1.38381 | 1 |
| 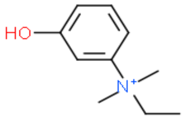 | 0 | 886 | 1010 | -7.09 | 28 | 2 | -3.5495 | -3.2847 | -13.01 | 0.6857 | 9.7142 | 12.435 | -55.0262 | 0.62668 | 1 |
| 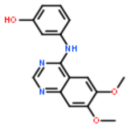 | 0 | 3039 | DTQ | -7.088 | 37 | 0 | -4.8003 | -5.4375 | -21.58 | 1.8583 | 22.035 | 19.147 | -100.3279 | 1.70879 | 1 |
| 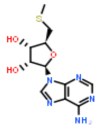 | 0 | 2026 | C11H15N5O3S | -7.087 | 35 | 5 | -9.4919 | -4.792 | -17.12 | 3.8404 | 25.871 | 19.02 | -99.2113 | 1.52121 | 1 |
| 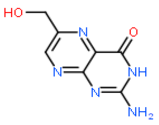 | 0 | 2878 | C7H7N5O2 | -7.07 | 21 | 2 | -8.093 | -2.0956 | -14.86 | 1.7051 | 20.255 | 18.567 | -72.5831 | 0.95646 | 1 |
| 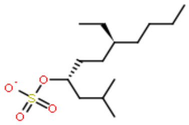 | 0 | 354 | C14H29NaO4S | -7.05 | 48 | 11 | -7.4225 | -5.5781 | -18.43 | 2.0545 | 19.046 | 17.844 | -130.7999 | 1.67154 | 1 |
| 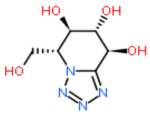 | 0 | 2204 | C6H10N4O4 | -7.047 | 24 | 5 | -7.6065 | -1.5375 | -11.01 | 1.7644 | 15.249 | 13.238 | -44.64549 | 0.83571 | 1 |

| | | | | | | | | | | | | | | | |
|---|---|---|---|---|---|---|---|---|---|---|---|---|---|---|---|
| 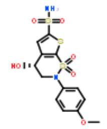 | 0 | 1728 | AL2 | -7.034 | 38 | 4 | -5.2878 | -3.569 | -18.28 | 2.6723 | 19.2 | 13.047 | -81.58495 | 1.62291 | 1 |
| 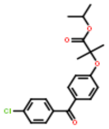 | 0 | 914 | 1039 | -7.027 | 46 | 4 | -2.9876 | -6.5029 | -27.45 | 3.9998 | 17.77 | 21.532 | -112.3922 | 1.80791 | 1 |
| 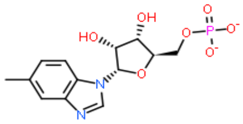 | 0 | 2523 | C13H17N2O7P | -7.017 | 38 | 6 | -10.603 | -4.6626 | -19.87 | 5.632 | 21.428 | 29.926 | -135.6818 | 1.33575 | 1 |
| 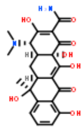 | 0 | 642 | C22H24N2O8 | -7.016 | 56 | 3 | -12.22 | -4.1263 | -22.93 | 8.0507 | 32.118 | 28.593 | -105.518 | 1.66788 | 1 |
| 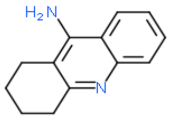 | 0 | 274 | 382 | -7.016 | 29 | 0 | -1.9621 | -3.7685 | -13.65 | 1.3285 | 11.533 | 7.0838 | -45.55718 | 0.92481 | 1 |
| 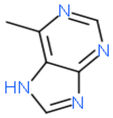 | 0 | 1871 | 5287547 | -7.001 | 16 | 0 | -3.2167 | -2.9021 | -12.33 | 0.538 | 10.012 | 11.489 | -52.69394 | 0.52925 | 1 |
| 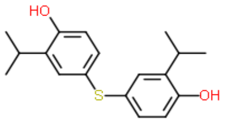 | 0 | 2582 | C18H22O2S | -7 | 43 | 2 | -5.3705 | -4.7608 | -16.69 | 3.9585 | 15.873 | 16.652 | -74.66812 | 1.12575 | 1 |
| 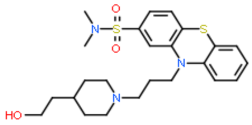 | 0 | 1413 | 1621 | -7 | 65 | 9 | -4.3191 | -7.029 | -31.74 | 5.6094 | 20.885 | 24.156 | -160.4035 | 2.23211 | 1 |
| 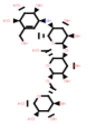 | 0 | 3158 | C25H43NO18 | -6.993 | 87 | 22 | -20.583 | -4.9983 | -29.66 | 21.497 | 41.694 | 31.395 | -161.6028 | 2.33647 | 1 |
| 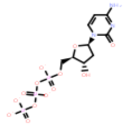 | 0 | 2939 | C9H16N3O13P3 | -6.992 | 40 | 9 | -10.809 | -2.8902 | -24.62 | 0 | 31.31 | 27.325 | -103.1461 | 1.45468 | 1 |
| 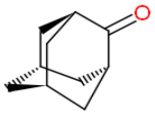 | 0 | 1882 | C10H14O | -6.989 | 25 | 0 | -0.9797 | -2.5628 | -11.22 | 0.1247 | 6.4547 | 5.3204 | -36.46539 | 0.55764 | 1 |

| | | | | | | | | | | | | | | | |
|---|---|---|---|---|---|---|---|---|---|---|---|---|---|---|---|
| 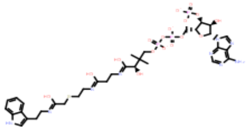 | 0 | 2629 | C33H48N9O17P3S | -6.986 | 107 | 29 | -20.516 | -6.7077 | -46.71 | 16.885 | 41.037 | 57.293 | -194.5614 | 3.3046 | 1 |
| 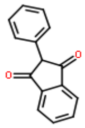 | 0 | 388 | 498 | -6.976 | 27 | 1 | -3.4249 | -4.5648 | -16.99 | 0.693 | 10.834 | 19.091 | -97.58793 | 1.23368 | 1 |
| 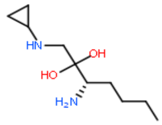 | 0 | 2144 | M1C | -6.974 | 36 | 10 | -8.3403 | -4.2338 | -19.64 | 6.554 | 18.285 | 19.627 | -77.08268 | 1.37579 | 1 |
| 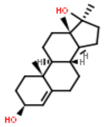 | 0 | 1277 | C20H32O2 | -6.969 | 54 | 2 | -4.4696 | -4.9099 | -15.36 | 1.5101 | 14.045 | 15.684 | -69.10432 | 1.08955 | 1 |
| 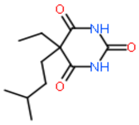 | 0 | 1179 | 1351 | -6.965 | 34 | 4 | -4.8822 | -3.4175 | -16.84 | 1.3084 | 15.626 | 14.569 | -97.41529 | 1.23683 | 1 |
| 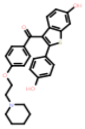 | 0 | 371 | 481 | -6.964 | 61 | 3 | -4.2236 | -7.5752 | -35.15 | 10.865 | 23.11 | 27.714 | -182.5753 | 2.06719 | 1 |
| 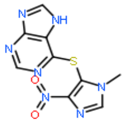 | 0 | 870 | 993 | -6.943 | 26 | 1 | -2.3332 | -3.9124 | -20.39 | 2.6824 | 17.517 | 10.037 | -73.82466 | 1.16387 | 1 |
| 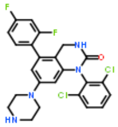 | 0 | 2575 | DQO | -6.939 | 53 | 0 | -3.3915 | -7.7429 | -29.05 | 4.7187 | 19.267 | 28.174 | -149.9641 | 1.55796 | 1 |
| 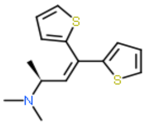 | 0 | 1250 | 1444 | -6.929 | 34 | 2 | -1.3509 | -5.7739 | -19.46 | 3.6409 | 10.105 | 13.414 | -59.63611 | 1.12466 | 1 |
| 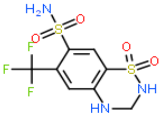 | 0 | 657 | 774 | -6.923 | 28 | 3 | -0.3278 | -2.8704 | -20.67 | 0 | 13.721 | 6.6821 | -84.33662 | 1.23295 | 1 |
| 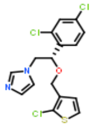 | 0 | 883 | 1007 | -6.887 | 36 | 6 | -3.5328 | -6.775 | -28.1 | 4.4079 | 16.307 | 24.572 | -127.466 | 1.57321 | 1 |

| | | | | | | | | | | | | | | | |
|---|---|---|---|---|---|---|---|---|---|---|---|---|---|---|---|
| 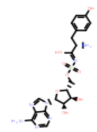 | 0 | 3001 | C19H23N7O8S | -6.883 | 58 | 12 | -12.459 | -5.1356 | -30.02 | 18.589 | 33.257 | 22.437 | -163.7654 | 1.91424 | 1 |
| 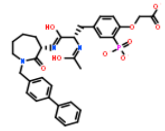 | 0 | 1676 | C32H36N3O9P | -6.871 | 78 | 13 | -6.0669 | -6.768 | -33.24 | 11.33 | 28.631 | 15.426 | -111.15 | 2.41518 | 1 |
| 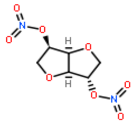 | 0 | 763 | C6H8N2O8 | -6.863 | 24 | 4 | -2.6449 | -2.5043 | -17.06 | 0.7822 | 13.307 | 8.6604 | -69.94541 | 0.97167 | 1 |
| 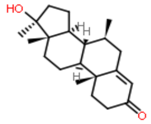 | 0 | 1360 | 1564 | -6.842 | 55 | 1 | -4.2481 | -4.9205 | -16.65 | 1.392 | 13.55 | 18.568 | -86.43007 | 1.28266 | 1 |
| 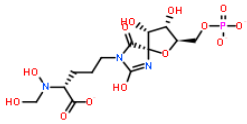 | 0 | 2386 | C13H22N3O13P | -6.834 | 49 | 15 | -13.422 | -3.5423 | -22.8 | 9.0455 | 35.496 | 17.043 | -142.5693 | 1.5774 | 1 |
| 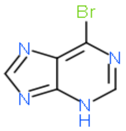 | 0 | 1922 | C5H3BrN4 | -6.824 | 13 | 0 | -2.9305 | -1.5525 | -13.96 | 0.1102 | 9.2847 | 12.964 | -37.50968 | 0.55645 | 1 |
| 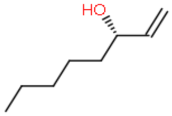 | 0 | 2719 | C8H16O | -6.823 | 25 | 6 | -4.5084 | -3.8975 | -11.07 | 1.0743 | 11.5 | 8.179 | -61.08167 | 1.02254 | 1 |
| 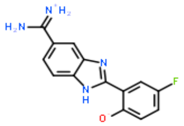 | 0 | 2576 | 802 | -6.818 | 31 | 0 | -7.6966 | -4.2009 | -20.47 | 1.4205 | 18.781 | 31.82 | -84.43934 | 1.26271 | 1 |
| 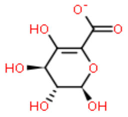 | 0 | 3223 | C6H8O7 | -6.802 | 20 | 4 | -5.9701 | -0.7408 | -8.165 | 0 | 19.335 | 0.0936 | -26.4236 | 0.79978 | 1 |
| 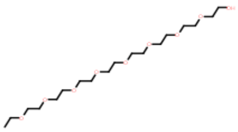 | 0 | 3212 | C18H38O9 | -6.798 | 65 | 25 | -11.262 | -6.3429 | -22.74 | 4.3877 | 20.178 | 24.121 | -112.4921 | 2.22355 | 1 |
| 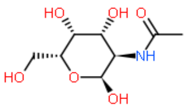 | 0 | 3221 | Untitled | -6.795 | 30 | 6 | -8.0359 | -2.7365 | -14.58 | 0 | 21.424 | 15.57 | -77.56783 | 1.08005 | 1 |

| | | | | | | | | | | | | | | | |
|---|---|---|---|---|---|---|---|---|---|---|---|---|---|---|---|
| 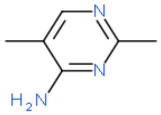 | 0 | 2111 | C6H9N3 | -6.794 | 18 | 0 | -4.3502 | -3.5546 | -9.793 | 0.763 | 12.229 | 10.238 | -33.16581 | 0.74942 | 1 |
| 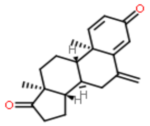 | 0 | 867 | 990 | -6.791 | 46 | 0 | -2.3319 | -4.6479 | -20.27 | 1.7006 | 11.568 | 19.389 | -83.5382 | 1.1603 | 1 |
| 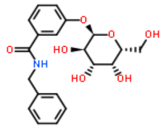 | 0 | 3113 | AI1 | -6.79 | 51 | 8 | -6.4209 | -5.8079 | -24.96 | 7.5265 | 21.899 | 19.039 | -163.7317 | 1.84241 | 1 |
| 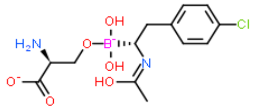 | 0 | 3258 | C13H19BClN2O6 | -6.782 | 41 | 12 | -7.3787 | -4.2852 | -19.63 | 0 | 22.48 | 15.65 | -54.91657 | 1.33858 | 1 |
| 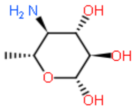 | 0 | 2202 | C6H13NO4 | -6.769 | 24 | 4 | -9.0618 | -2.3016 | -6.29 | 3.5286 | 16.544 | 10.704 | -35.25483 | 0.62537 | 1 |
| 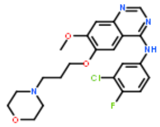 | 0 | 211 | 317 | -6.762 | 55 | 4 | -5.2489 | -7.9446 | -27.98 | 10.997 | 20.214 | 23.732 | -130.8565 | 1.62479 | 1 |
| 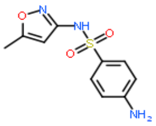 | 0 | 891 | C10H11N3O3S | -6.76 | 28 | 3 | -1.8408 | -3.9095 | -18.02 | 1.1077 | 11.589 | 11.188 | -69.90646 | 1.11474 | 1 |
| 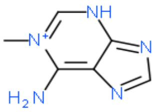 | 0 | 2846 | C6H8N5 | -6.758 | 19 | 0 | -3.4453 | -1.8762 | -13.68 | 0 | 12.81 | 11.047 | -5.642409 | 0.64313 | 1 |
| 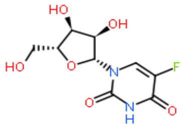 | 0 | 1421 | C9H11FN2O6 | -6.753 | 29 | 5 | -4.0477 | -2.4341 | -17.56 | 0 | 15.772 | 11.893 | -107.5638 | 1.18225 | 1 |
| 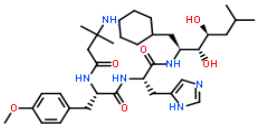 | 0 | 3065 | C35H56N6O6 | -6.749 | 103 | 20 | -5.0257 | -10.283 | -42.62 | 9.903 | 27.387 | 25.751 | -187.8139 | 2.57701 | 1 |
| 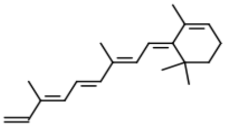 | 0 | 2613 | C20H28 | -6.741 | 48 | 0 | 0 | -6.7966 | -23.59 | 0 | 11.041 | 19.876 | -75.72965 | 1.392 | 1 |

| | | | | | | | | | | | | | | | |
|---|---|---|---|---|---|---|---|---|---|---|---|---|---|---|---|
| 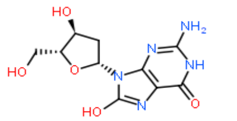 | 0 | 2232 | 2,5-Furandicarboxylate.mol | -6.738 | 33 | 4 | -6.9878 | -2.7044 | -18.71 | 2.1701 | 21.214 | 17.963 | -101.0621 | 1.28619 | 1 |
| 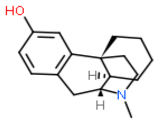 | 0 | 734 | 854 | -6.722 | 42 | 0 | -3.4749 | -4.417 | -15.9 | 0.7665 | 12.278 | 17.054 | -86.11671 | 0.85509 | 1 |
| 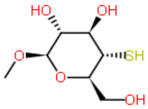 | 0 | 1440 | C7H14O5S | -6.702 | 27 | 6 | -6.3166 | -3.136 | -10.31 | 0.3602 | 15.374 | 9.6097 | -74.31115 | 0.96139 | 1 |
| 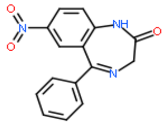 | 0 | 1389 | 1595 | -6.699 | 32 | 2 | -4.9568 | -4.0893 | -19.14 | 1.749 | 15.716 | 20.737 | -96.0884 | 1.25304 | 1 |
| 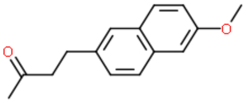 | 0 | 351 | 461 | -6.698 | 33 | 3 | -2.1455 | -5.3643 | -20.66 | 3.2049 | 12.743 | 14.701 | -111.6252 | 1.52499 | 1 |
| 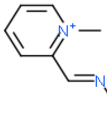 | 0 | 616 | C7H9N2O | -6.69 | 19 | 1 | -2.2173 | -2.2379 | -13.09 | 0.8099 | 10.616 | 6.7668 | -13.96816 | 0.61179 | 1 |
| 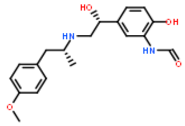 | 0 | 1136 | 1274 | -6.686 | 49 | 8 | -7.3348 | -5.0588 | -25.12 | 7.9638 | 21.701 | 22.005 | -102.0601 | 1.65639 | 1 |
| 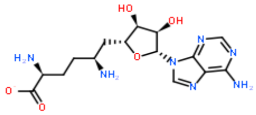 | 0 | 1678 | C15H23N7O5 | -6.682 | 49 | 11 | -14.808 | -4.031 | -19.5 | 6.4233 | 33.312 | 26.579 | -112.0631 | 1.36629 | 1 |
| 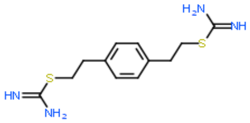 | 0 | 1898 | 4BT | -6.681 | 36 | 6 | -8.3991 | -3.8906 | -25.25 | 7.6287 | 24.259 | 24.327 | -95.43974 | 1.44699 | 1 |
| 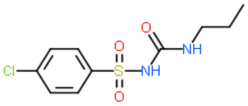 | 0 | 558 | 672 | -6.649 | 30 | 6 | -1.179 | -4.2111 | -21.97 | 1.8498 | 13.344 | 9.7009 | -84.78138 | 1.34022 | 1 |
| 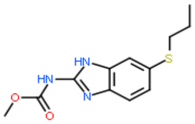 | 0 | 405 | 518 | -6.641 | 33 | 3 | -4.6303 | -5.4032 | -22.13 | 10.144 | 15.698 | 16.923 | -96.01476 | 1.45566 | 1 |

| | | | | | | | | | | | | | | | |
|---|---|---|---|---|---|---|---|---|---|---|---|---|---|---|---|
| 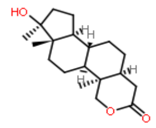 | 0 | 508 | 621 | -6.634 | 52 | 1 | -2.8205 | -4.602 | -18.5 | 0.4001 | 13.073 | 17.336 | -98.01505 | 1.20932 | 1 |
| 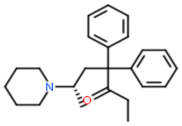 | 0 | 1294 | 1491 | -6.629 | 57 | 7 | -1.3771 | -6.3523 | -23.08 | 1.8257 | 12.493 | 14.425 | -90.29715 | 1.16557 | 1 |
| 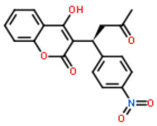 | 0 | 1225 | m | -6.62 | 41 | 5 | -3.4396 | -4.8888 | -27.59 | 4.0241 | 16.429 | 23.055 | -113.826 | 1.51048 | 1 |
| 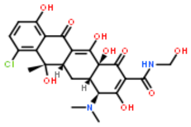 | 0 | 343 | m | -6.615 | 60 | 5 | -5.8701 | -4.9014 | -22.95 | 5.7997 | 21.1 | 18.433 | -101.3917 | 1.72926 | 1 |
| 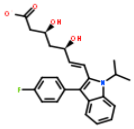 | 0 | 967 | 1095 | -6.607 | 55 | 9 | -4.8491 | -6.2177 | -24.22 | 8.3432 | 19.55 | 13.599 | -118.307 | 1.73933 | 1 |
| 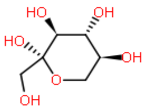 | 0 | 2289 | $C_6H_{12}O_6$ | -6.598 | 24 | 6 | -6.0693 | -1.303 | -9.106 | 1.3963 | 14.746 | 4.9077 | -15.08345 | 0.78146 | 1 |
| 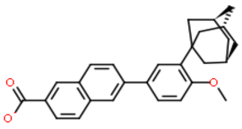 | 0 | 105 | 210 | -6.586 | 58 | 2 | -1.289 | -7.1956 | -27.59 | 2.7216 | 17.591 | 19.78 | -155.9369 | 1.89801 | 1 |
| 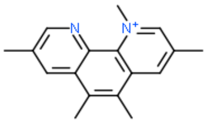 | 0 | 2052 | $C_{17}H_{19}N_2$ | -6.582 | 38 | 0 | 0 | -5.8836 | -18.39 | 1.6811 | 7.3021 | 14.032 | -104.6007 | 1.19937 | 1 |
| 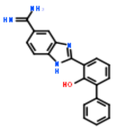 | 0 | 1510 | 130 | -6.567 | 41 | 1 | -4.6024 | -5.4049 | -27.95 | 6.4132 | 18.805 | 27.42 | -118.6057 | 1.68094 | 1 |
| 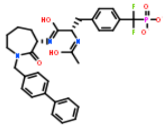 | 0 | 3243 | $C_{31}H_{34}F_2N_3O_6P$ | -6.561 | 75 | 12 | -5.0401 | -8.5236 | -32.05 | 10.587 | 26.822 | 15.577 | -172.8553 | 2.74981 | 1 |
| 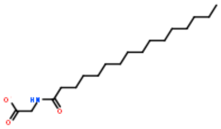 | 0 | 3107 | 140 | -6.557 | 56 | 16 | -8.3898 | -6.9964 | -20.64 | 5.7846 | 18.474 | 19.807 | -129.8624 | 1.65584 | 1 |

| | | | | | | | | | | | | | | | |
|---|---|---|---|---|---|---|---|---|---|---|---|---|---|---|---|
| 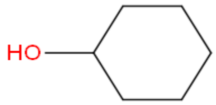 | 0 | 3346 | C6H12O | -6.557 | 19 | 1 | -3.5349 | -2.4815 | -9.101 | 0.3264 | 7.9234 | 9.715 | -45.32345 | 0.70455 | 1 |
| 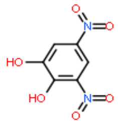 | 0 | 1863 | C6H4N2O6 | -6.551 | 18 | 2 | -0.6537 | -1.8277 | -16.58 | 0.373 | 9.4231 | 6.6864 | -64.8672 | 0.85251 | 1 |
| 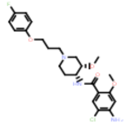 | 0 | 491 | C23H29ClFN3O4 | -6.544 | 61 | 7 | -2.9789 | -8.1534 | -26.18 | 4.403 | 16.805 | 19.988 | -95.121 | 1.84906 | 1 |
| 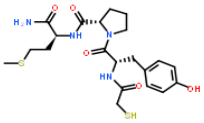 | 0 | 1656 | SD2 | -6.533 | 62 | 13 | -9.1711 | -5.8018 | -31.69 | 10.022 | 26.304 | 28.34 | -123.6522 | 1.61452 | 1 |
| 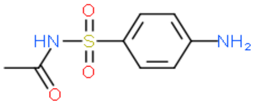 | 0 | 521 | 634 | -6.523 | 24 | 2 | -1.339 | -3.1963 | -19.26 | 1.0451 | 12.499 | 10.705 | -67.82392 | 0.97049 | 1 |
| 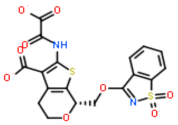 | 0 | 2530 | DBD | -6.517 | 43 | 4 | -3.3285 | -5.0447 | -24.64 | 1.6329 | 18.796 | 18.863 | -151.5151 | 1.90348 | 1 |
| 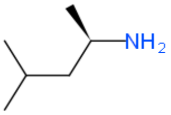 | 0 | 1865 | C6H15N | -6.517 | 22 | 3 | -3.9705 | -2.696 | -11.38 | 1.9409 | 9.091 | 10.497 | -35.88272 | 0.68787 | 1 |
| 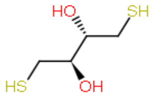 | 0 | 1480 | C4H10O2S2 | -6.517 | 18 | 7 | -6.323 | -1.4988 | -11.14 | 4.6396 | 10.512 | 10.241 | -49.68426 | 0.72541 | 1 |
| 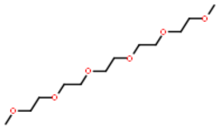 | 0 | 2306 | PG6 | -6.514 | 44 | 15 | -9.5612 | -5.68 | -19.08 | 8.3007 | 18.339 | 19.436 | -63.65289 | 1.44063 | 1 |
| 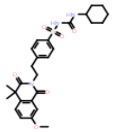 | 0 | 1120 | 1251 | -6.514 | 70 | 8 | -4.5343 | -7.1128 | -33.08 | 4.4983 | 22.154 | 28.32 | -182.1322 | 2.1376 | 1 |
| 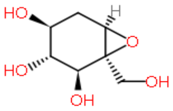 | 0 | 2130 | C7H12O5 | -6.512 | 24 | 5 | -5.8798 | -2.0104 | -9.337 | 0 | 14.258 | 8.1133 | -50.88336 | 0.77007 | 1 |

| | | | | | | | | | | | | | | | |
|---|---|---|---|---|---|---|---|---|---|---|---|---|---|---|---|
| 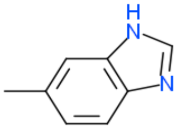 | 0 | 2859 | C8H8N2 | -6.499 | 18 | 0 | -3.1234 | -2.9694 | -12.46 | 0.4302 | 8.1919 | 14.318 | -54.49374 | 0.61882 | 1 |
| 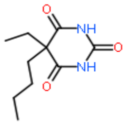 | 0 | 1181 | 1353 | -6.48 | 31 | 4 | -4.8701 | -3.1301 | -16.09 | 1.4321 | 15.143 | 14.254 | -87.86291 | 1.15742 | 1 |
| 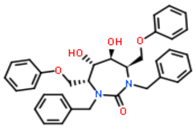 | 0 | 1516 | C33H34N2O5 | -6.451 | 74 | 12 | -2.6 | -7.6793 | -34.81 | 8.6218 | 18.456 | 21.016 | -149.7024 | 2.03397 | 1 |
| 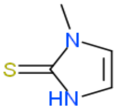 | 0 | 646 | 763 | -6.428 | 13 | 0 | -1.9291 | -2.3072 | -13.24 | 0.2883 | 7.5682 | 11.2 | -51.1705 | 0.49462 | 1 |
| 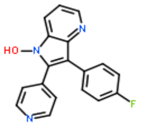 | 0 | 1947 | FPH | -6.416 | 35 | 2 | -2.8356 | -5.4566 | -20.8 | 3.357 | 13.789 | 17.658 | -98.34819 | 1.05925 | 1 |
| 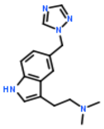 | 0 | 831 | 953 | -6.387 | 39 | 5 | -5.4347 | -5.7222 | -22.56 | 5.2383 | 16.393 | 23.208 | -93.935 | 1.23152 | 1 |
| 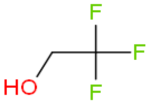 | 0 | 2907 | C2H3F3O | -6.371 | 9 | 1 | -4.1149 | -2.1863 | -6.823 | 0.3975 | 8.1623 | 8.1311 | -46.33112 | 0.3373 | 1 |
| 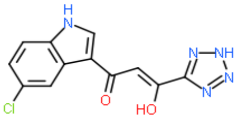 | 0 | 2805 | C12H8ClN5O2 | -6.356 | 28 | 0 | -4.6275 | -3.7973 | -23.56 | 1.0433 | 18.355 | 25.856 | -106.4159 | 1.4194 | 1 |
| 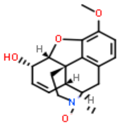 | 0 | 1364 | 1568 | -6.347 | 44 | 1 | -3.4989 | -4.7402 | -16.98 | 0.7731 | 14.776 | 15.938 | -72.50375 | 1.0944 | 1 |
| 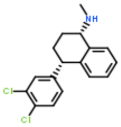 | 0 | 976 | 1104 | -6.337 | 37 | 2 | -0.5107 | -5.7468 | -20.28 | 1.0016 | 9.0914 | 15.864 | -82.56554 | 0.91664 | 1 |
| 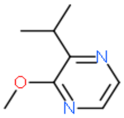 | 0 | 1542 | C8H12N2O | -6.33 | 23 | 1 | -2.2496 | -3.864 | -12.39 | 0.9576 | 8.9145 | 9.8151 | -40.14928 | 0.7856 | 1 |

| | | | | | | | | | | | | | | | |
|---|---|---|---|---|---|---|---|---|---|---|---|---|---|---|---|
| 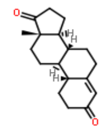 | 0 | 1240 | 1434 | -6.324 | 44 | 0 | -2.0031 | -4.6565 | -15.13 | 0.7237 | 14.481 | 8.628 | -126.4283 | 1.62566 | 1 |
| 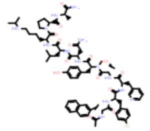 | 0 | 10 | C72H95ClN14O14 | -6.308 | 196 | 41 | -13.566 | -12.66 | -71.13 | 40.896 | 46.31 | 35.997 | -302.4486 | 5.16122 | 1 |
| 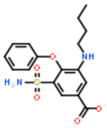 | 0 | 767 | 887 | -6.306 | 44 | 8 | -4.3154 | -4.8745 | -17.76 | 3.0056 | 15.599 | 11.113 | -92.22601 | 1.26675 | 1 |
| 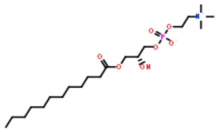 | 0 | 1493 | C20H43NO7P | -6.3 | 71 | 20 | -8.1969 | -6.55 | -29.43 | 10.756 | 21.234 | 21.024 | -129.4587 | 1.91242 | 1 |
| 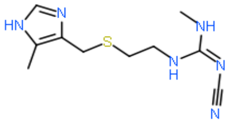 | 0 | 391 | C10H16N6S | -6.295 | 33 | 7 | -5.8951 | -4.8269 | -19.46 | 3.3628 | 15.945 | 19.902 | -66.21464 | 1.13862 | 1 |
| 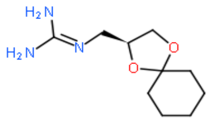 | 0 | 121 | 38521 | -6.293 | 34 | 2 | -5.373 | -3.6645 | -13.08 | 0.8169 | 18.09 | 11.485 | -102.8101 | 1.28149 | 1 |
| 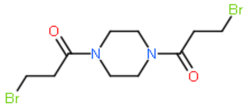 | 0 | 131 | 236 | -6.288 | 32 | 6 | -3.6238 | -4.9917 | -19.03 | 1.0938 | 13.841 | 16.09 | -101.5866 | 1.73015 | 1 |
| 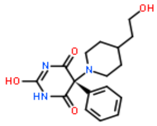 | 0 | 3272 | BBT | -6.277 | 45 | 6 | -7.7212 | -4.1619 | -21.64 | 1.8799 | 23.901 | 22.779 | -105.3312 | 1.47835 | 1 |
| 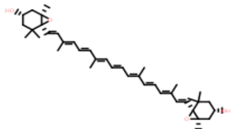 | 0 | 3126 | C40H56O4 | -6.266 | 100 | 4 | -3.3742 | -11.639 | -37.34 | 5.3048 | 27.24 | 32.41 | -182.8575 | 2.94089 | 1 |
| 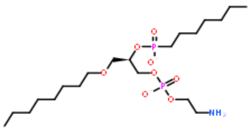 | 0 | 3219 | C20H45NO8P2 | -6.265 | 74 | 24 | -12.399 | -6.503 | -30.3 | 14.928 | 30.75 | 20.215 | -119.8785 | 2.44319 | 1 |
| 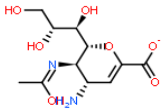 | 0 | 2998 | C11H18N2O7 | -6.259 | 37 | 10 | -6.3236 | -2.2344 | -17.79 | 3.6709 | 22.193 | 6.3266 | -45.19251 | 1.31062 | 1 |

| | | | | | | | | | | | | | | | |
|---|---|---|---|---|---|---|---|---|---|---|---|---|---|---|---|
| 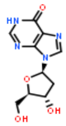 | 0 | 2119 | ChEBI | -6.252 | 30 | 4 | -5.7036 | -2.2201 | -15.65 | 0.4156 | 19.983 | 11.632 | -40.37618 | 1.00714 | 1 |
| 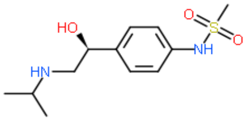 | 0 | 379 | 489 | -6.251 | 38 | 7 | -5.6586 | -4.7565 | -21.07 | 4.5255 | 16.278 | 19.994 | -71.81406 | 1.62829 | 1 |
| 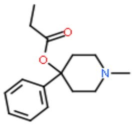 | 0 | 1281 | 1478 | -6.245 | 39 | 3 | -1.6167 | -4.624 | -16.97 | 1.3779 | 10.907 | 10.761 | -29.86622 | 0.87273 | 1 |
| 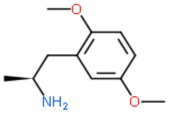 | 0 | 1268 | 1465 | -6.225 | 31 | 3 | -2.0769 | -3.7842 | -18.34 | 4.2127 | 12.937 | 8.9047 | -17.3154 | 0.85076 | 1 |
| 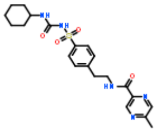 | 0 | 939 | C21H27N5O4S | -6.222 | 58 | 8 | -2.7999 | -6.387 | -35.06 | 8.41 | 16.888 | 26.803 | -127.6512 | 2.08016 | 1 |
| 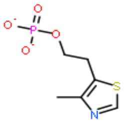 | 0 | 2828 | C6H10NO4PS | -6.214 | 21 | 4 | -7.6046 | -3.2507 | -11.58 | 0 | 15.971 | 18.79 | -58.48345 | 0.91633 | 1 |
| 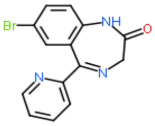 | 0 | 1354 | 1558 | -6.208 | 29 | 0 | -2.0516 | -4.5974 | -22 | 1.0966 | 15.343 | 18.021 | -56.02273 | 1.02396 | 1 |
| 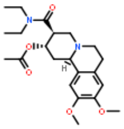 | 0 | 650 | 767 | -6.203 | 61 | 5 | -3.5798 | -7.3499 | -25.35 | 6.8506 | 18.738 | 18.122 | -117.4843 | 1.64392 | 1 |
| 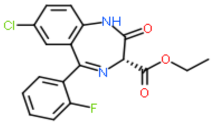 | 0 | 1341 | 1545 | -6.201 | 39 | 2 | -1.7763 | -5.7522 | -22.48 | 1.3658 | 14.694 | 17.556 | -93.2042 | 1.21655 | 1 |
| 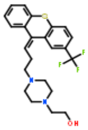 | 0 | 755 | C23H25F3N2OS | -6.198 | 55 | 6 | -4.8517 | -7.3264 | -25.14 | 4.8651 | 18.878 | 23.407 | -138.2045 | 1.88437 | 1 |
| 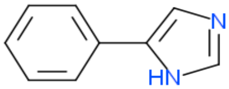 | 0 | 2935 | C9H8N2 | -6.172 | 19 | 1 | -3.1581 | -3.1111 | -12.45 | 0.7581 | 8.0961 | 13.979 | -44.32342 | 0.60211 | 1 |

| | | | | | | | | | | | | | | | |
|---|---|---|---|---|---|---|---|---|---|---|---|---|---|---|---|
| 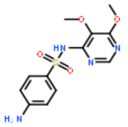 | 0 | 1151 | 1299 | -6.169 | 35 | 3 | -5.0499 | -4.9765 | -20.5 | 0 | 20.593 | 19.988 | -111.596 | 1.31512 | 1 |
| 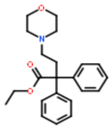 | 0 | 1278 | 1475 | -6.16 | 53 | 7 | -1.4007 | -6.8146 | -24.71 | 4.8319 | 11.364 | 16.73 | -96.30593 | 1.11026 | 1 |
| 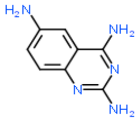 | 0 | 2262 | TAQ | -6.157 | 22 | 0 | -3.46 | -3.7116 | -13.07 | 0.5208 | 15.78 | 9.0961 | -57.65308 | 0.77884 | 1 |
| 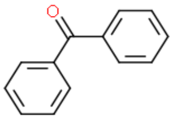 | 0 | 1651 | C13H10O | -6.15 | 24 | 2 | -1.9375 | -4.2738 | -16.89 | 2.3775 | 10.822 | 11.768 | -89.61137 | 1.13965 | 1 |
| 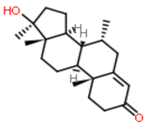 | 0 | 1274 | 1471 | -6.137 | 55 | 1 | -4.3555 | -4.7366 | -17.24 | 1.8881 | 14.17 | 19.601 | -82.93969 | 1.29816 | 1 |
| 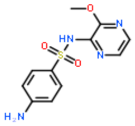 | 0 | 551 | 664 | -6.132 | 31 | 3 | -1.6442 | -4.5613 | -25 | 3.5208 | 14.189 | 17.472 | -95.31638 | 1.25482 | 1 |
| 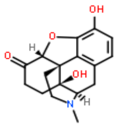 | 0 | 1062 | 1192 | -6.122 | 41 | 1 | -2.1268 | -4.2824 | -17.11 | 0 | 15.729 | 10.367 | -94.64481 | 1.23968 | 1 |
| 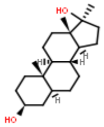 | 0 | 1311 | C20H34O2 | -6.109 | 56 | 2 | -4.1379 | -4.8486 | -15.57 | 0.7544 | 14.152 | 16.539 | -80.12041 | 1.13438 | 1 |
| 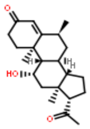 | 0 | 148 | 253 | -6.108 | 57 | 2 | -3.8457 | -4.8977 | -20.01 | 2.8245 | 15.524 | 18.744 | -92.58051 | 1.19585 | 1 |
| 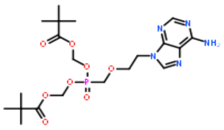 | 0 | 602 | 718 | -6.105 | 66 | 13 | -5.7316 | -7.6147 | -31.37 | 4.2797 | 24.85 | 24.032 | -120.898 | 1.87677 | 1 |
| 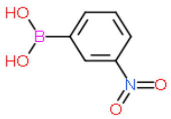 | 0 | 2502 | C6H6BNO4 | -6.093 | 18 | 3 | -1.4091 | -2.1474 | -16.19 | 0.9015 | 11.688 | 6.0519 | -47.65272 | 0.75079 | 1 |

| | | | | | | | | | | | | | | | |
|---|---|---|---|---|---|---|---|---|---|---|---|---|---|---|---|
| 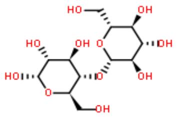 | 0 | 1820 | C12H22O11 | -6.087 | 45 | 12 | -10.032 | -3.3485 | -17.95 | 7.8094 | 25.205 | 13.183 | -119.4935 | 1.64074 | 1 |
| 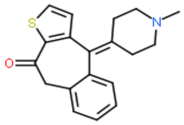 | 0 | 799 | 920 | -6.077 | 41 | 0 | -1.8925 | -5.1618 | -21.17 | 1.4807 | 11.972 | 20.411 | -52.99954 | 1.10227 | 1 |
| 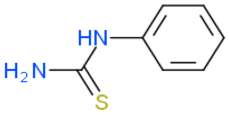 | 0 | 3337 | C7H8N2S | -6.069 | 18 | 2 | -1.2645 | -2.6565 | -13.92 | 0.1696 | 9.7003 | 6.4454 | -44.4161 | 0.78646 | 1 |
| 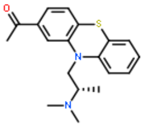 | 0 | 1408 | 1615 | -6.065 | 45 | 3 | -2.329 | -6.089 | -25.89 | 0.9342 | 15.451 | 24.013 | -110.5979 | 1.54389 | 1 |
| 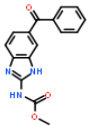 | 0 | 530 | 643 | -6.056 | 35 | 2 | -2.7113 | -5.2204 | -24.67 | 0 | 18.271 | 21.314 | -120.6096 | 1.60795 | 1 |
| 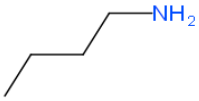 | 0 | 3307 | C4H11N | -6.055 | 16 | 3 | -3.8135 | -2.6339 | -8.737 | 0.1117 | 10.735 | 6.3286 | -28.56375 | 0.63278 | 1 |
| 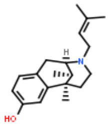 | 0 | 539 | m | -6.049 | 48 | 2 | -3.6446 | -5.1855 | -18.88 | 4.1466 | 13.973 | 17.212 | -92.04456 | 1.07628 | 1 |
| 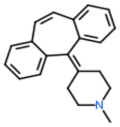 | 0 | 324 | 434 | -6.042 | 43 | 0 | 0 | -5.6196 | -23.12 | 1.3981 | 10.295 | 18.611 | -63.03811 | 1.13637 | 1 |
| 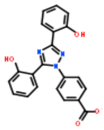 | 0 | 1402 | m | -6.022 | 42 | 2 | -4.2027 | -4.7805 | -23.04 | 5.4078 | 20.449 | 16.783 | -116.9477 | 1.83188 | 1 |
| 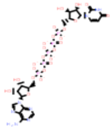 | 0 | 3312 | C19H28N7O24P5 | -5.968 | 78 | 20 | -22.122 | -3.5619 | -34.52 | 1.5835 | 42.353 | 63.918 | -175.5796 | 2.45508 | 1 |
| 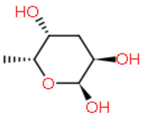 | 0 | 2315 | C6H12O4 | -5.966 | 22 | 3 | -6.1479 | -2.6317 | -11.31 | 0 | 14.895 | 14.68 | -70.09005 | 0.68481 | 1 |

| | | | | | | | | | | | | | | | |
|---|---|---|---|---|---|---|---|---|---|---|---|---|---|---|---|
| 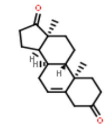 | 0 | 1260 | m | -5.956 | 47 | 0 | -1.9823 | -4.4873 | -19.04 | 0.6514 | 11.423 | 18.401 | -89.23395 | 1.12993 | 1 |
| 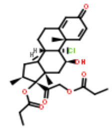 | 0 | 285 | [NO | -5.951 | 73 | 7 | -7.141 | -7.67 | -27.97 | 5.2302 | 24.068 | 30.056 | -180.2096 | 1.89773 | 1 |
| 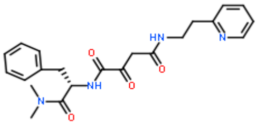 | 0 | 1897 | C48 | -5.95 | 56 | 11 | -4.1882 | -6.3198 | -32.33 | 8.2287 | 19.845 | 22.413 | -157.3445 | 2.1228 | 1 |
| 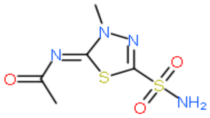 | 0 | 588 | C5H8N4O3S2 | -5.945 | 22 | 2 | -1.7283 | -2.6687 | -18.34 | 0 | 14.684 | 9.6107 | -43.08125 | 0.97064 | 1 |
| 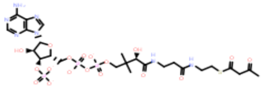 | 0 | 2748 | C25H40N7O18P3S | -5.944 | 90 | 23 | -14.697 | -7.0509 | -37.99 | 0 | 46.49 | 38.305 | -156.461 | 2.95427 | 1 |
| 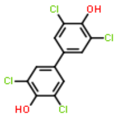 | 0 | 3021 | PCQ | -5.928 | 24 | 0 | -2.286 | -4.9357 | -16.47 | 1.0807 | 14.685 | 12.082 | -96.04684 | 1.52026 | 1 |
| 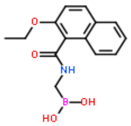 | 0 | 2544 | NBF | -5.907 | 36 | 5 | -5.3292 | -4.1995 | -17.46 | 0.1375 | 15.334 | 20.103 | -58.78606 | 1.14263 | 1 |
| 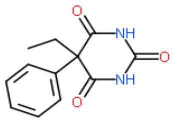 | 0 | 1044 | 1174 | -5.904 | 29 | 2 | -1.9597 | -3.6351 | -20.42 | 2.5331 | 13.259 | 14.007 | -91.38635 | 1.03952 | 1 |
| 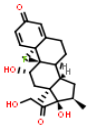 | 0 | 1103 | C22H29FO5 | -5.899 | 57 | 5 | -5.9108 | -4.7301 | -21.23 | 7.3029 | 20.039 | 16.736 | -88.71639 | 1.30173 | 1 |
| 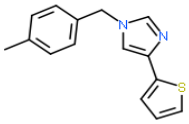 | 0 | 2722 | 654 | -5.899 | 32 | 2 | -0.6598 | -5.8847 | -20.34 | 0.5663 | 14.4 | 11.694 | -75.79012 | 1.39139 | 1 |
| 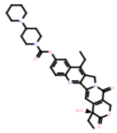 | 0 | 645 | C33H38N4O6 | -5.89 | 81 | 5 | -2.9641 | -9.4692 | -31.2 | 2.3534 | 22.585 | 26.978 | -172.2173 | 2.55846 | 1 |

| | | | | | | | | | | | | | | | |
|---|---|---|---|---|---|---|---|---|---|---|---|---|---|---|---|
| 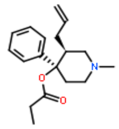 | 0 | 1338 | 1542 | -5.885 | 46 | 5 | -1.5431 | -4.8214 | -20.42 | 3.5123 | 13.102 | 10.201 | -53.17698 | 1.28267 | 1 |
| 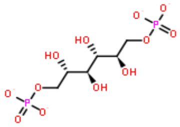 | 0 | 2471 | C6H16O12P2 | -5.884 | 32 | 13 | -11.495 | -2.0576 | -15.67 | 0 | 21.659 | 24.548 | -119.8637 | 1.28096 | 1 |
| 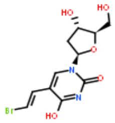 | 0 | 2989 | C11H13BrN2O5 | -5.882 | 32 | 5 | -4.9157 | -3.4524 | -20.47 | 0 | 19.575 | 17.654 | -65.44053 | 1.0229 | 1 |
| 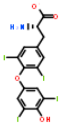 | 0 | 341 | 451 | -5.869 | 34 | 6 | -5.1827 | -6.5935 | -25.2 | 0.4594 | 24.274 | 22.398 | -68.81934 | 1.74166 | 1 |
| 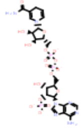 | 0 | 2079 | C21H30N7O17P3 | -5.864 | 74 | 15 | -21.502 | -3.9157 | -36.22 | 16.828 | 42.298 | 55.396 | -171.9777 | 2.6758 | 1 |
| 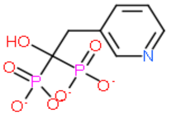 | 0 | 764 | 884 | -5.858 | 24 | 5 | -13.156 | -2.5257 | -17.79 | 1.6132 | 17.907 | 44.714 | -82.19006 | 0.8812 | 1 |
| 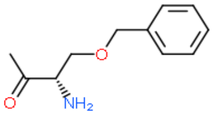 | 0 | 1905 | C11H15NO2 | -5.854 | 29 | 6 | -5.1539 | -4.3442 | -18.51 | 3.6062 | 14.021 | 18.673 | -76.52617 | 1.07595 | 1 |
| 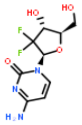 | 0 | 331 | 441 | -5.849 | 29 | 4 | -7.2985 | -3.0452 | -11.1 | 3.5313 | 18.509 | 11.143 | -81.4479 | 0.96516 | 1 |
| 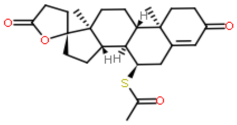 | 0 | 311 | 421 | -5.842 | 61 | 1 | -1.3859 | -6.4259 | -24.44 | 1.5424 | 14.675 | 21.029 | -117.5489 | 1.42014 | 1 |
| 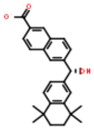 | 0 | 3130 | C26H28O3 | -5.824 | 56 | 4 | -2.2253 | -6.1816 | -28.13 | 3.2757 | 19.698 | 19.602 | -148.291 | 1.93718 | 1 |
| 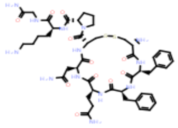 | 0 | 8 | C46H65N13O11S2 | -5.819 | 137 | 22 | -12.06 | -8.004 | -45.1 | 23.85 | 39.17 | 27.673 | -227.6676 | 3.73998 | 1 |

| | | | | | | | | | | | | | | | |
|---|---|---|---|---|---|---|---|---|---|---|---|---|---|---|---|
| 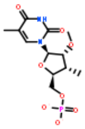 | 0 | 2930 | C12H19N2O8P | -5.809 | 40 | 5 | -10.646 | -3.4522 | -20.02 | 11.997 | 22.609 | 24.705 | -124.4856 | 1.49359 | 1 |
| 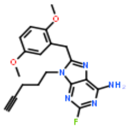 | 0 | 2820 | PUZ | -5.801 | 47 | 6 | -4.7413 | -6.7449 | -20.7 | 5.0342 | 17.732 | 17.413 | -91.13844 | 1.31292 | 1 |
| 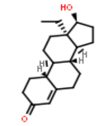 | 0 | 1339 | C19H28O2 | -5.789 | 49 | 2 | -2.6435 | -4.5221 | -18.61 | 1.3257 | 12.441 | 16.988 | -58.60601 | 1.06841 | 1 |
| 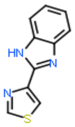 | 0 | 613 | 730 | -5.789 | 21 | 0 | -3.4518 | -4.634 | -18.65 | 4.9114 | 12.949 | 18.238 | -64.82985 | 1.13992 | 1 |
| 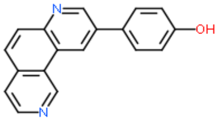 | 0 | 3273 | 9HP | -5.785 | 33 | 0 | -2.8821 | -5.261 | -21.61 | 3.7478 | 14.878 | 20.046 | -109.3862 | 1.21315 | 1 |
| 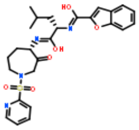 | 0 | 3291 | C26H30N4O6S | -5.779 | 67 | 9 | -1.2875 | -7.1258 | -32.04 | 6.06 | 17.524 | 18.35 | -112.2654 | 2.14726 | 1 |
| 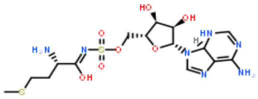 | 0 | 1977 | C15H25N7O7S2 | -5.775 | 56 | 13 | -6.8487 | -4.2952 | -34.64 | 10.14 | 27.974 | 21.523 | -95.4269 | 1.93822 | 1 |
| 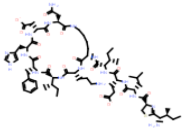 | 0 | 513 | C66H103N17O16S | -5.752 | 201 | 33 | -10.203 | -12.237 | -56.34 | 4.4517 | 53.265 | 33.601 | -276.2189 | 5.23482 | 1 |
| 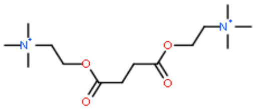 | 0 | 97 | 202 | -5.738 | 50 | 9 | -6.9547 | -4.9523 | -21.58 | 3.2739 | 17.085 | 25.11 | -105.4441 | 2.03005 | 1 |
| 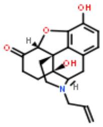 | 0 | 1053 | C19H21NO4 | -5.737 | 45 | 3 | -4.6586 | -3.9963 | -19.98 | 4.0914 | 14.962 | 20.039 | -97.13136 | 1.20867 | 1 |
| 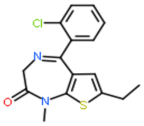 | 0 | 1355 | 1559 | -5.736 | 36 | 1 | -2.0053 | -5.5479 | -21.6 | 1.3308 | 13.007 | 20.453 | -70.33721 | 1.02706 | 1 |

| | | | | | | | | | | | | | | | |
|---|---|---|---|---|---|---|---|---|---|---|---|---|---|---|---|
| 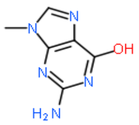 | 0 | 2219 | C6H7N5O | -5.732 | 19 | 0 | -5.6701 | -2.9187 | -11.18 | 0 | 16.935 | 13.711 | -55.80627 | 0.83018 | 1 |
| 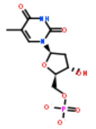 | 0 | 1435 | C10H15N2O8P | -5.722 | 34 | 5 | -10.223 | -3.0192 | -14.66 | 0 | 21.296 | 27.052 | -99.97637 | 1.29405 | 1 |
| 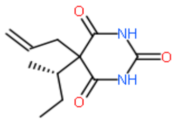 | 0 | 200 | C11H16N2O3 | -5.713 | 32 | 4 | -4.2495 | -3.2642 | -16.49 | 0.3455 | 13.792 | 16.312 | -73.90974 | 1.06549 | 1 |
| 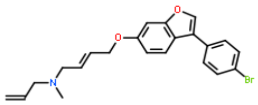 | 0 | 3410 | C22H22BrNO2 | -5.711 | 48 | 7 | 0 | -7.4599 | -30.33 | 5.7776 | 16.198 | 14.847 | -125.9106 | 2.00735 | 1 |
| 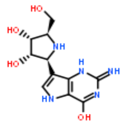 | 0 | 1978 | C11H15N5O4 | -5.706 | 35 | 6 | -7.3257 | -2.6549 | -22.66 | 2.9732 | 21.634 | 23.721 | -107.0123 | 1.30703 | 1 |
| 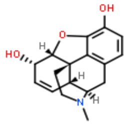 | 0 | 189 | 295 | -5.702 | 40 | 1 | -4.162 | -3.6675 | -17.35 | 0.1851 | 14.845 | 19.376 | -83.97838 | 1.11065 | 1 |
| 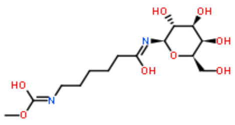 | 0 | 2207 | C14H26N2O8 | -5.678 | 50 | 14 | -7.0844 | -3.8819 | -22.83 | 3.5017 | 20.723 | 17.634 | -70.13297 | 1.63084 | 1 |
| 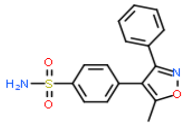 | 0 | 467 | 580 | -5.665 | 36 | 3 | -2.0604 | -4.163 | -24.07 | 3.3264 | 14.427 | 17.869 | -80.85369 | 1.11584 | 1 |
| 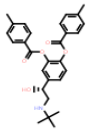 | 0 | 780 | C28H31NO5 | -5.65 | 65 | 5 | -3.6837 | -7.9971 | -26.8 | 11.949 | 19.056 | 17.225 | -151.2756 | 2.00374 | 1 |
| 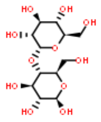 | 0 | 3000 | C12H22O11 | -5.648 | 45 | 12 | -9.9807 | -2.9221 | -11.16 | 3.609 | 19.881 | 12.791 | -109.2695 | 1.22531 | 1 |
| 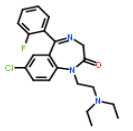 | 0 | 576 | 690 | -5.636 | 50 | 5 | -4.0973 | -6.4833 | -24.22 | 6.1548 | 16.086 | 22.03 | -130.0975 | 2.02682 | 1 |

| | | | | | | | | | | | | | | | |
|---|---|---|---|---|---|---|---|---|---|---|---|---|---|---|---|
| 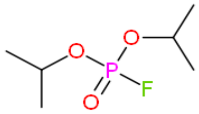 | 0 | 563 | C6H14FO3P | -5.63 | 25 | 4 | -3.4862 | -4.1166 | -17.05 | 1.5647 | 10.704 | 17.617 | -75.03588 | 1.05775 | 1 |
| 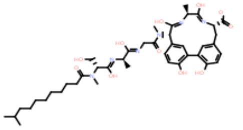 | 0 | 1699 | C42H60N6O11 | -5.628 | 118 | 25 | -6.2992 | -9.1035 | -36.48 | 0.5783 | 33.457 | 18.86 | -162.6519 | 3.17344 | 1 |
| 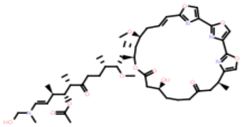 | 0 | 2715 | C46H66N4O13 | -5.618 | 129 | 16 | -6.8175 | -10.255 | -45.53 | 15.675 | 31.601 | 32.306 | -135.2493 | 3.40964 | 1 |
| 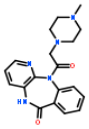 | 0 | 556 | 670 | -5.608 | 47 | 3 | -2.5148 | -5.411 | -25.31 | 6.3546 | 15.157 | 19.251 | -80.02047 | 1.37884 | 1 |
| 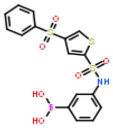 | 0 | 2561 | ETP | -5.597 | 41 | 7 | -4.6504 | -4.6262 | -28.71 | 7.4061 | 17.743 | 24.278 | -128.3522 | 1.91543 | 1 |
| 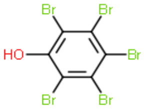 | 0 | 2849 | C6HBr5O | -5.595 | 13 | 0 | -1.0392 | -4.4374 | -15.9 | 1.5779 | 10.566 | 10.666 | -57.0957 | 1.11558 | 1 |
| 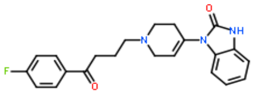 | 0 | 340 | 450 | -5.588 | 50 | 5 | -6.4392 | -5.7863 | -26.31 | 12.7 | 17.72 | 25.731 | -114.4174 | 1.52727 | 1 |
| 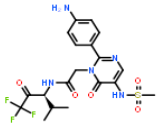 | 0 | 2883 | TFI | -5.585 | 55 | 7 | -4.7427 | -6.6182 | -26.89 | 4.992 | 19.28 | 24.372 | -120.6536 | 1.69577 | 1 |
| 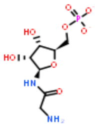 | 0 | 1983 | ChEBI | -5.585 | 31 | 8 | -11.588 | -2.3493 | -15.86 | 1.6897 | 22.087 | 28.463 | -112.5063 | 1.21376 | 1 |
| 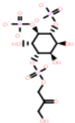 | 0 | 1789 | C9H19O17P3 | -5.585 | 43 | 14 | -16.968 | -2.7523 | -23.09 | 0 | 30.871 | 46.559 | -129.2914 | 1.66783 | 1 |
| 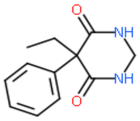 | 0 | 675 | 794 | -5.585 | 30 | 2 | -2.0058 | -3.9924 | -17.45 | 0.5951 | 12.9 | 12.698 | -78.05248 | 1.06069 | 1 |

| | | | | | | | | | | | | | | | |
|---|---|---|---|---|---|---|---|---|---|---|---|---|---|---|---|
| 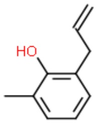 | 0 | 2978 | C10H12O | -5.573 | 23 | 2 | -1.0647 | -4.1517 | -16.33 | 0.6272 | 10.067 | 10.764 | -51.8125 | 0.8498 | 1 |
| 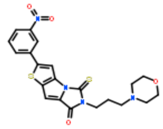 | 0 | 3170 | 703 | -5.57 | 51 | 5 | -3.4357 | -6.5536 | -26.19 | 7.0505 | 16.566 | 21.28 | -150.3296 | 1.66966 | 1 |
| 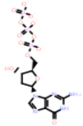 | 0 | 1934 | C10H16N5O13P3 | -5.567 | 43 | 9 | -13.976 | -3.3965 | -28.89 | 0 | 29.571 | 50.57 | -136.6832 | 1.46382 | 1 |
| 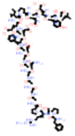 | 0 | 1 | C98H138N24O33 | -5.545 | 287 | 71 | -14.479 | -12.136 | -64.81 | 48.336 | 43.946 | -0.802 | -287.1651 | 7.26863 | 1 |
| 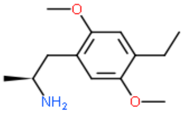 | 0 | 1270 | 1467 | -5.535 | 37 | 4 | -4.645 | -5.0723 | -18.1 | 3.1511 | 15.57 | 17.8 | -98.33334 | 1.42984 | 1 |
| 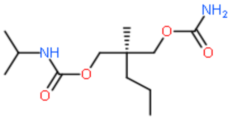 | 0 | 286 | 395 | -5.521 | 42 | 8 | -7.9968 | -5.0808 | -14.43 | 6.38 | 16.842 | 17.092 | -94.3708 | 1.28768 | 1 |
| 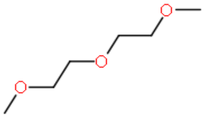 | 0 | 2633 | C6H14O3 | -5.476 | 23 | 6 | -2.7114 | -3.3678 | -9.09 | 0 | 8.9983 | 3.6085 | 8.332113 | 0.67836 | 1 |
| 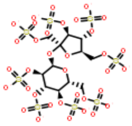 | 0 | 1671 | C12H22O35S8 | -5.464 | 69 | 21 | -13.978 | -3.0216 | -32.8 | 6.6373 | 44.713 | 22.138 | -91.6173 | 2.30564 | 1 |
| 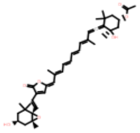 | 0 | 2696 | C39H50O7 | -5.458 | 96 | 4 | -4.6351 | -11.164 | -34.6 | 6.3595 | 25.711 | 34.624 | -153.9998 | 2.71355 | 1 |
| 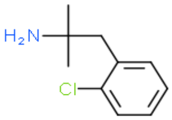 | 0 | 1323 | 1527 | -5.45 | 26 | 3 | -1.8379 | -3.3184 | -17.09 | 0.504 | 11.825 | 11.424 | -28.68912 | 0.78658 | 1 |
| 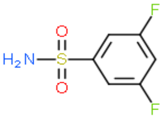 | 0 | 1845 | C6H5F2NO2S | -5.445 | 17 | 2 | -0.8476 | -2.3292 | -14.03 | 0.2272 | 8.8764 | 6.5105 | -18.97304 | 0.62649 | 1 |

| | | | | | | | | | | | | | | | |
|---|---|---|---|---|---|---|---|---|---|---|---|---|---|---|---|
| 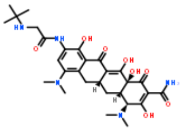 | 0 | 447 | m | -5.436 | 81 | 5 | -3.4258 | -7.9208 | -34.8 | 0 | 27.202 | 30.21 | -146.733 | 2.46405 | 1 |
| 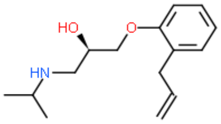 | 0 | 746 | 866 | -5.432 | 41 | 8 | -4.6895 | -5.4595 | -22.85 | 3.6413 | 15.835 | 21.336 | -106.16 | 1.5747 | 1 |
| 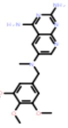 | 0 | 2618 | DTM | -5.428 | 49 | 2 | -5.9725 | -6.7537 | -24.63 | 2.6263 | 26.263 | 24.798 | -113.3478 | 1.62177 | 1 |
| 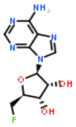 | 0 | 3357 | C10H12FN5O3 | -5.424 | 31 | 4 | -10.412 | -3.8307 | -7.453 | 4.1675 | 21.709 | 14.781 | -70.71375 | 1.12088 | 1 |
| 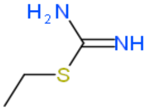 | 0 | 1981 | C3H8N2S | -5.422 | 14 | 1 | -4.7132 | -1.1508 | -8.778 | 3.6893 | 11.278 | 7.2418 | -10.72693 | 0.4341 | 1 |
| 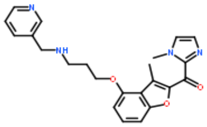 | 0 | 2751 | R64 | -5.416 | 54 | 6 | -2.7106 | -7.8768 | -31.63 | 7.7244 | 20.512 | 22.313 | -153.4385 | 1.70868 | 1 |
| 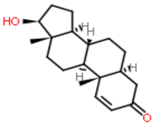 | 0 | 1284 | 1481 | -5.411 | 49 | 1 | -4.9665 | -4.4881 | -13.82 | 1.9887 | 13.434 | 18.35 | -81.09465 | 1.1068 | 1 |
| 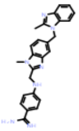 | 0 | 2093 | C25H25N7 | -5.41 | 57 | 4 | -5.0878 | -7.9243 | -28.57 | 7.1421 | 23.214 | 26.133 | -142.4049 | 1.94168 | 1 |
| 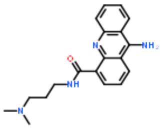 | 0 | 2545 | C19H22N4O | -5.4 | 46 | 4 | -2.6358 | -6.4466 | -21.95 | 2.1081 | 17.581 | 16.19 | -127.13 | 1.41183 | 1 |
| 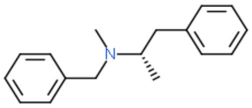 | 0 | 745 | C17H21N | -5.397 | 39 | 5 | -0.8892 | -5.7158 | -21.81 | 4.0254 | 9.5189 | 15.057 | -121.9355 | 1.39782 | 1 |
| 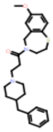 | 0 | 2627 | K21 | -5.386 | 62 | 6 | -1.7164 | -7.3894 | -31 | 8.8073 | 18.382 | 18.54 | -142.4341 | 1.89815 | 1 |

| | | | | | | | | | | | | | | | |
|---|---|---|---|---|---|---|---|---|---|---|---|---|---|---|---|
| 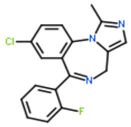 | 0 | 569 | 683 | -5.383 | 36 | 0 | -2.9224 | -6.1567 | -20.17 | 1.3677 | 14.551 | 22.076 | -135.2438 | 1.39115 | 1 |
| 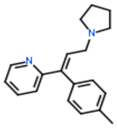 | 0 | 317 | 5282443 | -5.379 | 43 | 2 | -1.538 | -6.6351 | -22.57 | 1.7442 | 14.43 | 18.877 | -112.4105 | 1.40835 | 1 |
| 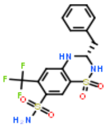 | 0 | 326 | 436 | -5.379 | 41 | 5 | -1.9601 | -4.7961 | -28.41 | 4.8166 | 16.751 | 19.326 | -129.6871 | 1.84073 | 1 |
| 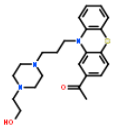 | 0 | 935 | C23H29N3O2S | -5.378 | 58 | 7 | -6.9863 | -6.5907 | -22.95 | 8.0682 | 19.802 | 23.902 | -134.9186 | 1.66798 | 1 |
| 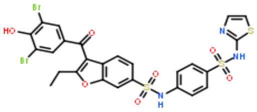 | 0 | 2988 | C26H19Br2N3O7S3 | -5.339 | 60 | 7 | -4.0695 | -6.3009 | -37.88 | 8.4632 | 24.662 | 29.215 | -161.2255 | 2.21302 | 1 |
| 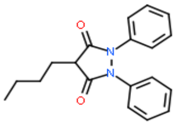 | 0 | 693 | 812 | -5.332 | 43 | 5 | -3.0388 | -5.7308 | -21.03 | 1.1547 | 14.851 | 18.778 | -112.0123 | 1.65428 | 1 |
| 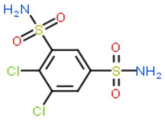 | 0 | 1015 | 1144 | -5.315 | 22 | 4 | -2.668 | -2.4842 | -19.24 | 0 | 13.915 | 14.398 | -79.91603 | 1.3037 | 1 |
| 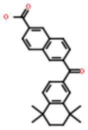 | 0 | 2453 | C26H26O3 | -5.308 | 54 | 1 | -2.0454 | -6.3212 | -26.93 | 3.7188 | 19.963 | 20.051 | -144.6694 | 1.76236 | 1 |
| 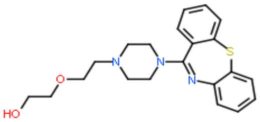 | 0 | 1094 | 1224 | -5.305 | 52 | 7 | -5.972 | -6.9741 | -24.98 | 5.1829 | 23.202 | 22.335 | -116.6357 | 1.73972 | 1 |
| 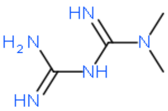 | 0 | 224 | C4H11N5 | -5.3 | 20 | 3 | -3.8946 | -1.7336 | -13.01 | 0 | 10.028 | 14.253 | -19.04641 | 0.57241 | 1 |
| 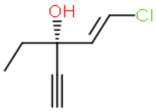 | 0 | 84 | C7H9ClO | -5.298 | 18 | 3 | -3.0949 | -4.0001 | -13.44 | 1.0292 | 9.9085 | 13.343 | -50.38908 | 0.85345 | 1 |

| | | | | | | | | | | | | | | | |
|---|---|---|---|---|---|---|---|---|---|---|---|---|---|---|---|
| 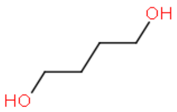 | 0 | 1719 | C4H10O2 | -5.298 | 16 | 5 | -5.2571 | -2.1858 | -8.847 | 1.9693 | 13.077 | 6.5531 | -33.3371 | 0.5781 | 1 |
| 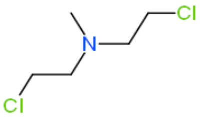 | 0 | 768 | 888 | -5.295 | 19 | 4 | 0 | -3.5977 | -14.05 | 0.2466 | 5.1385 | 7.2202 | -9.121141 | 0.57907 | 1 |
| 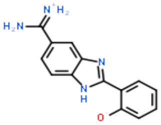 | 0 | 3292 | CR4 | -5.274 | 31 | 0 | -7.4025 | -4.0043 | -19.4 | 1.4375 | 18.419 | 31.601 | -81.90596 | 1.21365 | 1 |
| 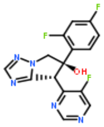 | 0 | 469 | 582 | -5.267 | 39 | 6 | -4.6792 | -5.4762 | -20.71 | 5.213 | 18.381 | 15.82 | -90.08448 | 1.02675 | 1 |
| 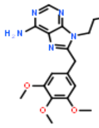 | 0 | 2465 | PU3 | -5.26 | 52 | 5 | -3.3483 | -6.4113 | -25.52 | 3.8497 | 18.87 | 20.556 | -104.5684 | 1.40576 | 1 |
| 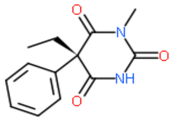 | 0 | 729 | 849 | -5.257 | 32 | 2 | -4.7999 | -3.561 | -15.65 | 3.2775 | 14.159 | 16.877 | -79.1808 | 1.1054 | 1 |
| 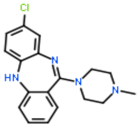 | 0 | 256 | m | -5.255 | 42 | 1 | 0 | -5.9582 | -23.49 | 1.2708 | 12.108 | 17.856 | -84.72987 | 1.10617 | 1 |
| 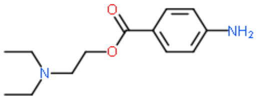 | 0 | 605 | 721 | -5.253 | 37 | 5 | -4.4215 | -5.4152 | -22.2 | 2.2764 | 15.488 | 23.905 | -80.63998 | 1.43874 | 1 |
| 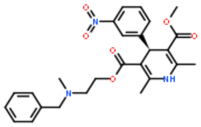 | 0 | 509 | 622 | -5.247 | 64 | 7 | -5.8553 | -7.6881 | -31.72 | 11.689 | 21.832 | 28.565 | -67.27823 | 1.73075 | 1 |
| 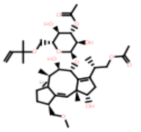 | 0 | 1560 | C36H56O12 | -5.243 | 104 | 16 | -6.2907 | -9.0451 | -35.38 | 11.32 | 26.172 | 24.387 | -188.5762 | 2.62431 | 1 |
| 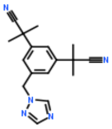 | 0 | 1087 | C17H19N5 | -5.236 | 41 | 4 | -4.1864 | -5.2705 | -17.56 | 0.8373 | 17 | 16.352 | -78.04726 | 1.18757 | 1 |

| | | | | | | | | | | | | | | | |
|---|---|---|---|---|---|---|---|---|---|---|---|---|---|---|---|
| 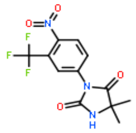 | 0 | 552 | 665 | -5.208 | 32 | 2 | -1.9271 | -4.428 | -21.71 | 1.4166 | 13.646 | 18.275 | -106.784 | 1.3221 | 1 |
| 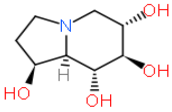 | 0 | 1593 | C8H15NO4 | -5.191 | 28 | 4 | -6.6636 | -2.7811 | -12.95 | 3.3562 | 19.539 | 11.238 | -86.153 | 0.9189 | 1 |
| 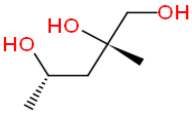 | 0 | 1654 | C6H14O3 | -5.185 | 23 | 6 | -6.9447 | -2.7861 | -10.14 | 5.8942 | 16.229 | 7.6976 | -52.31356 | 0.75782 | 1 |
| 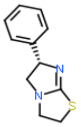 | 0 | 728 | 848 | -5.175 | 26 | 1 | -1.2066 | -3.5367 | -15.58 | 0.4897 | 11.065 | 10.052 | -33.22885 | 0.951 | 1 |
| 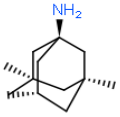 | 0 | 917 | 1043 | -5.172 | 34 | 1 | -1.7348 | -3.156 | -13.11 | 0.4148 | 10.54 | 8.6173 | -27.25051 | 0.75458 | 1 |
| 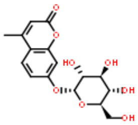 | 0 | 2360 | C16H18O8 | -5.139 | 42 | 6 | -8.508 | -4.283 | -20.65 | 4.7333 | 19.688 | 28.328 | -123.651 | 1.5874 | 1 |
| 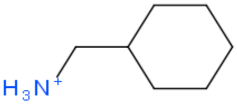 | 0 | 2171 | C7H16N | -5.136 | 24 | 1 | -2.7529 | -2.6187 | -10.99 | 0.2095 | 8.9747 | 10.786 | -25.85205 | 0.57361 | 1 |
| 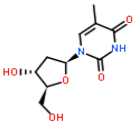 | 0 | 1132 | 1265 | -5.134 | 31 | 4 | -4.7682 | -3.0574 | -16.85 | 1.6557 | 19.468 | 11.861 | -94.57419 | 1.31119 | 1 |
| 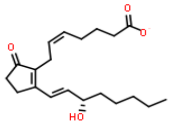 | 0 | 2046 | C20H30O4 | -5.128 | 53 | 12 | -10.252 | -5.7938 | -22.39 | 9.6968 | 22.328 | 26.232 | -108.5683 | 1.65706 | 1 |
| 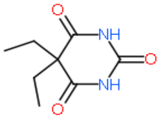 | 0 | 1286 | 1483 | -5.12 | 25 | 2 | -1.9623 | -3.0967 | -18.48 | 0.6498 | 12.616 | 14.16 | -77.02961 | 0.86934 | 1 |
| 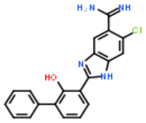 | 0 | 2105 | C20H15ClN4O | -5.114 | 41 | 1 | -4.0141 | -5.7989 | -25.61 | 4.159 | 19.989 | 24.929 | -116.7559 | 1.71237 | 1 |

| | | | | | | | | | | | | | | | |
|---|---|---|---|---|---|---|---|---|---|---|---|---|---|---|---|
| 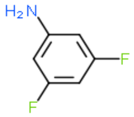 | 0 | 2919 | $C_6H_5F_2N$ | -5.1 | 14 | 0 | -0.8144 | -2.6928 | -10.79 | 0.3993 | 6.635 | 6.5775 | -16.4016 | 0.39432 | 1 |
| 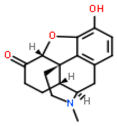 | 0 | 221 | 327 | -5.098 | 40 | 0 | -3.7431 | -3.6007 | -17.74 | 0.651 | 13.373 | 21.37 | -96.68421 | 1.11036 | 1 |
| 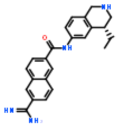 | 0 | 2279 | 497 | -5.098 | 52 | 1 | -3.5208 | -6.0818 | -24.44 | 2.1319 | 20.368 | 22.984 | -139.4018 | 1.61422 | 1 |
| 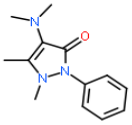 | 0 | 1231 | 1424 | -5.093 | 34 | 1 | -2.6416 | -5.304 | -19.6 | 3.5584 | 11.475 | 20.31 | -96.33316 | 1.39231 | 1 |
| 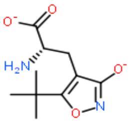 | 0 | 2996 | $C_{10}H_{15}N_2O_4$ | -5.092 | 30 | 5 | -9.7665 | -3.0381 | -16.15 | 0 | 19.044 | 31.087 | -82.17109 | 1.12202 | 1 |
| 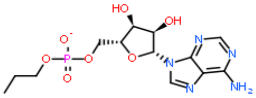 | 0 | 2911 | $C_{13}H_{20}N_5O_7P$ | -5.087 | 45 | 9 | -12.887 | -4.3834 | -21.88 | 2.7817 | 31.742 | 32.509 | -113.3662 | 1.65318 | 1 |
| 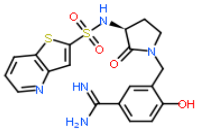 | 0 | 2456 | $C_{19}H_{19}N_5O_4S_2$ | -5.082 | 49 | 5 | -6.2785 | -4.4401 | -30.07 | 8.4602 | 22.473 | 28.654 | -147.9633 | 2.04531 | 1 |
| 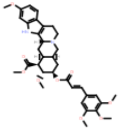 | 0 | 1050 | 1180 | -5.079 | 88 | 3 | -4.175 | -9.7693 | -36.03 | 5.7265 | 29.172 | 31.739 | -179.6681 | 2.5976 | 1 |
| 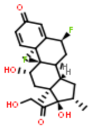 | 0 | 118 | $C_{22}H_{28}F_2O_5$ | -5.077 | 57 | 5 | -7.1027 | -4.5483 | -14.74 | 3.9025 | 19.473 | 16.258 | -85.67089 | 1.22996 | 1 |
| 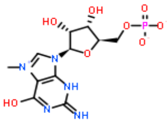 | 0 | 3245 | $C_{11}H_{17}N_5O_8P$ | -5.064 | 40 | 7 | -9.3957 | -2.9316 | -20.46 | 0 | 28.192 | 23.921 | -67.50369 | 1.20667 | 1 |
| 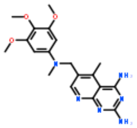 | 0 | 1862 | $CO_4$ | -5.05 | 52 | 2 | -5.4385 | -7.2797 | -25.76 | 2.8651 | 25.818 | 25.857 | -131.9626 | 1.82561 | 1 |

| | | | | | | | | | | | | | | | |
|---|---|---|---|---|---|---|---|---|---|---|---|---|---|---|---|
| 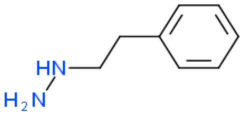 | 0 | 663 | 780 | -5.046 | 22 | 4 | -2.3783 | -2.6319 | -15.36 | 0.8265 | 12.56 | 8.7537 | -25.81753 | 0.67271 | 1 |
| 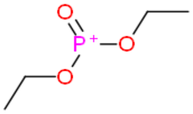 | 0 | 2516 | C4H10O3P | -5.035 | 19 | 4 | -2.5413 | -3.1317 | -12.65 | 0.0488 | 11.045 | 8.2275 | -46.60542 | 0.76485 | 1 |
| 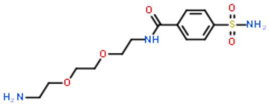 | 0 | 2264 | C13H21N3O5S | -5.033 | 43 | 11 | -6.6876 | -5.0812 | -22.96 | 3.7431 | 24.274 | 16.972 | -117.3078 | 1.65209 | 1 |
| 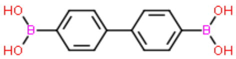 | 0 | 2350 | BDB | -5.023 | 30 | 4 | -5.6242 | -3.8088 | -18.96 | 1.5687 | 20.661 | 17.997 | -91.39601 | 1.48996 | 1 |
| 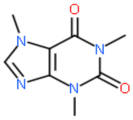 | 0 | 96 | 201 | -5.023 | 24 | 0 | -3.0787 | -3.6306 | -14.4 | 0.4324 | 12.794 | 14.771 | -85.57136 | 1.20111 | 1 |
| 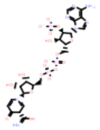 | 0 | 1536 | C21H29N7O18P3 | -5.021 | 74 | 17 | -16.764 | -4.8003 | -40.93 | 13.605 | 43.036 | 46.821 | -156.4589 | 2.18832 | 1 |
| 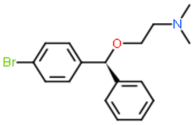 | 0 | 1106 | 1237 | -5.019 | 40 | 6 | -1.2678 | -6.8933 | -21.03 | 1.7726 | 13.498 | 13.717 | -88.29388 | 1.3178 | 1 |
| 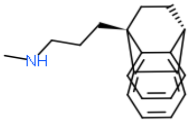 | 0 | 812 | 934 | -5.015 | 44 | 4 | -1.9412 | -6.3258 | -20.93 | 3.1666 | 11.683 | 18.181 | -113.5281 | 1.41652 | 1 |
| 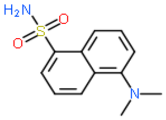 | 0 | 2568 | MNS | -5.011 | 31 | 2 | -1.7345 | -3.5095 | -17.21 | 1.5087 | 9.9919 | 14.155 | -75.78282 | 0.95958 | 1 |
| 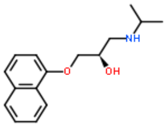 | 0 | 2999 | C16H21NO2 | -5.006 | 40 | 6 | -5.6719 | -5.7993 | -14.23 | 4.8606 | 13.294 | 16.628 | -76.96887 | 1.55586 | 1 |
| 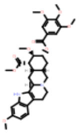 | 0 | 101 | C33H40N2O9 | -5.006 | 84 | 3 | -2.3222 | -10.108 | -34.87 | 7.0429 | 27.815 | 23.667 | -165.4328 | 2.58221 | 1 |

| | | | | | | | | | | | | | | | | |
|---|---|---|---|---|---|---|---|---|---|---|---|---|---|---|---|---|
| 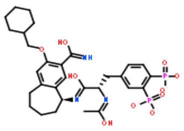 | 0 | 1607 | C30H41N3O10P2 | -5.005 | 82 | 12 | -8.413 | -5.493 | -24.22 | 9.4634 | 33.084 | 9.8384 | -131.5697 | 2.47253 | 1 |
| 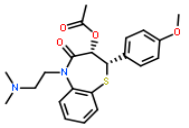 | 0 | 236 | C22H26N2O4S | -5.005 | 55 | 5 | -4.5814 | -6.5325 | -25.5 | 4.8559 | 22.563 | 20.642 | -99.64291 | 1.9849 | 1 |
| 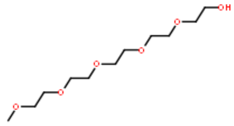 | 0 | 1802 | C11H24O6 | -5.003 | 41 | 15 | -9.2443 | -5.7803 | -17.02 | 5.5814 | 21.316 | 16.58 | -95.43995 | 1.61388 | 1 |
| 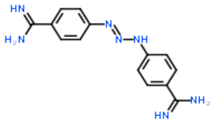 | 0 | 3259 | BRN | -5.002 | 36 | 0 | -6.6799 | -3.9287 | -22.29 | 6.3362 | 26.571 | 19.835 | -92.18545 | 1.48473 | 1 |
| 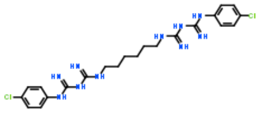 | 0 | 758 | [NO | -4.98 | 64 | 17 | -5.6456 | -5.4638 | -35.08 | 11.778 | 25.01 | 18.209 | -56.55159 | 2.06885 | 1 |
| 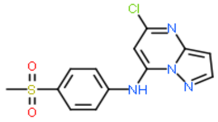 | 0 | 2659 | CT7 | -4.964 | 32 | 1 | -1.9286 | -4.7345 | -24.13 | 1.5178 | 16.089 | 20.662 | -102.3563 | 1.70717 | 1 |
| 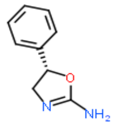 | 0 | 1293 | 1490 | -4.96 | 22 | 1 | -3.597 | -3.1505 | -12.44 | 0.3318 | 12.731 | 12.642 | -52.96018 | 0.73621 | 1 |
| 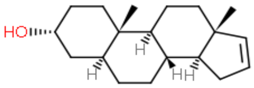 | 0 | 1661 | ATE | -4.954 | 50 | 1 | -2.3398 | -4.9022 | -15.97 | 1.0256 | 11.352 | 15.894 | -89.43263 | 1.07548 | 1 |
| 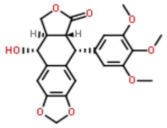 | 0 | 1049 | 1179 | -4.949 | 52 | 2 | -4.4296 | -6.1429 | -29.67 | 7.3683 | 25.082 | 23.651 | -145.4506 | 1.62031 | 1 |
| 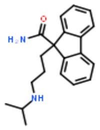 | 0 | 87 | 192 | -4.945 | 47 | 6 | -3.1701 | -5.6502 | -21.13 | 3.8923 | 14.522 | 16.914 | -78.71713 | 1.19076 | 1 |
| 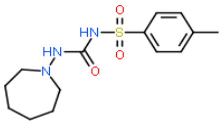 | 0 | 720 | 839 | -4.944 | 42 | 5 | -2.7124 | -5.4859 | -25.63 | 2.5405 | 14.616 | 23.849 | -98.36313 | 1.64545 | 1 |

| | | | | | | | | | | | | | | | |
|---|---|---|---|---|---|---|---|---|---|---|---|---|---|---|---|
| 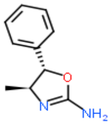 | 0 | 1253 | 1447 | -4.931 | 25 | 1 | -3.3169 | -3.4885 | -14.1 | 0.7151 | 12.893 | 13.937 | -60.57433 | 0.85323 | 1 |
| 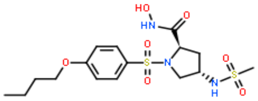 | 0 | 2106 | C16H25N3O7S2 | -4.929 | 53 | 10 | -2.7446 | -5.8527 | -30.33 | 1.3404 | 18.876 | 23.195 | -124.0482 | 1.80706 | 1 |
| 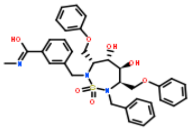 | 0 | 2765 | C34H37N3O7S | -4.921 | 82 | 13 | -4.5105 | -7.0175 | -36.14 | 14.439 | 19.62 | 24.479 | -142.9388 | 2.10389 | 1 |
| 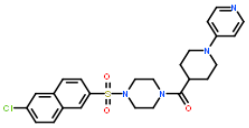 | 0 | 1613 | C25H27ClN4O3S | -4.915 | 61 | 4 | -1.1353 | -8.4746 | -33.36 | 5.9366 | 17.375 | 27.268 | -145.8551 | 2.22462 | 1 |
| 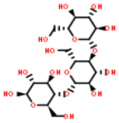 | 0 | 1483 | C18H32O16 | -4.911 | 66 | 18 | -18.429 | -4.3818 | -19.43 | 27.216 | 32.821 | 18.691 | -145.073 | 1.77193 | 1 |
| 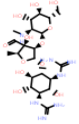 | 0 | 954 | C21H39N7O12 | -4.899 | 79 | 18 | -10.239 | -4.3088 | -28.45 | 9.8049 | 27.32 | 22.991 | -179.7696 | 2.38806 | 1 |
| 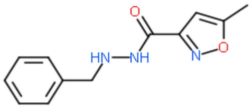 | 0 | 1116 | 1247 | -4.894 | 30 | 3 | -3.1058 | -4.3301 | -20.09 | 0.0231 | 17.081 | 17.166 | -85.68524 | 1.22302 | 1 |
| 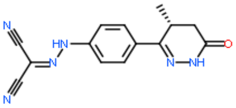 | 0 | 801 | 922 | -4.865 | 33 | 1 | -4.2788 | -3.629 | -21.01 | 3.5876 | 14.97 | 23.363 | -44.48758 | 1.27302 | 1 |
| 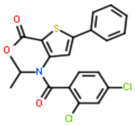 | 0 | 3059 | C21H17Cl2NO3S | -4.862 | 44 | 4 | -1.4982 | -6.3739 | -21.97 | 7.3067 | 14.34 | 11.554 | -94.97297 | 1.615 | 1 |
| 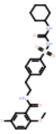 | 0 | 892 | 1016 | -4.857 | 61 | 8 | -3.6484 | -6.2272 | -29.18 | 9.3095 | 19.384 | 19.399 | -157.4655 | 2.11768 | 1 |
| 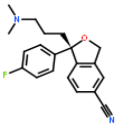 | 0 | 1045 | C20H21FN2O | -4.852 | 45 | 5 | -2.879 | -5.9812 | -22.51 | 5.3371 | 15.163 | 17.233 | -98.94203 | 1.43704 | 1 |

| | | | | | | | | | | | | | | | |
|---|---|---|---|---|---|---|---|---|---|---|---|---|---|---|---|
| 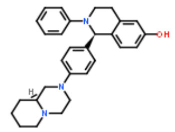 | 0 | 3439 | C29H33N3O | -4.85 | 66 | 2 | -4.7249 | -8.2539 | -25.64 | 8.3006 | 19.864 | 26.048 | -166.7085 | 2.07948 | 1 |
| 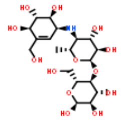 | 0 | 2589 | C19H33NO13 | -4.845 | 66 | 16 | -14.693 | -4.3678 | -22.13 | 11.957 | 36.867 | 19.634 | -139.3674 | 1.87595 | 1 |
| 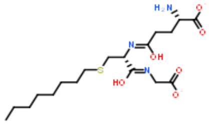 | 0 | 2724 | C18H33N3O6S | -4.844 | 59 | 20 | -7.8608 | -5.8558 | -27.57 | 1.7834 | 25.051 | 22.245 | -150.5536 | 1.60448 | 1 |
| 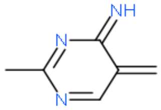 | 0 | 1566 | C6H7N3 | -4.84 | 16 | 0 | -3.1862 | -2.317 | -14.23 | 0 | 12.749 | 14.312 | -41.36013 | 0.61982 | 1 |
| 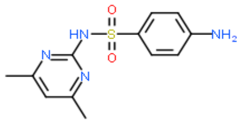 | 0 | 1378 | 1582 | -4.84 | 33 | 3 | -0.8181 | -4.7203 | -24.19 | 0.8196 | 13.971 | 17.898 | -99.65608 | 1.25702 | 1 |
| 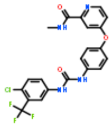 | 0 | 289 | 398 | -4.837 | 48 | 4 | -1.3551 | -6.507 | -26.62 | 0 | 16.141 | 22.833 | -102.0514 | 2.04742 | 1 |
| 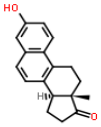 | 0 | 3178 | EQU | -4.837 | 38 | 0 | -1.9929 | -4.8159 | -18.06 | 1.7937 | 15.181 | 13.677 | -122.5612 | 1.5226 | 1 |
| 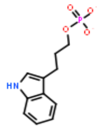 | 0 | 2853 | IPL | -4.829 | 29 | 5 | -6.9265 | -3.6109 | -17.82 | 3.666 | 15.703 | 24.182 | -120.3521 | 1.25846 | 1 |
| 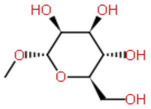 | 0 | 1743 | C7H14O6 | -4.817 | 27 | 6 | -5.9164 | -2.1934 | -11.02 | 0 | 14.415 | 12.466 | -57.01454 | 0.86647 | 1 |
| 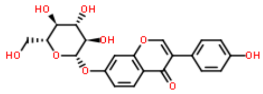 | 0 | 1873 | Daidzin | -4.812 | 50 | 6 | -7.1639 | -4.7401 | -24.54 | 6.0977 | 25.451 | 22.242 | -99.64051 | 1.75729 | 1 |
| 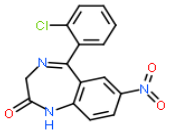 | 0 | 940 | 1068 | -4.802 | 32 | 1 | -0.5603 | -4.4802 | -21.46 | 1.5267 | 11.523 | 16.539 | -107.7091 | 1.41997 | 1 |

| | | | | | | | | | | | | | | | |
|---|---|---|---|---|---|---|---|---|---|---|---|---|---|---|---|
| 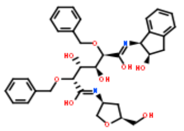 | 0 | 1659 | C34H40N2O9 | -4.801 | 85 | 20 | -5.7276 | -7.7297 | -35.44 | 6.0008 | 23.335 | 26.078 | -165.439 | 2.42815 | 1 |
| 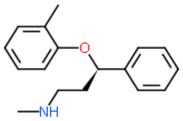 | 0 | 183 | 54841 | -4.796 | 40 | 5 | -1.8063 | -5.807 | -19.86 | 2.7052 | 11.474 | 15.598 | -97.28283 | 1.46962 | 1 |
| 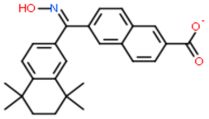 | 0 | 2002 | C26H27NO3 | -4.78 | 56 | 2 | -3.2225 | -6.7142 | -26.65 | 4.1217 | 22.51 | 21.156 | -163.2837 | 1.91158 | 1 |
| 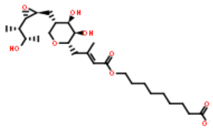 | 0 | 300 | m | -4.776 | 78 | 18 | -9.5936 | -7.6193 | -22.18 | 8.9602 | 31.093 | 11.03 | -183.9907 | 2.99242 | 1 |
| 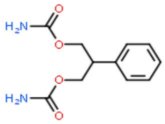 | 0 | 827 | 949 | -4.77 | 31 | 5 | -6.9069 | -3.2356 | -12.86 | 4.0343 | 18.513 | 12.765 | -88.83838 | 1.08777 | 1 |
| 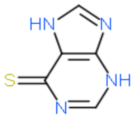 | 0 | 908 | 1033 | -4.768 | 14 | 0 | -2.9923 | -1.5064 | -12.03 | 0.2966 | 11.503 | 10.655 | -41.40916 | 0.61144 | 1 |
| 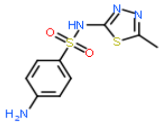 | 0 | 463 | 576 | -4.766 | 27 | 3 | 0 | -4.27 | -22.47 | 0 | 13.206 | 13.431 | -94.63521 | 1.19633 | 1 |
| 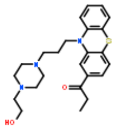 | 0 | 913 | C24H31N3O2S | -4.757 | 61 | 8 | -6.4225 | -7.2542 | -26.17 | 14.269 | 20.754 | 20.603 | -135.3062 | 1.85626 | 1 |
| 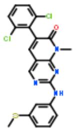 | 0 | 2294 | C21H16Cl2N4OS | -4.744 | 45 | 0 | -1.2556 | -7.137 | -26.9 | 5.414 | 16.053 | 22.465 | -98.39832 | 1.53987 | 1 |
| 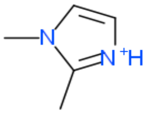 | 0 | 3426 | C5H9N2 | -4.74 | 16 | 0 | -1.9914 | -2.6855 | -11.16 | 0.0726 | 5.6814 | 13.559 | -30.02831 | 0.67319 | 1 |
| 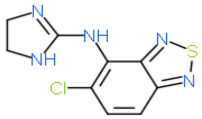 | 0 | 583 | 697 | -4.737 | 24 | 1 | -2.6007 | -3.9794 | -17.26 | 0 | 13.72 | 16.48 | -51.46819 | 0.80337 | 1 |

| | | | | | | | | | | | | | | | |
|---|---|---|---|---|---|---|---|---|---|---|---|---|---|---|---|
| 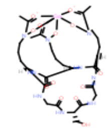 | 0 | 3103 | C28H44GaN9O13 | -4.732 | 95 | 2 | -6.3423 | -7.4129 | -20.76 | 15.893 | 25.354 | 11.044 | -137.3001 | 2.09618 | 1 |
| 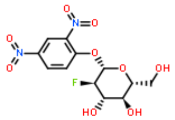 | 0 | 2378 | NFG | -4.723 | 37 | 7 | -3.8855 | -3.5038 | -23.89 | 7.2157 | 15.382 | 17.084 | -121.894 | 1.62619 | 1 |
| 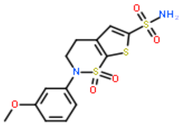 | 0 | 2327 | AL1 | -4.723 | 37 | 3 | -1.3207 | -4.8817 | -23.32 | 3.119 | 15.193 | 15.369 | -118.226 | 1.82327 | 1 |
| 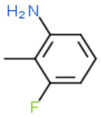 | 0 | 1750 | C7H8FN | -4.72 | 17 | 0 | -1.2223 | -3.4536 | -11.73 | 0.3732 | 8.5036 | 8.8122 | -36.91536 | 0.59287 | 1 |
| 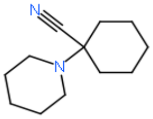 | 0 | 1335 | 1539 | -4.717 | 34 | 2 | -3.5331 | -4.3036 | -11.49 | 0.6856 | 11.556 | 12.595 | -82.14767 | 1.19539 | 1 |
| 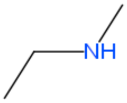 | 0 | 2132 | C3H9N | -4.695 | 13 | 1 | -2.1734 | -2.7007 | -8.095 | 0.0317 | 7.8058 | 6.4163 | -26.22399 | 0.30606 | 1 |
| 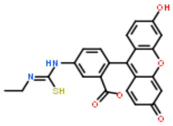 | 0 | 2188 | C23H18N2O5S | -4.687 | 48 | 4 | -5.2851 | -5.3935 | -31.89 | 10.425 | 25.718 | 24.62 | -116.8633 | 2.03376 | 1 |
| 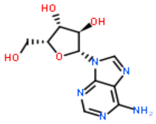 | 0 | 3188 | C10H13N5O4 | -4.68 | 32 | 5 | -6.35 | -3.9382 | -14.7 | 2.8301 | 22.416 | 10.935 | -80.71305 | 1.33469 | 1 |
| 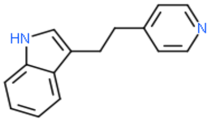 | 0 | 1717 | L12 | -4.68 | 31 | 3 | -2.0107 | -4.9165 | -22.24 | 1.098 | 13.58 | 20.147 | -111.0665 | 1.25008 | 1 |
| 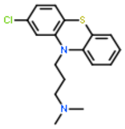 | 0 | 367 | 477 | -4.674 | 40 | 4 | -1.3376 | -6.1769 | -27.61 | 4.4892 | 12.628 | 24.078 | -119.5613 | 1.37765 | 1 |
| 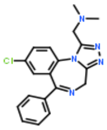 | 0 | 433 | 546 | -4.647 | 43 | 3 | -2.5114 | -6.2886 | -23.01 | 3.2939 | 16.185 | 19.715 | -131.7568 | 1.64032 | 1 |

| | | | | | | | | | | | | | | | |
|---|---|---|---|---|---|---|---|---|---|---|---|---|---|---|---|
| 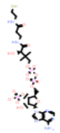 | 0 | 1756 | m | -4.637 | 80 | 21 | -15.866 | -5.2278 | -36.35 | 17.558 | 34.282 | 40.163 | -190.7657 | 2.52824 | 1 |
| 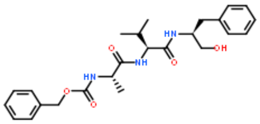 | 0 | 1663 | INT | -4.637 | 66 | 13 | -4.4976 | -7.3515 | -30.48 | 6.8188 | 21.044 | 21.936 | -104.9685 | 1.85074 | 1 |
| 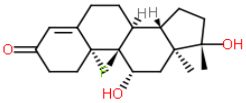 | 0 | 1055 | 1185 | -4.634 | 53 | 2 | -4.9319 | -4.3992 | -15.77 | 2.1597 | 15.938 | 18.352 | -87.06633 | 1.24081 | 1 |
| 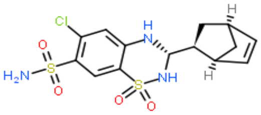 | 0 | 493 | C14H16ClN3O4S2 | -4.632 | 40 | 3 | -1.0374 | -4.4687 | -25.22 | 0 | 16.994 | 17.671 | -114.2946 | 1.87731 | 1 |
| 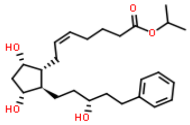 | 0 | 541 | C26H40O5 | -4.63 | 71 | 16 | -8.4385 | -6.1537 | -30.32 | 10.136 | 26.005 | 24.204 | -151.1269 | 2.56608 | 1 |
| 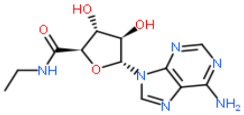 | 0 | 3360 | C12H16N6O4 | -4.619 | 38 | 5 | -6.0594 | -4.1163 | -14.46 | 1.0181 | 26.075 | 7.2185 | -83.19768 | 1.57922 | 1 |
| 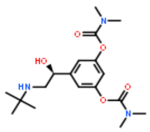 | 0 | 1216 | 1408 | -4.617 | 55 | 7 | -2.3819 | -7.2354 | -27.2 | 6.6694 | 17.729 | 18.127 | -107.8052 | 1.55915 | 1 |
| 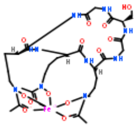 | 0 | 3228 | C28H44FeN9O13 | -4.605 | 95 | 2 | -3.215 | -6.5883 | -30.52 | 58.738 | 23.975 | -24.18 | -180.3088 | 2.33607 | 1 |
| 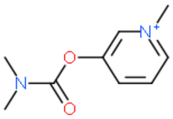 | 0 | 432 | 545 | -4.59 | 26 | 1 | -1.6926 | -4.6685 | -17.49 | 0.416 | 10.643 | 17.384 | -70.72376 | 1.15508 | 1 |
| 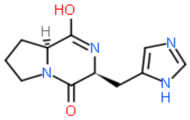 | 0 | 2150 | C11H14N4O2 | -4.582 | 31 | 3 | -5.2212 | -4.0865 | -14.91 | 2.4572 | 16.217 | 16.421 | -113.3375 | 1.23994 | 1 |
| 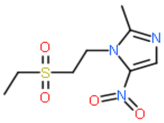 | 0 | 790 | 911 | -4.58 | 29 | 5 | 0 | -3.058 | -19.94 | 0.2497 | 8.8927 | 11.502 | -24.61743 | 0.89565 | 1 |

| | | | | | | | | | | | | | | | |
|---|---|---|---|---|---|---|---|---|---|---|---|---|---|---|---|
| 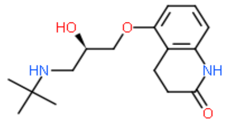 | 0 | 408 | 521 | -4.573 | 45 | 6 | -5.388 | -5.0905 | -22.13 | 5.9435 | 18.751 | 20.264 | -115.386 | 1.2764 | 1 |
| 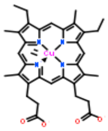 | 0 | 1941 | C35H39CuN4O4 | -4.57 | 81 | 8 | -7.3404 | -6.7694 | -23.98 | 12.994 | 22.871 | 19.343 | -132.9299 | 2.02461 | 1 |
| 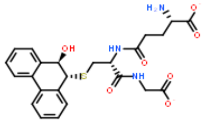 | 0 | 1611 | C24H27N3O7S | -4.568 | 60 | 13 | -11.036 | -5.6844 | -29.47 | 8.8236 | 26.583 | 35.763 | -180.1852 | 2.03138 | 1 |
| 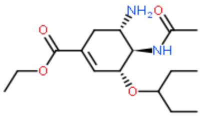 | 0 | 93 | 65028 | -4.555 | 50 | 7 | -2.9871 | -6.0819 | -25.92 | 3.2771 | 19.117 | 18.927 | -100.6256 | 1.60139 | 1 |
| 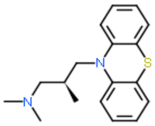 | 0 | 1115 | 1246 | -4.552 | 43 | 4 | 0 | -5.7622 | -21.39 | 0.8558 | 10.228 | 15.318 | -93.43131 | 1.26642 | 1 |
| 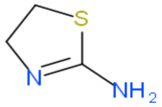 | 0 | 2076 | C3H6N2S | -4.549 | 12 | 0 | -2.9836 | -1.5372 | -8.82 | 0 | 8.53 | 9.7754 | -24.83923 | 0.53937 | 1 |
| 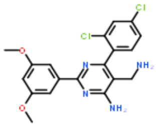 | 0 | 1766 | 5AP | -4.548 | 45 | 2 | -4.8886 | -6.3524 | -23.24 | 2.1667 | 23.406 | 23.129 | -119.9333 | 2.08567 | 1 |
| 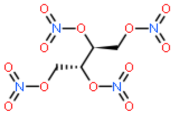 | 0 | 1406 | C4H6N4O12 | -4.545 | 26 | 11 | -2.2869 | -1.8555 | -22.11 | 8.1688 | 11.481 | 6.7913 | -90.22253 | 1.02403 | 1 |
| 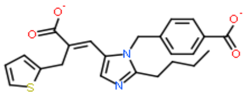 | 0 | 756 | C23H24N2O4S | -4.542 | 52 | 9 | -8.3709 | -5.8473 | -21.38 | 8.0461 | 20.183 | 25.102 | -148.4599 | 1.97941 | 1 |
| 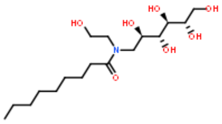 | 0 | 1734 | C17H35NO7 | -4.54 | 60 | 22 | -8.743 | -6.3961 | -24.35 | 2.6495 | 23.399 | 21.021 | -136.9688 | 1.79508 | 1 |
| 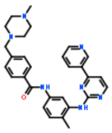 | 0 | 506 | 619 | -4.539 | 68 | 2 | -1.9524 | -8.9448 | -34.03 | 8.5521 | 19.67 | 29.298 | -158.4191 | 2.26698 | 1 |

| | | | | | | | | | | | | | | | |
|---|---|---|---|---|---|---|---|---|---|---|---|---|---|---|---|
| 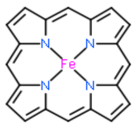 | 0 | 1496 | C20H12FeN4 | -4.538 | 37 | 0 | 0 | -5.493 | -22.77 | 1.6289 | 10.056 | 20.298 | -67.60841 | 1.06842 | 1 |
| 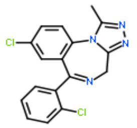 | 0 | 776 | 897 | -4.536 | 35 | 0 | -2.0253 | -5.8837 | -15.55 | 2.3015 | 14.06 | 12.358 | -93.8613 | 1.44839 | 1 |
| 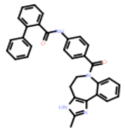 | 0 | 752 | 872 | -4.527 | 64 | 2 | -2.3376 | -7.9259 | -35.71 | 13.141 | 20.226 | 27.507 | -182.0497 | 2.01435 | 1 |
| 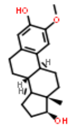 | 0 | 2083 | C19H26O3 | -4.512 | 48 | 1 | -4.0939 | -4.856 | -14.08 | 2.9748 | 13.261 | 16.442 | -90.70895 | 1.16106 | 1 |
| 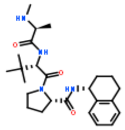 | 0 | 2351 | 998 | -4.503 | 70 | 8 | -5.0085 | -7.9395 | -28.33 | 2.2294 | 22.581 | 28.397 | -166.6768 | 1.76072 | 1 |
| 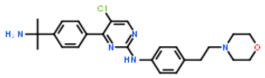 | 0 | 2221 | C25H30ClN5O | -4.493 | 62 | 5 | -4.6719 | -8.3438 | -26.51 | 4.2044 | 25.804 | 22.049 | -139.2565 | 2.03949 | 1 |
| 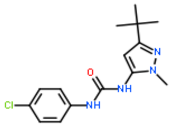 | 0 | 2021 | 446816 | -4.487 | 40 | 4 | -1.9174 | -5.7494 | -21.98 | 1.7725 | 15.907 | 16.366 | -89.98183 | 1.62377 | 1 |
| 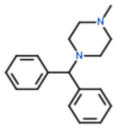 | 0 | 1046 | 1176 | -4.484 | 42 | 3 | 0 | -5.8985 | -21.9 | 1.4503 | 9.7255 | 17.272 | -78.56802 | 1.02248 | 1 |
| 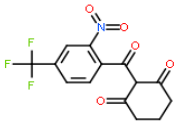 | 0 | 241 | 348 | -4.482 | 33 | 3 | -3.9552 | -3.965 | -20.57 | 5.1791 | 15.361 | 18.673 | -121.7082 | 1.18121 | 1 |
| 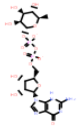 | 0 | 2275 | C16H25N5O15P2 | -4.479 | 61 | 13 | -16.594 | -3.8605 | -32.62 | 16.092 | 37.582 | 41.021 | -166.6828 | 2.07287 | 1 |
| 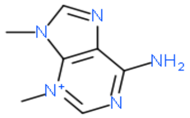 | 0 | 3371 | C7H10N5 | -4.478 | 22 | 0 | -1.6449 | -3.3012 | -13.1 | 0.3436 | 9.8779 | 11.188 | -46.9694 | 0.52213 | 1 |

| | | | | | | | | | | | | | | | |
|---|---|---|---|---|---|---|---|---|---|---|---|---|---|---|---|
| 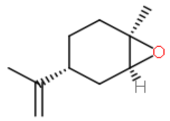 | 0 | 2623 | C10H16O | -4.453 | 27 | 1 | -1.3841 | -4.1144 | -14.28 | 0.2527 | 10.443 | 11.365 | -70.44257 | 0.9701 | 1 |
| 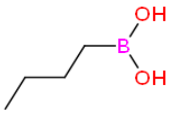 | 0 | 2384 | C4H11BO2 | -4.432 | 18 | 5 | -4.27 | -2.0717 | -9.044 | 0 | 10.688 | 8.7674 | -24.2199 | 0.71416 | 1 |
| 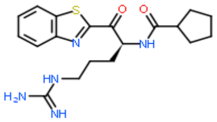 | 0 | 2932 | RWJ | -4.43 | 52 | 8 | -4.2517 | -6.7741 | -28.08 | 4.0638 | 24.699 | 19.977 | -95.16425 | 1.54983 | 1 |
| 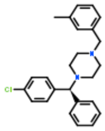 | 0 | 620 | 737 | -4.424 | 55 | 5 | 0 | -8.5413 | -28.55 | 3.067 | 15.552 | 20.223 | -133.7183 | 1.81137 | 1 |
| 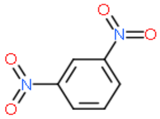 | 0 | 2399 | C6H4N2O4 | -4.406 | 16 | 2 | 0 | -2.0866 | -15.41 | 0.6376 | 6.8104 | 8.6502 | -49.09924 | 0.70485 | 1 |
| 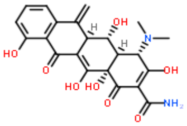 | 0 | 809 | C22H22N2O8 | -4.403 | 54 | 3 | -4.0768 | -5.2433 | -26.94 | 3.2748 | 23.294 | 22.922 | -125.738 | 1.91927 | 1 |
| 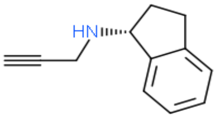 | 0 | 1191 | 1367 | -4.394 | 26 | 2 | -1.1967 | -4.8343 | -15.44 | 2.7772 | 10.319 | 10.155 | -59.04293 | 1.04067 | 1 |
| 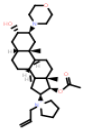 | 0 | 612 | C32H53N2O4 | -4.393 | 91 | 6 | -3.4689 | -7.508 | -22.32 | 0 | 16.08 | 24.361 | -118.5202 | 1.72238 | 1 |
| 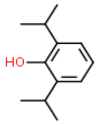 | 0 | 699 | 818 | -4.384 | 31 | 2 | -1.4705 | -4.8126 | -15.11 | 0.8467 | 10.883 | 11.813 | -79.96256 | 1.19717 | 1 |
| 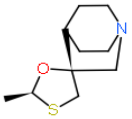 | 0 | 80 | C10H17NOS | -4.382 | 30 | 0 | -3.0294 | -4.5648 | -10.55 | 2.8668 | 9.667 | 12.002 | -67.73845 | 0.88208 | 1 |
| 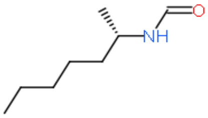 | 0 | 2750 | C8H17NO | -4.373 | 27 | 6 | -2.1558 | -2.9413 | -14.94 | 3.2902 | 9.3865 | 8.1539 | -11.14803 | 0.72012 | 1 |

| | | | | | | | | | | | | | | | |
|---|---|---|---|---|---|---|---|---|---|---|---|---|---|---|---|
| 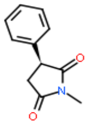 | 0 | 713 | 832 | -4.372 | 25 | 1 | -1.4006 | -3.9234 | -16.17 | 0.3935 | 10.925 | 13.552 | -78.19645 | 1.06502 | 1 |
| 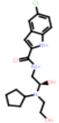 | 0 | 2965 | 288 | -4.358 | 49 | 8 | -6.5361 | -5.9968 | -22.1 | 3.5479 | 22.139 | 22.377 | -117.954 | 1.78851 | 1 |
| 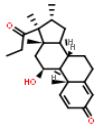 | 0 | 775 | C24H34O3 | -4.346 | 61 | 3 | -2.0611 | -5.3392 | -20.58 | 1.0407 | 12.612 | 19.915 | -84.95359 | 1.21575 | 1 |
| 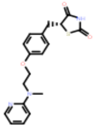 | 0 | 302 | C18H19N3O3S | -4.326 | 44 | 5 | -4.1588 | -5.8578 | -27.02 | 4.5287 | 21.282 | 23.424 | -132.0189 | 1.71825 | 1 |
| 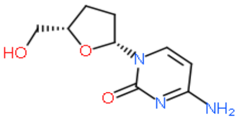 | 0 | 821 | 943 | -4.319 | 28 | 3 | -6.5818 | -2.9345 | -10.5 | 3.4251 | 17.268 | 12.104 | -67.71717 | 0.90306 | 1 |
| 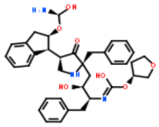 | 0 | 1601 | C36H43N3O7 | -4.318 | 89 | 16 | -2.4328 | -7.7123 | -32.37 | 3.078 | 23.013 | 16.157 | -131.4525 | 2.05149 | 1 |
| 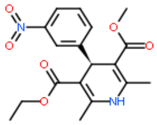 | 0 | 926 | 1054 | -4.317 | 46 | 3 | -2.7596 | -5.2832 | -26.84 | 4.5418 | 16.499 | 24.456 | -119.4528 | 1.56624 | 1 |
| 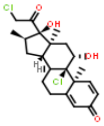 | 0 | 647 | C22H28Cl2O4 | -4.31 | 56 | 4 | -2.9081 | -5.0095 | -22.57 | 1.7726 | 15.981 | 20.493 | -76.57471 | 1.23345 | 1 |
| 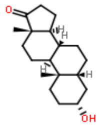 | 0 | 2557 | AE2 | -4.307 | 51 | 1 | -1.7541 | -4.7989 | -16.68 | 0.8142 | 13.559 | 13.279 | -78.79801 | 1.18757 | 1 |
| 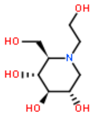 | 0 | 381 | 491 | -4.292 | 31 | 8 | -7.5477 | -2.1379 | -12.28 | 4.0138 | 17.575 | 12.315 | -49.97026 | 0.92583 | 1 |
| 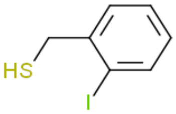 | 0 | 3279 | C7H7IS | -4.29 | 16 | 2 | -0.8938 | -3.4284 | -14.33 | 0.9313 | 9.086 | 9.1351 | -43.24621 | 0.89057 | 1 |

| | | | | | | | | | | | | | | | |
|---|---|---|---|---|---|---|---|---|---|---|---|---|---|---|---|
| 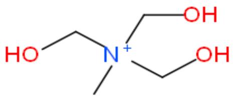 | 0 | 3224 | C4H12NO3 | -4.288 | 20 | 6 | -5.8817 | -1.8877 | -8.73 | 2.7388 | 10.538 | 11.274 | -26.02675 | 0.65442 | 1 |
| 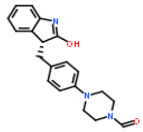 | 0 | 1817 | C20H21N3O2 | -4.277 | 46 | 4 | -3.8771 | -5.0363 | -19.07 | 1.5657 | 17.363 | 17.596 | -100.9678 | 1.46328 | 1 |
| 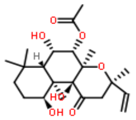 | 0 | 2312 | FOK | -4.275 | 63 | 5 | -2.6844 | -5.4553 | -21.78 | 1.847 | 17.959 | 15.715 | -100.3052 | 1.50013 | 1 |
| 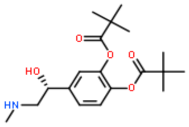 | 0 | 339 | 449 | -4.271 | 54 | 6 | -5.4068 | -6.3886 | -22.58 | 8.5771 | 19.765 | 19.244 | -95.05029 | 1.40755 | 1 |
| 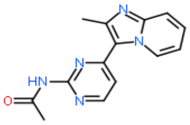 | 0 | 2267 | HDU | -4.269 | 33 | 0 | -3.0626 | -5.6057 | -19.01 | 3.2907 | 15.944 | 18.657 | -109.9756 | 1.44429 | 1 |
| 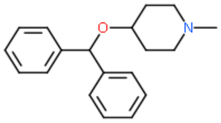 | 0 | 1017 | 1146 | -4.267 | 44 | 4 | -1.753 | -6.4504 | -25.59 | 2.2879 | 12.314 | 25.86 | -143.2102 | 1.62328 | 1 |
| 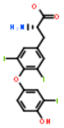 | 0 | 174 | 279 | -4.267 | 34 | 6 | -5.606 | -5.8792 | -20.77 | 2.9439 | 19.57 | 22.089 | -107.2709 | 1.88684 | 1 |
| 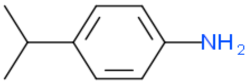 | 0 | 1872 | C9H13N | -4.266 | 23 | 1 | -2.7875 | -4.1184 | -9.694 | 1.0073 | 9.8535 | 10.069 | -52.3733 | 0.79273 | 1 |
| 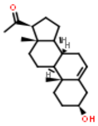 | 0 | 2495 | C21H32O2 | -4.262 | 55 | 2 | -4.1941 | -4.7477 | -15.13 | 1.8345 | 15.327 | 16.454 | -73.77078 | 1.17263 | 1 |
| 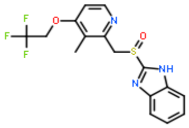 | 0 | 338 | 448 | -4.259 | 39 | 5 | -3.7411 | -6.2547 | -23.87 | 6.9779 | 17.822 | 19.267 | -75.09307 | 1.49983 | 1 |
| 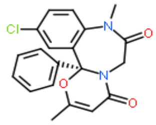 | 0 | 1381 | 1587 | -4.255 | 43 | 1 | -0.836 | -5.0034 | -19.42 | 8.4449 | 13.335 | 7.5161 | -72.6076 | 1.25051 | 1 |

| | | | | | | | | | | | | | | | |
|---|---|---|---|---|---|---|---|---|---|---|---|---|---|---|---|
| 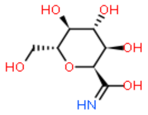 | 0 | 2434 | C7H13NO6 | -4.219 | 27 | 7 | -7.3227 | -1.7112 | -12.88 | 0 | 19.754 | 14.118 | -64.37309 | 0.94749 | 1 |
| 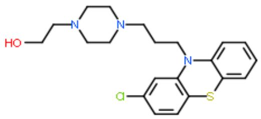 | 0 | 730 | 850 | -4.201 | 53 | 7 | -4.1356 | -7.1248 | -27.18 | 4.2721 | 21.006 | 23.786 | -142.5683 | 1.96951 | 1 |
| 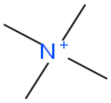 | 0 | 2782 | C4H12N | -4.2 | 17 | 0 | 0 | -2.616 | -8.185 | 0.053 | 3.2692 | 4.9307 | -4.257619 | 0.35461 | 1 |
| 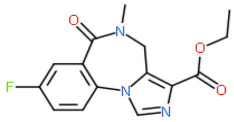 | 0 | 1075 | 1205 | -4.194 | 36 | 1 | -3.1059 | -5.6557 | -23.19 | 3.4854 | 18.349 | 21.505 | -115.4598 | 1.69137 | 1 |
| 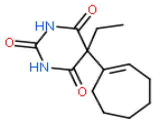 | 0 | 1182 | 1354 | -4.192 | 36 | 2 | -3.8468 | -3.6029 | -15.18 | 0 | 13.811 | 17.533 | -95.06176 | 1.17436 | 1 |
| 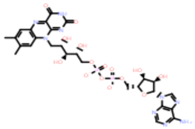 | 0 | 2830 | C27H33N9O15P2 | -4.191 | 84 | 18 | -20.016 | -4.4719 | -32.88 | 26.778 | 42.314 | 35.946 | -210.9621 | 2.59919 | 1 |
| 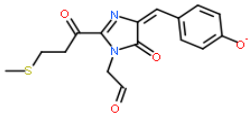 | 0 | 2488 | C16H15N2O4S | -4.189 | 38 | 6 | -5.3238 | -4.8737 | -25.03 | 4.1647 | 18.776 | 26.352 | -97.994 | 1.45095 | 1 |
| 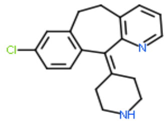 | 0 | 844 | 967 | -4.181 | 41 | 0 | -1.7623 | -5.2498 | -18.26 | 1.4373 | 12.043 | 18.43 | -83.11884 | 1.13651 | 1 |
| 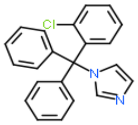 | 0 | 152 | 257 | -4.162 | 42 | 4 | -2.704 | -5.8229 | -22.32 | 6.579 | 13.344 | 19.013 | -85.17958 | 1.11042 | 1 |
| 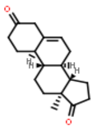 | 0 | 1249 | C18H24O2 | -4.161 | 44 | 0 | -1.9277 | -4.6118 | -13.7 | 1.3433 | 14.077 | 9.3095 | -117.6829 | 1.63151 | 1 |
| 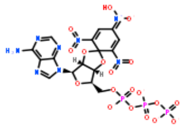 | 0 | 2254 | C16H17N8O19P3 | -4.142 | 59 | 11 | -12.501 | -3.7638 | -34.47 | 9.202 | 38.329 | 35.774 | -145.5589 | 2.4246 | 1 |

| | | | | | | | | | | | | | | | |
|---|---|---|---|---|---|---|---|---|---|---|---|---|---|---|---|
| 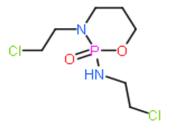 | 0 | 1051 | 1181 | -4.134 | 29 | 5 | -4.2626 | -4.6837 | -11.04 | 6.5274 | 11.133 | 8.4224 | -65.23422 | 1.17518 | 1 |
| 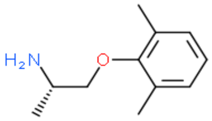 | 0 | 271 | 379 | -4.128 | 30 | 3 | -3.0838 | -4.2007 | -15.38 | 2.5748 | 13.178 | 13.084 | -61.60582 | 1.15301 | 1 |
| 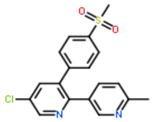 | 0 | 1420 | 1628 | -4.105 | 39 | 1 | 0 | -6.4616 | -27.37 | 2.8428 | 12.602 | 23.993 | -125.7545 | 1.47131 | 1 |
| 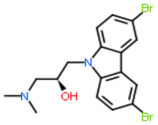 | 0 | 1515 | WSK | -4.097 | 40 | 5 | -2.9806 | -6.4979 | -20.27 | 1.254 | 12.614 | 22.51 | -115.2318 | 1.37107 | 1 |
| 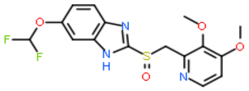 | 0 | 108 | 213 | -4.08 | 41 | 4 | -2.8481 | -6.1749 | -23.15 | 6.5648 | 19.298 | 14.55 | -105.6125 | 1.58587 | 1 |
| 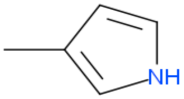 | 0 | 1697 | C5H7N | -4.079 | 13 | 0 | -1.2746 | -2.751 | -10.61 | 0.0097 | 7.2259 | 9.3601 | -42.85571 | 0.50763 | 1 |
| 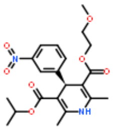 | 0 | 284 | 393 | -4.069 | 56 | 6 | -3.5844 | -6.0529 | -32.03 | 6.5184 | 19.42 | 28.795 | -143.3788 | 1.90169 | 1 |
| 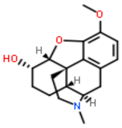 | 0 | 1347 | 1551 | -4.065 | 45 | 1 | -4.08 | -4.7964 | -16.01 | 1.9472 | 15.725 | 18.056 | -84.64132 | 1.17941 | 1 |
| 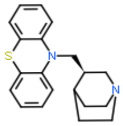 | 0 | 943 | 1071 | -4.051 | 45 | 2 | -1.4319 | -5.6928 | -22.64 | 2.0082 | 13.085 | 20.927 | -90.01709 | 1.1227 | 1 |
| 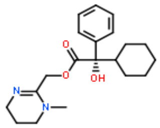 | 0 | 275 | 383 | -4.042 | 53 | 6 | -3.6746 | -6.9927 | -25.59 | 3.2583 | 18.306 | 24.614 | -105.7304 | 1.45523 | 1 |
| 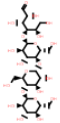 | 0 | 1984 | C24H42O21 | -4.037 | 87 | 28 | -15.228 | -5.1733 | -32.81 | 15.876 | 35.042 | 27.517 | -184.2405 | 2.41717 | 1 |

| | | | | | | | | | | | | | | | |
|---|---|---|---|---|---|---|---|---|---|---|---|---|---|---|---|
| 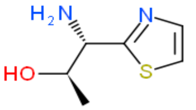 | 0 | 3365 | C6H10N2OS | -4.027 | 20 | 4 | -6.5107 | -3.1598 | -12.36 | 3.6073 | 16.91 | 14.662 | -58.92211 | 0.89375 | 1 |
| 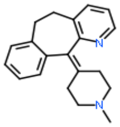 | 0 | 603 | 719 | -3.999 | 44 | 0 | 0 | -6.0941 | -19.59 | 1.4551 | 10.572 | 16.478 | -120.4196 | 1.3234 | 1 |
| 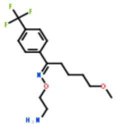 | 0 | 71 | C15H21F3N2O2 | -3.982 | 43 | 8 | -6.0268 | -6.0417 | -18.48 | 4.5445 | 16.891 | 20.6 | -81.22456 | 1.58702 | 1 |
| 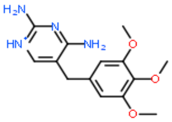 | 0 | 2809 | TRR | -3.956 | 40 | 2 | -5.19 | -5.6594 | -19.43 | 3.188 | 20.244 | 21.361 | -115.2959 | 1.41743 | 1 |
| 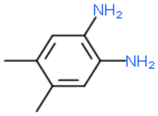 | 0 | 2862 | C8H12N2 | -3.95 | 22 | 0 | -3.7998 | -3.115 | -10.49 | 0.7494 | 10.557 | 14.956 | -51.28158 | 0.57212 | 1 |
| 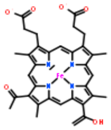 | 0 | 3086 | C34H32FeN4O6 | -3.949 | 75 | 6 | -8.2203 | -6.4032 | -24.35 | 12.171 | 25.343 | 23.608 | -152.9827 | 2.21542 | 1 |
| 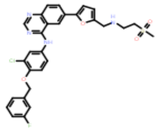 | 0 | 1126 | 1259 | -3.943 | 66 | 7 | -2.149 | -8.2433 | -36.37 | 15.073 | 22.868 | 19.683 | -124.29 | 2.71651 | 1 |
| 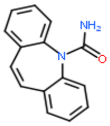 | 0 | 451 | 564 | -3.935 | 30 | 1 | -1.1488 | -3.8456 | -20.54 | 0.8878 | 12.135 | 17.962 | -59.60244 | 0.84609 | 1 |
| 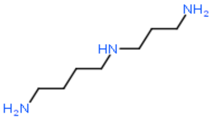 | 0 | 3220 | C7H19N3 | -3.934 | 29 | 9 | -9.1688 | -3.3057 | -13.85 | 7.3733 | 20.446 | 15.408 | -48.27684 | 1.03363 | 1 |
| 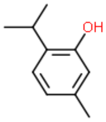 | 0 | 2243 | Thymol | -3.932 | 25 | 1 | -3.0757 | -4.0051 | -13.36 | 2.4418 | 11.618 | 13.813 | -70.31496 | 1.01724 | 1 |
| 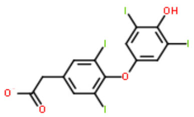 | 0 | 1534 | C14H8I4O4 | -3.925 | 29 | 4 | -3.902 | -6.4644 | -23.4 | 1.7536 | 20.358 | 22.663 | -70.01199 | 1.63269 | 1 |

| | | | | | | | | | | | | | | | |
|---|---|---|---|---|---|---|---|---|---|---|---|---|---|---|---|
| 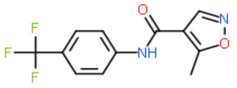 | 0 | 969 | 1097 | -3.918 | 28 | 1 | -1.5385 | -4.4184 | -18.72 | 0.9495 | 12.364 | 16.987 | -74.28579 | 1.27677 | 1 |
| 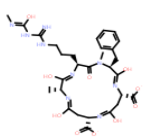 | 0 | 3281 | C29H41N9O10 | -3.906 | 87 | 16 | -14.077 | -6.5381 | -29.92 | 1.0922 | 37.409 | 41.964 | -151.0675 | 2.75364 | 1 |
| 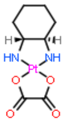 | 0 | 413 | C8H14N2O4Pt | -3.899 | 29 | 0 | -3.6136 | -2.4045 | -15.82 | 0.6913 | 17.365 | 14 | -42.18649 | 0.94927 | 1 |
| 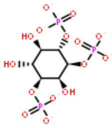 | 0 | 1514 | C6H15O15P3 | -3.872 | 33 | 9 | -11.725 | -1.8508 | -17.88 | 0 | 24.081 | 32.507 | -127.5213 | 1.47844 | 1 |
| 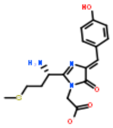 | 0 | 3062 | C16H19N3O4S | -3.86 | 42 | 7 | -5.1656 | -4.5632 | -20.72 | 3.6367 | 22.863 | 14.331 | -109.5357 | 1.6377 | 1 |
| 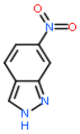 | 0 | 2787 | C7H5N3O2 | -3.855 | 17 | 1 | -1.2888 | -2.3044 | -15.34 | 0.8361 | 9.613 | 12.095 | -36.62772 | 0.7025 | 1 |
| 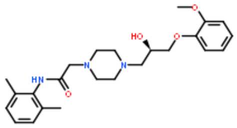 | 0 | 138 | 243 | -3.852 | 64 | 7 | -3.874 | -8.4562 | -29.38 | 4.643 | 22.784 | 25.636 | -134.6393 | 2.0767 | 1 |
| 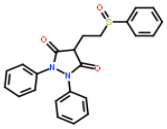 | 0 | 1009 | 1138 | -3.832 | 49 | 6 | -3.9749 | -6.523 | -23.66 | 2.5686 | 19.251 | 22.239 | -151.9807 | 1.9202 | 1 |
| 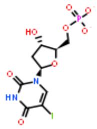 | 0 | 2066 | C9H12IN2O8P | -3.828 | 31 | 5 | -10.533 | -3.1764 | -15.04 | 0 | 21.751 | 31.319 | -92.2533 | 1.37597 | 1 |
| 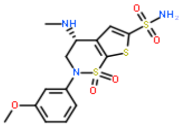 | 0 | 1968 | C14H17N3O5S3 | -3.816 | 42 | 4 | -4.6538 | -5.2305 | -24.28 | 5.5058 | 19.874 | 22.923 | -131.3292 | 1.96787 | 1 |
| 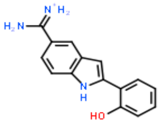 | 0 | 2196 | 124 | -3.816 | 33 | 0 | -5.3701 | -4.1344 | -19.81 | 4.379 | 19.335 | 23.062 | -94.5924 | 1.32869 | 1 |

| | | | | | | | | | | | | | | | | |
|---|---|---|---|---|---|---|---|---|---|---|---|---|---|---|---|---|
| 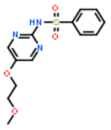 | 0 | 1199 | 1382 | -3.814 | 36 | 6 | -3.01 | -5.3039 | -23.23 | 5.4587 | 16.497 | 17.178 | -98.21579 | 1.62792 | 1 |
| 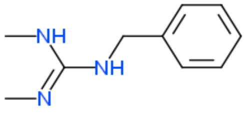 | 0 | 112 | 2368 | -3.809 | 28 | 4 | -0.5533 | -4.2989 | -14.25 | 1.2964 | 7.67 | 8.7779 | -21.58946 | 0.87378 | 1 |
| 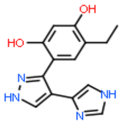 | 0 | 3389 | 43P | -3.804 | 34 | 1 | -6.1269 | -4.0961 | -16.79 | 4.518 | 19.645 | 19.981 | -100.3102 | 1.50512 | 1 |
| 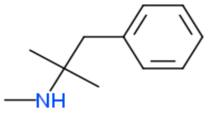 | 0 | 1189 | 1365 | -3.791 | 29 | 3 | -2.2102 | -4.6532 | -15.1 | 2.4858 | 9.7181 | 14.298 | -64.42894 | 0.86986 | 1 |
| 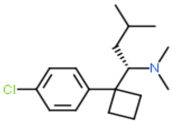 | 0 | 977 | 1105 | -3.778 | 45 | 5 | 0 | -5.5558 | -18.34 | 1.6705 | 10.318 | 9.9136 | -51.56046 | 0.95439 | 1 |
| 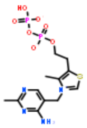 | 0 | 1751 | C12H18N4O7P2S | -3.765 | 43 | 9 | -7.3415 | -4.8074 | -23.99 | 0 | 23.924 | 28.305 | -92.69512 | 1.66326 | 1 |
| 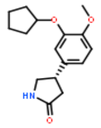 | 0 | 3257 | C16H21NO3 | -3.751 | 41 | 2 | -4.1815 | -5.3898 | -22.01 | 3.4711 | 16.055 | 25.953 | -146.8379 | 1.52098 | 1 |
| 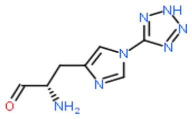 | 0 | 2258 | C7H9N7O | -3.743 | 24 | 4 | -7.9906 | -2.2237 | -18.17 | 5.4125 | 23.223 | 19.802 | -76.80458 | 1.11868 | 1 |
| 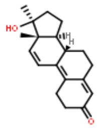 | 0 | 2693 | R18 | -3.732 | 45 | 1 | -1.8771 | -5.0152 | -18.01 | 1.1598 | 14.169 | 15.856 | -118.8858 | 1.725 | 1 |
| 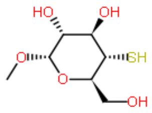 | 0 | 3074 | C7H14O5S | -3.732 | 27 | 6 | -4.3609 | -2.2946 | -11.93 | 2.1393 | 10.884 | 11.697 | -41.89854 | 0.78947 | 1 |
| 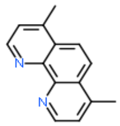 | 0 | 2311 | DPT | -3.731 | 28 | 0 | -1.5641 | -4.1901 | -12.98 | 1.163 | 10.311 | 11.488 | -41.7224 | 0.95836 | 1 |

| | | | | | | | | | | | | | | | |
|---|---|---|---|---|---|---|---|---|---|---|---|---|---|---|---|
| 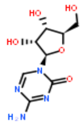 | 0 | 807 | 928 | -3.73 | 29 | 5 | -6.599 | -1.8458 | -17.29 | 0 | 22.809 | 17.255 | -79.56999 | 1.24781 | 1 |
| 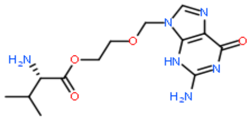 | 0 | 464 | 577 | -3.729 | 43 | 8 | -10.264 | -3.7845 | -21.88 | 6.1624 | 29.481 | 24.311 | -102.4926 | 1.74938 | 1 |
| 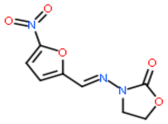 | 0 | 501 | C8H7N3O5 | -3.726 | 23 | 1 | -0.9423 | -3.1637 | -19.01 | 2.4994 | 12.553 | 12.538 | -80.56145 | 1.12579 | 1 |
| 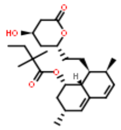 | 0 | 528 | C25H38O5 | -3.725 | 68 | 7 | -4.4812 | -7.133 | -25.95 | 6.2344 | 18.802 | 24.693 | -158.3325 | 1.85442 | 1 |
| 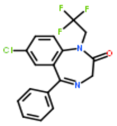 | 0 | 682 | 801 | -3.724 | 36 | 2 | -2.7535 | -5.88 | -19.88 | 4.9845 | 13.281 | 19.556 | -133.7469 | 1.60122 | 1 |
| 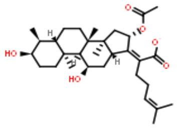 | 0 | 2419 | 2703 | -3.723 | 84 | 7 | -4.2897 | -7.0165 | -25.38 | 0 | 21.153 | 25.981 | -137.6169 | 2.10435 | 1 |
| 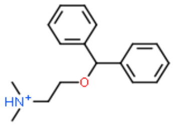 | 0 | 862 | m | -3.712 | 41 | 6 | 0 | -4.9374 | -21.17 | 1.0549 | 10.391 | 13.247 | -9.051656 | 1.14229 | 1 |
| 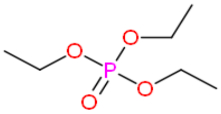 | 0 | 3022 | TEN | -3.707 | 26 | 6 | -3.138 | -4.2456 | -14.34 | 4.6241 | 11.324 | 9.9298 | -64.62464 | 0.8675 | 1 |
| 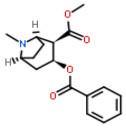 | 0 | 786 | C17H21NO4 | -3.693 | 43 | 3 | -1.4698 | -5.4432 | -20.4 | 2.4517 | 13.138 | 16.585 | -80.45692 | 1.30637 | 1 |
| 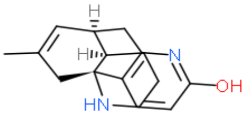 | 0 | 3023 | C16H20N2O | -3.69 | 39 | 0 | -0.5372 | -4.0415 | -16.94 | 0.1928 | 12.563 | 11.793 | -39.75983 | 0.99335 | 1 |
| 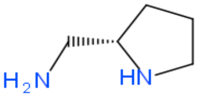 | 0 | 2934 | C5H12N2 | -3.681 | 19 | 2 | -4.1998 | -2.7925 | -7.895 | 1.9669 | 11.859 | 8.2372 | -38.08368 | 0.65064 | 1 |

| | | | | | | | | | | | | | | | |
|---|---|---|---|---|---|---|---|---|---|---|---|---|---|---|---|
| 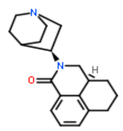 | 0 | 269 | C19H24N2O | -3.681 | 46 | 1 | -2.57 | -5.7168 | -20.51 | 2.1019 | 14.752 | 21.567 | -121.6512 | 1.58387 | 1 |
| 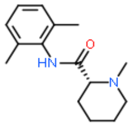 | 0 | 839 | 961 | -3.677 | 40 | 1 | -2.2845 | -5.7214 | -19.57 | 1.2417 | 12.092 | 22.866 | -119.297 | 1.26626 | 1 |
| 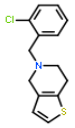 | 0 | 103 | 208 | -3.677 | 31 | 2 | 0 | -5.5299 | -20.54 | 0.7271 | 9.4892 | 17.9 | -76.66189 | 1.24308 | 1 |
| 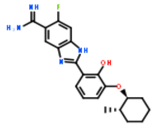 | 0 | 3211 | C21H23FN4O2 | -3.676 | 51 | 1 | -4.7321 | -6.2818 | -29.31 | 13.562 | 21.063 | 26.178 | -132.4279 | 1.85924 | 1 |
| 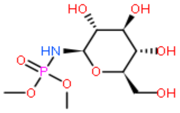 | 0 | 3063 | C8H18NO8P | -3.672 | 36 | 9 | -4.676 | -2.8956 | -17.04 | 0.7594 | 16.334 | 13.693 | -30.30841 | 1.01503 | 1 |
| 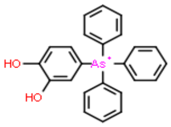 | 0 | 1844 | C24H20AsO2 | -3.664 | 47 | 4 | -0.723 | -6.0687 | -26.63 | 3.9301 | 13.341 | 21.36 | -81.88248 | 1.29116 | 1 |
| 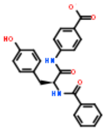 | 0 | 409 | C23H20N2O5 | -3.62 | 49 | 7 | -4.1145 | -6.0241 | -23.24 | 6.5281 | 19.725 | 16.903 | -117.8381 | 1.76885 | 1 |
| 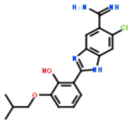 | 0 | 2685 | C18H19ClN4O2 | -3.611 | 44 | 2 | -4.6424 | -6.0546 | -29.4 | 11.188 | 20.737 | 27.463 | -121.1141 | 1.76856 | 1 |
| 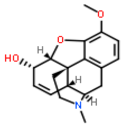 | 0 | 212 | 318 | -3.608 | 43 | 1 | -3.5305 | -4.7052 | -15.49 | 0 | 15.675 | 17.594 | -70.35409 | 1.13593 | 1 |
| 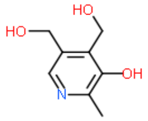 | 0 | 60 | C8H11NO3 | -3.589 | 23 | 4 | -4.3816 | -2.9091 | -10.64 | 0 | 13.189 | 11.809 | -78.44332 | 1.0355 | 1 |
| 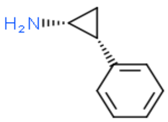 | 0 | 635 | C9H11N | -3.584 | 21 | 2 | -2.4007 | -3.5471 | -12.71 | 0 | 11.384 | 11.878 | -53.67815 | 0.77042 | 1 |

| | | | | | | | | | | | | | | | |
|---|---|---|---|---|---|---|---|---|---|---|---|---|---|---|---|
| 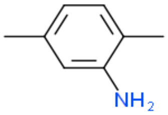 | 0 | 1917 | C8H11N | -3.572 | 20 | 0 | -1.2812 | -3.6562 | -11.82 | 0.5703 | 9.2612 | 10.075 | -47.29199 | 0.74708 | 1 |
| 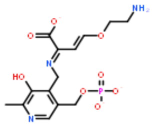 | 0 | 2964 | C14H20N3O8P | -3.563 | 43 | 9 | -12.637 | -4.7731 | -18.22 | 0 | 23.525 | 40.577 | -136.1603 | 1.75204 | 1 |
| 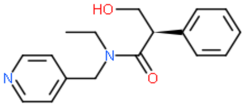 | 0 | 690 | 809 | -3.557 | 41 | 8 | -5.8818 | -5.4936 | -21.38 | 6.5168 | 17.429 | 22.126 | -118.6448 | 1.32928 | 1 |
| 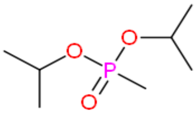 | 0 | 1884 | DII | -3.554 | 28 | 4 | -1.9887 | -4.5027 | -17.18 | 0.846 | 10.237 | 16.805 | -72.78551 | 1.13697 | 1 |
| 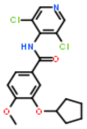 | 0 | 1569 | PIL | -3.545 | 43 | 1 | -2.9784 | -6.6494 | -22.83 | 4.9386 | 19.561 | 19.752 | -104.6737 | 2.00128 | 1 |
| 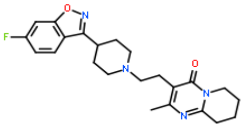 | 0 | 617 | 734 | -3.526 | 57 | 4 | -2.6274 | -7.4288 | -31.4 | 3.9462 | 20.052 | 29.834 | -134.4011 | 1.91506 | 1 |
| 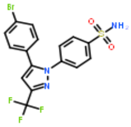 | 0 | 3141 | S58 | -3.521 | 37 | 3 | 0 | -5.5969 | -27.37 | 0.6312 | 13.694 | 22.952 | -96.5634 | 1.31614 | 1 |
| 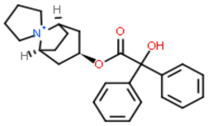 | 0 | 104 | C25H30NO3 | -3.497 | 59 | 5 | -2.2087 | -6.0987 | -23.31 | 5.1495 | 15.132 | 18.251 | -108.7335 | 1.37943 | 1 |
| 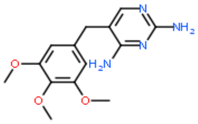 | 0 | 330 | 440 | -3.487 | 39 | 2 | -4.6995 | -6.032 | -22.71 | 3.2386 | 21.819 | 23.712 | -128.5849 | 1.40109 | 1 |
| 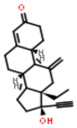 | 0 | 188 | C22H28O2 | -3.486 | 52 | 2 | -1.8767 | -5.6726 | -20.05 | 1.424 | 14.379 | 18.591 | -104.4943 | 1.45312 | 1 |
| 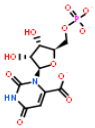 | 0 | 2653 | C10H13N2O11P | -3.477 | 34 | 7 | -9.426 | -2.3356 | -17.45 | 0 | 24.089 | 25.945 | -112.6457 | 1.5299 | 1 |

| | | | | | | | | | | | | | | | |
|---|---|---|---|---|---|---|---|---|---|---|---|---|---|---|---|
| 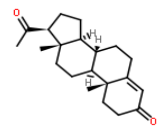 | 0 | 287 | 396 | -3.445 | 53 | 1 | -2.8887 | -4.5794 | -18.23 | 3.5828 | 13.178 | 18.986 | -81.8274 | 1.25726 | 1 |
| 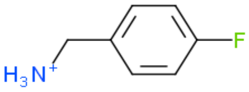 | 0 | 2924 | C7H9FN | -3.443 | 18 | 1 | -1.5495 | -2.5834 | -12.4 | 0.9766 | 6.7848 | 12.621 | -11.03347 | 0.49639 | 1 |
| 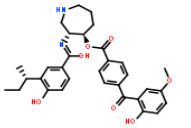 | 0 | 1910 | C32H36N2O7 | -3.435 | 77 | 5 | -4.963 | -7.8799 | -29.75 | 12.273 | 24.37 | 23.494 | -149.2867 | 2.50829 | 1 |
| 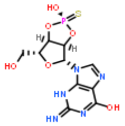 | 0 | 2860 | C10H12N5O6PS | -3.386 | 35 | 5 | -8.4411 | -2.3069 | -20.36 | 1.2296 | 27.909 | 23.046 | -89.66291 | 1.25396 | 1 |
| 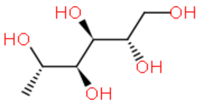 | 0 | 2135 | C6H14O5 | -3.37 | 25 | 9 | -8.948 | -2.365 | -11.73 | 3.7896 | 20.509 | 14.434 | -61.68427 | 0.85014 | 1 |
| 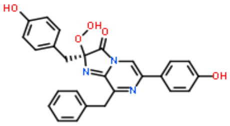 | 0 | 1988 | C26H21N3O5 | -3.348 | 55 | 6 | -4.6416 | -6.1491 | -31.16 | 5.9888 | 24.641 | 27.279 | -137.2384 | 1.66499 | 1 |
| 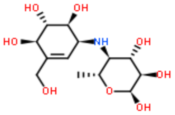 | 0 | 1966 | C13H23NO8 | -3.33 | 45 | 10 | -7.6574 | -3.5138 | -17.61 | 0 | 22.046 | 20.416 | -86.40944 | 1.35803 | 1 |
| 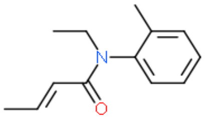 | 0 | 160 | 265 | -3.329 | 32 | 2 | -1.3607 | -5.0441 | -17.76 | 3.3857 | 11.365 | 14.384 | -72.57318 | 1.41622 | 1 |
| 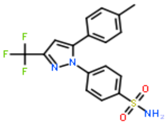 | 0 | 372 | 482 | -3.314 | 40 | 3 | -1.4178 | -5.49 | -24.23 | 1.1834 | 14.285 | 22.659 | -105.1948 | 1.23385 | 1 |
| 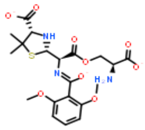 | 0 | 2177 | C20H25N3O9S | -3.307 | 57 | 8 | -8.0633 | -5.87 | -23.59 | 0 | 24.364 | 32.636 | -105.6097 | 1.68394 | 1 |
| 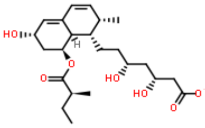 | 0 | 70 | 175 | -3.297 | 65 | 13 | -8.0694 | -5.9965 | -25.3 | 7.9653 | 21.555 | 26.797 | -157.575 | 1.62604 | 1 |

| | | | | | | | | | | | | | | | |
|---|---|---|---|---|---|---|---|---|---|---|---|---|---|---|---|
| 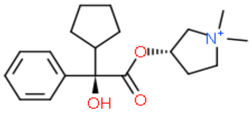 | 0 | 863 | C19H28NO3 | -3.293 | 51 | 5 | -1.932 | -4.8261 | -19.86 | 3.248 | 12.979 | 15.167 | -39.94012 | 1.2607 | 1 |
| 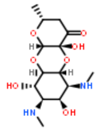 | 0 | 798 | 919 | -3.291 | 47 | 5 | -6.0179 | -4.4892 | -18.81 | 0.5738 | 21.061 | 22.141 | -111.3997 | 1.55098 | 1 |
| 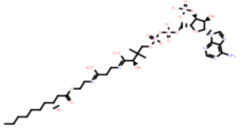 | 0 | 2873 | C31H54N7O18P3S | -3.288 | 110 | 32 | -9.9144 | -8.5771 | -41.33 | 1.1367 | 41.911 | 26.608 | -203.5034 | 3.37424 | 1 |
| 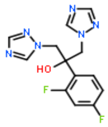 | 0 | 91 | 196 | -3.275 | 34 | 6 | -2.2836 | -4.1103 | -20.31 | 2.8566 | 16.328 | 12.133 | -29.45407 | 0.95837 | 1 |
| 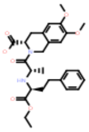 | 0 | 577 | 691 | -3.272 | 69 | 10 | -9.8947 | -7.0284 | -26.58 | 16.182 | 27.617 | 25.195 | -134.2913 | 2.11704 | 1 |
| 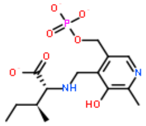 | 0 | 2356 | C14H23N2O7P | -3.262 | 44 | 9 | -9.8308 | -4.5645 | -21.17 | 0 | 20.774 | 37.665 | -117.8792 | 1.64389 | 1 |
| 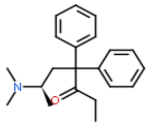 | 0 | 226 | C21H27NO | -3.252 | 50 | 7 | -1.274 | -5.4372 | -20.46 | 5.6992 | 11.603 | 11.747 | -41.21775 | 1.32875 | 1 |
| 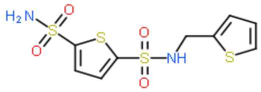 | 0 | 2682 | TPD | -3.222 | 29 | 6 | -2.364 | -3.6203 | -20.32 | 2.402 | 14.321 | 14.724 | -70.77219 | 1.40927 | 1 |
| 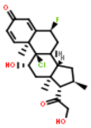 | 0 | 719 | C22H28ClFO4 | -3.21 | 56 | 4 | -2.5665 | -5.0967 | -20.57 | 2.0486 | 18.556 | 14.725 | -93.50091 | 1.59975 | 1 |
| 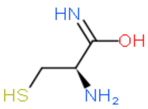 | 0 | 2953 | C3H8N2OS | -3.2 | 15 | 5 | -4.7526 | -1.3361 | -12.25 | 0 | 14.692 | 12.047 | -39.84435 | 0.60776 | 1 |
| 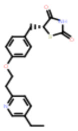 | 0 | 1003 | 1132 | -3.187 | 45 | 6 | -4.5534 | -6.0629 | -27.13 | 2.9394 | 20.16 | 28.852 | -149.3333 | 1.70579 | 1 |

| | | | | | | | | | | | | | | | |
|---|---|---|---|---|---|---|---|---|---|---|---|---|---|---|---|
| 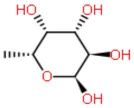 | 0 | 3149 | C6H12O5 | -3.184 | 23 | 4 | -4.5299 | -1.4668 | -9.669 | 3.2293 | 16.024 | 3.8977 | -26.91633 | 0.72431 | 1 |
| 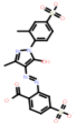 | 0 | 3109 | C18H16N4O9S2 | -3.172 | 46 | 3 | -8.0198 | -5.5239 | -19.94 | 8.9115 | 26.553 | 20.759 | -161.4916 | 1.77003 | 1 |
| 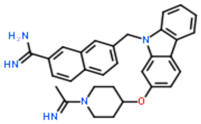 | 0 | 1870 | C31H31N5O | -3.163 | 68 | 4 | -4.5381 | -7.075 | -28.24 | 5.7021 | 20.982 | 29.905 | -109.8764 | 1.72941 | 1 |
| 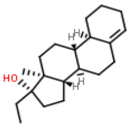 | 0 | 1295 | 1493 | -3.161 | 53 | 2 | -1.3689 | -5.0473 | -18.56 | 1.0161 | 11.789 | 17.572 | -92.50232 | 1.2127 | 1 |
| 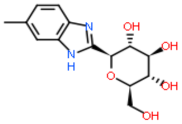 | 0 | 2816 | C14H18N2O5 | -3.151 | 39 | 6 | -5.3778 | -4.5896 | -22.05 | 0 | 22.239 | 23.178 | -91.97188 | 1.53341 | 1 |
| 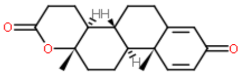 | 0 | 773 | 894 | -3.15 | 46 | 0 | -1.2736 | -4.9856 | -18.48 | 0.7695 | 13.764 | 16.827 | -99.11015 | 1.63638 | 1 |
| 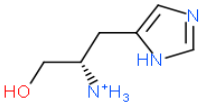 | 0 | 3446 | C6H12N3O | -3.147 | 22 | 4 | -4.2565 | -2.1644 | -12.53 | 0.7513 | 10.96 | 15.952 | -38.9072 | 0.83679 | 1 |
| 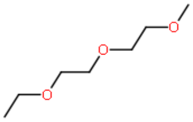 | 0 | 3171 | C7H16O3 | -3.131 | 26 | 7 | -4.2338 | -4.4682 | -11.21 | 0 | 13.445 | 11.358 | -64.78674 | 1.2691 | 1 |
| 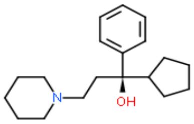 | 0 | 820 | 942 | -3.113 | 50 | 6 | 0 | -5.9754 | -23.3 | 2.0909 | 10.324 | 17.564 | -86.29239 | 1.29031 | 1 |
| 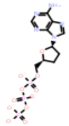 | 0 | 1942 | C10H16N5O11P3 | -3.084 | 41 | 8 | -16.1 | -4.0462 | -29.43 | 5.1304 | 32.938 | 56.371 | -119.1495 | 1.70318 | 1 |
| 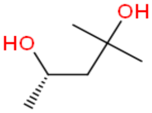 | 0 | 3218 | C6H14O2 | -3.082 | 22 | 4 | -4.4619 | -3.1464 | -6.35 | 0.1393 | 10.011 | 10.11 | -53.16376 | 0.73298 | 1 |

| | | | | | | | | | | | | | | | |
|---|---|---|---|---|---|---|---|---|---|---|---|---|---|---|---|
| 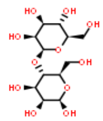 | 0 | 1475 | C12H22O11 | -3.079 | 45 | 12 | -17.792 | -3.2637 | -6.036 | 13.412 | 29.317 | 19.579 | -85.83214 | 1.40445 | 1 |
| 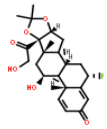 | 0 | 75 | C24H31FO6 | -3.079 | 62 | 4 | -2.8476 | -5.2815 | -19.97 | 1.1404 | 14.319 | 20.923 | -81.2868 | 1.20655 | 1 |
| 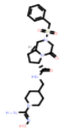 | 0 | 2437 | C22H32N6O5S | -3.07 | 66 | 8 | -9.8225 | -6.8304 | -25.27 | 12.827 | 26.85 | 28.773 | -143.5693 | 2.18529 | 1 |
| 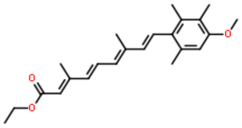 | 0 | 805 | 926 | -3.054 | 56 | 1 | -3.2736 | -8.3139 | -29.04 | 2.6659 | 20.661 | 33.545 | -104.5072 | 2.01735 | 1 |
| 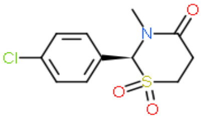 | 0 | 1048 | 1178 | -3.05 | 29 | 1 | -1.2067 | -4.1984 | -17.85 | 7.9509 | 9.3096 | 12.595 | -88.62902 | 1.01001 | 1 |
| 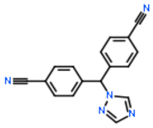 | 0 | 882 | 1006 | -3.049 | 33 | 4 | -4.4565 | -5.0822 | -21.69 | 1.8807 | 19.283 | 23.229 | -117.3873 | 1.47721 | 1 |
| 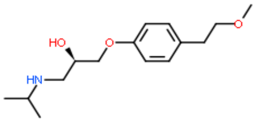 | 0 | 159 | 264 | -3.049 | 44 | 9 | -4.2859 | -6.3439 | -21.8 | 3.5326 | 18.521 | 18.837 | -89.10274 | 1.32349 | 1 |
| 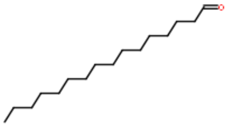 | 0 | 3052 | C16H32O | -3.032 | 49 | 14 | -4.5541 | -6.5895 | -20.84 | 3.9902 | 15.188 | 17.511 | -87.72454 | 1.48753 | 1 |
| 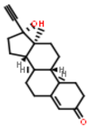 | 0 | 601 | C20H26O2 | -3.017 | 48 | 1 | -1.9568 | -5.3537 | -16.59 | 0.8898 | 14.293 | 15.538 | -144.9077 | 1.76745 | 1 |
| 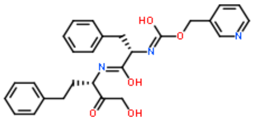 | 0 | 1885 | C27H29N3O5 | -3.015 | 64 | 15 | -5.6542 | -6.91 | -37.03 | 9.4396 | 25.974 | 28.495 | -141.357 | 1.80927 | 1 |
| 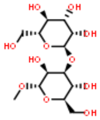 | 0 | 2096 | C13H24O11 | -3.004 | 48 | 12 | -10.542 | -3.6239 | -19.39 | 12.261 | 23.558 | 20.084 | -132.4134 | 1.47607 | 1 |

| | | | | | | | | | | | | | | | |
|---|---|---|---|---|---|---|---|---|---|---|---|---|---|---|---|
| 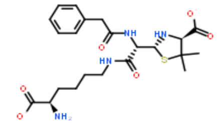 | 0 | 774 | C28H46N6O8S | -2.997 | 63 | 13 | -10.566 | -5.1167 | -26.76 | 7.8537 | 27.233 | 31.8 | -136.6366 | 1.97686 | 1 |
| 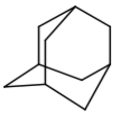 | 0 | 3276 | C10H16 | -2.97 | 26 | 0 | 0 | -2.9508 | -9.392 | 0.138 | 5.0642 | 6.845 | -35.2129 | 0.55571 | 1 |
| 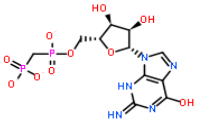 | 0 | 3150 | C11H17N5O10P2 | -2.965 | 42 | 9 | -14.1 | -3.467 | -22.62 | 0 | 23.437 | 52.543 | -140.2282 | 1.58992 | 1 |
| 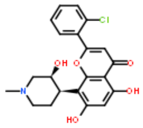 | 0 | 3159 | C21H20ClNO5 | -2.964 | 48 | 2 | -1.4429 | -5.3588 | -22.67 | 0 | 16.402 | 20.378 | -78.80192 | 1.22402 | 1 |
| 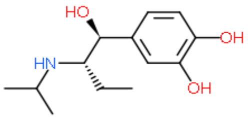 | 0 | 116 | 221 | -2.957 | 38 | 6 | -5.2187 | -4.2637 | -19.36 | 4.4478 | 15.586 | 21.981 | -71.30612 | 1.27613 | 1 |
| 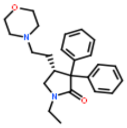 | 0 | 448 | 561 | -2.95 | 58 | 6 | 0 | -6.6381 | -28.51 | 1.7486 | 15.218 | 21.128 | -93.59055 | 1.40483 | 1 |
| 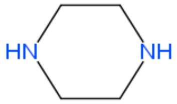 | 0 | 479 | 592 | -2.91 | 16 | 0 | -2.2547 | -2.7286 | -9.957 | 1.3094 | 9.4827 | 10.124 | -37.34644 | 0.40594 | 1 |
| 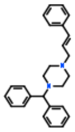 | 0 | 455 | 568 | -2.902 | 56 | 6 | 0 | -7.9853 | -30.51 | 5.9439 | 14.209 | 22.928 | -109.5097 | 1.60329 | 1 |
| 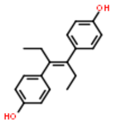 | 0 | 150 | C18H20O2 | -2.9 | 40 | 2 | -2.6905 | -5.4065 | -19.53 | 1.8381 | 15.557 | 19.828 | -111.7629 | 1.33046 | 1 |
| 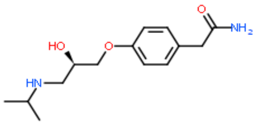 | 0 | 228 | 335 | -2.889 | 41 | 8 | -4.5558 | -5.1095 | -23.04 | 3.932 | 21.244 | 18.192 | -88.26411 | 1.24262 | 1 |
| 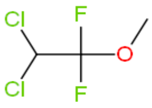 | 0 | 903 | 1028 | -2.884 | 12 | 2 | -1.7626 | -3.491 | -9.57 | 0 | 8.2158 | 9.2768 | -51.29971 | 0.68685 | 1 |

| | | | | | | | | | | | | | | | |
|---|---|---|---|---|---|---|---|---|---|---|---|---|---|---|---|
| 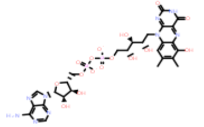 | 0 | 2374 | C27H33N9O16P2 | -2.867 | 85 | 18 | -9.8678 | -4.7407 | -29.43 | 19.469 | 30.781 | 13.84 | -161.2703 | 2.4947 | 1 |
| 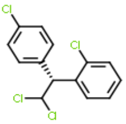 | 0 | 535 | 648 | -2.858 | 28 | 3 | 0 | -5.5942 | -20.76 | 0.9232 | 8.3637 | 19.731 | -83.76972 | 0.85491 | 1 |
| 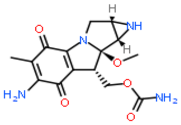 | 0 | 199 | 305 | -2.828 | 42 | 3 | -3.8565 | -4.386 | -23.75 | 1.0174 | 23.829 | 20.206 | -118.705 | 1.47712 | 1 |
| 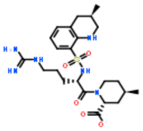 | 0 | 173 | C23H36N6O5S | -2.815 | 70 | 11 | -10.031 | -5.7246 | -30.04 | 15.155 | 28.102 | 29.801 | -161.3136 | 1.84533 | 1 |
| 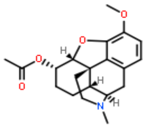 | 0 | 1334 | 1538 | -2.811 | 50 | 1 | -0.6838 | -5.2882 | -20.19 | 0 | 17.105 | 13.982 | -84.15506 | 1.21045 | 1 |
| 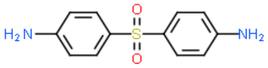 | 0 | 145 | 250 | -2.807 | 29 | 2 | -0.843 | -4.0727 | -18.49 | 1.9196 | 12.945 | 12.857 | -78.22842 | 1.13448 | 1 |
| 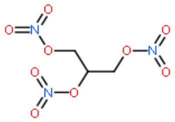 | 0 | 611 | 727 | -2.806 | 20 | 8 | -1.8399 | -1.4923 | -19.81 | 5.4624 | 10.53 | 10.126 | -76.47041 | 0.87096 | 1 |
| 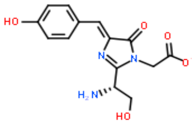 | 0 | 3196 | C14H15N3O5 | -2.799 | 36 | 6 | -6.5442 | -3.5979 | -19.23 | 3.3123 | 21.13 | 21.161 | -89.02261 | 1.43542 | 1 |
| 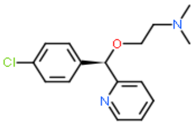 | 0 | 631 | 748 | -2.771 | 39 | 6 | -1.9475 | -6.4848 | -21.98 | 4.3138 | 13.672 | 18.244 | -133.0381 | 1.41219 | 1 |
| 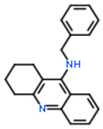 | 0 | 3319 | C20H20N2 | -2.769 | 42 | 2 | -1.1843 | -6.1992 | -20.96 | 2.4854 | 11.878 | 20.812 | -110.6073 | 1.23929 | 1 |
| 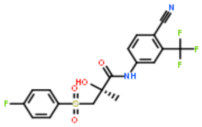 | 0 | 999 | 2375 | -2.763 | 43 | 5 | -2.2013 | -5.6306 | -27.04 | 5.032 | 17.235 | 22.23 | -126.9484 | 1.51028 | 1 |

| | | | | | | | | | | | | | | | |
|---|---|---|---|---|---|---|---|---|---|---|---|---|---|---|---|
| 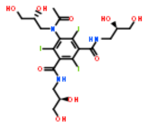 | 0 | 1187 | 1362 | -2.74 | 60 | 16 | -7.0569 | -5.9812 | -23.29 | 0.5895 | 26.442 | 19.139 | -124.4178 | 2.16924 | 1 |
| 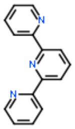 | 0 | 1680 | C15H11ClN3Pt | -2.733 | 29 | 0 | -0.8795 | -5.4735 | -20.25 | 1.1959 | 13.388 | 19.162 | -105.5638 | 1.33258 | 1 |
| 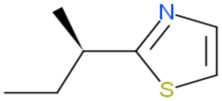 | 0 | 2775 | C7H11NS | -2.723 | 20 | 2 | -1.6498 | -4.1345 | -11.85 | 0.5421 | 8.0297 | 12.914 | -36.49621 | 0.76093 | 1 |
| 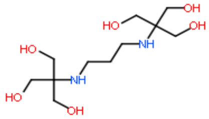 | 0 | 2395 | C11H26N2O6 | -2.709 | 45 | 18 | -9.7294 | -2.9917 | -17.99 | 7.7784 | 20.816 | 16.392 | -43.48919 | 1.14368 | 1 |
| 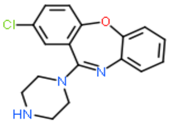 | 0 | 430 | 543 | -2.703 | 38 | 1 | -3.0797 | -5.6466 | -21.43 | 2.6688 | 16.666 | 23.636 | -90.06774 | 1.52655 | 1 |
| 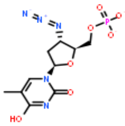 | 0 | 3313 | C10H14N5O7P | -2.688 | 35 | 6 | -10.629 | -2.7218 | -26.08 | 9.6386 | 25.439 | 35.821 | -80.42565 | 1.37563 | 1 |
| 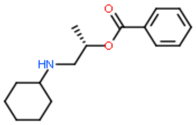 | 0 | 363 | 473 | -2.683 | 42 | 5 | -5.5772 | -6.1087 | -19.52 | 7.8456 | 15.097 | 24.265 | -86.74754 | 1.42216 | 1 |
| 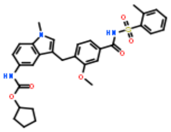 | 0 | 436 | C31H33N3O6S | -2.68 | 74 | 6 | -2.0045 | -9.1889 | -38.95 | 11.053 | 21.595 | 31.923 | -193.9842 | 2.18967 | 1 |
| 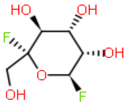 | 0 | 2703 | C6H10F2O5 | -2.677 | 23 | 5 | -5.5405 | -2.4161 | -11.06 | 0.4432 | 15.996 | 13.271 | -82.68156 | 0.83483 | 1 |
| 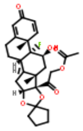 | 0 | 182 | C28H35FO7 | -2.676 | 71 | 4 | -2.8368 | -5.7182 | -21.11 | 1.6744 | 16.094 | 21.161 | -106.2338 | 1.51208 | 1 |
| 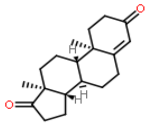 | 0 | 1332 | 1536 | -2.667 | 47 | 0 | -0.9943 | -5.0927 | -17.13 | 0.4804 | 13.063 | 15.61 | -113.9819 | 1.58486 | 1 |

| | | | | | | | | | | | | | | | |
|---|---|---|---|---|---|---|---|---|---|---|---|---|---|---|---|
| 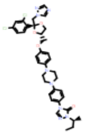 | 0 | 1037 | C35H38Cl2N8O4 | -2.654 | 87 | 7 | -4.3804 | -10.568 | -35.46 | 12.092 | 26.041 | 30.279 | -204.3546 | 2.96574 | 1 |
| 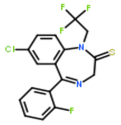 | 0 | 1383 | 1589 | -2.65 | 36 | 1 | -1.3835 | -5.3108 | -20.73 | 4.4499 | 12.863 | 18.524 | -123.9556 | 1.6668 | 1 |
| 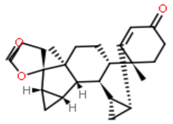 | 0 | 1206 | 1395 | -2.64 | 57 | 0 | -3.2255 | -5.0232 | -16.1 | 2.0598 | 14.123 | 19.934 | -54.31573 | 1.24521 | 1 |
| 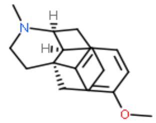 | 0 | 402 | C18H25NO | -2.636 | 45 | 0 | -1.6143 | -5.2891 | -17.2 | 1.3018 | 12.188 | 18.59 | -79.5204 | 1.09696 | 1 |
| 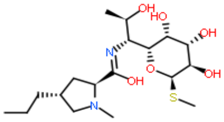 | 0 | 1419 | C18H34N2O6S | -2.633 | 61 | 12 | -4.2617 | -5.1575 | -20.3 | 0 | 20.57 | 14.058 | -69.79813 | 1.69178 | 1 |
| 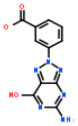 | 0 | 1675 | A45 | -2.623 | 27 | 1 | -7.7495 | -3.2222 | -15.22 | 5.933 | 22.835 | 19.815 | -90.12026 | 1.13734 | 1 |
| 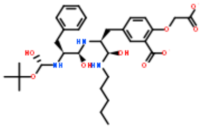 | 0 | 2673 | C31H47N3O9 | -2.623 | 88 | 23 | -6.0981 | -7.0485 | -30.95 | 4.5414 | 31.353 | 12.783 | -75.81776 | 2.60189 | 1 |
| 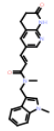 | 0 | 1479 | IDN | -2.608 | 50 | 3 | -2.9714 | -6.1935 | -28.51 | 5.5766 | 22.413 | 23.662 | -155.6016 | 1.82263 | 1 |
| 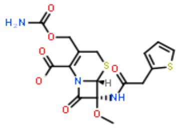 | 0 | 1165 | C16H17N3O7S2 | -2.598 | 44 | 7 | -4.7576 | -4.6311 | -22.02 | 7.2814 | 23.246 | 12.968 | -90.19656 | 1.80026 | 1 |
| 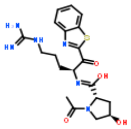 | 0 | 2517 | C20H26N6O4S | -2.595 | 57 | 11 | -6.2514 | -5.7233 | -27.9 | 7.3848 | 25.571 | 22.338 | -82.33997 | 1.28387 | 1 |
| 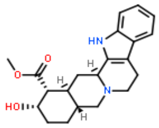 | 0 | 1203 | 1392 | -2.585 | 52 | 2 | -1.8054 | -5.4839 | -20.77 | 2.5023 | 15.259 | 18.613 | -114.7561 | 1.45942 | 1 |

| | | | | | | | | | | | | | | | |
|---|---|---|---|---|---|---|---|---|---|---|---|---|---|---|---|
| 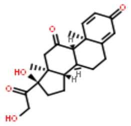 | 0 | 522 | 635 | -2.578 | 52 | 4 | -7.0999 | -4.774 | -12.36 | 4.0475 | 21.564 | 15.066 | -113.8156 | 1.70725 | 1 |
| 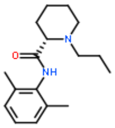 | 0 | 190 | C17H26N2O | -2.567 | 46 | 3 | -1.7761 | -6.173 | -18.85 | 0 | 12.032 | 21.352 | -112.1052 | 1.33472 | 1 |
| 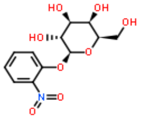 | 0 | 1686 | C12H15NO8 | -2.547 | 36 | 7 | -3.7993 | -3.2196 | -19.65 | 7.2536 | 14.676 | 14.108 | -106.0448 | 1.45982 | 1 |
| 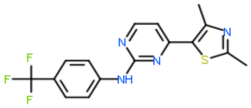 | 0 | 2614 | C16H13F3N4S | -2.535 | 37 | 1 | -2.6098 | -6.6749 | -18.86 | 2.0957 | 18.019 | 18.228 | -111.5866 | 1.51249 | 1 |
| 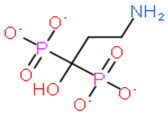 | 0 | 177 | 282 | -2.526 | 20 | 6 | -11.325 | -1.3033 | -14.78 | 3.4844 | 20.175 | 31.758 | -61.10903 | 0.7123 | 1 |
| 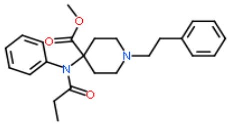 | 0 | 1331 | 1535 | -2.525 | 59 | 8 | -2.0236 | -7.6663 | -24.23 | 4.8999 | 16.129 | 18.377 | -125.3732 | 1.8892 | 1 |
| 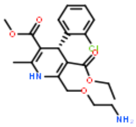 | 0 | 273 | C20H25ClN2O5 | -2.507 | 53 | 7 | -5.645 | -6.6368 | -27.58 | 8.3146 | 20.354 | 29.317 | -116.1516 | 1.65989 | 1 |
| 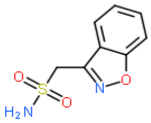 | 0 | 788 | 909 | -2.503 | 22 | 3 | -1.1987 | -2.899 | -16.92 | 1.9794 | 11.159 | 12.18 | -57.50277 | 0.83828 | 1 |
| 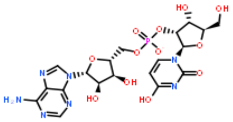 | 0 | 3114 | C19H24N7O12P | -2.487 | 62 | 13 | -13.108 | -4.4046 | -28.2 | 11.721 | 36.098 | 30.19 | -141.4091 | 2.01563 | 1 |
| 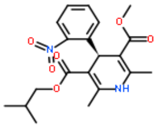 | 0 | 292 | 401 | -2.466 | 52 | 4 | -2.8274 | -5.5518 | -23.47 | 7.7205 | 16.38 | 19.035 | -79.8164 | 1.38495 | 1 |
| 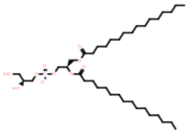 | 0 | 1803 | C38H75O10P | -2.457 | 123 | 40 | -14.438 | -8.9127 | -43.33 | 24.71 | 36.413 | 26.117 | -178.5565 | 3.85632 | 1 |

| | | | | | | | | | | | | | | | |
|---|---|---|---|---|---|---|---|---|---|---|---|---|---|---|---|
| 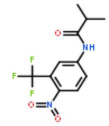 | 0 | 389 | 499 | -2.454 | 30 | 3 | -1.1207 | -4.7022 | -21.62 | 2.4594 | 11.061 | 20.573 | -91.71401 | 1.2284 | 1 |
| 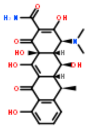 | 0 | 149 | m | -2.435 | 56 | 3 | -6.4157 | -4.7518 | -22.02 | 0 | 25.211 | 27.714 | -132.2801 | 1.72825 | 1 |
| 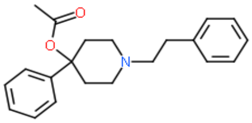 | 0 | 1358 | C21H25NO2 | -2.422 | 49 | 5 | -2.7815 | -6.1305 | -19.96 | 4.1528 | 12.459 | 20.984 | -77.09538 | 1.5124 | 1 |
| 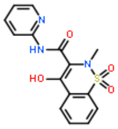 | 0 | 441 | C15H13N3O4S | -2.417 | 36 | 0 | -0.7174 | -5.192 | -22.75 | 0.926 | 14.145 | 21.919 | -107.8434 | 1.6044 | 1 |
| 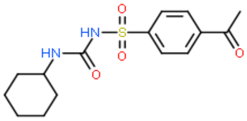 | 0 | 304 | 414 | -2.404 | 42 | 5 | -2.0198 | -4.947 | -25.05 | 0.9648 | 17.548 | 21.717 | -98.19846 | 1.69473 | 1 |
| 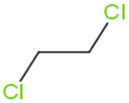 | 0 | 3374 | C2H4Cl2 | -2.4 | 8 | 1 | 0 | -2.4234 | -7.415 | 0.0228 | 3.696 | 4.929 | -1.149235 | 0.37751 | 1 |
| 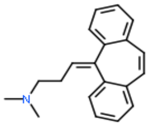 | 0 | 803 | 924 | -2.396 | 42 | 3 | -0.6952 | -5.8408 | -21.34 | 0.8703 | 12.379 | 19.716 | -79.27933 | 1.26391 | 1 |
| 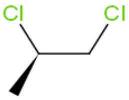 | 0 | 1487 | C3H6Cl2 | -2.392 | 11 | 1 | 0 | -2.5051 | -8.81 | 0.2443 | 4.857 | 5.616 | -5.011306 | 0.46601 | 1 |
| 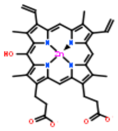 | 0 | 2619 | C34H32N4O5Zn | -2.385 | 74 | 6 | -6.2177 | -6.5327 | -25.48 | 9.1468 | 24.607 | 23.731 | -143.5047 | 2.06537 | 1 |
| 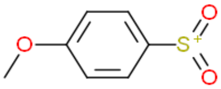 | 0 | 2725 | [NO | -2.382 | 18 | 1 | 0 | -2.8295 | -16.24 | 0.1075 | 8.4923 | 13.209 | -46.8753 | 0.74823 | 1 |
| 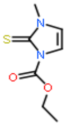 | 0 | 280 | 389 | -2.381 | 22 | 2 | -1.5694 | -3.5267 | -16.74 | 0.8604 | 10.322 | 17.017 | -72.03452 | 1.10336 | 1 |

| | | | | | | | | | | | | | | | |
|---|---|---|---|---|---|---|---|---|---|---|---|---|---|---|---|
| 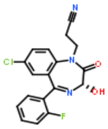 | 0 | 1388 | 1594 | -2.374 | 38 | 3 | -2.7751 | -4.9914 | -24.41 | 9.4694 | 16.868 | 18.588 | -84.80306 | 1.34902 | 1 |
| 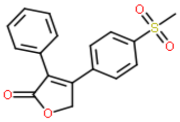 | 0 | 420 | 533 | -2.369 | 36 | 2 | -1.1193 | -5.0569 | -22.34 | 1.53 | 14.847 | 19.602 | -120.7452 | 1.76567 | 1 |
| 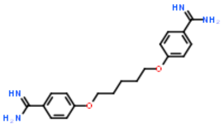 | 0 | 621 | 738 | -2.365 | 49 | 6 | -5.5992 | -5.4242 | -26.53 | 7.7477 | 24.799 | 22.9 | -110.2859 | 1.5969 | 1 |
| 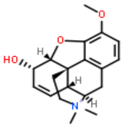 | 0 | 1280 | 1477 | -2.351 | 47 | 1 | -3.1457 | -4.7941 | -17.4 | 3.1072 | 15.122 | 18.807 | -78.11459 | 1.14991 | 1 |
| 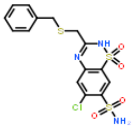 | 0 | 449 | C15H14ClN3O4S3 | -2.35 | 40 | 6 | -0.6799 | -4.6311 | -30.65 | 3.3193 | 17.095 | 22.288 | -119.2675 | 1.80546 | 1 |
| 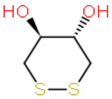 | 0 | 2409 | C4H8O2S2 | -2.337 | 16 | 2 | -3.1722 | -2.1689 | -9.204 | 0 | 13.086 | 8.077 | -53.79945 | 0.60616 | 1 |
| 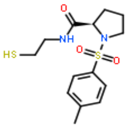 | 0 | 3453 | TP2 | -2.329 | 41 | 6 | -1.8571 | -4.9854 | -24.36 | 6.4423 | 12.656 | 19.862 | -103.9159 | 1.42753 | 1 |
| 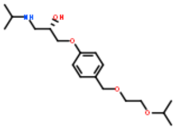 | 0 | 499 | C18H31NO4 | -2.326 | 54 | 12 | -7.5594 | -7.8801 | -24.6 | 7.0597 | 21.362 | 29.118 | -119.9788 | 1.77502 | 1 |
| 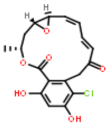 | 0 | 3398 | C18H17ClO6 | -2.323 | 42 | 0 | -4.4257 | -4.2035 | -17.36 | 5.0952 | 18.334 | 18.553 | -52.70054 | 1.21165 | 1 |
| 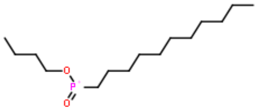 | 0 | 2190 | C11 | -2.32 | 51 | 14 | -2.2733 | -6.4756 | -23.37 | 3.2084 | 14.703 | 14.833 | -103.4158 | 1.67091 | 1 |
| 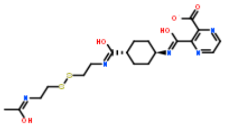 | 0 | 2343 | C19H27N5O5S2 | -2.314 | 57 | 13 | -8.4666 | -6.0175 | -23.33 | 7.2982 | 28.002 | 20.141 | -108.9326 | 2.00613 | 1 |

| | | | | | | | | | | | | | | | |
|---|---|---|---|---|---|---|---|---|---|---|---|---|---|---|---|
| 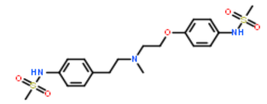 | 0 | 99 | 204 | -2.305 | 56 | 10 | 0 | -6.1762 | -31.1 | 3.0343 | 16.881 | 18.868 | -136.0473 | 1.80294 | 1 |
| 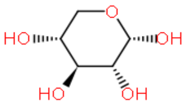 | 0 | 3060 | C5H10O5 | -2.285 | 20 | 4 | -4.8107 | -1.6046 | -8.709 | 0.0277 | 13.013 | 11.355 | -40.33876 | 0.66305 | 1 |
| 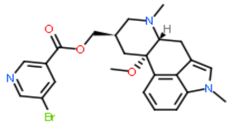 | 0 | 585 | C24H26BrN3O3 | -2.278 | 57 | 3 | -2.7078 | -7.8785 | -30.84 | 5.2209 | 21.292 | 29.985 | -114.5415 | 1.8206 | 1 |
| 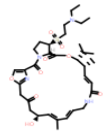 | 0 | 1546 | C34H50N4O9S | -2.27 | 98 | 8 | -3.9003 | -9.8293 | -33.19 | 11.254 | 22.846 | 28.314 | -122.2445 | 2.29667 | 1 |
| 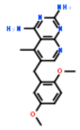 | 0 | 3338 | MXA | -2.265 | 43 | 2 | -5.4744 | -6.6039 | -21.4 | 5.616 | 20.272 | 26.716 | -116.8107 | 1.62642 | 1 |
| 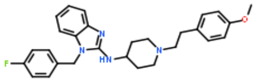 | 0 | 524 | 637 | -2.264 | 65 | 6 | -1.0717 | -9.486 | -31.36 | 6.3116 | 18.195 | 25.954 | -161.2273 | 1.99817 | 1 |
| 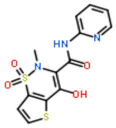 | 0 | 359 | C13H11N3O4S2 | -2.258 | 33 | 0 | -0.6806 | -4.9578 | -19.53 | 1.7029 | 15.408 | 14.755 | -98.02659 | 1.42445 | 1 |
| 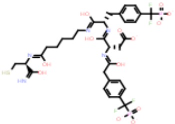 | 0 | 3213 | C32H41F4N5O13P2S | -2.258 | 93 | 29 | -13.623 | -7.2152 | -38.72 | 20.346 | 48.121 | 15.252 | -187.0215 | 3.30903 | 1 |
| 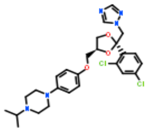 | 0 | 146 | 251 | -2.255 | 67 | 6 | -3.8367 | -9.2076 | -30.87 | 8.256 | 23.903 | 27.291 | -146.7227 | 2.5834 | 1 |
| 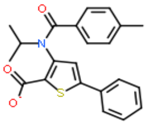 | 0 | 3296 | IPC | -2.253 | 47 | 4 | -6.8104 | -6.3927 | -20.77 | 9.0322 | 18.825 | 27.419 | -137.9963 | 1.96492 | 1 |
| 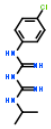 | 0 | 1002 | C11H16ClN5 | -2.227 | 33 | 6 | -6.0282 | -4.9254 | -14.57 | 0 | 15.888 | 23.303 | -87.97095 | 1.26912 | 1 |

| | | | | | | | | | | | | | | | |
|---|---|---|---|---|---|---|---|---|---|---|---|---|---|---|---|
| 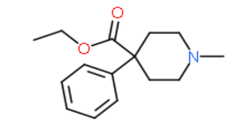 | 0 | 344 | 454 | -2.203 | 39 | 3 | -2.1607 | -5.2224 | -18.58 | 1.9853 | 12.3 | 19.886 | -119.2803 | 1.44866 | 1 |
| 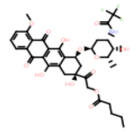 | 0 | 277 | C34H36F3NO13 | -2.192 | 87 | 11 | -7.2127 | -8.1873 | -36.35 | 7.282 | 30.861 | 36.025 | -209.854 | 2.52683 | 1 |
| 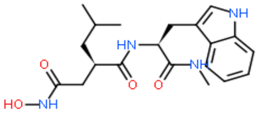 | 0 | 1999 | GM6 | -2.191 | 56 | 11 | -6.5621 | -5.9483 | -25.13 | 9.4792 | 22.981 | 21.222 | -148.1436 | 1.82481 | 1 |
| 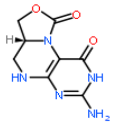 | 0 | 3008 | C8H9N5O3 | -2.173 | 25 | 0 | -4.4942 | -2.1185 | -15.86 | 0 | 19.519 | 17.943 | -85.0426 | 1.1406 | 1 |
| 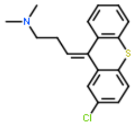 | 0 | 1108 | C18H18ClNS | -2.167 | 39 | 3 | -1.265 | -6.2742 | -24.5 | 3.1543 | 13.925 | 23.637 | -112.5181 | 1.38173 | 1 |
| 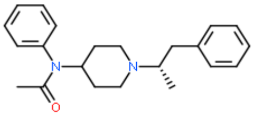 | 0 | 1328 | 1532 | -2.161 | 53 | 6 | -0.856 | -7.392 | -24.19 | 3.2104 | 16.748 | 16.752 | -130.9017 | 1.77826 | 1 |
| 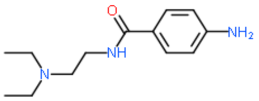 | 0 | 910 | 1035 | -2.158 | 38 | 5 | -2.5783 | -4.2977 | -17.23 | 3.1935 | 12.907 | 14.961 | -57.24237 | 1.26814 | 1 |
| 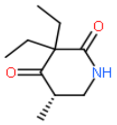 | 0 | 979 | 1107 | -2.149 | 30 | 2 | -1.9463 | -3.9575 | -15.1 | 2.4892 | 11.578 | 13.87 | -72.30609 | 0.92465 | 1 |
| 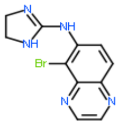 | 0 | 374 | 484 | -2.145 | 27 | 1 | -1.7279 | -5.0209 | -19.73 | 1.3451 | 15.185 | 18.969 | -85.6522 | 1.41038 | 1 |
| 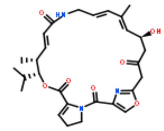 | 0 | 1458 | [NO | -2.139 | 73 | 2 | -2.3176 | -8.3023 | -29.3 | 9.1785 | 24.841 | 20.073 | -154.6006 | 2.07329 | 1 |
| 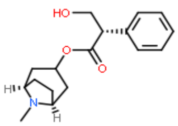 | 0 | 459 | C17H23NO3 | -2.13 | 44 | 5 | -5.8945 | -5.1409 | -14.81 | 5.6929 | 15.506 | 19.68 | -121.6038 | 1.48466 | 1 |

| | | | | | | | | | | | | | | | |
|---|---|---|---|---|---|---|---|---|---|---|---|---|---|---|---|
| 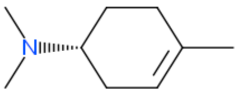 | 0 | 1579 | C9H17N | -2.119 | 27 | 1 | 0 | -4.4693 | -14.04 | 0.3774 | 8.7928 | 11.356 | -53.36559 | 0.94079 | 1 |
| 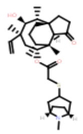 | 0 | 1125 | C30H47NO4S | -2.115 | 83 | 6 | -2.4542 | -7.6866 | -29.87 | 11.89 | 19.252 | 21.123 | -128.4341 | 2.01937 | 1 |
| 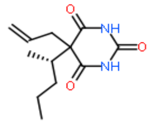 | 0 | 308 | C12H18N2O3 | -2.101 | 35 | 5 | -4.7659 | -3.7807 | -20.44 | 2.683 | 18.086 | 22.361 | -103.788 | 1.32279 | 1 |
| 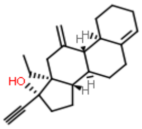 | 0 | 198 | 304 | -2.098 | 53 | 2 | -0.6613 | -5.7864 | -19.87 | 0.6803 | 13.665 | 17.372 | -112.762 | 1.42686 | 1 |
| 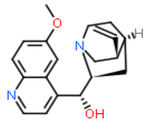 | 0 | 358 | C20H24N2O2 | -2.091 | 48 | 4 | -3.1434 | -5.982 | -17.26 | 4.2206 | 15.787 | 15.69 | -128.2458 | 1.29977 | 1 |
| 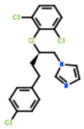 | 0 | 526 | 639 | -2.087 | 42 | 6 | -2.5339 | -6.9553 | -30.89 | 8.0278 | 17.028 | 28.315 | -118.6869 | 1.68022 | 1 |
| 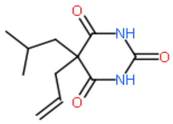 | 0 | 136 | 241 | -2.081 | 32 | 4 | -4.2627 | -3.1458 | -15.72 | 1.5437 | 14.3 | 18.914 | -77.41271 | 1.12121 | 1 |
| 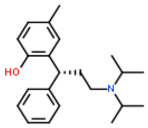 | 0 | 911 | C22H31NO | -2.054 | 55 | 7 | -1.2495 | -6.4351 | -24.38 | 4.3441 | 13.7 | 19.266 | -94.05467 | 1.23302 | 1 |
| 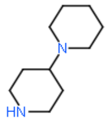 | 0 | 2745 | 4PN | -2.051 | 32 | 1 | -1.3124 | -4.3616 | -12.83 | 0.8631 | 11.498 | 11.036 | -70.03201 | 1.02815 | 1 |
| 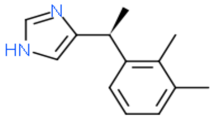 | 0 | 520 | C13H16N2 | -2.047 | 31 | 2 | -1.2865 | -4.9097 | -16.29 | 1.1433 | 10.838 | 16.318 | -74.27638 | 1.15072 | 1 |
| 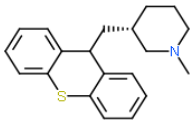 | 0 | 233 | 4167 | -2.046 | 45 | 2 | 0 | -5.8677 | -23.94 | 2.2246 | 12.238 | 21.387 | -86.67791 | 1.27274 | 1 |

| | | | | | | | | | | | | | | | |
|---|---|---|---|---|---|---|---|---|---|---|---|---|---|---|---|
| 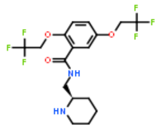 | 0 | 1065 | 1195 | -2.039 | 48 | 5 | -2.8679 | -6.4943 | -23.61 | 5.7208 | 17.303 | 20.862 | -161.0651 | 1.94505 | 1 |
| 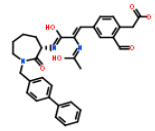 | 0 | 2791 | C33H33N3O6 | -2.033 | 74 | 8 | -6.0685 | -6.6907 | -27.51 | 12.406 | 28.117 | 18.158 | -196.2939 | 2.57837 | 1 |
| 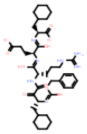 | 0 | 1748 | C38H57N7O10 | -2.023 | 110 | 26 | -8.756 | -8.5222 | -35.43 | 0 | 34.759 | 29.697 | -199.8417 | 2.86882 | 1 |
| 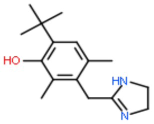 | 0 | 813 | 935 | -2.008 | 43 | 3 | -1.5408 | -5.7393 | -18.01 | 0.974 | 14.64 | 15.724 | -105.8517 | 1.40424 | 1 |
| 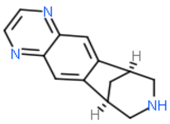 | 0 | 1135 | 1273 | -1.999 | 29 | 0 | -1.4727 | -4.0983 | -15.73 | 1.8974 | 11.478 | 15.839 | -59.70918 | 0.86113 | 1 |
| 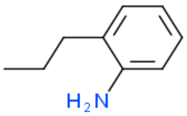 | 0 | 2666 | C9H13N | -1.995 | 23 | 2 | -1.1514 | -4.1579 | -13.42 | 0.5895 | 9.7164 | 12.554 | -45.8318 | 0.82288 | 1 |
| 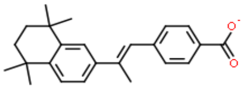 | 0 | 2578 | C24H28O2 | -1.995 | 53 | 2 | -1.2891 | -6.9288 | -22.92 | 2.6329 | 17.487 | 19.597 | -128.5952 | 1.80779 | 1 |
| 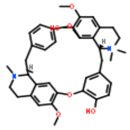 | 0 | 1069 | 1199 | -1.989 | 86 | 0 | -4.2432 | -8.1547 | -26.44 | 12.052 | 20.619 | 27.207 | -112.9242 | 2.34265 | 1 |
| 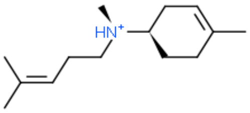 | 0 | 1857 | C14H26N | -1.989 | 41 | 4 | 0 | -5.308 | -18.03 | 1.6012 | 7.0107 | 16.63 | -51.46002 | 0.91834 | 1 |
| 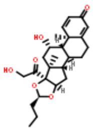 | 0 | 1092 | C25H34O6 | -1.987 | 65 | 6 | -2.8889 | -5.5021 | -20.83 | 2.0851 | 15.856 | 19.838 | -97.09758 | 1.21742 | 1 |
| 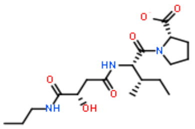 | 0 | 2558 | 074 | -1.972 | 57 | 12 | -7.3166 | -5.6838 | -23.6 | 6.7535 | 24.541 | 21.654 | -154.9842 | 1.79485 | 1 |

| | | | | | | | | | | | | | | | |
|---|---|---|---|---|---|---|---|---|---|---|---|---|---|---|---|
| 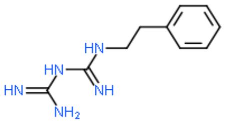 | 0 | 793 | C10H15N5 | -1.96 | 30 | 6 | -3.4382 | -3.5956 | -19.72 | 0 | 17.42 | 18.491 | -65.89049 | 1.25858 | 1 |
| 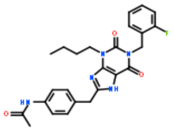 | 0 | 1770 | FTB | -1.955 | 60 | 7 | -4.665 | -7.5007 | -31.27 | 13.486 | 24.259 | 23.715 | -148.256 | 2.23729 | 1 |
| 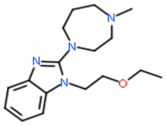 | 0 | 956 | 1084 | -1.955 | 48 | 4 | -0.8586 | -6.5611 | -19.33 | 2.8589 | 11.95 | 16.464 | -98.51662 | 1.56264 | 1 |
| 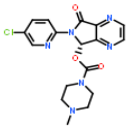 | 0 | 1068 | 1198 | -1.944 | 44 | 2 | -1.8867 | -6.5161 | -23.04 | 0.3281 | 17.95 | 23.291 | -90.74619 | 1.26634 | 1 |
| 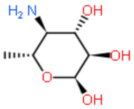 | 0 | 3106 | C6H13NO4 | -1.941 | 24 | 4 | -5.6432 | -2.3931 | -11.79 | 1.3536 | 19.244 | 12.249 | -60.21478 | 0.7098 | 1 |
| 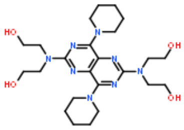 | 0 | 852 | 975 | -1.935 | 76 | 12 | -5.8367 | -7.3147 | -27.44 | 4.5448 | 20.63 | 29.955 | -162.674 | 2.08276 | 1 |
| 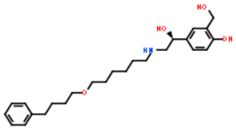 | 0 | 816 | 938 | -1.923 | 67 | 18 | -5.1926 | -7.8536 | -31.23 | 4.4058 | 19.172 | 30.127 | -167.7412 | 2.4858 | 1 |
| 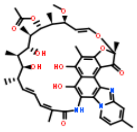 | 0 | 1090 | m | -1.912 | 108 | 4 | 0 | -9.7551 | -33 | 0 | 25.524 | 24.37 | -161.9022 | 2.46978 | 1 |
| 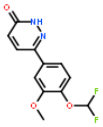 | 0 | 2617 | C12H10F2N2O3 | -1.906 | 29 | 1 | -3.62 | -4.0762 | -18.84 | 1.2981 | 17.843 | 21.305 | -109.9971 | 1.50063 | 1 |
| 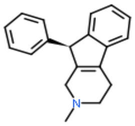 | 0 | 1411 | 1619 | -1.903 | 39 | 1 | 0 | -6.0767 | -22.78 | 1.0182 | 11.277 | 23.145 | -115.2405 | 1.33686 | 1 |
| 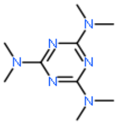 | 0 | 378 | 488 | -1.901 | 33 | 0 | 0 | -4.5367 | -14.7 | 0.9993 | 6.5065 | 15.695 | -26.97952 | 0.75491 | 1 |

| | | | | | | | | | | | | | | | |
|---|---|---|---|---|---|---|---|---|---|---|---|---|---|---|---|
| 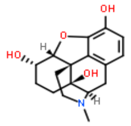 | 0 | 1310 | 1512 | -1.888 | 43 | 2 | -2.6923 | -4.0601 | -18.57 | 4.775 | 15.943 | 15.436 | -91.92529 | 0.98462 | 1 |
| 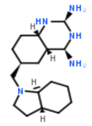 | 0 | 2287 | TQT | -1.86 | 55 | 4 | -3.8067 | -5.6857 | -22.75 | 1.737 | 22.549 | 21.176 | -124.7687 | 1.67626 | 1 |
| 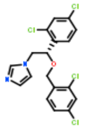 | 0 | 981 | 1110 | -1.856 | 39 | 6 | -3.085 | -7.0471 | -27.32 | 7.3502 | 16.785 | 26.345 | -127.1589 | 1.55881 | 1 |
| 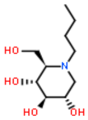 | 0 | 309 | 419 | -1.842 | 36 | 8 | -4.5963 | -3.1087 | -16.72 | 1.3018 | 16.647 | 15.941 | -56.38386 | 0.97425 | 1 |
| 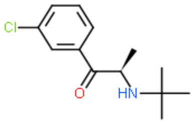 | 0 | 1026 | 1156 | -1.839 | 34 | 3 | -0.4457 | -5.4211 | -21.35 | 0.4347 | 11.685 | 20.379 | -95.87326 | 1.26359 | 1 |
| 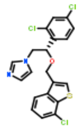 | 0 | 1023 | 1153 | -1.834 | 42 | 6 | -2.16 | -7.49 | -29.67 | 6.6452 | 17.038 | 27.204 | -124.9458 | 1.67149 | 1 |
| 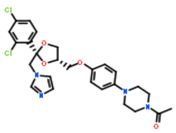 | 0 | 902 | C26H28Cl2N4O4 | -1.829 | 64 | 6 | -2.9862 | -7.4343 | -30.83 | 7.984 | 22.228 | 24.966 | -134.088 | 1.96933 | 1 |
| 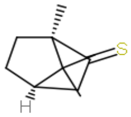 | 0 | 2554 | TCM | -1.808 | 27 | 0 | 0 | -2.882 | -11.07 | 0 | 7.0214 | 8.9824 | -32.99326 | 0.79148 | 1 |
| 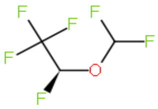 | 0 | 1059 | 1189 | -1.807 | 12 | 2 | -1.1263 | -3.2828 | -8.076 | 0.33 | 5.966 | 8.2505 | -40.36798 | 0.35799 | 1 |
| 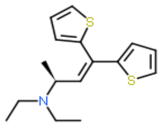 | 0 | 1329 | 1533 | -1.802 | 40 | 4 | -1.221 | -6.3284 | -16.24 | 4.0131 | 9.9042 | 14.437 | -72.57147 | 1.30757 | 1 |
| 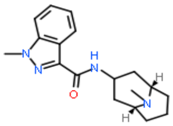 | 0 | 769 | C18H24N4O | -1.802 | 47 | 1 | -1.5538 | -6.332 | -19.11 | 0 | 13.316 | 22.575 | -125.0819 | 1.30925 | 1 |

| | | | | | | | | | | | | | | | |
|---|---|---|---|---|---|---|---|---|---|---|---|---|---|---|---|
| 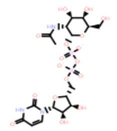 | 0 | 1948 | C17H27N3O17P2 | -1.786 | 64 | 15 | -13.621 | -4.2439 | -22.79 | 4.9926 | 32.596 | 33.13 | -175.1671 | 2.06563 | 1 |
| 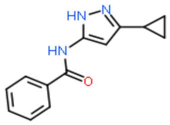 | 0 | 2368 | N5B | -1.774 | 30 | 2 | -3.2568 | -5.0593 | -19.09 | 4.1489 | 13.778 | 22.606 | -86.94244 | 1.32011 | 1 |
| 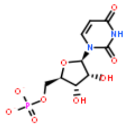 | 0 | 3329 | C9H13N2O9P | -1.766 | 32 | 6 | -7.9452 | -2.5922 | -12.1 | 0.5705 | 18.786 | 21.423 | -93.94173 | 1.15187 | 1 |
| 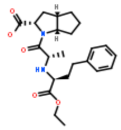 | 0 | 73 | 178 | -1.766 | 61 | 10 | -6.9598 | -6.6642 | -27.83 | 8.7523 | 20.55 | 32.543 | -157.6602 | 1.96077 | 1 |
| 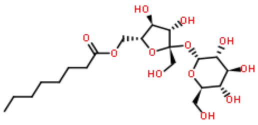 | 0 | 2871 | C20H36O12 | -1.759 | 68 | 19 | -7.3299 | -4.6064 | -29.27 | 10.027 | 22.462 | 22.543 | -78.28587 | 1.65733 | 1 |
| 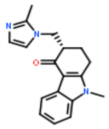 | 0 | 783 | 904 | -1.729 | 41 | 2 | -3.3844 | -5.7771 | -17.71 | 1.8851 | 16.394 | 20.889 | -127.725 | 1.62524 | 1 |
| 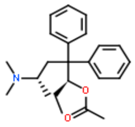 | 0 | 1096 | 1227 | -1.727 | 57 | 8 | -1.6114 | -6.4276 | -23.58 | 2.5348 | 13.35 | 21.071 | -90.59186 | 1.35787 | 1 |
| 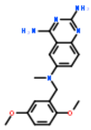 | 0 | 2163 | COG | -1.717 | 46 | 2 | -2.9644 | -6.5496 | -22.43 | 7.2912 | 21.623 | 16.373 | -126.5251 | 1.72556 | 1 |
| 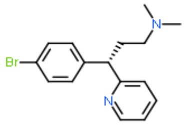 | 0 | 296 | 405 | -1.716 | 38 | 5 | -0.7038 | -6.0959 | -21.45 | 0.8549 | 12.3 | 19.488 | -110.4014 | 1.45622 | 1 |
| 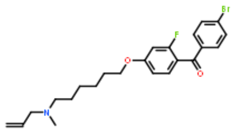 | 0 | 1777 | C23H27BrFNO2 | -1.713 | 55 | 10 | -2.148 | -7.8245 | -29.94 | 3.2894 | 18.243 | 26.134 | -133.2954 | 2.09748 | 1 |
| 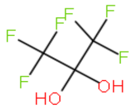 | 0 | 2621 | C3H2F6O2 | -1.707 | 13 | 2 | -3.7338 | -2.5411 | -10.16 | 0 | 11.765 | 14.519 | -69.46743 | 0.52588 | 1 |

| | | | | | | | | | | | | | | | |
|---|---|---|---|---|---|---|---|---|---|---|---|---|---|---|---|
| 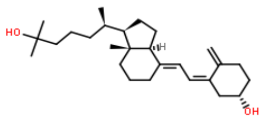 | 0 | 42 | C27H44O2 | -1.702 | 73 | 7 | -5.5755 | -8.0566 | -19.3 | 9.6896 | 20.007 | 18.314 | -128.74 | 2.09906 | 1 |
| 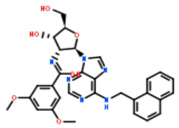 | 0 | 3007 | C30H30N6O6 | -1.698 | 72 | 8 | -5.2189 | -5.9675 | -27.16 | 8.1793 | 23.217 | 23.549 | -99.68449 | 2.2212 | 1 |
| 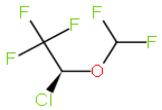 | 0 | 636 | 753 | -1.691 | 12 | 2 | -1.66 | -3.6028 | -10.16 | 0 | 7.8897 | 12.072 | -53.42771 | 0.60832 | 1 |
| 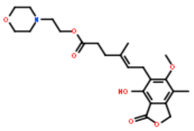 | 0 | 574 | 688 | -1.669 | 62 | 8 | -4.4228 | -6.4427 | -30.98 | 9.0606 | 21.327 | 28.158 | -140.0767 | 2.16224 | 1 |
| 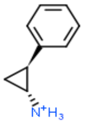 | 0 | 2385 | C9H12N | -1.653 | 22 | 1 | -1.9353 | -2.9761 | -13.91 | 0.9818 | 8.4252 | 17.641 | -47.50835 | 0.70716 | 1 |
| 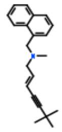 | 0 | 737 | C21H25N | -1.652 | 47 | 5 | -0.4404 | -6.901 | -25.11 | 2.4744 | 13.056 | 22.611 | -107.2813 | 1.46521 | 1 |
| 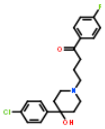 | 0 | 392 | 502 | -1.647 | 49 | 7 | -4.3154 | -5.8144 | -17.8 | 3.7986 | 17.174 | 17.587 | -135.0937 | 1.89242 | 1 |
| 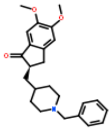 | 0 | 723 | 843 | -1.626 | 57 | 4 | -2.3923 | -7.8868 | -27.52 | 3.673 | 19.978 | 26.735 | -145.0557 | 1.90193 | 1 |
| 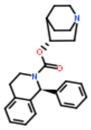 | 0 | 1385 | C23H26N2O2 | -1.609 | 53 | 3 | -2.3058 | -6.1088 | -21.36 | 5.4215 | 15.109 | 20.134 | -108.0965 | 1.53012 | 1 |
| 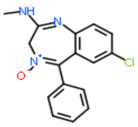 | 0 | 365 | C16H14ClN3O | -1.605 | 35 | 1 | -3.195 | -5.6382 | -17.15 | 3.8899 | 14.246 | 20.895 | -105.6921 | 1.47694 | 1 |
| 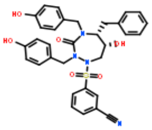 | 0 | 2824 | C32H30N4O6S | -1.59 | 73 | 9 | -4.369 | -7.2072 | -32.88 | 10.844 | 27.458 | 22.237 | -169.7362 | 2.11008 | 1 |

| | | | | | | | | | | | | | | | |
|---|---|---|---|---|---|---|---|---|---|---|---|---|---|---|---|
| 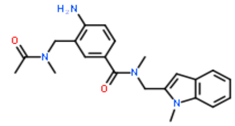 | 0 | 3193 | ZAM | -1.587 | 54 | 6 | -4.0186 | -6.3915 | -24.7 | 5.0258 | 17.446 | 27.071 | -118.7489 | 1.78135 | 1 |
| 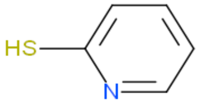 | 0 | 3005 | C5H5NS | -1.583 | 12 | 1 | -2.3287 | -2.3363 | -10.31 | 2.6682 | 10.438 | 9.3212 | -29.33501 | 0.46642 | 1 |
| 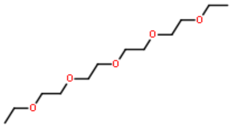 | 0 | 2084 | 6JZ | -1.57 | 43 | 14 | -5.3796 | -6.2683 | -17.02 | 4.0877 | 16.995 | 14.637 | -96.66103 | 1.61293 | 1 |
| 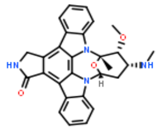 | 0 | 1772 | C28H26N4O3 | -1.564 | 61 | 2 | -2.2106 | -6.0821 | -22.17 | 9.1154 | 16.045 | 17.533 | -96.13557 | 1.49996 | 1 |
| 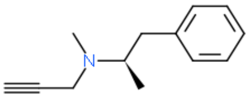 | 0 | 912 | C13H17N | -1.559 | 31 | 5 | -1.1716 | -5.4567 | -18.33 | 4.756 | 9.4058 | 15.876 | -78.46233 | 1.16941 | 1 |
| 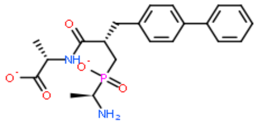 | 0 | 1821 | BIR | -1.54 | 54 | 10 | -11.537 | -5.3844 | -25.35 | 3.2876 | 28.673 | 41.113 | -152.4073 | 1.84862 | 1 |
| 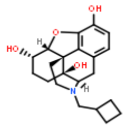 | 0 | 724 | C21H27NO4 | -1.535 | 53 | 4 | -3.87 | -4.9266 | -19.7 | 7.4107 | 15.97 | 18.836 | -105.333 | 1.26841 | 1 |
| 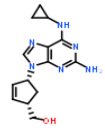 | 0 | 922 | m | -1.524 | 39 | 4 | -4.6741 | -5.6215 | -20.99 | 2.8072 | 23.304 | 20.454 | -100.1956 | 1.68161 | 1 |
| 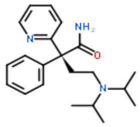 | 0 | 175 | C21H29N3O | -1.515 | 54 | 8 | -1.9316 | -6.2364 | -22.6 | 4.8979 | 13.753 | 18.323 | -74.66987 | 1.24816 | 1 |
| 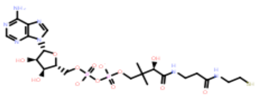 | 0 | 2852 | C21H35N7O13P2S | -1.514 | 77 | 20 | -14.866 | -5.7612 | -30.26 | 0 | 33.759 | 49.838 | -182.2435 | 2.21188 | 1 |
| 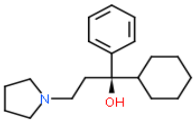 | 0 | 278 | 387 | -1.499 | 50 | 6 | 0 | -5.9328 | -22.79 | 0.6131 | 11.047 | 19.712 | -88.16843 | 1.10528 | 1 |

| | | | | | | | | | | | | | | | |
|---|---|---|---|---|---|---|---|---|---|---|---|---|---|---|---|
| 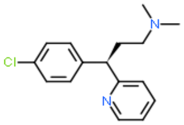 | 0 | 985 | 1114 | -1.497 | 38 | 5 | -1.7444 | -6.2957 | -22.17 | 4.9331 | 13.067 | 20.515 | -117.9731 | 1.3782 | 1 |
| 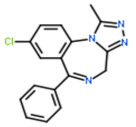 | 0 | 295 | 404 | -1.495 | 35 | 1 | -1.7839 | -5.724 | -22.97 | 1.3669 | 15.436 | 25.495 | -86.28509 | 1.14885 | 1 |
| 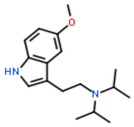 | 0 | 1247 | 1441 | -1.494 | 46 | 5 | -2.2368 | -5.4988 | -19.05 | 4.7719 | 12.071 | 18.154 | -81.42557 | 1.13141 | 1 |
| 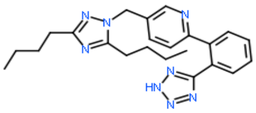 | 0 | 1173 | 1342 | -1.491 | 59 | 8 | -3.2455 | -7.1132 | -34.92 | 7.1647 | 22.106 | 31.406 | -150.9891 | 2.06592 | 1 |
| 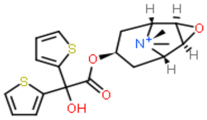 | 0 | 1217 | C19H22NO4S2 | -1.479 | 48 | 5 | -2.3632 | -5.8922 | -25.03 | 4.2119 | 16.05 | 24.113 | -110.61 | 1.65195 | 1 |
| 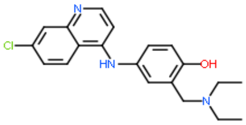 | 0 | 500 | 613 | -1.478 | 47 | 4 | -0.8503 | -7.3336 | -25.71 | 0.0976 | 17.306 | 24.03 | -132.9646 | 1.65084 | 1 |
| 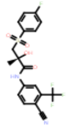 | 0 | 2630 | 198 | -1.464 | 43 | 5 | -0.8929 | -5.8853 | -26.1 | 0.9451 | 15.616 | 23.578 | -157.5702 | 1.62466 | 1 |
| 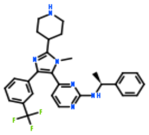 | 0 | 1543 | C28H29F3N6 | -1.452 | 66 | 3 | -1.9405 | -8.2041 | -31.46 | 8.9873 | 23.312 | 23.839 | -129.3674 | 1.85893 | 1 |
| 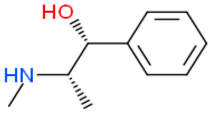 | 0 | 1188 | C10H15NO | -1.449 | 27 | 4 | -3.0348 | -4.0128 | -14.51 | 0 | 14.027 | 15.927 | -72.5825 | 1.00794 | 1 |
| 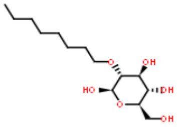 | 0 | 2834 | BGL | -1.449 | 48 | 13 | -6.4115 | -5.5299 | -19 | 3.0059 | 21.393 | 17.931 | -90.079 | 1.58047 | 1 |
| 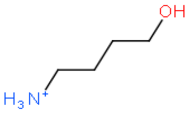 | 0 | 2269 | C4H12NO | -1.427 | 18 | 4 | -4.0441 | -1.8858 | -10.43 | 1.1597 | 10.457 | 14.487 | -26.34832 | 0.6122 | 1 |

| | | | | | | | | | | | | | | | |
|---|---|---|---|---|---|---|---|---|---|---|---|---|---|---|---|
| 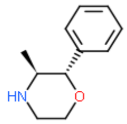 | 0 | 711 | 830 | -1.416 | 28 | 1 | -2.8324 | -4.288 | -14.51 | 3.111 | 12.868 | 16.691 | -99.60362 | 1.10253 | 1 |
| 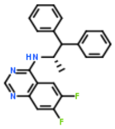 | 0 | 3324 | C23H19F2N3 | -1.41 | 47 | 4 | -0.9232 | -7.1192 | -25.85 | 5.7049 | 15.936 | 20.867 | -131.0919 | 1.47699 | 1 |
| 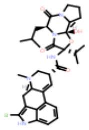 | 0 | 1070 | 1200 | -1.404 | 83 | 6 | -1.6855 | -8.5426 | -35.39 | 6.1326 | 24.002 | 28.326 | -175.2913 | 2.34137 | 1 |
| 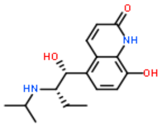 | 0 | 1190 | 1366 | -1.403 | 43 | 6 | -2.611 | -4.4142 | -21.68 | 3.2047 | 15.555 | 19.173 | -62.50089 | 0.95737 | 1 |
| 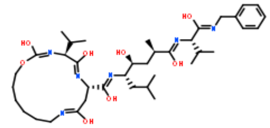 | 0 | 2117 | C38H62N6O8 | -1.382 | 114 | 21 | -2.7434 | -8.9236 | -41.51 | 4.2464 | 27.196 | 26.523 | -168.3258 | 2.70125 | 1 |
| 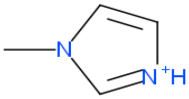 | 0 | 2391 | C4H7N2 | -1.375 | 13 | 0 | -0.8698 | -2.3206 | -9.382 | 0 | 5.4649 | 11.611 | -22.32639 | 0.60867 | 1 |
| 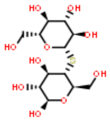 | 0 | 2943 | C12H22O10S | -1.375 | 45 | 12 | -12.261 | -3.2416 | -13.24 | 5.7402 | 29.778 | 18.303 | -124.2364 | 1.728 | 1 |
| 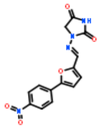 | 0 | 1089 | C14H10N4O5 | -1.37 | 33 | 2 | -4.6483 | -3.5828 | -18.79 | 0.2457 | 18.081 | 25.265 | -111.1262 | 1.36573 | 1 |
| 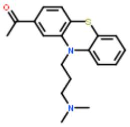 | 0 | 1407 | C19H22N2OS | -1.365 | 45 | 4 | -2.0174 | -5.9682 | -21.73 | 2.5643 | 14.047 | 22.716 | -114.5388 | 1.47597 | 1 |
| 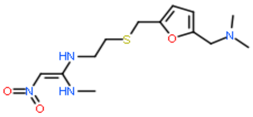 | 0 | 743 | 863 | -1.359 | 43 | 8 | 0 | -5.285 | -24.19 | 6.0489 | 10.745 | 14.976 | -95.97547 | 1.60018 | 1 |
| 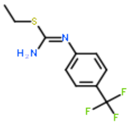 | 0 | 2687 | TFM | -1.347 | 27 | 1 | -1.2207 | -4.9821 | -19.59 | 0.8904 | 13.827 | 19.941 | -70.91264 | 1.30471 | 1 |

| | | | | | | | | | | | | | | | |
|---|---|---|---|---|---|---|---|---|---|---|---|---|---|---|---|
| 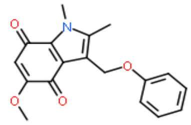 | 0 | 3275 | 340 | -1.336 | 40 | 3 | -1.9267 | -5.8422 | -23.02 | 2.4751 | 14.97 | 24.186 | -106.7333 | 1.31053 | 1 |
| 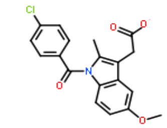 | 0 | 222 | C19H16ClNO4 | -1.317 | 40 | 4 | -3.6498 | -5.3683 | -19.46 | 5.32 | 15.32 | 21.053 | -118.1978 | 1.30685 | 1 |
| 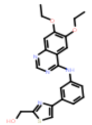 | 0 | 2551 | PFE | -1.3 | 52 | 4 | -4.4213 | -7.8754 | -30.24 | 5.3878 | 25.345 | 31.452 | -131.5988 | 1.9282 | 1 |
| 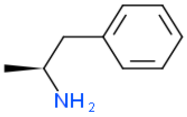 | 0 | 1372 | 1576 | -1.297 | 23 | 3 | -1.4177 | -3.7128 | -15.01 | 1.2788 | 10.892 | 13.711 | -58.77596 | 0.80638 | 1 |
| 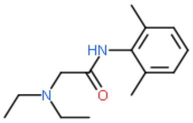 | 0 | 176 | 281 | -1.29 | 39 | 4 | -2.8887 | -5.875 | -17.77 | 1.6237 | 12.531 | 22.663 | -79.01441 | 1.4709 | 1 |
| 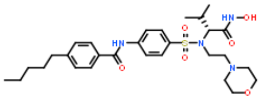 | 0 | 1422 | C29H42N4O6S | -1.264 | 82 | 14 | -3.4943 | -8.2795 | -30.8 | 5.7957 | 26.131 | 18.853 | -134.1964 | 2.81028 | 1 |
| 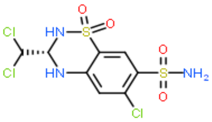 | 0 | 897 | 1021 | -1.261 | 28 | 3 | -1.6101 | -3.7739 | -25.67 | 3.1589 | 15.992 | 23.214 | -99.87647 | 1.63566 | 1 |
| 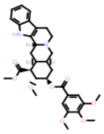 | 0 | 961 | 1089 | -1.253 | 80 | 3 | -2.1649 | -9.3118 | -33.11 | 9.0982 | 26.662 | 24.721 | -172.5529 | 2.47623 | 1 |
| 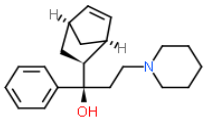 | 0 | 691 | C21H29NO | -1.247 | 52 | 6 | -1.4026 | -6.2573 | -23.5 | 2.8756 | 11.608 | 24.184 | -92.67725 | 1.29864 | 1 |
| 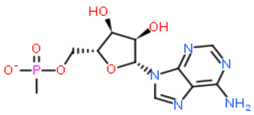 | 0 | 2503 | C11H16N5O6P | -1.215 | 38 | 6 | -9.7516 | -4.0886 | -21.11 | 3.4943 | 28.098 | 31.115 | -93.43424 | 1.38726 | 1 |
| 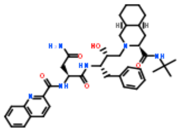 | 0 | 1101 | C38H50N6O5 | -1.213 | 99 | 13 | -6.002 | -8.2979 | -41.16 | 21.138 | 28.542 | 28.486 | -201.1134 | 2.56964 | 1 |

| | | | | | | | | | | | | | | | |
|---|---|---|---|---|---|---|---|---|---|---|---|---|---|---|---|
| 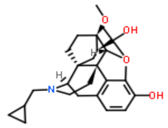 | 0 | 1344 | C26H35NO4 | -1.203 | 66 | 5 | -5.1086 | -6.2244 | -18.68 | 7.68 | 19.992 | 18.133 | -108.2626 | 1.33573 | 1 |
| 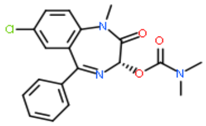 | 0 | 1292 | 1489 | -1.195 | 44 | 3 | -1.1135 | -6.7097 | -27.73 | 5.2386 | 17.876 | 23.482 | -149.7008 | 1.48447 | 1 |
| 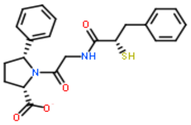 | 0 | 2389 | TI3 | -1.173 | 52 | 9 | -6.2416 | -5.0743 | -23.57 | 6.9152 | 21.011 | 24.665 | -128.562 | 1.87277 | 1 |
| 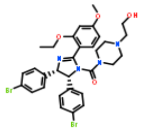 | 0 | 2574 | C31H34Br2N4O4 | -1.172 | 75 | 8 | -4.7585 | -7.5024 | -27.86 | 15.149 | 19.457 | 23.135 | -119.1315 | 2.11781 | 1 |
| 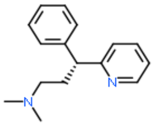 | 0 | 1412 | 1620 | -1.16 | 38 | 5 | -0.6952 | -5.569 | -22.39 | 3.2113 | 10.357 | 21.251 | -65.93456 | 0.98605 | 1 |
| 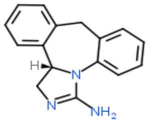 | 0 | 634 | 751 | -1.15 | 34 | 0 | -1.0501 | -4.226 | -19.39 | 0.6531 | 12.593 | 21.044 | -80.64145 | 0.89333 | 1 |
| 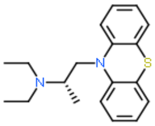 | 0 | 283 | 3290 | -1.145 | 46 | 5 | 0 | -6.2093 | -23.92 | 1.43 | 11.842 | 21.515 | -83.87867 | 1.24803 | 1 |
| 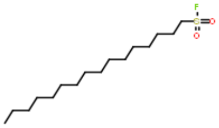 | 0 | 1796 | C16H33FO2S | -1.144 | 53 | 15 | 0 | -6.4506 | -23.9 | 6.4758 | 9.0844 | 11.188 | -97.40818 | 1.23197 | 1 |
| 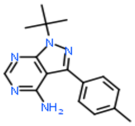 | 0 | 1586 | PP1 | -1.138 | 40 | 1 | -1.5626 | -5.9244 | -21.79 | 0.8792 | 16.362 | 23 | -106.9702 | 1.2515 | 1 |
| 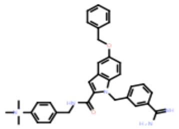 | 0 | 2013 | C34H36N5O2 | -1.132 | 77 | 7 | -5.0298 | -8.5357 | -34.02 | 12.036 | 27.565 | 29.139 | -168.6041 | 2.37249 | 1 |
| 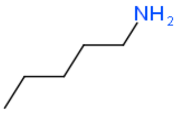 | 0 | 1805 | C5H13N | -1.129 | 19 | 4 | -1.8872 | -2.3326 | -10.87 | 0.7774 | 10.742 | 7.8051 | -9.327208 | 0.54999 | 1 |

| | | | | | | | | | | | | | | | |
|---|---|---|---|---|---|---|---|---|---|---|---|---|---|---|---|
| 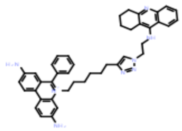 | 0 | 1974 | C42H45N8 | -1.117 | 95 | 11 | -3.486 | -8.6631 | -40.46 | 19.162 | 27.157 | 23.49 | -149.2565 | 2.4942 | 1 |
| 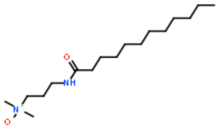 | 0 | 1520 | LDM | -1.104 | 57 | 14 | -2.148 | -6.7271 | -25.31 | 4.7617 | 14.617 | 18.071 | -134.8238 | 1.55988 | 1 |
| 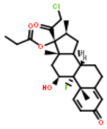 | 0 | 889 | C25H32ClFO5 | -1.101 | 64 | 5 | -0.386 | -7.288 | -21.48 | 1.577 | 18.03 | 13.283 | -124.0686 | 1.75031 | 1 |
| 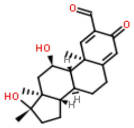 | 0 | 1365 | 1569 | -1.095 | 53 | 2 | -3.3093 | -4.6379 | -16.63 | 3.2275 | 19.558 | 13.955 | -120.9839 | 1.57134 | 1 |
| 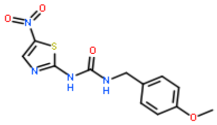 | 0 | 1714 | TMU | -1.094 | 33 | 5 | -3.1447 | -4.8733 | -24.87 | 1.1093 | 18.702 | 26.214 | -106.3188 | 1.49629 | 1 |
| 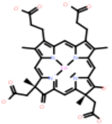 | 0 | 3133 | C34H32FeN4O10 | -1.03 | 77 | 10 | -8.3758 | -6.4684 | -24.21 | 6.6278 | 28.317 | 26.505 | -153.0116 | 2.1161 | 1 |
| 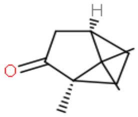 | 0 | 1527 | C10H16O | -1.005 | 27 | 0 | -0.8312 | -3.3364 | -10.64 | 0.3182 | 7.4346 | 12.44 | -66.61861 | 0.8923 | 1 |
| 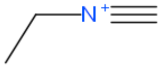 | 0 | 3068 | C3H6N | -1.001 | 10 | 0 | 0 | -2.4849 | -6.387 | 0.0179 | 4.4404 | 5.6209 | -7.384794 | 0.39045 | 1 |
| 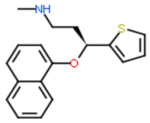 | 0 | 366 | 476 | -0.99 | 40 | 5 | -1.6801 | -5.7747 | -23.03 | 0.5961 | 15.913 | 22.519 | -101.3994 | 1.54447 | 1 |
| 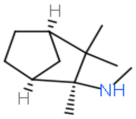 | 0 | 544 | 657 | -0.987 | 33 | 1 | -0.8021 | -3.4567 | -10.73 | 0.3559 | 9.0083 | 9.8716 | -39.80745 | 0.78217 | 1 |
| 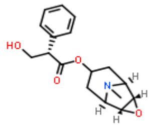 | 0 | 630 | ChEBI | -0.957 | 43 | 5 | -5.9749 | -5.0993 | -14.49 | 6.4888 | 15.673 | 20.332 | -119.638 | 1.47429 | 1 |

| | | | | | | | | | | | | | | | |
|---|---|---|---|---|---|---|---|---|---|---|---|---|---|---|---|
| 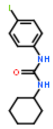 | 0 | 1790 | CIU | -0.957 | 34 | 4 | -1.8378 | -5.7047 | -22.9 | 0.7031 | 14.399 | 25.447 | -99.27119 | 1.4434 | 1 |
| 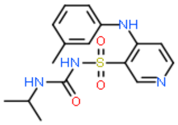 | 0 | 109 | 214 | -0.949 | 44 | 5 | -2.3322 | -5.4859 | -28.32 | 8.5268 | 16.733 | 24.673 | -103.1422 | 1.65482 | 1 |
| 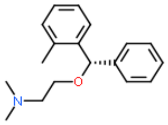 | 0 | 1043 | 1173 | -0.94 | 43 | 6 | -1.3213 | -6.6191 | -24.22 | 1.3676 | 14.216 | 24.212 | -111.8631 | 1.43079 | 1 |
| 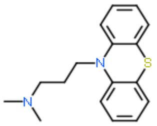 | 0 | 310 | 420 | -0.926 | 40 | 4 | -1.1314 | -5.7749 | -24.82 | 4.9106 | 11.923 | 24.749 | -113.1858 | 1.31483 | 1 |
| 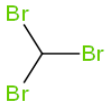 | 0 | 2744 | CHBr3 | -0.918 | 5 | 0 | 0 | -3.3441 | -11.14 | 0.0027 | 6.2399 | 11.781 | -16.80815 | 0.52237 | 1 |
| 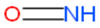 | 0 | 325 | NO | -0.907 | 2 | 0 | 0 | 0.05126 | -5.512 | 0.0002 | 4.499 | 1.8139 | -9.853142 | -0.0985 | 1 |
| 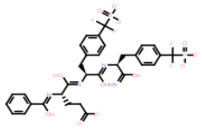 | 0 | 3147 | C32H34F4N4O12P2 | -0.895 | 83 | 22 | -10.366 | -7.6455 | -39.7 | 23.601 | 34.973 | 25.088 | -194.0784 | 2.6265 | 1 |
| 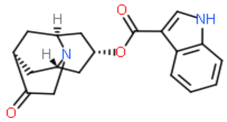 | 0 | 640 | C19H20N2O3 | -0.89 | 44 | 1 | -1.8531 | -5.4791 | -19.19 | 4.1704 | 13.399 | 20.461 | -115.3359 | 1.48314 | 1 |
| 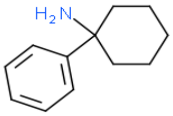 | 0 | 1307 | 1506 | -0.886 | 30 | 2 | -2.682 | -4.2221 | -13.93 | 1.09 | 12.966 | 16.785 | -85.0422 | 1.07782 | 1 |
| 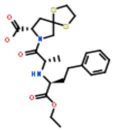 | 0 | 1177 | 1348 | -0.882 | 60 | 10 | -8.3032 | -6.644 | -27.66 | 15.556 | 21.054 | 31.993 | -172.6202 | 2.10968 | 1 |
| 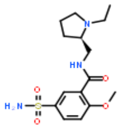 | 0 | 282 | 391 | -0.864 | 46 | 5 | -3.9574 | -5.6415 | -23.58 | 4.0786 | 18.771 | 25.735 | -123.1067 | 1.63303 | 1 |

| | | | | | | | | | | | | | | | |
|---|---|---|---|---|---|---|---|---|---|---|---|---|---|---|---|
| 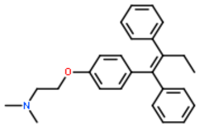 | 0 | 561 | 675 | -0.856 | 57 | 6 | -2.6934 | -7.7611 | -26.47 | 5.289 | 17.262 | 27.13 | -154.6248 | 2.13647 | 1 |
| 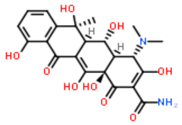 | 0 | 482 | C22H24N2O9 | -0.853 | 57 | 4 | -3.2209 | -5.1811 | -24.92 | 5.5681 | 24.864 | 17.121 | -167.3582 | 1.77571 | 1 |
| 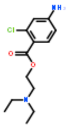 | 0 | 1031 | 1161 | -0.851 | 37 | 5 | -2.4076 | -5.9352 | -21.42 | 3.3051 | 15.368 | 21.411 | -76.72828 | 1.35707 | 1 |
| 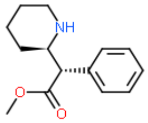 | 0 | 312 | 422 | -0.848 | 36 | 3 | -2.5153 | -5.026 | -16.33 | 4.3761 | 14.193 | 15.361 | -91.59744 | 1.27899 | 1 |
| 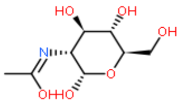 | 0 | 3381 | C8H15NO6 | -0.847 | 30 | 7 | -8.8366 | -2.8059 | -13.15 | 0.9495 | 21.552 | 23.644 | -74.08126 | 1.15932 | 1 |
| 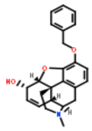 | 0 | 1369 | 1573 | -0.841 | 53 | 3 | -1.3091 | -6.3728 | -26.76 | 0 | 19.085 | 26.346 | -141.8643 | 1.5028 | 1 |
| 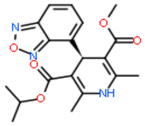 | 0 | 165 | 270 | -0.84 | 48 | 2 | -3.4049 | -5.5211 | -22.39 | 4.3953 | 20.07 | 22.691 | -108.7804 | 1.53969 | 1 |
| 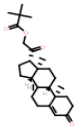 | 0 | 1005 | C26H38O4 | -0.839 | 68 | 4 | -4.1799 | -7.3395 | -22.34 | 5.0359 | 18.661 | 26.638 | -161.8727 | 1.67178 | 1 |
| 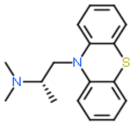 | 0 | 941 | 1069 | -0.836 | 40 | 3 | 0 | -5.4225 | -21.58 | 1.5321 | 11.357 | 19.902 | -81.97546 | 1.03769 | 1 |
| 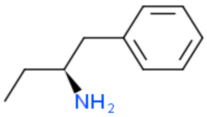 | 0 | 2668 | C10H15N | -0.835 | 26 | 4 | -1.299 | -4.0314 | -16.1 | 0.9904 | 12.016 | 14.008 | -51.88544 | 0.87853 | 1 |
| 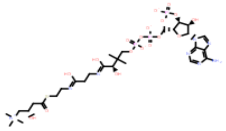 | 0 | 2246 | C28H49N8O18P3S | -0.832 | 104 | 28 | -12.282 | -7.946 | -36.54 | 0 | 40.86 | 37.365 | -189.6677 | 2.89432 | 1 |

| | | | | | | | | | | | | | | | |
|---|---|---|---|---|---|---|---|---|---|---|---|---|---|---|---|
| 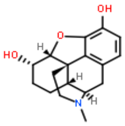 | 0 | 1361 | 1565 | -0.821 | 42 | 1 | -3.125 | -3.9951 | -15.16 | 0.8849 | 14.462 | 19.614 | -94.9489 | 1.13554 | 1 |
| 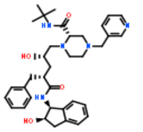 | 0 | 119 | 224 | -0.813 | 92 | 14 | -3.5073 | -10.023 | -36.28 | 16.201 | 22.383 | 24.463 | -197.5004 | 2.40988 | 1 |
| 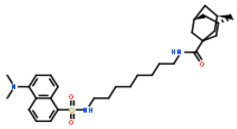 | 0 | 2723 | C31H45N3O3S | -0.809 | 83 | 12 | -3.2116 | -7.8675 | -28.28 | 10.005 | 17.495 | 22.038 | -160.6818 | 2.21535 | 1 |
| 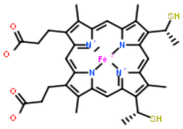 | 0 | 2994 | C34H36FeN4O4S2 | -0.804 | 79 | 10 | -11.895 | -7.1945 | -31.43 | 19.74 | 25.62 | 43.157 | -140.948 | 2.21105 | 1 |
| 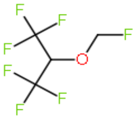 | 0 | 1105 | 1236 | -0.798 | 15 | 3 | -1.1243 | -3.5788 | -8.359 | 0 | 6.3163 | 9.4942 | -55.98042 | 0.41884 | 1 |
| 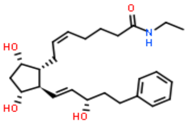 | 0 | 784 | C25H37NO4 | -0.783 | 67 | 15 | -5.5891 | -6.6944 | -31.42 | 11.916 | 21.376 | 25.874 | -127.106 | 1.99432 | 1 |
| 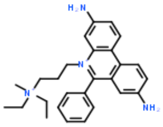 | 0 | 1920 | C27H34N4 | -0.782 | 65 | 7 | -3.5034 | -8.1387 | -25.1 | 2.9769 | 16.294 | 30.953 | -146.4421 | 1.46294 | 1 |
| 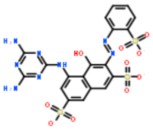 | 0 | 3138 | C19H16N8O10S3 | -0.78 | 53 | 3 | -12.355 | -5.3015 | -26.7 | 15.464 | 37.685 | 32.406 | -131.1226 | 1.6828 | 1 |
| 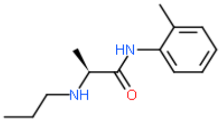 | 0 | 633 | 750 | -0.775 | 36 | 4 | -2.0972 | -5.4995 | -18.35 | 2.2189 | 13.182 | 19.625 | -83.21724 | 1.39433 | 1 |
| 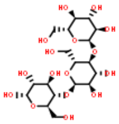 | 0 | 2955 | C18H32O16 | -0.77 | 66 | 18 | -7.9665 | -4.4174 | -24.86 | 2.2139 | 29.298 | 19.847 | -155.5184 | 1.88357 | 1 |
| 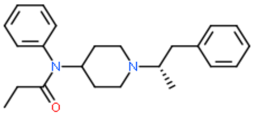 | 0 | 1353 | 1557 | -0.76 | 56 | 7 | -0.8538 | -7.8427 | -25.24 | 5.5488 | 16.703 | 17.933 | -130.5308 | 1.82816 | 1 |

| | | | | | | | | | | | | | | | |
|---|---|---|---|---|---|---|---|---|---|---|---|---|---|---|---|
| 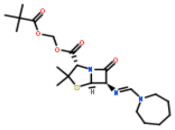 | 0 | 1398 | C21H33N3O5S | -0.738 | 63 | 6 | -5.7858 | -6.8013 | -19.8 | 4.9711 | 23.086 | 21.763 | -157.1515 | 2.14223 | 1 |
| 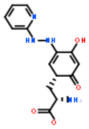 | 0 | 1426 | C14H16N4O4 | -0.738 | 37 | 5 | -6.8841 | -3.3254 | -19 | 1.8901 | 26.132 | 21.469 | -58.37605 | 1.50523 | 1 |
| 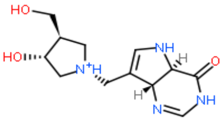 | 0 | 3207 | C12H19N4O3 | -0.736 | 38 | 5 | -5.6175 | -3.3619 | -18.12 | 3.3026 | 21.056 | 19.813 | -107.3486 | 1.50578 | 1 |
| 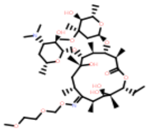 | 0 | 661 | C41H76N2O15 | -0.733 | 134 | 17 | -5.6222 | -11.382 | -32.61 | 7.8544 | 27.205 | 27.888 | -166.7361 | 3.16519 | 1 |
| 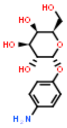 | 0 | 2923 | C12H17NO6 | -0.73 | 36 | 6 | -4.1636 | -3.9205 | -20.87 | 3.7829 | 18.339 | 20.73 | -89.48295 | 1.22957 | 1 |
| 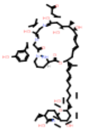 | 0 | 3064 | C60H91N5O13 | -0.726 | 169 | 21 | -3.2756 | -11.836 | -47.97 | 5.6328 | 34.757 | 32.452 | -272.3997 | 3.86614 | 1 |
| 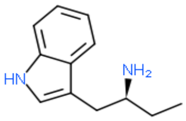 | 0 | 1342 | 1546 | -0.721 | 30 | 4 | -2.0732 | -4.103 | -18.67 | 2.5816 | 14.918 | 16.368 | -75.50617 | 1.13228 | 1 |
| 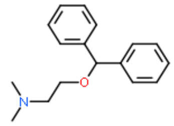 | 0 | 947 | 1075 | -0.713 | 40 | 6 | -1.3467 | -6.3395 | -22.81 | 1.3952 | 14.084 | 22.354 | -104.4884 | 1.40639 | 1 |
| 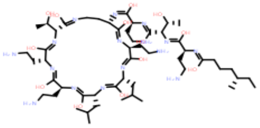 | 0 | 684 | C52H98N16O13 | -0.708 | 179 | 46 | -20.212 | -10.866 | -37.91 | 9.8919 | 50.411 | 36.779 | -236.6575 | 4.93073 | 1 |
| 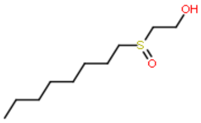 | 0 | 2151 | OES | -0.687 | 35 | 10 | -2.7522 | -5.2307 | -17.03 | 1.0672 | 12.588 | 16.258 | -85.10632 | 1.4959 | 1 |
| 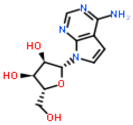 | 0 | 2854 | C11H14N4O4 | -0.678 | 33 | 5 | -5.5585 | -3.2104 | -18.59 | 4.4019 | 21.361 | 18.887 | -64.73761 | 0.91813 | 1 |

| | | | | | | | | | | | | | | | | |
|---|---|---|---|---|---|---|---|---|---|---|---|---|---|---|---|---|
| 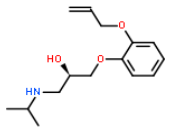 | 0 | 1376 | 1580 | -0.678 | 42 | 8 | -1.674 | -4.9895 | -22.53 | 3.8511 | 11.923 | 20.269 | -44.1947 | 1.16785 | 1 |
| 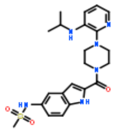 | 0 | 590 | 705 | -0.675 | 60 | 4 | -3.8907 | -7.0666 | -22.15 | 7.605 | 20.234 | 21.086 | -149.0692 | 2.26928 | 1 |
| 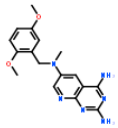 | 0 | 2309 | PRD | -0.674 | 45 | 2 | -2.4552 | -6.3339 | -27.08 | 1.7338 | 25.232 | 23.789 | -100.0765 | 1.41303 | 1 |
| 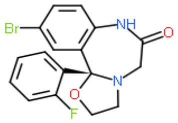 | 0 | 1279 | 1476 | -0.67 | 37 | 1 | -0.8916 | -5.4422 | -18.61 | 2.9393 | 13.046 | 17.718 | -81.8487 | 1.09043 | 1 |
| 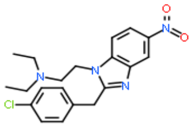 | 0 | 1319 | 1523 | -0.652 | 50 | 8 | 0 | -7.196 | -29.37 | 1.4576 | 14.096 | 26.205 | -126.9214 | 1.72646 | 1 |
| 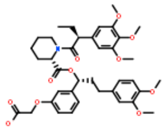 | 0 | 1508 | C38H47NO11 | -0.638 | 96 | 12 | -10.391 | -9.3553 | -38.89 | 33.088 | 33.459 | 28.28 | -228.5976 | 2.815 | 1 |
| 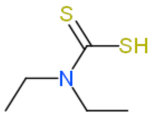 | 0 | 2250 | C5H11NS2 | -0.627 | 19 | 4 | -1.2843 | -2.652 | -13.13 | 1.4262 | 10.236 | 10 | -44.38139 | 0.83162 | 1 |
| 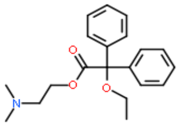 | 0 | 1264 | 1461 | -0.627 | 49 | 8 | -1.9986 | -6.7695 | -21.54 | 2.5662 | 16.985 | 17.309 | -107.3028 | 1.62759 | 1 |
| 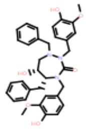 | 0 | 2509 | C34H37N3O6 | -0.619 | 80 | 9 | -1.9737 | -7.4666 | -32.04 | 10.284 | 21.848 | 20.398 | -171.3467 | 2.63071 | 1 |
| 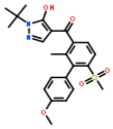 | 0 | 2553 | C23H26N2O5S | -0.61 | 57 | 2 | -1.0755 | -7.608 | -29.76 | 5.9987 | 20.326 | 25.643 | -166.2406 | 1.84198 | 1 |
| 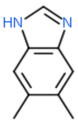 | 0 | 2316 | C9H10N2 | -0.61 | 21 | 0 | -1.3473 | -3.9724 | -12.34 | 1.0852 | 10.233 | 14.35 | -48.3268 | 0.80865 | 1 |

| | | | | | | | | | | | | | | | |
|---|---|---|---|---|---|---|---|---|---|---|---|---|---|---|---|
| 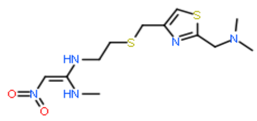 | 0 | 472 | C12H21N5O2S2 | -0.6 | 42 | 8 | 0 | -5.127 | -24.83 | 5.2861 | 11.741 | 16.501 | -70.7532 | 1.29556 | 1 |
| 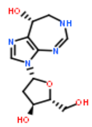 | 0 | 439 | m | -0.594 | 35 | 5 | -8.0583 | -3.1249 | -18.59 | 5.0101 | 26.091 | 22.588 | -95.43863 | 1.37242 | 1 |
| 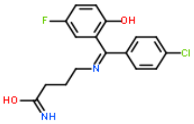 | 0 | 718 | C17H16ClFN2O2 | -0.571 | 39 | 6 | -2.7796 | -5.5777 | -22.81 | 0.0737 | 18.446 | 23.531 | -108.738 | 1.43466 | 1 |
| 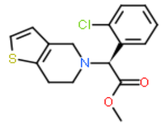 | 0 | 641 | C16H16ClNO2S | -0.567 | 37 | 3 | -0.9837 | -6.2589 | -24.75 | 1.6507 | 14.724 | 25.8 | -108.6586 | 1.44706 | 1 |
| 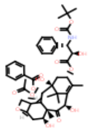 | 0 | 1117 | 1248 | -0.552 | 111 | 14 | -1.4232 | -9.3918 | -37.39 | 3.7717 | 25.313 | 25.882 | -154.2546 | 2.71197 | 1 |
| 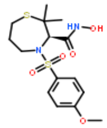 | 0 | 2090 | MM3 | -0.549 | 46 | 5 | -1.1906 | -5.5829 | -22.68 | 3.1134 | 13.336 | 21.233 | -102.1437 | 1.41621 | 1 |
| 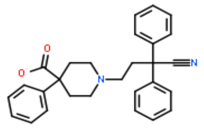 | 0 | 1303 | 1501 | -0.549 | 59 | 7 | -7.2935 | -6.817 | -18.82 | 8.7272 | 17.198 | 28.715 | -156.321 | 1.88179 | 1 |
| 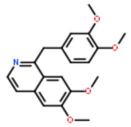 | 0 | 984 | 1113 | -0.549 | 46 | 2 | -3.2247 | -7.0995 | -24.12 | 6.0264 | 18.413 | 27.06 | -136.8984 | 1.49657 | 1 |
| 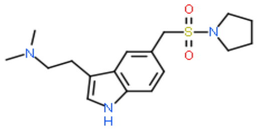 | 0 | 797 | 918 | -0.539 | 48 | 6 | -0.9679 | -5.3 | -20.76 | 5.9909 | 13.9 | 13.033 | -125.0741 | 1.72173 | 1 |
| 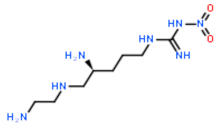 | 0 | 1788 | C8H21N7O2 | -0.523 | 38 | 13 | -6.9853 | -2.3351 | -18.16 | 8.4171 | 17.424 | 16.605 | -38.83265 | 0.86868 | 1 |
| 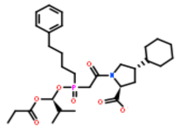 | 0 | 382 | C30H46NO7P | -0.492 | 84 | 15 | -8.4902 | -8.4287 | -33.06 | 13.595 | 24.844 | 36.227 | -172.4492 | 2.44623 | 1 |

| | | | | | | | | | | | | | | | |
|---|---|---|---|---|---|---|---|---|---|---|---|---|---|---|---|
| 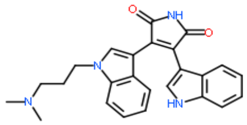 | 0 | 3416 | BI1 | -0.483 | 55 | 4 | -2.2318 | -5.2527 | -25.03 | 8.0397 | 17.82 | 19.88 | -86.54737 | 1.40601 | 1 |
| 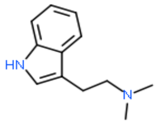 | 0 | 1291 | 1488 | -0.478 | 30 | 3 | -1.8861 | -4.9208 | -18.27 | 5.2549 | 11.137 | 19.017 | -97.80687 | 1.15876 | 1 |
| 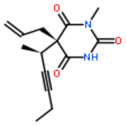 | 0 | 364 | 474 | -0.473 | 37 | 4 | -1.1104 | -4.5825 | -19.01 | 0.5447 | 14.111 | 16.827 | -82.42427 | 1.03109 | 1 |
| 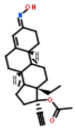 | 0 | 835 | C23H31NO3 | -0.469 | 58 | 3 | -3.7229 | -5.5551 | -19.75 | 3.3239 | 17.274 | 23.744 | -77.17599 | 1.55994 | 1 |
| 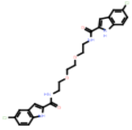 | 0 | 1847 | C24H24Cl2N4O4 | -0.468 | 58 | 9 | -3.8529 | -6.2802 | -31.53 | 9.5314 | 22.479 | 25.813 | -178.0082 | 2.36436 | 1 |
| 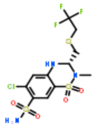 | 0 | 1158 | 1324 | -0.426 | 38 | 5 | -0.7365 | -4.8913 | -25.23 | 1.3061 | 15.659 | 21.85 | -113.3189 | 1.99338 | 1 |
| 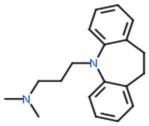 | 0 | 348 | 458 | -0.422 | 45 | 4 | -1.1993 | -6.3179 | -24.26 | 7.2679 | 10.64 | 24.792 | -124.282 | 1.29476 | 1 |
| 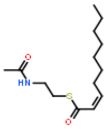 | 0 | 3448 | DAC | -0.419 | 43 | 9 | -2.6135 | -6.03 | -22.79 | 3.2763 | 17.331 | 19.173 | -93.87263 | 1.6591 | 1 |
| 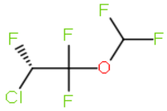 | 0 | 123 | 228 | -0.399 | 12 | 3 | -1.2083 | -3.4513 | -8.042 | 0.8211 | 6.5249 | 8.8265 | -64.96735 | 0.55701 | 1 |
| 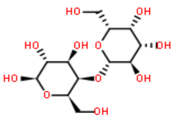 | 0 | 2455 | C12H22O11 | -0.387 | 45 | 12 | -10.834 | -2.8851 | -15.77 | 8.0808 | 26.132 | 19.813 | -118.6317 | 1.59383 | 1 |
| 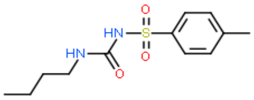 | 0 | 995 | 1124 | -0.369 | 36 | 7 | -1.7867 | -4.7338 | -23.56 | 1.2394 | 15.377 | 21.821 | -96.11662 | 1.53507 | 1 |

| | | | | | | | | | | | | | | | |
|---|---|---|---|---|---|---|---|---|---|---|---|---|---|---|---|
| 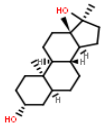 | 0 | 1282 | C20H34O2 | -0.368 | 56 | 2 | -2.0258 | -5.0025 | -15.91 | 1.8231 | 13.349 | 17.751 | -108.0114 | 1.20904 | 1 |
| 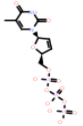 | 0 | 2587 | C10H15N2O13P3 | -0.359 | 39 | 8 | -13.141 | -3.1065 | -24.17 | 7.5255 | 25.258 | 46.787 | -82.1366 | 1.30162 | 1 |
| 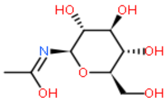 | 0 | 2062 | C8H15NO6 | -0.344 | 30 | 7 | -4.3273 | -2.2401 | -14.38 | 2.8546 | 17.391 | 11.4 | -23.25715 | 0.8453 | 1 |
| 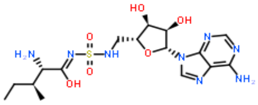 | 0 | 1538 | C16H26N8O6S | -0.343 | 57 | 12 | -6.1064 | -4.8682 | -30.85 | 7.878 | 30.537 | 21.795 | -133.6829 | 1.85013 | 1 |
| 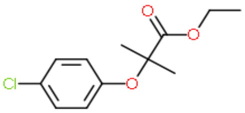 | 0 | 523 | 636 | -0.339 | 31 | 4 | -1.1688 | -5.2347 | -18.84 | 2.1238 | 12.738 | 17.783 | -66.78017 | 1.33468 | 1 |
| 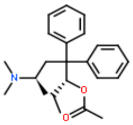 | 0 | 1351 | 1555 | -0.338 | 57 | 8 | 0 | -6.29 | -24.35 | 0.4244 | 12.322 | 21.11 | -104.1417 | 1.22919 | 1 |
| 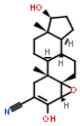 | 0 | 980 | C20H27NO3 | -0.326 | 51 | 1 | -3.1746 | -4.7693 | -15.47 | 0.4771 | 20.036 | 15.842 | -101.6248 | 1.36009 | 1 |
| 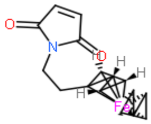 | 0 | 1489 | C16H15FeNO2 | -0.318 | 35 | 3 | -6.5289 | -4.726 | -17.27 | 2.9251 | 12.803 | 35.571 | -74.44012 | 1.25104 | 1 |
| 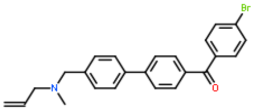 | 0 | 2915 | C24H22BrNO | -0.314 | 49 | 5 | -1.9631 | -7.7964 | -27.13 | 3.8521 | 17.79 | 27.805 | -148.8394 | 1.97186 | 1 |
| 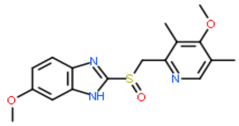 | 0 | 231 | 338 | -0.311 | 43 | 3 | -1.4016 | -6.5361 | -27.51 | 4.2476 | 19.297 | 24.708 | -99.66976 | 1.67747 | 1 |
| 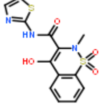 | 0 | 695 | m | -0.294 | 36 | 0 | -1.5832 | -4.8039 | -18.38 | 4.9626 | 12.875 | 19.203 | -56.28563 | 1.35561 | 1 |

| | | | | | | | | | | | | | | | |
|---|---|---|---|---|---|---|---|---|---|---|---|---|---|---|---|
| 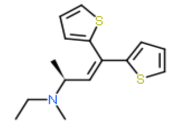 | 0 | 1271 | 1468 | -0.271 | 37 | 3 | -0.6881 | -5.6221 | -16.62 | 1.766 | 10.459 | 16.901 | -44.46312 | 1.06497 | 1 |
| 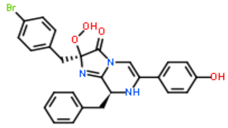 | 0 | 1768 | C26H22BrN3O4 | -0.267 | 56 | 6 | -4.1605 | -6.9386 | -30.24 | 13.423 | 23.888 | 23.597 | -147.1629 | 2.19229 | 1 |
| 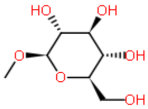 | 0 | 1434 | C7H14O6 | -0.233 | 27 | 6 | -4.5893 | -2.416 | -12.77 | 2.1675 | 14.022 | 15.676 | -50.32944 | 0.79839 | 1 |
| 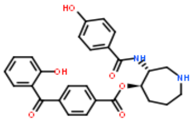 | 0 | 1705 | C27H26N2O6 | -0.227 | 61 | 2 | -4.5839 | -6.5611 | -25.98 | 6.5288 | 28.323 | 23.318 | -143.6742 | 2.06128 | 1 |
| 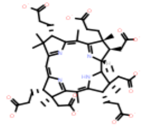 | 0 | 2193 | C45H60N4O14 | -0.217 | 116 | 18 | -10.567 | -6.7207 | -26.98 | 12.027 | 34.562 | 21.313 | -169.363 | 2.8195 | 1 |
| 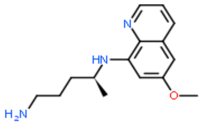 | 0 | 959 | 1087 | -0.194 | 40 | 5 | -2.2172 | -5.2874 | -20.61 | 2.3411 | 17.283 | 18.492 | -63.10453 | 1.24604 | 1 |
| 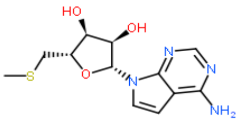 | 0 | 2631 | MTH | -0.194 | 36 | 5 | -5.9432 | -4.3637 | -18.03 | 4.0921 | 22.834 | 20.018 | -72.08523 | 1.07738 | 1 |
| 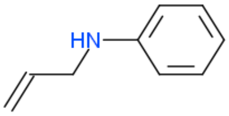 | 0 | 2572 | C9H11N | -0.19 | 21 | 3 | -0.631 | -3.972 | -15.3 | 0.6166 | 9.4524 | 15.317 | -50.89936 | 0.98111 | 1 |
| 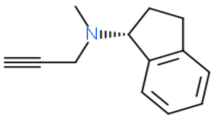 | 0 | 1960 | RMA | -0.189 | 29 | 3 | -0.7515 | -5.2516 | -15.19 | 2.6595 | 8.7961 | 15.797 | -59.94499 | 0.99683 | 1 |
| 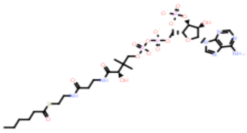 | 0 | 2291 | m | -0.183 | 97 | 25 | -10.935 | -6.2498 | -35.73 | 26.024 | 34.979 | 15.982 | -201.919 | 4.01692 | 1 |
| 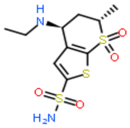 | 0 | 749 | C10H16N2O4S3 | -0.182 | 35 | 4 | -1.506 | -4.1192 | -21.25 | 3.6877 | 14.459 | 18.454 | -102.0533 | 1.47769 | 1 |

| | | | | | | | | | | | | | | | |
|---|---|---|---|---|---|---|---|---|---|---|---|---|---|---|---|
| 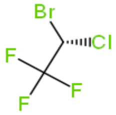 | 0 | 1029 | C2HBrClF3 | -0.179 | 8 | 1 | 0 | -3.3253 | -9.84 | 0 | 5.6674 | 10.664 | -27.06801 | 0.42637 | 1 |
| 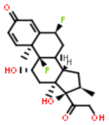 | 0 | 550 | C22H28F2O5 | -0.159 | 57 | 5 | -2.3862 | -4.8927 | -20.95 | 0.9318 | 21.365 | 15.948 | -79.17664 | 1.47108 | 1 |
| 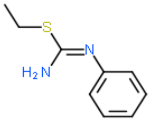 | 0 | 3350 | PTU | -0.15 | 24 | 2 | -2.1352 | -3.9468 | -16.84 | 6.5839 | 13.229 | 14.651 | -61.90038 | 1.0613 | 1 |
| 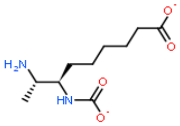 | 0 | 3274 | C10H20N2O4 | -0.137 | 34 | 10 | -11.07 | -3.2574 | -13.7 | 2.0291 | 21.45 | 30.978 | -110.7163 | 1.34526 | 1 |
| 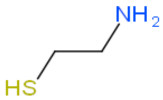 | 0 | 727 | 847 | -0.124 | 11 | 3 | -2.7001 | -1.0695 | -8.294 | 0.3104 | 8.6372 | 10.991 | -20.79386 | 0.54359 | 1 |
| 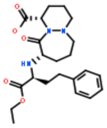 | 0 | 1172 | 1340 | -0.104 | 60 | 8 | -1.6203 | -7.1325 | -25.54 | 6.9455 | 18.908 | 16.917 | -137.4546 | 2.01106 | 1 |
| 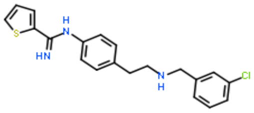 | 0 | 3116 | ARR | -0.079 | 45 | 5 | -2.5292 | -6.5355 | -28.36 | 6.2941 | 17.176 | 29.392 | -98.94765 | 1.85673 | 1 |
| 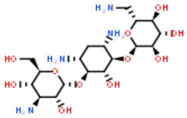 | 0 | 1042 | C18H36N4O11 | -0.078 | 69 | 17 | -9.5029 | -4.0812 | -23.51 | 2.1423 | 32.228 | 22.046 | -143.2727 | 2.02482 | 1 |
| 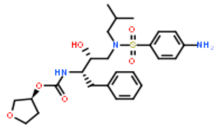 | 0 | 587 | 701 | -0.061 | 70 | 13 | -4.5828 | -8.0722 | -32.35 | 6.979 | 25.841 | 27.156 | -144.8233 | 1.80881 | 1 |
| 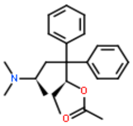 | 0 | 1318 | 1522 | -0.035 | 57 | 8 | -1.591 | -6.4007 | -21.92 | 2.2316 | 13.218 | 21.438 | -83.08171 | 1.30754 | 1 |
| 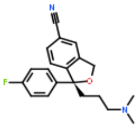 | 0 | 110 | 215 | -0.032 | 45 | 5 | -2.0583 | -5.9765 | -21.18 | 3.1898 | 14.644 | 21.918 | -102.402 | 1.31521 | 1 |

| | | | | | | | | | | | | | | | |
|---|---|---|---|---|---|---|---|---|---|---|---|---|---|---|---|
| 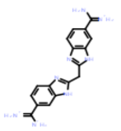 | 0 | 1491 | C17H19N8 | 0.0026 | 44 | 2 | -5.4586 | -4.299 | -25.73 | 0 | 26.297 | 32.574 | -90.7906 | 1.68759 | 1 |
| 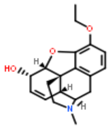 | 0 | 1269 | 1466 | 0.0073 | 46 | 2 | -1.7038 | -5.4766 | -18.6 | 2.1114 | 14.944 | 19.563 | -121.5826 | 1.2102 | 1 |
| 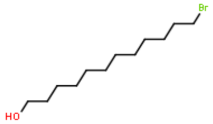 | 0 | 2342 | C12H25BrO | 0.0123 | 39 | 12 | -3.7937 | -6.1805 | -16.81 | 1.5218 | 16.354 | 15.422 | -97.27039 | 1.51225 | 1 |
| 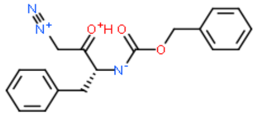 | 0 | 2476 | C18H18N3O3 | 0.0212 | 42 | 7 | -4.4933 | -4.8872 | -22.46 | 0 | 22.687 | 24.102 | -109.7373 | 1.58942 | 1 |
| 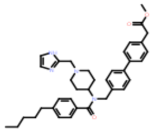 | 0 | 2235 | C37H44N4O3 | 0.0323 | 88 | 12 | -4.0698 | -8.9539 | -34.21 | 10.478 | 24.067 | 28.794 | -206.5846 | 2.62102 | 1 |
| 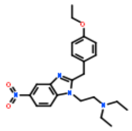 | 0 | 1265 | 1462 | 0.0348 | 57 | 9 | -0.6607 | -5.1273 | -25.4 | 6.8819 | 12.233 | 17.932 | -61.39774 | 1.48098 | 1 |
| 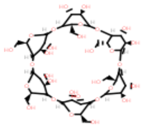 | 0 | 1677 | C36H60O30 | 0.0466 | 126 | 24 | -10.952 | -6.4807 | -28.01 | 2.883 | 40.443 | 20.669 | -200.7305 | 2.72221 | 1 |
| 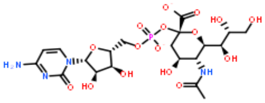 | 0 | 2216 | m | 0.0529 | 70 | 17 | -13.891 | -4.4302 | -31.2 | 0 | 41.914 | 42.008 | -188.981 | 2.29532 | 1 |
| 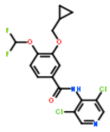 | 0 | 1446 | 1656 | 0.0531 | 40 | 3 | -2.8534 | -6.2075 | -19.58 | 3.4599 | 18.703 | 19.86 | -95.24051 | 1.88517 | 1 |
| 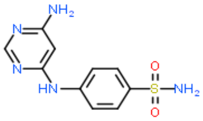 | 0 | 2984 | U55 | 0.0649 | 29 | 2 | -2.2212 | -3.7976 | -19.36 | 0 | 18.697 | 18.73 | -77.49036 | 1.33614 | 1 |
| 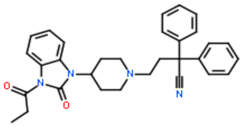 | 0 | 1262 | 1459 | 0.0715 | 69 | 9 | -2.6814 | -7.1563 | -30.17 | 4.9811 | 21.244 | 26.516 | -116.7839 | 2.26867 | 1 |

| | | | | | | | | | | | | | | | |
|---|---|---|---|---|---|---|---|---|---|---|---|---|---|---|---|
| 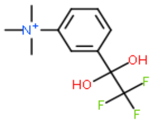 | 0 | 3033 | C11H15F3NO2 | 0.0756 | 32 | 4 | -2.9561 | -4.6492 | -13.61 | 0 | 15.511 | 15.532 | -102.3225 | 1.3055 | 1 |
| 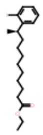 | 0 | 1057 | 1187 | 0.0797 | 51 | 11 | -1.955 | -6.6244 | -23.29 | 4.055 | 15.269 | 18.587 | -110.0695 | 1.98819 | 1 |
| 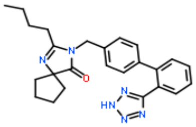 | 0 | 904 | 1029 | 0.0806 | 60 | 5 | -4.4513 | -7.0904 | -30.71 | 5.8264 | 23.522 | 34.335 | -158.7444 | 2.128 | 1 |
| 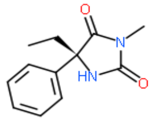 | 0 | 419 | 532 | 0.0862 | 30 | 2 | -2.0268 | -4.0395 | -15.26 | 1.7086 | 13.17 | 16.781 | -78.40858 | 1.26831 | 1 |
| 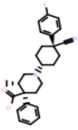 | 0 | 978 | C26H29FN2O2 | 0.107 | 59 | 4 | -9.9263 | -6.8788 | -14.08 | 9.2807 | 20.534 | 31.117 | -133.7568 | 1.6495 | 1 |
| 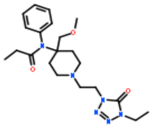 | 0 | 683 | C21H32N6O3 | 0.1088 | 62 | 10 | -4.5202 | -7.8318 | -24.1 | 6.0034 | 21.807 | 22.63 | -140.1438 | 1.99602 | 1 |
| 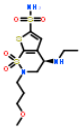 | 0 | 1064 | 1194 | 0.1166 | 44 | 8 | -3.8435 | -5.0121 | -23.81 | 6.3359 | 16.539 | 24.244 | -81.29285 | 1.42806 | 1 |
| 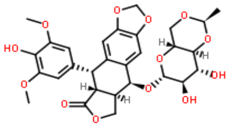 | 0 | 656 | 773 | 0.1362 | 74 | 5 | -6.5013 | -8.2255 | -33.99 | 10.12 | 29.809 | 37.363 | -193.7891 | 2.17013 | 1 |
| 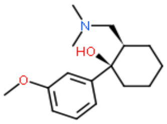 | 0 | 88 | 193 | 0.1431 | 44 | 4 | -2.8028 | -5.701 | -18.48 | 2.6232 | 15.18 | 21.475 | -110.5006 | 1.38762 | 1 |
| 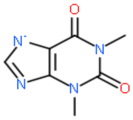 | 0 | 1153 | m | 0.1445 | 20 | 0 | -4.5762 | -2.3673 | -12.02 | 0.2473 | 13.773 | 22.5 | -54.59412 | 1.1064 | 1 |
| 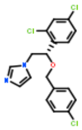 | 0 | 998 | 1127 | 0.1499 | 39 | 6 | -1.9399 | -7.0209 | -29.2 | 4.4787 | 16.972 | 30.217 | -110.4138 | 1.65708 | 1 |

| | | | | | | | | | | | | | | | |
|---|---|---|---|---|---|---|---|---|---|---|---|---|---|---|---|
| 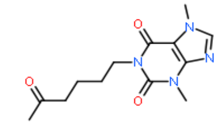 | 0 | 687 | 806 | 0.1562 | 38 | 5 | -2.6186 | -5.3976 | -22.72 | 3.2669 | 16.932 | 23.426 | -84.4976 | 1.35752 | 1 |
| 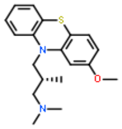 | 0 | 1212 | 1403 | 0.1942 | 47 | 4 | -1.4469 | -6.1361 | -20.7 | 5.9703 | 13.481 | 19.048 | -75.93265 | 1.17144 | 1 |
| 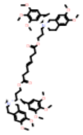 | 0 | 1095 | 1226 | 0.2337 | 154 | 18 | -5.0568 | -11.969 | -58.09 | 31.035 | 34.198 | 36.459 | -212.4822 | 3.7562 | 1 |
| 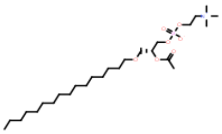 | 0 | 2005 | C26H54NO7P | 0.274 | 89 | 25 | -9.935 | -9.3538 | -34.51 | 12.946 | 28.983 | 32.85 | -145.706 | 2.78277 | 1 |
| 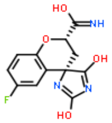 | 0 | 1782 | C12H10FN3O4 | 0.2768 | 30 | 4 | -3.6702 | -3.1202 | -16.61 | 0.9163 | 20.643 | 14.894 | -97.92481 | 1.24231 | 1 |
| 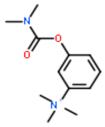 | 0 | 1210 | 1400 | 0.3039 | 35 | 2 | -1.3176 | -5.2697 | -17.32 | 1.5939 | 11.942 | 20.271 | -88.02295 | 1.20112 | 1 |
| 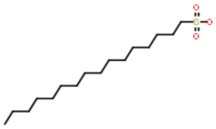 | 0 | 2483 | C16H34O3S | 0.3275 | 53 | 15 | -3.1906 | -7.3579 | -20.37 | 1.5871 | 19.42 | 13.912 | -115.8194 | 2.18988 | 1 |
| 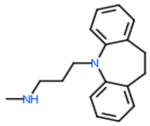 | 0 | 1022 | 1151 | 0.3312 | 42 | 4 | -1.2901 | -5.5897 | -20.23 | 4.2371 | 11.872 | 20.792 | -71.45093 | 1.02543 | 1 |
| 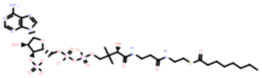 | 0 | 2610 | C29H50N7O17P3S | 0.3411 | 103 | 27 | -19.324 | -7.2549 | -46 | 11.111 | 47.537 | 62.979 | -197.2394 | 3.27349 | 1 |
| 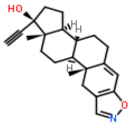 | 0 | 1214 | 1406 | 0.3438 | 52 | 1 | -2.1093 | -5.3168 | -15.44 | 3.3011 | 15.502 | 15.871 | -126.1716 | 1.45006 | 1 |
| 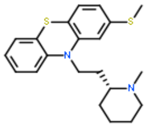 | 0 | 565 | 679 | 0.3517 | 51 | 3 | 0 | -6.799 | -28.63 | 5.1335 | 14.961 | 26.421 | -121.4678 | 1.56134 | 1 |

| | | | | | | | | | | | | | | | |
|---|---|---|---|---|---|---|---|---|---|---|---|---|---|---|---|
| 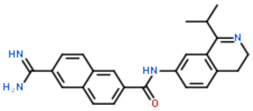 | 0 | 2206 | 155 | 0.3786 | 53 | 1 | -4.4975 | -5.314 | -22.96 | 5.0333 | 19.221 | 30.673 | -75.74877 | 1.50126 | 1 |
| 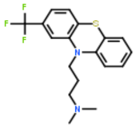 | 0 | 397 | 508 | 0.381 | 43 | 5 | -0.3802 | -6.3008 | -21 | 1.5729 | 9.919 | 23.007 | -77.37324 | 1.13352 | 1 |
| 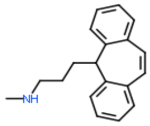 | 0 | 237 | 344 | 0.3986 | 41 | 4 | -2.0937 | -5.6555 | -22.74 | 7.203 | 12.987 | 23.883 | -71.19398 | 1.07026 | 1 |
| 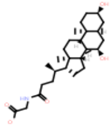 | 0 | 1880 | C26H43NO5 | 0.4013 | 74 | 8 | -9.9689 | -6.5409 | -15.77 | 6.9043 | 23.738 | 28.829 | -156.1778 | 1.7041 | 1 |
| 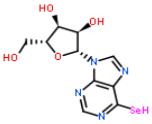 | 0 | 2464 | C10H12N4O4Se | 0.416 | 31 | 6 | -5.817 | -3.6508 | -23.41 | 3.5926 | 24.06 | 26.047 | -78.27778 | 1.36638 | 1 |
| 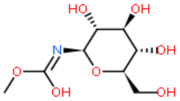 | 0 | 3305 | C8H15NO7 | 0.4236 | 31 | 7 | -4.8374 | -3.27 | -11.1 | 1.1185 | 13.994 | 16.035 | -92.46295 | 1.2987 | 1 |
| 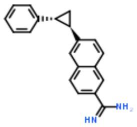 | 0 | 3140 | 745 | 0.4339 | 40 | 2 | -3.2455 | -5.6539 | -21.04 | 3.2108 | 17.258 | 26.365 | -128.048 | 1.58607 | 1 |
| 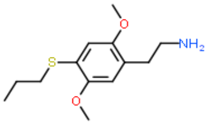 | 0 | 1261 | C13H21NO2S | 0.4473 | 38 | 5 | -5.3097 | -5.0715 | -16.61 | 7.3205 | 18.168 | 19.503 | -72.4874 | 1.55614 | 1 |
| 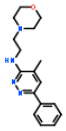 | 0 | 686 | 805 | 0.452 | 44 | 4 | -1.9619 | -6.8323 | -18.13 | 3.4604 | 16.953 | 16.57 | -93.51124 | 1.59158 | 1 |
| 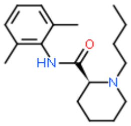 | 0 | 878 | 1002 | 0.4546 | 49 | 4 | -0.9923 | -6.68 | -22.64 | 2.2499 | 14.685 | 23.201 | -117.5238 | 1.60871 | 1 |
| 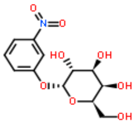 | 0 | 1961 | C12H15NO8 | 0.4748 | 36 | 7 | -5.1026 | -2.715 | -17.23 | 3.5105 | 17.668 | 19.509 | -59.79752 | 1.11898 | 1 |

| | | | | | | | | | | | | | | | |
|---|---|---|---|---|---|---|---|---|---|---|---|---|---|---|---|
| 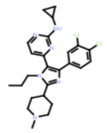 | 0 | 2772 | C25H30Cl2N6 | 0.491 | 63 | 4 | 0 | -8.2932 | -24.12 | 3.1274 | 14.63 | 22.601 | -112.9584 | 1.71038 | 1 |
| 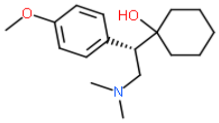 | 0 | 180 | 285 | 0.497 | 47 | 5 | -1.6289 | -5.9293 | -21.12 | 1.4091 | 16.176 | 20.811 | -110.307 | 1.42138 | 1 |
| 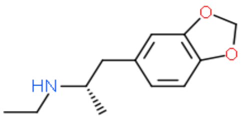 | 0 | 1362 | 1566 | 0.5018 | 32 | 4 | -1.3545 | -4.9498 | -18.75 | 3.3998 | 13.304 | 17.544 | -84.3849 | 1.19313 | 1 |
| 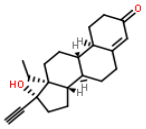 | 0 | 260 | 367 | 0.5025 | 51 | 2 | -1.3276 | -5.6219 | -16.8 | 1.233 | 14.816 | 17.239 | -146.2204 | 1.89001 | 1 |
| 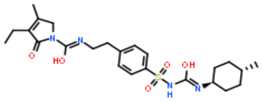 | 0 | 117 | C24H34N4O5S | 0.516 | 68 | 11 | -4.3798 | -6.6951 | -28.07 | 5.7711 | 22.389 | 26.209 | -153.0747 | 2.26999 | 1 |
| 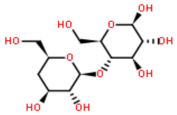 | 0 | 1823 | C12H22O10 | 0.5769 | 44 | 11 | -7.4293 | -3.4697 | -18.13 | 4.111 | 25.697 | 17.402 | -124.5432 | 1.67207 | 1 |
| 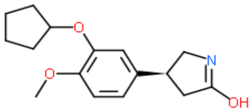 | 0 | 1718 | C16H21NO3 | 0.5817 | 41 | 3 | -2.364 | -5.5121 | -22.01 | 3.1383 | 15.844 | 25.301 | -144.1602 | 1.61527 | 1 |
| 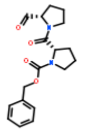 | 0 | 3194 | ZPR | 0.5857 | 46 | 6 | -3.6169 | -5.3524 | -22.59 | 7.4394 | 16.433 | 23.517 | -132.7711 | 1.70217 | 1 |
| 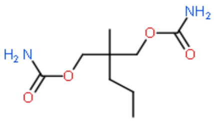 | 0 | 264 | 371 | 0.6032 | 33 | 6 | -3.4785 | -2.5922 | -16.06 | 3.7056 | 16.812 | 13.29 | -54.91607 | 0.93996 | 1 |
| 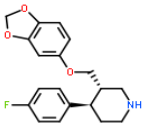 | 0 | 599 | 715 | 0.6063 | 44 | 3 | -2.2014 | -6.1678 | -27.21 | 3.7133 | 19.756 | 28.286 | -114.5742 | 1.644 | 1 |
| 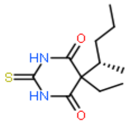 | 0 | 486 | 599 | 0.6199 | 34 | 4 | -2.8783 | -3.3755 | -13.73 | 3.1613 | 13.471 | 14.39 | -93.24423 | 1.1745 | 1 |

| | | | | | | | | | | | | | | | |
|---|---|---|---|---|---|---|---|---|---|---|---|---|---|---|---|
| 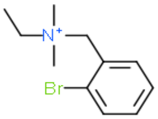 | 0 | 1028 | 1158 | 0.6214 | 30 | 3 | 0 | -4.5548 | -15.7 | 1.0392 | 7.2914 | 17.398 | -49.17372 | 0.94241 | 1 |
| 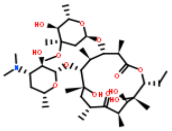 | 0 | 94 | 12560 | 0.6235 | 118 | 12 | -4.026 | -8.1468 | -24.84 | 8.6972 | 24.143 | 16.036 | -150.469 | 2.60066 | 1 |
| 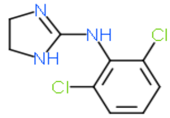 | 0 | 462 | 575 | 0.6244 | 23 | 1 | -1.4376 | -4.5832 | -16.99 | 0.3831 | 13.148 | 20.646 | -67.76917 | 1.19452 | 1 |
| 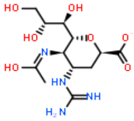 | 0 | 3088 | C12H22N4O7 | 0.6297 | 44 | 11 | -4.6784 | -3.1534 | -17.34 | 0 | 22.977 | 12.251 | -123.0788 | 1.57262 | 1 |
| 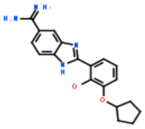 | 0 | 2256 | 655 | 0.6386 | 45 | 1 | -3.3118 | -5.6269 | -22.18 | 0.0106 | 15.785 | 34.126 | -103.1624 | 1.55855 | 1 |
| 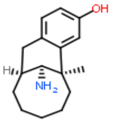 | 0 | 1079 | C16H23NO | 0.6418 | 41 | 1 | -4.4791 | -3.8533 | -17.69 | 9.8469 | 16.755 | 19.95 | -92.1853 | 1.00762 | 1 |
| 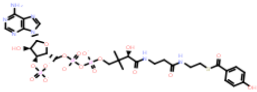 | 0 | 1444 | C28H40N7O18P3S | 0.65 | 93 | 21 | -18.326 | -6.1758 | -45.63 | 16.656 | 40.496 | 66.394 | -192.3079 | 2.68612 | 1 |
| 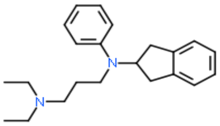 | 0 | 1236 | 1429 | 0.6917 | 54 | 8 | 0 | -7.0659 | -26.3 | 5.3141 | 12.241 | 22.061 | -116.6692 | 1.60288 | 1 |
| 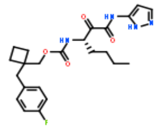 | 0 | 1633 | FSP | 0.7006 | 61 | 11 | -3.887 | -4.4081 | -25 | 10.142 | 18.183 | 18.433 | -45.97548 | 1.55822 | 1 |
| 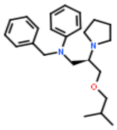 | 0 | 1113 | 1244 | 0.7133 | 61 | 10 | 0 | -8.1623 | -29.38 | 7.2444 | 14.003 | 22.291 | -119.2585 | 1.78556 | 1 |
| 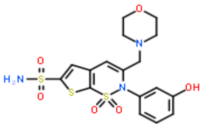 | 0 | 2942 | INQ | 0.7145 | 48 | 5 | -1.6228 | -5.2603 | -26.74 | 4.946 | 19.256 | 22.232 | -116.0625 | 1.98174 | 1 |

| | | | | | | | | | | | | | | | |
|---|---|---|---|---|---|---|---|---|---|---|---|---|---|---|---|
| 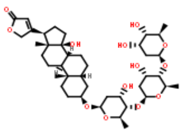 | 0 | 1207 | C41H64O13 | 0.7331 | 118 | 12 | -5.9043 | -9.9473 | -30.14 | 11.26 | 27.614 | 26.984 | -221.6717 | 3.02092 | 1 |
| 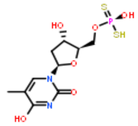 | 0 | 2676 | C10H15N2O6PS2 | 0.7367 | 36 | 8 | -5.9468 | -3.0632 | -23.23 | 3.9533 | 26.021 | 21.789 | -127.0957 | 1.69561 | 1 |
| 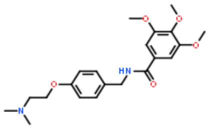 | 0 | 549 | 662 | 0.7418 | 56 | 5 | -3.035 | -8.396 | -29.83 | 9.7701 | 21.123 | 29.046 | -139.2696 | 1.95729 | 1 |
| 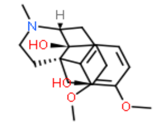 | 0 | 1343 | 1547 | 0.7492 | 51 | 2 | -1.7567 | -5.4893 | -15.92 | 3.8974 | 16.123 | 13.873 | -90.57732 | 1.37103 | 1 |
| 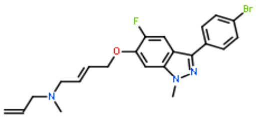 | 0 | 1896 | R04 | 0.7494 | 51 | 7 | 0 | -8.1956 | -29.43 | 2.5666 | 17.495 | 25.469 | -135.3425 | 2.07665 | 1 |
| 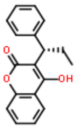 | 0 | 824 | C18H16O3 | 0.7503 | 37 | 3 | -2.6558 | -5.0927 | -19.59 | 3.7297 | 16.432 | 21.382 | -101.4151 | 1.39894 | 1 |
| 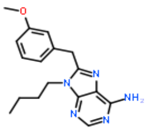 | 0 | 3444 | PU5 | 0.7549 | 44 | 5 | -0.62 | -5.75 | -23.98 | 3.3903 | 15.671 | 20.255 | -84.84335 | 1.14727 | 1 |
| 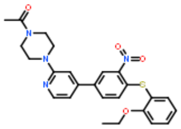 | 0 | 1930 | C25H26N4O4S | 0.7664 | 60 | 3 | -2.6029 | -7.51 | -29.66 | 14.746 | 19.358 | 25.571 | -130.7506 | 2.14044 | 1 |
| 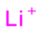 | 0 | 1184 | 1356 | 0.7693 | 1 | 0 | -4.4601 | 0.12086 | -0.833 | 0 | 1.9334 | 17.205 | 21.55538 | -0.1226 | 1 |
| 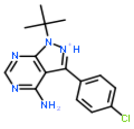 | 0 | 2717 | PP2 | 0.7984 | 38 | 2 | -1.5595 | -5.9039 | -21.47 | 1.3195 | 16.308 | 24.16 | -102.5739 | 1.25148 | 1 |
| 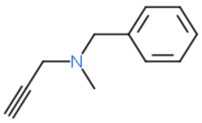 | 0 | 1418 | 1626 | 0.8279 | 25 | 3 | 0 | -4.7852 | -16.85 | 1.12 | 8.6758 | 18.036 | -65.89548 | 0.99728 | 1 |

| | | | | | | | | | | | | | | | |
|---|---|---|---|---|---|---|---|---|---|---|---|---|---|---|---|
| 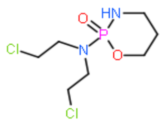 | 0 | 418 | 531 | 0.8286 | 29 | 5 | -0.6813 | -4.8033 | -12.13 | 1.6933 | 11.565 | 7.8956 | -48.09273 | 1.04308 | 1 |
| 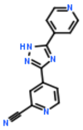 | 0 | 1473 | FYX | 0.8347 | 27 | 0 | -2.8269 | -4.8704 | -20.1 | 1.683 | 18.166 | 25.334 | -82.26131 | 1.40834 | 1 |
| 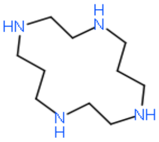 | 0 | 2870 | C10H24CuN4 | 0.8429 | 38 | 0 | -1.4021 | -4.6611 | -12.5 | 0.3663 | 15.33 | 12.575 | -67.0953 | 1.26325 | 1 |
| 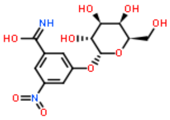 | 0 | 2507 | C13H16N2O9 | 0.8521 | 40 | 8 | -7.2037 | -3.157 | -18.43 | 4.5048 | 26.139 | 18.969 | -113.0179 | 1.52725 | 1 |
| 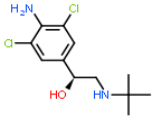 | 0 | 1215 | 1407 | 0.8659 | 35 | 5 | -3.5752 | -5.2994 | -15.57 | 3.5935 | 15.86 | 18.146 | -70.83323 | 1.05651 | 1 |
| 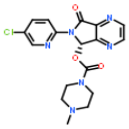 | 0 | 293 | 402 | 0.8885 | 44 | 2 | -1.092 | -6.754 | -29.41 | 2.1987 | 20.724 | 29.58 | -120.0498 | 1.63582 | 1 |
| 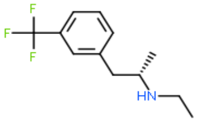 | 0 | 461 | 574 | 0.9181 | 32 | 5 | -0.9571 | -5.4573 | -18.24 | 2.0254 | 11.208 | 19.114 | -76.69022 | 1.20087 | 1 |
| 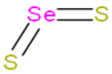 | 0 | 848 | S2Se | 0.9232 | 3 | 0 | 0 | -0.4156 | -7.576 | 0 | 5.0164 | 7.599 | -9.066186 | 0.274 | 1 |
| 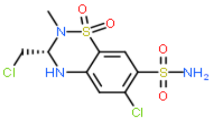 | 0 | 127 | 232 | 0.926 | 31 | 3 | 0 | -3.9535 | -20.14 | 0.5299 | 12.742 | 18.25 | -98.99952 | 1.65103 | 1 |
| 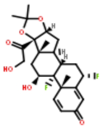 | 0 | 478 | C24H30F2O6 | 0.9784 | 62 | 4 | -5.4624 | -4.9801 | -15.24 | 2.1895 | 21.302 | 20.74 | -98.8637 | 1.57853 | 1 |
| 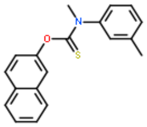 | 0 | 412 | 525 | 0.987 | 39 | 1 | -0.2508 | -5.6519 | -26.53 | 9.5628 | 13.674 | 23.243 | -101.3679 | 1.37072 | 1 |

| | | | | | | | | | | | | | | | |
|---|---|---|---|---|---|---|---|---|---|---|---|---|---|---|---|
| 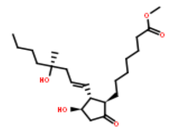 | 0 | 808 | 929 | 1.0026 | 65 | 15 | -6.1135 | -6.4185 | -24.96 | 4.3324 | 26.104 | 22.043 | -126.3634 | 2.28912 | 1 |
| 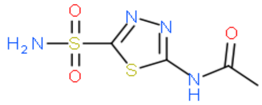 | 0 | 700 | 819 | 1.0028 | 19 | 2 | -2.2187 | -2.6274 | -14.94 | 1.7953 | 16.038 | 13.648 | -66.07217 | 1.03295 | 1 |
| 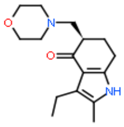 | 0 | 1410 | 1618 | 1.0068 | 44 | 3 | -2.6098 | -5.3638 | -14.2 | 1.6939 | 13.121 | 19.314 | -106.5404 | 1.47474 | 1 |
| 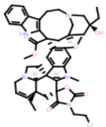 | 0 | 2570 | C46H56ClN5O7 | 1.01 | 115 | 7 | -1.919 | -10.45 | -29.44 | 6.4129 | 22.45 | 26.391 | -167.081 | 2.5113 | 1 |
| 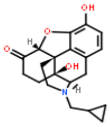 | 0 | 589 | 704 | 1.0138 | 48 | 3 | -2.0635 | -5.426 | -19.41 | 0 | 19.538 | 19.435 | -119.4489 | 1.55331 | 1 |
| 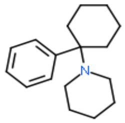 | 0 | 3229 | C17H25N | 1.0327 | 43 | 2 | 0 | -5.619 | -19.22 | 1.3401 | 10.743 | 21.154 | -108.645 | 1.41208 | 1 |
| 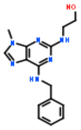 | 0 | 1874 | OLO | 1.0334 | 40 | 5 | -2.5092 | -4.8489 | -26.89 | 1.9524 | 18.005 | 30.066 | -69.80829 | 1.3354 | 1 |
| 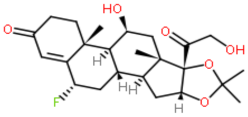 | 0 | 726 | C24H33FO6 | 1.0475 | 64 | 4 | -5.429 | -4.968 | -15.34 | 1.9388 | 21.37 | 21.003 | -101.0746 | 1.57443 | 1 |
| 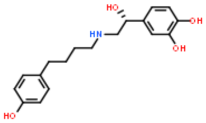 | 0 | 974 | 1102 | 1.0698 | 46 | 9 | -6.3266 | -5.2318 | -23.27 | 7.332 | 22.014 | 26.579 | -116.2455 | 1.6896 | 1 |
| 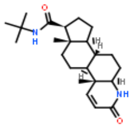 | 0 | 1086 | 1216 | 1.0712 | 63 | 2 | -1.6619 | -6.885 | -19.76 | 4.8761 | 16.673 | 19.733 | -175.972 | 1.94723 | 1 |
| 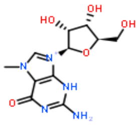 | 0 | 3157 | C11H16N5O5 | 1.0732 | 37 | 5 | -6.9665 | -2.6233 | -20.17 | 3.0692 | 25.506 | 25.234 | -45.43218 | 1.01907 | 1 |

| | | | | | | | | | | | | | | | |
|---|---|---|---|---|---|---|---|---|---|---|---|---|---|---|---|
| 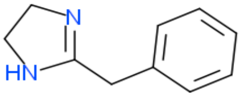 | 0 | 678 | 797 | 1.094 | 24 | 2 | -1.2189 | -4.36 | -17.28 | 0.6648 | 11.635 | 21.29 | -73.28278 | 1.09686 | 1 |
| 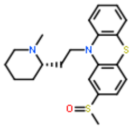 | 0 | 811 | C21H26N2OS2 | 1.0974 | 52 | 4 | -3.3198 | -6.5074 | -20.97 | 9.3662 | 15.337 | 23.195 | -106.4351 | 1.43158 | 1 |
| 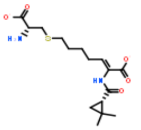 | 0 | 1390 | C16H26N2O5S | 1.0977 | 48 | 11 | -10.587 | -4.3019 | -22.83 | 9.6678 | 23.785 | 35.385 | -138.9653 | 1.9512 | 1 |
| 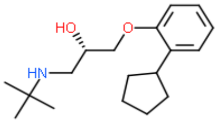 | 0 | 1186 | 1359 | 1.1376 | 50 | 7 | -1.3426 | -5.4071 | -20.7 | 8.8867 | 9.4722 | 18.514 | -67.14463 | 1.32674 | 1 |
| 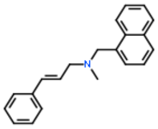 | 0 | 618 | 735 | 1.16 | 43 | 5 | 0 | -6.2282 | -25.38 | 3.9534 | 13.133 | 23.471 | -108.0246 | 1.36609 | 1 |
| 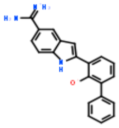 | 0 | 2841 | 696 | 1.1604 | 42 | 1 | -3.3 | -5.7042 | -24.63 | 5.3873 | 18.735 | 30.419 | -111.5551 | 1.73222 | 1 |
| 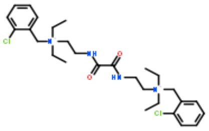 | 0 | 993 | 1122 | 1.1822 | 78 | 15 | -1.2593 | -7.6715 | -34.06 | 3.4353 | 19.233 | 27.397 | -78.87513 | 1.95766 | 1 |
| 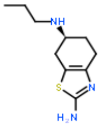 | 0 | 303 | C10H17N3S | 1.1937 | 31 | 3 | -2.6917 | -4.8668 | -14.89 | 1.2989 | 16.92 | 16.496 | -83.64761 | 1.32486 | 1 |
| 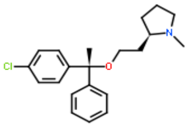 | 0 | 178 | 26987 | 1.2024 | 50 | 6 | -0.4617 | -7.0235 | -21.38 | 3.2591 | 13.048 | 19.876 | -128.0537 | 1.60762 | 1 |
| 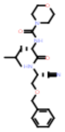 | 0 | 3406 | BLN | 1.2312 | 59 | 11 | -4.2919 | -6.519 | -29.3 | 8.8743 | 18.466 | 30.283 | -155.5721 | 1.81494 | 1 |
| 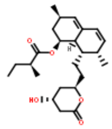 | 0 | 122 | 227 | 1.2344 | 65 | 7 | -2.2115 | -6.4301 | -24.03 | 3.4974 | 19.546 | 21.529 | -133.9571 | 1.67737 | 1 |

| | | | | | | | | | | | | | | | |
|---|---|---|---|---|---|---|---|---|---|---|---|---|---|---|---|
| 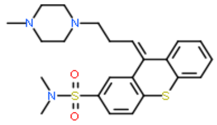 | 0 | 1415 | 1623 | 1.2401 | 59 | 5 | 0 | -7.2462 | -30.29 | 4.8884 | 17.635 | 26.097 | -121.7803 | 1.71878 | 1 |
| 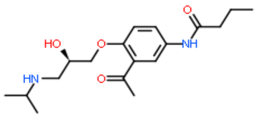 | 0 | 1063 | 1193 | 1.2421 | 52 | 8 | -2.6895 | -5.6866 | -26.75 | 5.2805 | 17.723 | 26.281 | -54.45837 | 1.58424 | 1 |
| 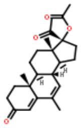 | 0 | 244 | C24H32O4 | 1.2568 | 60 | 2 | -1.1431 | -6.0474 | -15.62 | 2.3104 | 15.323 | 14.756 | -126.9865 | 1.77763 | 1 |
| 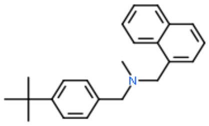 | 0 | 963 | 1091 | 1.2611 | 51 | 5 | 0 | -6.4251 | -26.45 | 6.335 | 13.025 | 23.401 | -131.4647 | 1.65752 | 1 |
| 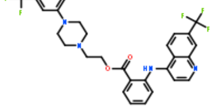 | 0 | 1226 | 1419 | 1.2796 | 68 | 4 | -0.1156 | -8.4249 | -31.73 | 4.9449 | 20.937 | 27.086 | -142.9368 | 2.49209 | 1 |
| 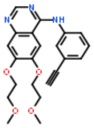 | 0 | 417 | C22H23N3O4 | 1.2869 | 52 | 6 | -3.6048 | -6.8629 | -22.38 | 8.7615 | 20.2 | 20.271 | -44.81685 | 1.78415 | 1 |
| 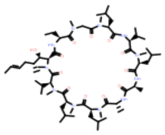 | 0 | 7 | C62H111N11O12 | 1.2892 | 196 | 16 | -3.5673 | -12.688 | -44.59 | 19.072 | 31.197 | 28.734 | -220.2275 | 3.83251 | 1 |
| 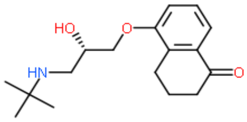 | 0 | 1080 | 1210 | 1.3073 | 46 | 6 | -2.1133 | -5.403 | -20.82 | 3.1548 | 17.617 | 18.765 | -101.2504 | 1.62921 | 1 |
| 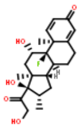 | 0 | 333 | C22H29FO5 | 1.3524 | 57 | 5 | -3.7715 | -5.0221 | -18.84 | 0.5212 | 19.737 | 22.705 | -134.1108 | 1.41452 | 1 |
| 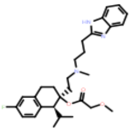 | 0 | 1201 | 1388 | 1.3593 | 74 | 11 | -0.7671 | -8.896 | -32 | 7.5414 | 17.077 | 26.254 | -145.3335 | 1.92632 | 1 |
| 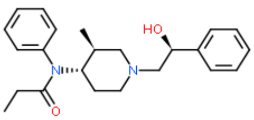 | 0 | 1366 | 1570 | 1.3659 | 57 | 8 | -3.2898 | -6.5676 | -22.21 | 2.0517 | 17.642 | 25.819 | -110.484 | 2.09761 | 1 |

| | | | | | | | | | | | | | | | |
|---|---|---|---|---|---|---|---|---|---|---|---|---|---|---|---|
| 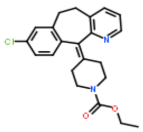 | 0 | 345 | 455 | 1.367 | 50 | 2 | 0 | -7.0742 | -21.84 | 1.6506 | 14.233 | 22.855 | -105.5734 | 1.53502 | 1 |
| 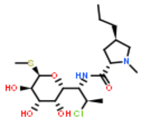 | 0 | 1060 | C18H33ClN2O5S | 1.384 | 60 | 10 | -4.2365 | -6.5895 | -22.05 | 5.9916 | 20.104 | 21.058 | -142.2586 | 1.80004 | 1 |
| 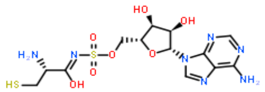 | 0 | 2402 | C13H19N7O7S2 | 1.3943 | 48 | 12 | -10.494 | -3.9205 | -26.56 | 7.3491 | 33.481 | 30.931 | -100.0705 | 1.49666 | 1 |
| 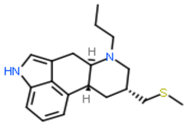 | 0 | 1056 | 1186 | 1.4141 | 48 | 4 | 0 | -6.1604 | -22.14 | 2.7503 | 11.562 | 22.664 | -92.68638 | 1.07061 | 1 |
| 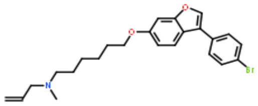 | 0 | 2080 | C24H28BrNO2 | 1.4173 | 56 | 10 | -0.833 | -7.9506 | -31.7 | 7.7058 | 17.608 | 25.342 | -141.8148 | 1.98809 | 1 |
| 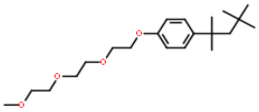 | 0 | 1838 | TRT | 1.4287 | 61 | 12 | -4.2635 | -7.1479 | -22.23 | 8.8394 | 16.724 | 21.492 | -69.63843 | 1.66435 | 1 |
| 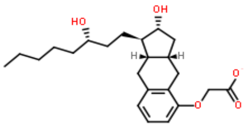 | 0 | 267 | C23H34O5 | 1.4308 | 61 | 11 | -5.7193 | -7.3014 | -23.95 | 6.9676 | 23.471 | 24.793 | -139.4469 | 1.90601 | 1 |
| 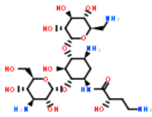 | 0 | 369 | C22H43N5O13 | 1.4316 | 83 | 22 | -11.527 | -5.2774 | -29.72 | 14.363 | 33.877 | 25.19 | -120.6569 | 1.93028 | 1 |
| 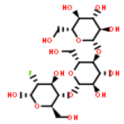 | 0 | 1425 | C18H31FO15 | 1.4371 | 65 | 17 | -7.2304 | -4.7713 | -20.4 | 0 | 29.736 | 16.404 | -139.8228 | 2.23705 | 1 |
| 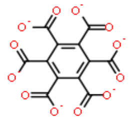 | 0 | 1469 | C12H6O12 | 1.4409 | 24 | 6 | -11.112 | -1.6901 | -17.02 | 5.1088 | 24.05 | 34.848 | -118.5026 | 1.25803 | 1 |
| 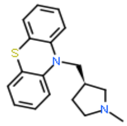 | 0 | 781 | 902 | 1.4437 | 41 | 2 | -1.2526 | -5.9632 | -21.58 | 2.2197 | 13.906 | 26.104 | -121.6615 | 1.37791 | 1 |

| | | | | | | | | | | | | | | | |
|---|---|---|---|---|---|---|---|---|---|---|---|---|---|---|---|
| 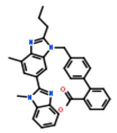 | 0 | 843 | 966 | 1.4453 | 68 | 5 | -3.5827 | -8.4746 | -31.81 | 11.281 | 22.572 | 32.266 | -181.4791 | 2.14578 | 1 |
| 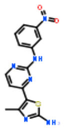 | 0 | 2536 | CK7 | 1.4615 | 35 | 1 | -1.2376 | -5.1641 | -23.35 | 4.5111 | 17.282 | 22.934 | -85.35534 | 1.48431 | 1 |
| 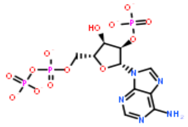 | 0 | 2102 | C10H16N5O13P3 | 1.4853 | 42 | 9 | -14.368 | -3.5948 | -26.25 | 0 | 33.616 | 54.372 | -113.751 | 1.62359 | 1 |
| 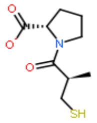 | 0 | 1793 | MCO | 1.517 | 28 | 5 | -4.3452 | -3.1722 | -14.32 | 2.2336 | 14.005 | 21.385 | -89.61314 | 1.02109 | 1 |
| 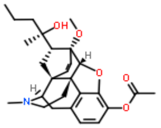 | 0 | 1272 | C27H35NO5 | 1.5201 | 68 | 5 | -1.1054 | -7.6575 | -23.15 | 3.8686 | 16.629 | 22.436 | -131.5385 | 1.65152 | 1 |
| 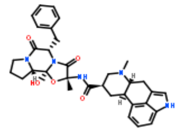 | 0 | 214 | 320 | 1.5244 | 80 | 5 | -1.4516 | -8.2603 | -32.85 | 6.2187 | 25.716 | 26.609 | -210.5567 | 2.47021 | 1 |
| 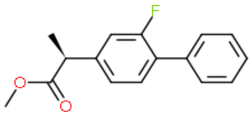 | 0 | 3393 | C16H15FO2 | 1.5471 | 34 | 3 | -0.8405 | -5.7799 | -19.94 | 2.5398 | 14.615 | 20.001 | -109.5626 | 1.56139 | 1 |
| 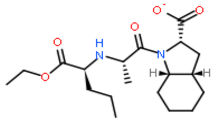 | 0 | 671 | C19H32N2O5 | 1.5767 | 57 | 9 | -6.8598 | -6.0836 | -19.02 | 7.947 | 20.423 | 25.061 | -133.7155 | 1.51308 | 1 |
| 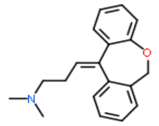 | 0 | 1013 | C19H21NO | 1.5863 | 42 | 3 | -0.9276 | -5.7437 | -19.65 | 2.4555 | 12.601 | 22.29 | -74.12027 | 1.20039 | 1 |
| 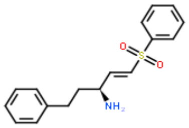 | 0 | 2571 | C17H19NO2S | 1.5915 | 40 | 7 | -0.4608 | -4.9833 | -24.37 | 4.9391 | 14.47 | 18.871 | -97.3818 | 1.35958 | 1 |
| 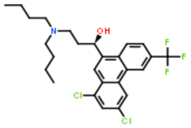 | 0 | 1088 | 1218 | 1.5933 | 63 | 11 | -0.8624 | -7.9989 | -27.05 | 4.9826 | 17.154 | 20.796 | -140.3631 | 1.77564 | 1 |

| | | | | | | | | | | | | | | | |
|---|---|---|---|---|---|---|---|---|---|---|---|---|---|---|---|
| 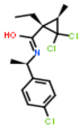 | 0 | 2642 | C15H18Cl3NO | 1.5975 | 38 | 5 | 0 | -5.931 | -20.17 | 1.1676 | 15.127 | 16.204 | -116.256 | 1.70735 | 1 |
| 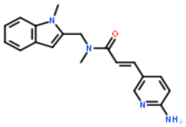 | 0 | 1640 | AYM | 1.5979 | 44 | 3 | -2.1969 | -5.9055 | -24.09 | 3.808 | 19.818 | 24.629 | -111.5616 | 1.55372 | 1 |
| 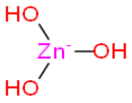 | 0 | 1919 | H3O3Zn | 1.6079 | 7 | 3 | -6.3453 | -0.1704 | -5.27 | 0.5557 | 12.107 | 17.927 | -7.908581 | 0.26001 | 1 |
| 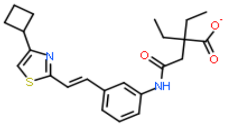 | 0 | 474 | C23H28N2O3S | 1.6118 | 56 | 6 | -5.591 | -8.0723 | -26.36 | 14.279 | 19.811 | 31.061 | -163.0623 | 1.83342 | 1 |
| 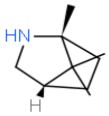 | 0 | 2145 | C9H17N | 1.6146 | 27 | 0 | -2.441 | -3.9194 | -3.8 | 0.514 | 9.0457 | 10.794 | -59.58586 | 0.6821 | 1 |
| 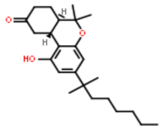 | 0 | 376 | 486 | 1.617 | 63 | 6 | -1.9 | -6.5868 | -20.42 | 3.2396 | 16.749 | 19.931 | -80.00291 | 1.67387 | 1 |
| 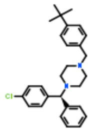 | 0 | 247 | 354 | 1.6173 | 64 | 6 | 0 | -9.0602 | -28.57 | 2.7737 | 17.342 | 27.247 | -172.474 | 2.11915 | 1 |
| 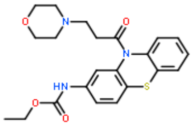 | 0 | 566 | 680 | 1.6197 | 55 | 6 | -1.9849 | -6.4917 | -27.18 | 3.634 | 20.674 | 25.583 | -131.0448 | 1.84391 | 1 |
| 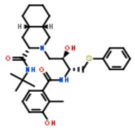 | 0 | 115 | 220 | 1.6379 | 85 | 10 | -3.0103 | -8.8322 | -30.75 | 10.728 | 25.131 | 22.177 | -147.6354 | 2.14114 | 1 |
| 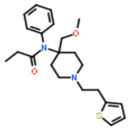 | 0 | 592 | 708 | 1.6512 | 57 | 9 | -1.8482 | -7.9164 | -24.76 | 3.8894 | 17.043 | 24.061 | -130.6493 | 1.93792 | 1 |
| 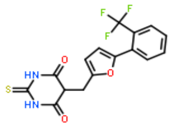 | 0 | 1298 | 1496 | 1.6554 | 36 | 2 | -2.6618 | -4.5071 | -22.32 | 4.2124 | 17.68 | 25.374 | -146.4341 | 1.74951 | 1 |

| | | | | | | | | | | | | | | |
|---|---|---|---|---|---|---|---|---|---|---|---|---|---|---|
| 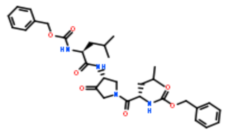 | 0 | 3073 | C32H42N4O7 | 1.6672 | 85 | 16 | -3.3129 | -8.5984 | -40.59 | 7.8879 | 27.777 | 32.171 | -173.6897 | 2.85299 | 1 |
| 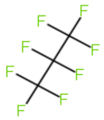 | 0 | 443 | C3F8 | 1.6853 | 11 | 1 | 0 | -3.3511 | -9.636 | 0 | 5.6262 | 13.253 | -60.75742 | 0.43599 | 1 |
| 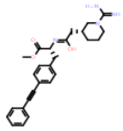 | 0 | 3120 | C26H30N4O3 | 1.6866 | 63 | 9 | -3.6574 | -7.5735 | -32.32 | 8.8309 | 26.32 | 27.111 | -146.5483 | 2.15264 | 1 |
| 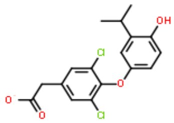 | 0 | 2858 | C17H16Cl2O4 | 1.6916 | 38 | 5 | -4.311 | -5.604 | -17.47 | 4.976 | 19.308 | 20.244 | -113.7782 | 1.86926 | 1 |
| 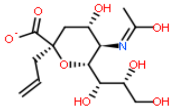 | 0 | 1502 | C14H23NO8 | 1.7082 | 45 | 12 | -3.1465 | -4.0987 | -19.47 | 0 | 18.66 | 16.204 | -87.08008 | 1.5218 | 1 |
| 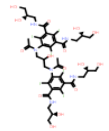 | 0 | 1118 | C35H44I6N6O15 | 1.7142 | 106 | 27 | -12.591 | -7.5013 | -43.59 | 24.971 | 46.161 | 24.828 | -175.4435 | 3.47257 | 1 |
| 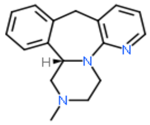 | 0 | 263 | 370 | 1.7229 | 39 | 0 | 0 | -5.3582 | -18.6 | 0.7187 | 10.608 | 23.529 | -91.36516 | 1.0462 | 1 |
| 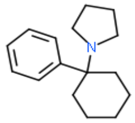 | 0 | 1345 | C16H23N | 1.7482 | 40 | 2 | 0 | -5.4537 | -17.13 | 1.3568 | 10.466 | 19.2 | -103.2149 | 1.39775 | 1 |
| 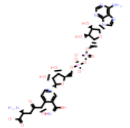 | 0 | 2089 | C26H35N8O18P2 | 1.7642 | 86 | 22 | -12.183 | -4.3382 | -36.97 | 0 | 44.833 | 38.844 | -146.041 | 2.63126 | 1 |
| 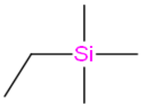 | 0 | 2009 | C5H14Si | 1.7742 | 20 | 1 | 0 | -3.9948 | -10.49 | 0.2567 | 6.9785 | 13.571 | -38.19892 | 0.70027 | 1 |
| 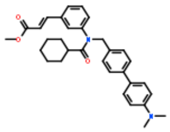 | 0 | 2273 | C32H36N2O3 | 1.7782 | 73 | 4 | -1.3262 | -10.115 | -30.69 | 6.2672 | 22.543 | 29.589 | -193.777 | 2.32179 | 1 |

| | | | | | | | | | | | | | | | |
|---|---|---|---|---|---|---|---|---|---|---|---|---|---|---|---|
| 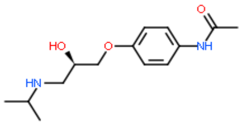 | 0 | 1149 | C14H22N2O3 | 1.7836 | 41 | 6 | -4.4037 | -5.758 | -20.8 | 6.2682 | 20.329 | 22.696 | -98.37028 | 1.29841 | 1 |
| 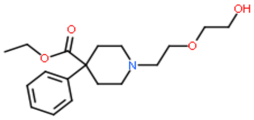 | 0 | 1306 | 1505 | 1.7983 | 50 | 9 | -3.4042 | -6.1393 | -20.4 | 6.1117 | 17.585 | 19.192 | -108.1045 | 1.61396 | 1 |
| 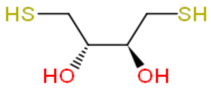 | 0 | 1937 | C4H10O2S2 | 1.8102 | 18 | 7 | -4.7626 | -1.4328 | -9.943 | 5.5897 | 13.83 | 10.359 | -37.27753 | 0.78585 | 1 |
| 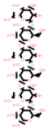 | 0 | 2879 | C36H62O28S3 | 1.8175 | 129 | 36 | -18.5 | -7.6642 | -29.92 | 12.216 | 43.023 | 34.069 | -176.7233 | 2.86127 | 1 |
| 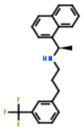 | 0 | 888 | 1012 | 1.8191 | 48 | 7 | -0.7288 | -6.8391 | -25.79 | 8.8917 | 14.325 | 20.844 | -134.3084 | 1.49615 | 1 |
| 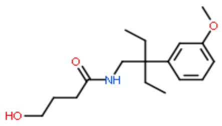 | 0 | 1290 | 1487 | 1.8246 | 48 | 9 | -5.2904 | -5.7913 | -19.86 | 7.9171 | 17.722 | 23.5 | -115.2805 | 1.63888 | 1 |
| 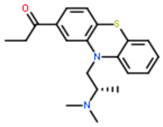 | 0 | 660 | 777 | 1.8271 | 48 | 4 | -0.2616 | -6.6246 | -28.31 | 5.7351 | 16.139 | 26.195 | -120.4869 | 1.55311 | 1 |
| 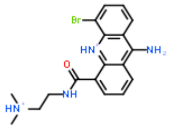 | 0 | 2127 | C18H21BrN4O | 1.8325 | 45 | 3 | -0.7474 | -5.7874 | -24.85 | 0 | 17.131 | 26.976 | -85.12778 | 1.57449 | 1 |
| 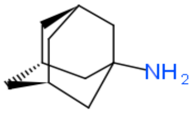 | 0 | 794 | 915 | 1.8358 | 28 | 1 | -3.2214 | -3.5365 | -3.675 | 0.8146 | 10.675 | 10.507 | -66.90909 | 0.61903 | 1 |
| 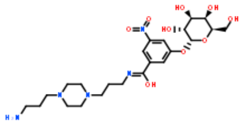 | 0 | 2916 | C23H37N5O9 | 1.8402 | 74 | 16 | -8.6156 | -5.8249 | -28.55 | 15.351 | 31.457 | 20.738 | -98.01295 | 2.40295 | 1 |
| 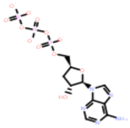 | 0 | 1635 | C10H16N5O12P3 | 1.8486 | 42 | 9 | -16.14 | -3.7845 | -31.25 | 4.7217 | 37.373 | 60.914 | -110.9277 | 1.51825 | 1 |

| | | | | | | | | | | | | | | | |
|---|---|---|---|---|---|---|---|---|---|---|---|---|---|---|---|
| 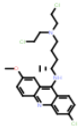 | 0 | 1987 | C23H28Cl3N3O | 1.8494 | 58 | 9 | -1.8307 | -8.1433 | -26.45 | 2.897 | 17.211 | 27.792 | -138.124 | 1.6703 | 1 |
| 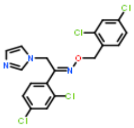 | 0 | 134 | C18H13Cl4N3O | 1.8501 | 39 | 4 | -2.0027 | -6.944 | -27.41 | 5.7977 | 18.075 | 29.61 | -114.7895 | 1.52218 | 1 |
| 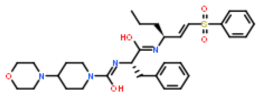 | 0 | 1990 | C31H42N4O5S | 1.8514 | 83 | 14 | -1.6119 | -8.7349 | -33.3 | 9.9228 | 23.711 | 19.617 | -142.7728 | 2.41214 | 1 |
| 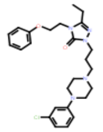 | 0 | 1020 | 1149 | 1.8886 | 65 | 9 | -1.5643 | -7.3826 | -32.2 | 8.1093 | 19.695 | 27.201 | -144.3376 | 2.24559 | 1 |
| 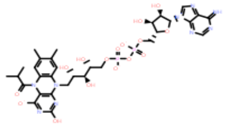 | 0 | 3190 | C31H39N9O16P2 | 1.8901 | 95 | 20 | -14.596 | -5.8845 | -36.58 | 0 | 42.369 | 53.882 | -166.9446 | 2.55622 | 1 |
| 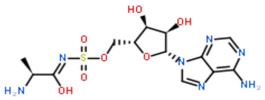 | 0 | 3049 | C13H19N7O7S | 1.9045 | 47 | 10 | -7.4998 | -4.2232 | -28.28 | 6.1987 | 29.153 | 30.913 | -115.7474 | 1.79822 | 1 |
| 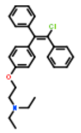 | 0 | 762 | C26H28ClNO | 1.9066 | 57 | 7 | -1.6713 | -8.8065 | -30.76 | 8.992 | 17.223 | 30.726 | -134.8969 | 1.6699 | 1 |
| 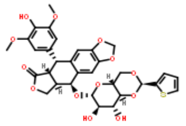 | 0 | 334 | C32H32O13S | 1.9072 | 78 | 6 | -6.9424 | -8.5087 | -30.57 | 15.03 | 30.28 | 30.927 | -213.7316 | 2.26659 | 1 |
| 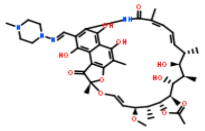 | 0 | 919 | C43H58N4O12 | 1.911 | 117 | 5 | -1.4934 | -7.9616 | -28.58 | 0 | 29.127 | 22.367 | -137.3835 | 2.66828 | 1 |
| 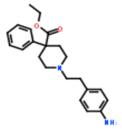 | 0 | 792 | C22H28N2O2 | 1.9388 | 54 | 6 | -3.1592 | -7.5125 | -21.41 | 4.4109 | 19.586 | 23.386 | -132.3519 | 1.87497 | 1 |
| 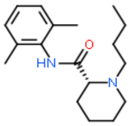 | 0 | 191 | 297 | 1.9439 | 49 | 4 | -0.8858 | -6.5005 | -17.72 | 0.9505 | 13.285 | 20.185 | -100.1561 | 1.56723 | 1 |

| | | | | | | | | | | | | | | |
|---|---|---|---|---|---|---|---|---|---|---|---|---|---|---|
| 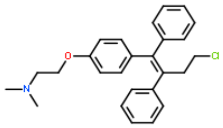 | 0 | 426 | 539 | 1.9457 | 57 | 7 | -2.0746 | -8.4542 | -27.57 | 7.4528 | 18.745 | 26.813 | -121.2895 | 1.74554 | 1 |
| 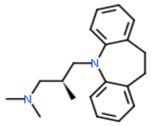 | 0 | 610 | 726 | 1.9469 | 48 | 4 | 0 | -6.3848 | -22.08 | 4.0673 | 11.525 | 22.454 | -83.46857 | 1.20758 | 1 |
| 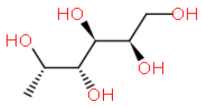 | 0 | 3450 | C6H14O5 | 1.9514 | 25 | 9 | -5.195 | -2.1777 | -12.88 | 5.5371 | 15.09 | 14.236 | -83.16713 | 0.89409 | 1 |
| 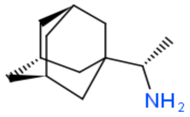 | 0 | 368 | 478 | 1.9579 | 34 | 2 | -2.7295 | -3.9576 | -7.635 | 1.411 | 12.289 | 11.95 | -68.8995 | 0.91982 | 1 |
| 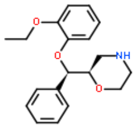 | 0 | 129 | C19H23NO3 | 1.9728 | 46 | 4 | -0.9747 | -6.2904 | -19.68 | 4.2358 | 15.049 | 18.343 | -104.7645 | 1.66039 | 1 |
| 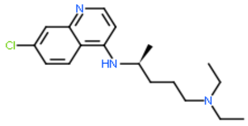 | 0 | 495 | 608 | 2.0025 | 48 | 7 | -0.836 | -6.8281 | -26.77 | 7.469 | 14.781 | 23.783 | -98.90131 | 1.63161 | 1 |
| 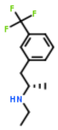 | 0 | 1061 | C12H16F3N | 2.0181 | 32 | 4 | -1.3062 | -4.8562 | -17.2 | 3.9744 | 11.09 | 19.207 | -52.09288 | 1.06889 | 1 |
| 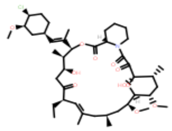 | 0 | 230 | C43H68ClNO11 | 2.0519 | 124 | 8 | -0.6462 | -11.143 | -33.65 | 0 | 26.574 | 30.414 | -188.4032 | 2.72587 | 1 |
| 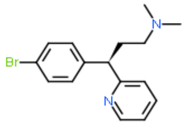 | 0 | 716 | 835 | 2.0712 | 38 | 5 | -0.7662 | -6.447 | -20.67 | 4.1043 | 13.491 | 20.335 | -86.3398 | 1.42827 | 1 |
| 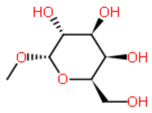 | 0 | 1858 | C7H14O6 | 2.0752 | 27 | 6 | -5.1707 | -2.4204 | -9.421 | 0.9526 | 16.201 | 14.965 | -42.63853 | 0.85719 | 1 |
| 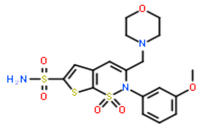 | 0 | 3250 | INL | 2.0763 | 51 | 5 | -0.8446 | -5.8262 | -26.78 | 6.4036 | 20.246 | 19.495 | -141.5534 | 2.01251 | 1 |

| | | | | | | | | | | | | | | |
|---|---|---|---|---|---|---|---|---|---|---|---|---|---|---|
| 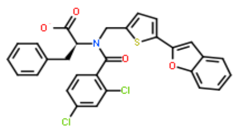 | 0 | 2072 | C29H21Cl2NO4S | 2.0764 | 57 | 7 | -0.7725 | -8.1589 | -32.85 | 7.3396 | 19.989 | 28.409 | -144.7641 | 2.12569 | 1 |
| 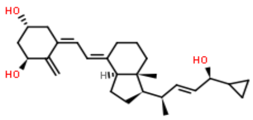 | 0 | 2042 | C27H40O3 | 2.0809 | 70 | 7 | -7.6073 | -7.5459 | -17.62 | 8.6035 | 25.093 | 23.966 | -132.8489 | 1.67027 | 1 |
| 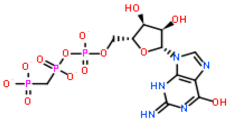 | 0 | 3366 | C11H18N5O13P3 | 2.0886 | 46 | 11 | -10.217 | -2.9459 | -29.31 | 0 | 32.95 | 42.281 | -132.5309 | 1.79417 | 1 |
| 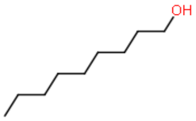 | 0 | 2826 | C9H20O | 2.0972 | 30 | 8 | -3.9686 | -4.415 | -12.41 | 3.2472 | 12.415 | 17.342 | -72.1218 | 1.07278 | 1 |
| 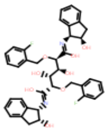 | 0 | 2352 | C38H38F2N2O8 | 2.1293 | 88 | 19 | -2.0346 | -8.9403 | -37.68 | 0 | 29.902 | 25.938 | -164.2509 | 2.50201 | 1 |
| 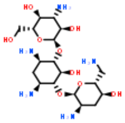 | 0 | 570 | m | 2.1605 | 69 | 16 | -8.6341 | -4.4483 | -25.97 | 8.7369 | 33.101 | 20.138 | -105.8573 | 1.5674 | 1 |
| 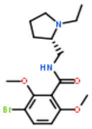 | 0 | 299 | 409 | 2.1701 | 45 | 3 | -0.8554 | -6.509 | -19.55 | 6.8683 | 13.65 | 18.321 | -107.7117 | 1.41106 | 1 |
| 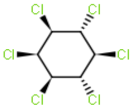 | 0 | 321 | C6H6Cl6 | 2.1879 | 18 | 0 | 0 | -4.8095 | -7.042 | 0.53 | 8.0615 | 9.2246 | -61.02235 | 0.98199 | 1 |
| 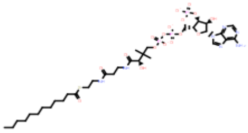 | 0 | 2944 | C33H58N7O17P3S | 2.2145 | 115 | 31 | -12.827 | -7.3355 | -44.34 | 0 | 42.655 | 50.502 | -175.1952 | 2.91088 | 1 |
| 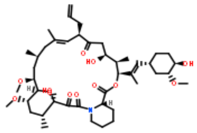 | 0 | 744 | m | 2.2281 | 126 | 10 | -5.2804 | -8.9942 | -32.96 | 19.364 | 26.571 | 25.8 | -141.4418 | 3.06249 | 1 |
| 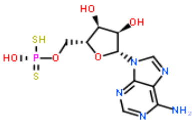 | 0 | 2706 | C10H14N5O5PS2 | 2.2346 | 37 | 8 | -6.9082 | -3.6813 | -15.95 | 2.4489 | 25.165 | 19.68 | -121.2379 | 1.78833 | 1 |

| | | | | | | | | | | | | | | | |
|---|---|---|---|---|---|---|---|---|---|---|---|---|---|---|---|
| 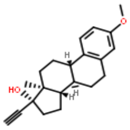 | 0 | 1185 | C21H26O2 | 2.2425 | 49 | 1 | -1.1636 | -5.6237 | -18 | 0.5597 | 14.719 | 22.703 | -101.83 | 1.45949 | 1 |
| 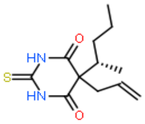 | 0 | 1024 | 1154 | 2.2448 | 35 | 5 | -2.7901 | -3.6746 | -15.35 | 4.6361 | 13.914 | 16.506 | -97.27143 | 1.24038 | 1 |
| 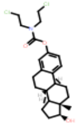 | 0 | 1066 | C23H31Cl2NO3 | 2.2809 | 60 | 6 | -3.5556 | -7.3405 | -19.13 | 7.5459 | 17.861 | 20.907 | -132.5886 | 1.97044 | 1 |
| 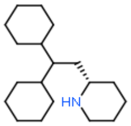 | 0 | 946 | 1074 | 2.3314 | 55 | 4 | 0 | -6.1371 | -20.38 | 1.5832 | 13.195 | 20.576 | -88.63226 | 1.24963 | 1 |
| 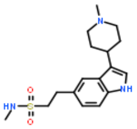 | 0 | 830 | 952 | 2.341 | 48 | 5 | -0.3128 | -5.6751 | -20.9 | 4.545 | 14.315 | 17.232 | -89.25987 | 1.53547 | 1 |
| 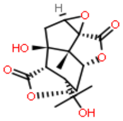 | 0 | 356 | 466 | 2.3417 | 40 | 3 | -4.0257 | -3.0566 | -16.21 | 0.6163 | 19.027 | 21.739 | -85.14088 | 1.17017 | 1 |
| 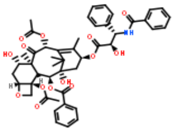 | 0 | 1098 | C47H51NO14 | 2.3435 | 113 | 13 | -5.404 | -10.198 | -39.75 | 21.024 | 32.241 | 27.274 | -159.5015 | 2.89912 | 1 |
| 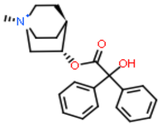 | 0 | 654 | C22H26NO3 | 2.3542 | 52 | 5 | -2.1563 | -6.2574 | -23.4 | 3.7902 | 17.577 | 25.674 | -116.8256 | 1.75011 | 1 |
| 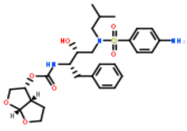 | 0 | 1131 | 1264 | 2.3674 | 75 | 13 | -3.2817 | -7.6347 | -27.84 | 6.7522 | 21.891 | 23.262 | -129.8828 | 1.85881 | 1 |
| 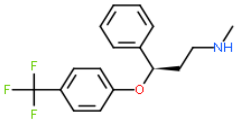 | 0 | 362 | 472 | 2.3954 | 40 | 6 | -0.4754 | -6.4598 | -23.69 | 2.8279 | 13.554 | 24.48 | -123.0395 | 1.44667 | 1 |
| 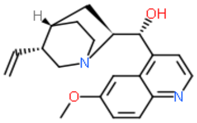 | 0 | 1175 | C32H36N4O5 | 2.4126 | 48 | 4 | -2.7859 | -6.3331 | -15.94 | 2.8255 | 17.198 | 19.162 | -121.9177 | 1.50887 | 1 |

| | | | | | | | | | | | | | | | |
|---|---|---|---|---|---|---|---|---|---|---|---|---|---|---|---|
| 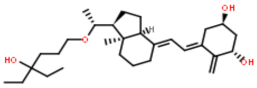 | 0 | 3118 | C29H48O4 | 2.437 | 81 | 11 | -2.3005 | -7.0022 | -20.48 | 2.9176 | 17.866 | 17.69 | -136.9453 | 1.80784 | 1 |
| 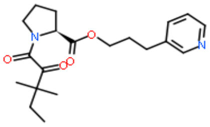 | 0 | 1715 | GPI | 2.4446 | 54 | 9 | -0.9235 | -6.778 | -29.93 | 2.9966 | 17.955 | 28.163 | -134.176 | 1.59221 | 1 |
| 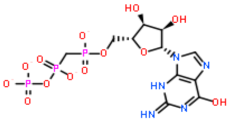 | 0 | 3191 | C11H18N5O13P3 | 2.4484 | 46 | 11 | -15.664 | -3.1912 | -28.6 | 5.2273 | 31.661 | 59.602 | -123.8108 | 1.58617 | 1 |
| 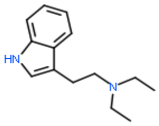 | 0 | 1263 | 1460 | 2.4521 | 36 | 5 | 0 | -5.4479 | -21.72 | 2.0127 | 13.449 | 20.5 | -82.73988 | 1.28718 | 1 |
| 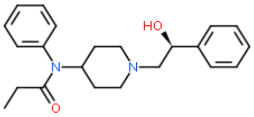 | 0 | 1257 | 1453 | 2.4662 | 54 | 8 | -2.4794 | -6.0734 | -22.17 | 0.3388 | 17.785 | 25.208 | -107.4314 | 2.07775 | 1 |
| 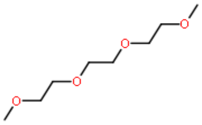 | 0 | 1836 | PG5 | 2.4766 | 30 | 9 | -4.5227 | -4.9588 | -13.36 | 1.7133 | 15.297 | 19.236 | -69.98692 | 1.40652 | 1 |
| 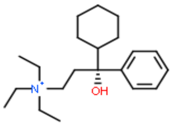 | 0 | 395 | 505 | 2.4894 | 59 | 9 | -1.0751 | -6.5541 | -19.86 | 1.0326 | 13.627 | 20.025 | -102.6745 | 1.4731 | 1 |
| 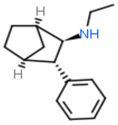 | 0 | 1266 | 1463 | 2.492 | 37 | 3 | 0 | -5.2256 | -13.82 | 0.7268 | 10.56 | 14.631 | -89.88014 | 1.18947 | 1 |
| 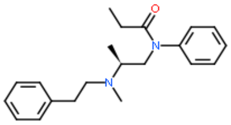 | 0 | 1304 | 1502 | 2.5167 | 52 | 9 | -2.7196 | -6.8924 | -21.98 | 4.7719 | 15.671 | 24.215 | -104.7794 | 1.63593 | 1 |
| 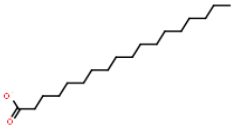 | 0 | 2874 | C18H36O2 | 2.523 | 55 | 16 | -2.8236 | -7.3734 | -19.35 | 1.925 | 19.491 | 13.002 | -116.4947 | 2.16617 | 1 |
| 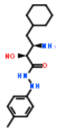 | 0 | 2774 | AO2 | 2.5302 | 49 | 7 | -3.659 | -5.4184 | -25.53 | 1.825 | 21.343 | 29.76 | -106.2587 | 1.47092 | 1 |

| | | | | | | | | | | | | | | | |
|---|---|---|---|---|---|---|---|---|---|---|---|---|---|---|---|
| 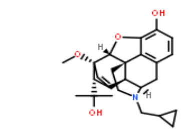 | 0 | 1283 | C26H33NO4 | 2.5434 | 64 | 5 | -1.7465 | -7.0148 | -21.18 | 7.9067 | 15.211 | 20.737 | -123.3195 | 1.57928 | 1 |
| 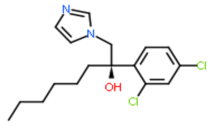 | 0 | 2340 | PFZ | 2.5495 | 44 | 9 | -2.9132 | -7.0428 | -22.21 | 2.6269 | 18.47 | 24.274 | -121.7269 | 1.53057 | 1 |
| 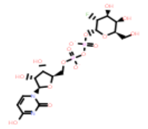 | 0 | 2672 | C15H23FN2O16P2 | 2.5685 | 57 | 15 | -12.708 | -3.4275 | -26.74 | 7.4808 | 29.227 | 42.958 | -171.2677 | 2.08157 | 1 |
| 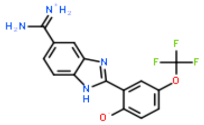 | 0 | 3247 | 785 | 2.5705 | 35 | 0 | -5.6539 | -4.7715 | -20.21 | 1.7573 | 21.438 | 35.074 | -90.8826 | 1.61258 | 1 |
| 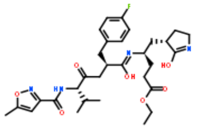 | 0 | 2055 | C31H41FN4O7 | 2.572 | 84 | 17 | -5.3932 | -7.5009 | -33.34 | 8.4763 | 23.699 | 32.616 | -174.0632 | 2.22558 | 1 |
| 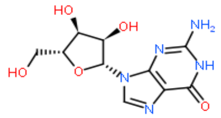 | 0 | 2560 | Guanosine.mol | 2.5759 | 33 | 5 | -6.8397 | -2.4958 | -19.82 | 2.4513 | 27.855 | 24.256 | -95.43536 | 1.33063 | 1 |
| 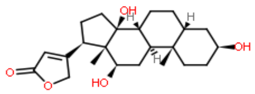 | 0 | 3318 | C23H34O5 | 2.5834 | 62 | 4 | -5.2646 | -5.5649 | -13.53 | 3.6417 | 21.046 | 19.388 | -149.9623 | 1.41849 | 1 |
| 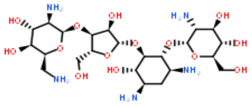 | 0 | 1228 | 1421 | 2.6091 | 87 | 22 | -13.066 | -4.6427 | -27.63 | 0 | 39.599 | 35.619 | -177.4296 | 2.46264 | 1 |
| 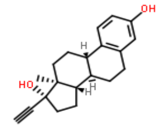 | 0 | 854 | C20H24O2 | 2.6118 | 46 | 1 | -1.1244 | -5.1984 | -17.23 | 0.9238 | 14.712 | 21.18 | -98.71228 | 1.28716 | 1 |
| 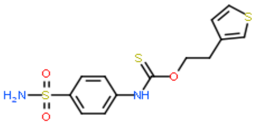 | 0 | 3009 | SUA | 2.6124 | 35 | 6 | -1.0846 | -4.8396 | -21.67 | 2.6507 | 14.048 | 22.037 | -115.2002 | 1.81841 | 1 |
| 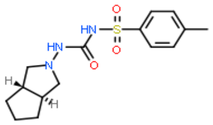 | 0 | 991 | 1120 | 2.6179 | 43 | 5 | -2.2803 | -5.5517 | -23.03 | 3.8234 | 17.651 | 25.154 | -97.23861 | 1.54083 | 1 |

| | | | | | | | | | | | | | | | |
|---|---|---|---|---|---|---|---|---|---|---|---|---|---|---|---|
| 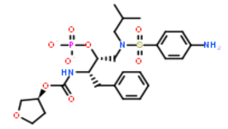 | 0 | 1154 | 1319 | 2.6234 | 73 | 14 | -14.142 | -7.5614 | -26.52 | 11.1 | 26.226 | 53.428 | -167.0817 | 2.35026 | 1 |
| 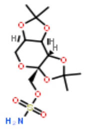 | 0 | 168 | 273 | 2.6261 | 43 | 4 | -1.214 | -4.3381 | -18.59 | 1.3721 | 15.091 | 19.172 | -61.72618 | 1.14477 | 1 |
| 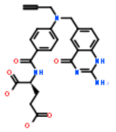 | 0 | 3199 | CB3 | 2.6276 | 56 | 8 | -6.9032 | -6.2483 | -26.89 | 14.178 | 26.258 | 27.526 | -159.5129 | 1.86685 | 1 |
| 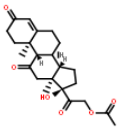 | 0 | 1198 | C23H30O6 | 2.6286 | 59 | 4 | -4.7197 | -5.7311 | -20 | 5.8098 | 22.316 | 23.92 | -155.3088 | 1.89197 | 1 |
| 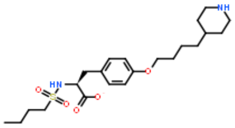 | 0 | 658 | 775 | 2.629 | 65 | 13 | -2.9336 | -6.7915 | -27.12 | 10.112 | 21.189 | 18.132 | -78.08029 | 1.8323 | 1 |
| 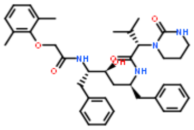 | 0 | 1394 | 1601 | 2.65 | 94 | 15 | -3.9777 | -9.071 | -34.41 | 8.4233 | 26.774 | 28.884 | -181.89 | 2.40945 | 1 |
| 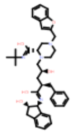 | 0 | 1771 | C39H48N4O5 | 2.6528 | 96 | 16 | -2.941 | -9.6453 | -37.19 | 7.8856 | 23.276 | 33.263 | -129.2188 | 2.81412 | 1 |
| 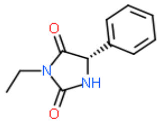 | 0 | 637 | 754 | 2.6622 | 27 | 2 | -1.8371 | -3.9182 | -16.91 | 1.7463 | 14.272 | 21.049 | -93.20895 | 1.21363 | 1 |
| 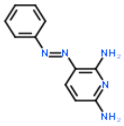 | 0 | 1244 | 1438 | 2.663 | 27 | 1 | -2.3126 | -4.8958 | -19.36 | 1.7749 | 18.827 | 23.286 | -90.13111 | 1.30784 | 1 |
| 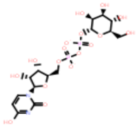 | 0 | 2157 | C15H24N2O17P2 | 2.6751 | 58 | 16 | -9.1901 | -3.6538 | -27.37 | 7.7111 | 32.271 | 26.237 | -140.1778 | 1.83767 | 1 |
| 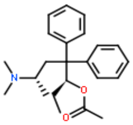 | 0 | 1239 | 1433 | 2.6819 | 57 | 8 | 0 | -6.881 | -23.72 | 2.7732 | 12.833 | 22.609 | -111.4959 | 1.31583 | 1 |

| | | | | | | | | | | | | | | | |
|---|---|---|---|---|---|---|---|---|---|---|---|---|---|---|---|
| 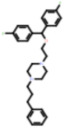 | 0 | 3344 | C28H32F2N2O | 2.6928 | 65 | 10 | -0.7323 | -8.9915 | -30.97 | 6.5934 | 16.881 | 28.669 | -152.752 | 2.11346 | 1 |
| 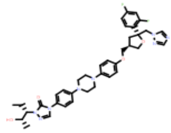 | 0 | 1130 | C37H42F2N8O4 | 2.7001 | 93 | 9 | -5.6376 | -10.355 | -36.38 | 14.224 | 30.891 | 35.12 | -209.3136 | 3.21363 | 1 |
| 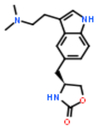 | 0 | 209 | C16H21N3O2 | 2.7008 | 42 | 5 | -2.3716 | -5.1056 | -22.14 | 1.1599 | 17.255 | 26.71 | -74.02901 | 1.43782 | 1 |
| 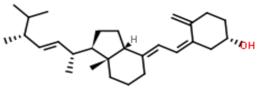 | 0 | 49 | 153 | 2.725 | 73 | 5 | -2.4816 | -7.4124 | -18.79 | 8.5234 | 17.449 | 17.513 | -121.2762 | 2.23289 | 1 |
| 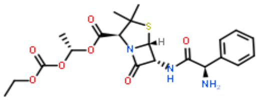 | 0 | 1395 | 1602 | 2.73 | 59 | 8 | -5.3035 | -6.685 | -25.62 | 9.1503 | 29.431 | 21.038 | -118.9779 | 1.897 | 1 |
| 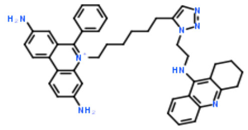 | 0 | 2700 | C42H45N8 | 2.7316 | 95 | 11 | -4.8673 | -9.1455 | -42.83 | 26.132 | 28.619 | 30.698 | -208.0623 | 2.7227 | 1 |
| 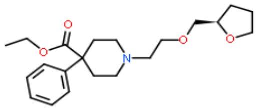 | 0 | 1267 | 1464 | 2.7601 | 57 | 8 | -4.1986 | -7.2783 | -22.32 | 7.987 | 20.276 | 23.912 | -156.2425 | 2.08247 | 1 |
| 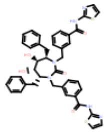 | 0 | 2418 | C41H38N6O5S2 | 2.7675 | 92 | 10 | -2.8751 | -9.0155 | -41.01 | 13.743 | 31.046 | 29.632 | -185.5489 | 2.51002 | 1 |
| 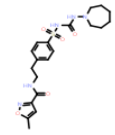 | 0 | 1144 | 1289 | 2.7928 | 58 | 8 | -3.584 | -6.5587 | -24.02 | 5.1757 | 22.508 | 23.473 | -145.3289 | 2.01769 | 1 |
| 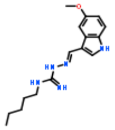 | 0 | 951 | C16H23N5O | 2.7946 | 45 | 6 | -4.0299 | -4.9848 | -25.34 | 9.9602 | 19.629 | 26.318 | -81.74009 | 1.70961 | 1 |
| 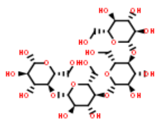 | 0 | 2432 | C24H42O21 | 2.8151 | 87 | 24 | -10.373 | -5.2998 | -28.64 | 6.0924 | 35.206 | 25.441 | -167.4417 | 2.40357 | 1 |

| | | | | | | | | | | | | | | | |
|---|---|---|---|---|---|---|---|---|---|---|---|---|---|---|---|
| 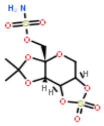 | 0 | 2594 | C9H15NO10S2 | 2.8189 | 37 | 4 | -2.3781 | -3.1194 | -12.64 | 3.0735 | 17.146 | 9.7874 | -84.07481 | 1.23394 | 1 |
| 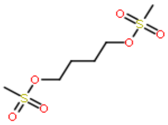 | 0 | 884 | C6H14O6S2 | 2.8472 | 28 | 7 | 0 | -4.1004 | -18.02 | 1.2395 | 11.002 | 15.784 | -96.19769 | 1.23642 | 1 |
| 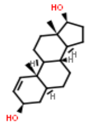 | 0 | 1305 | C19H30O2 | 2.8492 | 51 | 2 | -1.3713 | -5.2421 | -14.43 | 1.494 | 15.039 | 16.491 | -122.7442 | 1.70215 | 1 |
| 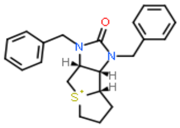 | 0 | 987 | 1116 | 2.8588 | 51 | 4 | 0 | -6.7152 | -27.58 | 7.7659 | 10.625 | 30.172 | -112.3483 | 1.27387 | 1 |
| 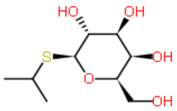 | 0 | 1637 | C9H18O5S | 2.8982 | 33 | 7 | -3.1943 | -3.9907 | -15.64 | 4.2512 | 16.549 | 15.348 | -110.8241 | 1.32499 | 1 |
| 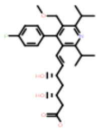 | 0 | 329 | 439 | 2.9066 | 66 | 12 | -5.0418 | -7.3154 | -22.15 | 7.5243 | 21.628 | 22.397 | -173.5983 | 1.8104 | 1 |
| 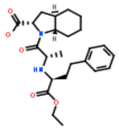 | 0 | 406 | 519 | 2.9452 | 64 | 10 | -4.9719 | -6.8291 | -27.81 | 7.5625 | 21.772 | 31.886 | -165.3839 | 2.11867 | 1 |
| 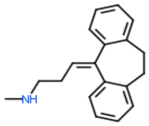 | 0 | 427 | 540 | 2.9558 | 41 | 3 | -1.4079 | -5.6377 | -20.79 | 4.7997 | 13.597 | 24.551 | -73.6295 | 1.17397 | 1 |
| 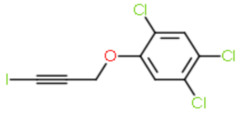 | 0 | 674 | 793 | 2.9674 | 18 | 2 | -1.1121 | -5.9948 | -15.09 | 1.7358 | 11.138 | 21.651 | -50.49226 | 1.35771 | 1 |
| 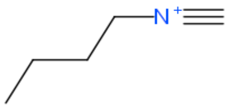 | 0 | 1603 | C5H9N | 2.9754 | 15 | 2 | 0 | -3.6663 | -8.287 | 0.3863 | 7.4589 | 10.14 | -30.88286 | 0.61017 | 1 |
| 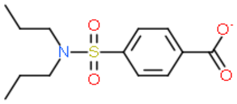 | 0 | 907 | 1032 | 2.9821 | 37 | 7 | -1.9613 | -4.8593 | -19.92 | 5.387 | 14.048 | 19.931 | -103.5309 | 1.34689 | 1 |

| | | | | | | | | | | | | | | | |
|---|---|---|---|---|---|---|---|---|---|---|---|---|---|---|---|
| 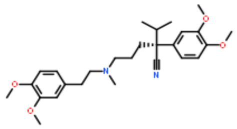 | 0 | 548 | 661 | 3.0524 | 71 | 10 | -2.2722 | -7.8812 | -26.13 | 6.6001 | 20.866 | 22.129 | -152.0217 | 1.85896 | 1 |
| 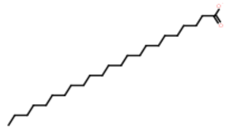 | 0 | 3163 | C23H46O2 | 3.0887 | 70 | 21 | -2.8953 | -9.0585 | -24.66 | 4.1547 | 20.923 | 15.695 | -153.5921 | 2.74626 | 1 |
| 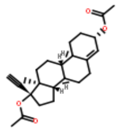 | 0 | 704 | 823 | 3.0903 | 60 | 2 | -1.1254 | -7.4576 | -21.07 | 2.1408 | 19.791 | 22.243 | -137.5095 | 1.96642 | 1 |
| 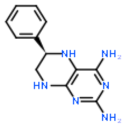 | 0 | 2611 | AP6 | 3.1081 | 32 | 1 | -2.0282 | -4.661 | -17.73 | 2.1924 | 20.492 | 17.889 | -81.76512 | 1.32074 | 1 |
| 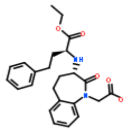 | 0 | 429 | C24H28N2O5 | 3.1089 | 58 | 9 | -2.9419 | -7.3449 | -26.66 | 2.4437 | 20.057 | 30.649 | -119.5313 | 1.65585 | 1 |
| 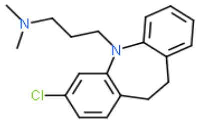 | 0 | 1111 | 1242 | 3.1107 | 45 | 4 | -1.7046 | -6.3741 | -17.48 | 3.3787 | 11.565 | 24.356 | -67.29179 | 1.22618 | 1 |
| 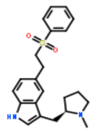 | 0 | 111 | C22H26N2O2S | 3.1157 | 53 | 6 | -0.7847 | -5.9686 | -25.24 | 4.9678 | 16.691 | 23.095 | -119.4852 | 1.76642 | 1 |
| 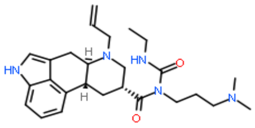 | 0 | 143 | 248 | 3.1184 | 70 | 11 | -1.9706 | -7.9524 | -33.6 | 7.6872 | 18.789 | 32.916 | -154.262 | 1.90333 | 1 |
| 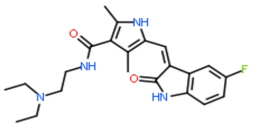 | 0 | 1134 | 1268 | 3.139 | 56 | 5 | -1.9193 | -7.7262 | -26.13 | 6.9978 | 21.353 | 24.378 | -134.8955 | 1.80114 | 1 |
| 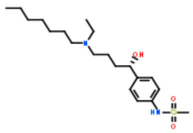 | 0 | 202 | m | 3.1419 | 62 | 15 | -3.3285 | -6.4191 | -27.84 | 9.9192 | 19.342 | 21.583 | -120.7664 | 1.99539 | 1 |
| 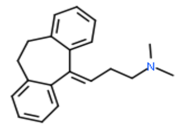 | 0 | 215 | 321 | 3.1676 | 44 | 3 | 0 | -6.0255 | -21.5 | 4.852 | 11.67 | 23.09 | -73.4495 | 1.14745 | 1 |

| | | | | | | | | | | | | | | | |
|---|---|---|---|---|---|---|---|---|---|---|---|---|---|---|---|
| 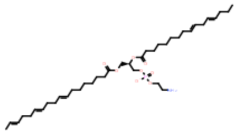 | 0 | 2738 | C39H68NO8P | 3.1739 | 116 | 35 | -11.479 | -9.4716 | -40.78 | 21.096 | 29.811 | 35.342 | -194.58 | 2.7829 | 1 |
| 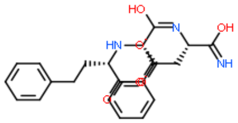 | 0 | 2049 | C23H27N3O6 | 3.1766 | 57 | 15 | -6.3608 | -5.9931 | -30.31 | 6.3476 | 26.881 | 31.254 | -129.8933 | 1.83282 | 1 |
| 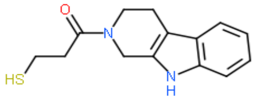 | 0 | 3046 | MHC | 3.21 | 34 | 4 | -0.9245 | -4.5235 | -19.26 | 0 | 12.723 | 24.085 | -109.5242 | 1.44973 | 1 |
| 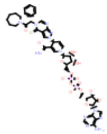 | 0 | 2524 | C40H44N12O15P2S | 3.2148 | 113 | 17 | -17.866 | -6.8076 | -43.88 | 21.681 | 38.211 | 68.161 | -204.3347 | 2.77332 | 1 |
| 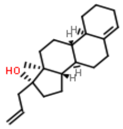 | 0 | 1238 | 1431 | 3.2379 | 54 | 3 | -0.5603 | -6.3351 | -17.97 | 1.13 | 13.664 | 21.425 | -137.8593 | 1.7843 | 1 |
| 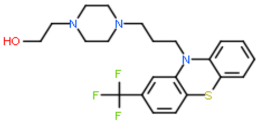 | 0 | 510 | 623 | 3.2813 | 56 | 8 | -0.6412 | -4.9574 | -24.82 | 4.7446 | 14.557 | 21.945 | -50.58732 | 1.66256 | 1 |
| 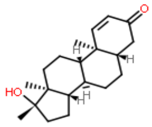 | 0 | 1368 | C20H30O2 | 3.2903 | 52 | 1 | -1.929 | -5.1985 | -12.17 | 1.8258 | 14.218 | 17.377 | -130.7128 | 1.73623 | 1 |
| 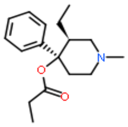 | 0 | 1301 | 1499 | 3.3146 | 45 | 4 | -1.0895 | -5.7294 | -16.94 | 2.3971 | 12.268 | 20.921 | -107.4158 | 1.5568 | 1 |
| 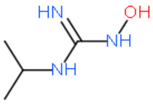 | 0 | 1900 | C4H11N3O | 3.3287 | 19 | 4 | -2.3132 | -2.5546 | -10.33 | 0 | 12.689 | 14.108 | -34.38758 | 0.71602 | 1 |
| 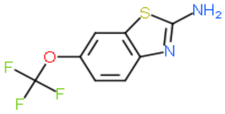 | 0 | 623 | 740 | 3.3432 | 20 | 1 | -0.8807 | -4.2203 | -16.15 | 0.5367 | 13.79 | 20.128 | -80.91322 | 1.08391 | 1 |
| 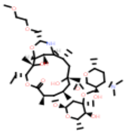 | 0 | 832 | C42H78N2O14 | 3.3728 | 136 | 16 | -2.3169 | -10.815 | -31.2 | 7.8447 | 24.481 | 22.753 | -144.1268 | 3.191 | 1 |

| | | | | | | | | | | | | | | | |
|---|---|---|---|---|---|---|---|---|---|---|---|---|---|---|---|
| 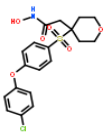 | 0 | 1809 | CBP | 3.3806 | 48 | 8 | -3.2949 | -6.6841 | -20.45 | 2.2623 | 19.708 | 23.791 | -126.1912 | 1.81693 | 1 |
| 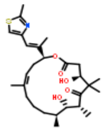 | 0 | 1646 | C27H41NO5S | 3.3909 | 75 | 3 | -2.3592 | -7.7163 | -25.58 | 8.2117 | 22.347 | 25.192 | -123.1051 | 1.90186 | 1 |
| 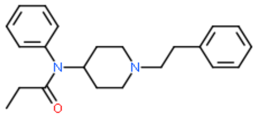 | 0 | 694 | 813 | 3.4062 | 53 | 7 | -0.8267 | -7.3039 | -26.69 | 6.6198 | 16.013 | 25.608 | -124.2401 | 1.71165 | 1 |
| 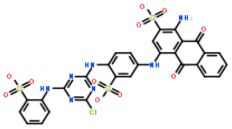 | 0 | 2355 | C29H20ClN7O11S3 | 3.4587 | 68 | 3 | -8.6591 | -6.6282 | -37.16 | 11.389 | 35.535 | 48.001 | -176.2142 | 2.35419 | 1 |
| 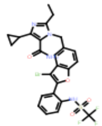 | 0 | 1176 | 1347 | 3.461 | 60 | 6 | -2.7447 | -7.3924 | -29.92 | 4.747 | 22.037 | 33.793 | -152.5103 | 2.11424 | 1 |
| 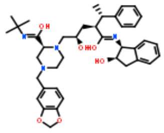 | 0 | 1507 | C39H50N4O6 | 3.4794 | 99 | 16 | -9.0277 | -7.4586 | -28.23 | 17.919 | 26.033 | 29.807 | -157.9984 | 2.94355 | 1 |
| 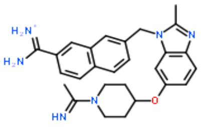 | 0 | 3047 | C27H31N6O | 3.4973 | 65 | 4 | -3.4857 | -6.7681 | -31.43 | 8.3472 | 23.548 | 35.168 | -142.6131 | 2.22874 | 1 |
| 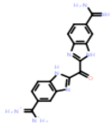 | 0 | 2894 | C17H16N8O | 3.5062 | 42 | 0 | -5.8034 | -3.9282 | -23.77 | 0.7177 | 24.655 | 38.888 | -79.39796 | 1.66075 | 1 |
| 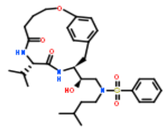 | 0 | 3407 | C30H43N3O6S | 3.52 | 83 | 10 | -2.0776 | -8.1106 | -31.8 | 7.868 | 23.679 | 26.564 | -162.3938 | 2.6415 | 1 |
| 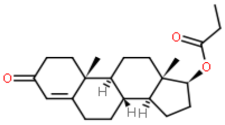 | 0 | 1227 | 1420 | 3.5213 | 57 | 2 | -1.0939 | -5.9169 | -19.99 | 2.1307 | 16.148 | 23.721 | -91.02618 | 1.77626 | 1 |
| 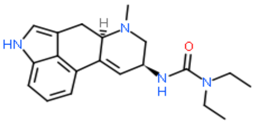 | 0 | 476 | 589 | 3.5341 | 51 | 5 | -1.1397 | -6.9634 | -22.87 | 0.6515 | 17.249 | 26.708 | -137.763 | 1.63685 | 1 |

| | | | | | | | | | | | | | | | |
|---|---|---|---|---|---|---|---|---|---|---|---|---|---|---|---|
| 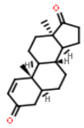 | 0 | 1255 | C19H26O2 | 3.5883 | 47 | 0 | -2.0846 | -4.8653 | -13.95 | 1.9507 | 14.629 | 21.11 | -129.8746 | 1.60753 | 1 |
| 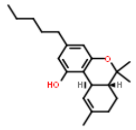 | 0 | 360 | C21H30O2 | 3.6183 | 53 | 4 | 0 | -7.3477 | -23.86 | 2.4971 | 15.518 | 25.559 | -125.2698 | 1.73554 | 1 |
| 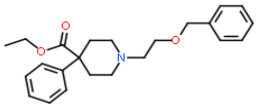 | 0 | 1315 | 1518 | 3.6691 | 56 | 8 | -0.7786 | -7.9753 | -26.99 | 6.6468 | 15.123 | 27.028 | -139.3548 | 1.82526 | 1 |
| 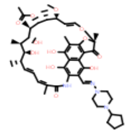 | 0 | 1071 | C47H64N4O12 | 3.6792 | 127 | 6 | -1.6125 | -10.388 | -39.83 | 24.663 | 22.304 | 29.427 | -237.503 | 2.48821 | 1 |
| 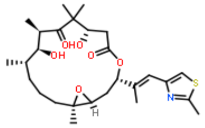 | 0 | 2705 | C27H41NO6S | 3.6882 | 76 | 3 | -2.7468 | -7.003 | -25.57 | 13.188 | 19.926 | 24.613 | -142.1127 | 1.90851 | 1 |
| 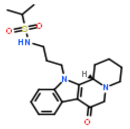 | 0 | 1731 | WAC | 3.7219 | 57 | 6 | 0 | -6.8089 | -27.95 | 5.4875 | 15.514 | 26.822 | -157.6687 | 1.74772 | 1 |
| 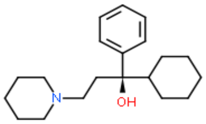 | 0 | 268 | 376 | 3.7558 | 53 | 6 | -1.9211 | -6.3639 | -17.94 | 3.7246 | 15.471 | 20.277 | -132.4152 | 1.47517 | 1 |
| 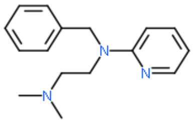 | 0 | 673 | 792 | 3.7664 | 40 | 5 | 0 | -6.2681 | -22.87 | 3.0242 | 12.65 | 25.045 | -102.0643 | 1.39041 | 1 |
| 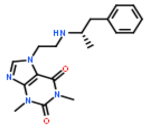 | 0 | 1285 | 1482 | 3.7787 | 48 | 6 | -1.7097 | -6.8106 | -22.34 | 1.2847 | 21.871 | 21.566 | -121.5671 | 1.63971 | 1 |
| 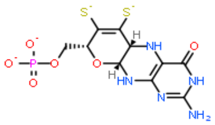 | 0 | 1894 | C10H12MoN5O8PS2 | 3.794 | 34 | 3 | -11.484 | -2.3762 | -13.06 | 0 | 26.886 | 38.693 | -116.2012 | 1.51496 | 1 |
| 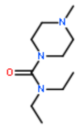 | 0 | 595 | 711 | 3.8794 | 35 | 4 | -0.8598 | -4.8774 | -14.95 | 1.2455 | 11.823 | 18.573 | -80.18476 | 1.28502 | 1 |

| | | | | | | | | | | | | | | | |
|---|---|---|---|---|---|---|---|---|---|---|---|---|---|---|---|
| 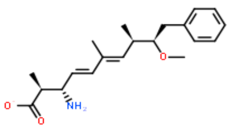 | 0 | 1625 | C20H29NO3 | 3.9223 | 52 | 9 | -4.9967 | -6.162 | -20.35 | 6.8529 | 21.544 | 23.293 | -110.6443 | 1.76 | 1 |
| 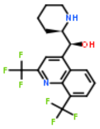 | 0 | 251 | 358 | 3.9443 | 42 | 4 | 0 | -6.1253 | -22.41 | 0.7509 | 13.752 | 26.2 | -96.53194 | 1.40226 | 1 |
| 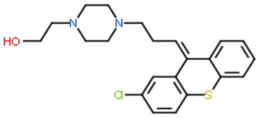 | 0 | 1416 | 1624 | 3.9598 | 52 | 6 | -2.1316 | -7.5349 | -25.94 | 5.7481 | 19.272 | 28.536 | -115.0723 | 1.60222 | 1 |
| 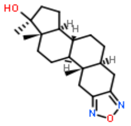 | 0 | 1312 | 1514 | 4.0115 | 54 | 1 | -1.9439 | -5.304 | -12.58 | 1.3813 | 15.474 | 18.224 | -131.0972 | 1.87188 | 1 |
| 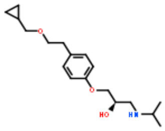 | 0 | 90 | C18H29NO3 | 4.0183 | 51 | 11 | -2.4642 | -7.2713 | -25.78 | 5.9788 | 18.971 | 24.979 | -135.7598 | 1.9266 | 1 |
| 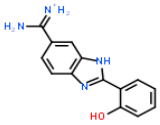 | 0 | 2030 | 122 | 4.0266 | 32 | 0 | -2.6426 | -4.1482 | -20.72 | 2.2943 | 19.793 | 27.26 | -74.81207 | 1.24128 | 1 |
| 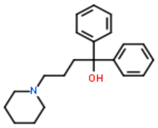 | 0 | 1100 | 1231 | 4.0296 | 50 | 7 | 0 | -6.6289 | -26.3 | 4.7715 | 14.883 | 25.064 | -128.0682 | 1.54794 | 1 |
| 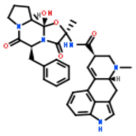 | 0 | 582 | C33H35N5O5 | 4.0342 | 78 | 5 | -3.6299 | -7.8146 | -29.34 | 4.3065 | 25.471 | 35.008 | -188.1709 | 2.47928 | 1 |
| 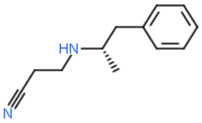 | 0 | 1346 | 1550 | 4.0344 | 30 | 5 | -1.9051 | -4.8705 | -15.86 | 1.6094 | 14.403 | 20.008 | -75.92563 | 1.48249 | 1 |
| 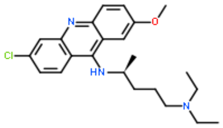 | 0 | 975 | 1103 | 4.0358 | 58 | 7 | -2.0435 | -7.4799 | -25.07 | 5.9958 | 17.208 | 28.163 | -146.0434 | 1.61455 | 1 |
| 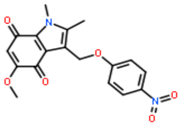 | 0 | 2136 | 936 | 4.0624 | 42 | 4 | -0.7209 | -5.3571 | -23.52 | 2.0151 | 18.625 | 23.36 | -99.03525 | 1.64888 | 1 |

| | | | | | | | | | | | | | | | |
|---|---|---|---|---|---|---|---|---|---|---|---|---|---|---|---|
| 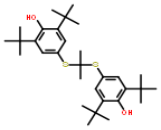 | 0 | 1392 | C31H48O2S2 | 4.0735 | 83 | 6 | -0.7297 | -8.3665 | -30.73 | 5.4991 | 22.221 | 28.32 | -159.5274 | 2.54382 | 1 |
| 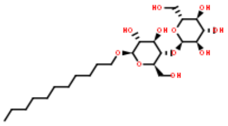 | 0 | 2404 | C23H44O11 | 4.1344 | 78 | 22 | -11.96 | -7.2399 | -24.44 | 15.929 | 33.639 | 23.789 | -142.6113 | 2.39165 | 1 |
| 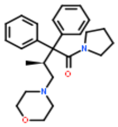 | 0 | 1325 | C25H32N2O2 | 4.1401 | 61 | 7 | -2.1504 | -6.5058 | -21.51 | 4.0597 | 17.572 | 23.681 | -143.515 | 1.83347 | 1 |
| 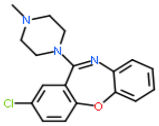 | 0 | 298 | 408 | 4.1475 | 41 | 1 | 0 | -6.9702 | -22.66 | 3.5207 | 13.169 | 28.615 | -114.4834 | 1.51985 | 1 |
| 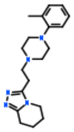 | 0 | 192 | 298 | 4.2056 | 51 | 3 | -1.8232 | -7.2591 | -19.99 | 8.6162 | 17.594 | 20.459 | -122.2592 | 1.76471 | 1 |
| 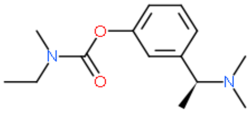 | 0 | 866 | 989 | 4.2251 | 40 | 4 | -0.7297 | -5.9786 | -19.57 | 2.4624 | 14.367 | 22.712 | -115.0793 | 1.53384 | 1 |
| 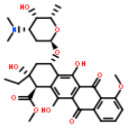 | 0 | 2880 | C31H37NO11 | 4.2526 | 80 | 7 | -4.9689 | -7.4158 | -24.1 | 11.015 | 26.573 | 22.956 | -207.1191 | 2.18804 | 1 |
| 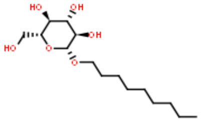 | 0 | 2184 | C15H30O6 | 4.2603 | 51 | 14 | -4.4314 | -5.8916 | -20.28 | 3.1109 | 18.142 | 23.983 | -131.2119 | 1.68505 | 1 |
| 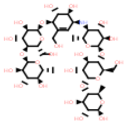 | 0 | 3268 | C37H63NO26 | 4.2713 | 127 | 30 | -16.323 | -7.6965 | -33.52 | 24.87 | 43.661 | 28.145 | -188.4506 | 3.25784 | 1 |
| 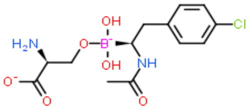 | 0 | 2288 | C13H19BClN2O6 | 4.3155 | 41 | 11 | -5.1334 | -4.5572 | -22.46 | 0 | 25.008 | 26.454 | -75.57304 | 1.51858 | 1 |
| 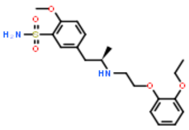 | 0 | 591 | 706 | 4.3531 | 56 | 9 | -1.3498 | -6.7092 | -28.8 | 4.1827 | 20.944 | 26.43 | -114.1295 | 1.91141 | 1 |

| | | | | | | | | | | | | | | | |
|---|---|---|---|---|---|---|---|---|---|---|---|---|---|---|---|
| 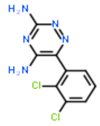 | 0 | 442 | 555 | 4.3572 | 23 | 0 | -2.2013 | -4.5659 | -10.75 | 1.7421 | 17.454 | 14.55 | -41.87744 | 0.98119 | 1 |
| 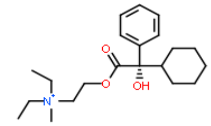 | 0 | 114 | 219 | 4.3702 | 59 | 9 | 0 | -6.9709 | -20.99 | 1.6411 | 12.098 | 22.085 | -78.74598 | 1.40261 | 1 |
| 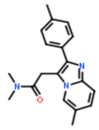 | 0 | 315 | 425 | 4.3753 | 44 | 3 | -0.9233 | -6.942 | -19.57 | 3.6474 | 15.31 | 23.419 | -115.6086 | 1.50608 | 1 |
| 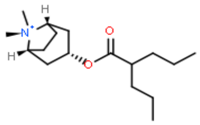 | 0 | 404 | C17H32BrNO2 | 4.3935 | 52 | 6 | 0 | -6.4791 | -19.35 | 1.9748 | 10.489 | 23.376 | -68.16879 | 1.21492 | 1 |
| 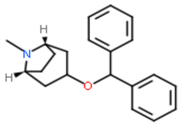 | 0 | 140 | C21H25NO | 4.4354 | 48 | 4 | -1.0302 | -6.9272 | -19.46 | 1.9205 | 14.31 | 25.539 | -135.9079 | 1.51489 | 1 |
| 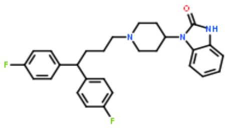 | 0 | 972 | 1100 | 4.5224 | 63 | 7 | -1.9668 | -8.5662 | -28.32 | 4.1732 | 20.987 | 31.985 | -160.9754 | 1.97019 | 1 |
| 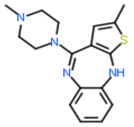 | 0 | 227 | C17H20N4S | 4.5453 | 42 | 1 | -0.219 | -6.4491 | -17.82 | 2.7325 | 13.335 | 22.721 | -112.9476 | 1.43578 | 1 |
| 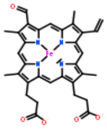 | 0 | 1428 | C33H30FeN4O5 | 4.5533 | 71 | 6 | -5.8689 | -6.9545 | -24.25 | 10.58 | 23.226 | 31.728 | -147.9984 | 1.86697 | 1 |
| 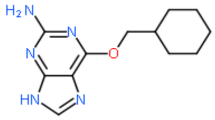 | 0 | 2143 | CMG | 4.6264 | 35 | 2 | -4.2251 | -5.2074 | -13.91 | 3.9242 | 19.406 | 22.127 | -110.1387 | 1.22991 | 1 |
| 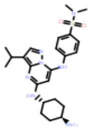 | 0 | 1660 | C23H33N7O2S | 4.6524 | 66 | 5 | -0.4728 | -6.4354 | -28.87 | 4.9212 | 22.339 | 24.728 | -76.38424 | 1.948 | 1 |
| 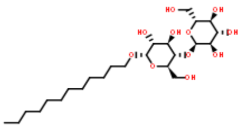 | 0 | 2957 | C24H46O11 | 4.6796 | 81 | 23 | -10.473 | -7.8132 | -25.38 | 7.8397 | 28.537 | 33.159 | -158.362 | 2.26727 | 1 |

| | | | | | | | | | | | | | | | |
|---|---|---|---|---|---|---|---|---|---|---|---|---|---|---|---|
| 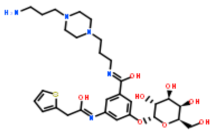 | 0 | 3185 | C29H43N5O8S | 4.7189 | 86 | 18 | -5.6178 | -8.45 | -37.07 | 13.747 | 32.253 | 27.917 | -175.8581 | 2.73879 | 1 |
| 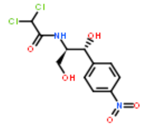 | 0 | 336 | C11H12Cl2N2O5 | 4.7258 | 32 | 8 | -2.875 | -4.2825 | -21.55 | 4.761 | 17.941 | 23.196 | -91.76289 | 1.45155 | 1 |
| 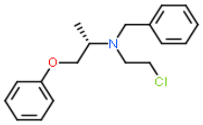 | 0 | 804 | 925 | 4.7294 | 43 | 8 | -1.3437 | -7.1527 | -25.38 | 6.1724 | 14.164 | 29.056 | -101.0589 | 1.63233 | 1 |
| 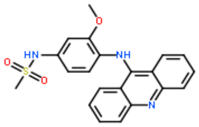 | 0 | 171 | 276 | 4.7341 | 47 | 2 | 0 | -6.9558 | -25.71 | 4.9726 | 16.377 | 28.239 | -142.8308 | 1.83919 | 1 |
| 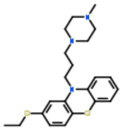 | 0 | 265 | 372 | 4.7496 | 56 | 5 | 0 | -6.6474 | -26.25 | 6.0163 | 16.7 | 24.726 | -134.0172 | 1.63233 | 1 |
| 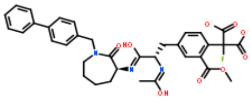 | 0 | 2168 | C35H36FN3O9 | 4.7512 | 82 | 13 | -12.77 | -7.5272 | -39.49 | 17.283 | 32.488 | 59.174 | -240.5012 | 2.42491 | 1 |
| 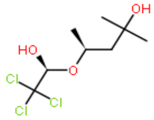 | 0 | 1330 | 1534 | 4.7577 | 29 | 7 | -2.0328 | -4.6523 | -14.12 | 1.2542 | 13.825 | 17.91 | -93.92467 | 1.38086 | 1 |
| 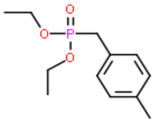 | 0 | 1895 | EBP | 4.7606 | 35 | 6 | 0 | -5.284 | -20.81 | 3.3719 | 10.99 | 23.105 | -94.62894 | 1.24798 | 1 |
| 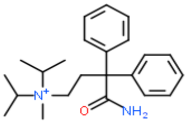 | 0 | 1417 | 1625 | 4.7659 | 59 | 8 | -0.0699 | -6.2392 | -25.57 | 1.5806 | 15.774 | 25.929 | -124.8111 | 1.54176 | 1 |
| 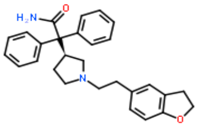 | 0 | 386 | 496 | 4.7664 | 62 | 7 | -1.5803 | -7.5718 | -26.06 | 3.6107 | 20.612 | 27.385 | -138.5891 | 1.88976 | 1 |
| 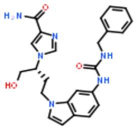 | 0 | 2533 | FR9 | 4.769 | 59 | 10 | -5.5128 | -6.6351 | -29.65 | 13.645 | 27.854 | 26.876 | -137.6886 | 2.1685 | 1 |

| | | | | | | | | | | | | | | | |
|---|---|---|---|---|---|---|---|---|---|---|---|---|---|---|---|
| 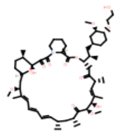 | 0 | 1384 | C53H83NO14 | 4.7964 | 151 | 12 | -8.5748 | -12.691 | -41.24 | 26.726 | 35.59 | 39.856 | -244.0778 | 4.09438 | 1 |
| 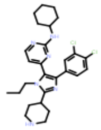 | 0 | 2126 | C27H34Cl2N6 | 4.805 | 69 | 4 | 0 | -8.9435 | -29.34 | 5.9093 | 21.371 | 27.513 | -135.6191 | 1.99867 | 1 |
| 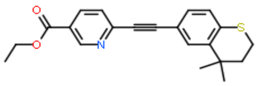 | 0 | 680 | 799 | 4.8063 | 46 | 2 | -1.7575 | -6.5305 | -21.13 | 3.5677 | 16.708 | 28.616 | -101.6642 | 2.13423 | 1 |
| 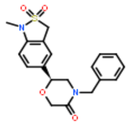 | 0 | 1875 | CP8 | 4.8988 | 46 | 3 | -0.3426 | -5.6901 | -26.52 | 3.0594 | 16.734 | 30.173 | -136.7083 | 1.64836 | 1 |
| 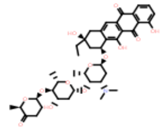 | 0 | 1583 | C40H51NO13 | 4.9076 | 105 | 10 | -3.7697 | -9.9996 | -34.31 | 5.2934 | 36.127 | 29.07 | -205.4565 | 3.4348 | 1 |
| 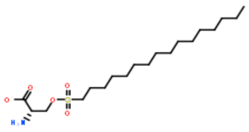 | 0 | 3335 | S1H | 4.9435 | 64 | 20 | -3.8617 | -7.729 | -22.97 | 5.6851 | 21.779 | 16.837 | -128.941 | 2.462 | 1 |
| 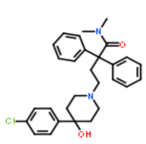 | 0 | 717 | 836 | 4.9531 | 67 | 9 | -4.5234 | -8.3692 | -21.7 | 7.6432 | 20.71 | 27.55 | -150.7277 | 1.99254 | 1 |
| 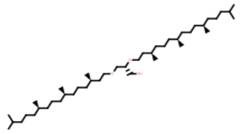 | 0 | 3295 | C43H88O3 | 4.9796 | 134 | 35 | -7.2599 | -10.517 | -36.72 | 17.237 | 23.504 | 27.488 | -200.562 | 2.50571 | 1 |
| 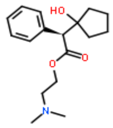 | 0 | 856 | 979 | 5.0532 | 46 | 7 | -1.9568 | -6.1156 | -18.77 | 2.4669 | 16.173 | 22.817 | -138.0052 | 1.67639 | 1 |
| 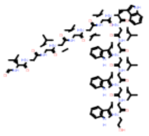 | 0 | 3 | C96H135N19O16 | 5.0788 | 266 | 52 | -10.399 | -14.74 | -78.74 | 34.975 | 43.597 | 53.223 | -323.8509 | 5.38154 | 1 |
| 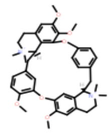 | 0 | 306 | 24244 | 5.0885 | 96 | 0 | -2.1474 | -9.4866 | -29.56 | 14.695 | 20.873 | 33.285 | -98.39706 | 2.28583 | 1 |

| | | | | | | | | | | | | | | | |
|---|---|---|---|---|---|---|---|---|---|---|---|---|---|---|---|
| 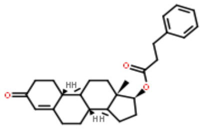 | 0 | 861 | 984 | 5.0994 | 64 | 4 | -1.9694 | -6.928 | -19.66 | 5.738 | 18.842 | 21.836 | -165.8977 | 2.05523 | 1 |
| 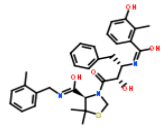 | 0 | 2388 | C32H37N3O5S | 5.1123 | 78 | 12 | -4.5458 | -6.5195 | -32.77 | 6.9428 | 30.244 | 29.855 | -135.5844 | 2.31818 | 1 |
| 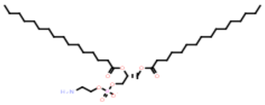 | 0 | 1513 | m22 | 5.1727 | 120 | 38 | -11.384 | -10.596 | -45.49 | 21.193 | 33.629 | 39.123 | -168.7681 | 3.51458 | 1 |
| 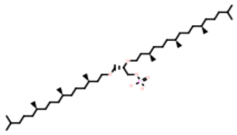 | 0 | 3071 | C43H89O6P | 5.1883 | 137 | 36 | -3.3537 | -10.086 | -39.86 | 18.149 | 25.398 | 13.269 | -141.633 | 3.4636 | 1 |
| 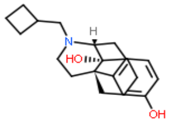 | 0 | 498 | 611 | 5.2164 | 53 | 3 | -2.1764 | -6.133 | -18.56 | 4.2248 | 17.406 | 24.249 | -150.9129 | 1.61863 | 1 |
| 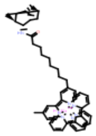 | 0 | 1452 | C50H59N7ORu | 5.2216 | 116 | 10 | -1.3138 | -10.03 | -39.53 | 27.044 | 20.936 | 25.556 | -121.5206 | 2.72496 | 1 |
| 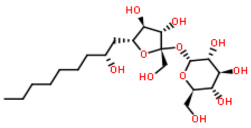 | 0 | 1762 | C20H38O11 | 5.249 | 69 | 20 | -7.6463 | -4.3141 | -19.51 | 7.9374 | 25.8 | 16.487 | -66.71627 | 1.70403 | 1 |
| 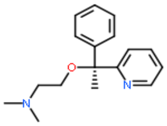 | 0 | 259 | 3162 | 5.2863 | 42 | 6 | -2.1753 | -6.296 | -14.17 | 1.9398 | 14.873 | 20.097 | -113.9818 | 1.47529 | 1 |
| 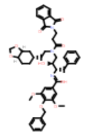 | 0 | 2752 | C46H51N3O10 | 5.3117 | 110 | 17 | -2.6865 | -11.75 | -41.36 | 12.955 | 30.836 | 30.667 | -250.683 | 3.03355 | 1 |
| 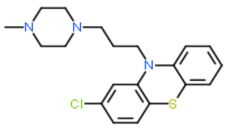 | 0 | 323 | 433 | 5.3228 | 49 | 4 | 0 | -7.2274 | -24.56 | 2.933 | 15.287 | 28.941 | -130.7012 | 1.8021 | 1 |
| 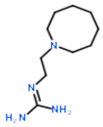 | 0 | 1040 | 1170 | 5.323 | 36 | 3 | -3.3508 | -3.9966 | -9.43 | 0.7095 | 15.969 | 17.736 | -83.38886 | 1.18119 | 1 |

| | | | | | | | | | | | | | | | |
|---|---|---|---|---|---|---|---|---|---|---|---|---|---|---|---|
| 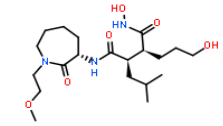 | 0 | 1848 | S80 | 5.3459 | 66 | 15 | -5.2426 | -7.3658 | -23.09 | 2.3855 | 25.092 | 26.347 | -116.2317 | 1.75697 | 1 |
| 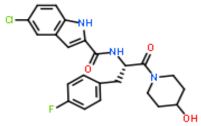 | 0 | 3054 | CHI | 5.3467 | 54 | 6 | -1.1429 | -7.4442 | -26.32 | 11.605 | 20.294 | 20.847 | -156.8673 | 1.65968 | 1 |
| 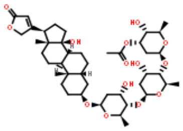 | 0 | 399 | C43H66O14 | 5.3987 | 123 | 12 | -10.578 | -9.9295 | -31.86 | 20.676 | 36.959 | 35.385 | -263.4306 | 3.90307 | 1 |
| 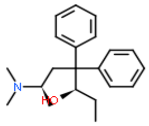 | 0 | 1300 | 1498 | 5.4542 | 52 | 8 | 0 | -6.5221 | -21.4 | 2.1701 | 13.402 | 22.829 | -113.9724 | 1.41292 | 1 |
| 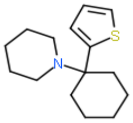 | 0 | 1316 | C15H23NS | 5.4787 | 40 | 2 | 0 | -5.3481 | -15.43 | 1.3991 | 10.693 | 21.861 | -104.4951 | 1.39863 | 1 |
| 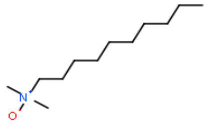 | 0 | 2336 | DDQ | 5.4814 | 41 | 9 | -0.7909 | -6.0488 | -15.59 | 1.501 | 13.104 | 16.667 | -83.33274 | 1.60387 | 1 |
| 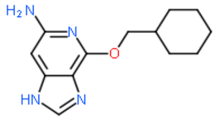 | 0 | 2328 | 207 | 5.5055 | 36 | 2 | -3.8454 | -5.2073 | -13.49 | 3.8321 | 18.687 | 22.268 | -113.2906 | 1.23443 | 1 |
| 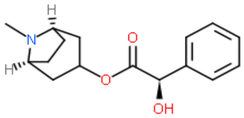 | 0 | 1915 | C16H21NO3 | 5.5312 | 41 | 4 | -1.3015 | -5.338 | -21.51 | 2.3278 | 18.695 | 24.379 | -103.3041 | 1.45843 | 1 |
| 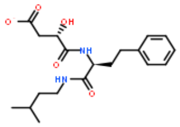 | 0 | 3334 | RL2 | 5.5413 | 53 | 12 | -3.3631 | -5.6708 | -27.28 | 0.208 | 20.799 | 33.644 | -129.336 | 1.71904 | 1 |
| 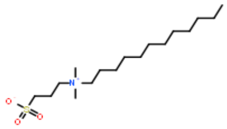 | 0 | 2364 | C15 | 5.5628 | 59 | 15 | -2.4984 | -6.6112 | -23.09 | 6.9257 | 20.227 | 16.782 | -86.21584 | 1.81551 | 1 |
| 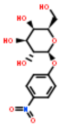 | 0 | 2354 | C12H15NO8 | 5.573 | 36 | 8 | -3.2114 | -3.6949 | -22.25 | 7.4222 | 18.159 | 23.564 | -90.44074 | 1.45791 | 1 |

| | | | | | | | | | | | | | | | |
|---|---|---|---|---|---|---|---|---|---|---|---|---|---|---|---|
| 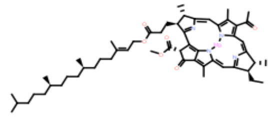 | 0 | 1628 | m | 5.5776 | 140 | 19 | -4.1976 | -11.985 | -46.78 | 0 | 28.919 | 57.452 | -251.0681 | 3.92741 | 1 |
| 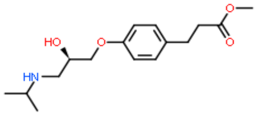 | 0 | 82 | 187 | 5.5967 | 46 | 9 | -3.4033 | -5.5863 | -22.51 | 6.54 | 18.153 | 26.548 | -59.56157 | 1.4692 | 1 |
| 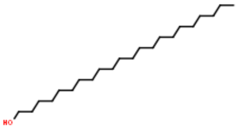 | 0 | 519 | 632 | 5.6183 | 69 | 21 | -3.7822 | -8.4694 | -21.56 | 7.0081 | 17.56 | 18.861 | -121.8994 | 2.06784 | 1 |
| 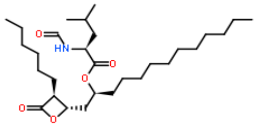 | 0 | 955 | 1083 | 5.6344 | 88 | 23 | -4.943 | -9.1113 | -32.46 | 11.024 | 23.616 | 28.148 | -125.8475 | 2.26759 | 1 |
| 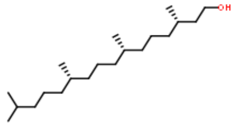 | 0 | 1429 | $C_{20}H_{42}O$ | 5.6486 | 63 | 15 | -3.6057 | -6.9751 | -22.05 | 4.4039 | 18.18 | 24.565 | -125.168 | 2.10234 | 1 |
| 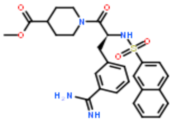 | 0 | 1528 | $C_{27}H_{30}N_4O_5S$ | 5.6812 | 67 | 8 | -4.5481 | -6.7986 | -31.56 | 12.26 | 28.451 | 30.013 | -186.3444 | 2.13618 | 1 |
| 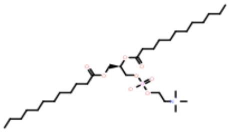 | 0 | 2484 | $C_{32}H_{65}NO_8P$ | 5.6838 | 106 | 30 | -10.257 | -8.9713 | -42.09 | 17.429 | 35.656 | 37.177 | -194.5383 | 3.3626 | 1 |
| 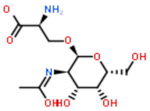 | 0 | 2133 | $C_{11}H_{20}N_2O_8$ | 5.7293 | 40 | 11 | -7.1019 | -3.269 | -19.73 | 5.6532 | 23.623 | 27.13 | -92.47969 | 1.34822 | 1 |
| 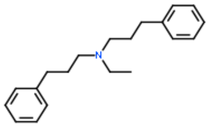 | 0 | 1409 | 1616 | 5.731 | 48 | 9 | 0 | -6.3722 | -27.37 | 6.2642 | 13.374 | 27.55 | -87.65283 | 1.58194 | 1 |
| 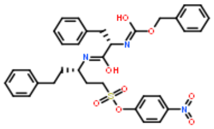 | 0 | 1810 | $C_{34}H_{35}N_3O_8S$ | 5.7707 | 81 | 18 | -1.3529 | -7.5447 | -37.37 | 11.84 | 22.268 | 25.779 | -156.2558 | 2.76124 | 1 |
| 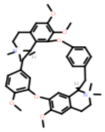 | 0 | 1168 | 1336 | 5.7983 | 96 | 0 | -1.0136 | -10.48 | -28.46 | 5.3233 | 19.533 | 39.402 | -179.3931 | 2.57612 | 1 |

| | | | | | | | | | | | | | | | |
|---|---|---|---|---|---|---|---|---|---|---|---|---|---|---|---|
| 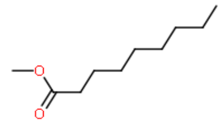 | 0 | 1423 | NON | 5.8161 | 32 | 7 | -0.9178 | -5.2118 | -15.74 | 1.6129 | 12.39 | 19.562 | -87.33479 | 1.48531 | 1 |
| 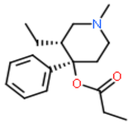 | 0 | 1348 | C17H25NO2 | 5.8189 | 45 | 4 | -0.6552 | -5.9678 | -16.67 | 3.0021 | 12.926 | 21.591 | -112.321 | 1.4598 | 1 |
| 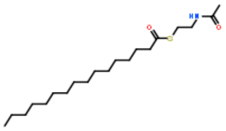 | 0 | 2686 | C20H39NO2S | 5.829 | 63 | 17 | -1.1602 | -8.6884 | -21.82 | 1.9791 | 16.66 | 18.978 | -109.7596 | 2.26917 | 1 |
| 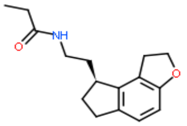 | 0 | 857 | 980 | 5.8471 | 40 | 4 | -0.8358 | -5.3362 | -17.67 | 1.9137 | 15.357 | 21.423 | -103.1939 | 1.49207 | 1 |
| 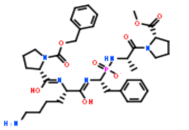 | 0 | 1753 | C36H52N6O9P | 5.8698 | 103 | 21 | -4.166 | -9.4591 | -48.02 | 4.7766 | 30.612 | 49.001 | -169.4126 | 2.49794 | 1 |
| 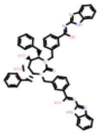 | 0 | 2443 | C49H44N8O5 | 5.883 | 106 | 12 | -4.952 | -9.6517 | -48.78 | 35.624 | 28.892 | 35.472 | -203.4891 | 2.91682 | 1 |
| 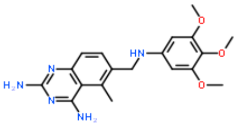 | 0 | 1027 | 1157 | 5.9095 | 50 | 2 | -0.2555 | -7.3793 | -27.12 | 0 | 23.386 | 30.117 | -108.2415 | 1.70293 | 1 |
| 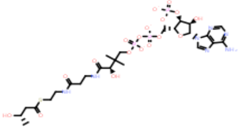 | 0 | 3263 | m | 5.9379 | 92 | 24 | -16.333 | -6.5234 | -41.23 | 16.776 | 44.097 | 53.524 | -192.6056 | 2.84769 | 1 |
| 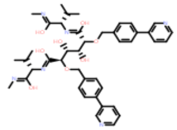 | 0 | 2401 | C42H52N6O8 | 6.0272 | 108 | 23 | -3.1369 | -10.008 | -45.59 | 6.5703 | 33.57 | 35.359 | -193.0874 | 3.09941 | 1 |
| 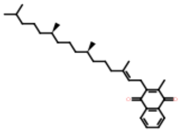 | 0 | 898 | C31H46O2 | 6.0676 | 79 | 14 | -2.9189 | -7.6708 | -32.26 | 8.9215 | 22.917 | 30.166 | -148.2897 | 2.71148 | 1 |
| 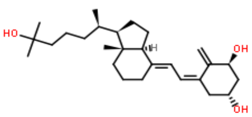 | 0 | 32 | C27H44O3 | 6.0844 | 74 | 8 | -5.0895 | -8.5258 | -18.03 | 11.706 | 21.858 | 21.956 | -149.7335 | 1.93502 | 1 |

| | | | | | | | | | | | | | | | |
|---|---|---|---|---|---|---|---|---|---|---|---|---|---|---|---|
| 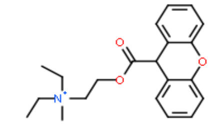 | 0 | 818 | 940 | 6.107 | 51 | 6 | -1.6254 | -6.4735 | -21.19 | 3.7732 | 15.263 | 27.932 | -113.868 | 1.47685 | 1 |
| 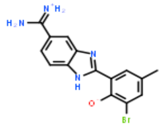 | 0 | 2031 | 334 | 6.1317 | 34 | 0 | -2.5124 | -5.1028 | -21.08 | 1.3623 | 21.394 | 30.493 | -101.4913 | 1.51825 | 1 |
| 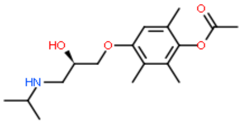 | 0 | 1084 | 1214 | 6.1363 | 49 | 6 | -4.3639 | -6.4417 | -20.02 | 9.6775 | 18.876 | 27.2 | -116.0625 | 1.70151 | 1 |
| 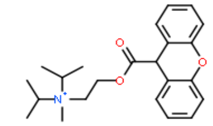 | 0 | 664 | 782 | 6.1416 | 57 | 6 | -1.0696 | -6.4202 | -25.16 | 8.2209 | 17.065 | 25.728 | -109.2744 | 1.88657 | 1 |
| 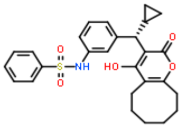 | 0 | 1794 | C27H29NO5S | 6.1669 | 63 | 6 | -0.8245 | -6.6118 | -28.03 | 10.825 | 21.665 | 21.765 | -152.9368 | 2.11264 | 1 |
| 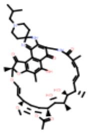 | 0 | 502 | C46H62N4O11 | 6.1856 | 123 | 6 | -0.5792 | -11.333 | -33.84 | 9.0053 | 27.705 | 29.241 | -196.1717 | 2.88048 | 1 |
| 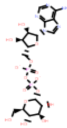 | 0 | 1554 | C16H25N5O15P2 | 6.1956 | 61 | 15 | -11.481 | -4.252 | -31.96 | 1.583 | 38.948 | 46.798 | -158.7306 | 2.05202 | 1 |
| 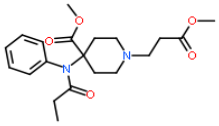 | 0 | 778 | 899 | 6.2352 | 55 | 8 | -2.6838 | -7.1065 | -23.64 | 5.2069 | 20.463 | 27.504 | -142.4656 | 1.80352 | 1 |
| 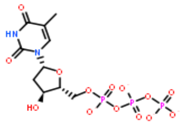 | 0 | 2185 | C10H17N2O14P3 | 6.2726 | 42 | 9 | -14.093 | -2.5934 | -29.13 | 10.499 | 32.031 | 56.177 | -123.128 | 1.67046 | 1 |
| 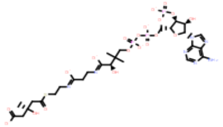 | 0 | 2851 | C27H39N7O20P3S | 6.2981 | 95 | 26 | -22.643 | -5.8254 | -37.78 | 11.883 | 43.446 | 75.693 | -180.0811 | 3.13044 | 1 |
| 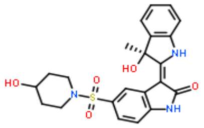 | 0 | 3235 | RYU | 6.2983 | 54 | 4 | -1.5117 | -5.767 | -23.54 | 6.8222 | 19.618 | 24.733 | -138.9449 | 2.04998 | 1 |

| | | | | | | | | | | | | | | | |
|---|---|---|---|---|---|---|---|---|---|---|---|---|---|---|---|
| 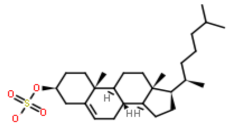 | 0 | 1754 | $C_{27}H_{46}O_4S$ | 6.3101 | 77 | 7 | -1.4649 | -7.1867 | -7.881 | 1.8745 | 15.469 | 8.7663 | -129.4587 | 2.22409 | 1 |
| 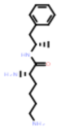 | 0 | 1124 | 1255 | 6.3324 | 44 | 10 | -3.4845 | -4.7861 | -20.8 | 1.6313 | 21.35 | 24.483 | -110.1993 | 1.49863 | 1 |
| 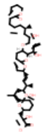 | 0 | 1923 | $C_{44}H_{68}O_{13}$ | 6.3505 | 124 | 14 | -5.6481 | -8.3599 | -28.27 | 17.733 | 26.814 | 23.28 | -154.5852 | 2.8797 | 1 |
| 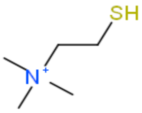 | 0 | 1580 | $C_5H_{14}NS$ | 6.3661 | 21 | 3 | 0 | -3.1347 | -8.02 | 0.3232 | 8.1661 | 12.679 | -29.14142 | 0.62341 | 1 |
| 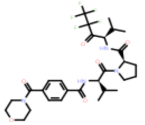 | 0 | 2082 | $C_{29}H_{37}F_5N_4O_6$ | 6.4465 | 81 | 10 | -4.5469 | -9.7826 | -32.57 | 10.901 | 27.239 | 36.521 | -173.2119 | 2.40617 | 1 |
| 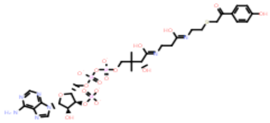 | 0 | 3264 | $C_{29}H_{42}N_7O_{18}P_3S$ | 6.4472 | 96 | 25 | -9.9904 | -6.2815 | -44.38 | 20.01 | 34.884 | 41.126 | -206.4456 | 3.30496 | 1 |
| 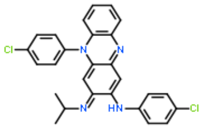 | 0 | 725 | $C_{27}H_{22}Cl_2N_4$ | 6.4567 | 55 | 3 | 0 | -9.1521 | -30.65 | 11.029 | 16.466 | 34.048 | -146.1267 | 2.16218 | 1 |
| 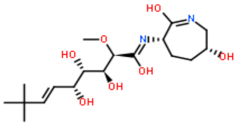 | 0 | 3066 | $C_{18}H_{32}N_2O_7$ | 6.464 | 59 | 14 | -4.9189 | -5.283 | -21.74 | 0 | 26.72 | 23.854 | -131.6803 | 1.74089 | 1 |
| 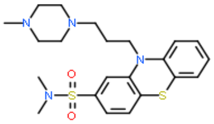 | 0 | 1414 | 1622 | 6.4701 | 60 | 6 | -2.6684 | -6.3846 | -29.32 | 13.983 | 16.382 | 34.148 | -131.6386 | 1.86625 | 1 |
| 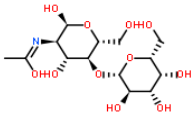 | 0 | 3269 | $C_{14}H_{25}NO_{11}$ | 6.4912 | 51 | 13 | -7.6013 | -3.8261 | -18.78 | 4.0581 | 29.644 | 22.118 | -53.43269 | 1.56117 | 1 |
| 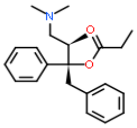 | 0 | 534 | $C_{22}H_{29}NO_2$ | 6.5879 | 54 | 8 | -1.8375 | -6.7823 | -19.82 | 6.6614 | 16.467 | 21.897 | -112.7286 | 1.66827 | 1 |

| | | | | | | | | | | | | | | | |
|---|---|---|---|---|---|---|---|---|---|---|---|---|---|---|---|
| 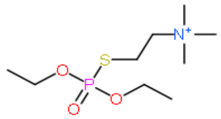 | 0 | 929 | 1057 | 6.6312 | 38 | 8 | -1.2768 | -5.5316 | -17.05 | 3.2273 | 14.196 | 20.054 | -75.97076 | 1.2817 | 1 |
| 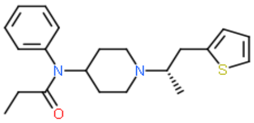 | 0 | 1273 | 1470 | 6.7149 | 53 | 7 | -1.4724 | -7.5741 | -25.1 | 6.8623 | 16.685 | 29.983 | -136.7552 | 1.83491 | 1 |
| 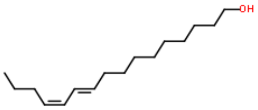 | 0 | 2678 | C16H30O | 6.7401 | 47 | 12 | -2.4952 | -6.5361 | -20.98 | 3.3078 | 18.146 | 23.658 | -93.64185 | 1.84363 | 1 |
| 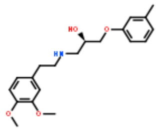 | 0 | 1147 | 1295 | 6.7915 | 52 | 8 | -0.9437 | -6.1188 | -24.73 | 7.4513 | 15.448 | 25.993 | -97.34191 | 1.69841 | 1 |
| 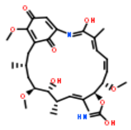 | 0 | 2160 | C29H40N2O9 | 6.8198 | 80 | 6 | -1.6991 | -7.1246 | -27.25 | 0 | 29.029 | 26.923 | -144.31 | 2.12032 | 1 |
| 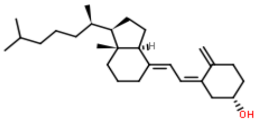 | 0 | 64 | C27H44O | 6.8654 | 72 | 6 | -3.1393 | -8.716 | -17.32 | 10.64 | 18.616 | 21.244 | -140.3978 | 1.95894 | 1 |
| 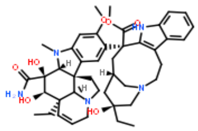 | 0 | 203 | C43H55N5O7 | 6.8874 | 110 | 8 | -4.0407 | -8.992 | -30.28 | 16.476 | 26.934 | 28.074 | -155.2076 | 2.49022 | 1 |
| 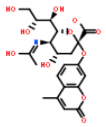 | 0 | 1550 | C21H25NO11 | 6.9715 | 57 | 11 | -9.1213 | -5.3172 | -15.35 | 7.7039 | 27.911 | 25.457 | -153.0483 | 2.1472 | 1 |
| 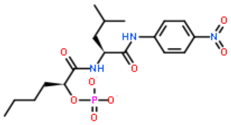 | 0 | 2020 | MLN | 6.9731 | 56 | 12 | -8.1275 | -6.2565 | -30.41 | 1.6533 | 21.766 | 57.006 | -148.0117 | 2.03101 | 1 |
| 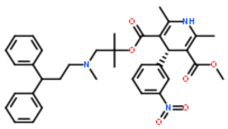 | 0 | 415 | 528 | 7.028 | 86 | 10 | -1.2293 | -8.9843 | -38.39 | 16.19 | 22.4 | 33.378 | -169.1029 | 2.27671 | 1 |
| 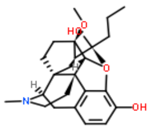 | 0 | 1254 | 1450 | 7.0496 | 65 | 5 | -1.5262 | -6.783 | -19.6 | 2.4945 | 18.164 | 25.659 | -122.2216 | 1.56473 | 1 |

| | | | | | | | | | | | | | | | |
|---|---|---|---|---|---|---|---|---|---|---|---|---|---|---|---|
| 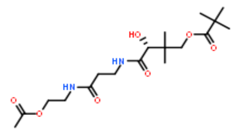 | 0 | 2736 | C18H32N2O7 | 7.0628 | 59 | 12 | -4.2171 | -6.3249 | -29.25 | 11.671 | 21.937 | 31.165 | -144.5595 | 2.00805 | 1 |
| 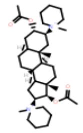 | 0 | 1169 | C35H60N2O4 | 7.0678 | 101 | 4 | -1.8113 | -8.481 | -25.58 | 7.5105 | 17.13 | 35.088 | -165.5356 | 2.31088 | 1 |
| 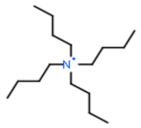 | 0 | 1626 | TBA | 7.091 | 53 | 12 | 0 | -6.4307 | -20.42 | 1.8338 | 11.5 | 22.566 | -55.72761 | 1.15307 | 1 |
| 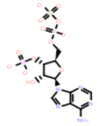 | 0 | 2602 | C10H15N5O13P2S | 7.2648 | 42 | 9 | -12.67 | -3.416 | -8.68 | 0 | 28.298 | 35.856 | -70.80907 | 1.42274 | 1 |
| 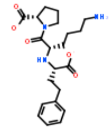 | 0 | 606 | 722 | 7.3604 | 58 | 14 | -11.192 | -5.1609 | -18.12 | 8.7886 | 27.17 | 35.15 | -123.2702 | 1.60639 | 1 |
| 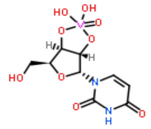 | 0 | 3175 | C9H12N2O9V | 7.3627 | 33 | 5 | -6.657 | -2.3578 | -18.21 | 0 | 26.238 | 32.304 | -94.52556 | 1.6338 | 1 |
| 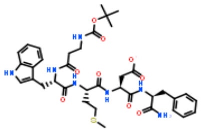 | 0 | 78 | C37H49N7O9S | 7.3727 | 102 | 22 | -5.6685 | -9.1454 | -45.64 | 19.916 | 34.104 | 34.469 | -205.7647 | 3.2954 | 1 |
| 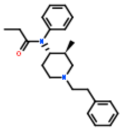 | 0 | 1367 | 1571 | 7.3979 | 56 | 7 | -1.038 | -7.7362 | -26.94 | 5.5545 | 17.053 | 33.073 | -139.5502 | 1.97846 | 1 |
| 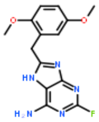 | 0 | 2780 | PU2 | 7.405 | 36 | 2 | -0.9123 | -5.6948 | -23.24 | 1.3652 | 22.002 | 27.652 | -116.6598 | 1.37957 | 1 |
| 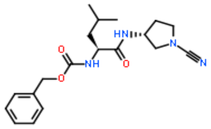 | 0 | 3123 | NBL | 7.4115 | 52 | 8 | -0.8892 | -6.8814 | -29.09 | 5.8332 | 20.255 | 30.108 | -96.86832 | 1.75398 | 1 |
| 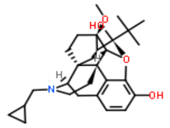 | 0 | 800 | 644073 | 7.419 | 75 | 6 | -1.5109 | -7.3396 | -19.89 | 5.7574 | 17.849 | 23.647 | -108.3655 | 1.58598 | 1 |

| | | | | | | | | | | | | | | | |
|---|---|---|---|---|---|---|---|---|---|---|---|---|---|---|---|
| 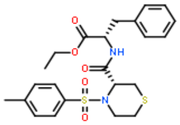 | 0 | 1498 | SUB | 7.4865 | 60 | 8 | -1.5248 | -7.0552 | -26.2 | 10.464 | 19.358 | 25.263 | -140.8096 | 2.05325 | 1 |
| 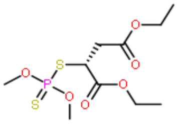 | 0 | 655 | 772 | 7.5071 | 38 | 9 | -0.9435 | -4.9587 | -24.55 | 3.4614 | 18.29 | 25.11 | -100.7447 | 1.53339 | 1 |
| 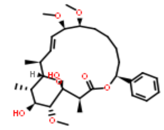 | 0 | 2562 | C29H44O8 | 7.616 | 81 | 6 | -0.9396 | -8.2211 | -25.01 | 13.207 | 19.675 | 21.56 | -140.0262 | 1.86232 | 1 |
| 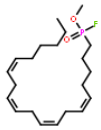 | 0 | 2198 | C21H36FO2P | 7.6323 | 61 | 16 | -1.3623 | -7.1426 | -29.91 | 12.671 | 15.33 | 25.877 | -137.2074 | 1.66262 | 1 |
| 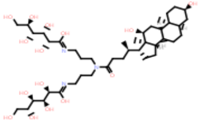 | 0 | 1662 | C42H75N3O15 | 7.7044 | 135 | 37 | -16.929 | -9.4747 | -38.85 | 24.538 | 46.941 | 35.772 | -204.3488 | 3.64202 | 1 |
| 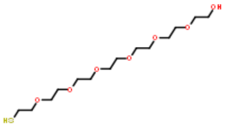 | 0 | 2140 | PE7 | 7.7107 | 52 | 21 | -5.7293 | -6.1827 | -16.4 | 7.9953 | 14.827 | 21.566 | -100.3832 | 1.85541 | 1 |
| 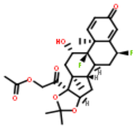 | 0 | 921 | 1047 | 7.7701 | 67 | 4 | -1.0085 | -6.9339 | -20.94 | 3.551 | 22.606 | 21.86 | -138.9077 | 1.84834 | 1 |
| 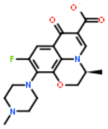 | 0 | 1035 | 1165 | 7.7962 | 45 | 1 | -4.0651 | -4.857 | 0.7475 | 3.2906 | 14.688 | 10.602 | -26.86196 | 1.45882 | 1 |
| 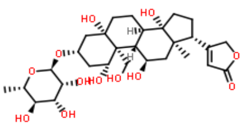 | 0 | 964 | 1092 | 7.8053 | 85 | 12 | -10.997 | -6.0154 | -20.45 | 15.278 | 29.475 | 32.777 | -192.5441 | 2.17988 | 1 |
| 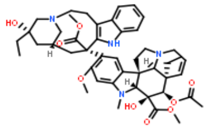 | 0 | 457 | C46H58N4O9 | 7.8149 | 117 | 8 | -1.8367 | -10.556 | -26.68 | 11.833 | 26.772 | 21.567 | -187.9821 | 2.52457 | 1 |
| 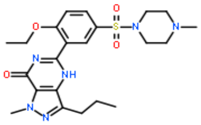 | 0 | 98 | 5212 | 7.8641 | 63 | 5 | -0.0526 | -6.9763 | -23.67 | 7.2323 | 21.198 | 19.85 | -123.9545 | 2.05955 | 1 |

| | | | | | | | | | | | | | | | |
|---|---|---|---|---|---|---|---|---|---|---|---|---|---|---|---|
| 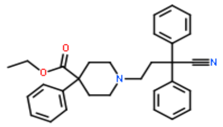 | 0 | 953 | 1081 | 7.8773 | 66 | 8 | -2.6632 | -7.9756 | -27.47 | 12.41 | 19.469 | 31.187 | -146.4777 | 1.91998 | 1 |
| 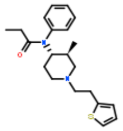 | 0 | 1245 | 1439 | 7.8996 | 53 | 7 | -1.1813 | -7.2616 | -23.93 | 6.0519 | 17.549 | 28.325 | -133.1785 | 1.94846 | 1 |
| 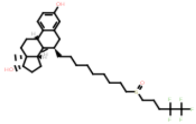 | 0 | 825 | C32H47F5O3S | 8.07 | 88 | 15 | -4.5398 | -7.9379 | -33.28 | 13.399 | 24.282 | 34.694 | -147.339 | 2.36017 | 1 |
| 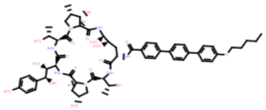 | 0 | 255 | C58H73N7O17 | 8.1562 | 155 | 18 | -7.677 | -11.354 | -44.9 | 21.252 | 43.325 | 36.423 | -216.0223 | 3.68538 | 1 |
| 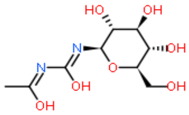 | 0 | 2899 | C9H16N2O7 | 8.271 | 34 | 8 | -7.1701 | -2.6836 | -12.47 | 5.6097 | 23.428 | 22.641 | -94.84229 | 1.49549 | 1 |
| 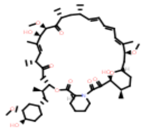 | 0 | 757 | C51H79NO13 | 8.2999 | 144 | 9 | -4.1277 | -10.98 | -46.82 | 21.051 | 38.183 | 39.776 | -256.7401 | 3.69805 | 1 |
| 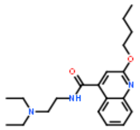 | 0 | 414 | 527 | 8.3415 | 54 | 8 | -0.6024 | -7.2856 | -26.23 | 7.1943 | 17.697 | 28.157 | -122.6535 | 1.66876 | 1 |
| 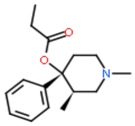 | 0 | 1276 | C16H23NO2 | 8.36 | 42 | 3 | -0.7761 | -4.9709 | -9.838 | 0.903 | 11.204 | 19.315 | -102.2106 | 1.40051 | 1 |
| 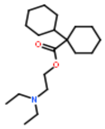 | 0 | 685 | 804 | 8.3746 | 57 | 7 | 0 | -6.0591 | -19.86 | 3.4346 | 14.876 | 22.547 | -102.7611 | 1.58015 | 1 |
| 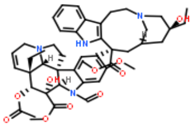 | 0 | 428 | C46H56N4O10 | 8.3964 | 116 | 9 | -1.9809 | -9.8732 | -31.36 | 14.781 | 26.365 | 26.272 | -209.5609 | 2.65575 | 1 |
| 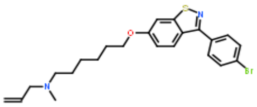 | 0 | 2272 | C23H27BrN2OS | 8.4194 | 55 | 10 | -2.5858 | -7.7275 | -25.4 | 7.7206 | 19.787 | 30.404 | -118.8165 | 2.05171 | 1 |

| | | | | | | | | | | | | | | | |
|---|---|---|---|---|---|---|---|---|---|---|---|---|---|---|---|
| 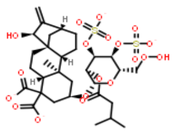 | 0 | 2162 | C31H46O19S2 | 8.5496 | 94 | 15 | -11.037 | -5.7314 | -32.9 | 6.5402 | 35.066 | 51.501 | -150.4382 | 2.38902 | 1 |
| 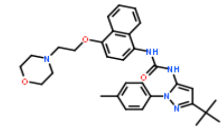 | 0 | 2735 | C31H37N5O3 | 8.5694 | 76 | 6 | -1.8369 | -9.9454 | -32.95 | 14.729 | 22.111 | 36.081 | -190.3457 | 2.58617 | 1 |
| 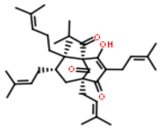 | 0 | 1664 | C35H52O4 | 8.6 | 91 | 11 | -0.7241 | -9.9372 | -30.72 | 10.866 | 20.819 | 28.897 | -135.561 | 2.03964 | 1 |
| 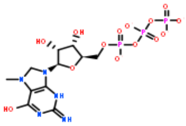 | 0 | 3032 | C11H20N5O14P3 | 8.6479 | 49 | 11 | -14.902 | -3.3375 | -31 | 2.9958 | 34.886 | 68.212 | -106.3793 | 1.61547 | 1 |
| 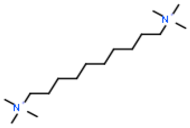 | 0 | 1114 | 1245 | 8.7454 | 56 | 11 | 0 | -7.5722 | -20.2 | 5.0334 | 13.407 | 21.733 | -85.11769 | 1.67276 | 1 |
| 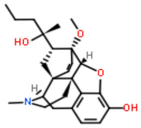 | 0 | 1299 | C25H33NO4 | 8.7925 | 63 | 5 | -1.2921 | -6.8414 | -19.84 | 2.5201 | 17.21 | 28.889 | -123.3567 | 1.56114 | 1 |
| 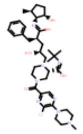 | 0 | 2491 | C37H55ClN8O5 | 8.8178 | 106 | 15 | -1.1725 | -9.6303 | -42.83 | 10.339 | 30.765 | 34.534 | -210.9535 | 3.60479 | 1 |
| 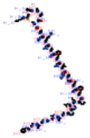 | 0 | 1138 | C171H267N51O53S2 | 8.926 | 544 | 123 | -19.361 | -21.18 | -127.4 | 63.051 | 58.624 | 66.446 | -453.6639 | 9.74877 | 1 |
| 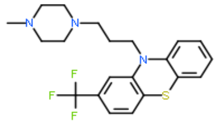 | 0 | 712 | 831 | 8.9514 | 52 | 5 | -1.2554 | -7.0163 | -23.62 | 7.249 | 15.031 | 33.075 | -115.9374 | 1.68098 | 1 |
| 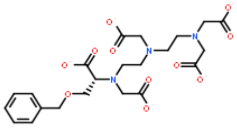 | 0 | 626 | C22H28GdN3O11 | 9.0231 | 62 | 20 | -11.838 | -4.7673 | -23.49 | 2.9758 | 29.401 | 45.178 | -146.4926 | 2.10144 | 1 |
| 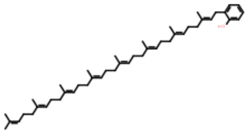 | 0 | 2913 | C46H70O | 9.0347 | 117 | 23 | -0.9672 | -11.532 | -45.86 | 17.848 | 24.849 | 33.217 | -169.0889 | 3.21468 | 1 |

| | | | | | | | | | | | | | | | |
|---|---|---|---|---|---|---|---|---|---|---|---|---|---|---|---|
| 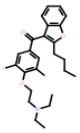 | 0 | 989 | C25H29I2NO3 | 9.0354 | 60 | 8 | -2.1083 | -7.962 | -24.94 | 9.8844 | 18.761 | 30.059 | -124.5209 | 2.34986 | 1 |
| 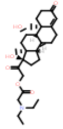 | 0 | 652 | C27H41NO6 | 9.1277 | 75 | 9 | -4.3709 | -7.6725 | -21.89 | 5.4432 | 22.448 | 32.898 | -174.436 | 2.1179 | 1 |
| 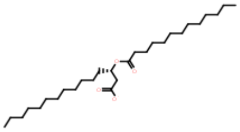 | 0 | 1591 | C28H54O4 | 9.2085 | 85 | 25 | -4.6677 | -7.0752 | -29.69 | 11.103 | 23.335 | 24.671 | -160.0389 | 2.53634 | 1 |
| 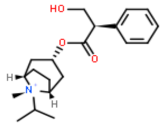 | 0 | 225 | C20H30NO3 | 9.2899 | 54 | 6 | -0.6295 | -6.2357 | -21.31 | 4.8305 | 18.285 | 24.482 | -98.2546 | 1.58251 | 1 |
| 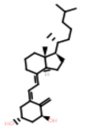 | 0 | 1242 | 1436 | 9.3241 | 73 | 7 | -1.5811 | -7.5318 | -20.93 | 5.4327 | 20.768 | 24.649 | -131.7041 | 2.14882 | 1 |
| 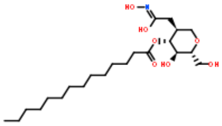 | 0 | 1755 | C22H41NO7 | 9.3468 | 71 | 20 | -7.1468 | -6.5272 | -29.5 | 16.114 | 28.808 | 27.314 | -84.95921 | 2.45986 | 1 |
| 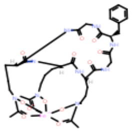 | 0 | 2349 | C34H48FeN9O12 | 9.3612 | 104 | 2 | -1.1445 | -8.7934 | -31.35 | 0 | 21.212 | 49.072 | -150.998 | 2.21158 | 1 |
| 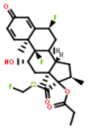 | 0 | 475 | m | 9.3733 | 65 | 5 | -0.9637 | -7.3992 | -20.69 | 4.8294 | 20.247 | 24.817 | -120.5802 | 1.76156 | 1 |
| 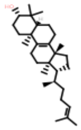 | 0 | 3339 | C30H50O | 9.3736 | 81 | 5 | 0 | -8.2105 | -17.75 | 2.5679 | 18.855 | 21.118 | -169.8812 | 2.20324 | 1 |
| 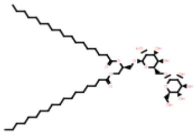 | 0 | 3094 | C51H96O15 | 9.5915 | 162 | 49 | -12.322 | -11.004 | -58.6 | 37.553 | 38.173 | 39.671 | -219.2667 | 4.70011 | 1 |
| 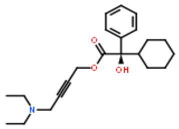 | 0 | 934 | 1062 | 9.6016 | 57 | 9 | -0.7268 | -7.2336 | -22.23 | 6.4936 | 19.15 | 22.515 | -108.3193 | 1.74072 | 1 |

| | | | | | | | | | | | | | | | |
|---|---|---|---|---|---|---|---|---|---|---|---|---|---|---|---|
| 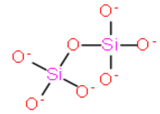 | 0 | 1371 | H4Al2O9Si2 | 9.624 | 9 | 2 | -13.396 | -0.483 | -4.757 | 0 | 15.595 | 54.09 | -20.3004 | 0.41222 | 1 |
| 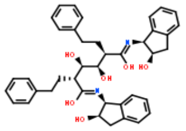 | 0 | 2420 | C40H44N2O6 | 9.7292 | 92 | 19 | -1.325 | -9.0158 | -29.28 | 4.6332 | 22.04 | 26.801 | -194.3059 | 2.3849 | 1 |
| 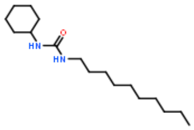 | 0 | 3323 | CDU | 9.7728 | 54 | 12 | -1.8323 | -7.2852 | -17.3 | 3.0203 | 15.823 | 23.801 | -101.6661 | 1.48382 | 1 |
| 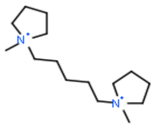 | 0 | 962 | 1090 | 9.8717 | 49 | 6 | 0 | -6.6833 | -18.38 | 1.9832 | 13.16 | 27.385 | -100.3412 | 1.73449 | 1 |
| 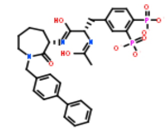 | 0 | 2077 | C30H35N3O9P2 | 9.8928 | 75 | 12 | -13.656 | -6.4333 | -34.24 | 5.3307 | 30.941 | 74.828 | -178.7254 | 2.17704 | 1 |
| 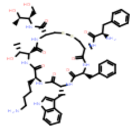 | 0 | 9 | C49H66N10O10S2 | 9.9228 | 137 | 22 | -8.1505 | -11.494 | -56.31 | 20.945 | 45.209 | 52.851 | -242.6591 | 3.78906 | 1 |
| 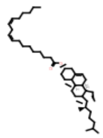 | 0 | 1850 | C45H76O2 | 9.9672 | 123 | 20 | -1.1302 | -11.603 | -36.32 | 12.952 | 21.666 | 31.672 | -227.1712 | 2.80189 | 1 |
| 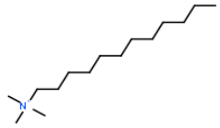 | 0 | 2486 | CAT | 10.008 | 50 | 11 | 0 | -7.0238 | -19.07 | 1.2646 | 14.233 | 23.858 | -87.83913 | 1.91585 | 1 |
| 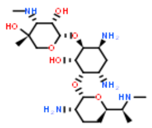 | 0 | 679 | 798 | 10.037 | 76 | 13 | -10.274 | -5.9936 | -21.01 | 17.868 | 29.941 | 30.425 | -128.1208 | 1.76799 | 1 |
| 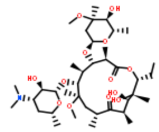 | 0 | 1081 | m | 10.086 | 121 | 12 | -2.5481 | -9.6519 | -28.66 | 20.123 | 20.945 | 25.271 | -138.068 | 2.65509 | 1 |
| Ca | 0 | 1194 | Ca | 10.165 | 1 | 0 | -5.244 | 0.12086 | -1.57 | 0 | 1.9805 | 35.51 | 17.125147 | -0.1226 | 1 |

| | | | | | | | | | | | | | | | |
|---|---|---|---|---|---|---|---|---|---|---|---|---|---|---|---|
| 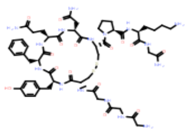 | 0 | 2359 | 2638 | 10.293 | 159 | 28 | -12.828 | -8.5108 | -64.1 | 35.185 | 61.939 | 42.708 | -227.2116 | 4.32673 | 1 |
| 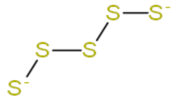 | 0 | 2652 | S5 | 10.303 | 5 | 2 | 0 | -0.5877 | -8.054 | 3.1934 | 9.0987 | 13.349 | -1.299347 | 0.36921 | 1 |
| 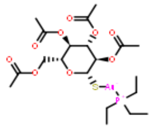 | 0 | 871 | C20H34AuO9PS | 10.388 | 66 | 11 | -4.895 | -7.0689 | -23.48 | 9.8831 | 23.19 | 31.923 | -107.9671 | 2.17285 | 1 |
| 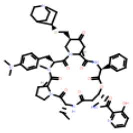 | 0 | 1192 | C53H67N9O10S | 10.395 | 140 | 8 | -2.1298 | -12.338 | -46.51 | 20.527 | 32.53 | 44.002 | -219.731 | 3.55478 | 1 |
| 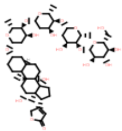 | 0 | 950 | m | 10.404 | 140 | 19 | -14.592 | -11.148 | -37.97 | 35.917 | 43.389 | 41.316 | -260.1649 | 3.73202 | 1 |
| 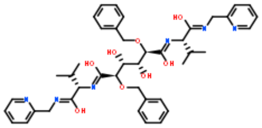 | 0 | 3440 | C42H52N6O8 | 10.487 | 108 | 27 | -4.0458 | -8.1082 | -44.32 | 9.119 | 35.05 | 34.124 | -130.8961 | 3.36434 | 1 |
| 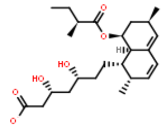 | 0 | 3423 | LVA | 10.517 | 67 | 12 | -3.428 | -6.7869 | -24.01 | 5.2408 | 22.7 | 30.876 | -145.8828 | 1.86649 | 1 |
| 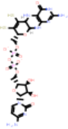 | 0 | 2977 | C19H26N8O13P2S2 | 10.704 | 68 | 13 | -17.984 | -3.584 | -14.96 | 10.907 | 36.826 | 47.882 | -150.131 | 2.33157 | 1 |
| 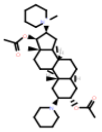 | 0 | 1171 | m | 10.795 | 97 | 4 | -1.08 | -8.2915 | -22.23 | 13.474 | 16.962 | 27.436 | -143.7312 | 2.33673 | 1 |
| 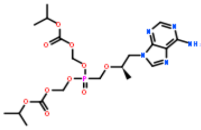 | 0 | 194 | C19H30N5O10P | 10.901 | 65 | 13 | -3.7544 | -7.0007 | -28.09 | 10.041 | 27.448 | 28.439 | -130.666 | 1.76292 | 1 |
| 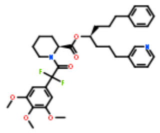 | 0 | 2588 | C35H42F2N2O6 | 10.904 | 87 | 13 | -2.5933 | -9.4047 | -33.99 | 13.085 | 25.365 | 34.97 | -187.8892 | 2.26809 | 1 |

| | | | | | | | | | | | | | | | |
|---|---|---|---|---|---|---|---|---|---|---|---|---|---|---|---|
| 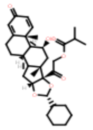 | 0 | 1218 | C32H44O7 | 11.152 | 83 | 6 | -2.0087 | -7.687 | -21.1 | 10.104 | 24.331 | 22.083 | -143.64 | 2.14037 | 1 |
| 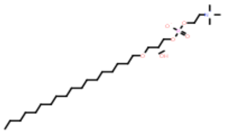 | 0 | 3282 | C26H57NO6P | 11.371 | 90 | 27 | -9.4269 | -7.7664 | -30.16 | 7.6739 | 25.467 | 46.488 | -146.6005 | 3.16019 | 1 |
| 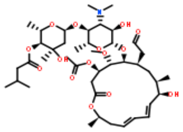 | 0 | 1156 | C42H69NO15 | 11.605 | 127 | 15 | -4.5658 | -10.197 | -36.78 | 26.028 | 31.36 | 28.223 | -230.6666 | 2.83925 | 1 |
| 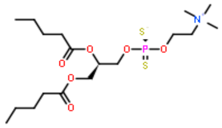 | 0 | 1973 | C18H36NO6PS2 | 11.635 | 64 | 16 | -1.1687 | -6.376 | -27.57 | 9.0481 | 19.242 | 25.723 | -130.0582 | 2.02737 | 1 |
| 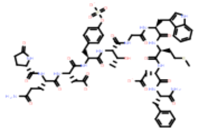 | 0 | 294 | C58H73N13O21S2 | 11.635 | 164 | 38 | -13.112 | -9.915 | -57.85 | 29.568 | 49.777 | 47.623 | -231.607 | 4.588 | 1 |
| 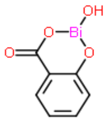 | 0 | 1146 | C7H5BiO4 | 11.718 | 17 | 1 | -2.7529 | -1.6895 | -14.99 | 0.5694 | 11.307 | 38.458 | -39.37071 | 0.58031 | 1 |
| 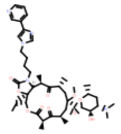 | 0 | 853 | C43H65N5O10 | 11.721 | 123 | 11 | -2.4557 | -10.968 | -28.34 | 10.761 | 30.883 | 26.368 | -193.8135 | 3.4947 | 1 |
| 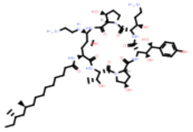 | 0 | 407 | C52H88N10O15 | 11.761 | 165 | 32 | -17.135 | -12.332 | -31.36 | 24.109 | 51.355 | 34.178 | -206.6559 | 4.72984 | 1 |
| 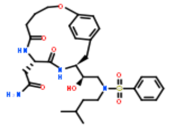 | 0 | 2147 | C29H40N4O7S | 11.773 | 81 | 11 | -2.3549 | -8.0186 | -27.87 | 10.978 | 26.515 | 27.126 | -178.2427 | 2.35681 | 1 |
| 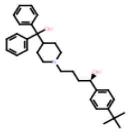 | 0 | 235 | m | 11.789 | 76 | 11 | -1.9163 | -8.2719 | -29.03 | 14.851 | 22.261 | 28.798 | -166.3734 | 2.3267 | 1 |
| 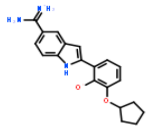 | 0 | 2855 | CR3 | 11.832 | 46 | 1 | -2.372 | -5.8121 | -15.6 | 4.1086 | 18.718 | 30.641 | -104.1043 | 1.84648 | 1 |

| | | | | | | | | | | | | | | | |
|---|---|---|---|---|---|---|---|---|---|---|---|---|---|---|---|
| 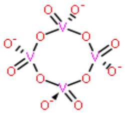 | 0 | 1904 | O12V4 | 12.09 | 16 | 0 | -4.7923 | -0.813 | -11.2 | 0 | 20.423 | 31.278 | 6.605525 | 0.83542 | 1 |
| 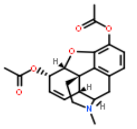 | 0 | 1256 | C21H23NO5 | 12.143 | 50 | 1 | -0.9918 | -6.1313 | -16.64 | 6.8388 | 17.87 | 26.202 | -118.7002 | 1.69885 | 1 |
| 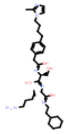 | 0 | 2209 | C33H52N6O4 | 12.307 | 95 | 24 | -8.0897 | -7.9495 | -36.13 | 9.4547 | 31.678 | 46.06 | -175.2381 | 2.3217 | 1 |
| 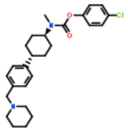 | 0 | 3388 | C26H33ClN2O2 | 12.417 | 64 | 6 | -1.0373 | -8.5894 | -21.25 | 6.081 | 19.177 | 30.934 | -136.7852 | 2.25087 | 1 |
| 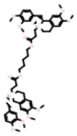 | 0 | 615 | C65H82N2O18S2 | 12.47 | 139 | 16 | -2.5165 | -10.8 | -42.36 | 23.117 | 31.902 | 31.877 | -258.529 | 4.05785 | 1 |
| 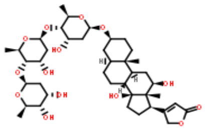 | 0 | 281 | C41H64O14 | 12.54 | 119 | 13 | -6.9667 | -10.806 | -34.53 | 19.116 | 35.043 | 39.977 | -224.4372 | 3.05155 | 1 |
| 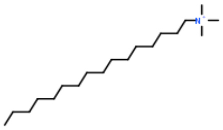 | 0 | 1504 | 16A | 12.584 | 62 | 15 | 0 | -8.6286 | -19.58 | 4.2533 | 12.053 | 26.292 | -107.5956 | 1.55294 | 1 |
| 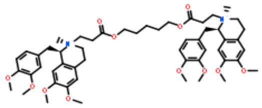 | 0 | 452 | C53H72N2O12 | 12.729 | 139 | 16 | -3.5649 | -10.419 | -53.46 | 32.455 | 38.393 | 36.849 | -155.2677 | 4.04525 | 1 |
| 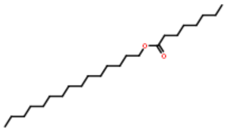 | 0 | 2161 | C23H46O2 | 12.828 | 71 | 20 | -1.876 | -8.2743 | -26.32 | 8.6503 | 17.802 | 28.628 | -138.01 | 2.06972 | 1 |
| 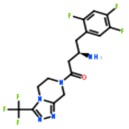 | 0 | 1128 | 1261 | 12.9 | 43 | 7 | -2.9453 | -5.529 | -21.22 | 5.427 | 22.636 | 31.58 | -113.1887 | 1.68382 | 1 |
| 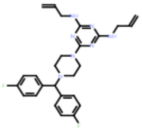 | 0 | 1237 | 1430 | 12.966 | 64 | 7 | -1.1352 | -8.8792 | -33.47 | 16.52 | 23.625 | 35.492 | -138.8281 | 2.13598 | 1 |

| | | | | | | | | | | | | | | | |
|---|---|---|---|---|---|---|---|---|---|---|---|---|---|---|---|
| 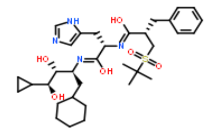 | 0 | 107 | C33H50N4O6S | 13.047 | 94 | 20 | -1.9382 | -9.8173 | -32.62 | 4.9009 | 26.451 | 33.899 | -145.7026 | 2.34234 | 1 |
| 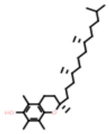 | 0 | 59 | 163 | 13.05 | 81 | 12 | -1.3591 | -8.3963 | -23.57 | 7.6257 | 18.881 | 29.924 | -152.9949 | 2.21335 | 1 |
| 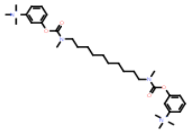 | 0 | 822 | C32H52N4O4 | 13.116 | 92 | 15 | -1.2374 | -8.7115 | -33.76 | 16.672 | 21.834 | 31.009 | -161.1172 | 2.53105 | 1 |
| 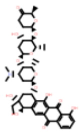 | 0 | 2900 | C40H51NO14 | 13.226 | 106 | 11 | -4.6024 | -9.1314 | -33.88 | 9.9337 | 35.537 | 38.877 | -196.9091 | 3.3238 | 1 |
| 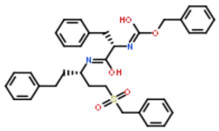 | 0 | 1952 | C35H38N2O5S | 13.556 | 81 | 17 | -0.5261 | -8.3326 | -40.05 | 23.28 | 23.546 | 28.323 | -190.4736 | 2.51147 | 1 |
| Al | 0 | 1193 | 1370 | 13.79 | 4 | 0 | 0 | -0.0254 | -1.142 | 0.0251 | 1.7044 | 20.691 | 0 | 0.08559 | 1 |
| 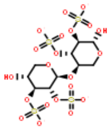 | 0 | 572 | 686 | 13.865 | 49 | 12 | -14.907 | -3.08 | -9.605 | 1.8389 | 36.249 | 42.057 | -97.58043 | 1.66788 | 1 |
| 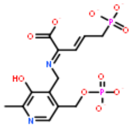 | 0 | 2070 | C13H18N2O10P2 | 13.937 | 40 | 8 | -10.713 | -3.7794 | -21.3 | 0 | 26.28 | 61.159 | -121.9173 | 1.63858 | 1 |
| 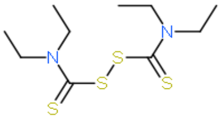 | 0 | 703 | 822 | 14.063 | 36 | 7 | 0 | -4.4943 | -20.06 | 14.805 | 14.91 | 19.496 | -62.12032 | 1.59411 | 1 |
| 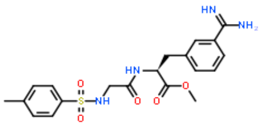 | 0 | 1521 | ANH | 14.067 | 54 | 8 | -4.0211 | -5.0433 | -28.08 | 15.557 | 22.422 | 37.492 | -126.1562 | 1.73947 | 1 |
| 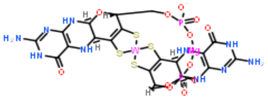 | 0 | 1572 | C20H20MgN10O12P2S4W | 14.102 | 70 | 0 | -5.9681 | -4.2816 | -31.01 | 0 | 42.281 | 47.532 | -90.35935 | 2.44813 | 1 |

| | | | | | | | | | | | | | | | |
|---|---|---|---|---|---|---|---|---|---|---|---|---|---|---|---|
| 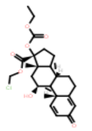 | 0 | 753 | C24H31ClO7 | 14.118 | 63 | 5 | -2.15 | -7.6355 | -18.81 | 13.27 | 21.42 | 24.94 | -151.4052 | 1.89926 | 1 |
| 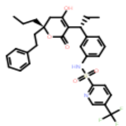 | 0 | 810 | C31H33F3N2O5S | 14.192 | 75 | 11 | -1.5406 | -7.9656 | -27.74 | 13.897 | 23.605 | 28.071 | -183.1009 | 2.11778 | 1 |
| 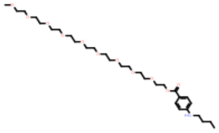 | 0 | 748 | C30H53NO11 | 14.44 | 95 | 30 | -7.331 | -10.415 | -39.73 | 20.488 | 30.759 | 39.906 | -139.8236 | 3.062 | 1 |
| 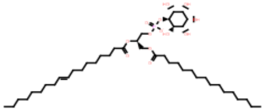 | 0 | 1901 | C43H80O13P | 14.526 | 137 | 42 | -8.4739 | -11.103 | -46.61 | 25.735 | 35.773 | 34.16 | -208.6702 | 3.49225 | 1 |
| 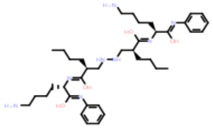 | 0 | 3297 | C38H62N8O4 | 14.644 | 112 | 33 | -7.4681 | -7.6247 | -44.82 | 10.682 | 40.995 | 40.175 | -164.8451 | 3.51173 | 1 |
| 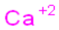 | 0 | 1034 | Cl2Ca | 14.822 | 1 | 0 | -2.6806 | 0.12086 | -1.222 | 0 | 1.9805 | 32.213 | 8.288088 | -0.1226 | 1 |
| 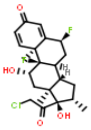 | 0 | 483 | C22H27ClF2O4 | 14.853 | 56 | 4 | -1.7348 | -6.0734 | -16.17 | 6.5368 | 18.968 | 28.686 | -130.7007 | 1.90386 | 1 |
| 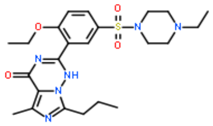 | 0 | 742 | 862 | 14.865 | 66 | 6 | -2.448 | -7.5186 | -25.53 | 18.65 | 23.596 | 29.005 | -144.9876 | 2.22642 | 1 |
| 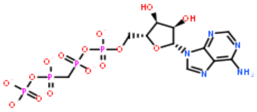 | 0 | 3395 | C11H19N5O15P4 | 14.901 | 49 | 12 | -16.354 | -3.8687 | -22.12 | 0 | 31.489 | 76.024 | -151.9795 | 1.87388 | 1 |
| 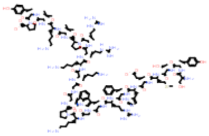 | 0 | 1142 | C136H210N40O31S | 14.95 | 416 | 106 | -21.421 | -18.512 | -90.75 | 51.205 | 65.45 | 43.762 | -479.6002 | 8.41215 | 1 |
| 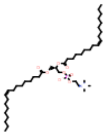 | 0 | 3333 | C44H85NO8P | 15.011 | 138 | 40 | -10.962 | -10.229 | -44.77 | 23.142 | 34.287 | 46.621 | -246.0628 | 3.50539 | 1 |

| | | | | | | | | | | | | | | | |
|---|---|---|---|---|---|---|---|---|---|---|---|---|---|---|---|
| 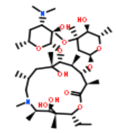 | 0 | 102 | C38H72N2O12 | 15.013 | 124 | 12 | -3.0376 | -10.927 | -33.01 | 24.286 | 26.785 | 32.067 | -209.8844 | 2.74029 | 1 |
| 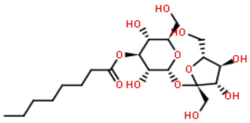 | 0 | 2086 | C20H36O12 | 15.213 | 68 | 19 | -5.6799 | -5.5152 | -26.45 | 13.693 | 28.485 | 28.409 | -122.2367 | 1.9631 | 1 |
| 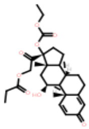 | 0 | 1001 | C27H36O8 | 15.314 | 71 | 7 | -1.454 | -7.6176 | -20.07 | 3.6326 | 25.313 | 28.495 | -152.2197 | 2.04961 | 1 |
| 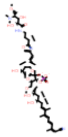 | 0 | 2563 | C50H81N4O15P | 15.489 | 149 | 27 | -8.1906 | -12.012 | -41.68 | 20.45 | 28.823 | 54.318 | -254.0556 | 3.98886 | 1 |
| 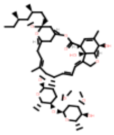 | 0 | 489 | C95H146O28 | 15.611 | 136 | 11 | -4.2995 | -10.164 | -34.54 | 22.559 | 33.725 | 33.959 | -225.1484 | 3.79653 | 1 |
| 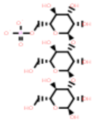 | 0 | 2466 | C18H33O19P | 15.613 | 69 | 19 | -5.6242 | -3.5132 | -21.42 | 0 | 39.149 | 19.472 | -118.0034 | 2.35885 | 1 |
| 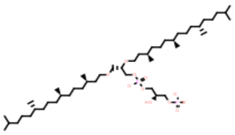 | 0 | 2035 | C46H94O11P2 | 15.617 | 152 | 43 | -4.5176 | -9.8753 | -46.66 | 17.02 | 31.072 | 31.823 | -206.1922 | 4.0584 | 1 |
| 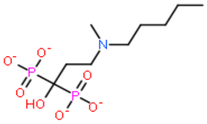 | 0 | 594 | 710 | 15.698 | 38 | 10 | -13.878 | -3.7045 | -16.69 | 0.7401 | 19.831 | 73.737 | -109.8879 | 1.37043 | 1 |
| 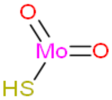 | 0 | 3004 | HMoO2S | 16.112 | 5 | 1 | -9.8646 | -0.3593 | -5.96 | 1.6803 | 12.12 | 55.414 | -5.155604 | 0.29242 | 1 |
| 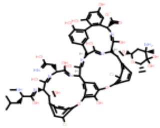 | 0 | 2497 | C60H65Cl2N9O20 | 16.287 | 155 | 21 | -8.2683 | -11.359 | -50.6 | 11.077 | 58.173 | 49.311 | -285.3415 | 4.15621 | 1 |
| 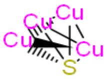 | 0 | 2833 | Cu4S | 16.553 | 5 | 0 | -4.0443 | -0.2003 | -3.735 | 0.0256 | 8.3666 | 36.916 | 10.994732 | 0.32245 | 1 |

| | | | | | | | | | | | | | | | |
|---|---|---|---|---|---|---|---|---|---|---|---|---|---|---|---|
| 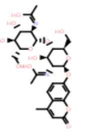 | 0 | 2470 | C26H34N2O13 | 16.782 | 75 | 14 | -5.0808 | -7.958 | -28.94 | 19.439 | 32.733 | 29.234 | -149.0122 | 2.23899 | 1 |
| 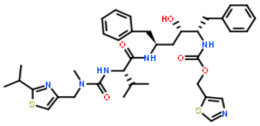 | 0 | 393 | C37H48N6O5S2 | 16.952 | 98 | 21 | -3.2194 | -8.9117 | -36.99 | 13.546 | 28.879 | 38.899 | -174.7091 | 2.67936 | 1 |
| 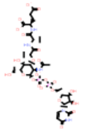 | 0 | 2056 | m | 17.162 | 97 | 24 | -14.509 | -5.2611 | -37.15 | 11.844 | 44.577 | 60.008 | -182.9092 | 3.32006 | 1 |
| 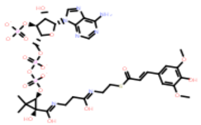 | 0 | 2861 | C32H44N7O20P3S | 17.583 | 103 | 21 | -12.459 | -6.8877 | -42.7 | 4.9682 | 46.951 | 69.13 | -218.412 | 3.21389 | 1 |
| 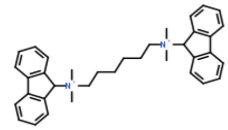 | 0 | 819 | C36H42N2 | 17.711 | 80 | 9 | 0 | -9.2759 | -25.3 | 7.5693 | 17.482 | 39.657 | -161.2023 | 2.21758 | 1 |
| 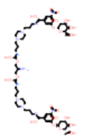 | 0 | 2301 | C51H79N11O22 | 18.435 | 163 | 39 | -13.102 | -9.6507 | -60.51 | 43.156 | 42.522 | 56.63 | -191.2659 | 4.48869 | 1 |
| 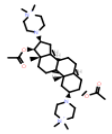 | 0 | 1170 | 1338 | 18.567 | 105 | 4 | -1.7458 | -10.148 | -21.14 | 16.3 | 22.58 | 33.05 | -166.4308 | 2.49343 | 1 |
| 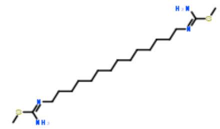 | 0 | 2675 | C18H38N4S2 | 18.817 | 62 | 15 | -3.4201 | -7.453 | -25.69 | 13.822 | 25.197 | 33.279 | -98.11567 | 2.23025 | 1 |
| 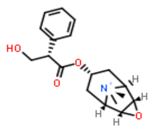 | 0 | 352 | m | 19.378 | 47 | 5 | -6.64 | -5.4643 | -22.68 | 53.477 | 17.429 | 21.756 | -118.034 | 1.49902 | 1 |
| 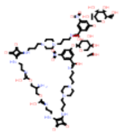 | 0 | 2603 | C63H91N15O26 | 19.733 | 195 | 45 | -15.727 | -11.318 | -63.97 | 44.425 | 51.496 | 58.782 | -240.8268 | 5.27349 | 1 |
| 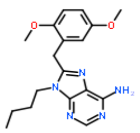 | 0 | 2098 | PU7 | 19.855 | 48 | 5 | -3.2167 | -7.0649 | -7.705 | 3.3885 | 19.561 | 31.406 | -115.8351 | 1.51896 | 1 |

| | | | | | | | | | | | | | | | |
|---|---|---|---|---|---|---|---|---|---|---|---|---|---|---|---|
| 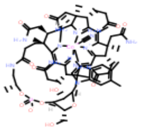 | 0 | 12 | C63H88CoN14O14P | 19.929 | 181 | 18 | -6.4655 | -8.4872 | -34.73 | 0 | 44.727 | 49.034 | -176.1554 | 3.15761 | 1 |
| 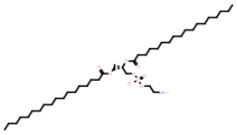 | 0 | 1730 | C41H82NO8P | 20.247 | 132 | 42 | -8.2731 | -10.435 | -41.65 | 27.6 | 32.055 | 36.396 | -207.0762 | 3.5585 | 1 |
| 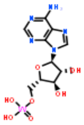 | 0 | 2112 | C10H14N5O7W | 20.421 | 37 | 8 | -6.732 | -2.6531 | -6.601 | 3.1197 | 21.228 | 35.211 | -28.99451 | 1.28881 | 1 |
| 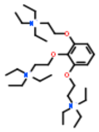 | 0 | 373 | 483 | 20.874 | 96 | 18 | -0.6672 | -10.23 | -30.28 | 9.3505 | 22.655 | 39.833 | -107.5023 | 2.46753 | 1 |
| 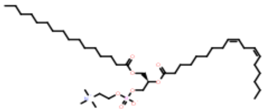 | 0 | 2048 | C42H80NO8P | 21.817 | 132 | 38 | -8.3139 | -10.276 | -52.76 | 28.088 | 37.748 | 52.324 | -252.691 | 3.76219 | 1 |
| 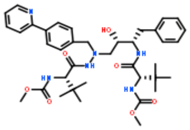 | 0 | 944 | C38H52N6O7 | 24.752 | 103 | 18 | -1.0218 | -10.404 | -45.84 | 22.076 | 34.808 | 45.452 | -184.7757 | 2.8383 | 1 |
| Fe | 0 | 1386 | 1592 | 25.26 | 1 | 0 | -3.2262 | 0.12086 | -1.633 | 0 | 1.966 | 50.757 | 12.195975 | -0.1226 | 1 |
| 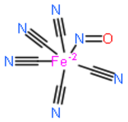 | 0 | 219 | C5FeN6O | 27.886 | 13 | 2 | -1.8503 | -0.6519 | 0.2925 | 1.0564 | 13.833 | 31.065 | -1.491458 | 0.61214 | 1 |
| 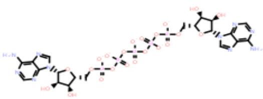 | 0 | 1503 | C20H29N10O22P5 | 28.291 | 81 | 20 | -18.013 | -4.2537 | -38.11 | 9.9149 | 40.715 | 100.51 | -179.2837 | 2.56836 | 1 |
| Zn | 0 | 1387 | 1593 | 28.846 | 1 | 0 | -3.0732 | 0.12086 | -1.634 | 0 | 1.964 | 55.607 | 15.543561 | -0.1226 | 1 |
| 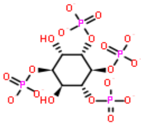 | 0 | 1638 | C6H16O18P4 | 28.947 | 36 | 10 | -9.5815 | -2.1793 | -12.43 | 0 | 26.77 | 62.13 | -135.275 | 1.77386 | 1 |

| | | | | | | | | | | | | | | |
|---|---|---|---|---|---|---|---|---|---|---|---|---|---|---|
| 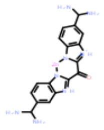 | 0 | 3110 | C17H18N8OZn | 29.436 | 45 | 6 | -9.3057 | -3.1582 | -23.71 | 3.759 | 29.678 | 76.801 | -50.5647 | 1.40011 | 1 |
| 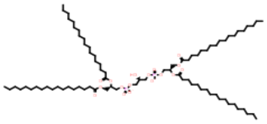 | 0 | 3097 | CL(18:0/18:0/18:0/18:0) | 29.508 | 256 | 83 | -14.207 | -14.981 | -89 | 60.395 | 50.699 | 61.251 | -329.7323 | 6.09987 | 1 |
| 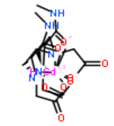 | 0 | 120 | C16H29GdN5O8 | 29.741 | 56 | 0 | -3.2197 | -3.6295 | -14.86 | 0 | 13.832 | 67.887 | -46.93496 | 1.18098 | 1 |
| 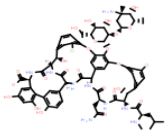 | 0 | 400 | C66H75Cl2N9O24 | 30.687 | 175 | 19 | -14.109 | -10.726 | -42.69 | 36.74 | 65.534 | 51.002 | -280.4023 | 5.04915 | 1 |
| 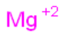 | 0 | 1197 | Mg | 30.869 | 1 | 0 | -3.5009 | 0.12086 | -1.34 | 0 | 1.9583 | 59.873 | 5.634181 | -0.1226 | 1 |
| 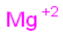 | 0 | 1196 | MgO | 31.677 | 1 | 0 | -3.3601 | 0.12086 | -1.36 | 0 | 1.9583 | 60.588 | 8.764312 | -0.1226 | 1 |
| 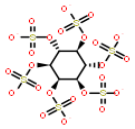 | 0 | 1455 | C6H12O24S6 | 33.496 | 42 | 12 | -8.2599 | -1.6804 | 13.433 | 3.4419 | 25.874 | 20.328 | -61.24151 | 1.56666 | 1 |
| 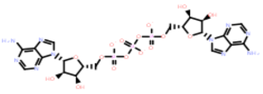 | 0 | 1478 | C20H27N10O16P3 | 35.202 | 73 | 16 | -16.09 | -4.4185 | -19.2 | 15.24 | 47.734 | 65.942 | -142.5905 | 2.30784 | 1 |
| 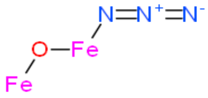 | 0 | 1774 | Fe2N3O | 37.712 | 6 | 2 | -2.7654 | -0.1554 | -6.559 | 5.5395 | 9.3743 | 60.33 | -3.888562 | 0.42418 | 1 |
| 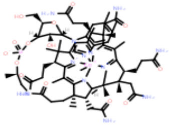 | 0 | 3265 | C63H91CoN13O14P | 39.874 | 183 | 18 | -10.762 | -9.6577 | -27.01 | 0 | 41.616 | 88.747 | -218.1281 | 3.5716 | 1 |
| 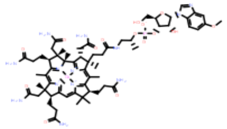 | 0 | 2387 | C61H85CoN13O15P | 39.92 | 176 | 28 | -8.213 | -9.4669 | -47.44 | 0 | 32.303 | 111.3 | -147.3701 | 2.68351 | 1 |

| | | | | | | | | | | | | | | | |
|---|---|---|---|---|---|---|---|---|---|---|---|---|---|---|---|
| 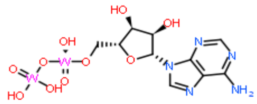 | 0 | 1936 | C10H15N5O10W2 | 43.235 | 42 | 11 | -8.1162 | -3.7592 | -15.38 | 0 | 32.486 | 76.825 | -63.87192 | 1.39897 | 1 |
| 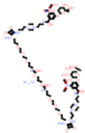 | 0 | 2299 | C79H123N15O32 | 44.492 | 249 | 67 | -9.6665 | -12.535 | -69.57 | 45.19 | 53.013 | 60.363 | -329.0207 | 5.45746 | 1 |
| 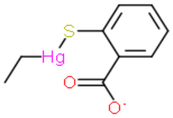 | 0 | 2445 | EMT | 48.267 | 22 | 3 | -0.7861 | -1.0798 | -2.072 | 0 | 10.705 | 65.488 | -14.23601 | 0.99869 | 1 |
| 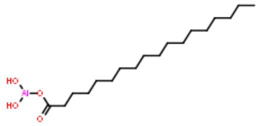 | 0 | 1195 | C18H37AlO4 | 54.548 | 60 | 19 | -1.392 | -7.1955 | -20.61 | 0 | 22.268 | 83.942 | -104.6182 | 1.85736 | 1 |
| 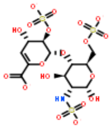 | 0 | 2064 | C12H15NO19S3 | 67.697 | 50 | 13 | -5.378 | -2.7757 | 19.627 | 12.069 | 28.712 | 40.85 | -19.77316 | 1.1619 | 1 |
| 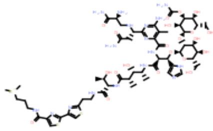 | 0 | 184 | C55H84N17O21S3 | 71.295 | 180 | 41 | -3.8914 | -3.6531 | 31.401 | 20.724 | 4.5958 | 17.735 | 35.334934 | 1.09533 | 1 |
| 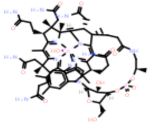 | 0 | 95 | C62H89CoN13O15P | 105.04 | 181 | 19 | -7.2961 | -8.2487 | 0.8137 | 10.713 | 43.762 | 117.52 | -96.49641 | 3.20061 | 1 |
| 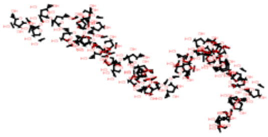 | 0 | 525 | 638 | 132.11 | 801 | 265 | -34.728 | -18.706 | -72.04 | 60.447 | 92.419 | 60.543 | -463.2307 | 10.2604 | 1 |
| 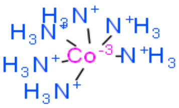 | 0 | 3025 | H18CoN6 | 140.85 | 25 | 0 | -5.3244 | -0.2567 | -8.288 | 0 | 22.795 | 220.74 | 3.487065 | 0.45852 | 1 |
| 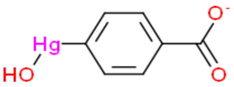 | 0 | 1460 | C7H6HgO3 | 162.2 | 16 | 3 | -6.1037 | -2.2928 | -3.01 | 0 | 10.793 | 261.04 | -25.59138 | 0.62758 | 1 |
| 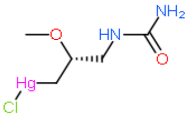 | 0 | 421 | 534 | 164.78 | 22 | 6 | -1.0503 | -2.7414 | 27.98 | 0 | 5.1067 | 202.83 | 79.780006 | 0.34576 | 1 |

| | | | | | | | | | | | | | | | |
|---|---|---|---|---|---|---|---|---|---|---|---|---|---|---|---|
| 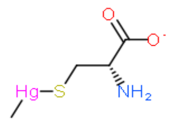 | 0 | 2461 | C4H9HgNO2S | 173.41 | 17 | 5 | -1.4315 | -2.1667 | -3.237 | 0 | 13.542 | 255.4 | -30.5468 | 0.73975 | 1 |
| 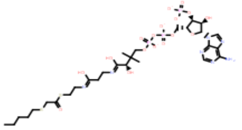 | 0 | 3083 | C28H48N7O17P3S2 | 197.5 | 101 | 29 | -9.2302 | -6.6777 | 90.521 | 9.699 | 40.549 | 122.98 | 111.24215 | 2.52339 | 1 |
| 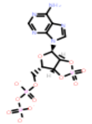 | 0 | 2381 | C10H13N5O12P2V | 250.9 | 40 | 6 | -4.3231 | -3.4263 | 0.5207 | 358.58 | 35.377 | 27.78 | -54.54076 | 1.41949 | 1 |
| 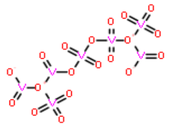 | 0 | 2400 | O19V7 | 329.88 | 26 | 10 | -4.0591 | -0.6589 | -7.871 | 4.1855 | 26.159 | 485.35 | -7.817133 | 1.0179 | 1 |
| 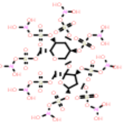 | 0 | 257 | C11H28Al8O51S8 | 459.37 | 106 | 52 | -10.925 | -3.0936 | 3.3275 | 0 | 44.07 | 638.64 | -32.02237 | 3.09319 | 1 |
| 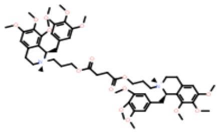 | 0 | 1006 | C56H78N2O16 | 959.63 | 152 | 15 | -1.5463 | -12.358 | 836.99 | 14.184 | 17.689 | 157.66 | 1862.7804 | 0.97074 | 1 |
