# Supplementary figures and images for: Potent β-lactam-based tyrosyl-DNA phosphodiesterase 1 inhibitors identified by a virtual screen
Source: Sci Rep. 2025 Jul 21;15:26510. doi: 10.1038/s41598-025-12503-8 (PMC12280126; doi:10.1038/s41598-025-12503-8)

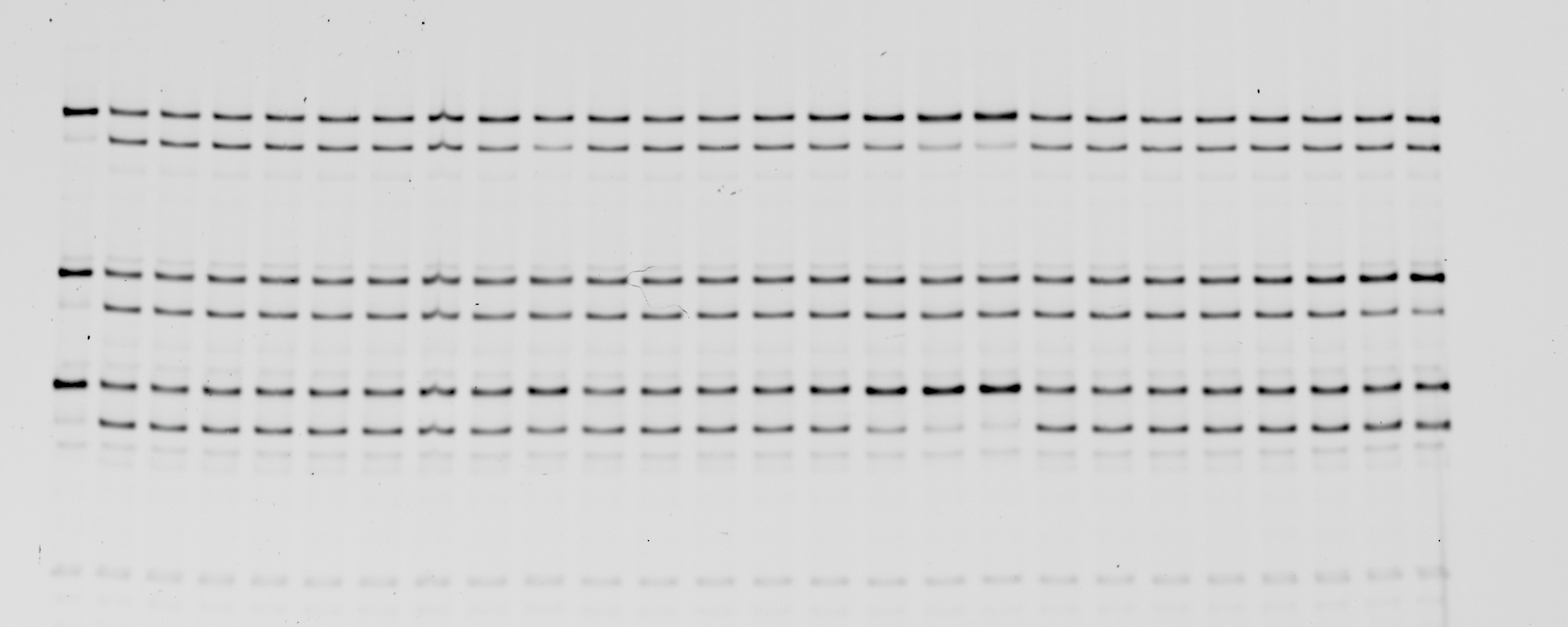

Supplement: Supplementary file 2 — Supplementary Material 2 [file 41598_2025_12503_MOESM2_ESM.tif]

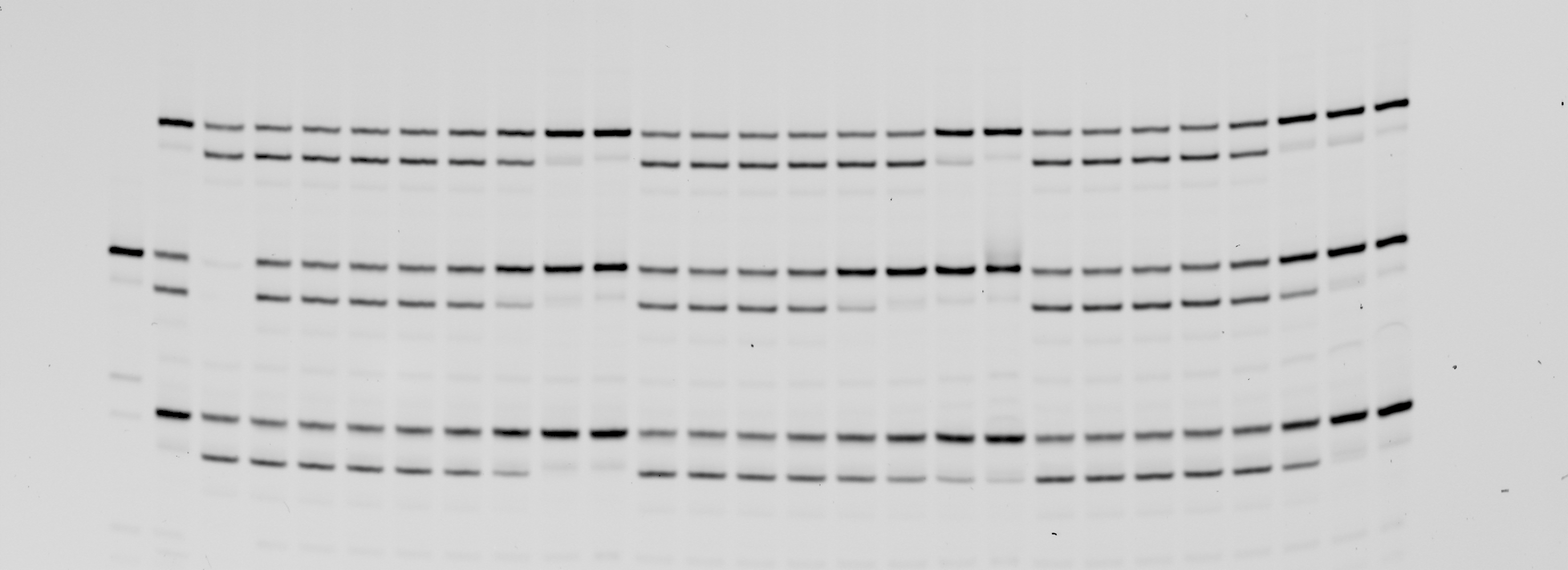

Supplement: Supplementary file 3 — Supplementary Material 3 [file 41598_2025_12503_MOESM3_ESM.tif]

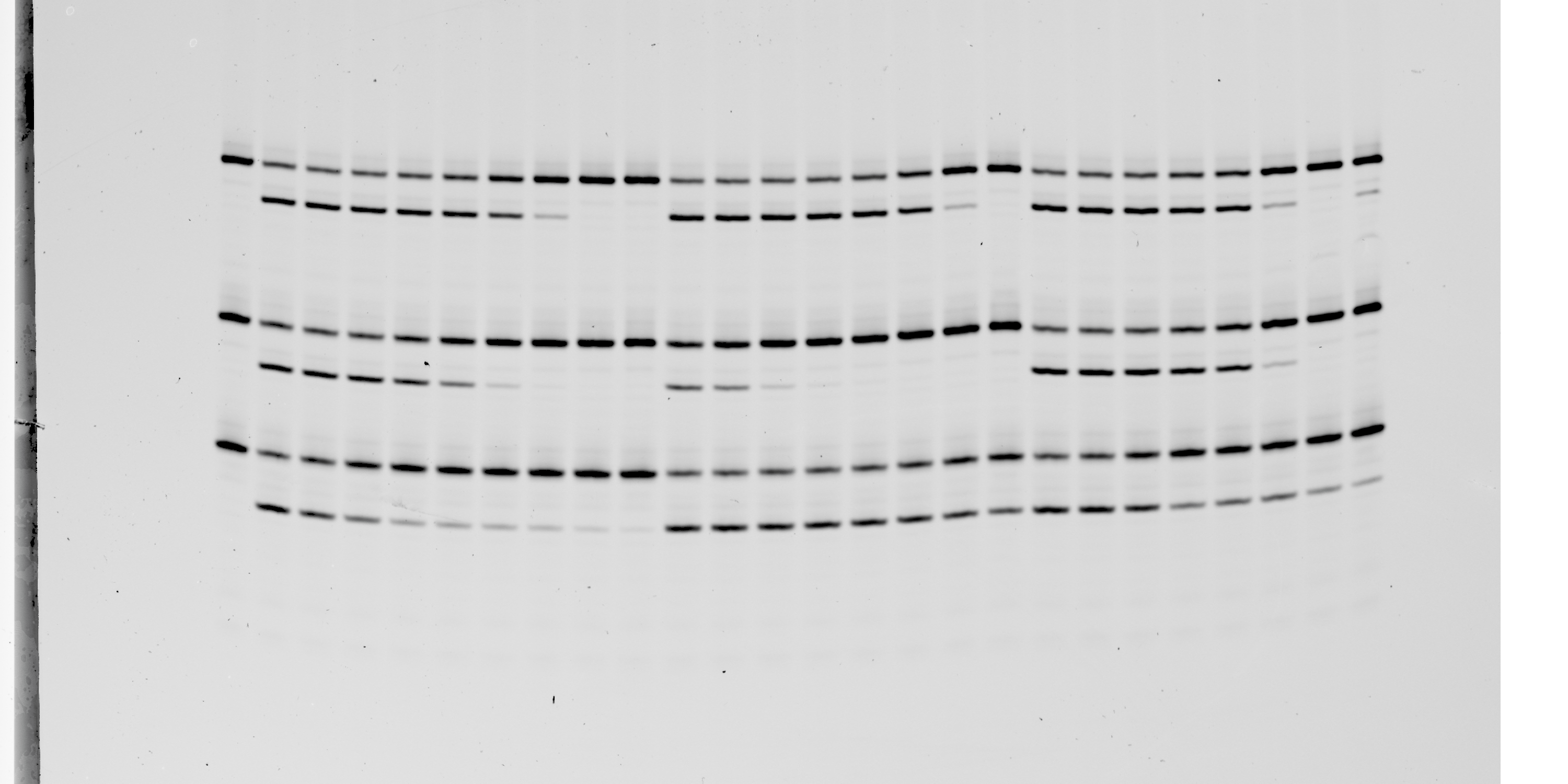

Supplement: Supplementary file 4 — Supplementary Material 4 [file 41598_2025_12503_MOESM4_ESM.tif]

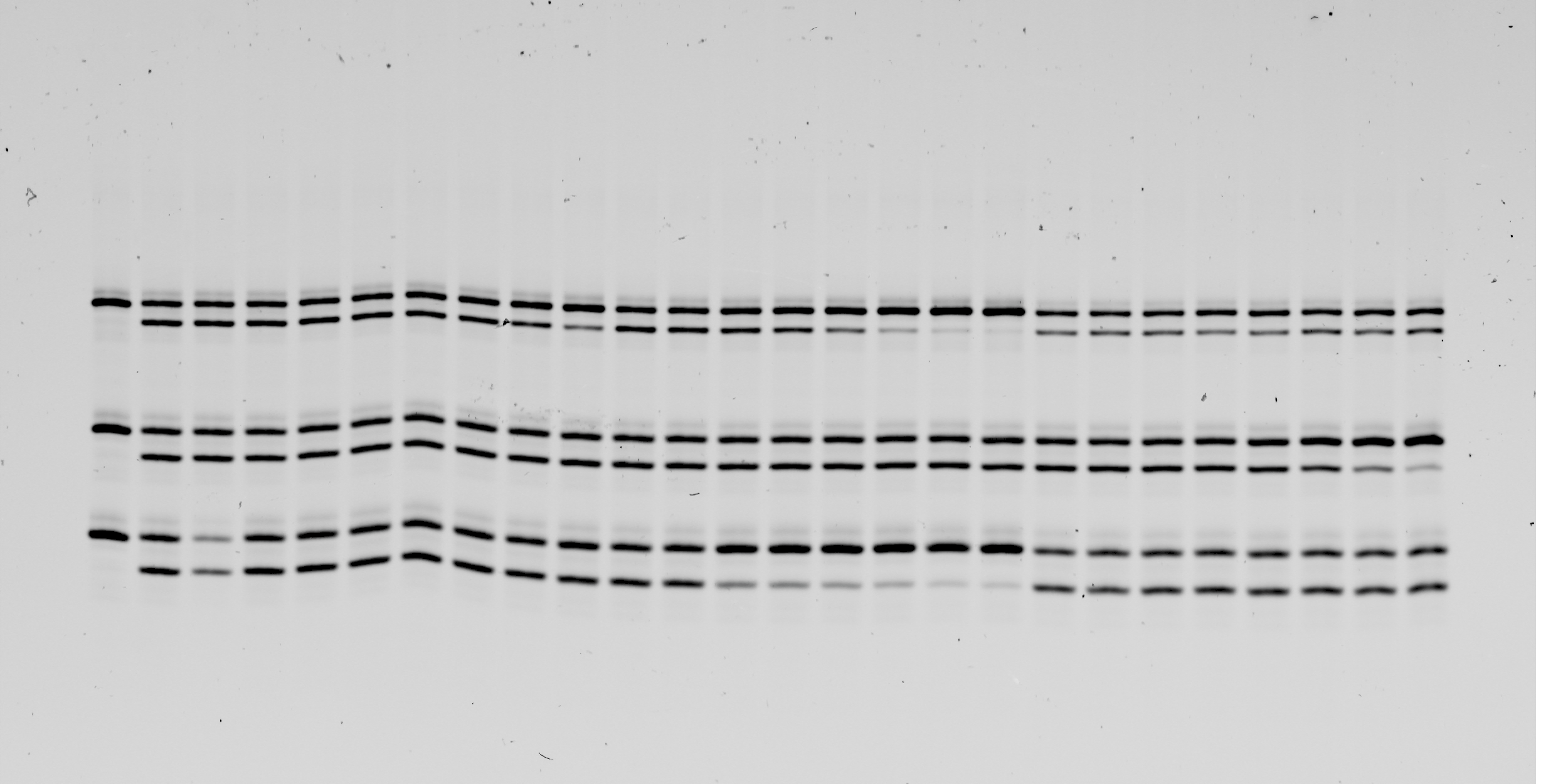

Supplement: Supplementary file 5 — Supplementary Material 5 [file 41598_2025_12503_MOESM5_ESM.tif]

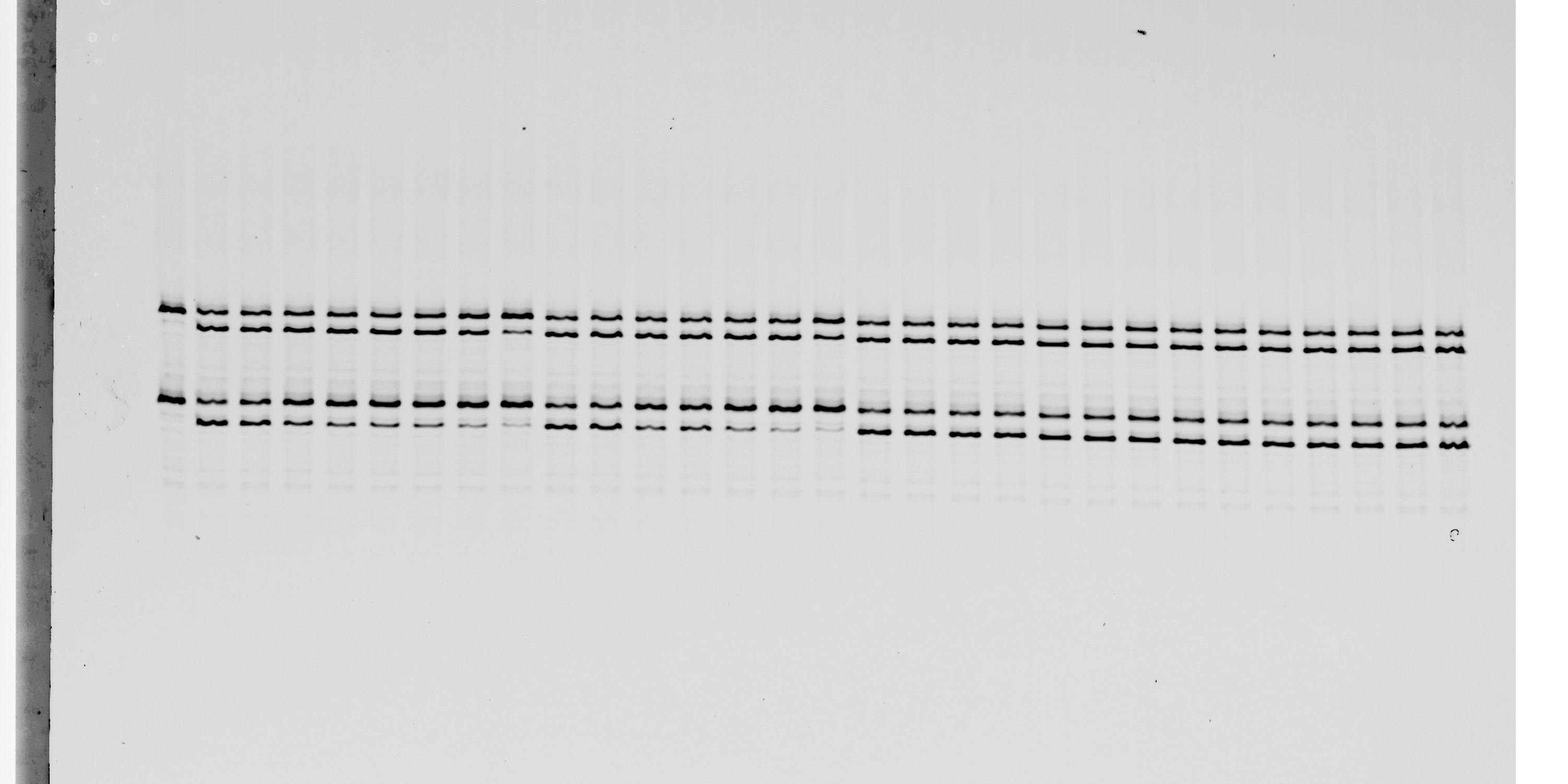

Supplement: Supplementary file 6 — Supplementary Material 6 [file 41598_2025_12503_MOESM6_ESM.tif]
